# Complex Genotype Mixtures Analyzed by Deep Sequencing in Two Different Regions of Hepatitis B Virus

**DOI:** 10.1371/journal.pone.0144816

**Published:** 2015-12-29

**Authors:** Andrea Caballero, Josep Gregori, Maria Homs, David Tabernero, Carolina Gonzalez, Josep Quer, Maria Blasi, Rosario Casillas, Leonardo Nieto, Mar Riveiro-Barciela, Rafael Esteban, Maria Buti, Francisco Rodriguez-Frias

**Affiliations:** 1 Liver Pathology Unit, Departments of Biochemistry and Microbiology, Hospital Universitari Vall d'Hebron, Universitat Autònoma de Barcelona (UAB), Barcelona, Spain; 2 Centro de Investigación Biomédica en Red de Enfermedades Hepáticas y Digestivas (CIBERehd), Instituto de Salud Carlos III, Madrid, Spain; 3 Liver Diseases Unit, Vall d’Hebron Research Institute, Barcelona, Spain; 4 Roche Diagnostics S.L., Sant Cugat del Vallès, Spain; 5 Liver Unit, Hospital Universitari Vall d'Hebron, Universitat Autònoma de Barcelona (UAB), Barcelona, Spain; University of Sydney, AUSTRALIA

## Abstract

This study assesses the presence and outcome of genotype mixtures in the polymerase/surface and X/preCore regions of the HBV genome in patients with chronic hepatitis B virus (HBV) infection. Thirty samples from ten chronic hepatitis B patients were included. The polymerase/surface and X/preCore regions were analyzed by deep sequencing (UDPS) in the first available sample at diagnosis, a pre-treatment sample, and a sample while under treatment. HBV genotype was determined by phylogenesis. Quasispecies complexity was evaluated by mutation frequency and nucleotide diversity. The polymerase/surface and X/preCore regions were validated for genotyping from 113 GenBank reference sequences. UDPS yielded a median of 10,960 sequences per sample (IQR 16,645) in the polymerase/surface region and 11_,_595 sequences per sample (IQR 14,682) in X/preCore. Genotype mixtures were more common in X/preCore (90%) than in polymerase/surface (30%) (p<0.001). On X/preCore genotyping, all samples were genotype A, whereas polymerase/surface yielded genotypes A (80%), D (16.7%), and F (3.3%) (p = 0.036). Genotype changes in polymerase/surface were observed in four patients during natural quasispecies dynamics and in two patients during treatment. There were no genotype changes in X/preCore. Quasispecies complexity was higher in X/preCore than in polymerase/surface (p = 0.004).

The results provide evidence of genotype mixtures and differential genotype proportions in the polymerase/surface and X/preCore regions. The genotype dynamics in HBV infection and the different patterns of quasispecies complexity in the HBV genome suggest a new paradigm for HBV genotype classification.

## Introduction

Hepatitis B virus (HBV) infection is a global health problem. Currently, around 240 million people are chronically infected by HBV, which confers a high risk of developing liver cirrhosis and hepatocellular carcinoma (HCC) [1].

HBV, a member of the Hepadnaviridae family, has a small (3.2 kb) partially double-stranded DNA genome with four highly overlapping open reading frames (ORFs): polymerase, surface, Core, and X [2]. To date, HBV has been classified into eight well-characterized (A-H) and two putative (I-J) genotypes (Gen/A-I) by >8% divergence in the entire genomic sequence. Genotypes are further divided into subgenotypes when genome divergence is 4% to 8%. The geographic and ethnic distribution of HBV genotypes and subgenotypes has been well characterized [3]. In the population of Spain, approximately 90% of HBV chronic carriers are infected by genotype A or D, and 6% by genotype F [4]. Several studies have reported associations of HBV genotype and subgenotype with HBeAg seroconversion status, clinical outcome, and prognosis [5–7]. In addition, HBV genotype has an impact on the therapeutic response to interferon and nucleos(t)ide analogues (NUCs) [8].

The most conclusive method for HBV genotyping is phylogenetic analysis of the nucleotide sequences of the whole HBV genome, although this is not performed in routine diagnostics. Instead, genotyping is done by sequencing a representative part of the genome. There is no consensus as to which region of the genome should be used for this purpose. Currently, the surface and polymerase regions are the ones most widely used, mainly because commercial line probe assays (LiPA) hybridize at the polymerase region [9].

Recombination events have been described between different HBV genotypes in HIV-positive patients [10], in integrated sequences from HCC patients [11], and in 30% of the 3300 HBV genome sequences available in public databases [12]. Intergenotype recombinations tend to have the same geographical distributions as their parent genotypes [13]. Inconsistencies in the local phylogenies of different fragments of the HBV genome may be a source of HBV genotype/subgenotype misclassification [14]. LiPA genotyping identified mixed genotype infections in 8% of HBV-infected individuals from Central and Eastern Europe [15]. In another study, LiPA documented mixed genotype infections in 22% of patients [16]. Nonetheless, detection of genotype mixtures or minor strains may be limited by the technology used [17]. Therefore, we determined the presence of HBV genotype mixtures by ultra-deep pyrosequencing (UDPS), which enables analysis of thousands of clonal sequences, 400 nucleotides (nt) in length. UDPS has been extensively used for detecting and quantifying low-frequency variants, for genotyping, and for investigating the viral quasispecies in HBV and other viruses [18–24]. In this study, we aimed to quantify genotype mixtures and evaluate changes occurring in the genotypic pattern of the HBV quasispecies in the absence and presence of nucleoside analogue treatment using UDPS. Two regions of the viral genome were selected for the study: a region including the polymerase/surface genes, and another essential region for regulation of HBV replication that includes the 5’ end of the X gene and the preCore region.

## Patients and Methods

### Patients

This is a retrospective study approved by the Ethics Committee of Vall d'Hebron Research Institute, and including sequential samples from chronic hepatitis B patients, who gave written consent for participation. All patients were born in Spain, had failed lamivudine (LMV) therapy, had complete clinical documentation, and tested negative for hepatitis delta virus (HDV), hepatitis C virus (HCV), and human immunodeficiency virus (HIV). Ten chronically infected HBV patients (Pt) were randomly selected for the study. Three samples per patient were analyzed: one sample obtained at the diagnosis (baseline), one taken before starting LMV, and the third taken at the time of partial virological response [25]. However, in two patients (Pt 5 and 7), a sample at viral breakthrough was included, because the partial virological response sample was not available. Clinical, biochemical, virological, and serological markers in the ten patients are summarized in Table 1. Two patients were HBeAg-positive at baseline (Pts 7 and 10). HBeAg loss occurred in one patient during antiviral treatment (Pt 10), and one patient (Pt 5) was HBeAg-negative at baseline and HBeAg-positive at the second and third sample.

**Table 1:** Baseline biochemical and serological markers of the ten patients included in the study.

Pt	Sex	First sample at diagnosis (baseline)					Second sample (before starting treatment)					Third sample (under treatment)				
		Age, years	ALT, IU/mL	HBV DNA, IU/mL	HBeAg	Gen	Months after 1st sample	ALT, IU/mL	HBV DNA, IU/mL	HBeAg	Gen	Months after 2nd sample	ALT, IU/mL	HBV DNA, IU/mL	HBeAg	Gen
1	M	51	105	3.13E+06	N	A	7	364	2.32E+07	N	A	25	38	1.64E+04	N	A
2	M	56	391	2.9E+07	N	DF	25	656.9	1.16E+08	N	A	13	19.5	6.67E+04	N	A
3	M	30	46.4	1.68E+08	N	A	20	101.8	9.67E+07	N	A	14	34.7	1.18E+04	N	A
4	M	29	116	1,37E+03	N	A	28	70.7	8.64E+06	N	D	5	49.5	2.93E+04	N	D
5	M	56	75	2.65E+05	N	A	7	60	7.87E+08	P	A	19	55.9	1.03E+09	P	A
6	M	45	92	7.80E+08	N	A	36	129	2.78E+09	N	A	32	38	2.38E+03	N	A
7	F	20	59	1.66E+10	P	A	4	60	2.23E+09	P	A	14	75	2.36E+09	P	A
8	M	32	78	9.64E+04	N	AF	16	242	4.42E+05	N	A	22	57	3.17E+04	N	A
9	M	48	65	6.12E+05	N	A	43	109	7.43E+06	N	D	11	50	9.73E+03	N	A
10	M	35	262	9.66E+08	P	D	17	137	8.99E+06	P	D	18	93.8	1.31E+04	N	A

Pt; Patient, M; Male, F; Female, P; Positive, N; Negative, Gen; genotype determined by LiPA

### Serological and virological determinations

Serological markers for HBV (HBsAg, HBeAg, and anti-HBe), HCV (anti-HCV), HDV (anti-HDV), and HIV (anti-HIV) were tested by commercial enzyme immunoassays. HBV-DNA was quantified by real-time PCR with a detection limit of 20 IU/mL (COBAS TaqMan HBV V2.0, Roche Diagnostics GmbH, Mannheim, Germany). HBV genotype was determined by LiPA (INNO-LiPA, Innogenetics, Ghent, Belgium). This method analyzes a fragment covering the polymerase and surface regions (nucleotide positions: nt 456–798), detects mixtures at proportions of 20%, and is considered to have a genotyping accuracy of >99% [9].

### HBV genome regions analyzed by UDPS

**Fig 1:**
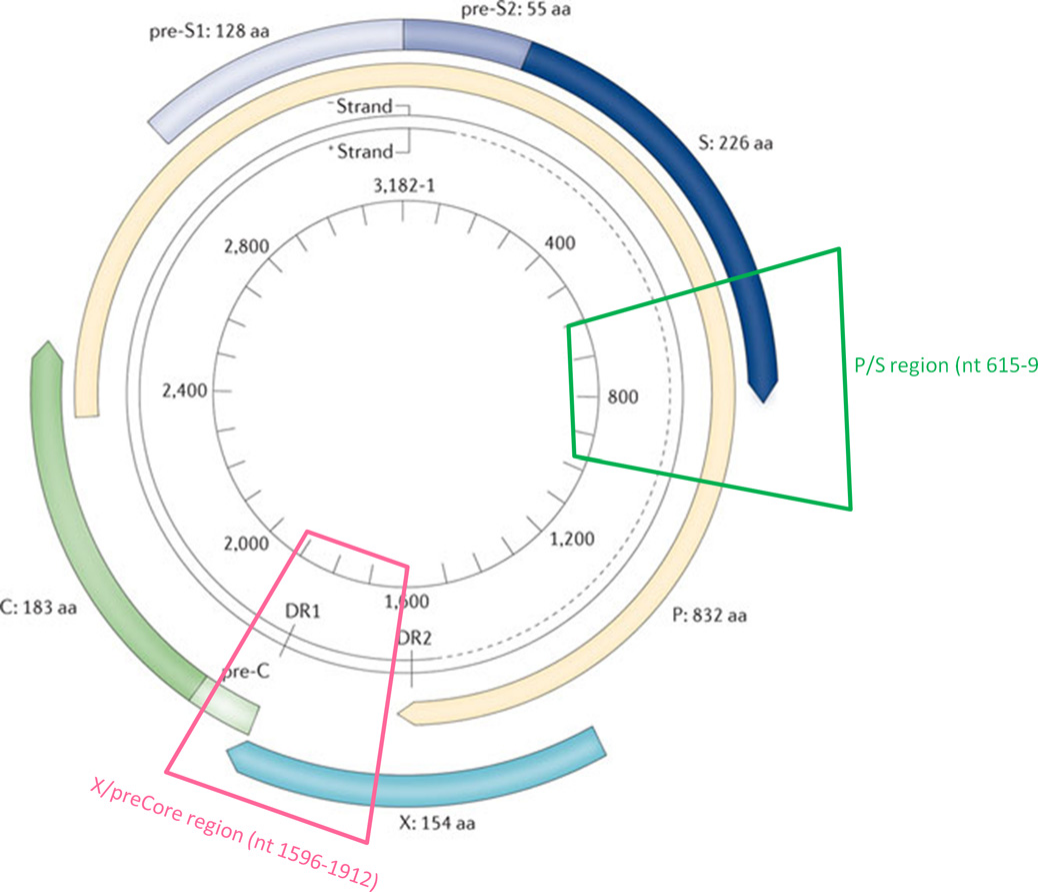
Regions of the HBV genome amplified for the present study: P/S is indicated in green and X/preCore in pink.

HBV-DNA was extracted from serum by QIAampMiniSpin columns (QIAampDNA Mini Kit, QIAGEN, Hilden, Germany), according to the manufacturer’s instructions. Two HBV genomic fragments were selected for UDPS (GS FLX, 454 Life Sciences, Roche) (Fig 1). One was a 355 bp (nt 615–969) fragment including the reverse transcriptase domain and overlapping the surface gene (P/S region); P/S includes 183 bp of the region amplified by LiPA. The other was a 356 bp (nt 1580–1936) fragment including the 3’-end of the X gene and the 5’-end of the preCore/Core gene (X/preCore region). The X/preCore region was chosen because it is clinically relevant, as it includes the ENHII region, the main motifs involved in HBV replication and regulation, and the sequence where synthesis of the HBV DNA minus strand is initiated (DR1). In addition, it is likely to be a region in which the viral quasispecies would show high complexity. Two PCRs with high-fidelity polymerase (Pfu Ultra-II, Stratagene, La Jolla, United States) were performed for P/S and X/preCore. PCR products were isolated from 1.5% agarose gel and their quality and quantity were checked. A detailed description of the reactions, concentrations, and primer sequences used is provided in supplementary materials and methods in S1 Protocol. Amplicons were pooled to a concentration of 2x10^6^ molecules. This working solution was enriched with capture beads for forward and reverse clonal amplification (emPCR kits II and III, 454 Life Sciences). UDPS was carried out on the FLX Genome Sequencer system (454Life Sciences).

### UDPS data treatment

UDPS sequences were demultiplexed and primers were trimmed. After a quality filter step, sequences covering the full amplicon and common to both strands were established as consensus haplotypes, as described elsewhere [26,27]. Details of the procedure are given in the S1 Protocol. Computations were made in the R language and platform [28] using an in-house-developed pipeline, with the help of functions provided in the Biostrings [29] and APE [30] packages.

The UDPS sequencing data from the samples analyzed in this study have been submitted to the GenBank SRA database. The BioProject accession number is PRJNA291355 and BioSample accession numbers are included in S1 Table.

### Genotyping and quasispecies complexity indexes

The consensus haplotypes of each sample were clustered in each region using identity thresholds of 96% (P/S) and 97% (X/preCore) (Genotyping in S1 Protocol). The centroid of each cluster was taken as the most abundant haplotype in the cluster. The Clustal Omega program was used to align the cluster centroids with a set of reference sequences from GenBank. Genotyping was done by distance-based discriminant analysis (DB rule) [31,32], which takes into account the intraclass variability of each genotype. Genetic distances were computed according to the Kimura-80 model [33]. Visualization of genetic distances between sequences was provided by UPGMA trees [34] and Multidimensional Scaling (MDS) plots [35].

HBV quasispecies (QA) complexity in the two regions was quantified using the mutation frequency (Mf) and nucleotide diversity (Pi) indexes [23,26]. Possible differences between the second and first sample were considered to result from the natural dynamics of the quasispecies; that is, changes occurring in haplotype fitness without external pressure (eg, antiviral treatment), but under intrinsic factors, such as the host immune response. Genotype mixtures were defined as the presence of haplotypes classified as belonging to different genotypes in a single quasispecies sample. As only two fragments of the HBV genome were studied, the information obtained did not suffice to determine whether this was the result of a mixed infection, the presence of recombinants, or both these circumstances. Whatever the case, all haplotypes detected were consistently classified, and with high confidence, using a collection of reference sequences for each HBV genotype, as described in Genotyping in S1 Protocol.

### Statistical analysis

Statistical analyses were carried out with IBM SPSS 20 (SPSS Inc., Chicago, USA). Categorical data were tested by the Fisher exact test, and independent variables were compared using the Mann-Whitney *U* test. Significance was set at p≤0.05.

## Results

### Validation of P/S and X/preCore regions for genotyping

To check the capacity to differentiate between genotypes in the two regions analyzed, a set of whole HBV genome sequences from GenBank was evaluated by the leave-one-out method using the DB rule discriminant analysis. Each whole genome sequence in the set was classified using the remaining sequences. The genotyping capacity of these reference sequences was explored using UPGMA trees and plotting on the first three components of a multidimensional scaling analysis of the matrix of genetic distances between pairs of reference sequences. All sequences could be perfectly classified except for the genotype J sequence in GenBank (S1 File).

The set of reference sequences was multiple-aligned and trimmed to positions 615 to 969, covering the P/S amplicon (S2 File), and positions 1596 to 1912, covering the X/preCore region (S3 File). Sequences that were equal after trimming were removed. The consensus P/S and X/preCore haplotypes were clustered at identities of 96% and 97%, respectively. The P/S region showed high genotyping capacity, despite the limited amplicon length. Only two Gen/C reference sequences were misclassified as Gen/A using the P/S region (S2 File). In contrast, the X/preCore regions showed clear intermixing of Gen/D and E and Gen/C and I (S3 File).

### Genotyping

Routine HBV genotyping by LiPA defined seven cases of Gen/A and one of Gen/D. Two genotype mixtures were detected by LiPA in the first sample (Pt 2, Gen/D and F; Pt 8, Gen/A and F) (Table 1).

#### UDPS Genotyping in the P/S region

A total of 379,438 sequences were obtained from the P/S region (median 10,960 reads, IQR 16,647). P/S showed high discriminating capacity for the eight main HBV genotypes (Gen/A-H) (S2 File). Among the 379,438 sequences analyzed, three different genotypes were observed (A, D, and F).

Twenty-one of the 30 samples (70%) showed single-genotype infection: 17 samples (81%) were Gen/A and four (19%) Gen/D (Table 2). Genotype mixtures were observed in nine samples (30%): the A/D mixture in five (56%) and the A/D/F mixture in four (44%).

**Table 2:** Percentage of HBV genotypes obtained from the polymerase/surface (P/S) and X/preCore regions of the ten patients. Three samples per patient were analyzed: the first available at diagnosis (1^st^), the second before starting treatment (2^nd^), and the third under treatment (3^rd^).

Patient	Samples analyzed	P/S region			X/preCore region		
		A (%)	D (%)	F (%)	A (%)	DE* (%)	F (%)
	1^st^	93.0	3.6	3.4	92.2	5.3	2.5
**1**	2^nd^	100.0	0	0	98.9	1.1	0
	3^rd^	100.0	0	0	84.5	14.4	1.1
	1^st^	6.0	16.2	77.8	86.0	8.5	5.5
**2**	2^nd^	99.0	1	0	86.9	8.4	4.6
	3^rd^	96.4	3.6	0	93.5	5.4	1.1
	1^st^	98.5	1.5	0	66.9	30.5	2.6
**3**	2^nd^	100.0	0	0	93.5	4.3	2.2
	3^rd^	100.0	0	0	83.6	15.0	1.3
	1^st^	100.0	0	0	87.8	10.7	1.5
**4**	2^nd^	0	100.0	0	86.9	9.9	3.2
	3^rd^	9.4	89.9	0.7	97.6	2.1	0.3
	1^st^	100.0	0	0	100.0	0	0
**5**	2^nd^	100.0	0	0	87.5	12.2	0.3
	3^rd^	100.0	0	0	90.0	7.4	2.6
	1^st^	100.0	0	0	98.9	1.1	0
**6**	2^nd^	100.0	0	0	97.1	2.1	0.9
	3^rd^	100.0	0	0	82.9	9.9	7.2
	1^st^	100.0	0	0	93.2	2.6	4.3
**7**	2^nd^	100.0	0	0	95.9	2.2	1.8
	3^rd^	100.0	0	0	71.7	9.9	18.4
	1^st^	77.0	3.0	19.9	89.4	9.7	0.9
**8**	2^nd^	93.0	7.0	0	86.5	10.3	3.2
	3^rd^	100.0	0	0	90.4	8.3	1.3
	1^st^	87.2	12.8	0	86.8	7.9	5.4
**9**	2^nd^	0	100	0	88.7	10.2	1.1
	3^rd^	100.0	0	0	76.5	19.3	4.2
	1^st^	0	100	0	83.3	15.2	1.6
**10**	2^nd^	0	100	0	94.5	1.1	4.4
	3^rd^	100.0	0	0	89.2	4.0	6.8

* Results are presented as DE because the X/preCore region showed an intermixing between Gen/D and E (S3 File).

Five of the ten (50%) patients (Pts 1, 2, 3, 8, and 9) showed HBV genotype mixtures in the first sample. Genotype mixtures were also detected in two patients (Pts 2 and 8) before starting treatment (second sample) and in two patients (Pt 2 and 4) under treatment (third sample). Four patients had genotype mixtures in percentages theoretically detectable by LiPA, but the method only detected mixtures in two patients (Pts 2 and 8). Sequence coverage was low in the treatment samples from Pts 9 and 10 (53 and 15, respectively); hence, a possible presence of mixtures was not excluded. Of note, these samples also yielded low coverage results in the P/S region. UPGMA trees and MDS plots of the first three most common haplotypes in the P/S region per sample are represented in S4 File with the reference sequences.

#### UDPS Genotyping in the X/preCore region

In total, 944,184 sequences were obtained in the X/preCore region (median 11,595 reads, IQR 14,682). X/preCore showed intermixing between Gen/D and E and Gen/C and I (S3 File). Gen/E is restricted to Africa [36], and none of the ten patients included in the study were from Africa; however, to be precise, the Gen/D results are presented as Gen/DE. None of the sequences were Gen/C, so GenC/I intermixing in the X/preCore region did not affect our study. Among the 944,184 sequences analyzed, three different genotypes were observed (A, DE, and F).

Gen/A infection alone was detected in only one baseline sample (Pt 5). The remaining 29 samples (96.7%) (baseline, second, and third samples from all patients) showed HBV genotype mixtures (Table 2). Triple mixtures (A/DE/F) were observed in 27 samples (90%) and double mixture (A/DE) in two samples (10%). UPGMA trees and MDS plots of the first three most common haplotypes in the X/preCore region per sample with the reference sequences are shown in S5 File.

#### P/S region vs. X/preCore region for genotyping

**Fig 2:**
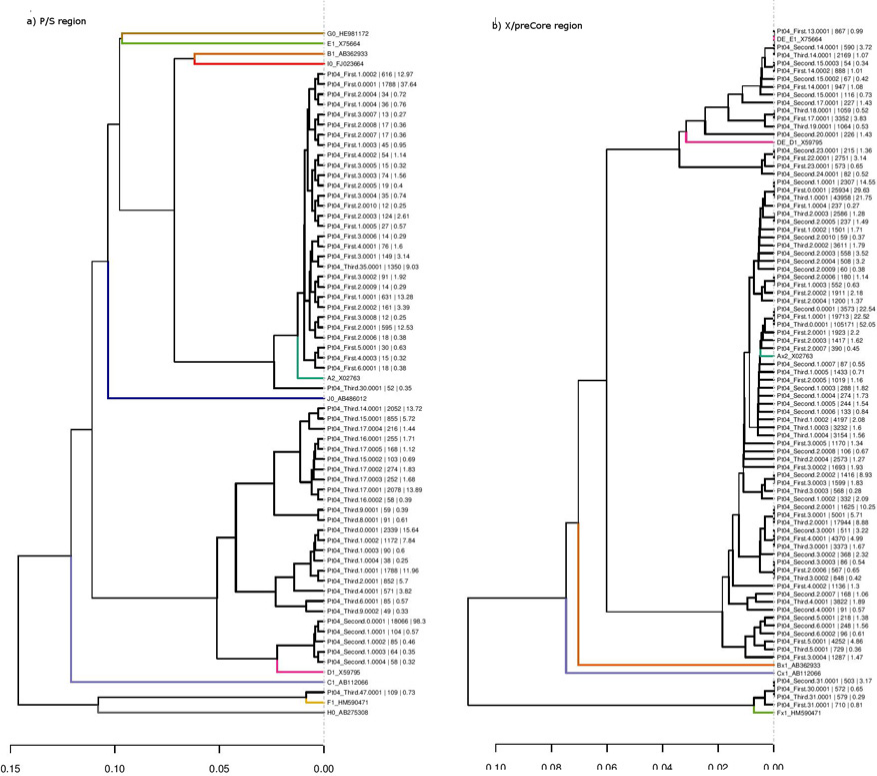
UPGMA tree obtained from the polymerase/surface (a) and the X/preCore (b) regions of the three samples from Pt 4. The main haplotype of the first sample (First.0.0.0001) was genotyped as A, whereas the main haplotype of the second and third sample (Second.0.0.0001 and Third.0.0.0001) was genotype as D. At the X/preCore region, the main genotype was A, but Gen/DE and F haplotypes were also observed. Genotype reference sequences for the two regions: A2_X02763; B1_AB362933; C1_AB112066; D1_X59795 (for P/S region); DE_ D1_X59795 (for X/preCore region); E1_X75664 (for P/S region); DE_E1_X75664 (for X/preCore region); F1_HM590471; G0_HE981172; H0_AB275308; I0_FJ023664; J0_AB486012.

Comparison of the HBV genotyping results showed several discrepancies between the P/S and X/preCore regions (Table 3). First, a significantly larger number of samples with genotype mixtures were detected in X/preCore (96.7%) than in P/S (30%) (p<0.001). Second, distribution of the genotypes differed: X/preCore genotyped all samples as A, whereas P/S genotyped 24 samples as A (80%), five as D (16.7%) and one as F (3.3%) (p = 0.036). Third, genotypes D and F were more commonly detected in the X/preCore (27 samples, 90%) than the P/S region (four samples, 13.3%, p<0.001). Finally, in six samples from four patients (Pts 2, 4, 9, and 10) the main genotype in P/S differed from the one obtained in X/preCore (Table 2). In the baseline sample from Pt 2, genotyping in the P/S region yielded Gen/F, whereas in the X/preCore region it was Gen/A. In the second and third samples from Pt 4, the P/S region mainly yielded Gen/D whereas in the X/preCore region, Gen/A predominated (Fig 2). The second sample from Pt 9 showed Gen/D in P/S and Gen/A in X/preCore. In Pt 10, X/preCore yielded a predominant Gen/A, whereas P/S showed Gen/D in the first and second samples, and Gen/A alone had been selected at the third (under treatment) sample.

**Table 3:** Comparison of the number of samples with mixed genotypes, the main genotypes and the genotype prevalence between the polymerase/surface (P/S) and X/preCore region.

		P/S region		X/preCore region		p-value
		Number samples (%)		Number samples (%)		
Presence of mixed genotypes		9	(30.0%)	29	(96.7%)	<0.001
Main genotype	A	24	(80.0%)	30	(100.0%)	
	D	5	(16.7%)	0		0.036
	F	1	(3.3%)	0		
Genotype prevalence	A	17	(56.7%)	1	(3.3%)	
	D	4	(13.3%)	0		<0.001
	AD	5	(16.7%)	2	(6.7%)	
	ADF	4	(13.3%)	27	(90.0%)	

### HBV genotype changes over the study period

#### Genotype dynamics

Genotype dynamics were defined as changes in the main genotype (selection of genotypes at percentages of 50%-100%) in each patient (Table 4). During the natural quasispecies dynamics of the infection (percentage difference between the second and first sample), the P/S region showed HBV genotype changes in three patients (Pts 2, 4, and 9) (Table 4). Pt 2 changed from a D/F mixture to Gen/A. Pts 4 and 9 changed from Gen/A to Gen/D. In contrast, no changes in the main genotype were observed with genotyping of the X/preCore region.

**Table 4:** Percentage of enriched genotypes (%, type of genotype) in the polymerase/surface (P/S) and X/preCore regions during natural quasispecies dynamics (QD) and under treatment.

Patient	Period*	P/S region	X/preCore region^#^
1	Natural QD	7%, A	6.7%, A
	Treatment		13.3%, D; 1.1%, F
2	Natural QD	93%, A	0.9%, A
	Treatment	2.6%, D	6.6%, A
3	Natural QD	1.5%, A	26.6%, A
	Treatment		10.7%, D
4	Natural QD	100%, D	1.7%, F
	Treatment	9.4%, A; 0.7%, F	10.7%, A
5	Natural QD	-	12%, D; 0.3%, F
	Treatment	-	2.5%, D; 2.3%, F
6	Natural QD	-	1%, D; 0.9%, F
	Treatment	-	7.8%, D; 6.3%, F
7	Natural QD	-	2.7%, A
	Treatment	-	7.7%, D; 16.6%, F
8	Natural QD	16%, A; 4%, D	0.6%, D
	Treatment	7%, A	3.9%, A
9	Natural QD	87.2%, D	1.9%, A; 2.3%, D
	Treatment	100%, A	9.1%, D; 3.1%, F
10	Natural QD		11.2%, A; 2.8%, F
	Treatment	100%, A	2.9%, D; 2.4%, F

*Natural quasispecies dynamics is defined by the percentage difference between the second sample (before starting treatment) and first sample (at diagnosis) analyzed. The treatment period is established by the percentage difference between the third sample (under treatment) and second sample (before starting treatment) analyzed.

^**#**^X/preCore region showed an intermixing between Gen/D and E (S3 File)

During the treatment period (percentage difference between the third and second sample), genotype changed from D to A in two patients (Pts 9 and 10). This change was detected by LiPA and UDPS genotyping of the P/S region, but not the X/preCore region.

#### Changes in genotype proportions

Changes in genotype percentage that did not imply selection of a new genotype (changes from 0%-49%) were detected in both P/S and X/preCore (Table 4). Fluctuations in genotype percentage in the P/S region were observed in three (30%) patients during natural quasispecies dynamics (Pts 1, 3, and 8) and in three (30%) during treatment (Pts 2, 4, and 8). In contrast, in the X/preCore region, changes in genotype percentages were observed in all patients during natural quasispecies dynamics and under treatment (Table 4).

In the analysis of the P/S region during natural quasispecies dynamics, the Gen/A population was found to be enriched in two patients (Pt 1, 7%, and Pt 3, 1.5%). In Pt 2, Gen/A sequences also increased, with 93% (Table 4), resulting from a decrease in genotypes F and D from the first sample (Table 2). In Pt 8, genotypes A and D were enriched, with 16% and 4%, respectively. The natural quasispecies dynamics of Pt 9 showed an 87.2% increase in Gen/D. During treatment, there was a 2.6% increase in Gen/D in Pt 2, a 9.4% increase in Gen/A and 0.7% increase in Gen/F in Pt 4, and a 7% increase in Gen/A in Pt 8. Two patients (Pt 9 and 10) changed from Gen/D in the second sample to Gen/A in the third (Table 4).

The natural quasispecies dynamics of the X/preCore region showed genotype percentage changes in all samples, but no changes in the main genotype. The percentage of Gen/A increased in four patients (Pt 1 6.7%, Pt 2 0.9%, Pt 3 26.69%, and Pt 7 2.7%). Pt 4 showed an increase in Gen/F (1.7%) and Pts 5 and 6 showed enrichment of both Gen/D and F (Pt 5, 12% D and 0.3% F and Pt 6, 1% D and 0.9% F). Gen/DE increased in Pt 8 (0.6%), Gen/A (1.9%) and D (2.3%) in Pt 9, and Gen/A (11.2%) and F (2.8%) in Pt 10. During treatment, Gen/D and F increased in six patients (Pts 1, 5, 6, 7, 9, and 10), and Gen/D (10.7%) in one (Pt 3) (Table 3). In three patients (Pts 2, 4, and 8) there was Gen/A enrichment (6.6%, 10.7%, and 3.9%, respectively) (Table 3).

### Quasispecies complexity

**Fig 3:**
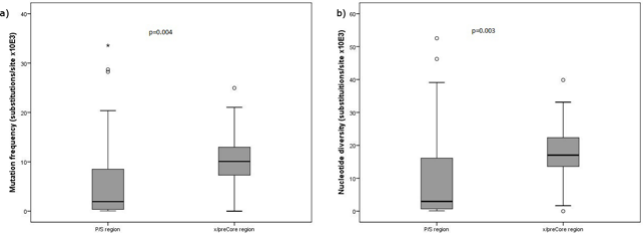
Distribution of quasispecies complexity in all the samples analyzed according to mutation frequency (a) and nucleotide diversity (b) in the polymerase/surface (P/S) and X/preCore regions.

Viral quasispecies complexity in the P/S and X/preCore regions was evaluated in all 30 samples using the mutation frequency (Mf) and nucleotide diversity (Pi) measures (S2 Table). The two indexes of quasispecies complexity were significantly higher in the X/preCore region than the P/S region (Mf, p = 0.004 and Pi, p = 0.003) (Fig 3), in accordance with the higher overlapping of P/S than X/preCore.

## Discussion

Hepatitis B virus genotype has been implicated in HBeAg seroconversion, in the clinical outcome of the infection, and in the response to treatment [8]. The accuracy of genotype classification is highly dependent on selection of appropriate reference sequences. Our references (S1, S2 and S3 Files) were validated as described in Results, and their information content was evaluated by sliding windows (SW) of 400 nucleotides in steps of 40 (S6 and S7 Files). Although whole genome analysis by SW shows that the PreS region has slightly higher discriminating power for genotyping HBV, we selected two amplicons in the P/S and X/preCore regions for this study. The X/preCore region was chosen because it includes the ENHII region and the main motifs involved in HBV replication. The P/S amplicon was selected to include the fragment generally used for HBV genotyping by LiPA, which was used in a previous step to genotype all samples in the study. The P/S fragment showed adequate genotype discrimination power, correctly classifying the eight main HBV genotypes (A-H).

Genotypes A, D, and F are the most prevalent in our population, and mixed infections with these three genotypes were found in both regions of the HBV genome studied. A significant prevalence of mixed HBV genotype infections has been reported in Asia, and it is suggested that these mixtures may correlate with clinical outcome and viral load [37]. Mixed genotypes (A/D, A/D/F, and D/F) have been described in Central and Eastern Europe [15], and mixed GenB/C has been reported in Taiwan [12]. In a previous study, we found a considerable rate (22%) [11] of mixed HBV genotypes (A/D and A/D/F) by LiPa and clonal methodologies in patients treated with nucleoside(t)e analogue treatment [7,16]. In the present study, genotype mixtures were observed in two (Pt 2 and 8) and nine samples (Pt 1, 2, 3, 4, 8, and 9) by LiPA and UDPS, respectively. Two major reasons may be the cause of these discrepancies. First, the fragment amplified for NGS is different from the fragment analyzed by LiPA; and second, the two techniques have methodological differences: NGS is a sequencing method and the definition of the genotype is obtained by phylogenesis, whereas LiPA is a hybridization method and therefore, an indirect measurement. Inconsistencies between LiPA and UDPS have been described in HCV genotyping [38] and HBV variant resistance detection [39].

The results of the present study were obtained by massive sequencing, a method widely used to study HBV and other viral infections [18–24,26,27]. Based on the experience acquired in previous efforts that confirmed the reproducibility of UDPS and its good correlation with molecular cloning for evaluating HBV and HCV quasispecies [26,27], we used UDPS to quantitatively analyze the presence and distribution of viral genotype mixtures. UDPS data treatment and filtering enabled detection of mixed infections, which were more frequent in X/preCore than in P/S (p<0.001). The high presence of mixed infection in X/preCore is a surprising result that warrants further study. Although this finding remains unexplained, it may be a result of high variability in this region, which encompasses positions (DR1, gap of the negative strand in the conformation of dsHBV-DNA) that may be points for recombination, which could be interpreted as mixed genotype infection. Furthermore, the presence of minor variants and genotype mixtures may have been underestimated previously because of the limited sensitivity of the methods used.

Selection of one genotype over another may occur due to immunological conditions or the effect of antiviral therapy. Selection or maintenance of Gen/A in the P/S region under treatment in eight of our patients (Pts 1, 3, 5, 6, 7, 8, 9, and 10) suggests that this region is under evolutive pressure during treatment and agrees with our previous observations [16]. HBV genotype fluctuations may be a common phenomenon in P/S, whereas genotype changes were not seen in the X/preCore region. In addition to the paradigm of mixed genotype infections, comparison of the genotypes found in the two regions studied showed discrepancies, suggesting intergenotypic recombination. This phenomena has been suggested previously, based on inconsistent genotyping between different regions of the HBV genome [38].

It cannot be excluded that the discrepancies between regions may have been due to UDPS variability or low coverage. However, in the remaining samples, there were notable differences in the percentages of HBV genotypes observed in the two regions, suggesting a high, complex recombination pattern, likely involving double and triple recombinants [38]. Intergenotypes tend to include the most frequent genotypes coexisting in a human population [13], and the putative intergenotypes observed in our study comprise the most common genotypes in the Spanish population [4]. This fact, added to co-infection with various genotypes and the long life of infected hepatocytes (2.4 to >70 days under LMV), may facilitate multiple infections in a single liver cell by different genotypes [40]. Nevertheless, recombination should be confirmed by UDPS using long sequencing fragments that cover both regions in the same sequence.

The study of quasispecies complexity is limited by the individual patterns that seem to evolve in each patient due to the host immune system. This observation is consistent with the dynamics of consecutive expansion and contraction of quasispecies complexity in response to changes in the host environment. An emerging mutant with greatly improved fitness for the situation would lead to homogenization of the quasispecies, in detriment to the less fit. On the other hand, long periods without environmental changes would produce synonymous mutants of the dominant haplotype, thus contributing to complexity expansion and providing possible escapes for future changes. In this respect, the observed diversity values would correspond to random samplings on sawtooth signals. Despite this limitations, the higher complexity of X/preCore than P/S is in accordance with the higher overlapping of P/S than X/preCore.

In conclusion, this UDPS study provides evidence of HBV genotype mixtures that change over time and illustrates the complex dynamics of the HBV quasispecies as an additional mechanism when adapting to new situations, such as host immune response and/or antiviral treatment. Discrepancies between genotypes in the P/S and X/preCore regions suggest phenomena of intergenotype recombination and indicate the need for an international expert consensus effort to set an HBV genotyping approach that will lead to a more comprehensive understanding of the clinical significance of HBV genotype classification.

## Supporting Information

S1 File Genotyping capacity of the reference sequences using UPGMA trees and plotting (complete HBV genome). (PDF)

S2 File Genotyping capacity of the reference sequences trimmed to positions 615 to 969 (covering the P/S amplicon). (PDF)

S3 File Genotyping capacity of the reference sequences trimmed to positions 1596 to1 912 (covering the P/S amplicon). (PDF)

S4 File UPGMA trees and MDS plots of the first three most common haplotypes in the P/S region per sample with the reference sequences. (PDF)

S5 File UPGMA trees and MDS plots of the first three most common haplotypes in the X/preCore region per sample with the reference sequences. (PDF)

S6 File UPGMA tree of the sliding windows analysis of 400 nucleotides in steps of 40 for the eight HBV genotypes. (PDF)

S7 File Sliding windows analysis of 400 nucleotides in steps of 40 for the eight different genotypes. (PDF)

S1 Protocol Supplementary Materials and methods, Genotyping and Examination of HBV genotypes reference sequence. Detailed description of amplification of the P/S and X/preCore regions, UDPS data treatment, and genotyping, description of genotyping and examination of HBV genomtypes reference sequence.
(DOCX)

S1 Table Biosample accession numbers for each sample and region analyzed. (DOCX)

S2 Table Complexity parameters obtained for the P/S and X/preCore regions. These parameters were obtained for the three samples per patient analyzed: the first available at diagnosis (1st), the second before starting treatment (2nd), and the third while under treatment (3rd).
(DOCX)
