# Supplementary material for: Complex Genotype Mixtures Analyzed by Deep Sequencing in Two Different Regions of Hepatitis B Virus
Source: PLoS One. 2015 Dec 29;10(12):e0144816. doi: 10.1371/journal.pone.0144816 (PMC4695080; doi:10.1371/journal.pone.0144816)
Supplement: S1 File — (PDF) [file pone.0144816.s001.pdf]

HBV reference sequences by genotype, with accessions, used in full genome exploration

A	B	C	D	E	F	G	H	I	J
A1_AB241115	B1_AB073858	C0_D23683	D0_X65259	E1_X75664	F1_AY090459	G0_AF160501	H0_AB179747	I0_FJ023660	J0_AB486012
A1_AY233278	B1_AB362933	C0_L08805	D0_X68292	E2_X75657	F1_DQ823095	G0_EF464098	H0_AB275308	I0_FJ023664	
A2_AJ309371	B1_D00329	C0_M38636	D1_X59795	E3_AM494694	F1_HE981184	G0_HE981172	H0_AB516395		
A2_AM282986	B2_AP011084	C0_X14193	D1_X80926	E3_FJ349237	F1_HM590471	G0_HE981176	H0_AP007261		
A2_AY738141	B2_AY596111	C1_AB031265	D2_X97848	E4_FJ349226	F1_HQ378247				
A2_X02763	B2_GQ924653	C1_AB112066	D2_Z35716	E4_HM363569	F2_AY090455				
A2_X51970	B2_GU815751	C10_AB540583	D3_AY233291	E5_DQ060828	F2_AY311369				
A2_Z72479	B3_AP011085	C2_AB033553	D3_V01460	E5_JQ000008	F2_X69798				
A3_AB194951	B3_M54923	C2_AF533983	D3_X65258		F3_AB036910				
A3_AB194952	B4_AB073835	C2_AY123041	D4_AB033559		F3_AB036911				
A4_AM180623	B4_AB115551	C2_D16665	D4_AB048702		F3_AB036915				
A4_AY934764	B5_AB219427	C2_D23681	D5_AB033558		F3_FJ589066				
A5_FJ692609	B5_AP011086	C2_X52939	D5_DQ315779		F3_X75663				
A5_FJ692613	B6_AB287316	C3_X75656	D6_AB493846		F4_AB166850				
A6_GQ331047	B6_DQ463787	C3_X75665	D6_AB554023		F4_DQ823090				
A6_GQ331048	B7_AP011091	C4_AB048704	D7_AM494716		F4_EU366116				
	B7_EF473977	C4_AB048705	D7_FJ904430		F4_HE974368				
	B8_AP011093	C5_AB241109							
	B8_AP011094	C5_AP011099							
		C6_AP011102							
		C6_AP011103							
		C7_EU670263							
		C8_AP011104							
		C8_AP011107							
		C9_AP011108							
16	19	25	17	8	17	4	4	2	1

Outgroup
X.chimpanzee_AF222323
X.gibbon_AB037927
X.orangutan_AF193863
X.wmonkey_AF046996

HBV Reference Sequences Discriminating Power

HBV - Whole Genome

Genotype	RefSeq No.	Geometric Variability	Mean Squared Distances Within Genotype Min	Median	Max	Max Corrected	Nearest Dif. Gen.	Nearest Genotype
A	16	0,000919	0,001320	0,001800	0,004760	0,003841	0,00660	I
B	19	0,001070	0,001540	0,001990	0,003460	0,002390	0,00637	C
C	25	0,001260	0,001470	0,002430	0,004450	0,003190	0,00505	I
D	17	0,000963	0,001250	0,002120	0,002960	0,001997	0,00637	E
E	8	0,000099	0,000188	0,000219	0,000293	0,000194	0,00586	D
F	17	0,000994	0,001630	0,002230	0,002440	0,001446	0,00675	H
G	4	0,000037	0,000055	0,000088	0,000161	0,000124	0,01540	A
H	4	0,000168	0,000256	0,000348	0,000836	0,000668	0,00852	F
I	2	0,000218	0,000872	0,000872	0,000872	0,000654	0,00758	C

Mean squared distances within genotype $$\frac{1}{n_I - 1} \sum_{i \in I} d_{i,j}^2; \quad j: 1..n_I$$

Genotype geometric variability $$\hat{V}_I = \frac{1}{2 n_I^2} \sum_{i,j \in I} d_{i,j}^2$$

DB rule $$\min_I \left(\hat{\phi}_I^2(k) = \frac{1}{n_I} \sum_{i \in I} d_{i,k}^2 - \hat{V}_I \right)$$

HBV Reference Sequences Classification by DB rule
Whole genome sequences

Subtype and Accession No.	$\phi_A^2(k)$	$\phi_B^2(k)$	$\phi_C^2(k)$	$\phi_D^2(k)$	$\phi_E^2(k)$	$\phi_F^2(k)$	$\phi_G^2(k)$	$\phi_H^2(k)$	DB Rule Type
A1_AB241115	**0,001100**	0,008020	0,006870	0,008210	0,007650	0,018200	0,011800	0,020300	A
A1_AY233278	**0,001180**	0,008760	0,007100	0,008990	0,008430	0,019500	0,013300	0,021700	A
A2_AJ309371	**0,000632**	0,007940	0,007040	0,008580	0,009390	0,019900	0,012600	0,021600	A
A2_AM282986	**0,000360**	0,007540	0,006240	0,007860	0,008780	0,018400	0,011700	0,020400	A
A2_AY738141	**0,000727**	0,008030	0,007250	0,008710	0,009600	0,019900	0,012900	0,021600	A
A2_X02763	**0,000599**	0,008020	0,007010	0,008200	0,009270	0,019700	0,012400	0,021600	A
A2_X51970	**0,000702**	0,007940	0,006700	0,008560	0,009000	0,019100	0,012700	0,021200	A
A2_Z72479	**0,004030**	0,015800	0,014200	0,015700	0,016800	0,030400	0,021000	0,031700	A
A3_AB194951	**0,001110**	0,008290	0,006970	0,009440	0,009260	0,020500	0,013200	0,022300	A
A3_AB194952	**0,001010**	0,008390	0,006510	0,009380	0,008930	0,019900	0,013000	0,021600	A
A4_AM180623	**0,000946**	0,008060	0,005830	0,008430	0,008010	0,018700	0,011600	0,022000	A
A4_AY934764	**0,000893**	0,007740	0,006160	0,008580	0,008290	0,019200	0,011800	0,022200	A
A5_FJ692609	**0,000756**	0,008180	0,006750	0,008600	0,008720	0,020000	0,012900	0,022500	A
A5_FJ692613	**0,000802**	0,008390	0,006910	0,008600	0,008640	0,019400	0,013100	0,021300	A
A6_GQ331047	**0,000841**	0,007580	0,005770	0,008310	0,008090	0,019300	0,011500	0,021300	A
A6_GQ331048	**0,001030**	0,007970	0,006140	0,008710	0,008540	0,019600	0,011900	0,022300	A
B1_AB073858	0,008050	**0,002150**	0,009020	0,010800	0,011800	0,020300	0,016200	0,022800	B
B1_AB362933	0,007920	**0,001620**	0,008120	0,010100	0,011100	0,018900	0,015900	0,021400	B
B1_D00329	0,006710	**0,001490**	0,007580	0,008870	0,009970	0,019100	0,015300	0,021300	B
B2_AP011084	0,008360	**0,000697**	0,006440	0,010500	0,011100	0,019500	0,015800	0,021600	B
B2_AY596111	0,009940	**0,001330**	0,008080	0,012000	0,012700	0,021700	0,018300	0,022800	B
B2_GQ924653	0,008030	**0,000624**	0,006400	0,010000	0,010700	0,019100	0,015100	0,021200	B
B2_GU815751	0,007790	**0,000515**	0,006150	0,009700	0,010600	0,018900	0,015200	0,020800	B
B3_AP011085	0,008040	**0,000439**	0,005610	0,009110	0,010400	0,019100	0,014600	0,021200	B
B3_M54923	0,009020	**0,000721**	0,006190	0,009900	0,011400	0,019900	0,015600	0,021400	B
B4_AB073835	0,007980	**0,000882**	0,006000	0,009580	0,010700	0,018400	0,014000	0,020900	B
B4_AB115551	0,009040	**0,001320**	0,006820	0,011100	0,011700	0,019900	0,015100	0,022900	B
B5_AB219427	0,009870	**0,001230**	0,007060	0,011400	0,012200	0,021300	0,015800	0,024000	B
B5_AP011086	0,009380	**0,000866**	0,006440	0,010600	0,011600	0,019200	0,015600	0,021700	B
B6_AB287316	0,009000	**0,002470**	0,009000	0,010300	0,011600	0,020000	0,016100	0,020900	B
B6_DQ463787	0,008980	**0,002450**	0,008460	0,010200	0,011200	0,020700	0,015100	0,021000	B
B7_AP011091	0,008840	**0,000759**	0,005930	0,009000	0,010500	0,018500	0,015300	0,020900	B
B7_EF473977	0,009450	**0,000911**	0,006460	0,010200	0,011000	0,019300	0,015200	0,021400	B
B8_AP011093	0,008640	**0,000900**	0,006270	0,009730	0,010800	0,019500	0,016500	0,021400	B
B8_AP011094	0,010100	**0,001200**	0,007080	0,010600	0,011400	0,020800	0,016800	0,021900	B
C0_D23683	0,007850	0,007300	**0,001070**	0,010300	0,011000	0,018700	0,015900	0,020200	C
C0_L08805	0,007260	0,006650	**0,000861**	0,010100	0,010200	0,017700	0,014500	0,019200	C
C0_M38636	0,007950	0,007580	**0,000860**	0,010700	0,011200	0,019100	0,016600	0,021900	C
C0_X14193	0,007880	0,007510	**0,000828**	0,010700	0,011200	0,019000	0,016500	0,021800	C
C1_AB031265	0,006910	0,004970	**0,001920**	0,009170	0,010600	0,017900	0,014300	0,019700	C
C1_AB112066	0,008000	0,007320	**0,001810**	0,010200	0,011500	0,018300	0,016700	0,020200	C
C10_AB540583	0,009030	0,008700	**0,002460**	0,011300	0,011000	0,020600	0,015500	0,021700	C
C2_AB033553	0,006350	0,006020	**0,000160**	0,008830	0,008910	0,016700	0,013800	0,018800	C
C2_AF533983	0,007320	0,006610	**0,000600**	0,009740	0,010100	0,018200	0,015500	0,020600	C
C2_AY123041	0,007140	0,006760	**0,000512**	0,009770	0,009660	0,018400	0,015300	0,020500	C
C2_D16665	0,005990	0,007530	**0,001350**	0,009060	0,008780	0,018200	0,014900	0,019500	C
C2_D23681	0,007950	0,007270	**0,000692**	0,010300	0,011000	0,018700	0,015700	0,020700	C
C2_X52939	0,007710	0,007600	**0,000870**	0,009560	0,010200	0,018300	0,015500	0,019800	C
C3_X75656	0,006840	0,006400	**0,001110**	0,008900	0,008490	0,016500	0,014500	0,019500	C
C3_X75665	0,006500	0,006930	**0,001370**	0,008640	0,008770	0,017000	0,013900	0,019000	C
C4_AB048704	0,008800	0,009480	**0,003270**	0,010800	0,011300	0,018800	0,018600	0,022000	C

HBV Reference Sequences Classification by DB rule
Whole genome sequences

Subtype and Accession No.	$\phi_A^2(k)$	$\phi_B^2(k)$	$\phi_C^2(k)$	$\phi_D^2(k)$	$\phi_E^2(k)$	$\phi_F^2(k)$	$\phi_G^2(k)$	$\phi_H^2(k)$	DB Rule Type
C4_AB048705	0,008310	0,008740	**0,002800**	0,010200	0,010600	0,017900	0,017900	0,020700	C
C5_AB241109	0,007600	0,007240	**0,002200**	0,009970	0,009740	0,017700	0,015000	0,022800	C
C5_AP011099	0,007160	0,007840	**0,002010**	0,009340	0,009810	0,017900	0,015300	0,023400	C
C6_AP011102	0,007460	0,007220	**0,001560**	0,010100	0,010300	0,017700	0,016300	0,019600	C
C6_AP011103	0,007320	0,007540	**0,001520**	0,010500	0,009500	0,017000	0,014700	0,019400	C
C7_EU670263	0,007220	0,006880	**0,001100**	0,009280	0,009700	0,017400	0,016000	0,019100	C
C8_AP011104	0,007230	0,006860	**0,001160**	0,009710	0,010100	0,016900	0,016000	0,018600	C
C8_AP011107	0,007470	0,007080	**0,001170**	0,009760	0,010100	0,017400	0,016500	0,019100	C
C9_AP011108	0,006570	0,006000	**0,000934**	0,008800	0,009320	0,017700	0,015100	0,018900	C
D0_X65259	0,006540	0,009560	0,009410	**0,001610**	0,007000	0,019300	0,013100	0,022100	D
D0_X68292	0,005670	0,009640	0,009670	**0,002050**	0,007770	0,019600	0,013300	0,020800	D
D1_X59795	0,010600	0,011200	0,011000	**0,001330**	0,007360	0,022400	0,014400	0,024800	D
D1_X80926	0,009970	0,010100	0,010100	**0,001160**	0,006410	0,020600	0,015300	0,023000	D
D2_X97848	0,009220	0,009100	0,009260	**0,000552**	0,005030	0,019000	0,012400	0,020900	D
D2_Z35716	0,009480	0,009940	0,009300	**0,000623**	0,005490	0,020300	0,013900	0,022500	D
D3_AY233291	0,008730	0,009610	0,008980	**0,000437**	0,005320	0,019800	0,012600	0,020000	D
D3_V01460	0,008920	0,009470	0,008760	**0,000246**	0,005180	0,019500	0,012800	0,020600	D
D3_X65258	0,009040	0,011000	0,010700	**0,001210**	0,007700	0,022000	0,014700	0,023700	D
D4_AB033559	0,008960	0,009730	0,008270	**0,000844**	0,005350	0,018600	0,013300	0,020200	D
D4_AB048702	0,008830	0,009730	0,008820	**0,000972**	0,005690	0,019300	0,013600	0,021000	D
D5_AB033558	0,010000	0,011300	0,009620	**0,001770**	0,007090	0,020000	0,014600	0,021600	D
D5_DQ315779	0,010500	0,011300	0,010400	**0,001640**	0,006780	0,019400	0,014000	0,021200	D
D6_AB493846	0,009170	0,009410	0,009090	**0,000588**	0,005750	0,019000	0,013300	0,020500	D
D6_AB554023	0,009330	0,009340	0,009240	**0,000534**	0,005660	0,019400	0,013300	0,020900	D
D7_AM494716	0,009420	0,010000	0,009150	**0,001200**	0,005400	0,019800	0,013500	0,022200	D
D7_FJ904430	0,010300	0,011000	0,010200	**0,001690**	0,005920	0,021100	0,014300	0,022400	D
E1_X75664	0,008790	0,010500	0,008850	0,005440	**0,000206**	0,018100	0,012200	0,020400	E
E2_X75657	0,007890	0,009840	0,008660	0,005120	**0,000094**	0,017300	0,011900	0,020100	E
E3_AM494694	0,008770	0,010700	0,009280	0,005390	**0,000133**	0,018200	0,012200	0,020800	E
E3_FJ349237	0,008200	0,009900	0,008740	0,005130	**0,000092**	0,017700	0,011900	0,020100	E
E4_FJ349226	0,008380	0,010100	0,009380	0,005330	**0,000162**	0,018000	0,011800	0,020600	E
E4_HM363569	0,008230	0,010200	0,008920	0,005550	**0,000110**	0,018200	0,012400	0,021000	E
E5_DQ060828	0,008620	0,010500	0,009240	0,005450	**0,000146**	0,018200	0,011900	0,021600	E
E5_JQ000008	0,008260	0,009990	0,008620	0,005040	**0,000086**	0,018000	0,012100	0,020800	E
F1_AY090459	0,019000	0,020400	0,017800	0,019900	0,017800	**0,001470**	0,021600	0,008060	F
F1_DQ823095	0,018400	0,019200	0,017300	0,019900	0,017100	**0,001320**	0,021100	0,007060	F
F1_HE981184	0,018500	0,019700	0,017600	0,020000	0,017300	**0,001440**	0,021400	0,007310	F
F1_HM590471	0,018600	0,019400	0,017400	0,019600	0,017000	**0,001200**	0,021500	0,006880	F
F1_HQ378247	0,018200	0,019100	0,017200	0,020000	0,016900	**0,001330**	0,020900	0,007040	F
F2_AY090455	0,020900	0,019600	0,018100	0,020000	0,019100	**0,001250**	0,021000	0,007330	F
F2_AY311369	0,021800	0,020300	0,018400	0,020500	0,019700	**0,001240**	0,021200	0,007490	F
F2_X69798	0,020700	0,019100	0,016800	0,019400	0,018300	**0,000985**	0,021100	0,006970	F
F3_AB036910	0,020800	0,019000	0,017300	0,019500	0,019400	**0,000606**	0,021600	0,006230	F
F3_AB036911	0,021400	0,019400	0,017700	0,020000	0,019700	**0,000717**	0,021400	0,006220	F
F3_AB036915	0,021200	0,019400	0,017800	0,020000	0,020000	**0,000800**	0,021300	0,006490	F
F3_FJ589066	0,021900	0,019800	0,018700	0,020500	0,020500	**0,001030**	0,022400	0,006760	F
F3_X75663	0,018400	0,019100	0,017100	0,019200	0,018000	**0,001430**	0,020900	0,007760	F
F4_AB166850	0,020900	0,019800	0,018200	0,020300	0,020200	**0,001360**	0,021600	0,007690	F
F4_DQ823090	0,020300	0,019300	0,017300	0,019900	0,019300	**0,000664**	0,021200	0,006590	F
F4_EU366116	0,020800	0,019700	0,017700	0,020100	0,019900	**0,000826**	0,021500	0,006810	F
F4_HE974368	0,021200	0,021100	0,018800	0,020400	0,020400	**0,001400**	0,023000	0,008020	F

HBV Reference Sequences Classification by DB rule
Whole genome sequences

Subtype and Accession No.	$\phi_A^2(k)$	$\phi_B^2(k)$	$\phi_C^2(k)$	$\phi_D^2(k)$	$\phi_E^2(k)$	$\phi_F^2(k)$	$\phi_G^2(k)$	$\phi_H^2(k)$	DB Rule Type
G0_AF160501	0,011500	0,014100	0,013900	0,012200	0,011400	0,020300	**0,000009**	0,023300	G
G0_EF464098	0,012600	0,014900	0,014800	0,013300	0,012700	0,019500	**0,000149**	0,022300	G
G0_HE981172	0,012700	0,015500	0,015200	0,013400	0,012500	0,022000	**0,000084**	0,024700	G
G0_HE981176	0,011600	0,014100	0,013800	0,012100	0,011300	0,020200	**0,000019**	0,023100	G
H0_AB179747	0,020000	0,019300	0,018000	0,019300	0,019200	0,005360	0,022200	**0,000044**	H
H0_AB275308	0,020400	0,019700	0,018300	0,019600	0,019400	0,005540	0,022600	**0,000091**	H
H0_AB516395	0,024500	0,023900	0,021800	0,024100	0,023800	0,008190	0,026000	**0,000817**	H
H0_AP007261	0,020900	0,020100	0,018600	0,020500	0,020600	0,006010	0,023200	**0,000240**	H
I0_FJ023660	0,005270	0,006840	0,003590	0,008840	0,009220	0,017600	0,011900	0,019300	I
I0_FJ023664	0,005640	0,006990	0,003690	0,008810	0,009190	0,018400	0,011600	0,021200	I
J0_AB486012	0,014400	0,012000	0,011300	0,015100	0,013700	0,020700	0,017000	0,022500	C
X.chimpanzee AF222323	0,009060	0,009410	0,008670	0,009010	0,008040	0,016600	0,012000	0,019200	E
X.gibbon AB037927	0,011700	0,011700	0,010700	0,013300	0,012500	0,019800	0,016500	0,023600	C
X.orangutan AF193863	0,012600	0,011700	0,010200	0,012900	0,012300	0,019000	0,017700	0,022000	C
X.wmonkey AF046996	0,067800	0,063100	0,063600	0,065100	0,064200	0,071900	0,068000	0,075700	X

$$\hat{\phi}_I^2(k) = \frac{1}{n_I}\sum_{i \in I} d_{i,k}^2 - \frac{1}{2n_I^2}\sum_{i,j \in I} d_{i,j}^2$$

UPGMA tree (raw): HDV whole genome

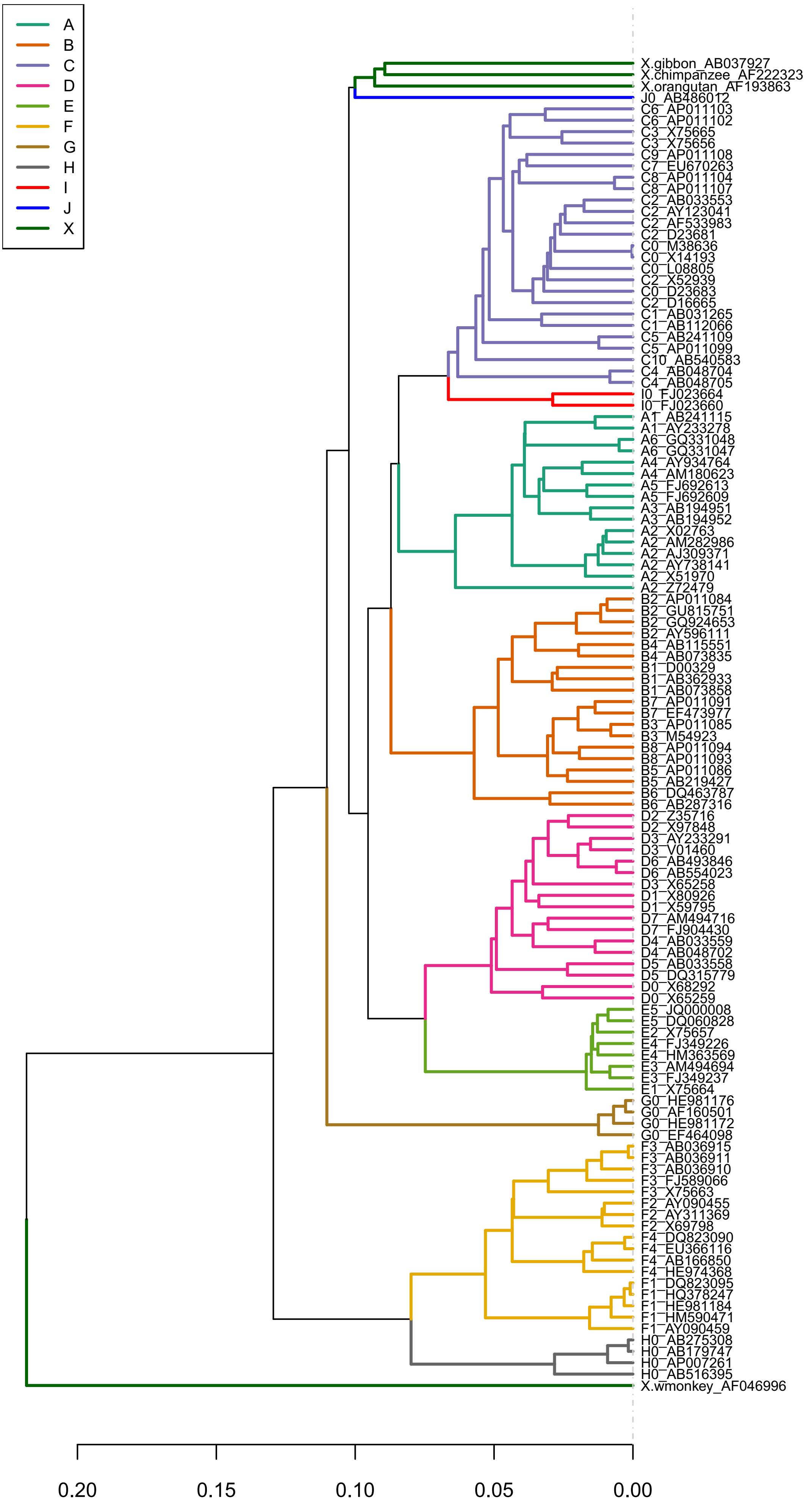

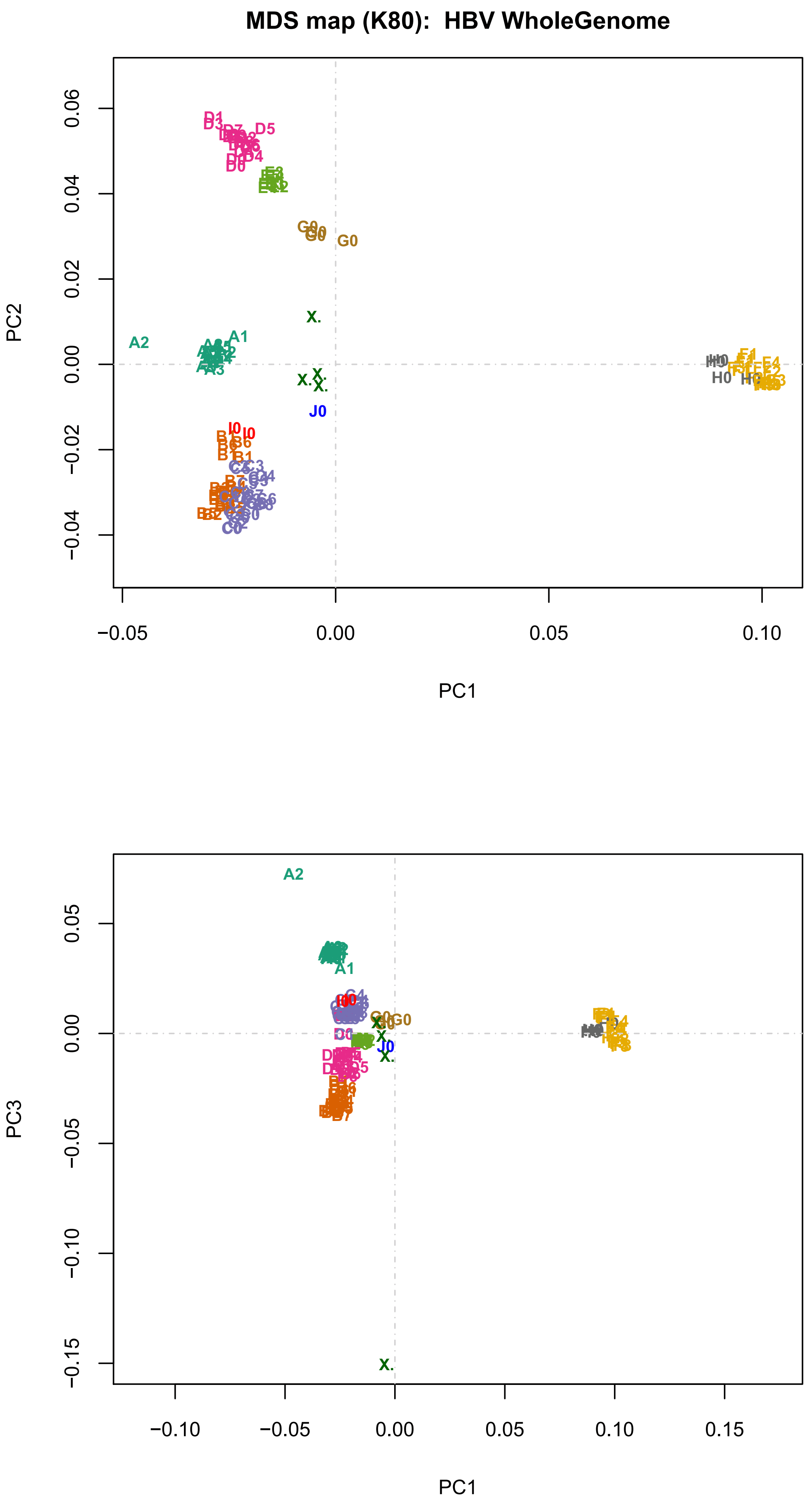
MDS map (K80): HBV WholeGenome
PC2
PC1
PC3
PC1
0.06
0.04
0.02
0.00
−0.02
−0.04
−0.05
0.00
0.05
0.10
0.05
0.00
−0.05
−0.10
−0.15
−0.10
−0.05
0.00
0.05
0.10
0.15
D1
D5
G0
A2
A1
X.
H0
J0
I0
B1
B6
C3
C4
C6
C0
E4
E3
F4
F3
