# Supplementary material for: Complex Genotype Mixtures Analyzed by Deep Sequencing in Two Different Regions of Hepatitis B Virus
Source: PLoS One. 2015 Dec 29;10(12):e0144816. doi: 10.1371/journal.pone.0144816 (PMC4695080; doi:10.1371/journal.pone.0144816)

HBV reference sequences by genotype, with accessions, used in genotyping region 616 to 969

A	B	C	D	E	F	G	H	I	J
A1_AB241115	B1_AB073858	C0_D23683	D1_X59795	E1_X75664	F1_AY090459	G0_EF464098	H0_AB275308	I0_FJ023660	J0_AB486012
A1_AY233278	B1_AB362933	C0_L08805	D1_X80926	E2_X75657	F1_HE981184	G0_HE981172	H0_AB516395	I0_FJ023664	
A2_AJ309371	B1_D00329	C0_M38636	D2_X97848	E3_AM494694	F1_HM590471	G0_HE981176	H0_AP007261		
A2_AM282986	B2_AP011084	C1_AB031265	D2_Z35716	E3_FJ349237	F2_AY090455				
A2_AY738141	B2_AY596111	C1_AB112066	D3_AY233291	E4_FJ349226	F2_AY311369				
A2_X02763	B2_GQ924653	C10_AB540583	D3_V01460	E4_HM363569	F2_X69798				
A2_X51970	B2_GU815751	C2_AB033553	D3_X65258	E5_DQ060828	F3_AB036910				
A2_Z72479	B3_AP011085	C2_AF533983	D4_AB033559	E5_JQ000008	F3_AB036915				
A3_AB194951	B3_M54923	C2_AY123041	D4_AB048702		F3_FJ589066				
A3_AB194952	B4_AB073835	C2_D16665	D5_AB033558		F3_X75663				
A4_AM180623	B4_AB115551	C2_D23681	D5_DQ315779		F4_AB166850				
A4_AY934764	B5_AB219427	C2_X52939	D6_AB493846		F4_DQ823090				
A5_FJ692609	B5_AP011086	C3_X75656	D6_AB554023		F4_EU366116				
A5_FJ692613	B6_AB287316	C3_X75665	D7_AM494716		F4_HE974368				
A6_GQ331047	B6_DQ463787	C4_AB048704	D7_FJ904430						
A6_GQ331048	B7_AP011091	C4_AB048705							
	B7_EF473977	C5_AB241109							
	B8_AP011093	C5_AP011099							
	B8_AP011094	C6_AP011102							
		C6_AP011103							
		C7_EU670263							
		C8_AP011104							
		C8_AP011107							
		C9_AP011108							
16	19	24	15	8	14	3	3	2	1

HBV Reference Sequences Discriminating Power

HBV - S/P Gene, amplicon 616 to 969

Genotype	RefSeq No.	Geometric Variability	Mean Squared Distances Within Genotype: Min	Median	Max	Max Corrected	Nearest Dif. Gen.	Nearest Genotype
A	16	0,001210	0,001310	0,002360	0,005580	0,004370	0,00458	B
B	19	0,001420	0,002090	0,002890	0,005060	0,003640	0,00496	A
C	24	0,001970	0,002490	0,003580	0,011400	0,009430	0,00635	A
D	15	0,001370	0,001760	0,002490	0,006180	0,004810	0,00617	I
E	8	0,000215	0,000336	0,000418	0,000729	0,000514	0,00633	D
F	14	0,000898	0,001390	0,001800	0,003080	0,002182	0,01210	H
G	3	0,000024	0,000042	0,000072	0,000106	0,000082	0,00476	I
H	3	0,000161	0,000293	0,000450	0,000709	0,000548	0,01430	F
I	2	0,000139	0,000554	0,000554	0,000554	0,000415	0,00522	A

Mean squared distances within genotype $$\frac{1}{n_I - 1} \sum_{i \in I} d_{i,j}^2; \quad j: 1..n_I$$

Genotype geometric variability $$\hat{V}_I = \frac{1}{2 n_I^2} \sum_{i,j \in I} d_{i,j}^2$$

DB rule $$\min_I \left(\hat{\phi}_I^2(k) = \frac{1}{n_I} \sum_{i \in I} d_{i,k}^2 - \hat{V}_I \right)$$

HBV Reference Sequences Classification by DB rule
S-P gene, amplicon 616 - 969

Subtype and Accession No.	$\phi_A^2(k)$	$\phi_B^2(k)$	$\phi_C^2(k)$	$\phi_D^2(k)$	$\phi_E^2(k)$	$\phi_F^2(k)$	$\phi_G^2(k)$	$\phi_H^2(k)$	DB Rule Type
A1_AB241115	**0,001730**	0,005150	0,006060	0,005970	0,006540	0,018300	0,005210	0,024900	A
A1_AY233278	**0,002190**	0,005760	0,006820	0,008070	0,008870	0,019300	0,007270	0,025300	A
A2_AJ309371	**0,000730**	0,007320	0,009130	0,007700	0,010800	0,021700	0,008030	0,025100	A
A2_AM282986	**0,000481**	0,006230	0,008960	0,007490	0,010100	0,019900	0,007440	0,022800	A
A2_AY738141	**0,001270**	0,007890	0,010600	0,009040	0,012200	0,023900	0,009300	0,025100	A
A2_X02763	**0,000554**	0,006790	0,008400	0,006880	0,010800	0,021300	0,007470	0,025100	A
A2_X51970	**0,000365**	0,004990	0,007710	0,006180	0,008170	0,017600	0,006330	0,022000	A
A2_Z72479	**0,004580**	0,016100	0,019600	0,016300	0,019900	0,035600	0,017600	0,040800	A
A3_AB194951	**0,001440**	0,006470	0,007090	0,009640	0,012100	0,026200	0,008000	0,025000	A
A3_AB194952	**0,001010**	0,004720	0,005160	0,007760	0,008440	0,019200	0,007410	0,022700	A
A4_AM180623	**0,001040**	0,005870	0,006720	0,007740	0,007050	0,018400	0,008400	0,023200	A
A4_AY934764	**0,001080**	0,005790	0,007220	0,007300	0,008210	0,018900	0,007770	0,021000	A
A5_FJ692609	**0,000024**	0,003280	0,004450	0,004880	0,006450	0,016900	0,004460	0,021400	A
A5_FJ692613	**0,001210**	0,004740	0,006730	0,007530	0,007660	0,020900	0,008000	0,023500	A
A6_GQ331047	**0,001770**	0,004960	0,004120	0,007640	0,008910	0,018800	0,005670	0,027000	A
A6_GQ331048	**0,002490**	0,006170	0,005170	0,009110	0,010800	0,020900	0,007470	0,029500	A
B1_AB073858	0,004400	**0,001670**	0,006360	0,008740	0,008030	0,021100	0,004010	0,021900	B
B1_AB362933	0,003310	**0,000735**	0,005290	0,008450	0,007530	0,020300	0,006310	0,020900	B
B1_D00329	0,003090	**0,001790**	0,006060	0,009250	0,009090	0,021400	0,007360	0,022900	B
B2_AP011084	0,004970	**0,001530**	0,006640	0,010500	0,009780	0,020600	0,007970	0,018900	B
B2_AY596111	0,006030	**0,001680**	0,008610	0,010500	0,011500	0,020800	0,008560	0,020500	B
B2_GQ924653	0,003780	**0,000749**	0,005620	0,008730	0,008180	0,019500	0,005330	0,020900	B
B2_GU815751	0,004480	**0,001210**	0,006100	0,009750	0,009120	0,020600	0,007390	0,018900	B
B3_AP011085	0,007620	**0,000629**	0,005370	0,011500	0,009950	0,020000	0,008000	0,023400	B
B3_M54923	0,008510	**0,001710**	0,005900	0,013200	0,011400	0,021700	0,010600	0,023000	B
B4_AB073835	0,004840	**0,001270**	0,005910	0,009010	0,008530	0,019300	0,005650	0,020900	B
B4_AB115551	0,004420	**0,000963**	0,005280	0,009110	0,008620	0,019800	0,005190	0,019900	B
B5_AB219427	0,007280	**0,002050**	0,007490	0,015700	0,012400	0,025400	0,007860	0,022700	B
B5_AP011086	0,007460	**0,000755**	0,005900	0,013300	0,010600	0,021800	0,007240	0,023500	B
B6_AB287316	0,011400	**0,003760**	0,007670	0,015400	0,011600	0,019400	0,010900	0,021900	B
B6_DQ463787	0,008850	**0,003170**	0,005170	0,012000	0,010600	0,020700	0,009190	0,020500	B
B7_AP011091	0,010800	**0,002590**	0,007010	0,014900	0,012400	0,023200	0,008590	0,025400	B
B7_EF473977	0,008490	**0,001200**	0,004620	0,013400	0,010900	0,020300	0,008970	0,023100	B
B8_AP011093	0,007700	**0,001100**	0,006610	0,014600	0,013600	0,023500	0,010600	0,022000	B
B8_AP011094	0,007990	**0,001470**	0,005980	0,015200	0,013600	0,025800	0,011000	0,023000	B
C0_D23683	0,011800	0,009130	**0,001800**	0,018200	0,018000	0,028800	0,011900	0,028400	C
C0_L08805	0,008610	0,005670	**0,000848**	0,016300	0,014000	0,022900	0,009220	0,022700	C
C0_M38636	0,011100	0,006870	**0,000881**	0,015200	0,016800	0,023500	0,010500	0,026000	C
C1_AB031265	0,006980	0,005850	**0,002980**	0,012000	0,013900	0,021100	0,009190	0,019300	C
C1_AB112066	0,008370	0,006450	**0,001950**	0,013700	0,017800	0,026600	0,012000	0,024900	C
C10_AB540583	0,009740	0,007740	**0,002100**	0,013600	0,017200	0,028900	0,007900	0,027600	C
C2_AB033553	0,008090	0,007120	**0,000695**	0,014000	0,013500	0,023600	0,010300	0,024900	C
C2_AF533983	0,009840	0,007160	**0,000456**	0,014600	0,016100	0,025200	0,009860	0,027200	C
C2_AY123041	0,009410	0,006930	**0,000486**	0,015700	0,013300	0,027300	0,012000	0,027200	C
C2_D16665	0,009410	0,006170	**0,000698**	0,015500	0,013300	0,026200	0,012000	0,026000	C
C2_D23681	0,011900	0,008260	**0,001220**	0,015300	0,016100	0,023600	0,012500	0,026000	C
C2_X52939	0,013400	0,012400	**0,002590**	0,018600	0,020600	0,029700	0,015300	0,027200	C
C3_X75656	0,006630	0,005150	**0,001680**	0,010700	0,011000	0,020700	0,010000	0,023200	C
C3_X75665	0,005040	0,004680	**0,001440**	0,008660	0,008940	0,020300	0,008100	0,023100	C
C4_AB048704	**0,007640**	0,008780	**0,009720**	0,012200	0,012300	0,019800	0,012700	0,026900	**A**
C4_AB048705	**0,006550**	0,007640	**0,008370**	0,010700	0,010900	0,017800	0,011300	0,024600	**A**
C5_AB241109	0,009590	0,004970	**0,003120**	0,013400	0,011900	0,021900	0,008340	0,027200	C

HBV Reference Sequences Classification by DB rule
S-P gene, amplicon 616 - 969

Subtype and Accession No.	$\phi_A^2(k)$	$\phi_B^2(k)$	$\phi_C^2(k)$	$\phi_D^2(k)$	$\phi_E^2(k)$	$\phi_F^2(k)$	$\phi_G^2(k)$	$\phi_H^2(k)$	DB Rule Type
C5_AP011099	0,008130	0,007780	**0,001730**	0,012100	0,011100	0,023900	0,011000	0,029700	C
C6_AP011102	0,005990	0,003470	**0,001430**	0,011800	0,007840	0,020900	0,008000	0,022100	C
C6_AP011103	0,008410	0,006430	**0,001920**	0,016200	0,011400	0,021700	0,010700	0,023200	C
C7_EU670263	0,006130	0,004680	**0,000505**	0,012900	0,012100	0,023000	0,011300	0,023800	C
C8_AP011104	0,006930	0,006580	**0,001490**	0,012200	0,015100	0,023100	0,011300	0,022700	C
C8_AP011107	0,008770	0,007590	**0,001920**	0,014100	0,015900	0,024800	0,012800	0,023700	C
C9_AP011108	0,005610	0,004220	**0,001390**	0,011000	0,009760	0,022700	0,009220	0,023300	C
D1_X59795	0,008290	0,010900	0,014400	**0,000867**	0,008020	0,018000	0,007310	0,026100	D
D1_X80926	0,009390	0,013900	0,016600	**0,001100**	0,008640	0,019100	0,008900	0,032100	D
D2_X97848	0,008820	0,009550	0,013300	**0,001260**	0,007250	0,016900	0,005570	0,026100	D
D2_Z35716	0,007620	0,010800	0,012400	**0,000367**	0,007410	0,017000	0,007130	0,031100	D
D3_AY233291	0,009570	0,011800	0,011800	**0,001730**	0,010800	0,019700	0,008370	0,025400	D
D3_V01460	0,008010	0,010800	0,011700	**0,000743**	0,007730	0,019000	0,007770	0,026600	D
D3_X65258	0,007940	0,010700	0,013200	**0,002000**	0,011300	0,022300	0,006620	0,032200	D
D4_AB033559	0,006220	0,011000	0,011300	**0,000763**	0,006310	0,014800	0,007130	0,029500	D
D4_AB048702	0,005540	0,009710	0,011300	**0,000614**	0,005320	0,014700	0,006080	0,029500	D
D5_AB033558	0,009150	0,013600	0,012100	**0,005050**	0,011300	0,020300	0,014300	0,030400	D
D5_DQ315779	0,009220	0,012900	0,014100	**0,002850**	0,008580	0,017000	0,010200	0,033400	D
D6_AB493846	0,007930	0,010800	0,012100	**0,000315**	0,007440	0,017400	0,007720	0,026600	D
D6_AB554023	0,008500	0,011600	0,012900	**0,000567**	0,008030	0,018400	0,008320	0,027900	D
D7_AM494716	0,009560	0,014800	0,015400	**0,002460**	0,010100	0,016000	0,010200	0,028400	D
D7_FJ904430	0,007820	0,012600	0,013900	**0,002890**	0,007110	0,015800	0,008250	0,024000	D
E1_X75664	0,007260	0,009300	0,010400	0,007500	**0,000553**	0,018000	0,007150	0,028200	E
E2_X75657	0,006380	0,006820	0,009490	0,006090	**0,000540**	0,015900	0,007170	0,025500	E
E3_AM494694	0,010400	0,010900	0,013000	0,007890	**0,000182**	0,019300	0,008850	0,027300	E
E3_FJ349237	0,008980	0,009500	0,013300	0,006680	**0,000103**	0,017400	0,007660	0,027300	E
E4_FJ349226	0,007780	0,007340	0,012300	0,006680	**0,000153**	0,017000	0,006590	0,028500	E
E4_HM363569	0,009010	0,009070	0,011600	0,008870	**0,000212**	0,018000	0,008260	0,031100	E
E5_DQ060828	0,009690	0,009990	0,012600	0,007290	**0,000177**	0,016500	0,007650	0,032400	E
E5_JQ000008	0,011100	0,010600	0,012800	0,006580	**0,000327**	0,018400	0,008240	0,032400	E
F1_AY090459	0,018500	0,021800	0,019900	0,016500	0,017100	**0,002280**	0,023900	0,015500	F
F1_HE981184	0,016300	0,019100	0,020900	0,016200	0,013800	**0,001230**	0,021700	0,011200	F
F1_HM590471	0,016400	0,018900	0,020200	0,016300	0,013700	**0,000954**	0,021700	0,010500	F
F2_AY090455	0,023900	0,023400	0,024600	0,017900	0,018500	**0,001420**	0,024900	0,012700	F
F2_AY311369	0,023200	0,021600	0,023800	0,016300	0,018500	**0,001590**	0,022700	0,011900	F
F2_X69798	0,022200	0,024400	0,021400	0,016200	0,016700	**0,001210**	0,023800	0,012700	F
F3_AB036910	0,020900	0,018300	0,021700	0,017500	0,017600	**0,000843**	0,021700	0,009450	F
F3_AB036915	0,021000	0,019500	0,022600	0,019500	0,020500	**0,000460**	0,024900	0,009400	F
F3_FJ589066	0,021900	0,020500	0,023800	0,018400	0,021500	**0,001240**	0,026000	0,011900	F
F3_X75663	0,019900	0,019700	0,022000	0,018700	0,019500	**0,000781**	0,026100	0,010100	F
F4_AB166850	0,021100	0,021500	0,024800	0,017000	0,019600	**0,000827**	0,028500	0,011400	F
F4_DQ823090	0,020200	0,019600	0,022400	0,017000	0,017900	**0,000493**	0,026100	0,010900	F
F4_EU366116	0,021200	0,020600	0,023600	0,016100	0,018900	**0,000653**	0,027300	0,011700	F
F4_HE974368	0,024600	0,022400	0,024600	0,018500	0,021800	**0,000615**	0,026100	0,011700	F
G0_EF464098	0,006340	0,006220	0,008180	0,006990	0,007700	0,024600	**0,000104**	0,026800	G
G0_HE981172	0,007140	0,006970	0,009390	0,007160	0,007690	0,023400	**0,000053**	0,027900	G
G0_HE981176	0,006580	0,006440	0,008770	0,006580	0,007130	0,023400	**0,000008**	0,028000	G
H0_AB275308	0,023300	0,019800	0,022700	0,026500	0,028600	0,010300	0,028100	**0,000076**	H
H0_AB516395	0,023800	0,020000	0,022100	0,026800	0,027400	0,011200	0,024600	**0,000701**	H
H0_AP007261	0,025500	0,021800	0,024800	0,029000	0,031100	0,010800	0,030500	**0,000312**	H
I0_FJ023660	0,004360	0,004920	0,006160	0,005360	0,007610	0,020300	0,003150	0,021700	I
I0_FJ023664	0,003380	0,003960	0,005540	0,004390	0,006380	0,015600	0,003160	0,020900	I

HBV Reference Sequences Classification by DB rule

S-P gene, amplicon 616 - 969

Subtype and Accession No.	$\phi_A^2(k)$	$\phi_B^2(k)$	$\phi_C^2(k)$	$\phi_D^2(k)$	$\phi_E^2(k)$	$\phi_F^2(k)$	$\phi_G^2(k)$	$\phi_H^2(k)$	DB Rule Type
J0_AB486012	0,007280	0,006740	0,007960	0,011800	0,012000	0,022200	0,008490	0,026800	B

$$\hat{\phi}_I^2(k) = \frac{1}{n_I} \sum_{i \in I} d_{i,k}^2 - \frac{1}{2 n_I^2} \sum_{i,j \in I} d_{i,j}^2$$

UPGMA tree (K80): HBV 616:969

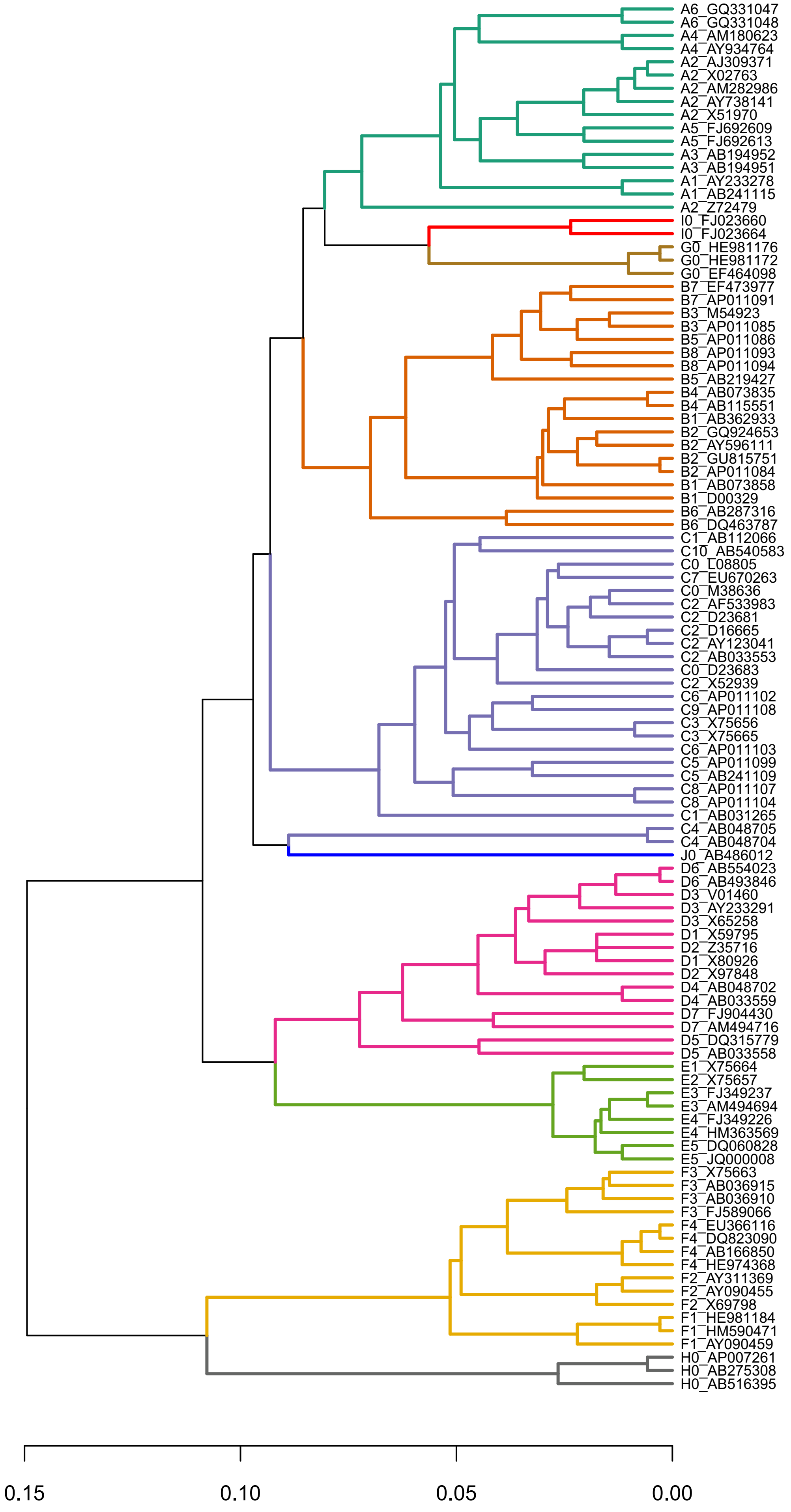

MDS map (K80): HBV 616:969

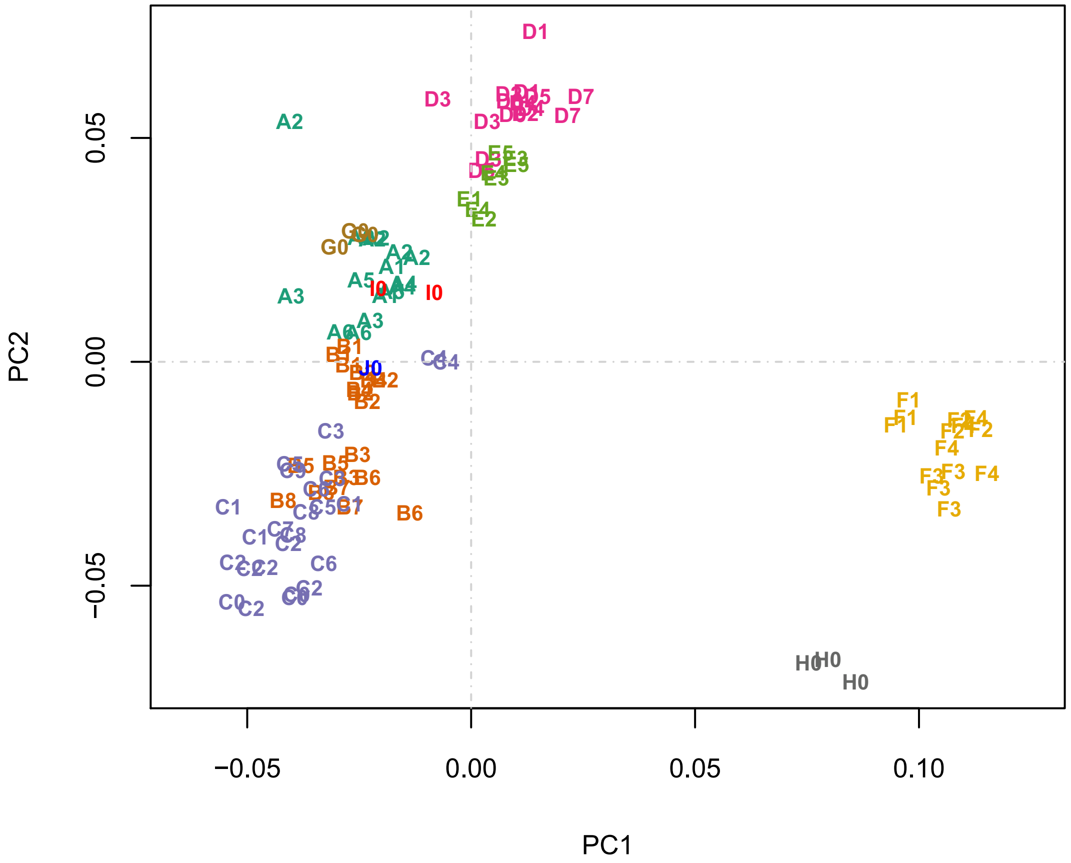

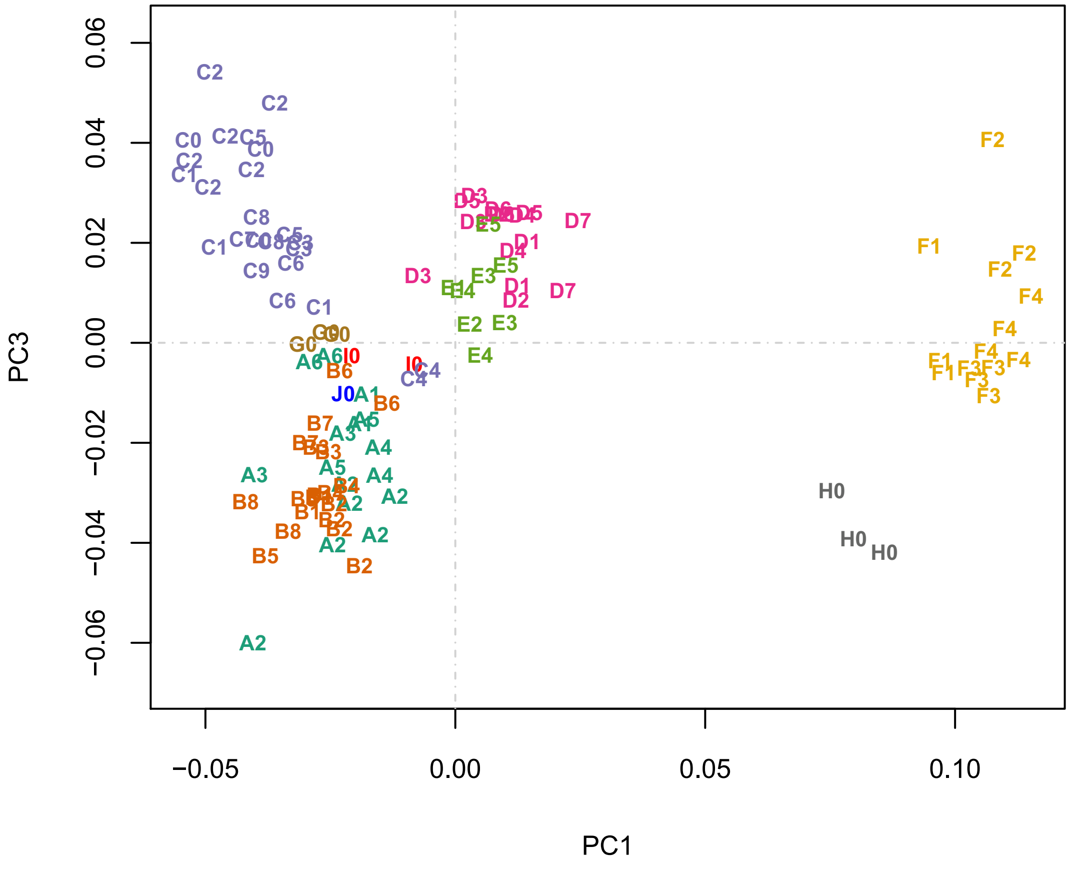

Supplement: S2 File — (PDF) [file pone.0144816.s002.pdf]
