# Supplementary material for: Complex Genotype Mixtures Analyzed by Deep Sequencing in Two Different Regions of Hepatitis B Virus
Source: PLoS One. 2015 Dec 29;10(12):e0144816. doi: 10.1371/journal.pone.0144816 (PMC4695080; doi:10.1371/journal.pone.0144816)

HBV reference sequences by genotype, with accessions, used in genotyping region 1596 to 1912

A	B	C	D	F
A2_AJ309371	B1_AB073858	C2_X52939	DE_E1_X75664	F3_X75663
A2_X02763	B1_AB362933	C2_D23681	DE_E3_FJ349237	F3_AB036910
A2_X51970	B2_GQ924653	C2_AY123041	DE_E3_AM494694	F2_AY311369
A2_Z72479	B2_GU815751	C0_D23683	DE_E4_HM363569	F2_X69798
A1_AY233278	B1_D00329	C0_L08805	DE_E4_FJ349226	F4_HE974368
A1_AB241115	B2_AY596111	C3_X75656	DE_E2_X75657	F4_EU366116
A2_AY738141	B2_AP011084	C1_AB112066	DE_E5_DQ060828	F1_HE981184
A3_AB194952	B3_M54923	C1_AB031265	DE_E5_JQ000008	F1_HM590471
A3_AB194951	B3_AP011085	C2_AB033553	DE_D3_V01460	F1_DQ823095
A4_AY934764	B4_AB073835	C2_AF533983	DE_D1_X59795	F1_AY090459
A5_FJ692609	B4_AB115551	C4_AB048705	DE_D2_X97848	F2_AY090455
A5_FJ692613	B5_AB219427	C4_AB048704	DE_D1_X80926	F3_AB036915
A6_GQ331047	B5_AP011086	C5_AP011099	DE_D2_Z35716	F4_DQ823090
A6_GQ331048	B6_AB287316	C5_AB241109	DE_D3_AY233291	F4_AB166850
	B6_DQ463787	C6_AP011102	DE_D4_AB048702	
	B7_EF473977	C6_AP011103	DE_D4_AB033559	
	B7_AP011091	C7_EU670263	DE_D5_DQ315779	
	B8_AP011093	C8_AP011107	DE_D5_AB033558	
	B8_AP011094	C8_AP011104	DE_D7_FJ904430	
		C9_AP011108	DE_D7_AM494716	
		C10_AB540583		
14	19	21	20	14

HBV Reference Sequences Discriminating Power

HBV - X Gene, amplicon 1596 to 1912

Genotype	RefSeq No.	Geometric Variability	Mean Squared Distances Within Genotype: Min	Median	Max	Max Corrected	Nearest Dif. Gen.	Nearest Genotype
A	14	0,000430	0,000538	0,000759	0,002380	0,001950	0,002190	C
B	19	0,000435	0,000467	0,000731	0,002980	0,002545	0,001620	C
C	21	0,000748	0,000886	0,001280	0,002960	0,002212	0,001420	I
D	12	0,000547	0,000577	0,001180	0,002110	0,001563	0,000541	E
E	8	0,000032	0,000039	0,000070	0,000162	0,000130	0,000062	D
F	14	0,000601	0,000671	0,001110	0,002660	0,002059	0,004800	H
G	4	0,000018	0,000020	0,000047	0,000075	0,000057	0,048300	J
H	2	0,000264	0,001060	0,001060	0,001060	0,000796	0,011200	F
I	2	0,000166	0,000665	0,000665	0,000665	0,000499	0,000924	C

Mean squared distances within genotype $$\frac{1}{n_I - 1} \sum_{i \in I} d^2_{i,j}; \quad j: 1..n_I$$

Genotype geometric variability $$\hat{V}_I = \frac{1}{2 n_I^2} \sum_{i,j \in I} d^2_{i,j}$$

DB rule $$\min_I \left(\hat{\phi}_I^2(k) = \frac{1}{n_I} \sum_{i \in I} d^2_{i,k} - \hat{V}_I \right)$$

HBV Reference Sequences Classification by DB rule
X gene, amplicon 1596 to 1912

Subtype and Accession No.	$\phi_A^2(k)$	$\phi_B^2(k)$	$\phi_C^2(k)$	$\phi_D^2(k)$	$\phi_E^2(k)$	$\phi_F^2(k)$	$\phi_G^2(k)$	$\phi_H^2(k)$	DB Rule Type
A1_AB241115	**0,000163**	0,005580	0,004230	0,003510	0,003020	0,017300	0,055000	0,023000	A
A1_AY233278	**0,000171**	0,005580	0,004250	0,003510	0,003020	0,017300	0,055000	0,023000	A
A2_AJ309371	**0,000465**	0,005390	0,003940	0,003410	0,003420	0,015500	0,045300	0,018700	A
A2_AY738141	**0,000234**	0,004470	0,003660	0,002850	0,002670	0,014200	0,051100	0,019600	A
A2_X02763	**0,000234**	0,004470	0,003660	0,002850	0,002670	0,014200	0,051100	0,019600	A
A2_X51970	**0,000081**	0,003990	0,003210	0,002460	0,002330	0,013300	0,049100	0,018500	A
A2_Z72479	**0,000804**	0,006060	0,005160	0,004160	0,003860	0,017100	0,055100	0,023000	A
A3_AB194951	**0,000566**	0,006630	0,005010	0,004630	0,004260	0,018300	0,051000	0,022700	A
A3_AB194952	**0,000814**	0,007730	0,005520	0,005230	0,004750	0,021700	0,054800	0,025100	A
A4_AY934764	**0,000176**	0,005570	0,004240	0,003440	0,003020	0,016400	0,054400	0,022900	A
A5_FJ692609	**0,000630**	0,005660	0,004430	0,003630	0,003410	0,018900	0,052800	0,025200	A
A5_FJ692613	**0,000168**	0,005570	0,004210	0,003500	0,003020	0,017200	0,054800	0,022900	A
A6_GQ331047	**0,000404**	0,004080	0,003250	0,002380	0,002070	0,014200	0,049000	0,015500	A
A6_GQ331048	**0,002070**	0,007250	0,006270	0,005260	0,004380	0,017500	0,057100	0,023100	A
B1_AB073858	0,006520	**0,002660**	0,004410	0,006370	0,006430	0,015200	0,053600	0,018100	B
B1_AB362933	0,005510	**0,000166**	0,002440	0,003940	0,003410	0,009790	0,047900	0,017300	B
B1_D00329	0,004720	**0,000307**	0,002340	0,003740	0,002970	0,009960	0,053600	0,016300	B
B2_AP011084	0,005020	**0,000009**	0,001720	0,003670	0,003050	0,010800	0,049500	0,016300	B
B2_AY596111	0,006110	**0,000222**	0,002310	0,004540	0,003860	0,011800	0,051400	0,016300	B
B2_GQ924653	0,005560	**0,000288**	0,001890	0,004060	0,003450	0,011000	0,053300	0,015300	B
B2_GU815751	0,004520	**0,000193**	0,001700	0,003640	0,003050	0,010500	0,051200	0,015300	B
B3_AP011085	0,004530	**0,000143**	0,002030	0,003940	0,003450	0,010200	0,043900	0,014400	B
B3_M54923	0,005020	**0,000300**	0,002390	0,004410	0,003870	0,011000	0,045600	0,015300	B
B4_AB073835	0,004280	**0,000091**	0,001380	0,002850	0,002690	0,009270	0,045600	0,014400	B
B4_AB115551	0,007320	**0,001270**	0,004370	0,005660	0,004780	0,014000	0,057800	0,019800	B
B5_AB219427	0,006890	**0,000823**	0,003760	0,005920	0,005820	0,014800	0,043800	0,018200	B
B5_AP011086	0,005830	**0,000273**	0,002220	0,004550	0,003870	0,012400	0,047900	0,016300	B
B6_AB287316	0,004910	**0,000639**	0,002330	0,003270	0,003070	0,008370	0,051700	0,016300	B
B6_DQ463787	0,005810	**0,000585**	0,002940	0,004060	0,003870	0,010400	0,045800	0,015200	B
B7_AP011091	0,005220	**0,000111**	0,001430	0,003180	0,002690	0,009490	0,051700	0,015300	B
B7_EF473977	0,006140	**0,000333**	0,001720	0,003680	0,003060	0,010900	0,045800	0,016300	B
B8_AP011093	0,006280	**0,000563**	0,002140	0,004320	0,003870	0,010100	0,053300	0,013600	B
B8_AP011094	0,005830	**0,000231**	0,002000	0,003790	0,003870	0,011000	0,049400	0,014300	B
C0_D23683	0,005320	0,003720	**0,001240**	0,002290	0,002280	0,015600	0,061600	0,021500	C
C0_L08805	0,005910	0,003850	**0,002280**	0,003050	0,003320	0,013800	0,047300	0,015100	C
C1_AB031265	0,003430	**0,001200**	**0,001320**	0,002690	0,001890	0,008260	0,057600	0,012900	**B**
C1_AB112066	0,006310	0,005460	**0,002110**	0,004840	0,004190	0,015200	0,064600	0,020500	C
C10_AB540583	0,006440	0,003990	**0,001280**	0,003470	0,003350	0,015100	0,045200	0,018600	C
C2_AB033553	0,003870	0,002140	**0,000106**	0,001160	0,000947	0,011600	0,051000	0,017200	C
C2_AF533983	0,003640	0,002420	**0,000247**	0,001320	0,001410	0,011600	0,053100	0,016100	C
C2_AY123041	0,004320	0,002450	**0,000417**	0,001410	0,001160	0,012300	0,053000	0,018300	C
C2_D23681	0,007020	0,004430	**0,001380**	0,002500	0,002610	0,016700	0,047000	0,019100	C
C2_X52939	0,004320	0,002390	**0,000190**	0,001410	0,001410	0,012800	0,047100	0,017100	C
C3_X75656	0,004830	0,003320	**0,001110**	0,001970	0,001470	0,013000	0,057100	0,019300	**I**
C4_AB048704	0,007210	0,003640	**0,001890**	0,003880	0,003480	0,017000	0,068600	0,022700	**I**
C4_AB048705	0,006470	0,002910	**0,001210**	0,003110	0,002350	0,014400	0,066000	0,020500	**I**
C5_AB241109	0,001600	0,002490	**0,000521**	0,000791	0,000751	0,012200	0,049200	0,019500	C
C5_AP011099	0,002260	0,002490	**0,000521**	0,000791	0,000751	0,012200	0,053300	0,019500	C
C6_AP011102	0,003870	0,001540	**0,000242**	0,001920	0,001670	0,010300	0,061800	0,016300	C
C6_AP011103	0,005330	0,002160	**0,000627**	0,002340	0,001660	0,012600	0,057400	0,017300	**I**
C7_EU670263	0,003890	0,001630	**0,000119**	0,001740	0,001410	0,011600	0,053100	0,017400	C
C8_AP011104	0,003990	0,001620	**0,000153**	0,001880	0,001670	0,009850	0,055300	0,014500	C
C8_AP011107	0,004360	0,001950	**0,000238**	0,002070	0,001670	0,010900	0,057400	0,016400	C
C9_AP011108	0,003870	0,001100	**0,000131**	0,001730	0,001400	0,010100	0,053300	0,015400	C
D1_X59795	0,004550	0,004560	0,002630	**0,000891**	0,001050	0,017400	0,049200	0,023100	D
D1_X80926	0,002590	0,003300	0,001910	**0,000942**	0,000974	0,011300	0,049100	0,020500	D
D2_X97848	0,003000	0,003380	0,001080	0,000137	**0,000076**	0,013000	0,049700	0,019300	E
D2_Z35716	0,004830	0,004360	0,001730	0,000614	**0,000338**	0,015600	0,049400	0,021500	E
D3_AY233291	0,002610	0,004180	0,001550	0,000250	**0,000151**	0,014000	0,049500	0,019400	E

HBV Reference Sequences Classification by DB rule
X gene, amplicon 1596 to 1912

Subtype and Accession No.	$\phi_A^2(k)$	$\phi_B^2(k)$	$\phi_C^2(k)$	$\phi_D^2(k)$	$\phi_E^2(k)$	$\phi_F^2(k)$	$\phi_G^2(k)$	$\phi_H^2(k)$	DB Rule Type
D3_V01460	0,002620	0,002920	0,001070	**0,000167**	0,000197	0,011400	0,049100	0,018200	D
D4_AB033559	0,002960	0,003380	0,001310	0,000156	**0,000040**	0,012200	0,050800	0,019400	E
D4_AB048702	0,003010	0,003380	0,001070	**-0,000021**	0,000022	0,012900	0,049500	0,019400	D
D5_AB033558	0,003860	0,004710	0,002780	**0,001100**	0,001180	0,012300	0,049100	0,015300	D
D5_DQ315779	0,005380	0,006620	0,004020	**0,001650**	0,001680	0,013000	0,047500	0,014400	D
D7_AM494716	0,003730	0,004130	0,001820	0,000655	**0,000468**	0,014700	0,051000	0,018100	E
D7_FJ904430	0,005820	0,006680	0,003100	**0,001280**	0,001410	0,019600	0,045600	0,022500	D
E1_X75664	0,002620	0,002980	0,001030	**-0,000005**	0,000003	0,012000	0,049400	0,018300	D
E2_X75657	0,003310	0,003830	0,001580	0,000223	**0,000049**	0,013100	0,051200	0,018300	E
E3_AM494694	0,002260	0,002590	0,000809	**-0,000051**	0,000019	0,011200	0,047400	0,017300	D
E3_FJ349237	0,003010	0,003330	0,001290	0,000134	**0,000046**	0,012900	0,051200	0,019400	E
E4_FJ349226	0,003310	0,003830	0,001360	0,000204	**0,000029**	0,013800	0,049500	0,018300	E
E4_HM363569	0,002910	0,003820	0,001420	0,000321	**0,000143**	0,013700	0,049400	0,018300	E
E5_DQ060828	0,003020	0,003390	0,001320	0,000119	**0,000046**	0,012000	0,047400	0,019400	E
E5_JQ000008	0,002620	0,002980	0,001030	**-0,000005**	0,000003	0,012000	0,049400	0,018300	D
F1_AY090459	0,014900	0,014100	0,014400	0,013400	0,013300	**0,001110**	0,060700	0,007080	F
F1_DQ823095	0,014600	0,011100	0,012600	0,012200	0,011700	**0,000901**	0,056400	0,006450	F
F1_HE981184	0,014600	0,010700	0,012600	0,012500	0,011700	**0,001350**	0,056400	0,007680	F
F1_HM590471	0,012800	0,009610	0,011100	0,012200	0,011700	**0,000479**	0,056600	0,005360	F
F2_AY090455	0,017500	0,011500	0,012300	0,014900	0,013200	**0,000880**	0,063800	0,006640	F
F2_AY311369	0,016500	0,010700	0,011400	0,013700	0,012400	**0,000280**	0,063800	0,006060	F
F2_X69798	0,015400	0,011300	0,012300	0,014000	0,013200	**0,000152**	0,060900	0,005510	F
F3_AB036910	0,015400	0,010400	0,013800	0,015800	0,015000	**0,000490**	0,059100	0,006060	F
F3_AB036915	0,016000	0,009650	0,013100	0,015200	0,014100	**0,000337**	0,057100	0,005530	F
F3_X75663	0,015200	0,010900	0,012100	0,014200	0,013200	**0,000224**	0,061400	0,006640	F
F4_AB166850	0,022600	0,013500	0,014000	0,016300	0,015900	**0,002160**	0,054600	0,008810	F
F4_DQ823090	0,017200	0,009710	0,010300	0,012100	0,010800	**0,000026**	0,057100	0,005400	F
F4_EU366116	0,020400	0,011900	0,012600	0,014600	0,013200	**0,000496**	0,059100	0,006400	F
F4_HE974368	0,022400	0,012700	0,013600	0,015300	0,015000	**0,000857**	0,065300	0,007580	F
G0_AF160501	0,053500	0,050600	0,056000	0,049900	0,050700	0,060400	**0,000032**	0,053700	G
G0_EF464098	0,051700	0,048700	0,054000	0,048200	0,048900	0,059100	**-0,000005**	0,051700	G
G0_HE981172	0,049600	0,046800	0,051900	0,046200	0,046900	0,056900	**0,000032**	0,049700	G
G0_HE981176	0,053700	0,050800	0,056200	0,050200	0,050900	0,059100	**0,000068**	0,051700	G
H0_AB179747	0,016300	0,012500	0,014300	0,015700	0,014500	0,003850	0,055100	**0,001060**	H
H0_AB516395	0,026600	0,019300	0,020400	0,022300	0,022900	0,008500	0,048900	**0,001060**	H
I0_FJ023660	0,004250	0,001550	**0,000603**	0,002220	0,001740	0,010300	0,061100	0,017200	C
I0_FJ023664	0,006090	0,002910	0,001150	0,003040	0,002510	0,013500	0,059500	0,019300	I
J0_AB486012	0,016200	0,011700	0,011400	0,014100	0,012600	0,011000	0,036200	0,010900	H

$$\hat{\phi}_I^2(k) = \frac{1}{n_I}\sum_{i\in I} d_{i,k}^2 - \frac{1}{2\,n_I^2}\sum_{i,j\in I} d_{i,j}^2$$

UPGMA tree (K80): HBV 1596:1912

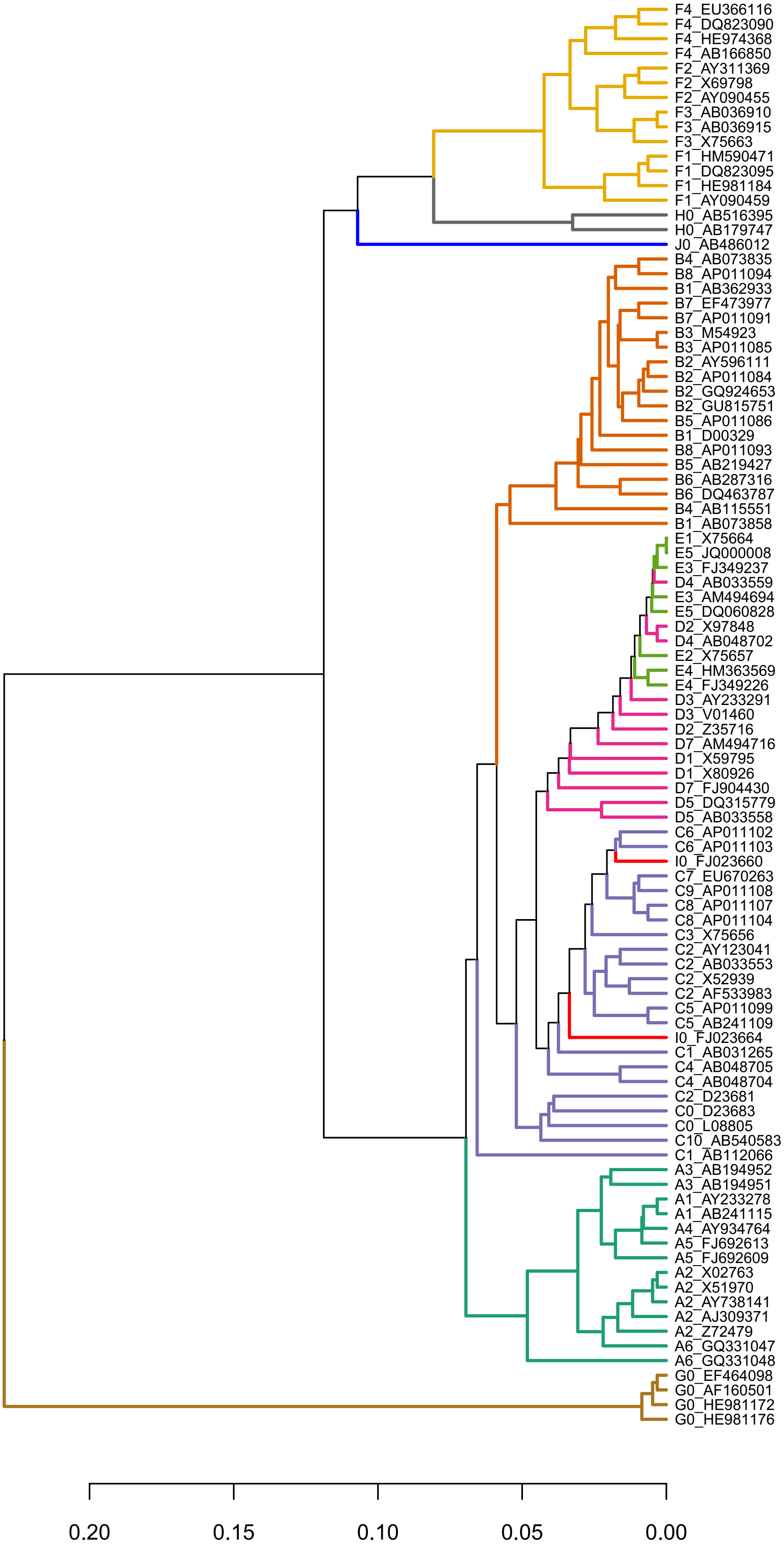

MDS map (K80): HBV 1596:1912

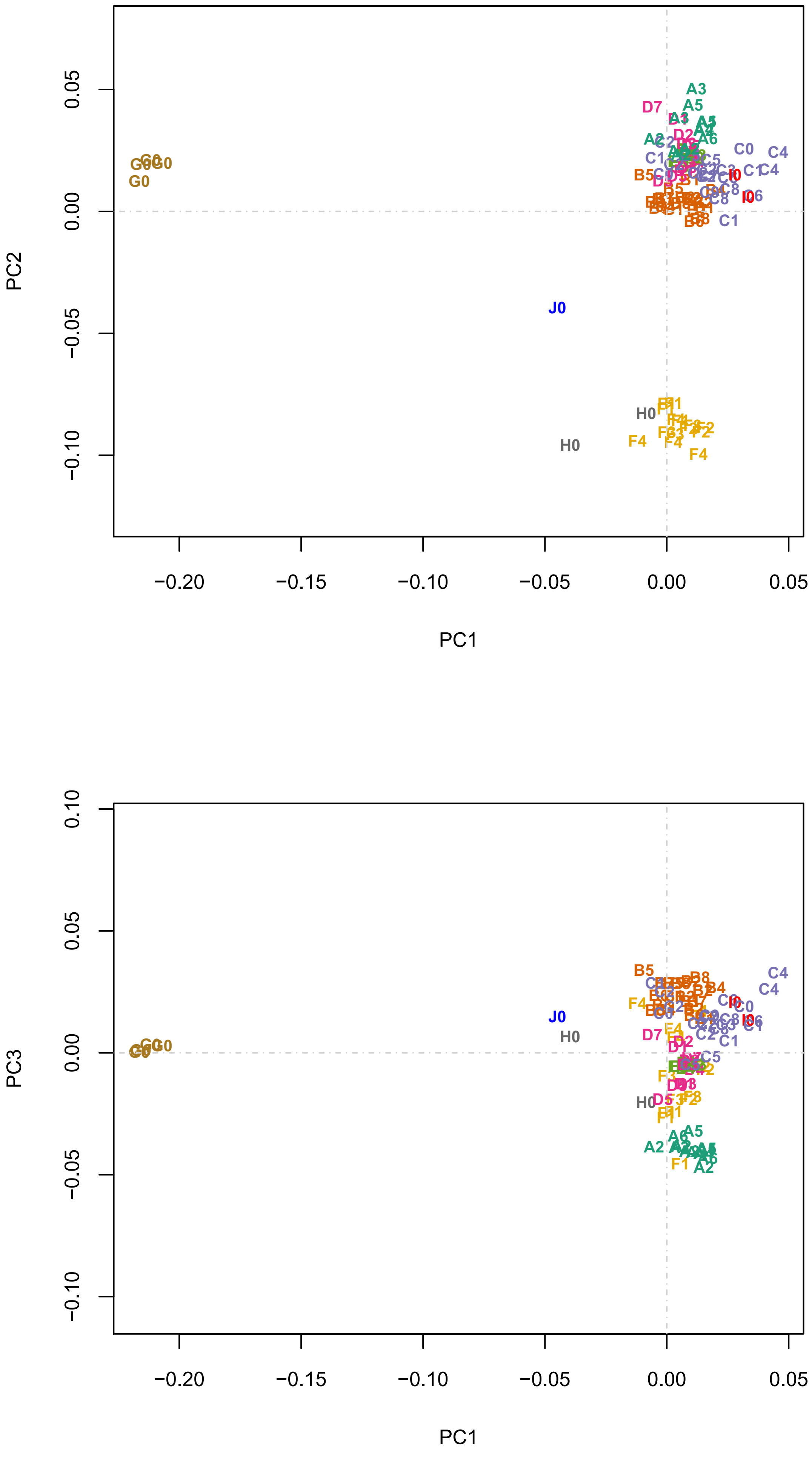

Supplement: S3 File — (PDF) [file pone.0144816.s003.pdf]
