# Supplementary material for: Complex Genotype Mixtures Analyzed by Deep Sequencing in Two Different Regions of Hepatitis B Virus
Source: PLoS One. 2015 Dec 29;10(12):e0144816. doi: 10.1371/journal.pone.0144816 (PMC4695080; doi:10.1371/journal.pone.0144816)
Supplement: S4 File — (PDF) [file pone.0144816.s004.pdf]

UPGMA tree (K80): Pt01 First sample P/S region

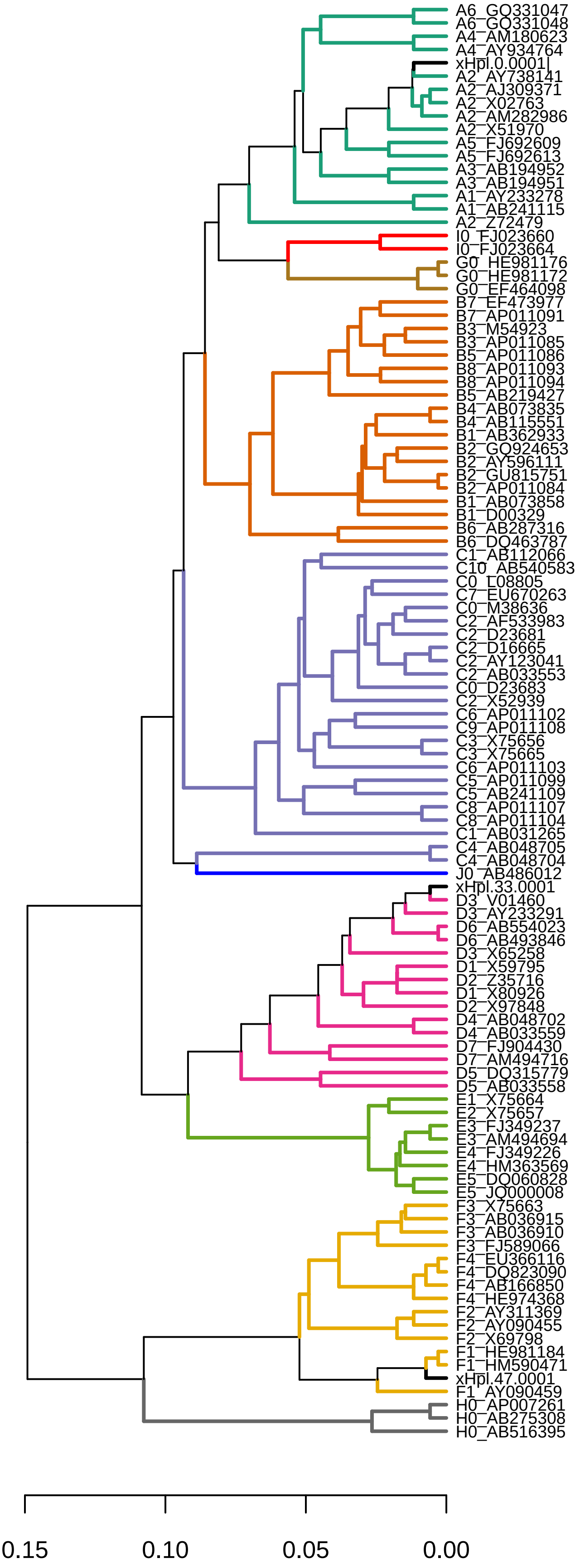

MDS map (K80): Pt01 First sample P/S region

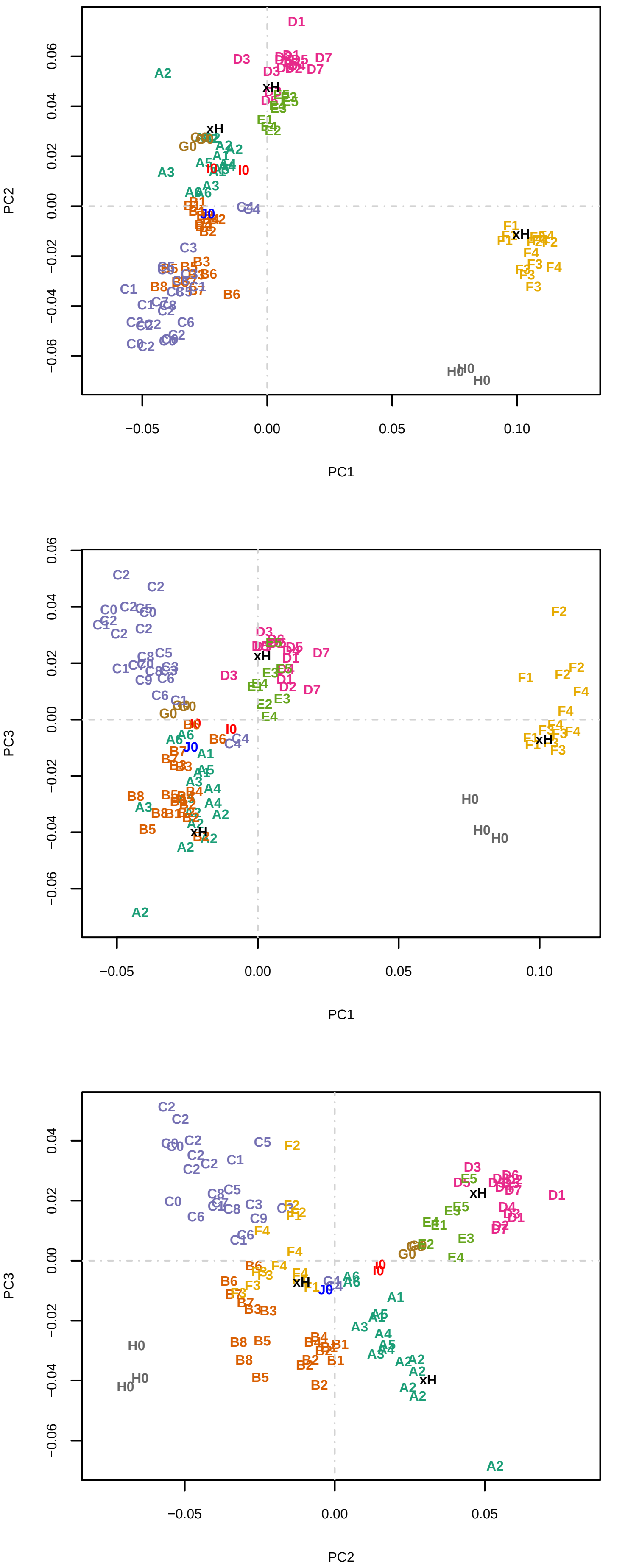

UPGMA tree (K80): Pt01 Second sample P/S region

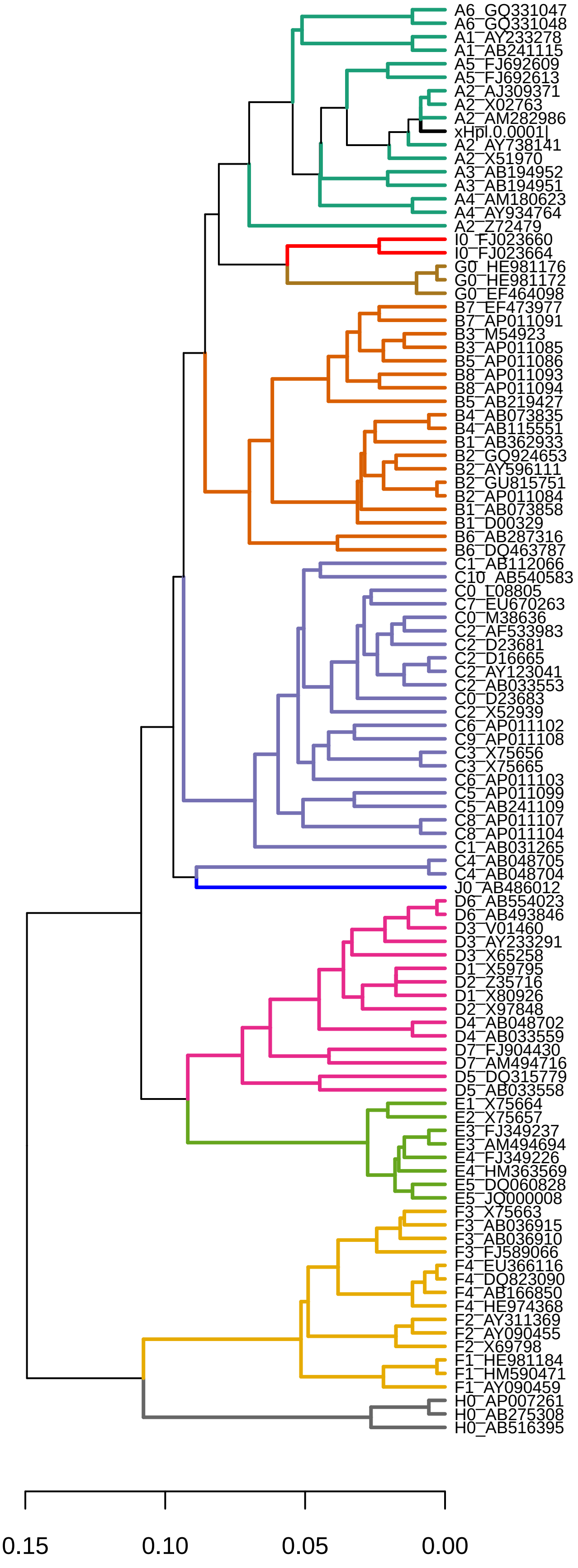

MDS map (K80): Pt01 Second sample P/S region

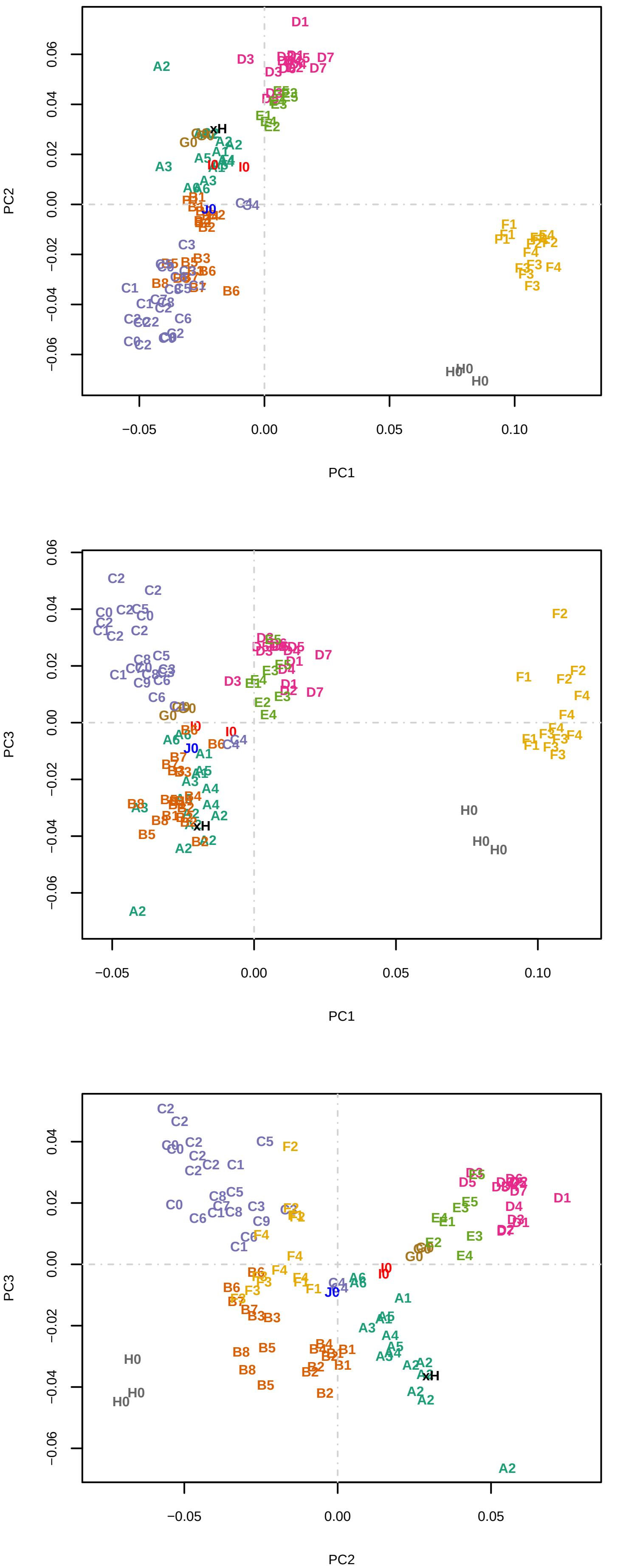

UPGMA tree (K80): Pt01 Third sample P/S region

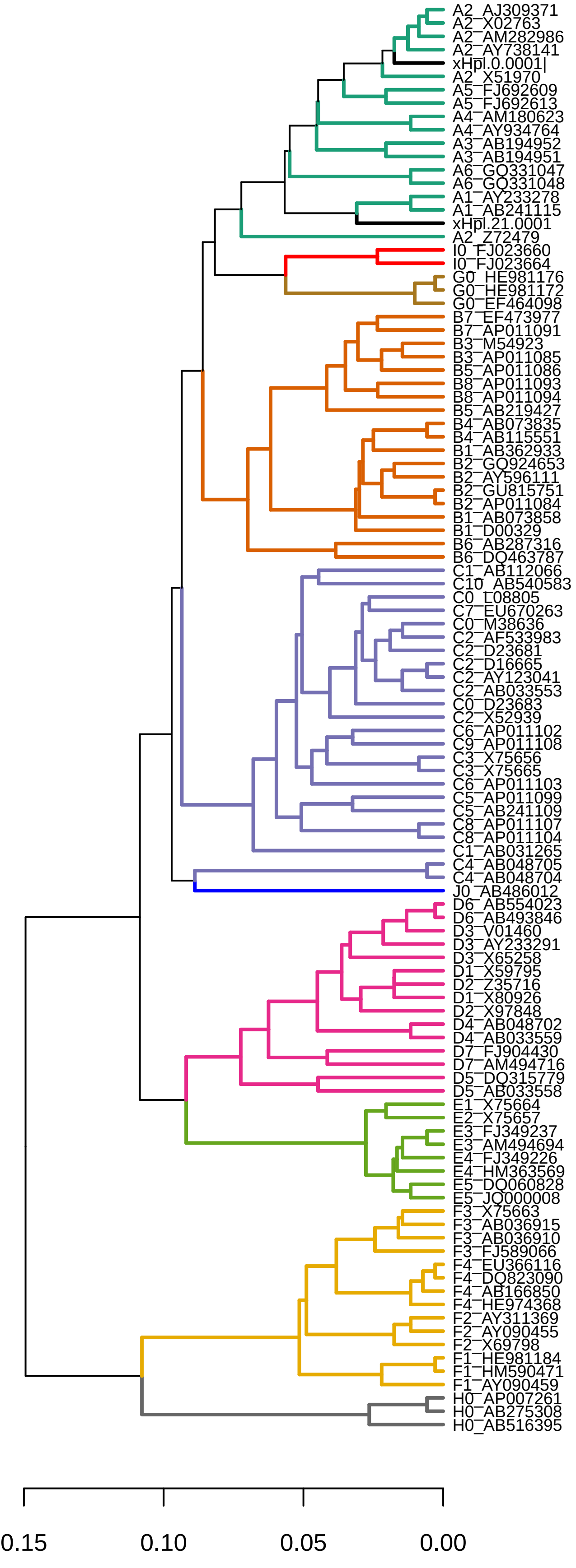

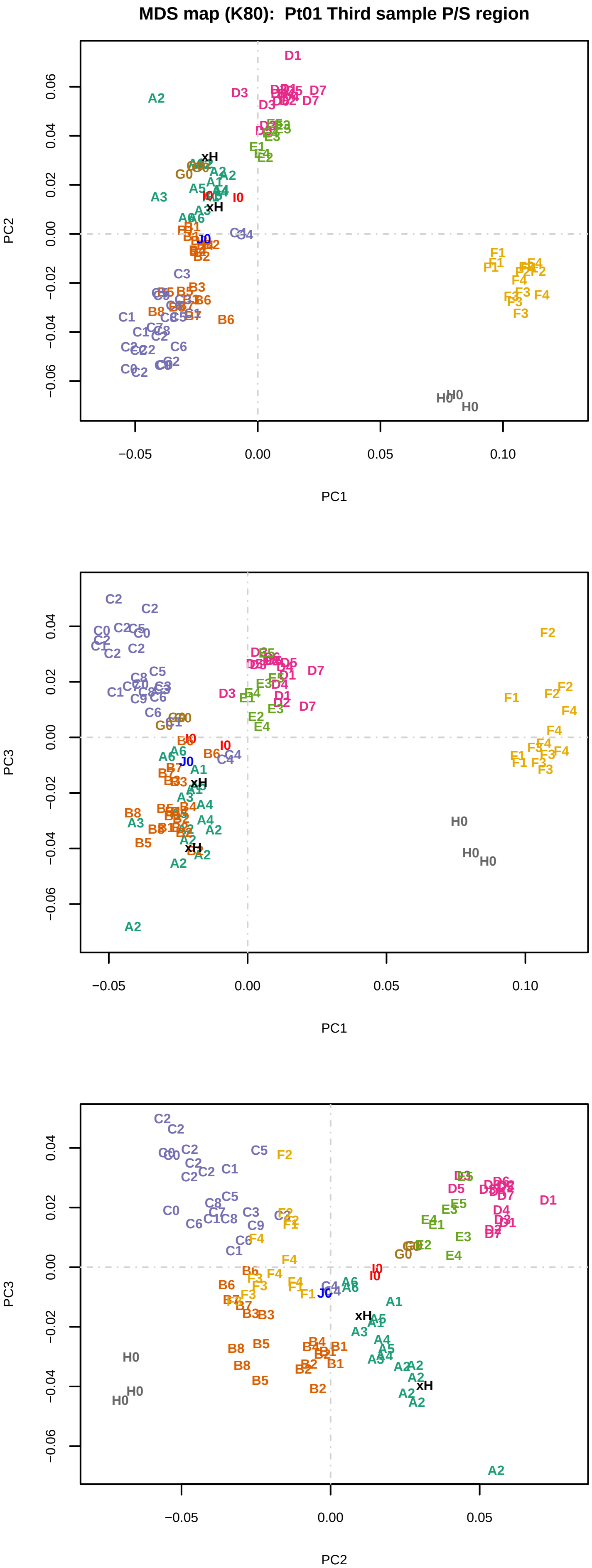
MDS map (K80): Pt01 Third sample P/S region
PC1
PC2
PC1
PC3
PC2
PC3

UPGMA tree (K80): Pt02 First sample P/S region

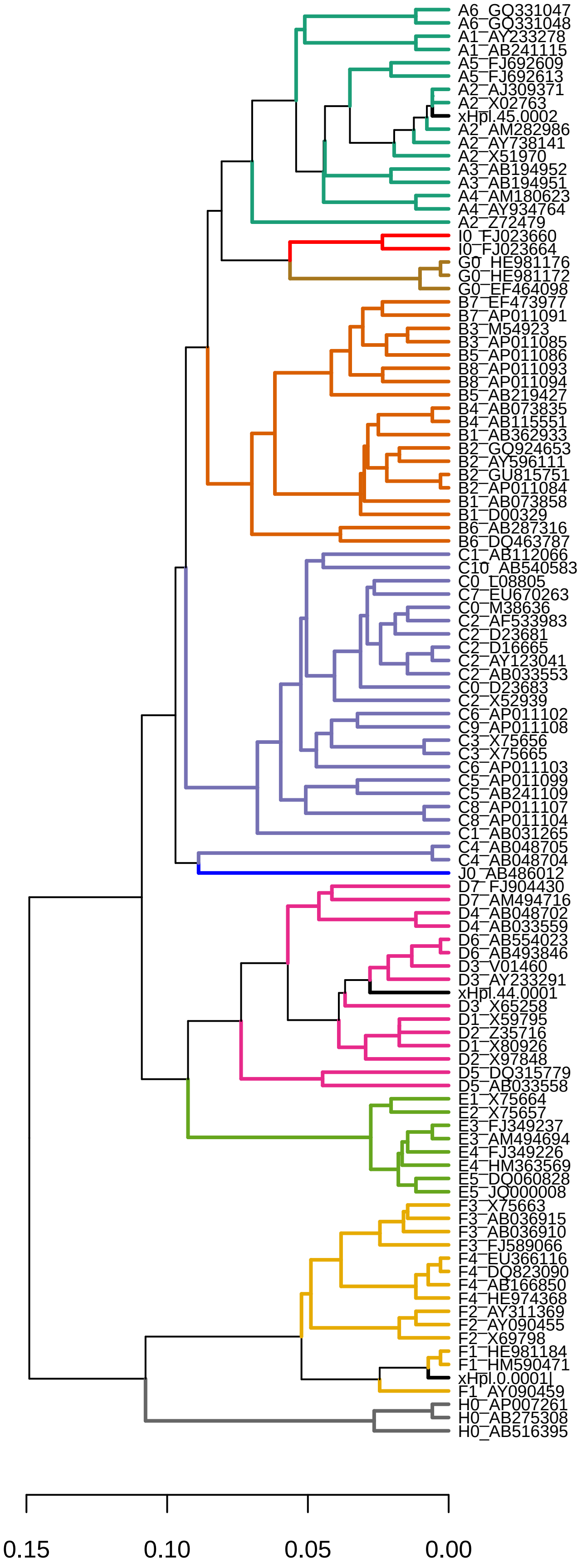

MDS map (K80): Pt02 First sample P/S region

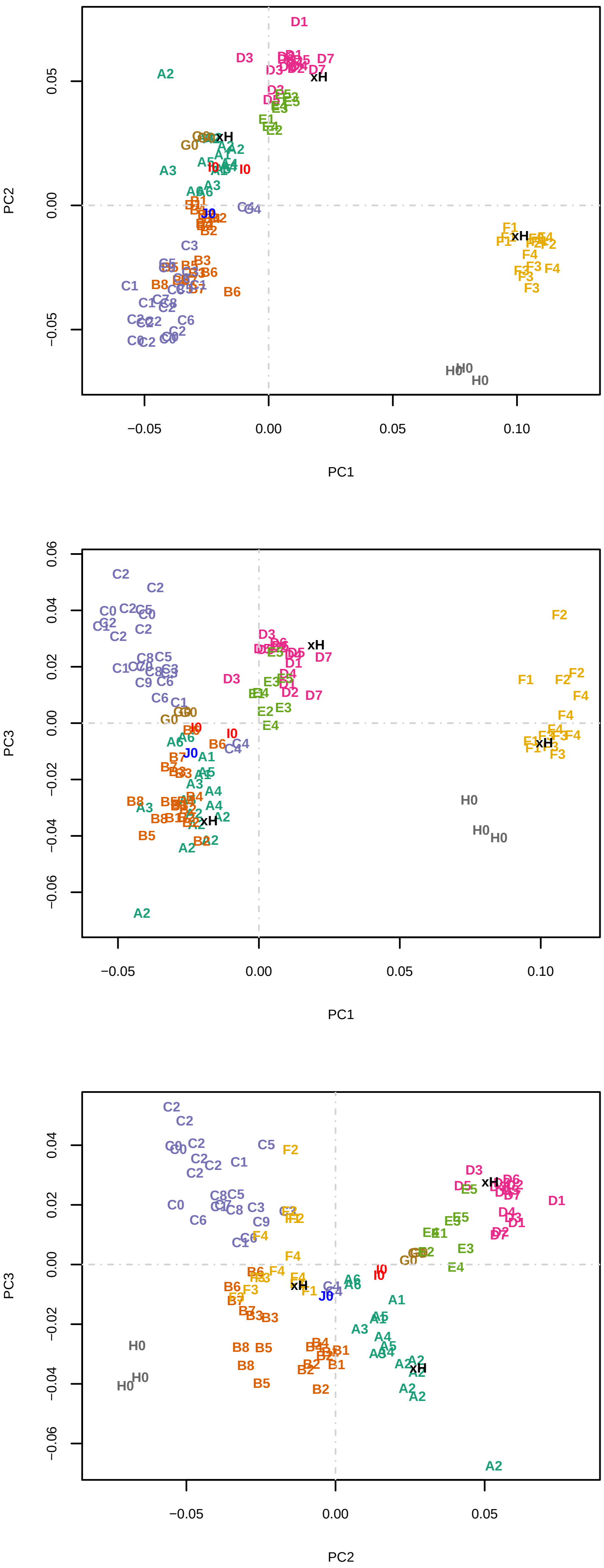

UPGMA tree (K80): Pt02 Second sample P/S region

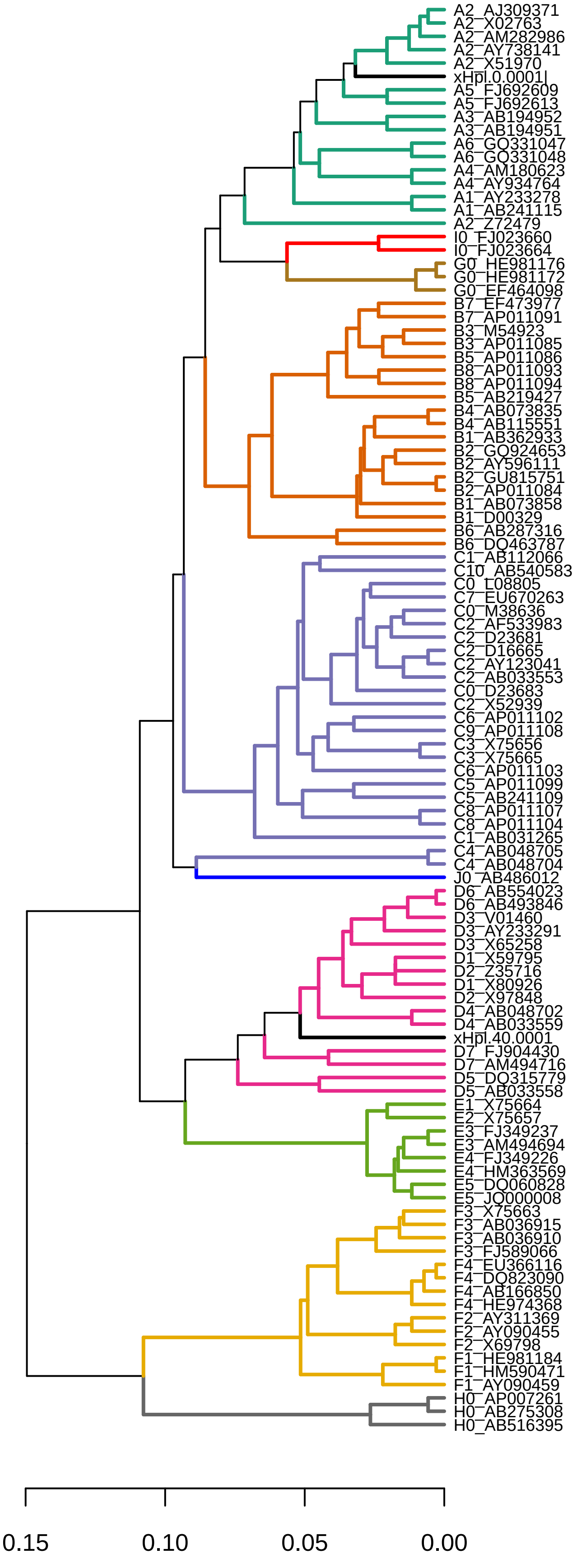

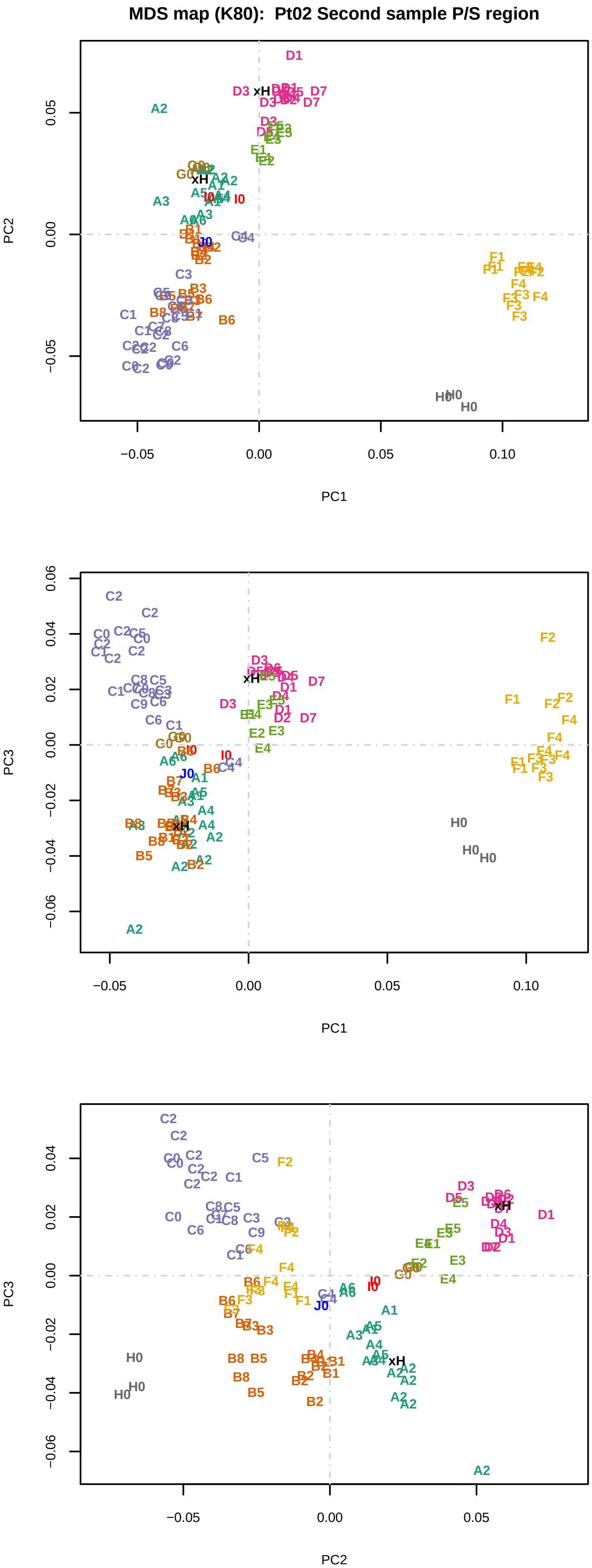

MDS map (K80): Pt02 Second sample P/S region
PC2
0.05
0.00
−0.05
−0.05
0.00
0.05
0.10
PC1
PC3
0.06
0.04
0.02
0.00
−0.02
−0.04
−0.06
−0.05
0.00
0.05
0.10
PC1
PC3
0.04
0.02
0.00
−0.02
−0.04
−0.06
−0.05
0.00
0.05
PC2

UPGMA tree (K80): Pt02 Third sample P/S region

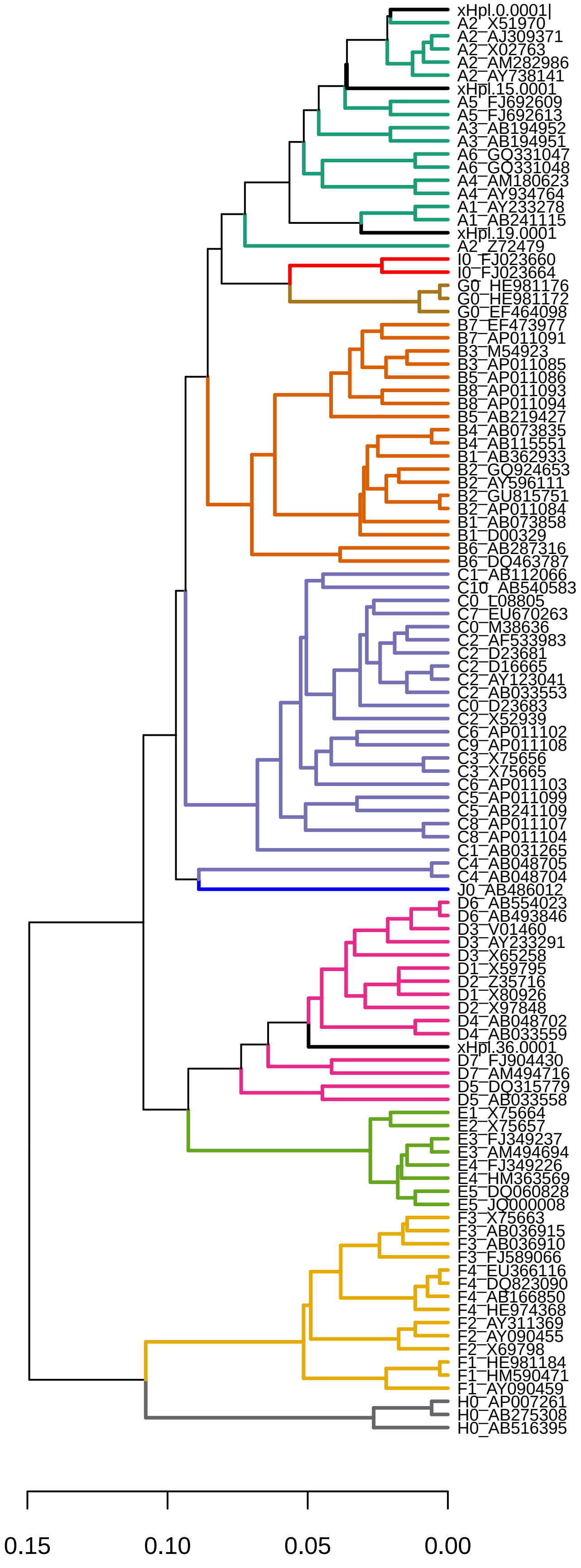

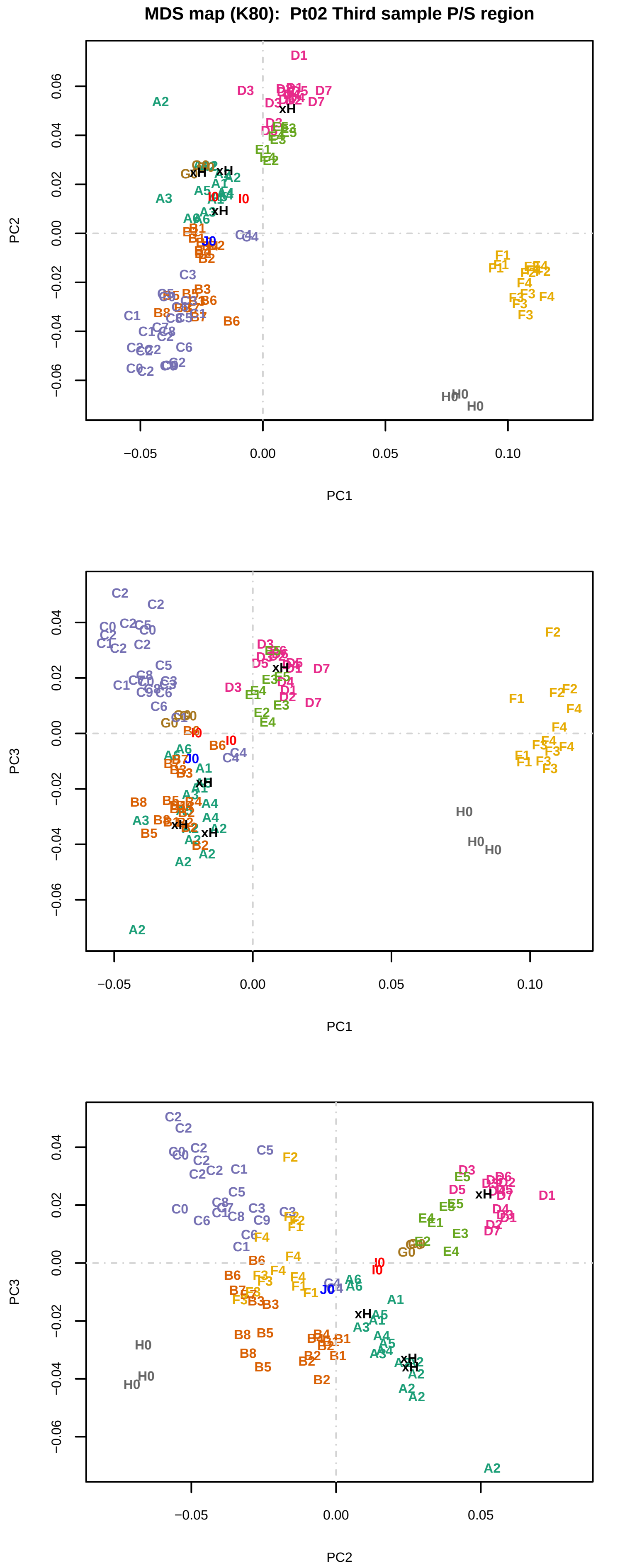
MDS map (K80): Pt02 Third sample P/S region
PC1
PC2
PC1
PC3
PC2
PC3

UPGMA tree (K80): Pt03 First sample P/S region

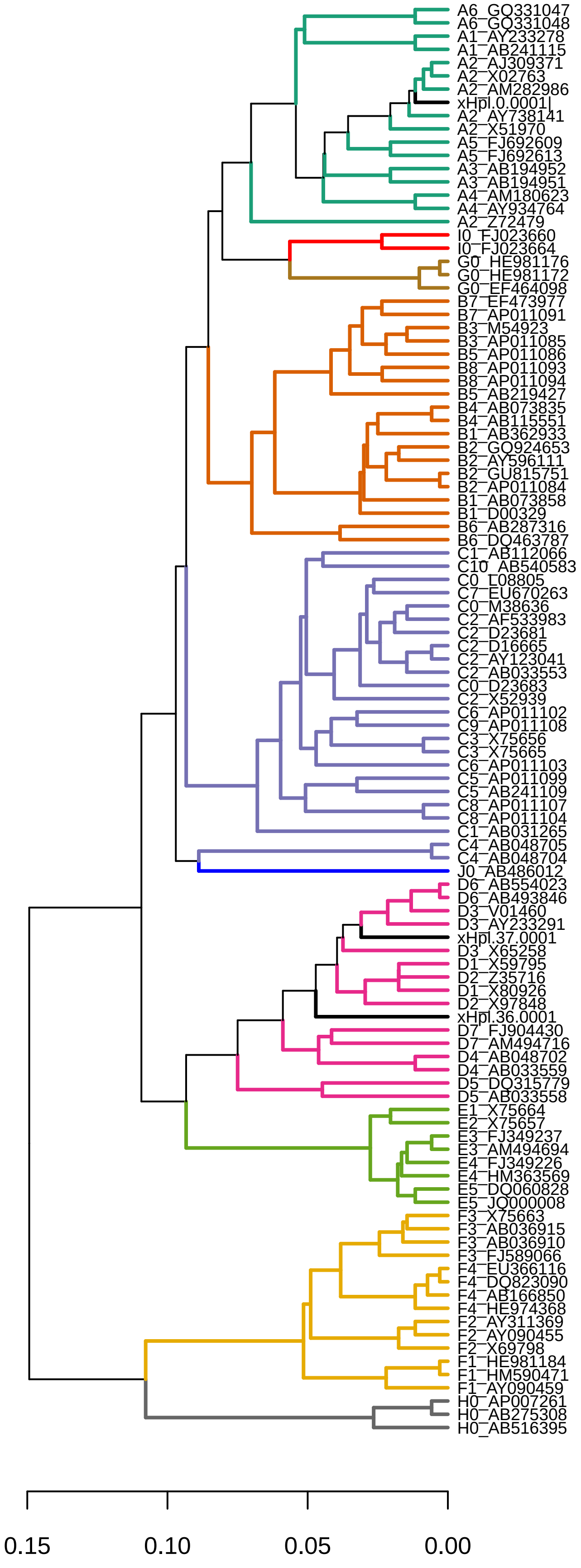

MDS map (K80): Pt03 First sample P/S region

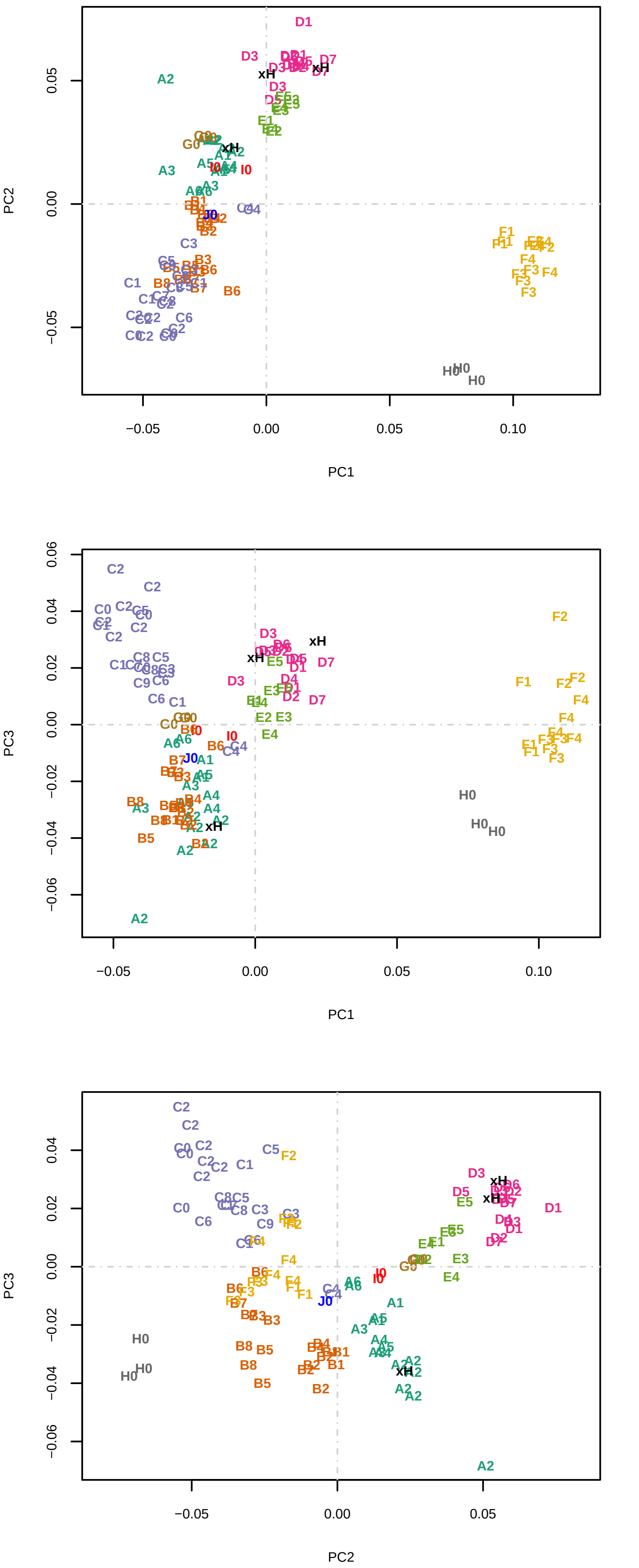

UPGMA tree (K80): Pt03 Second sample P/S region

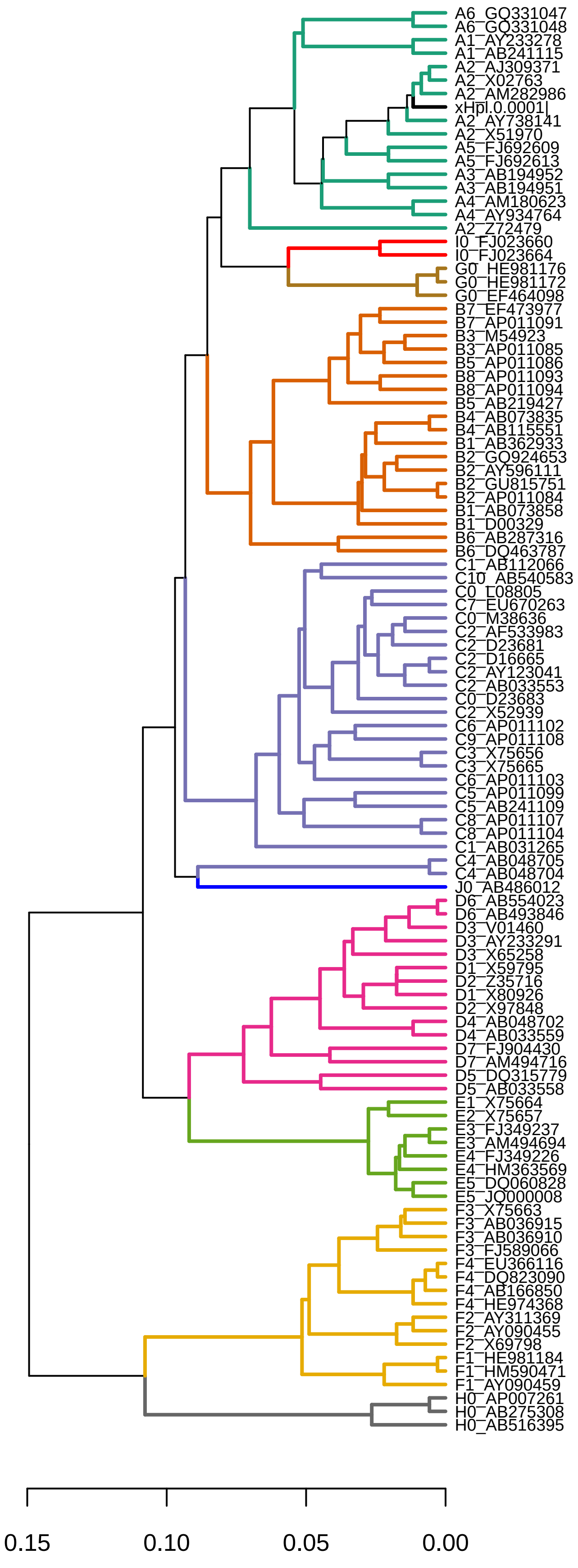

MDS map (K80): Pt03 Second sample P/S region

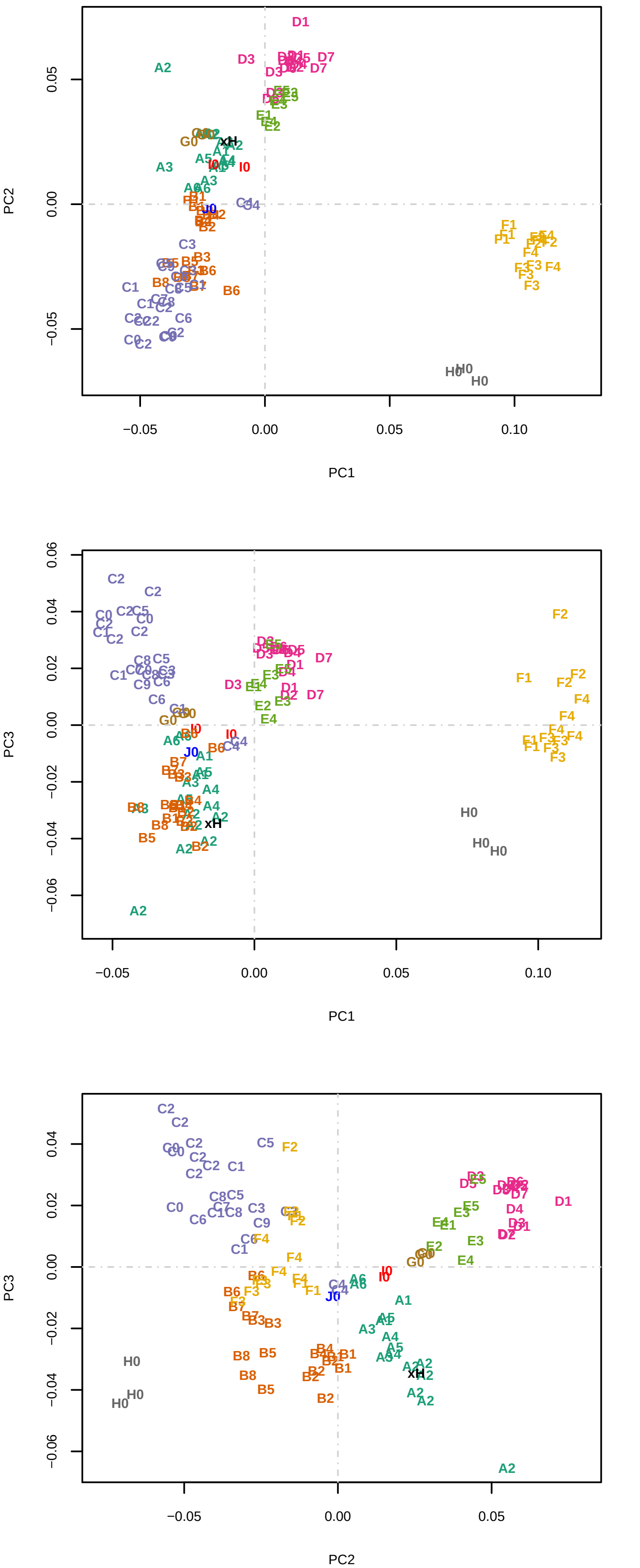

UPGMA tree (K80): Pt03 Third sample P/S region

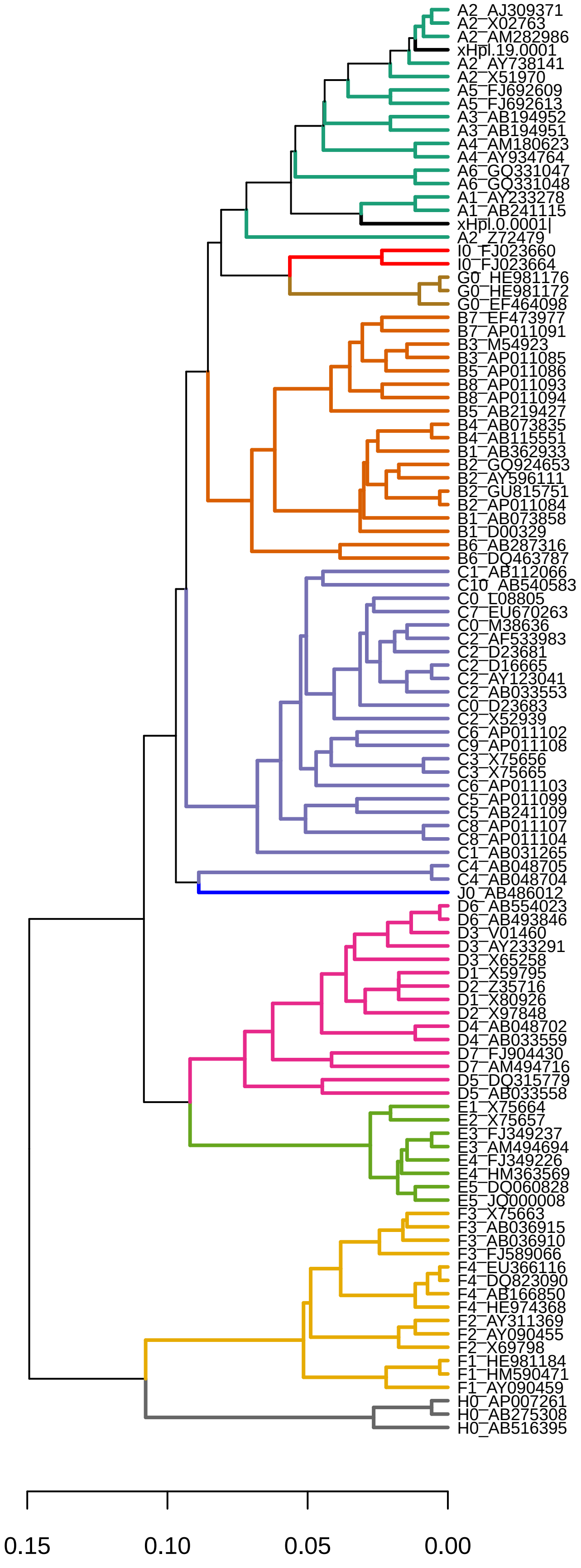

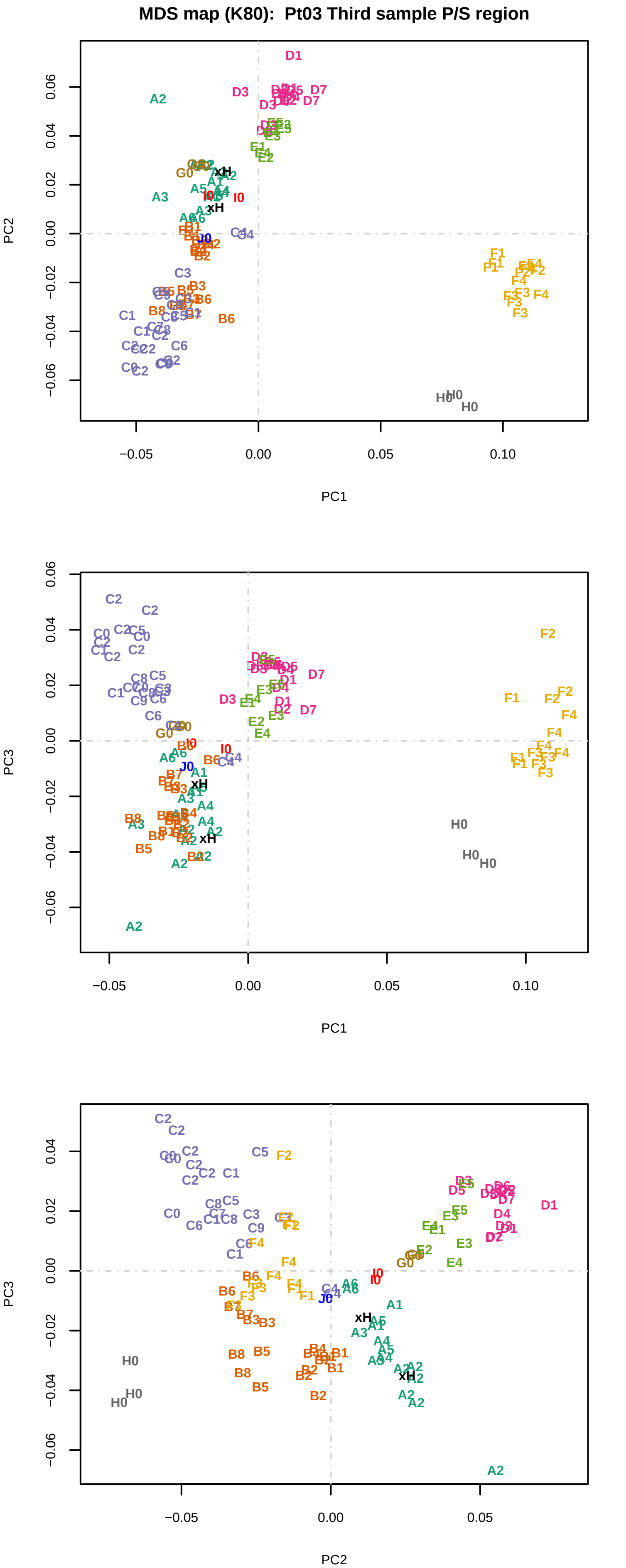
MDS map (K80): Pt03 Third sample P/S region
PC1
PC2
PC1
PC3
PC2
PC3

UPGMA tree (K80): Pt04 First sample P/S region

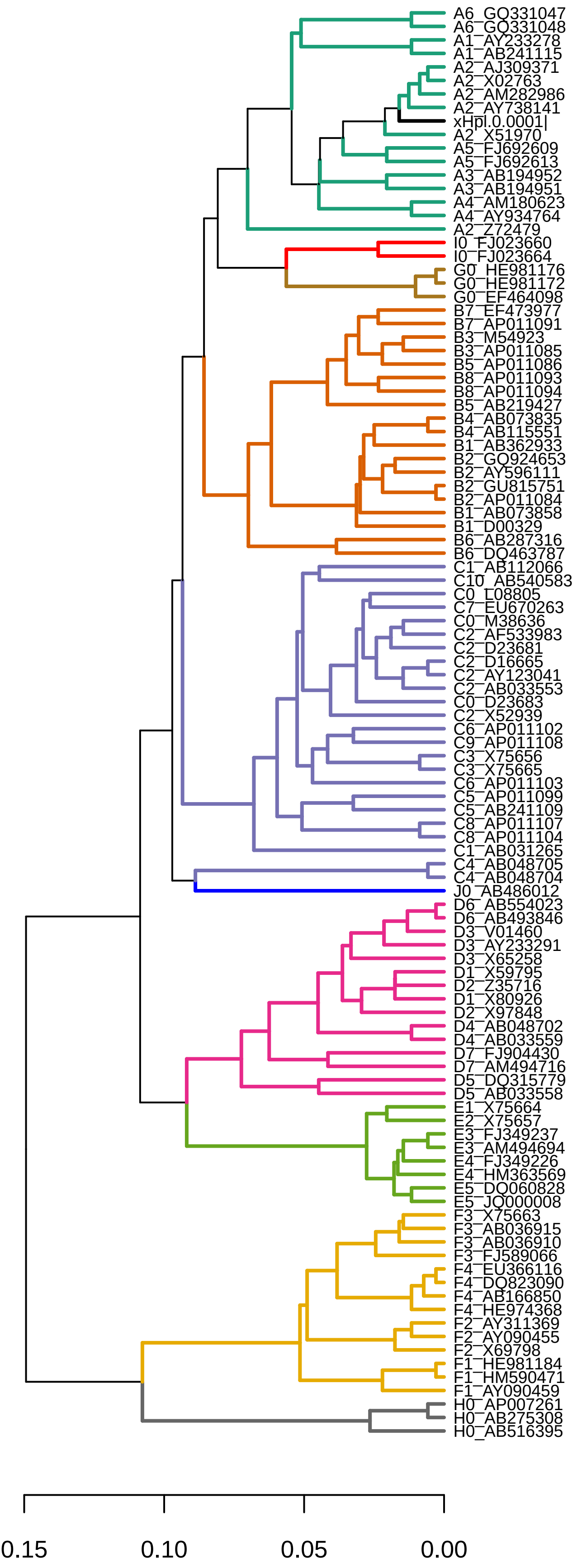

MDS map (K80): Pt04 First sample P/S region

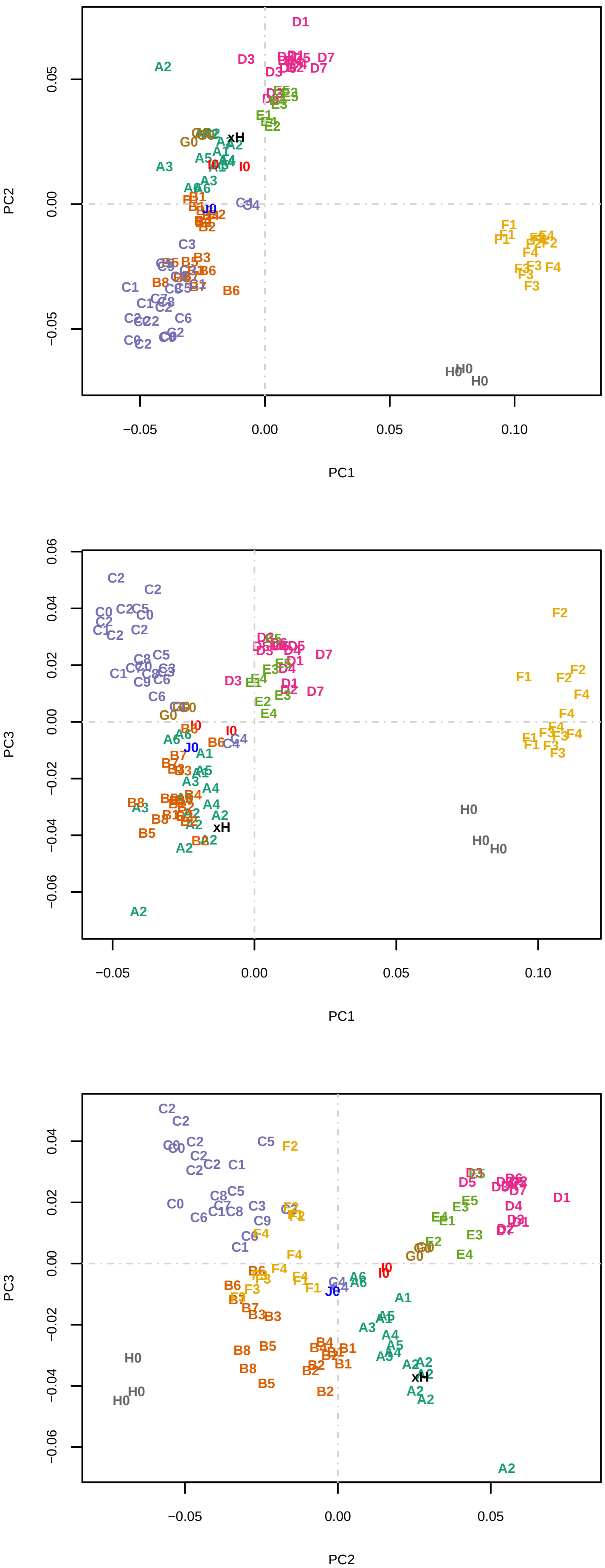

UPGMA tree (K80): Pt04 Second sample P/S region

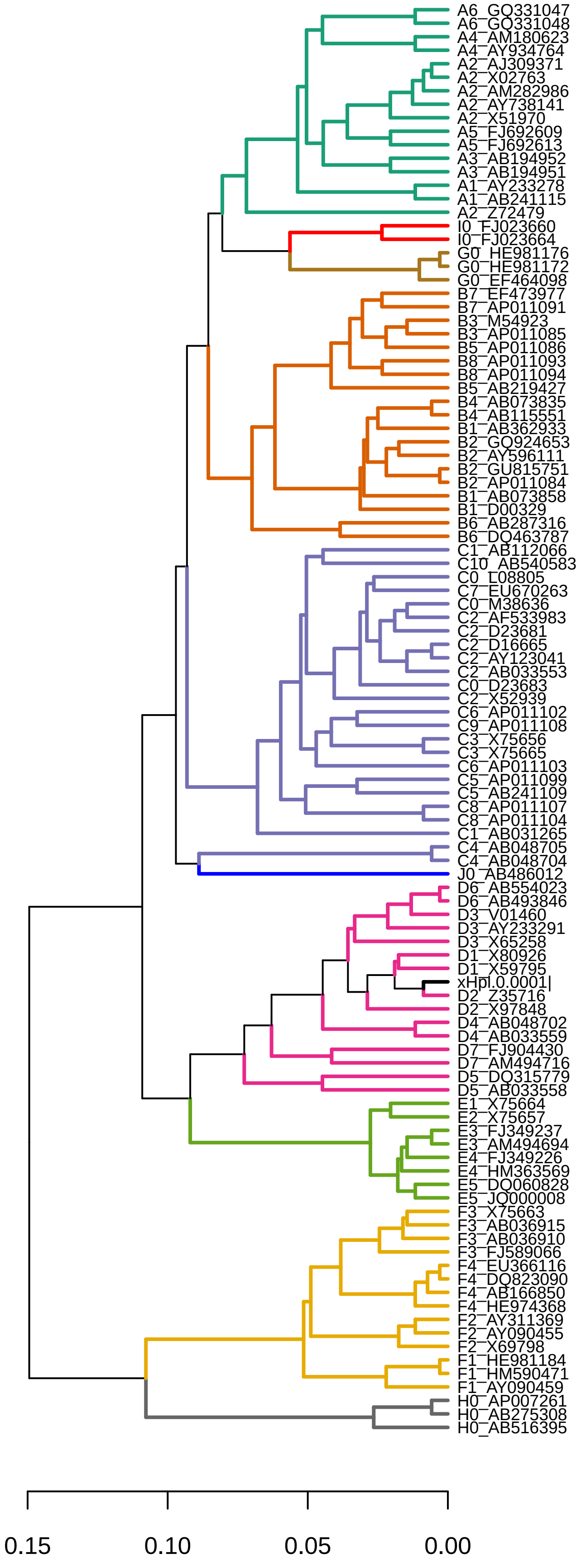

MDS map (K80): Pt04 Second sample P/S region

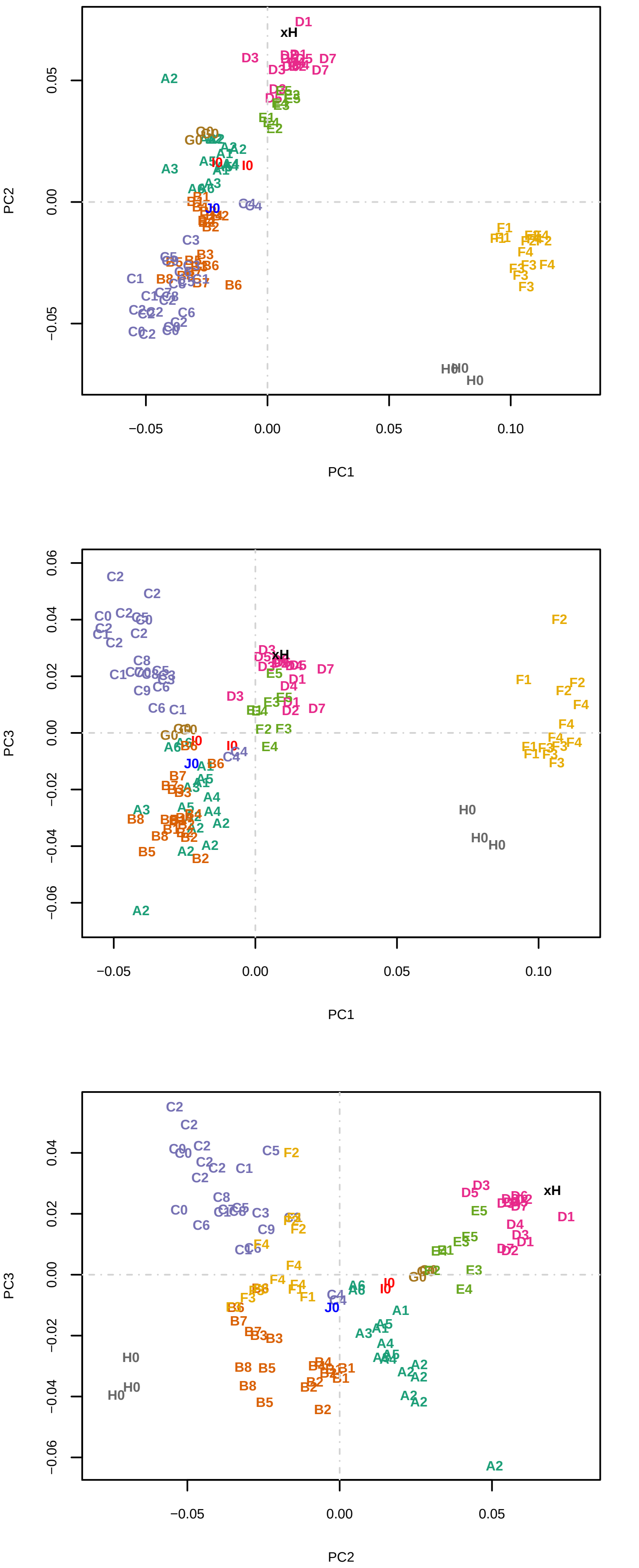

UPGMA tree (K80): Pt04 Third sample P/S region

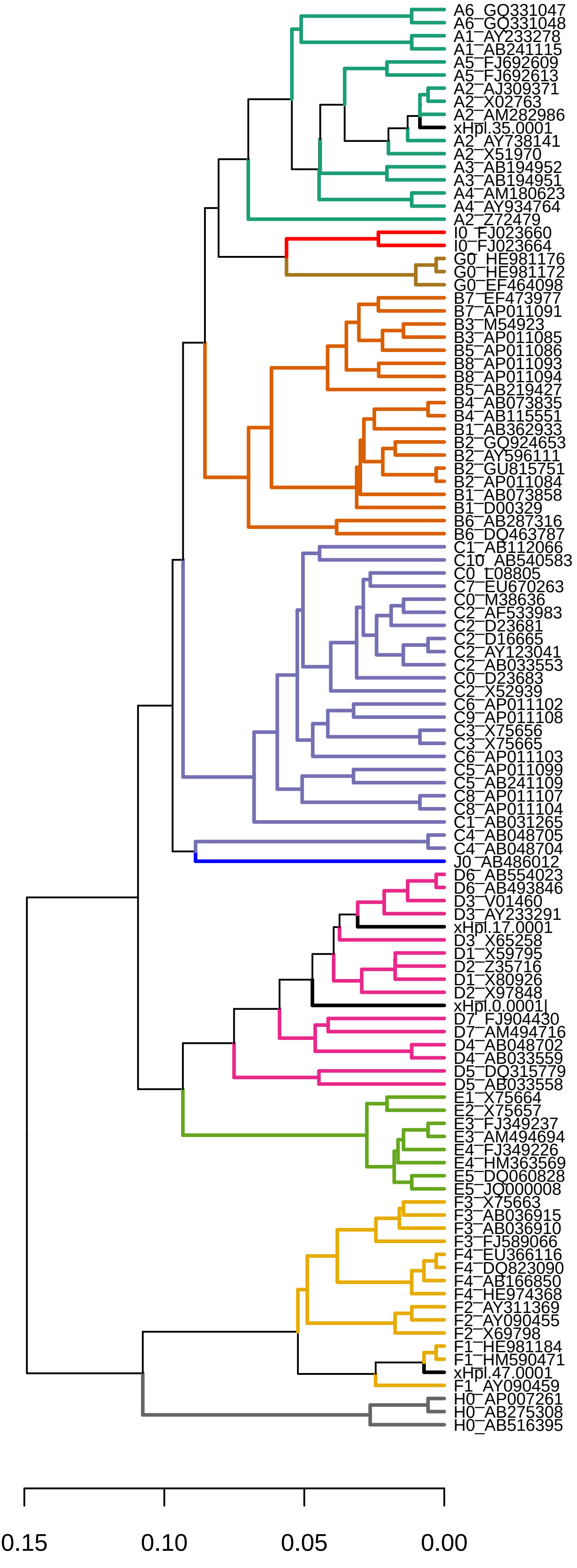

MDS map (K80): Pt04 Third sample P/S region

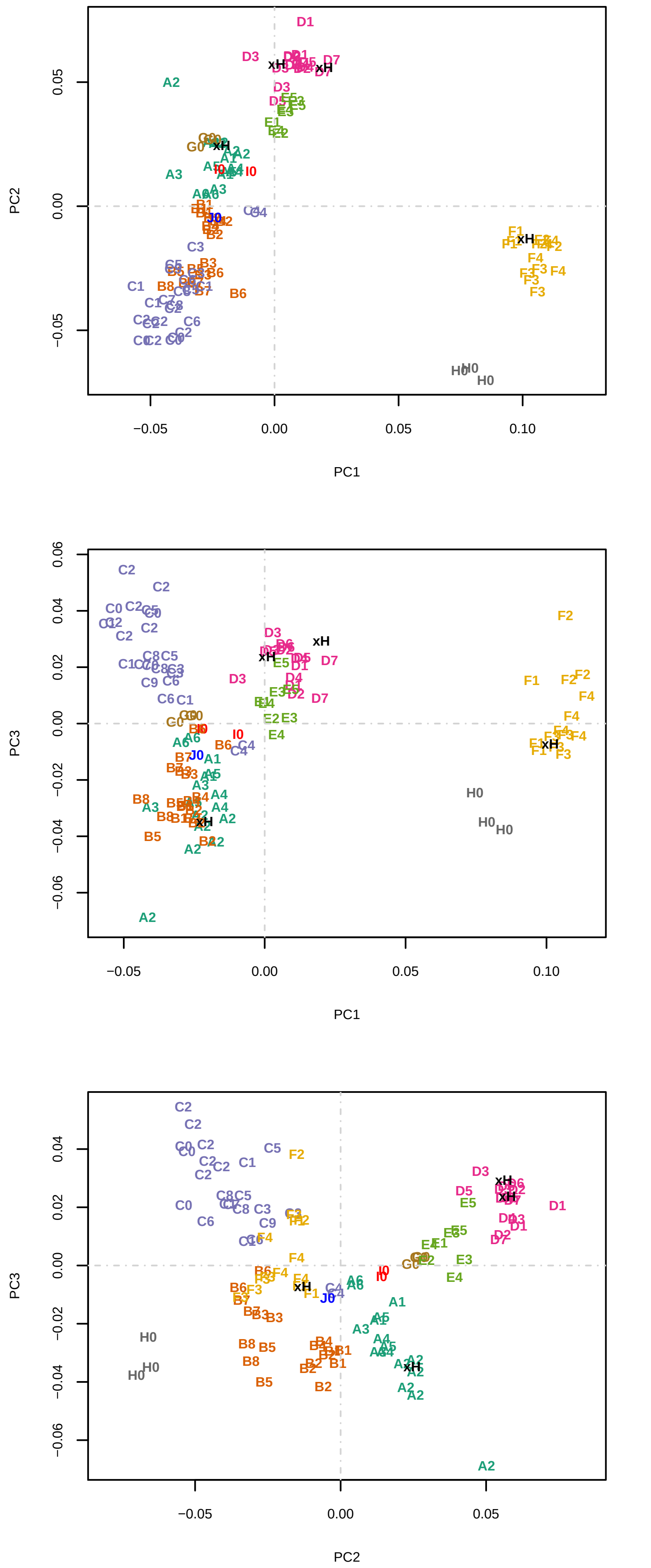

UPGMA tree (K80): Pt05 First sample P/S region

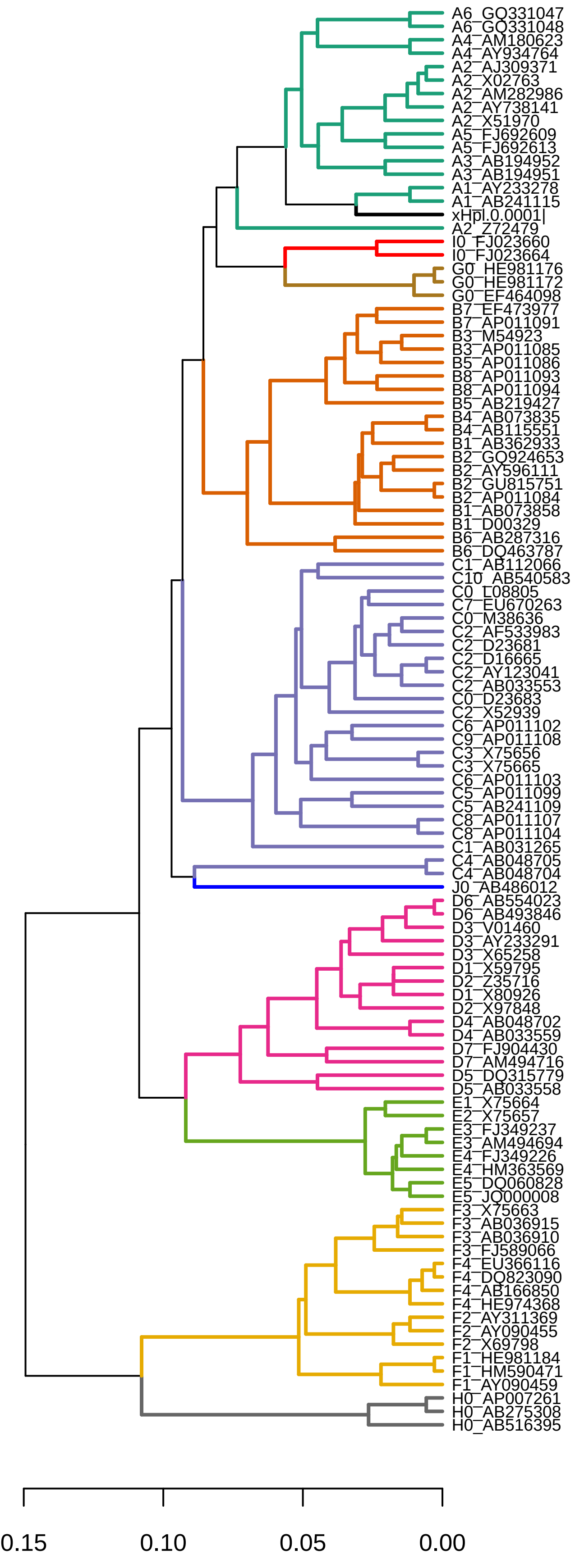

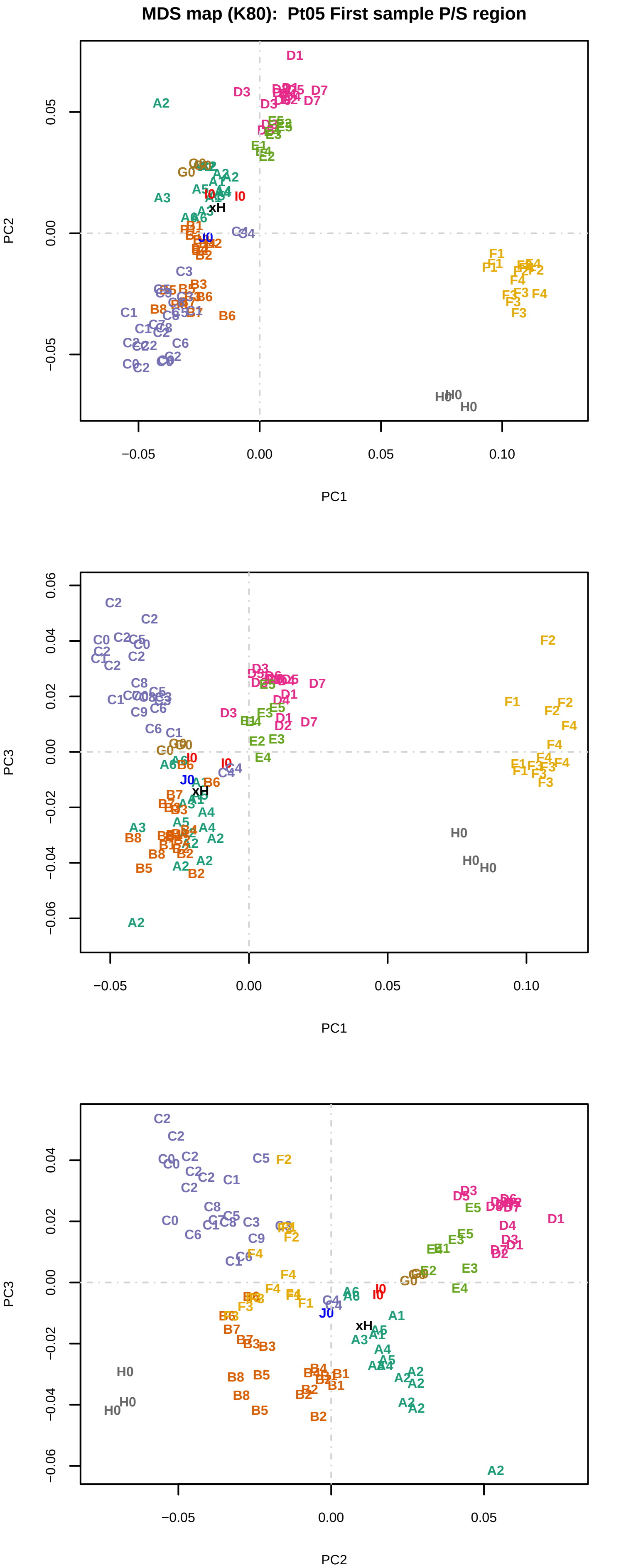
MDS map (K80): Pt05 First sample P/S region
PC1
PC2
PC3

UPGMA tree (K80): Pt05 Second sample P/S region

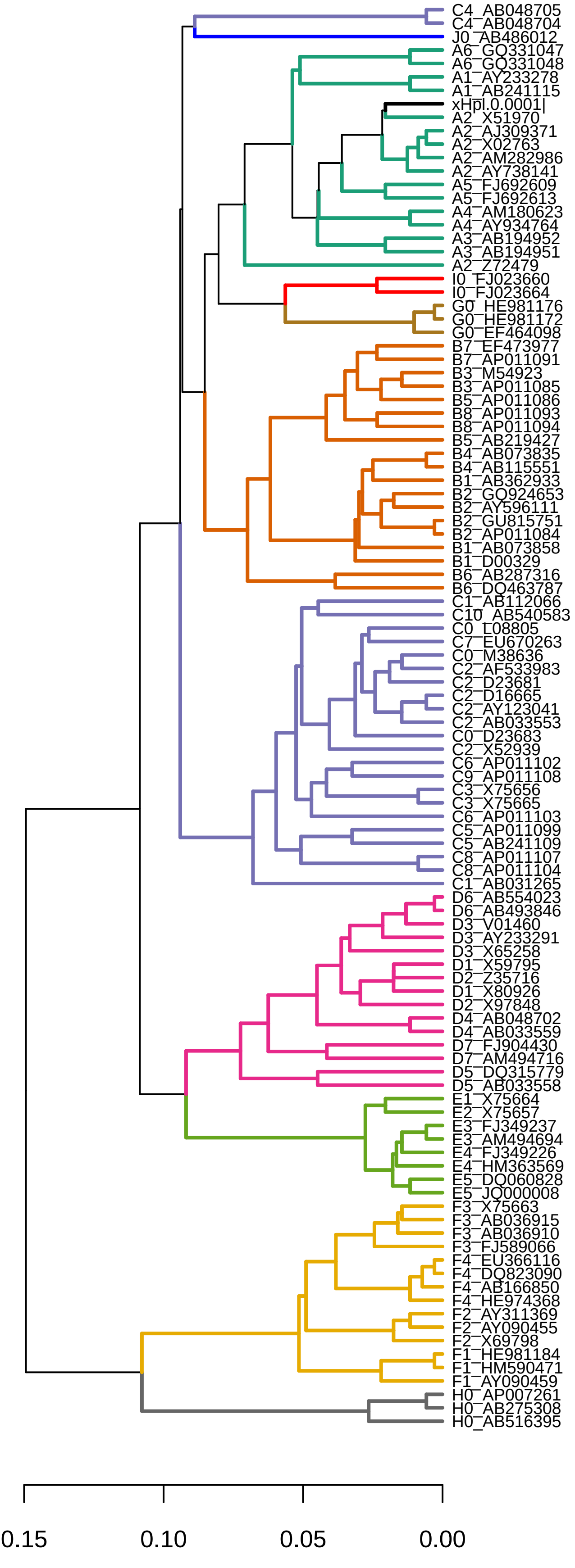

MDS map (K80): Pt05 Second sample P/S region

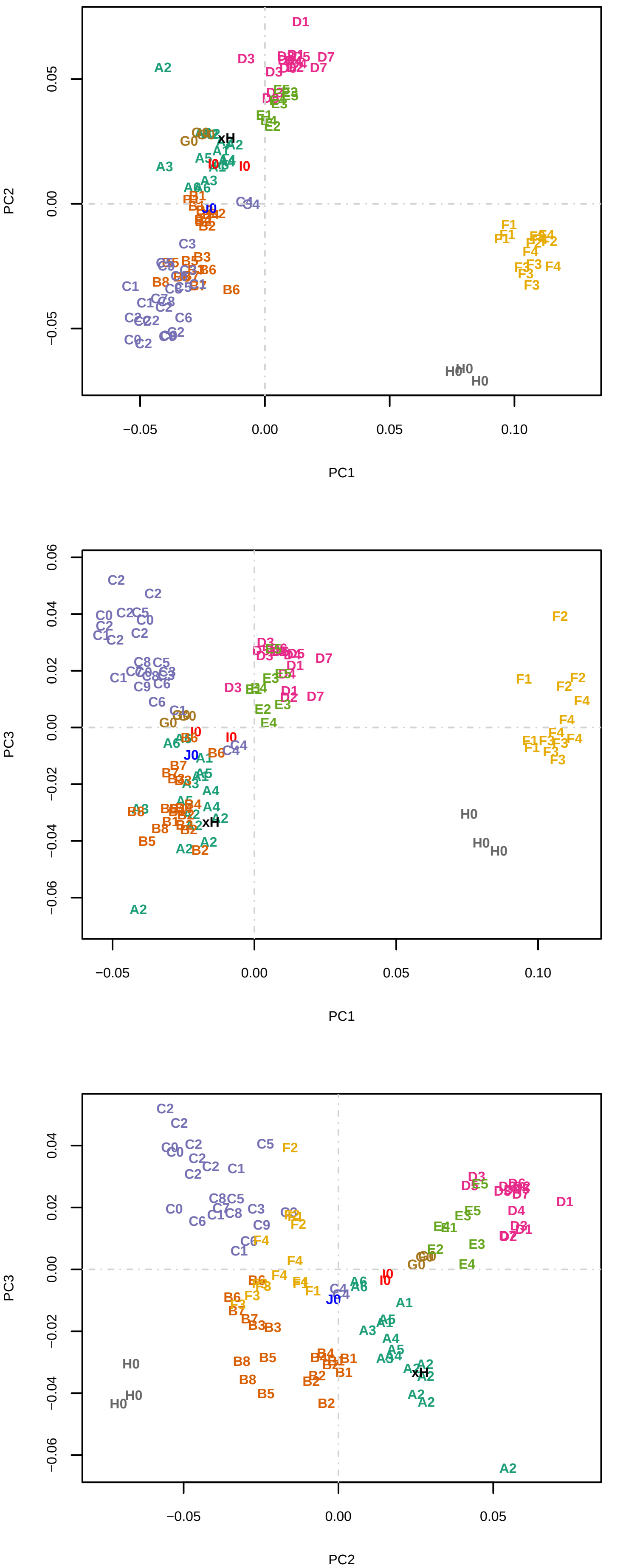

UPGMA tree (K80): Pt05 Third sample P/S region

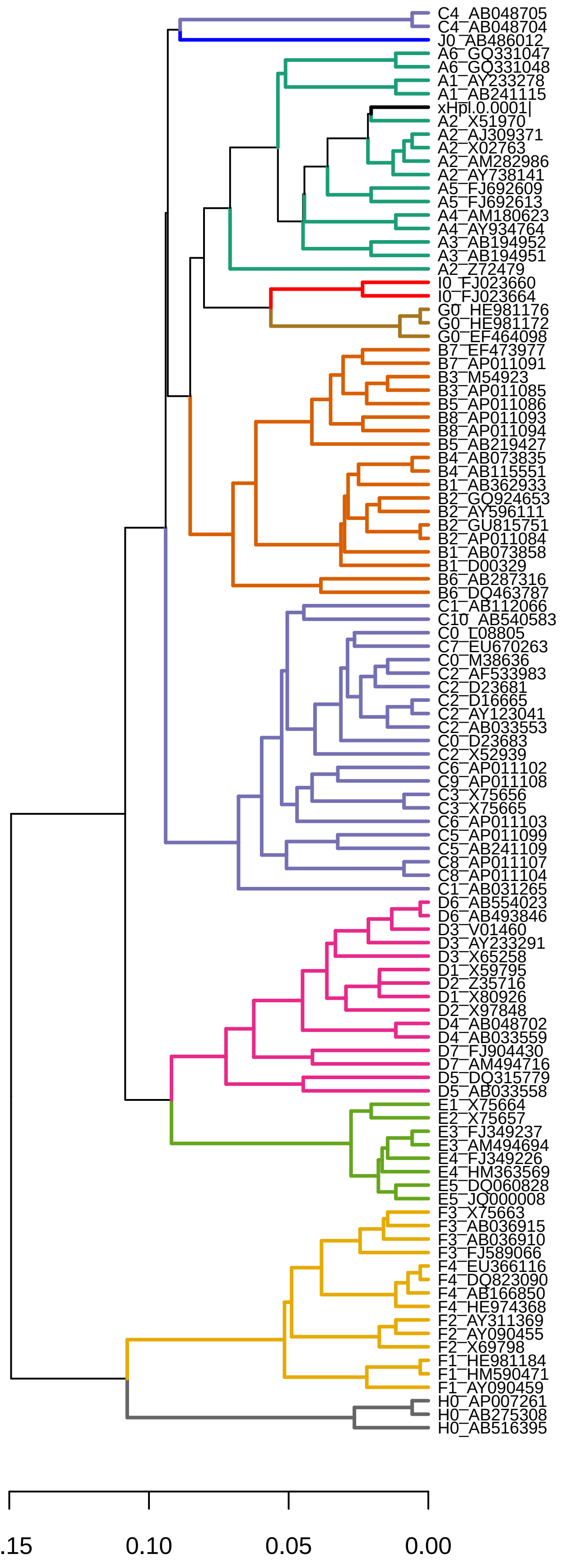

MDS map (K80): Pt05 Third sample P/S region

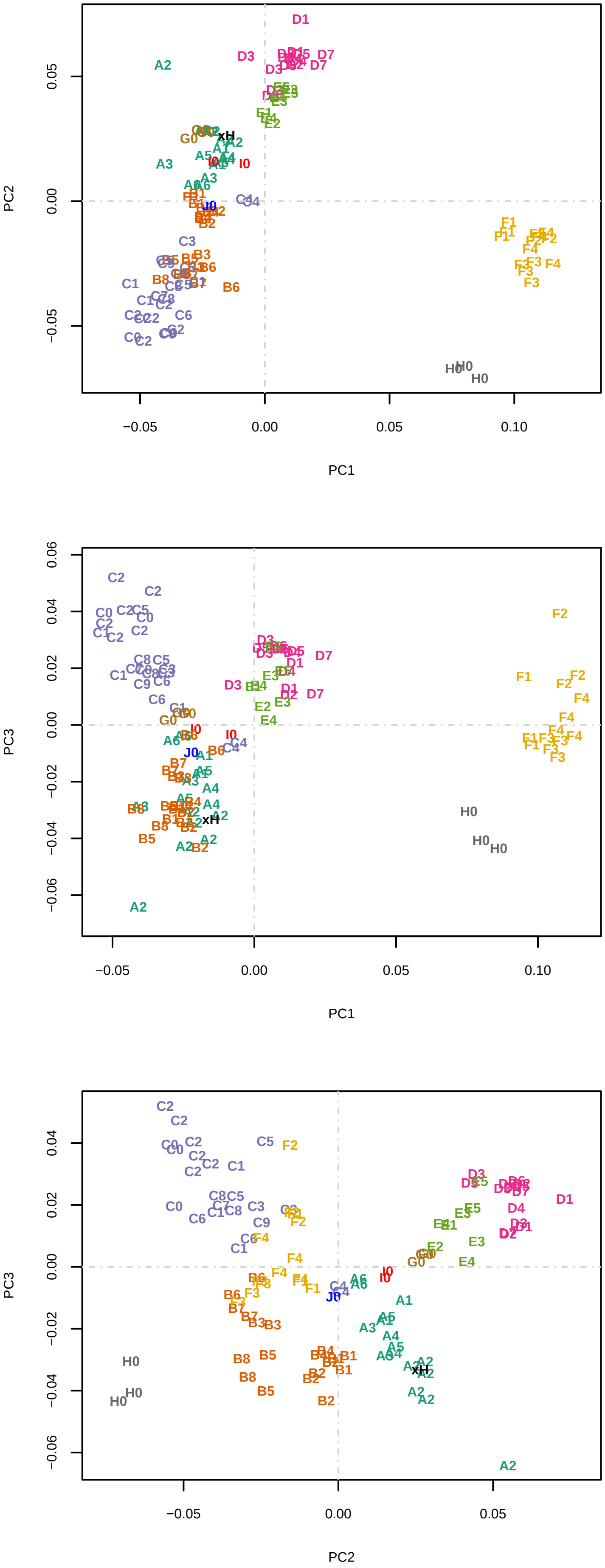

UPGMA tree (K80): Pt06 First sample P/S region

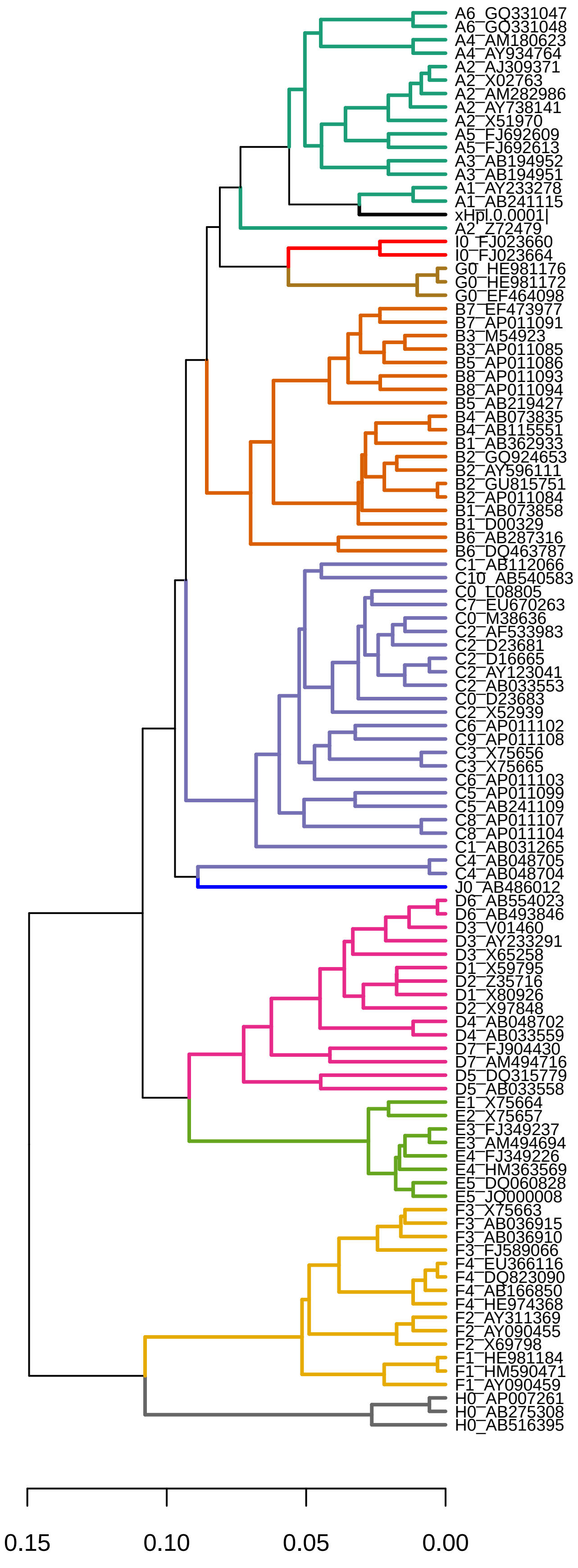

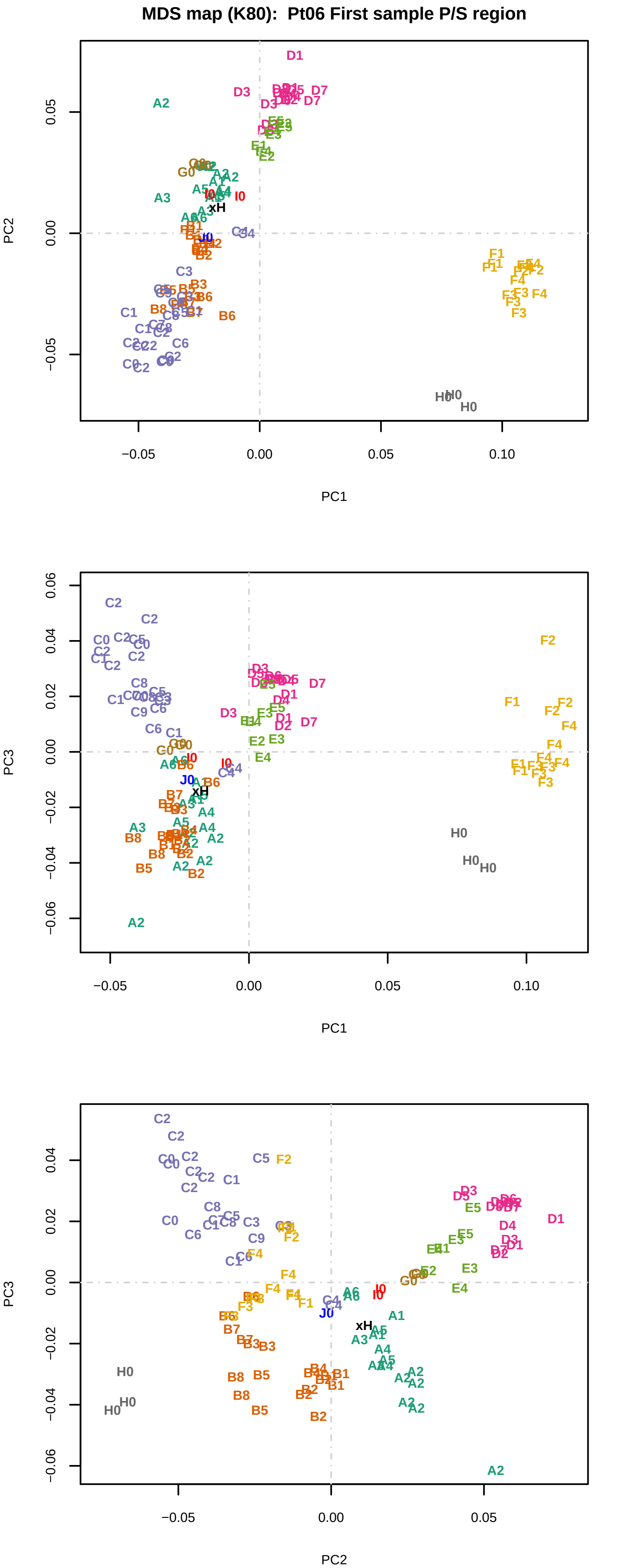
MDS map (K80): Pt06 First sample P/S region
PC1
PC2
PC3
−0.05
0.00
0.05
0.10
0.06
0.04
0.02
0.00
−0.02
−0.04
−0.06

UPGMA tree (K80): Pt06 Second sample P/S region

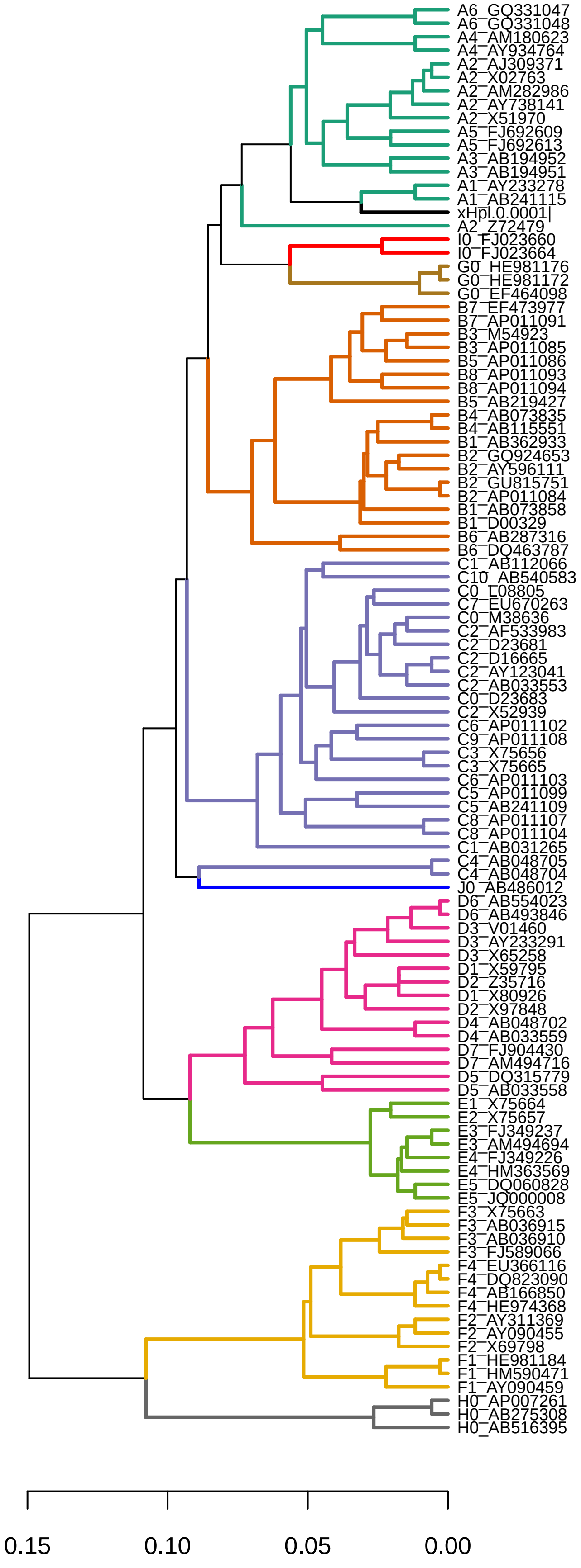

MDS map (K80): Pt06 Second sample P/S region

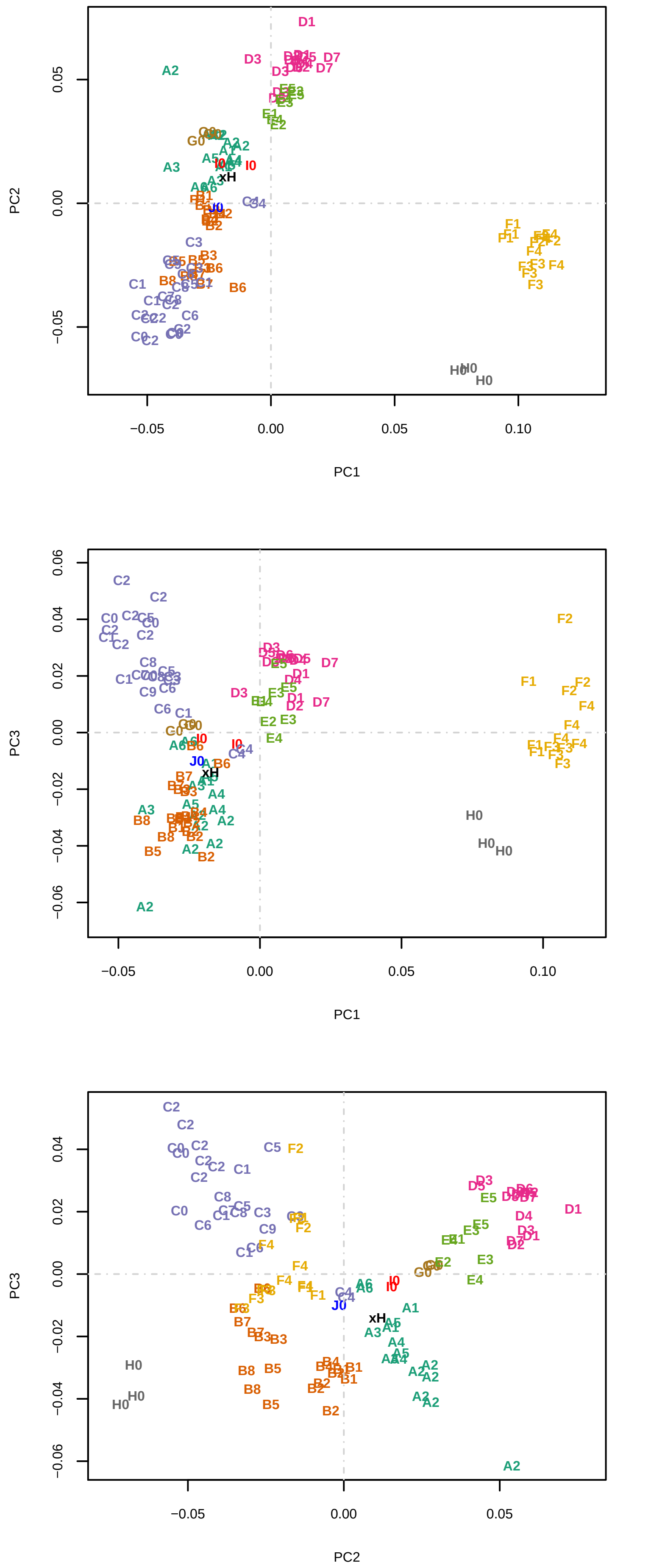

UPGMA tree (K80): Pt06 Third sample P/S region

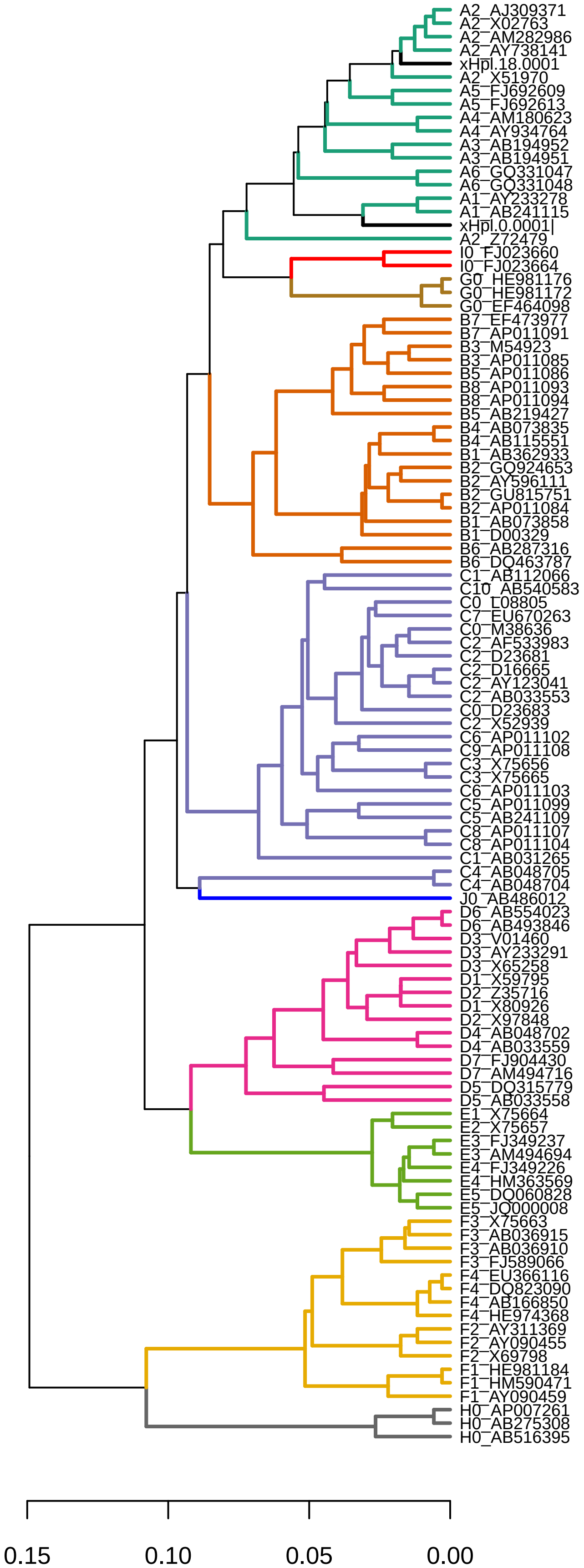

MDS map (K80): Pt06 Third sample P/S region

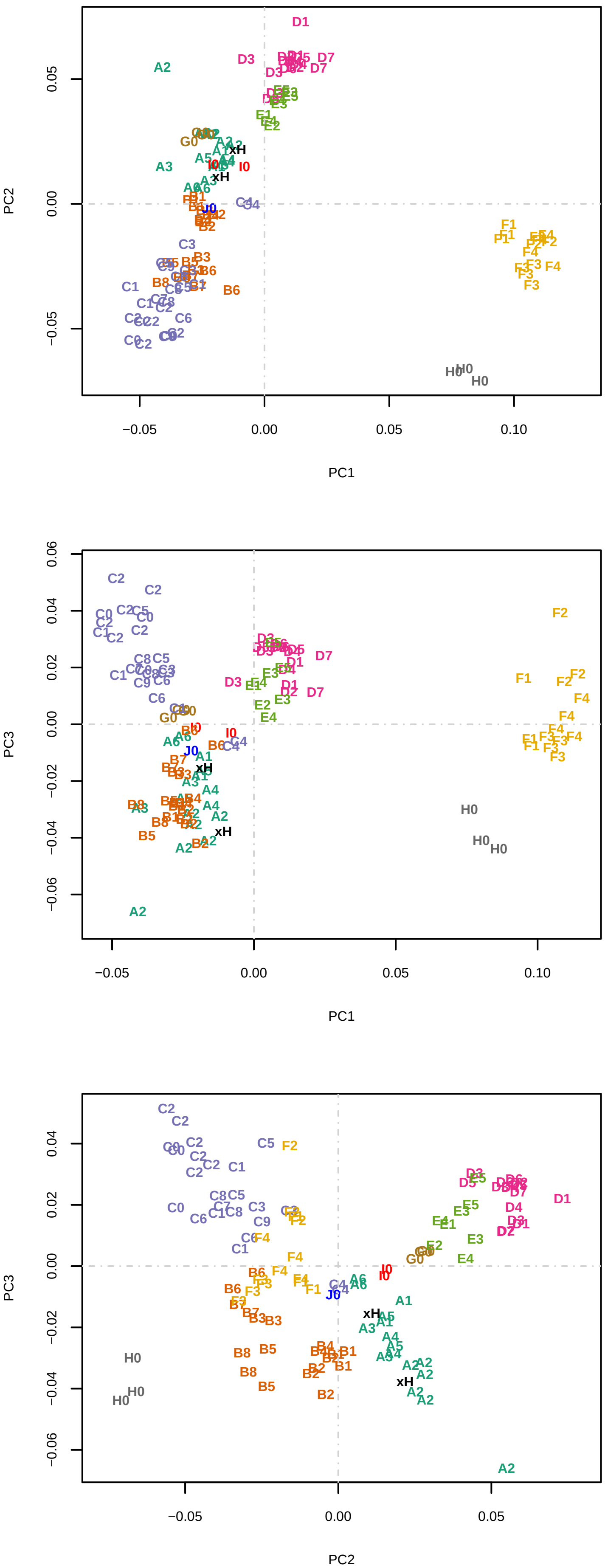

UPGMA tree (K80): Pt07 First sample P/S region

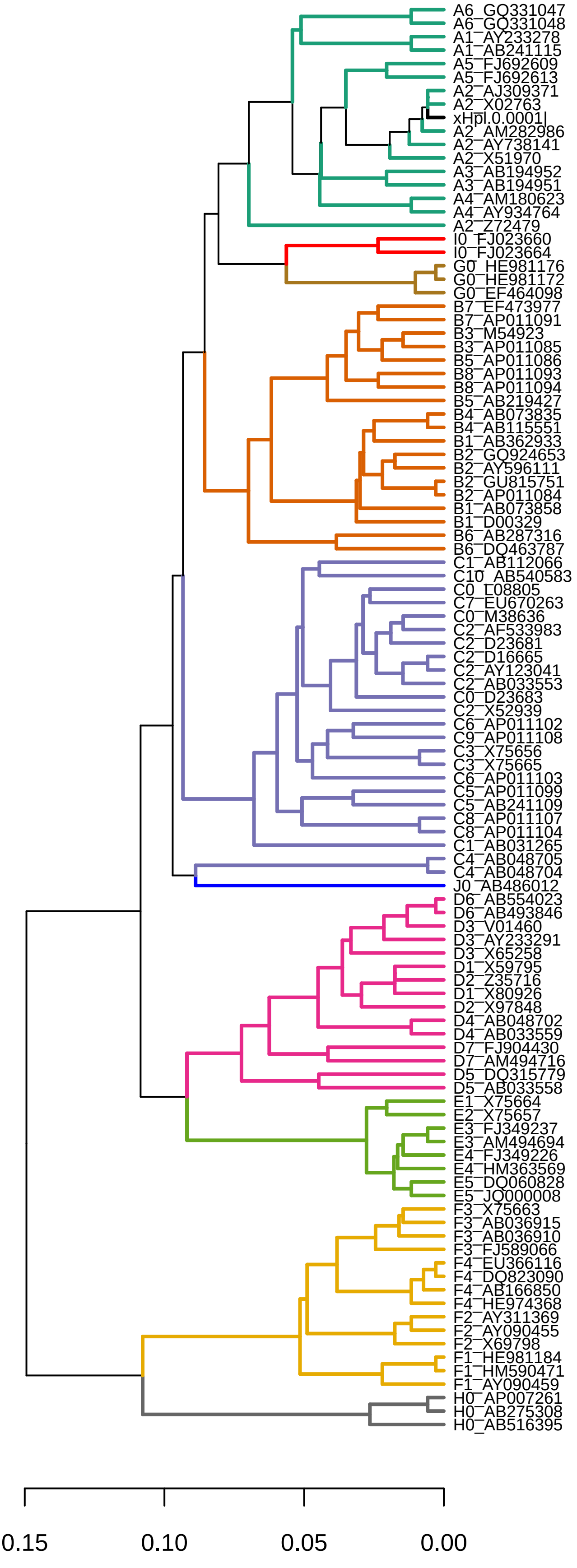

MDS map (K80): Pt07 First sample P/S region

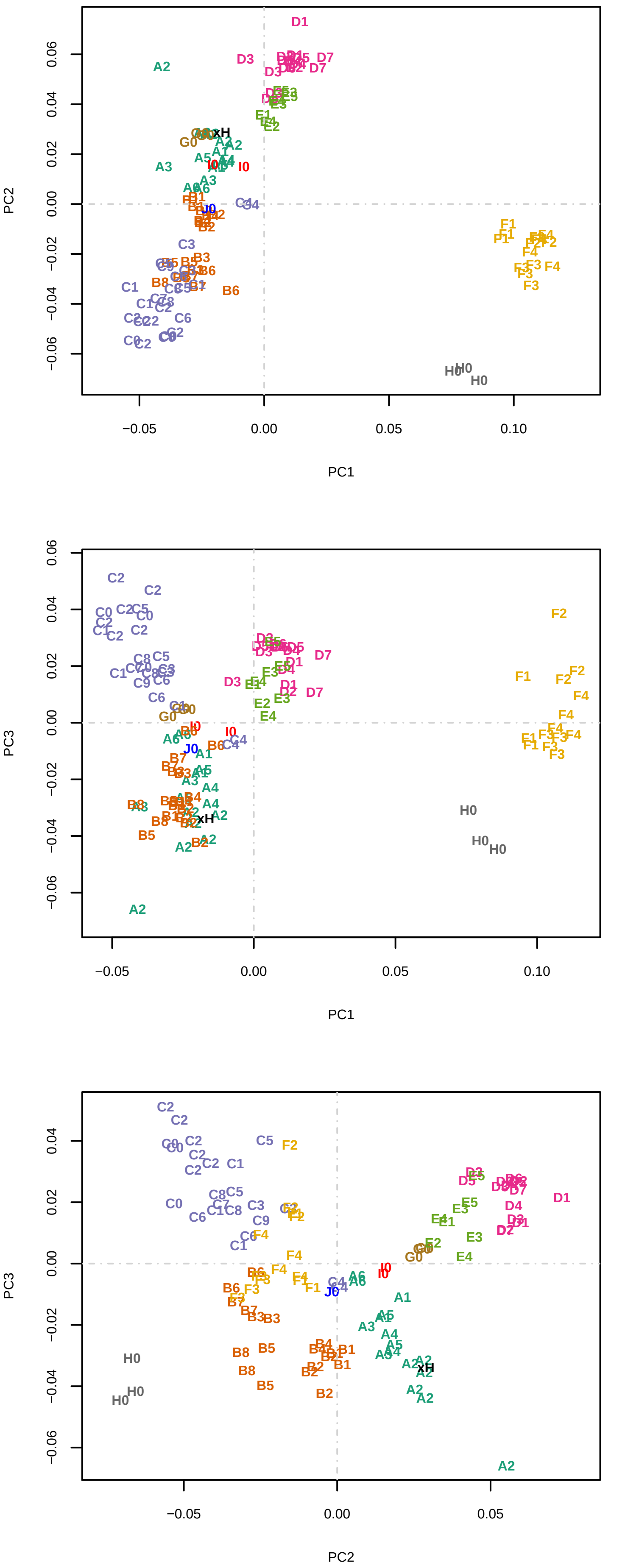

UPGMA tree (K80): Pt07 Second sample P/S region

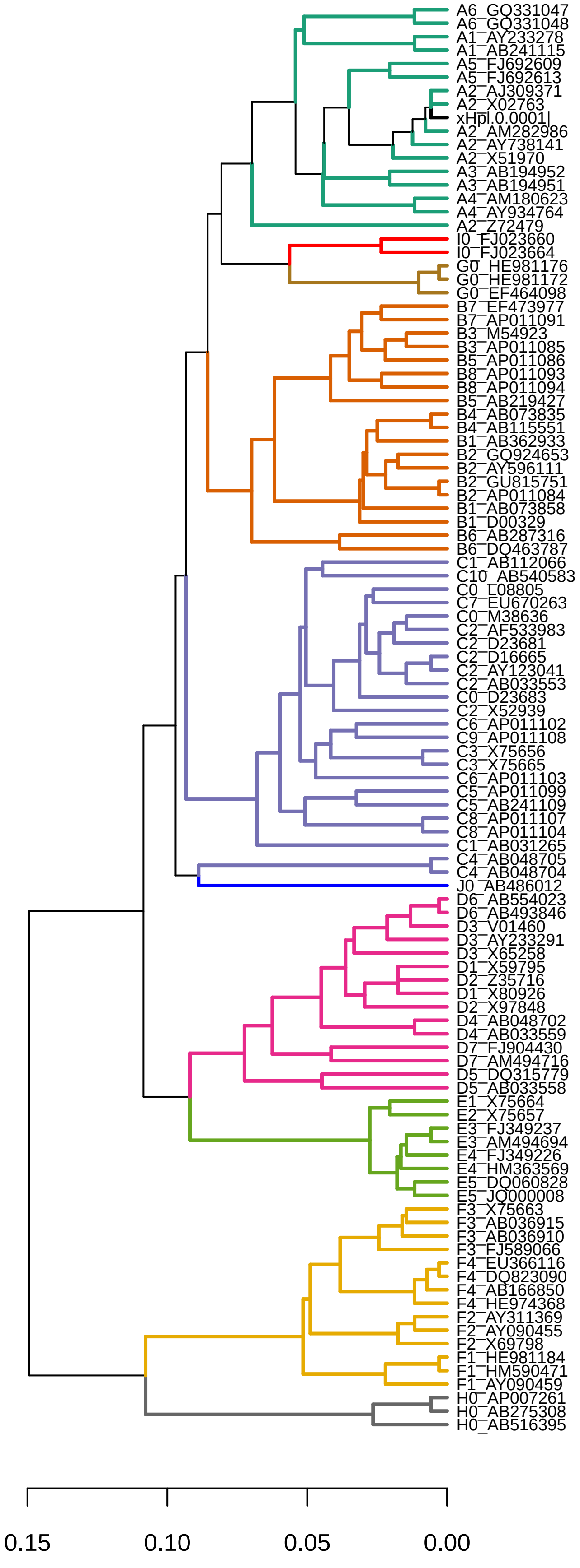

MDS map (K80): Pt07 Second sample P/S region

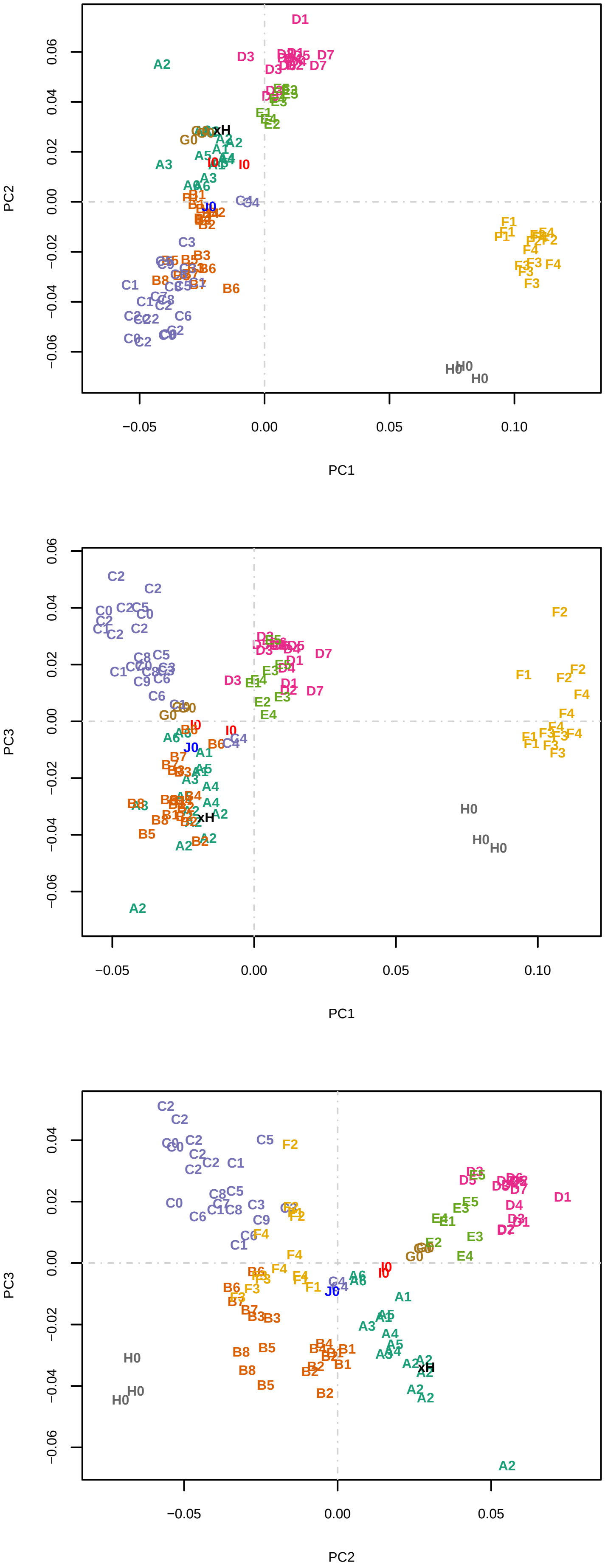

UPGMA tree (K80): Pt07 Third sample P/S region

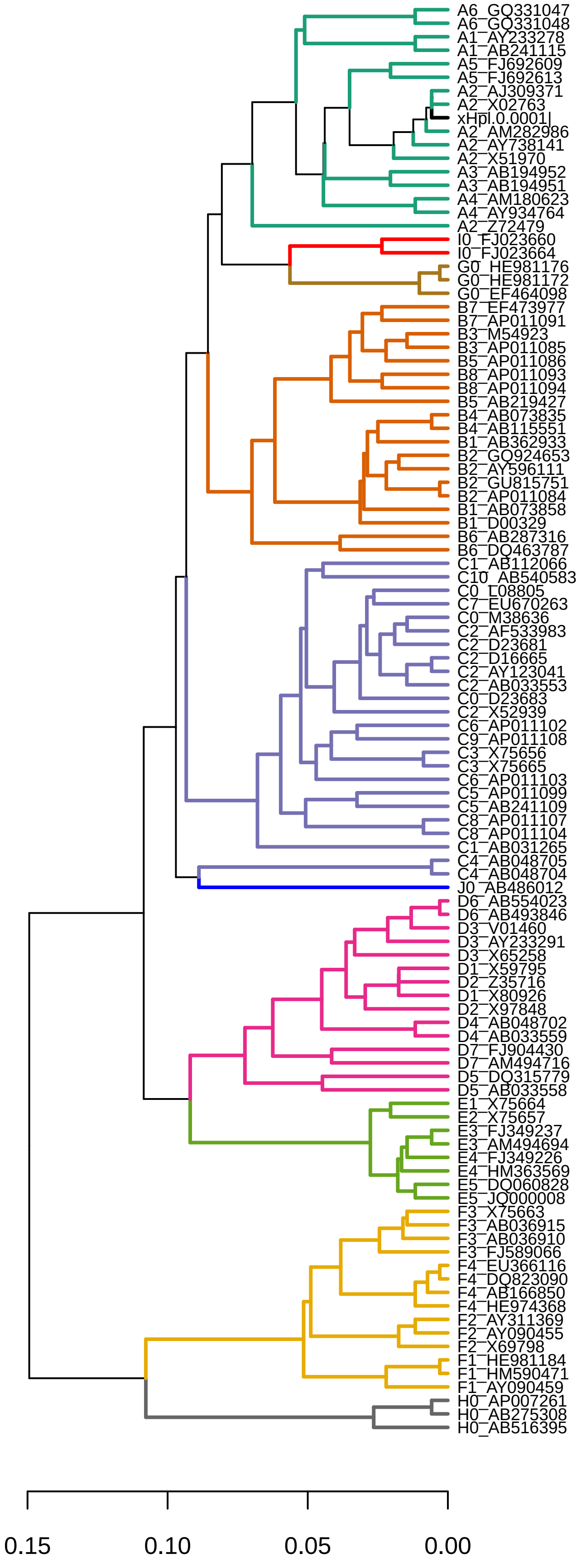

MDS map (K80): Pt07 Third sample P/S region

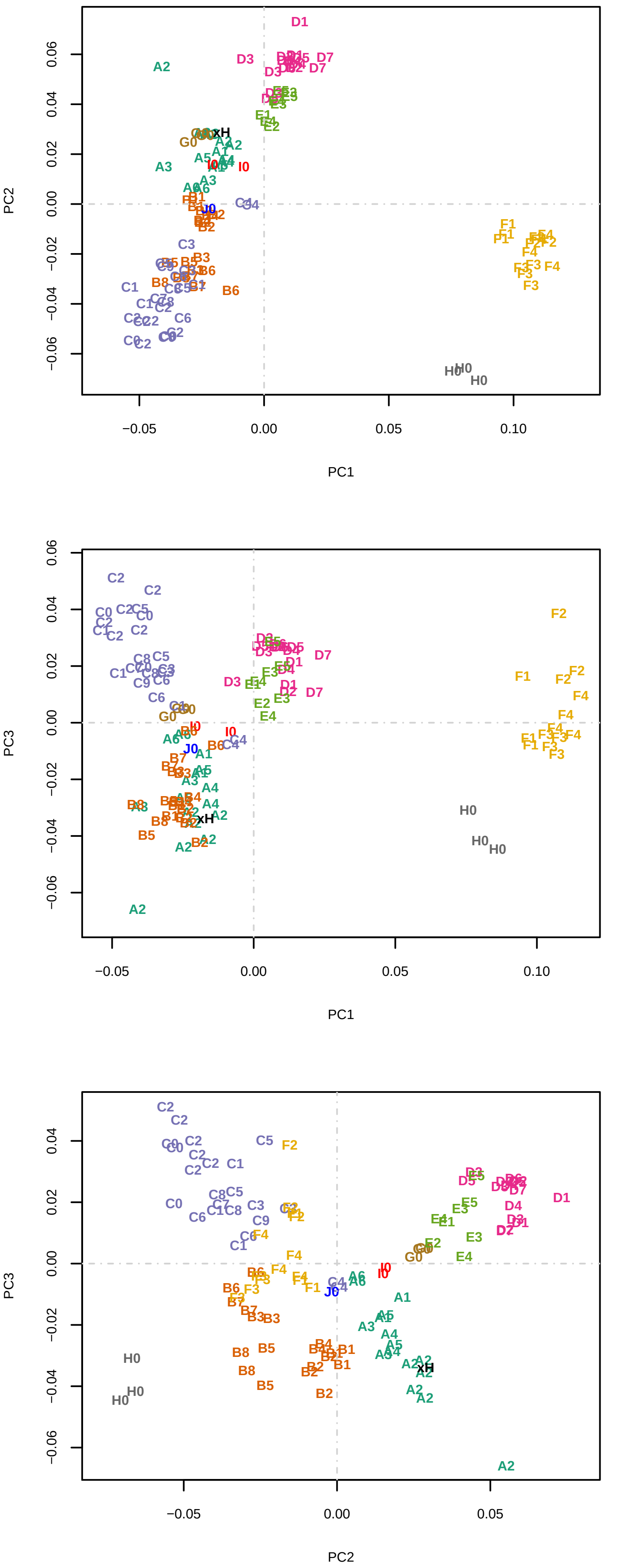

UPGMA tree (K80): Pt08 First sample P/S region

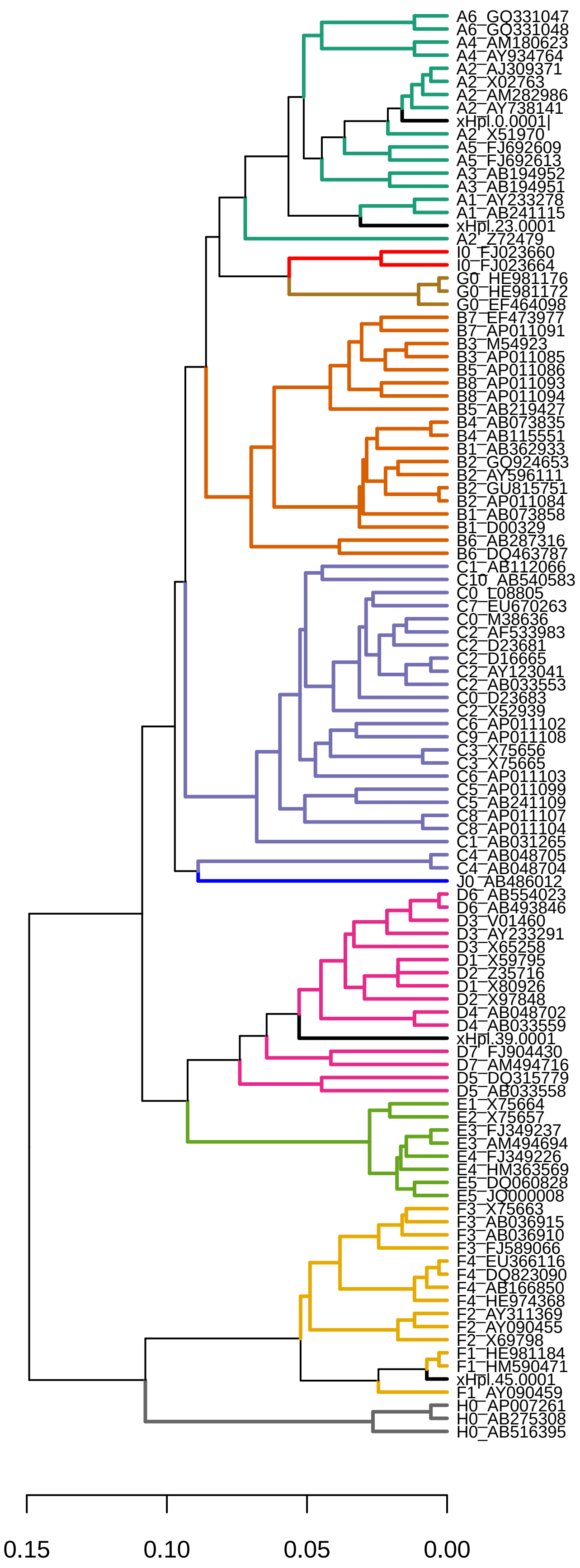

MDS map (K80): Pt08 First sample P/S region

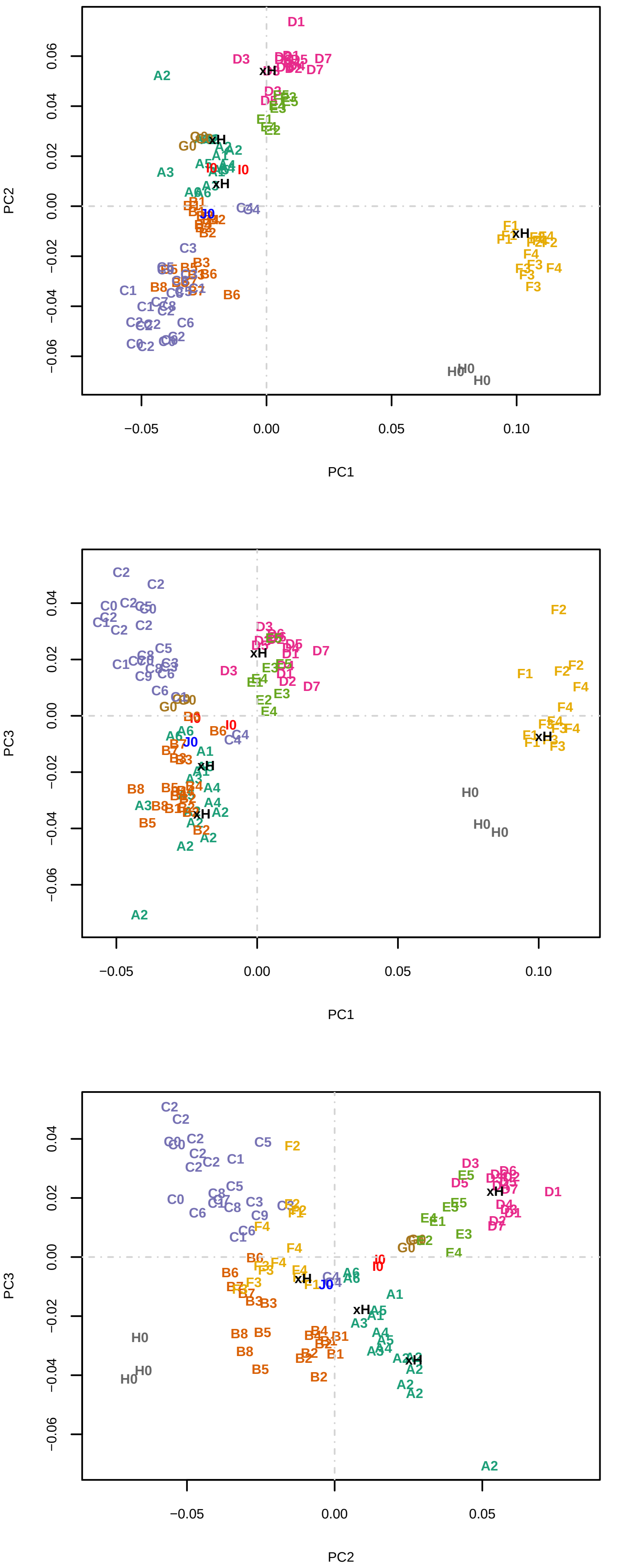

UPGMA tree (K80): Pt08 Second sample P/S region

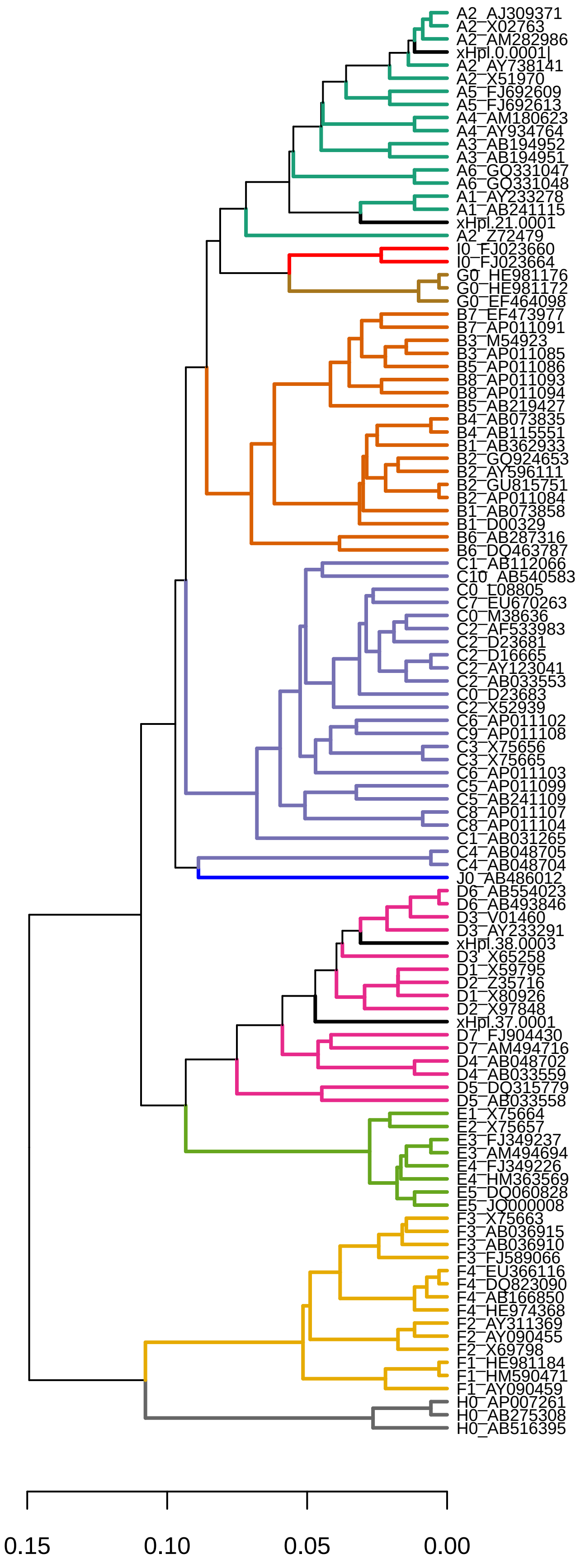

MDS map (K80): Pt08 Second sample P/S region

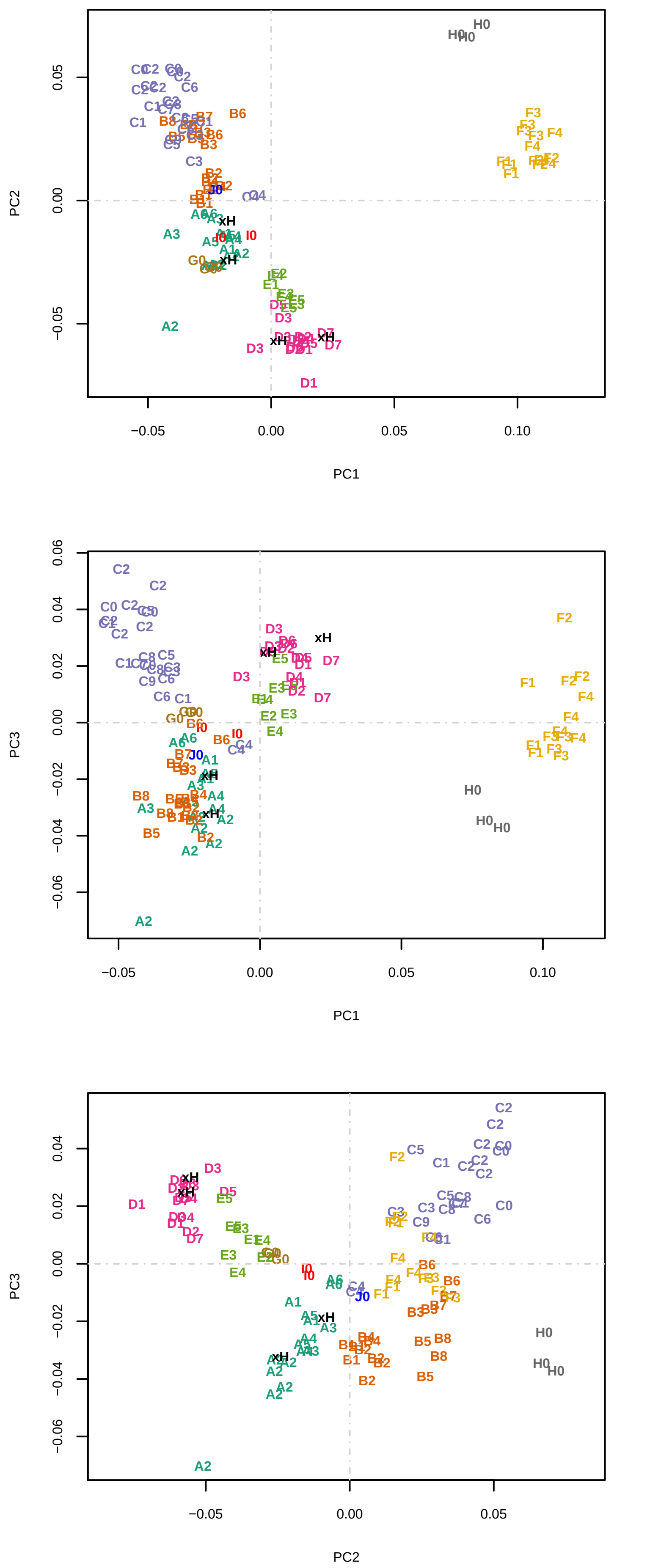

UPGMA tree (K80): Pt08 Third sample P/S region

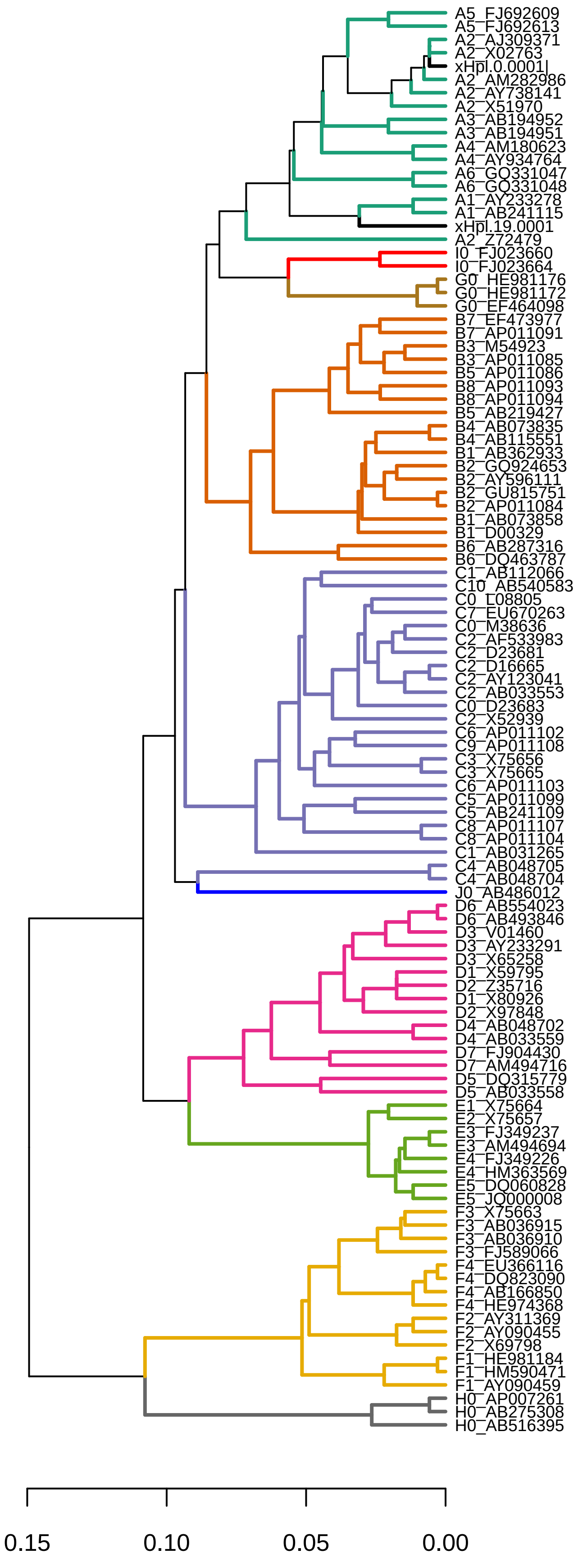

MDS map (K80): Pt08 Third sample P/S region

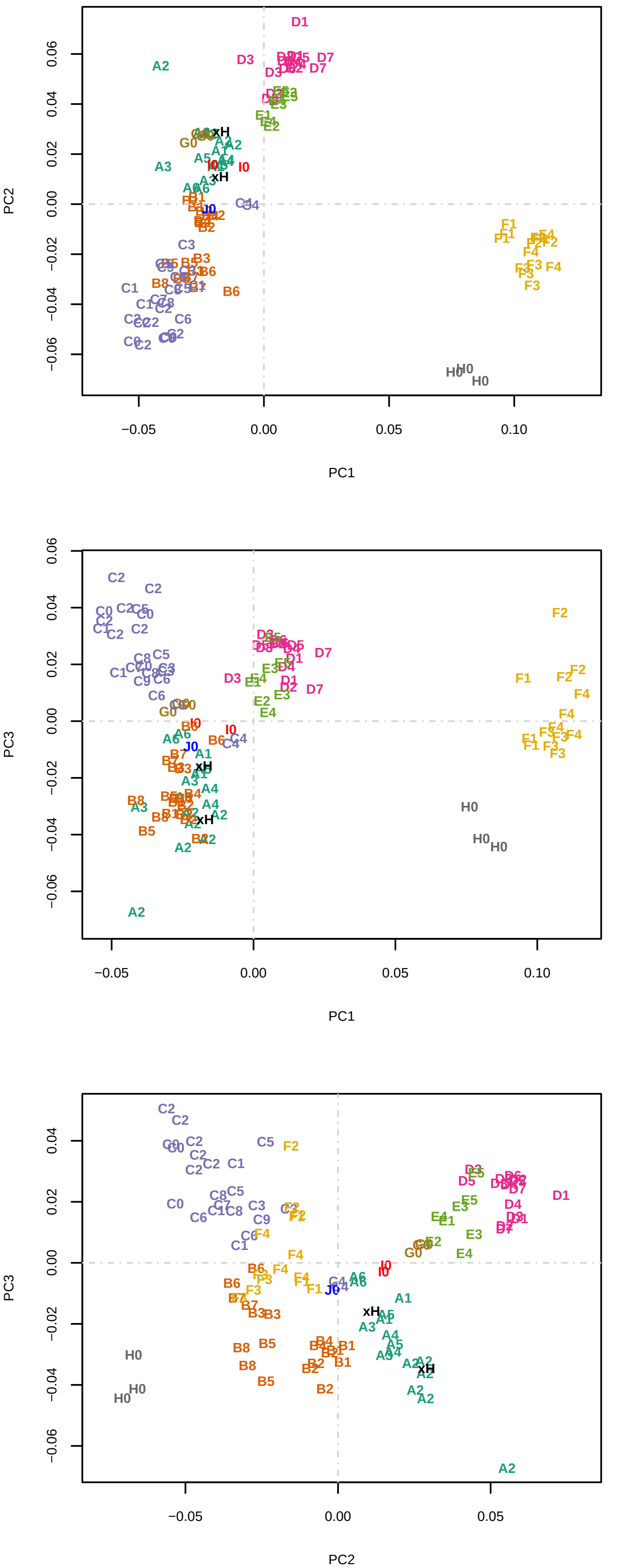

UPGMA tree (K80): Pt09 First sample P/S region

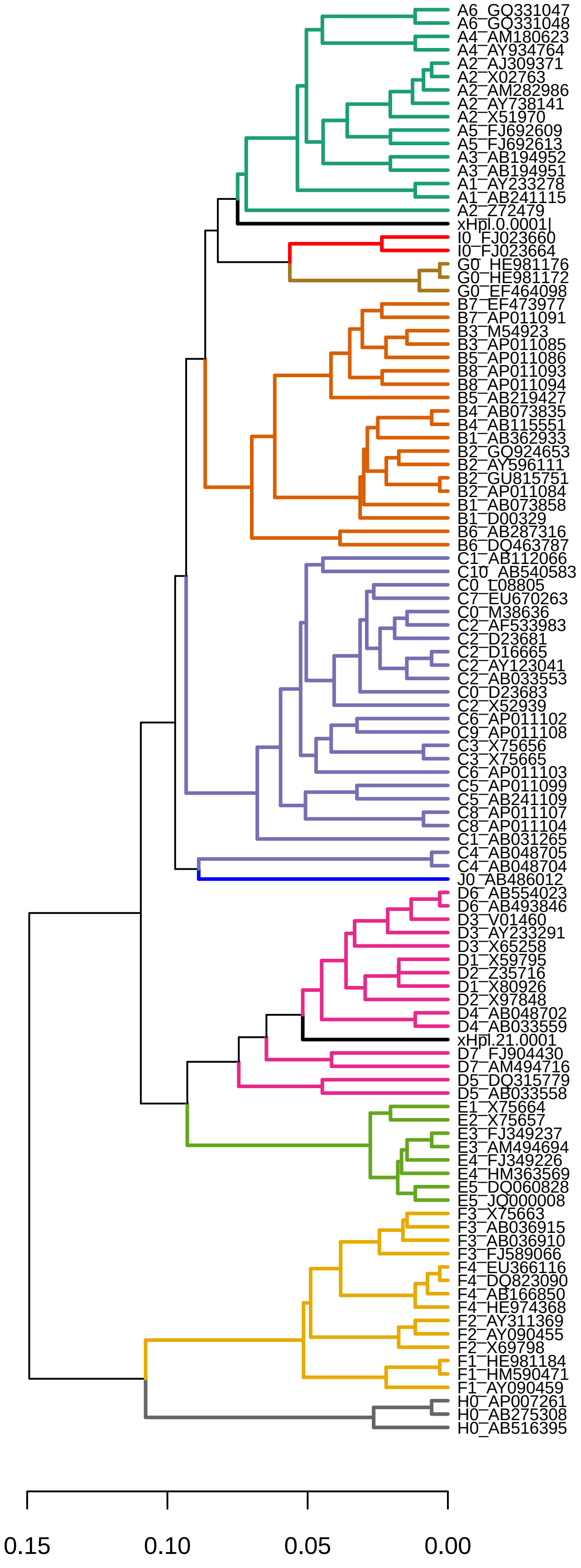

MDS map (K80): Pt09 First sample P/S region

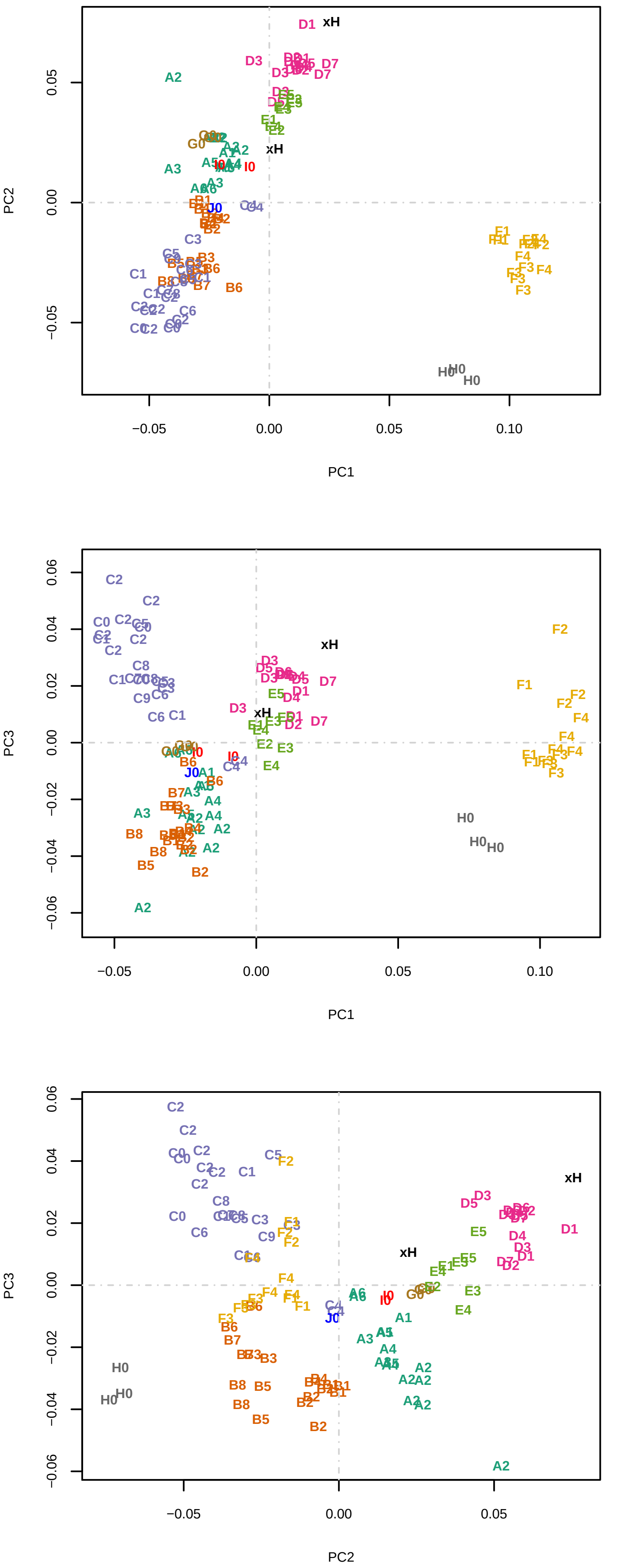

UPGMA tree (K80): Pt09 Second sample P/S region

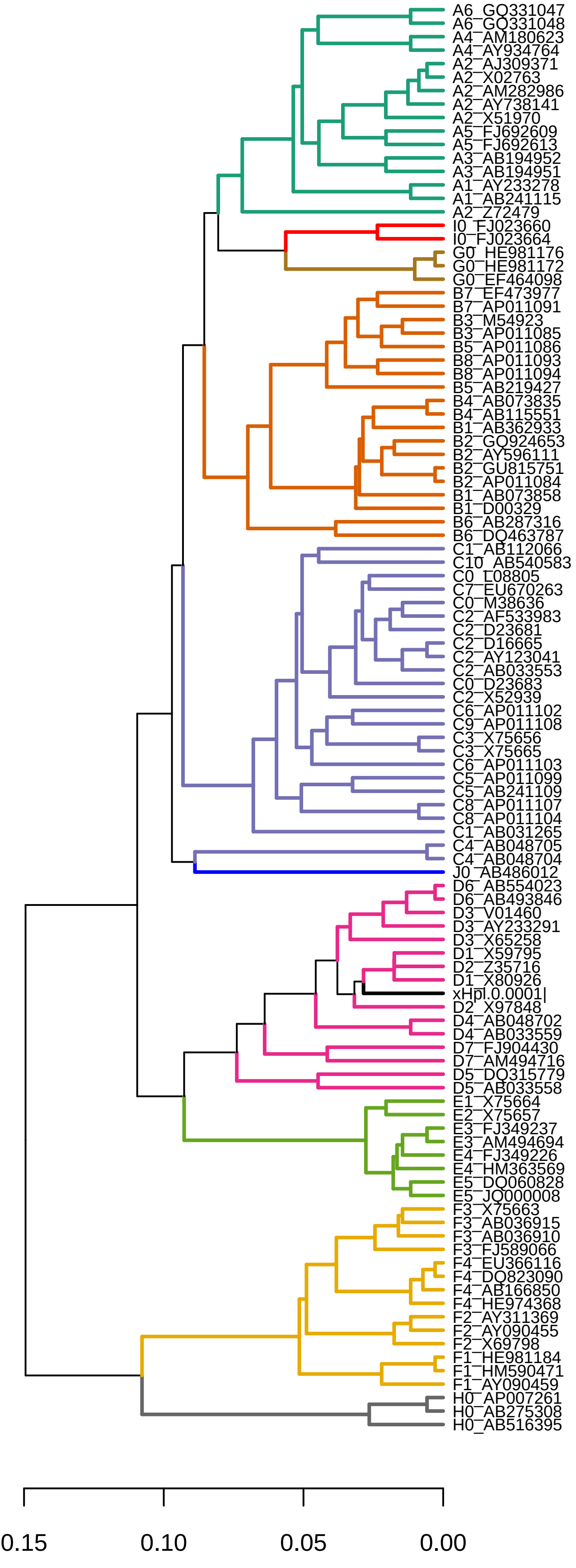

MDS map (K80): Pt09 Second sample P/S region

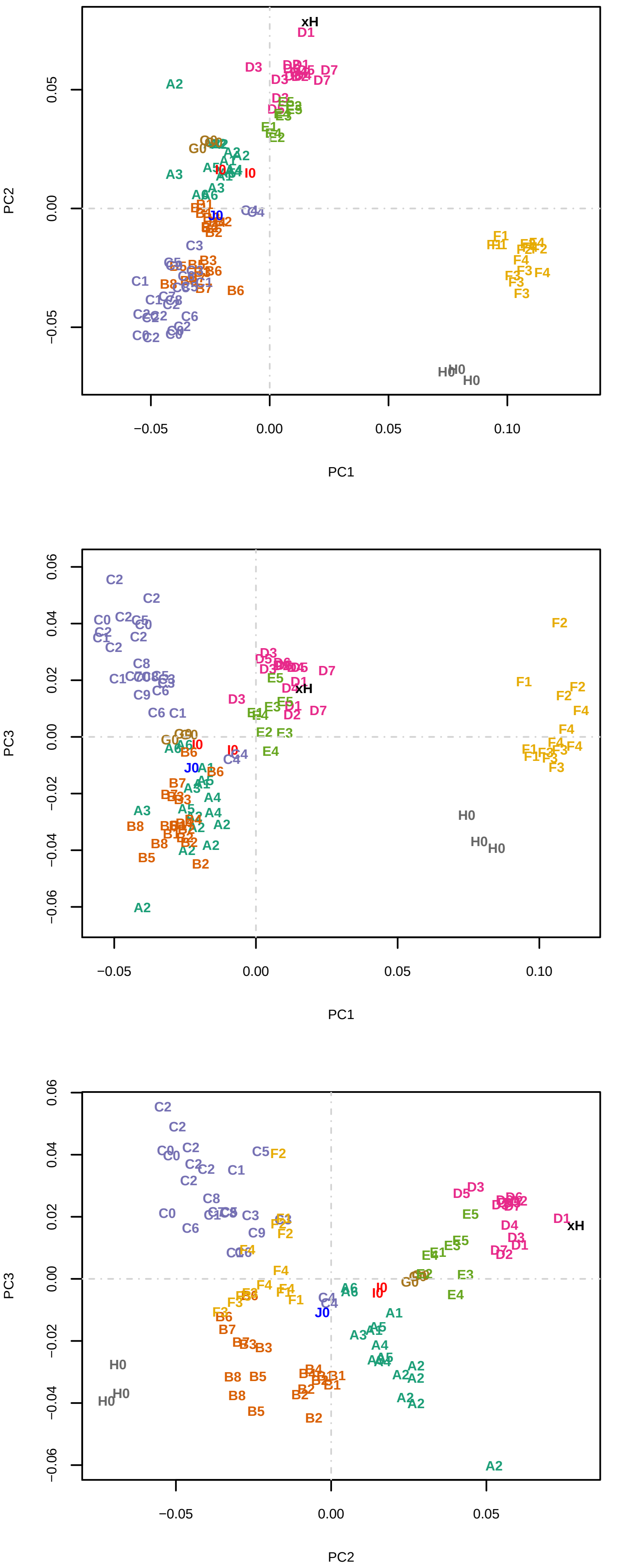

UPGMA tree (K80): Pt09 Third sample P/S region

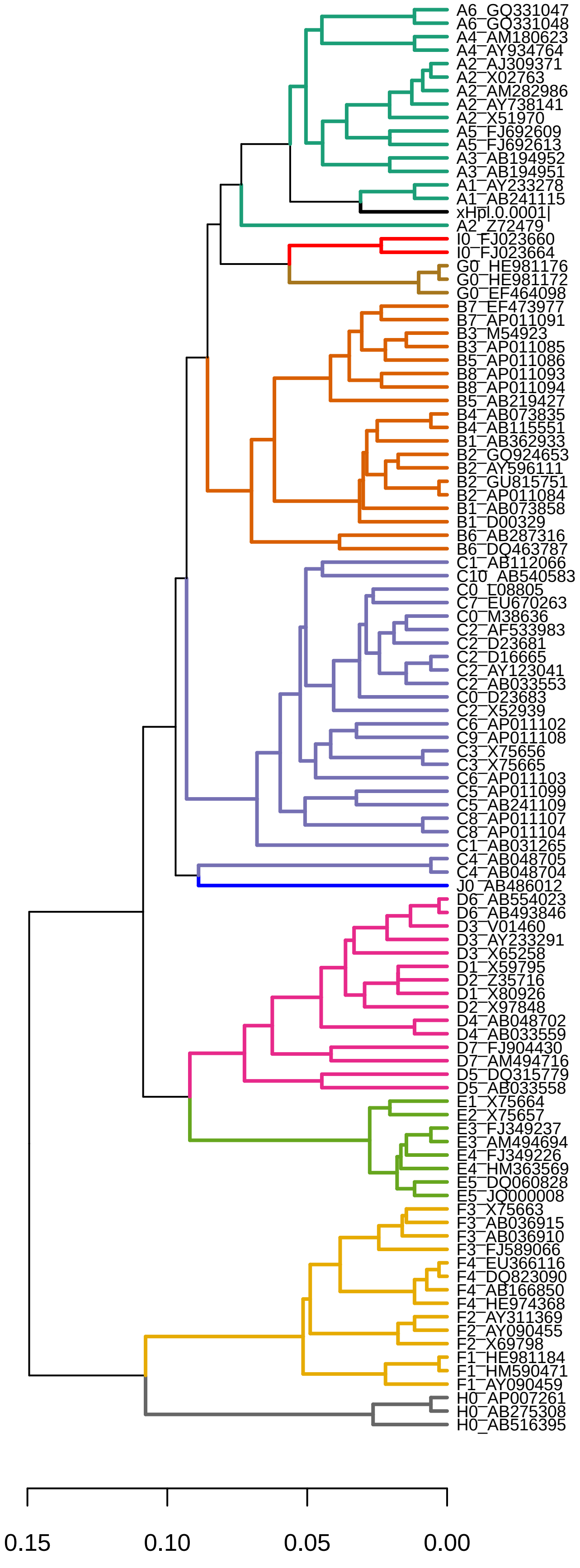

MDS map (K80): Pt09 Third sample P/S region

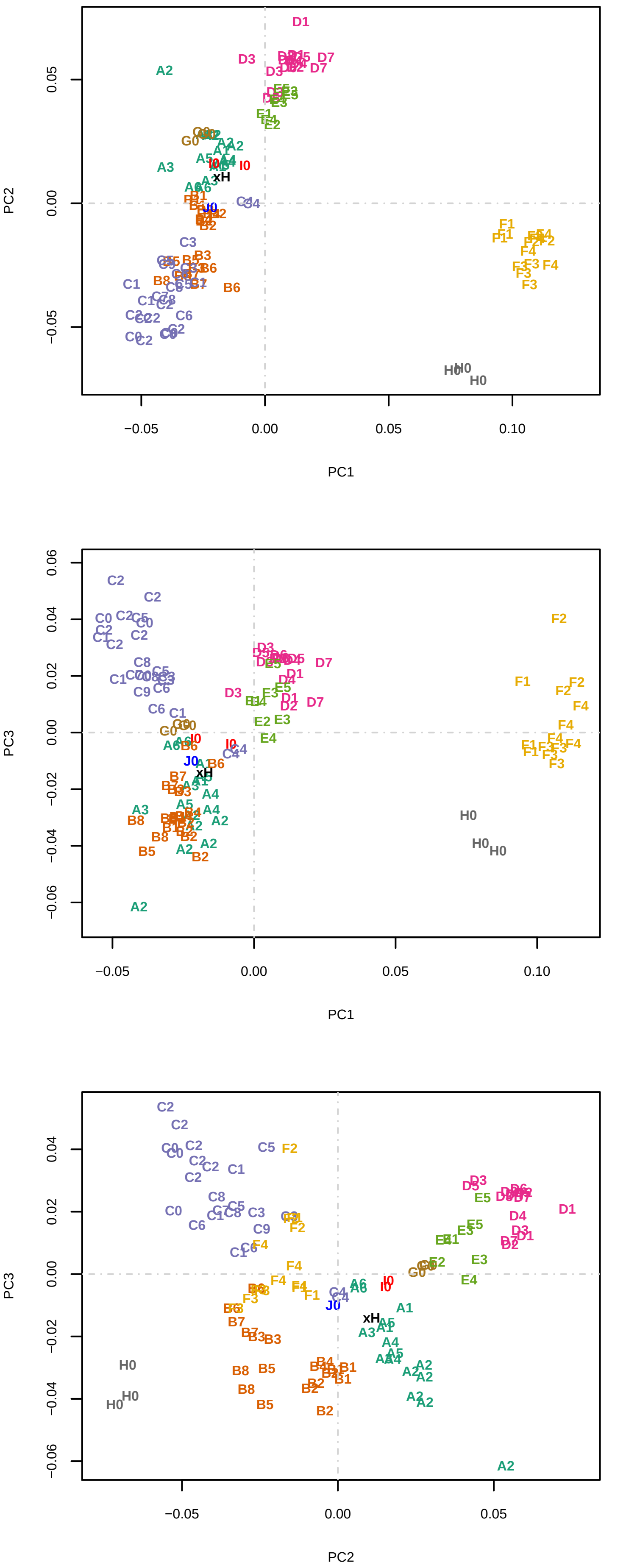

UPGMA tree (K80): Pt10 First sample P/S region

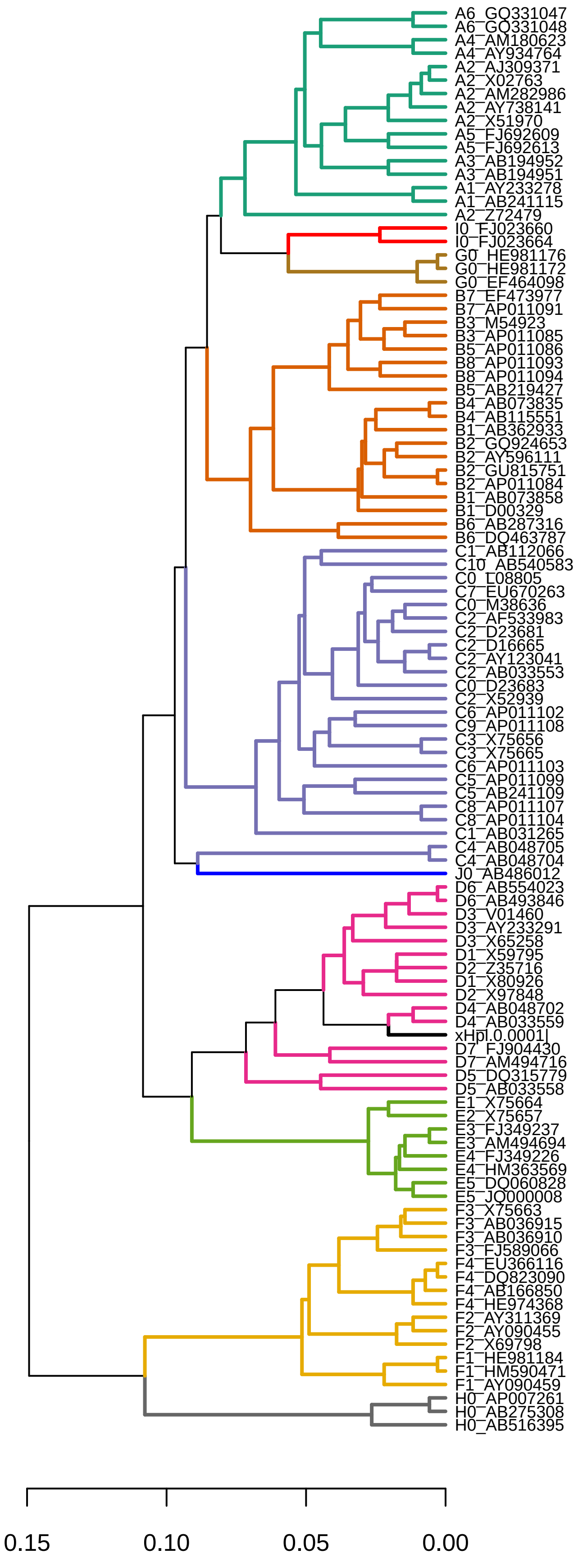

MDS map (K80): Pt10 First sample P/S region

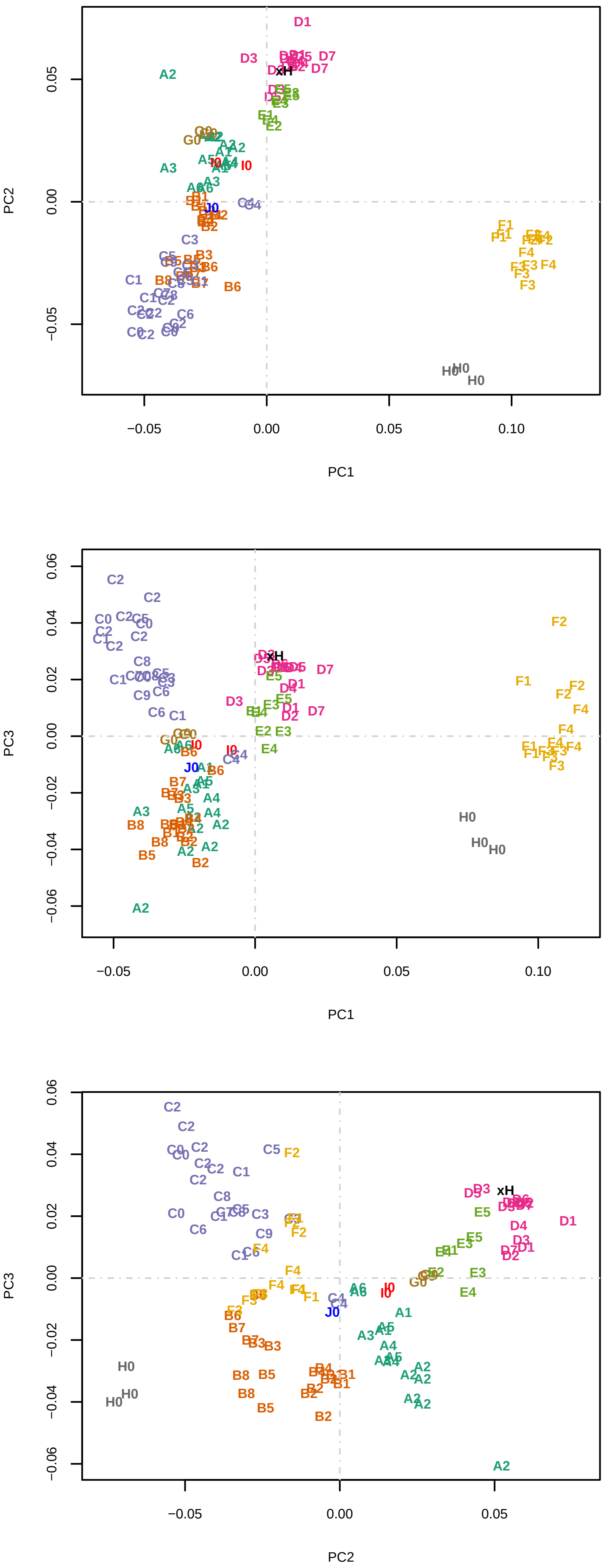

UPGMA tree (K80): Pt10 Second sample P/S region

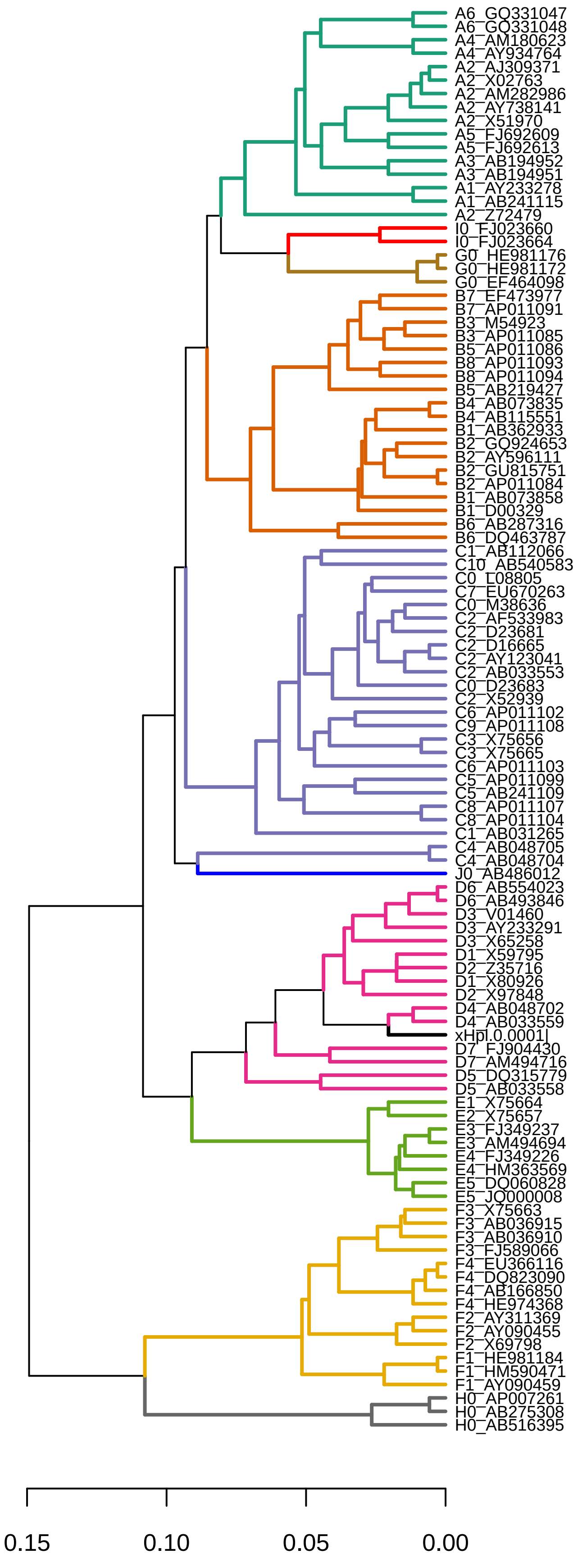

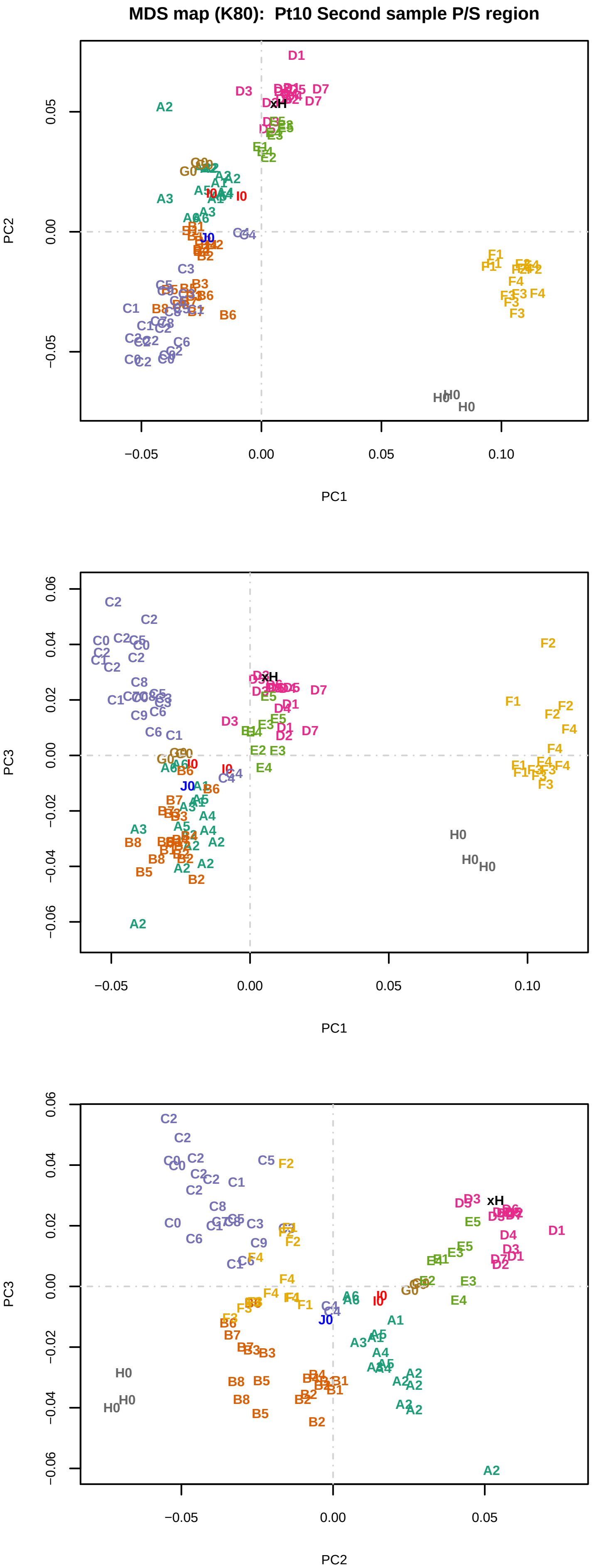
MDS map (K80): Pt10 Second sample P/S region
PC2
0.05
0.00
−0.05
−0.05
0.00
0.05
0.10
PC1
PC3
0.06
0.04
0.02
0.00
−0.02
−0.04
−0.06
−0.05
0.00
0.05
0.10
PC1
PC3
0.06
0.04
0.02
0.00
−0.02
−0.04
−0.06
−0.05
0.00
0.05
PC2

UPGMA tree (K80): Pt10 Third sample P/S region

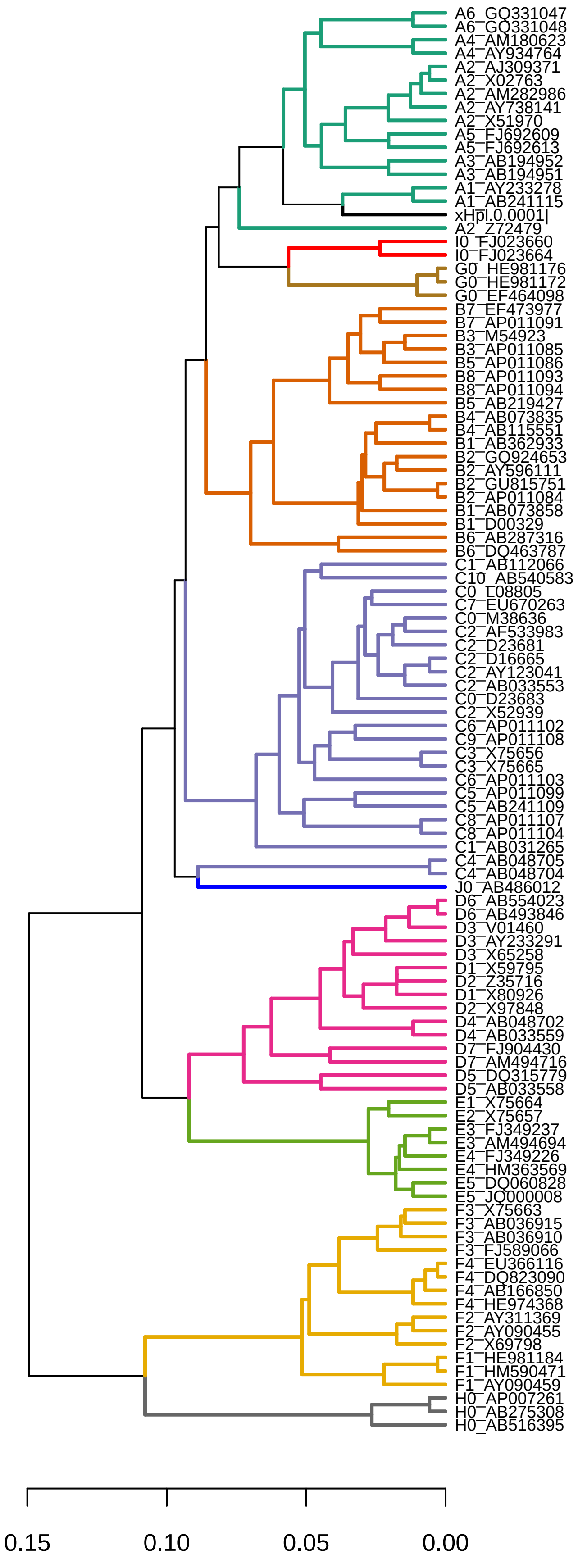

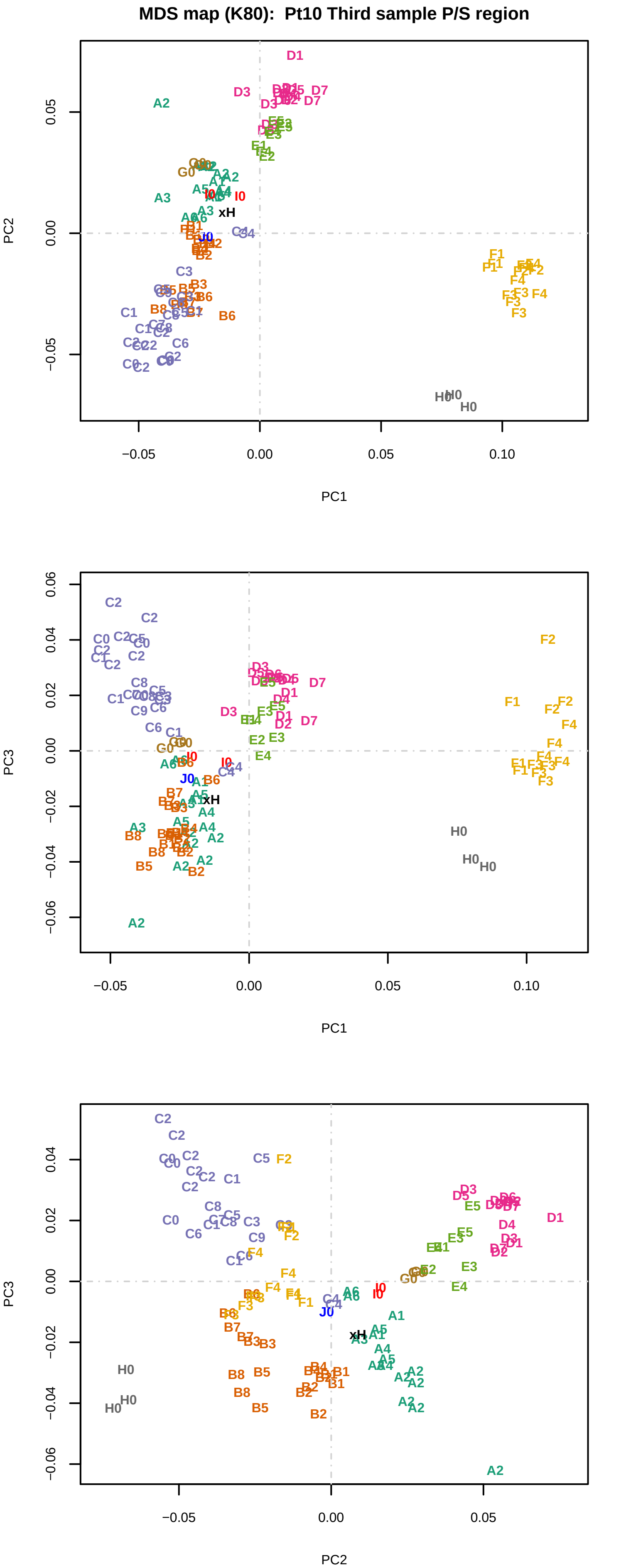
MDS map (K80): Pt10 Third sample P/S region
PC1
PC2
PC3
−0.05
0.00
0.05
0.10
0.06
0.04
0.02
−0.02
−0.04
−0.06
