# Supplementary material for: Complex Genotype Mixtures Analyzed by Deep Sequencing in Two Different Regions of Hepatitis B Virus
Source: PLoS One. 2015 Dec 29;10(12):e0144816. doi: 10.1371/journal.pone.0144816 (PMC4695080; doi:10.1371/journal.pone.0144816)
Supplement: S5 File — (PDF) [file pone.0144816.s005.pdf]

PGMA tree (K80): Pt01 First sample X/preCore region

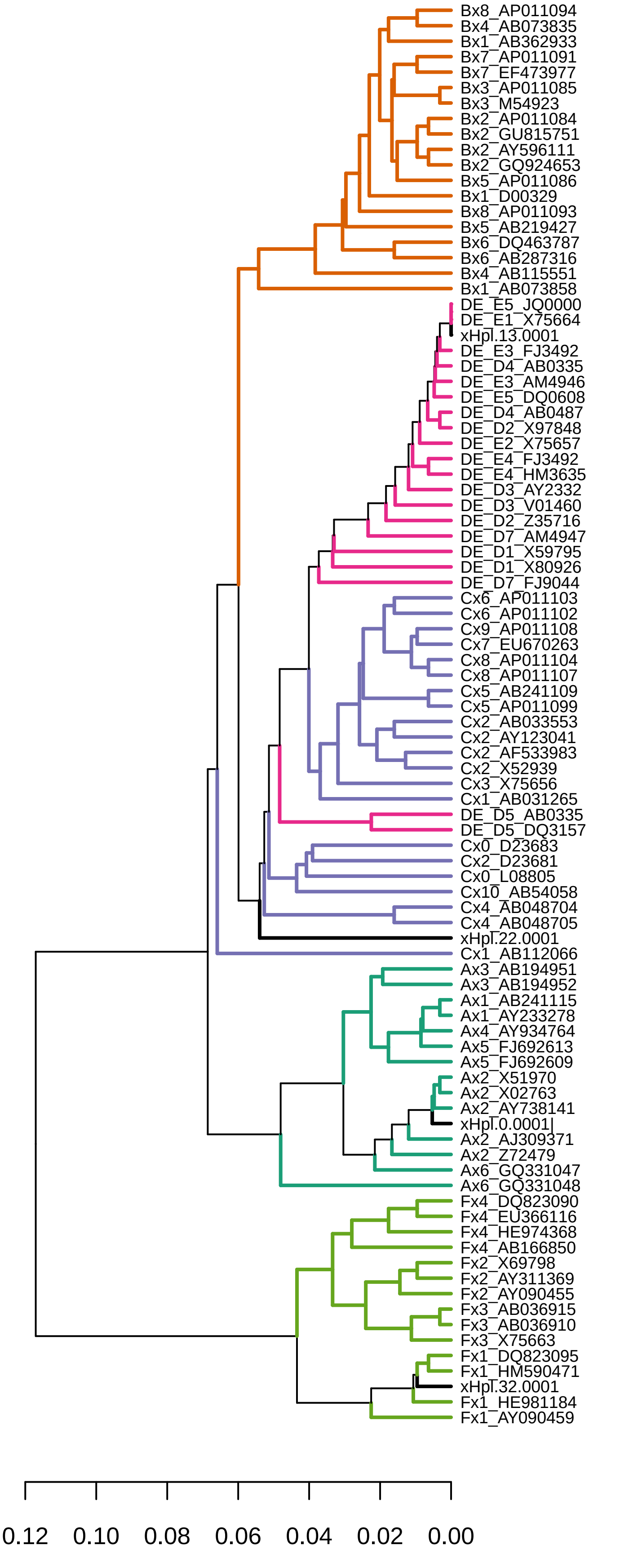

MDS map (K80): Pt01 First sample X/preCore region

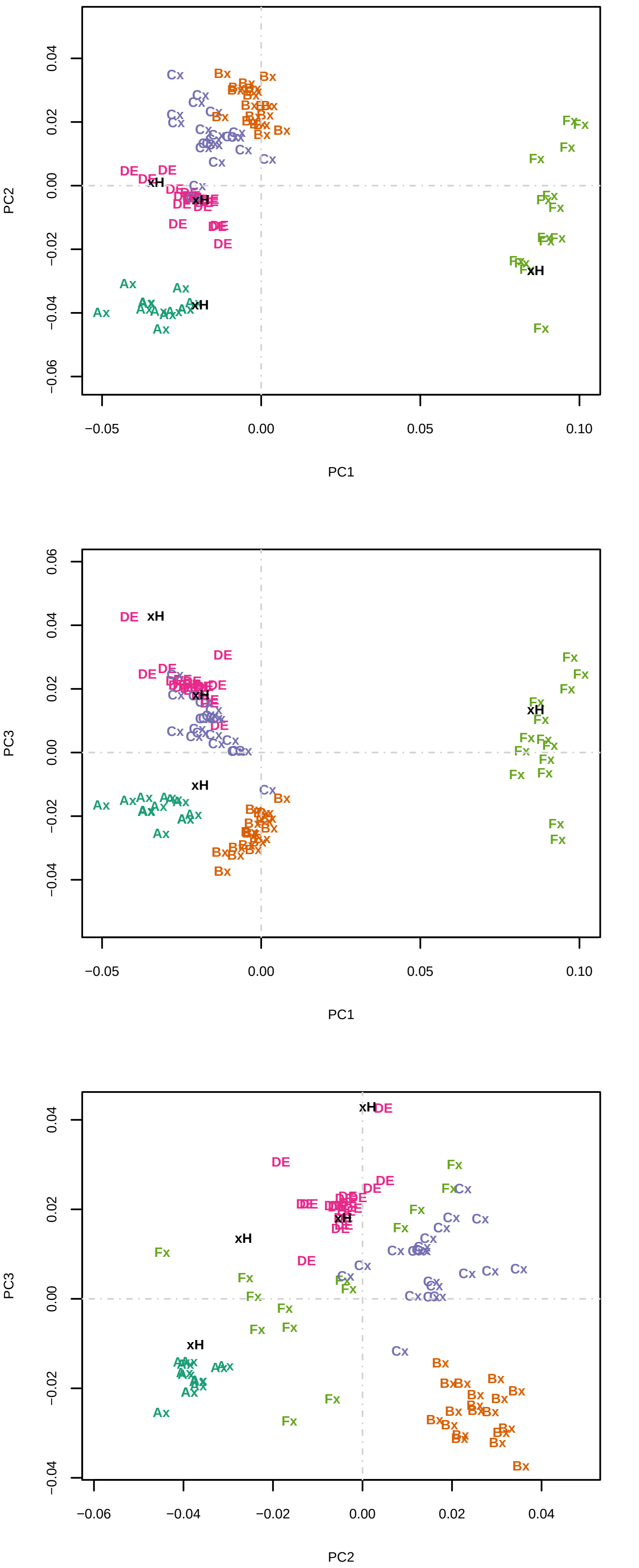

GMA tree (K80): Pt01 Second sample X/preCore region

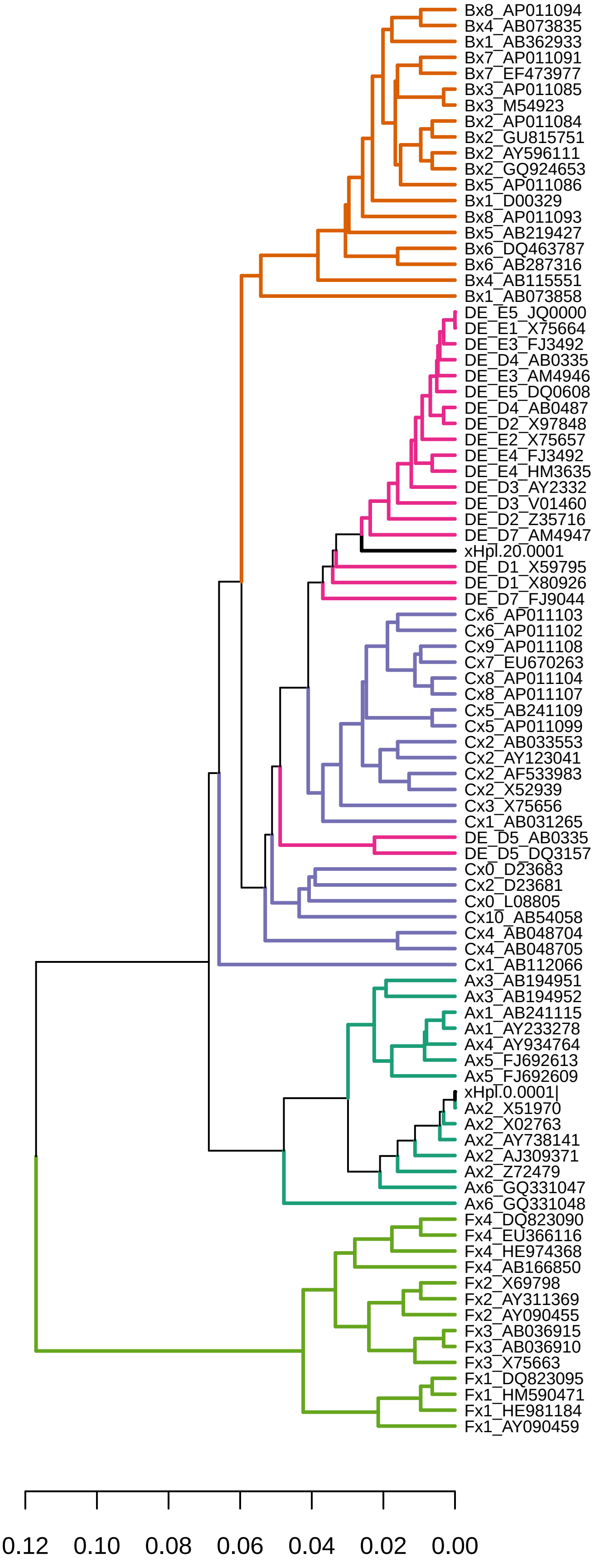

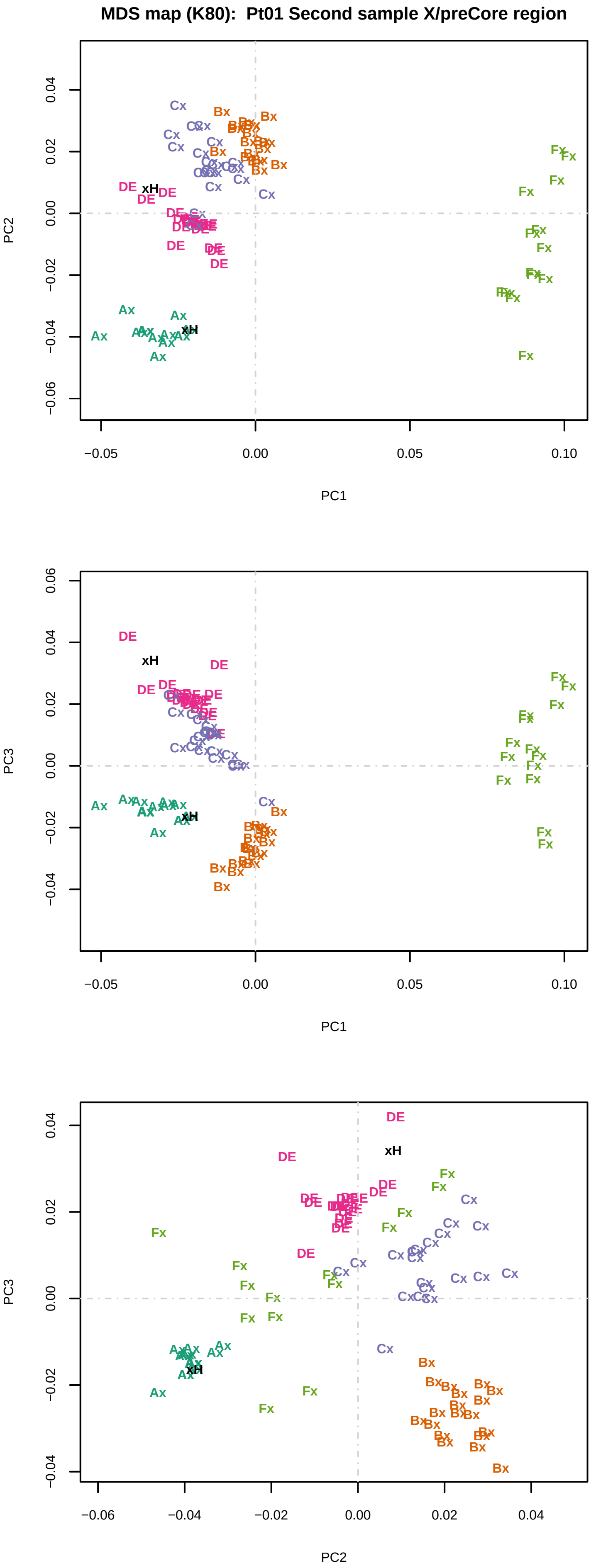
MDS map (K80): Pt01 Second sample X/preCore region
PC1
PC2
PC3
−0.05
0.00
0.05
0.10
−0.06
−0.04
−0.02
0.00
0.02
0.04
0.06
Ax
Bx
Cx
DE
Fx
xH

PGMA tree (K80): Pt01 Third sample X/preCore region

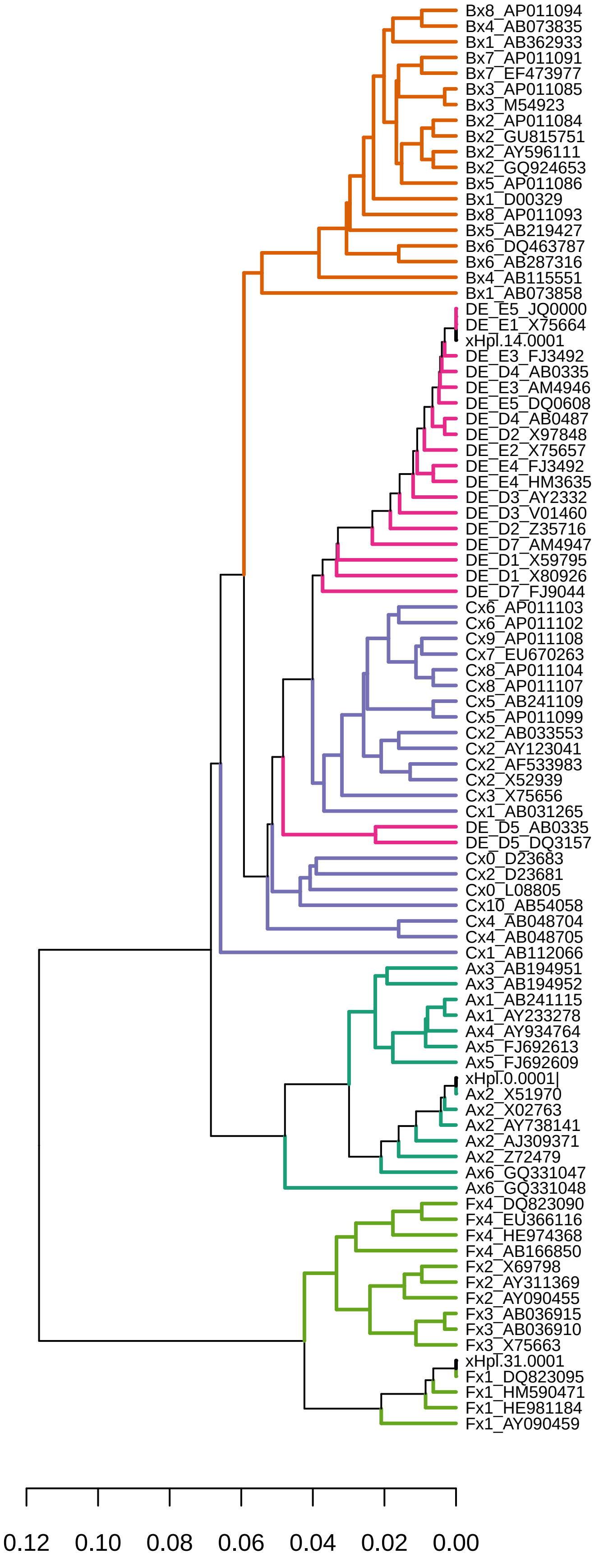

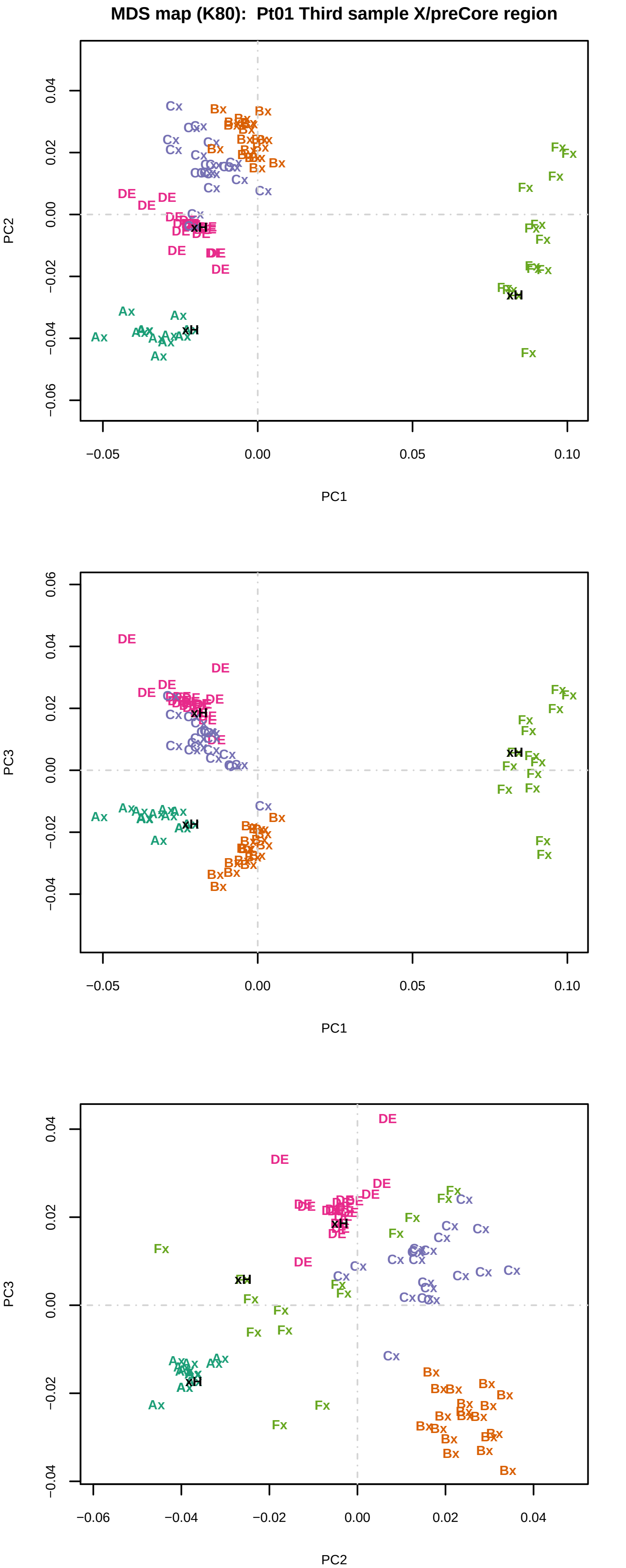

MDS map (K80): Pt01 Third sample X/preCore region
PC1
PC2
PC3
Ax
Bx
Cx
DE
Fx
xH

PGMA tree (K80): Pt02 First sample X/preCore region

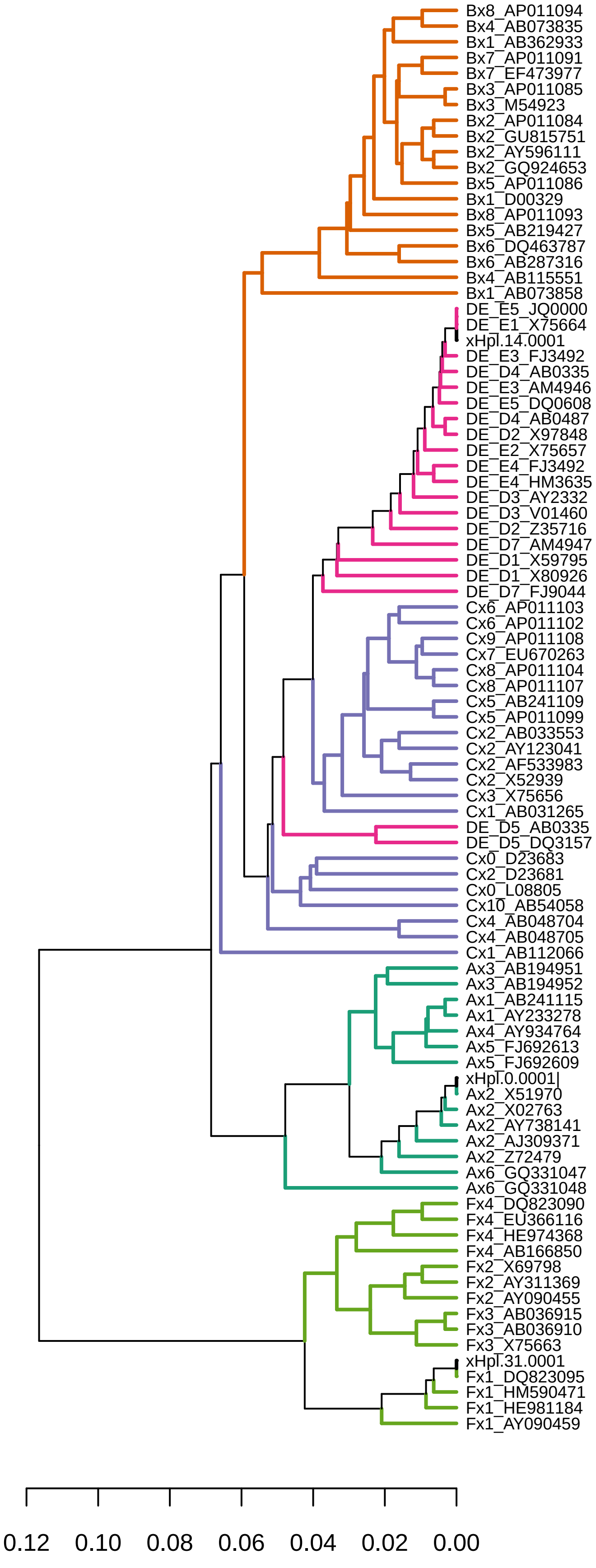

MDS map (K80): Pt02 First sample X/preCore region

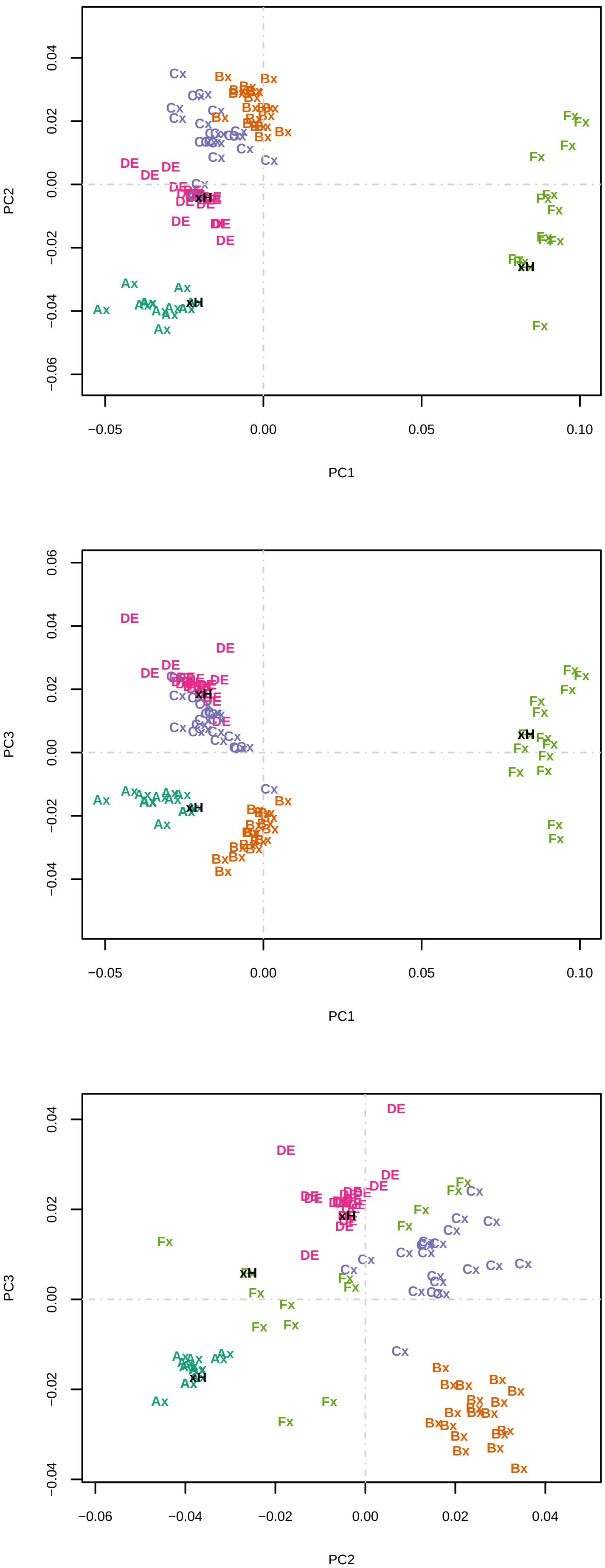

GMA tree (K80): Pt02 Second sample X/preCore region

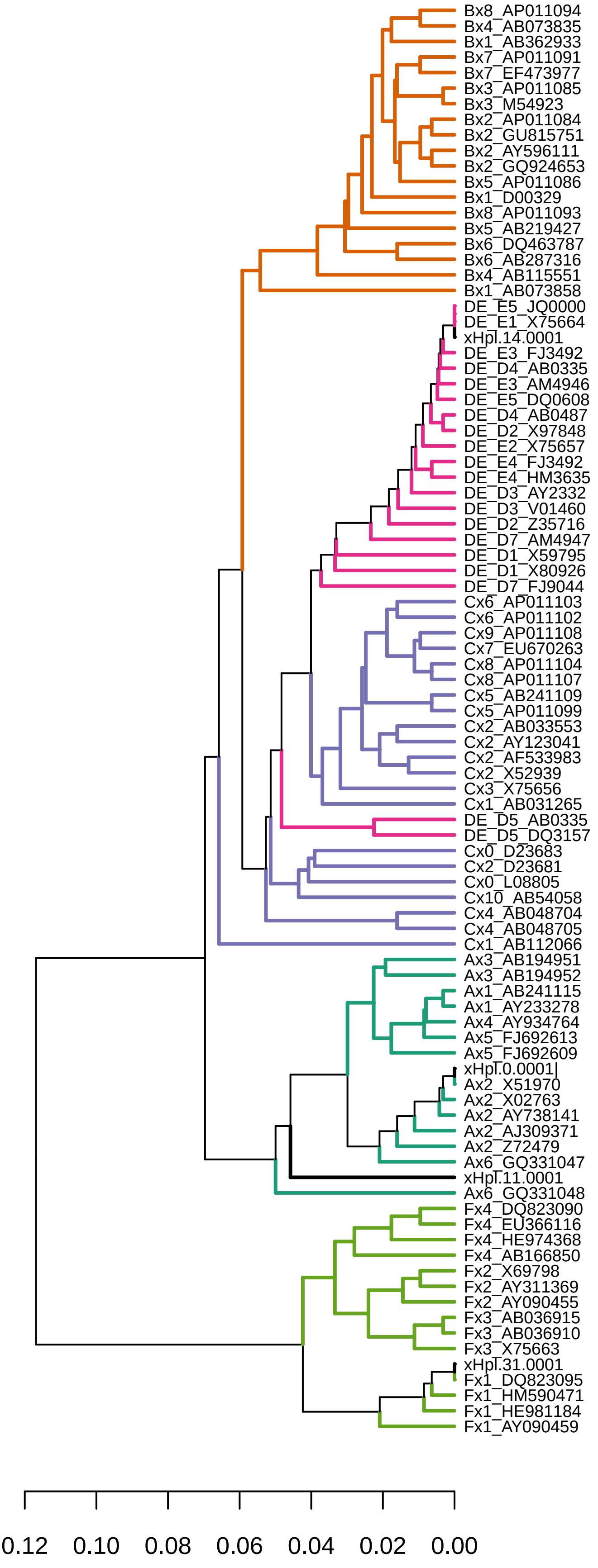

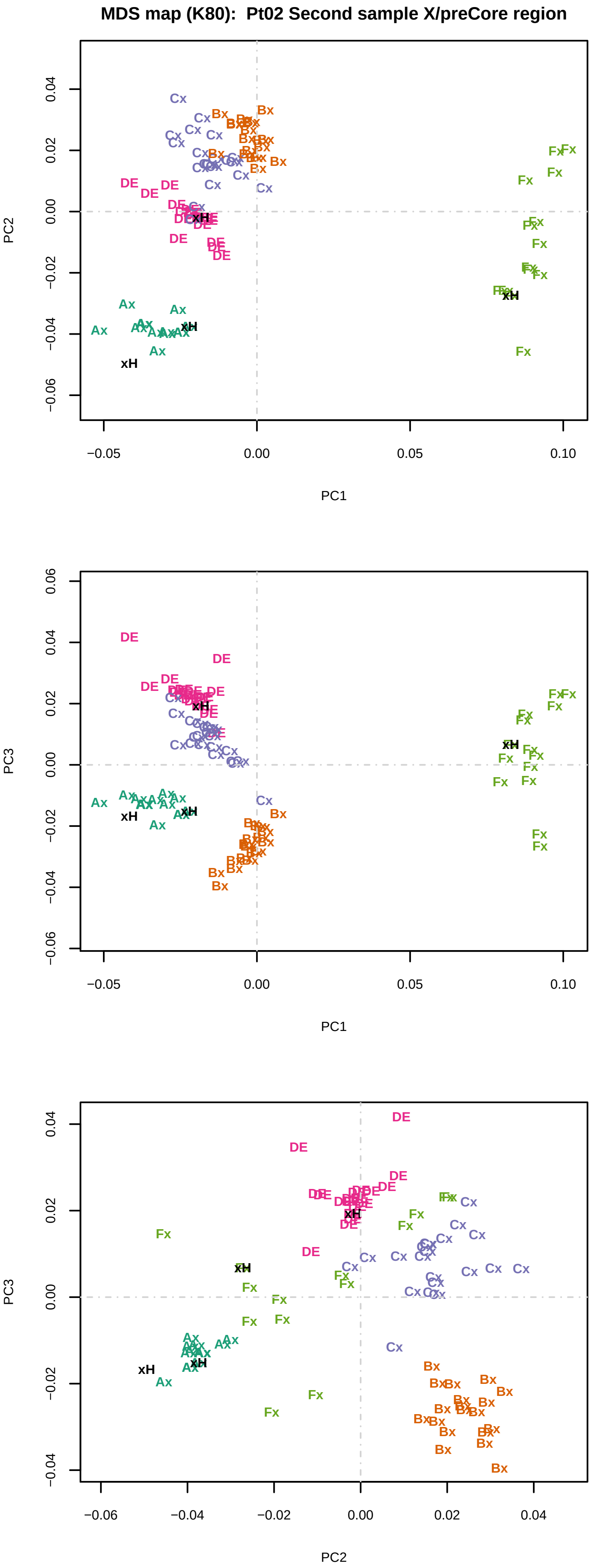
MDS map (K80): Pt02 Second sample X/preCore region
PC1
PC2
PC3
Ax
Bx
Cx
DE
Fx
xH
−0.05
0.00
0.05
0.10
−0.06
−0.04
−0.02
0.00
0.02
0.04
0.06

PGMA tree (K80): Pt02 Third sample X/preCore region

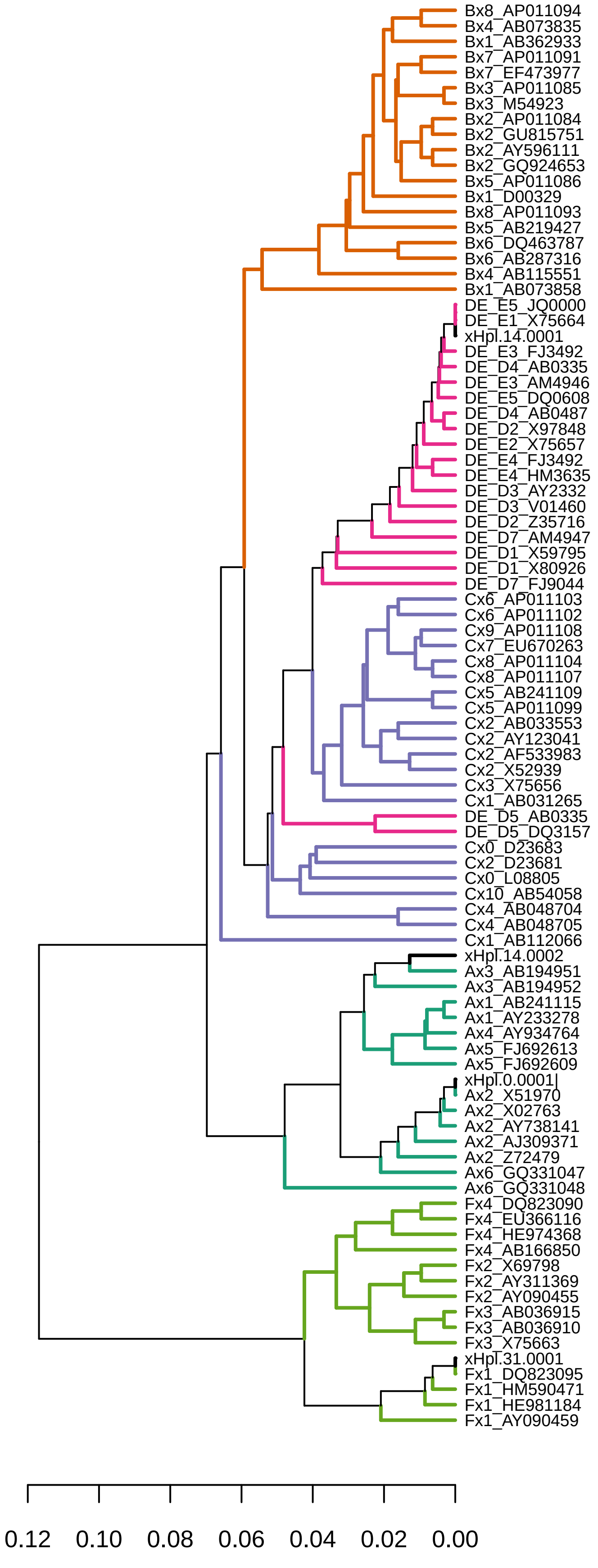

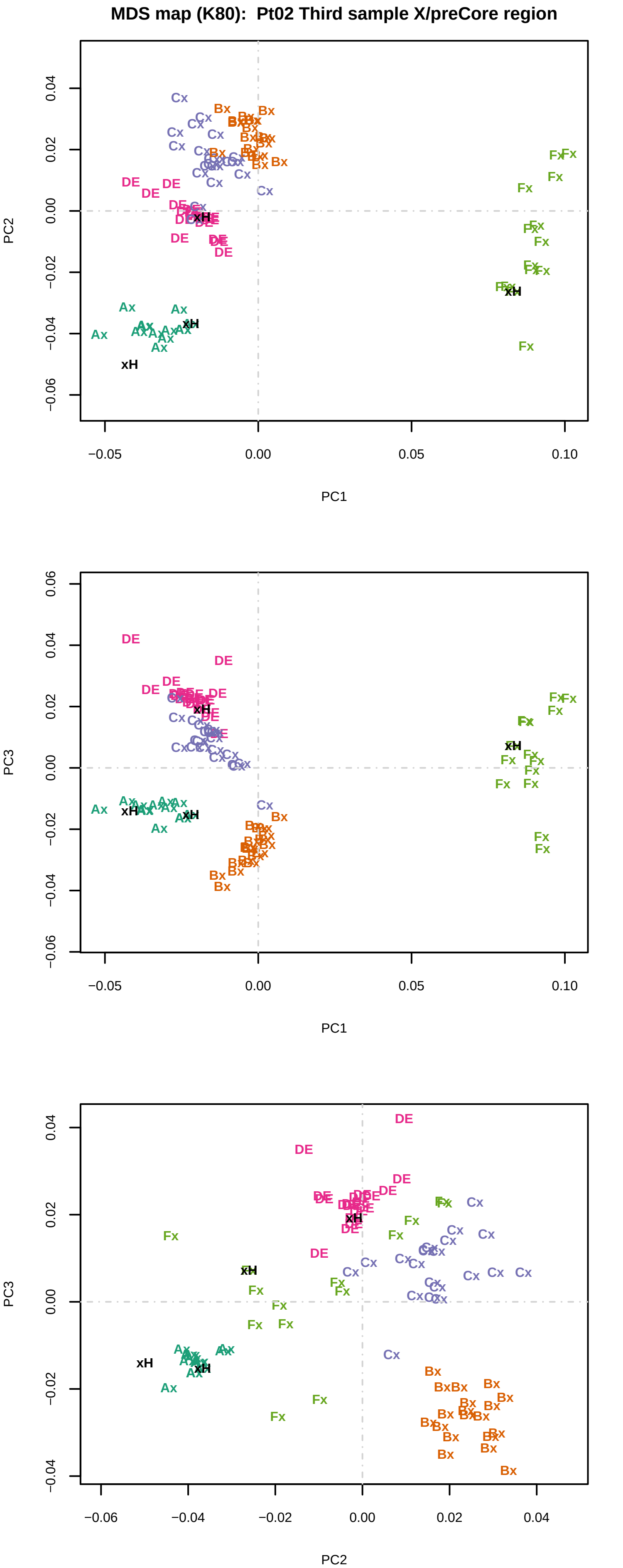
MDS map (K80): Pt02 Third sample X/preCore region
PC1
PC2
PC1
PC3
PC2
PC3

PGMA tree (K80): Pt03 First sample X/preCore region

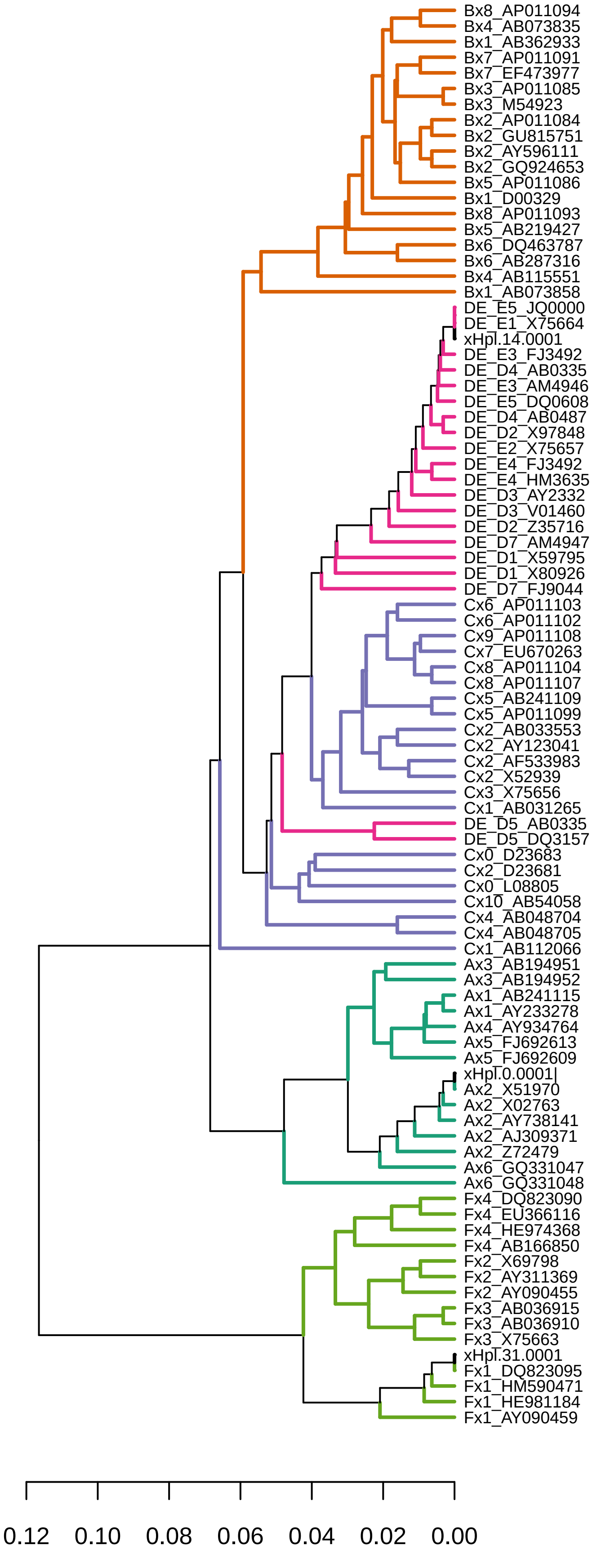

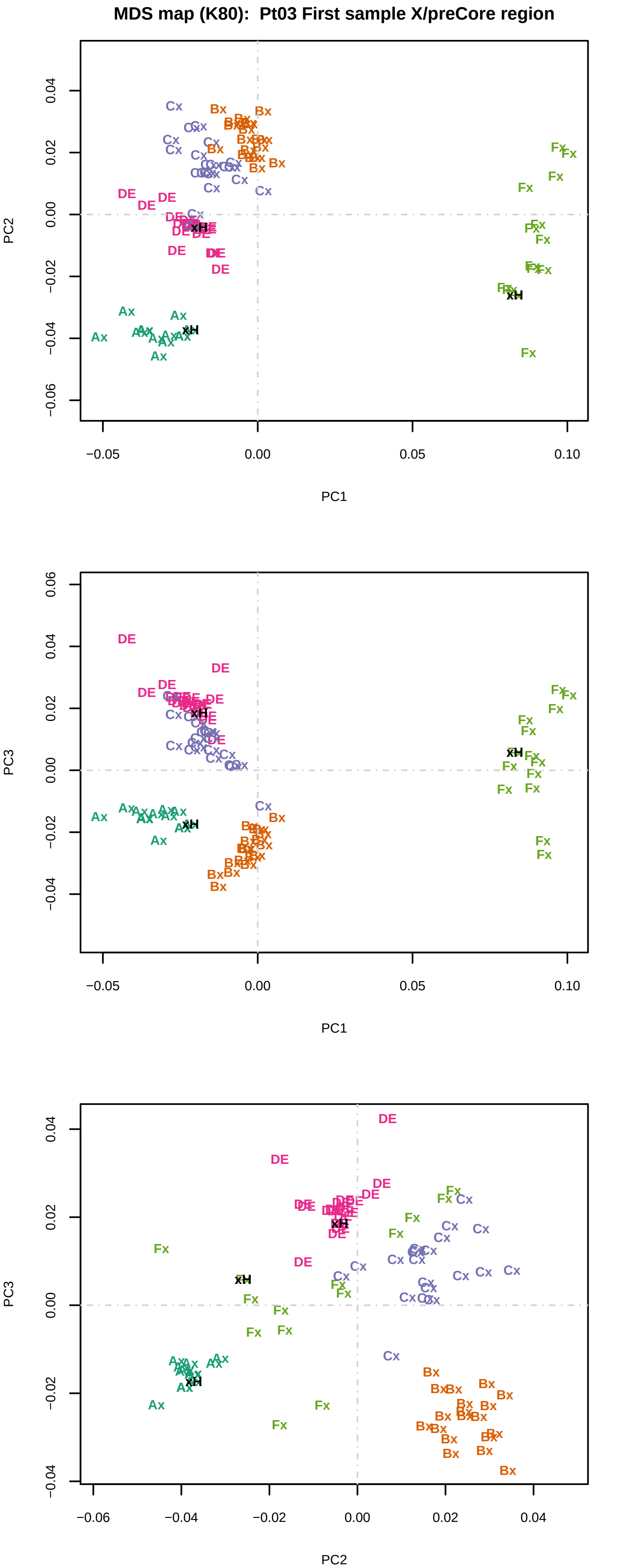

MDS map (K80): Pt03 First sample X/preCore region
PC1
PC2
PC3
−0.06
−0.05
−0.04
−0.02
0.00
0.02
0.04
0.05
0.06
0.10
Ax
Bx
Cx
DE
Fx
xH

GMA tree (K80): Pt03 Second sample X/preCore region

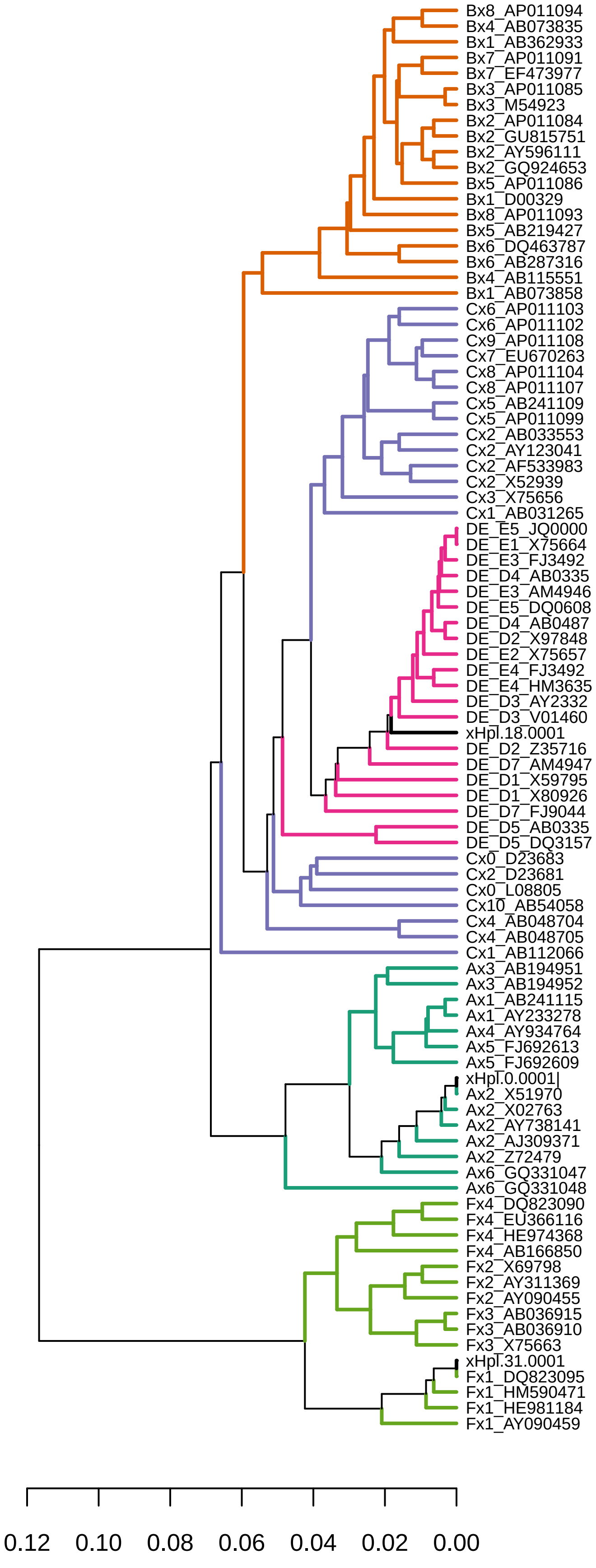

MDS map (K80): Pt03 Second sample X/preCore region

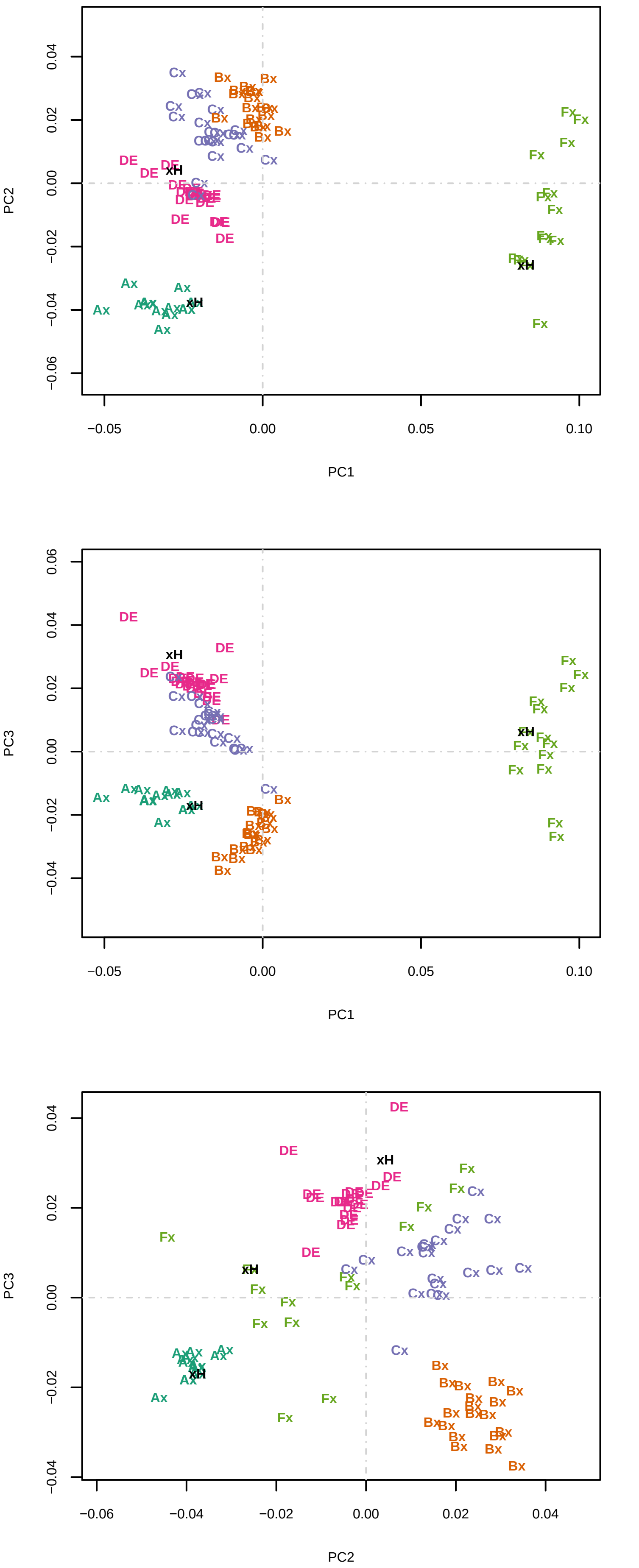

PGMA tree (K80): Pt03 Third sample X/preCore region

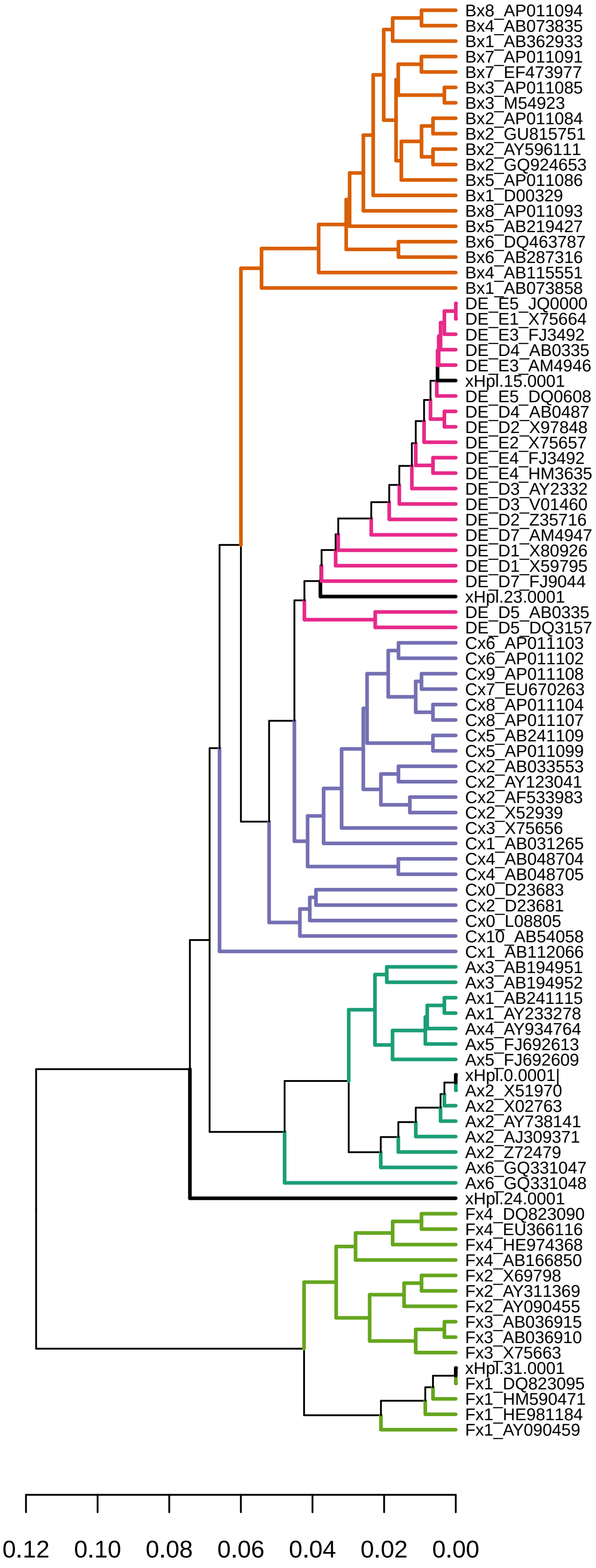

MDS map (K80): Pt03 Third sample X/preCore region

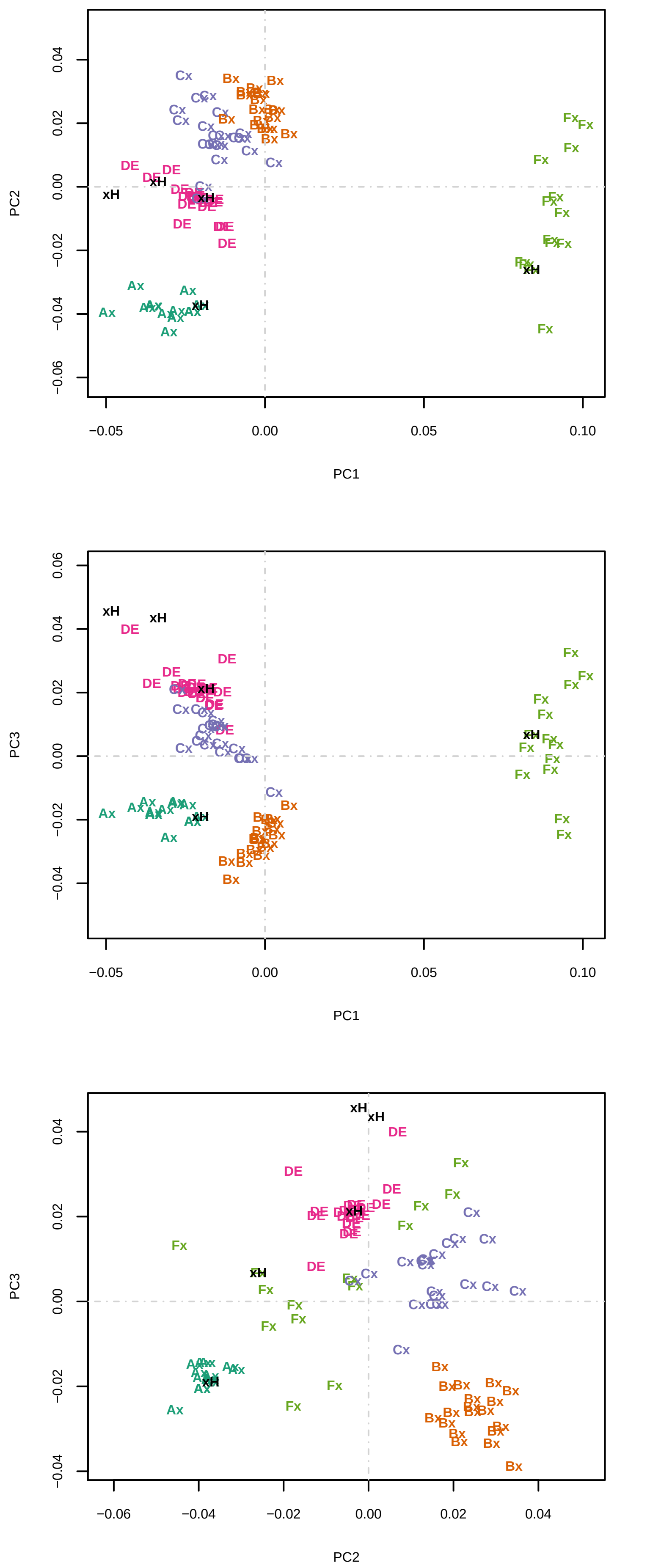

PGMA tree (K80): Pt04 First sample X/preCore region

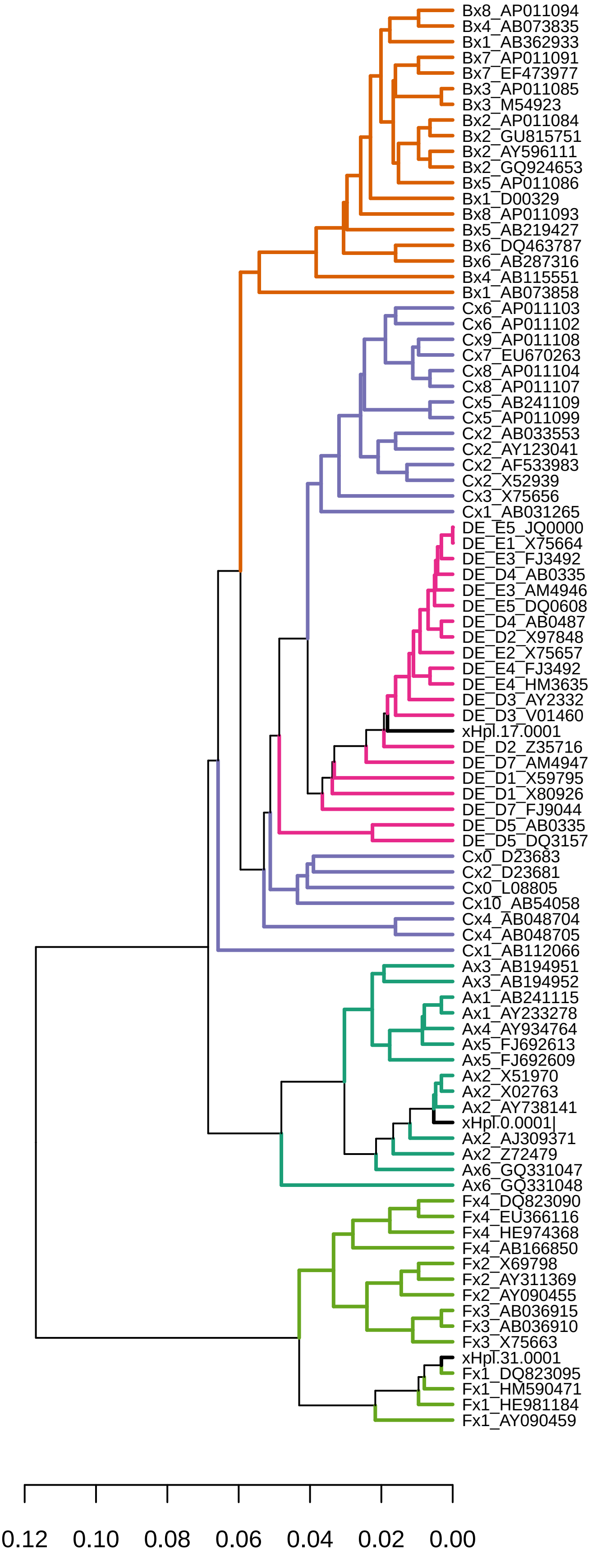

MDS map (K80): Pt04 First sample X/preCore region

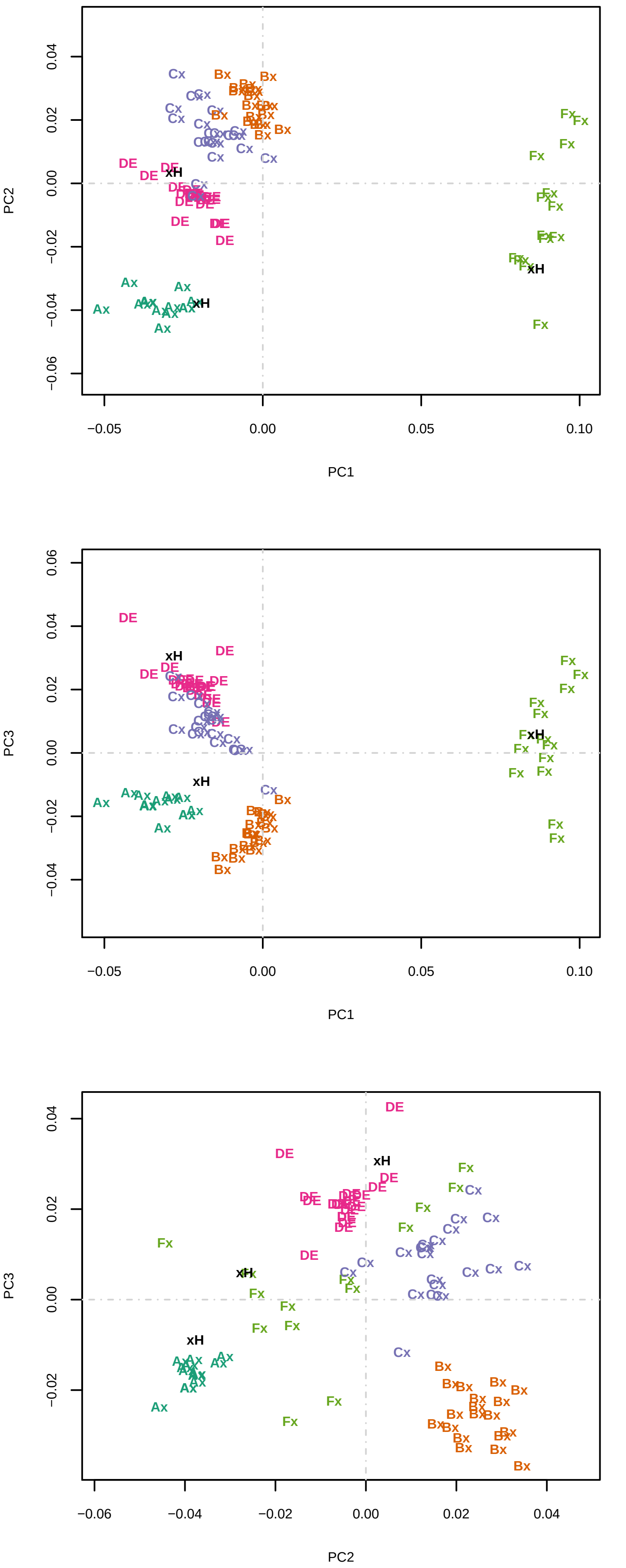

GMA tree (K80): Pt04 Second sample X/preCore region

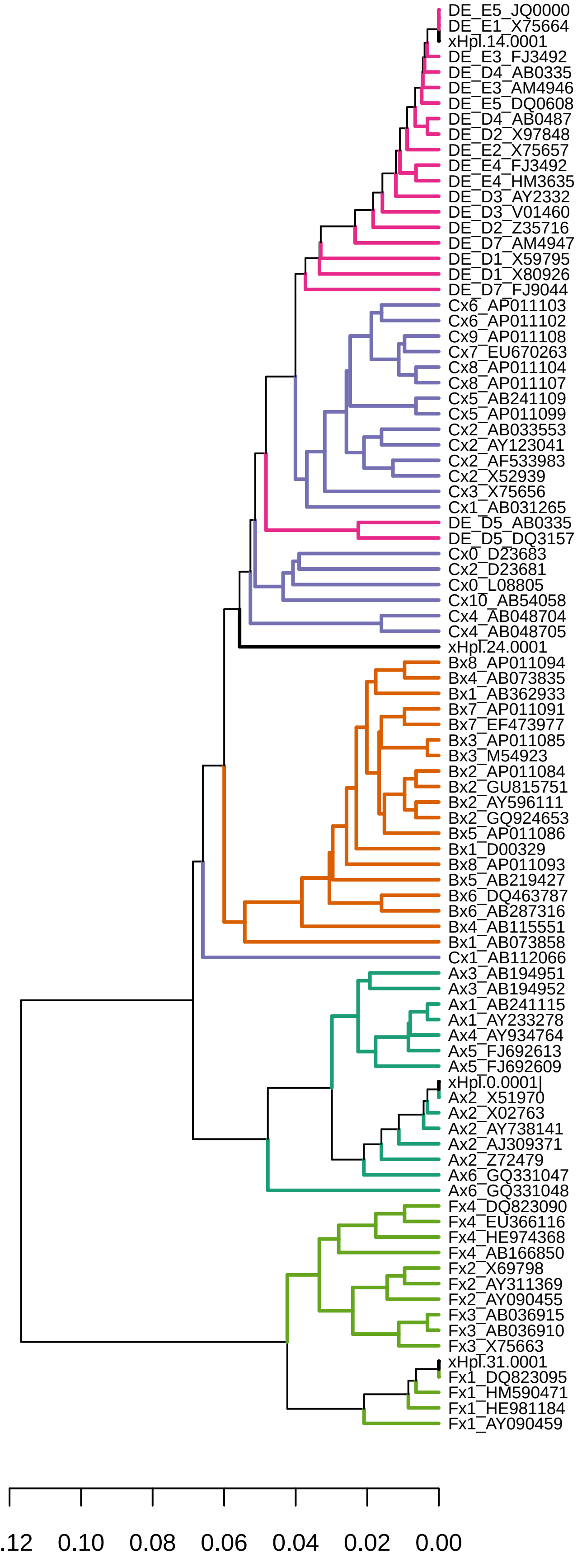

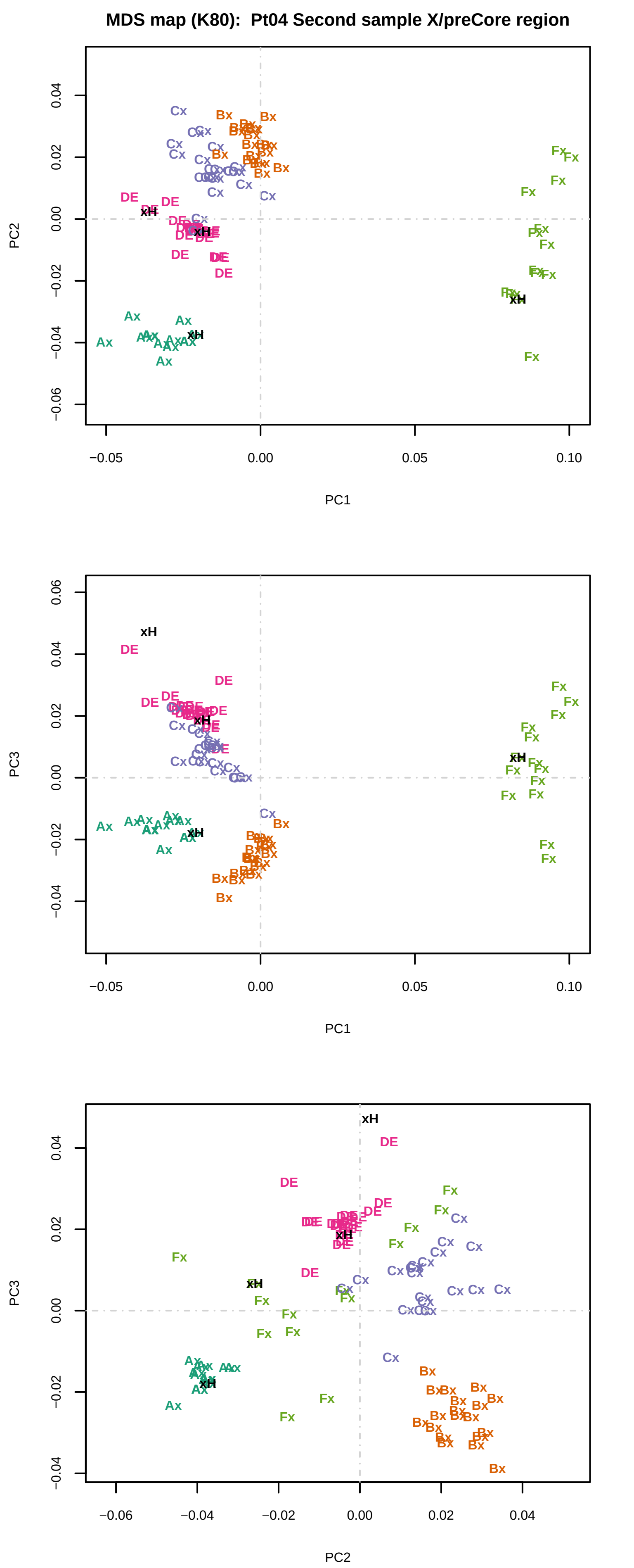

MDS map (K80): Pt04 Second sample X/preCore region
PC1
PC2
PC3
−0.05
0.00
0.05
0.10
0.04
0.02
0.00
−0.02
−0.04
−0.06
0.06
0.06
0.04
0.02
0.00
−0.02
−0.04
−0.06
Ax
Bx
Cx
DE
Fx
xH

PGMA tree (K80): Pt04 Third sample X/preCore region

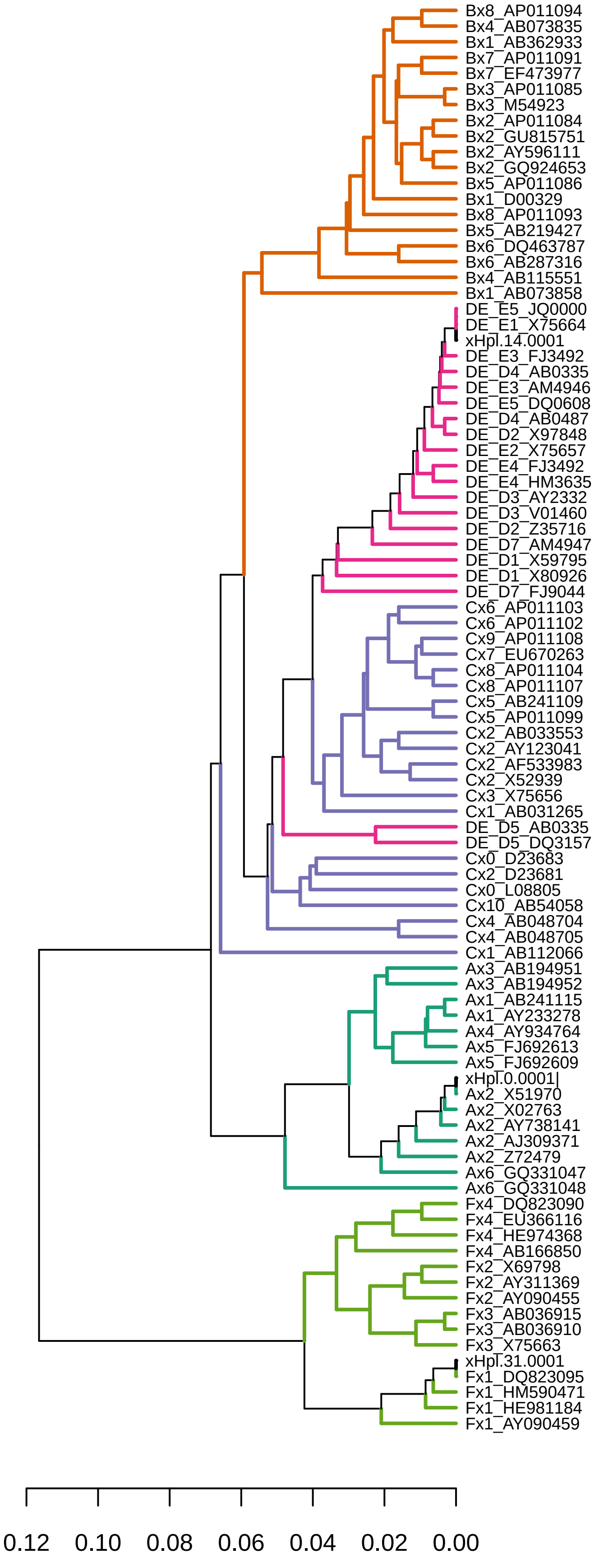

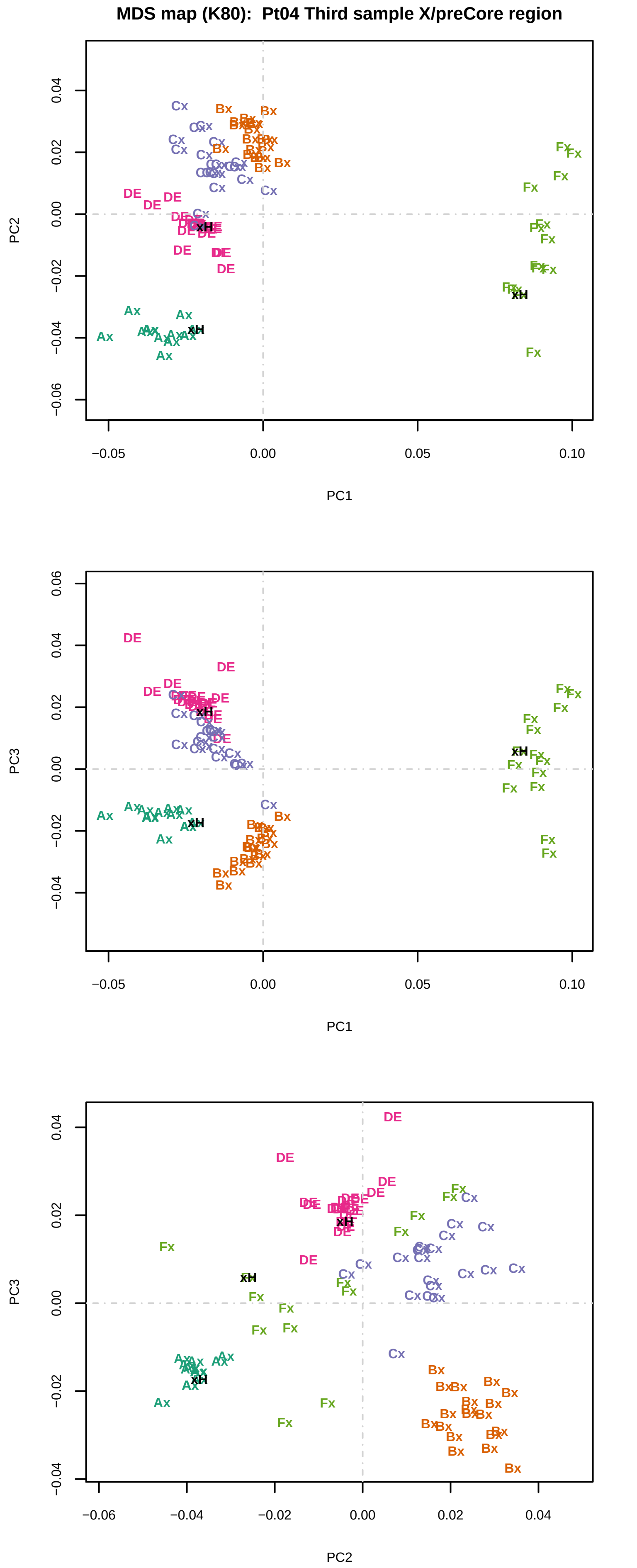

MDS map (K80): Pt04 Third sample X/preCore region
PC1
PC2
PC3
−0.05
0.00
0.05
0.10
−0.06
−0.04
−0.02
0.00
0.02
0.04
0.06
Ax
Bx
Cx
DE
Fx
xH

PGMA tree (K80): Pt05 First sample X/preCore region

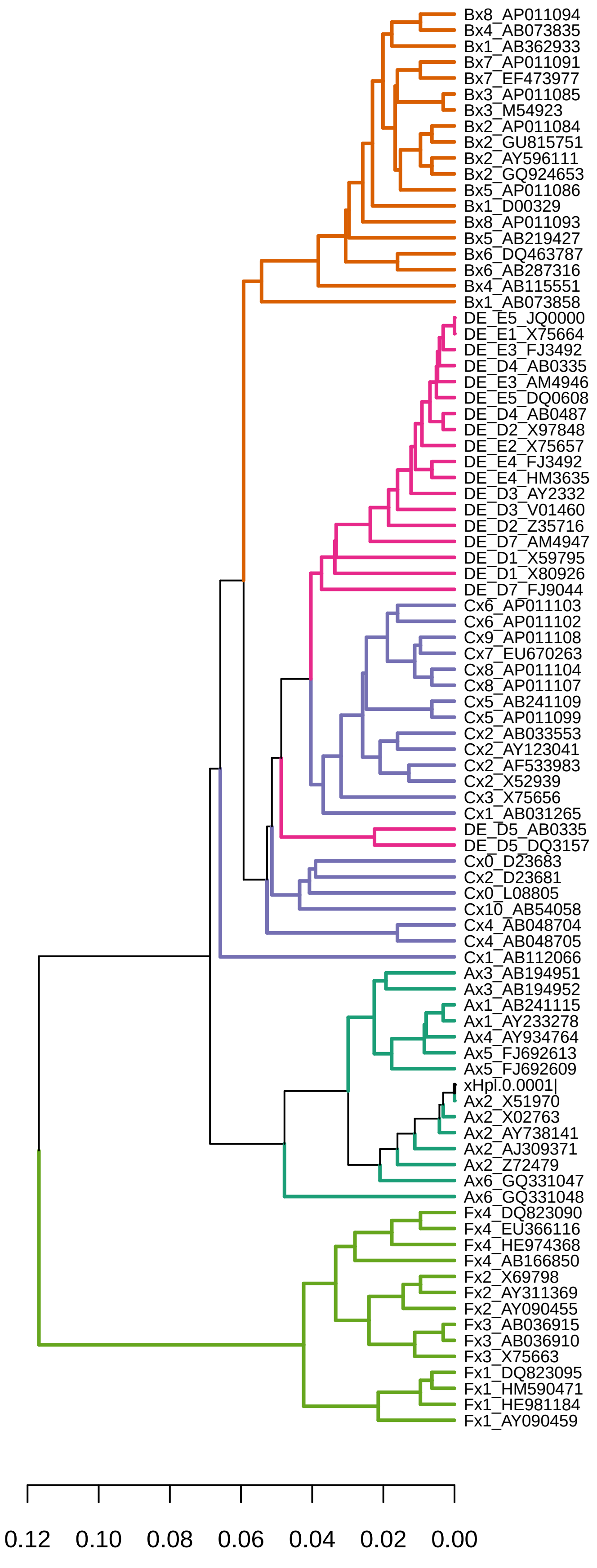

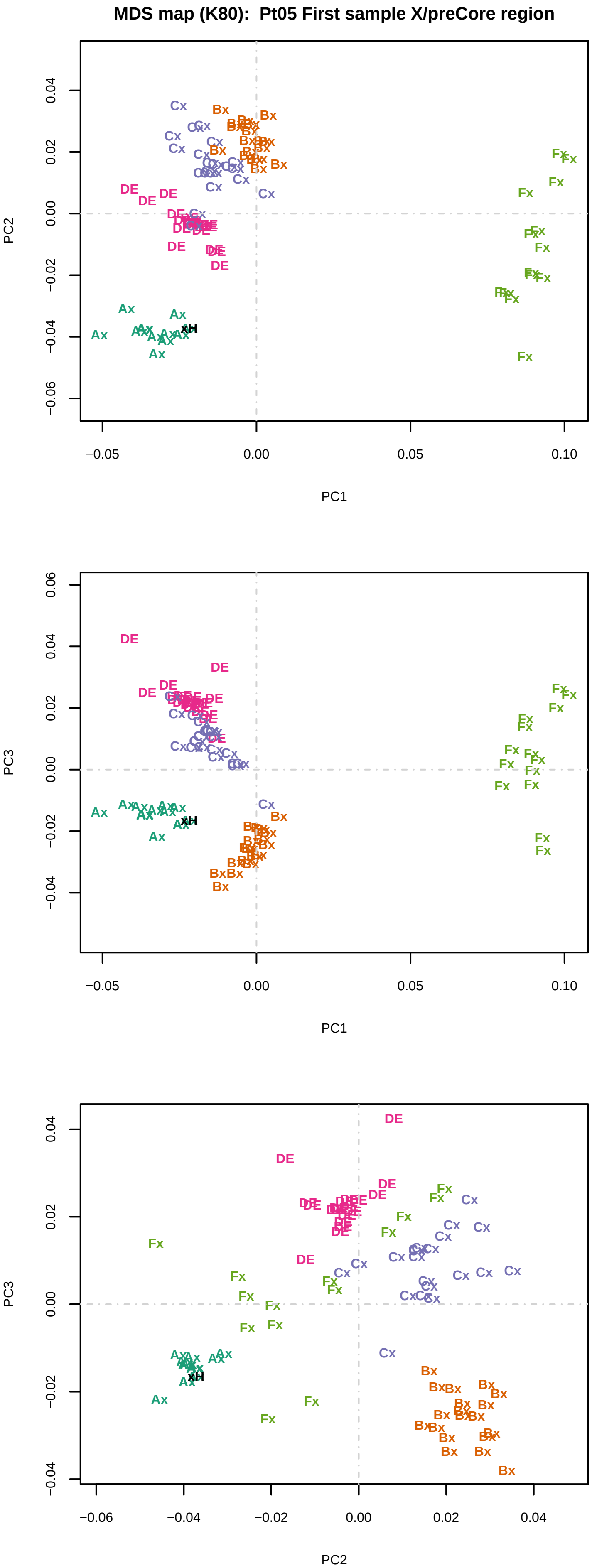
MDS map (K80): Pt05 First sample X/preCore region
PC1
PC2
PC3
−0.05
0.00
0.05
0.10
−0.06
−0.04
−0.02
0.02
0.04
0.06
Ax
Bx
Cx
DE
Fx
xH

GMA tree (K80): Pt05 Second sample X/preCore region

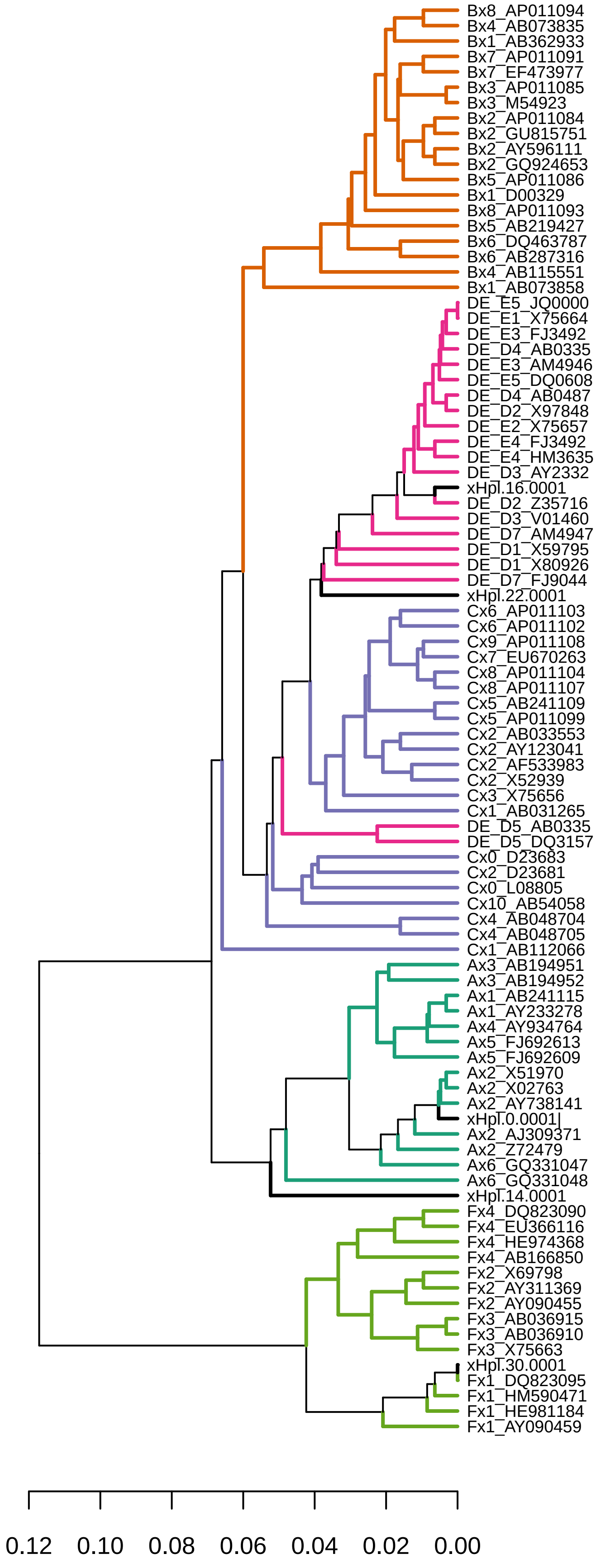

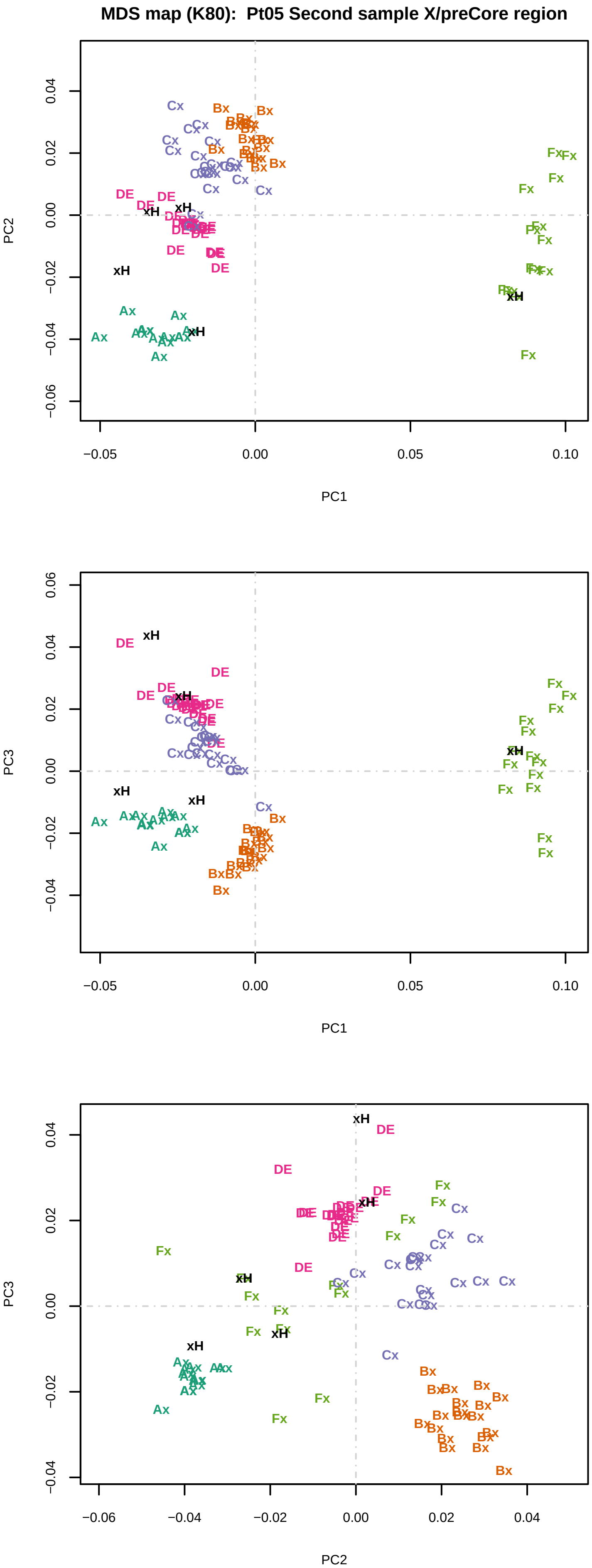
MDS map (K80): Pt05 Second sample X/preCore region
PC1
PC2
PC3
−0.05
0.00
0.05
0.10
−0.06
−0.04
−0.02
0.02
0.04
0.06
Ax
Bx
Cx
DE
Fx
xH

PGMA tree (K80): Pt05 Third sample X/preCore region

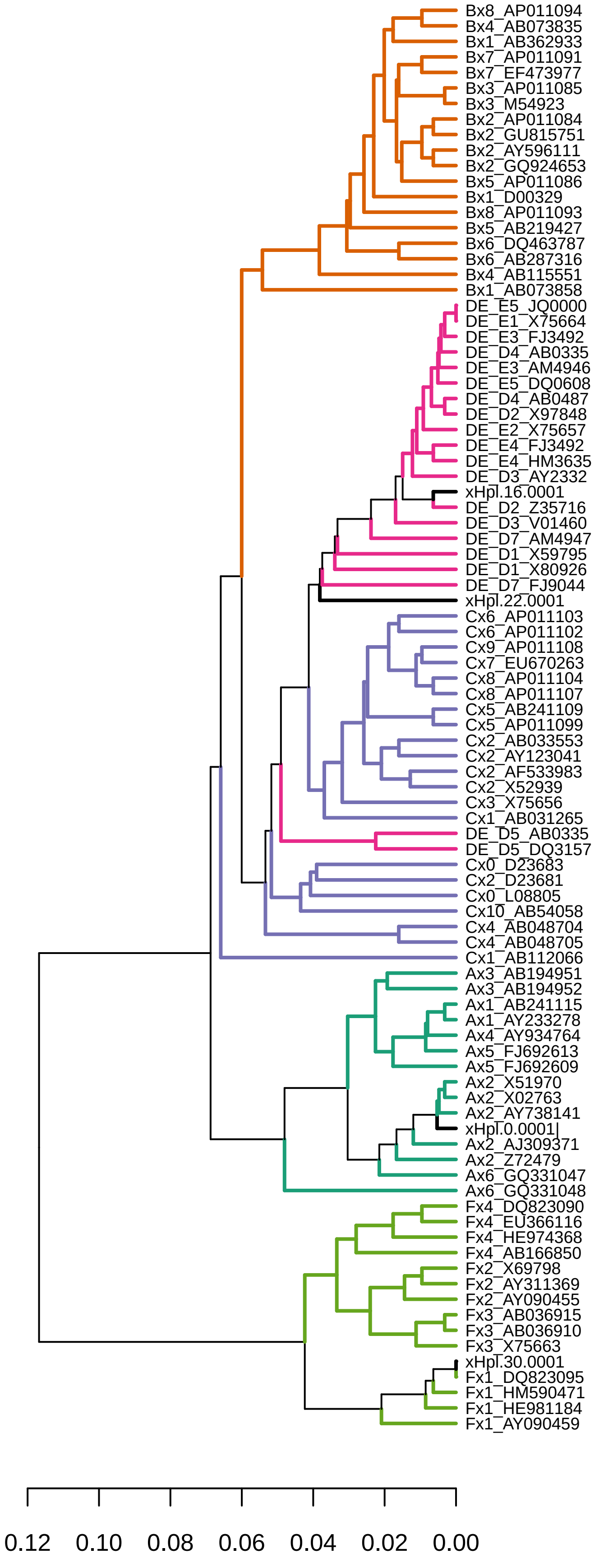

MDS map (K80): Pt05 Third sample X/preCore region

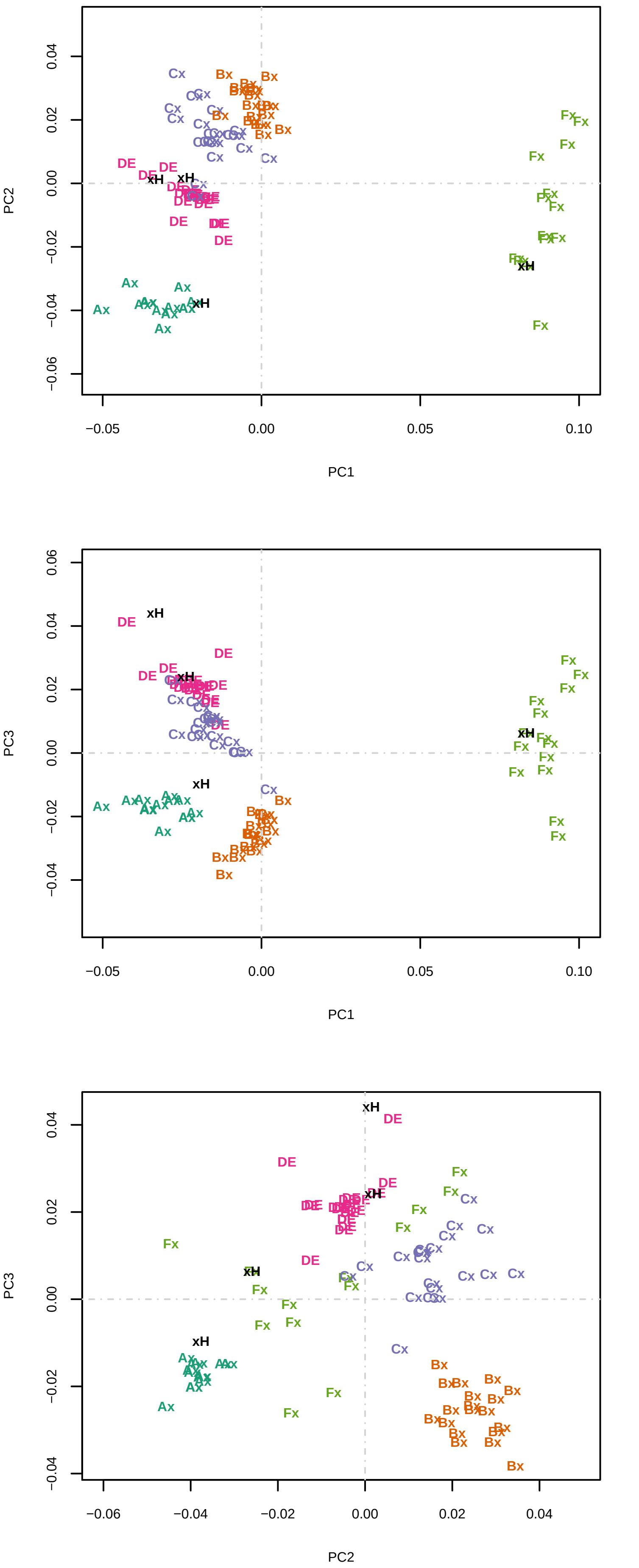

PGMA tree (K80): Pt06 First sample X/preCore region

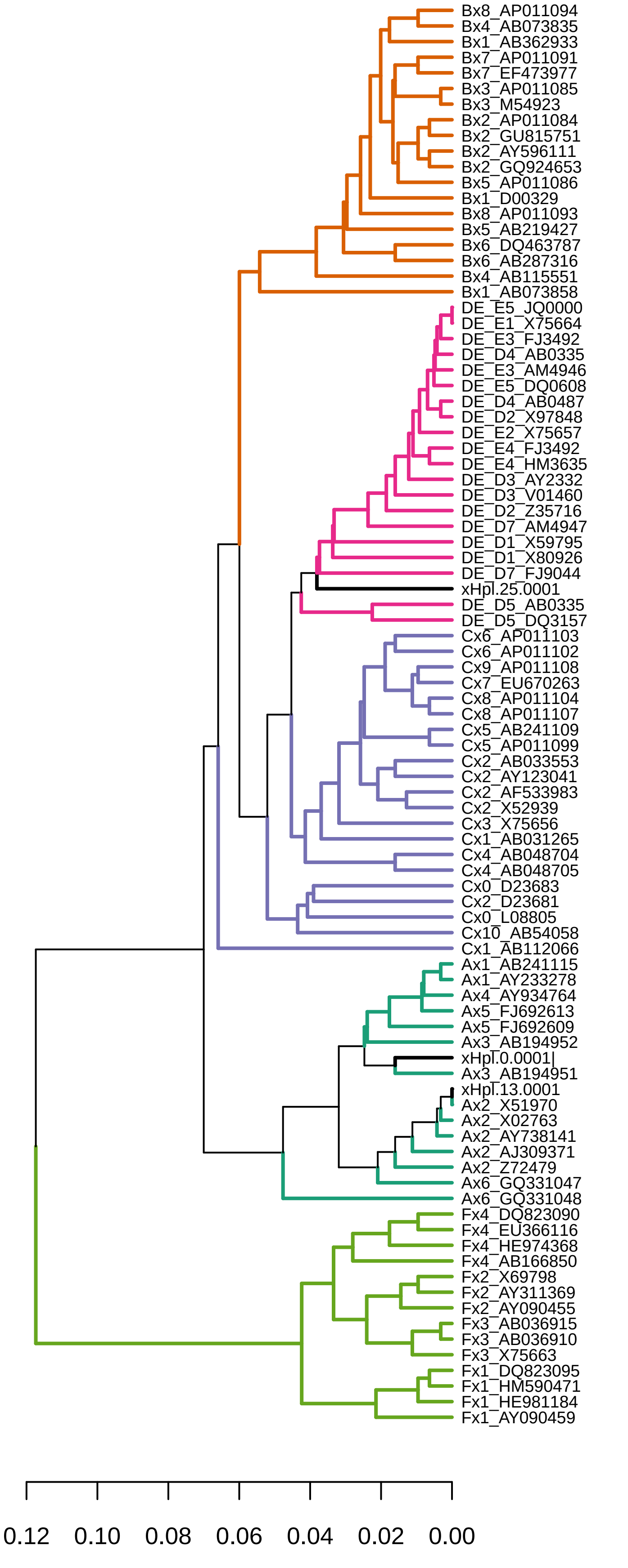

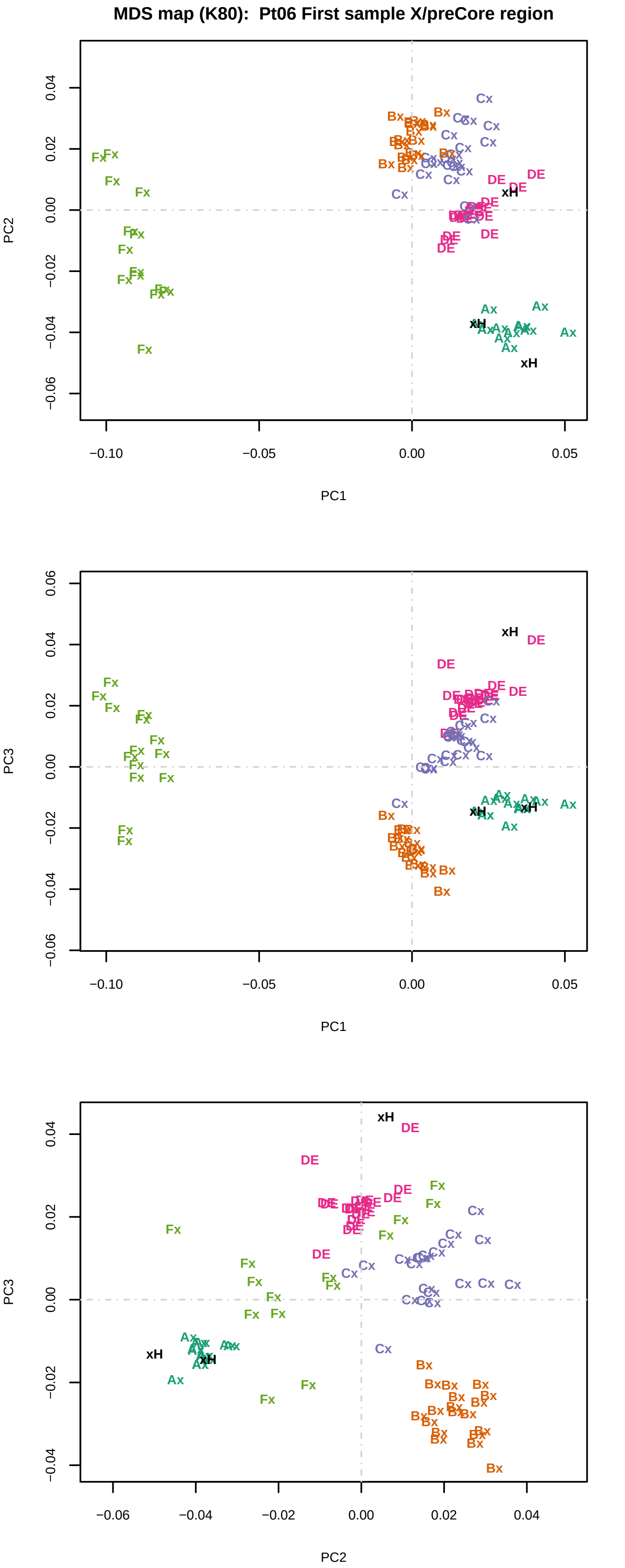

MDS map (K80): Pt06 First sample X/preCore region
PC1
PC2
PC3
Ax
Bx
Cx
DE
Fx
xH
−0.10
−0.05
0.00
0.05
−0.06
−0.04
−0.02
0.02
0.04
0.06

[illegible]GMA tree (K80): Pt06 Second sample X/preCore region

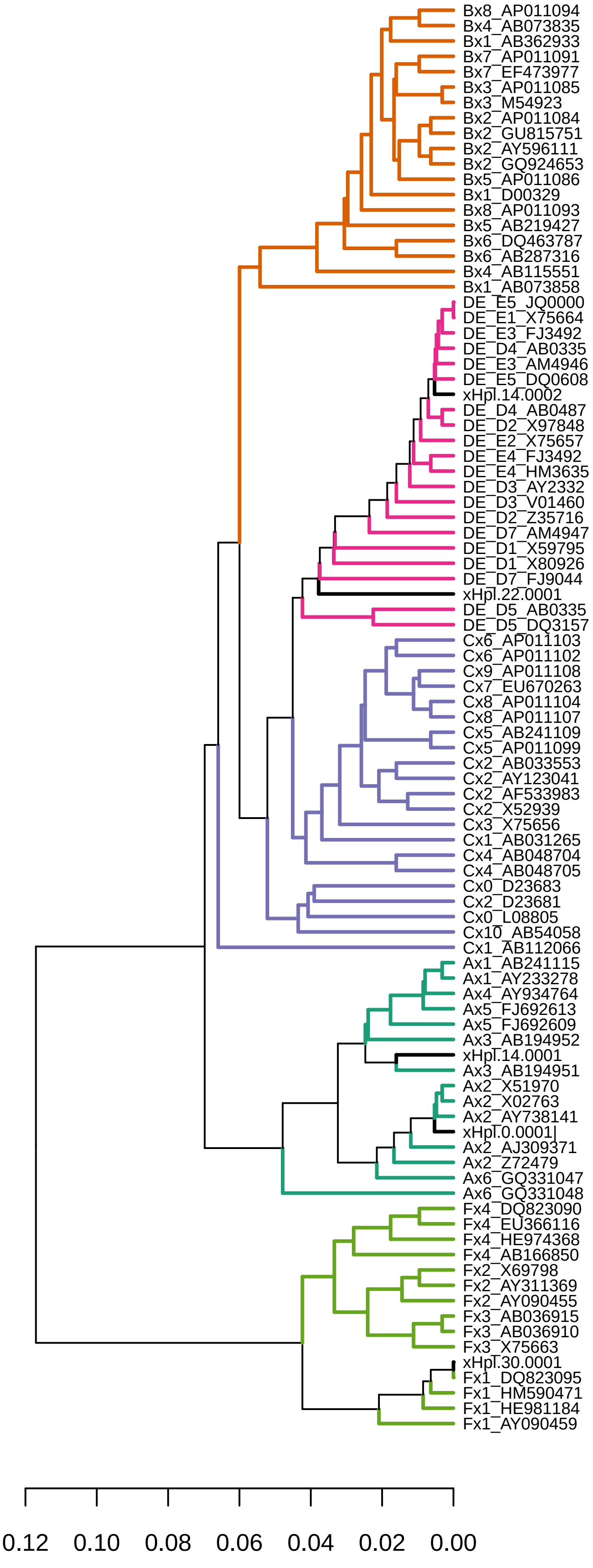

MDS map (K80): Pt06 Second sample X/preCore region

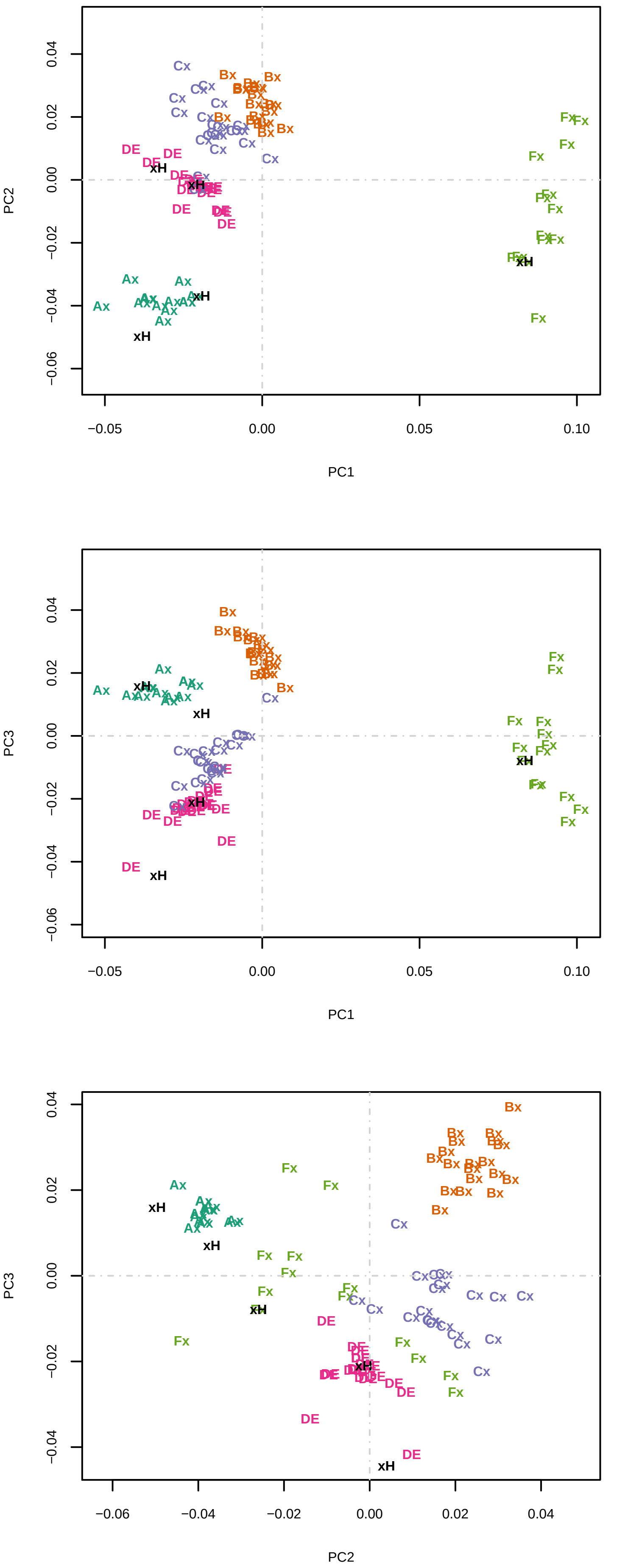

PGMA tree (K80): Pt06 Third sample X/preCore region

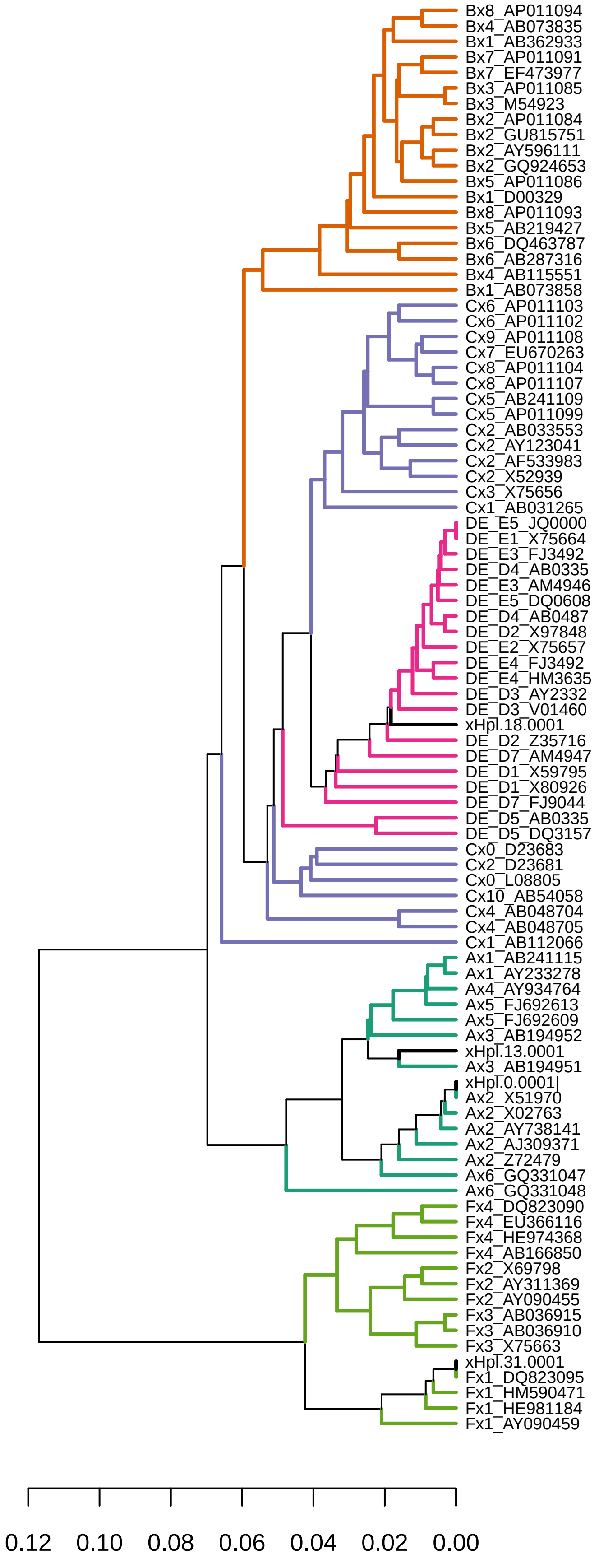

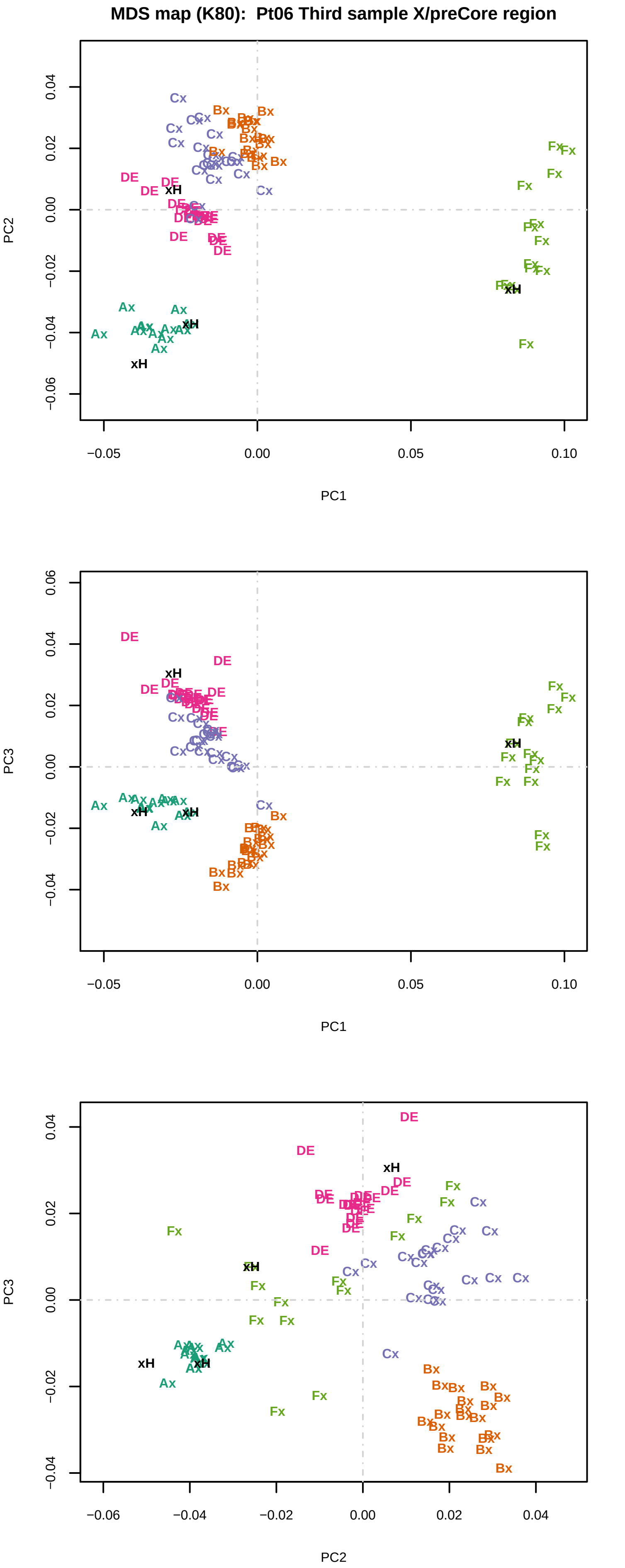
MDS map (K80): Pt06 Third sample X/preCore region
PC1
PC2
PC3
−0.05
0.00
0.05
0.10
−0.06
−0.04
−0.02
0.02
0.04
0.06
Ax
Bx
Cx
DE
Fx
xH

UPGMA tree (K80): Pt07 First sample X/preCore region

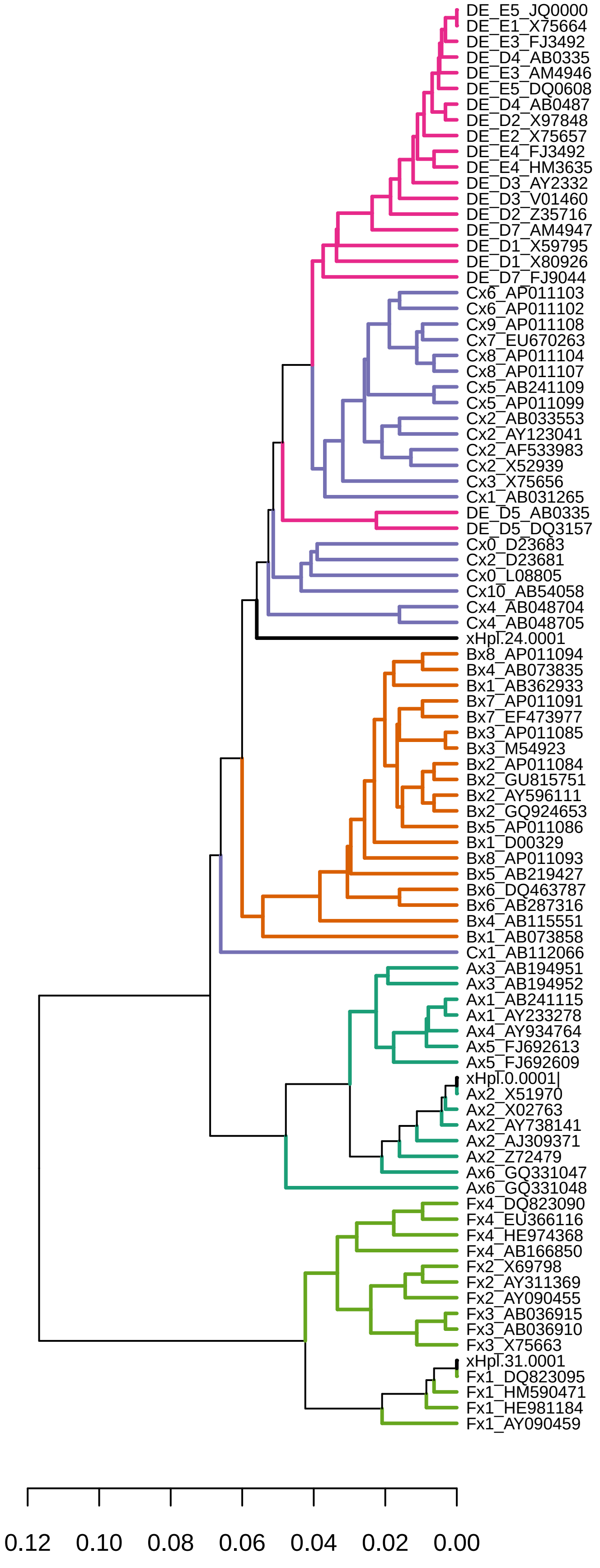

MDS map (K80): Pt07 First sample X/preCore region

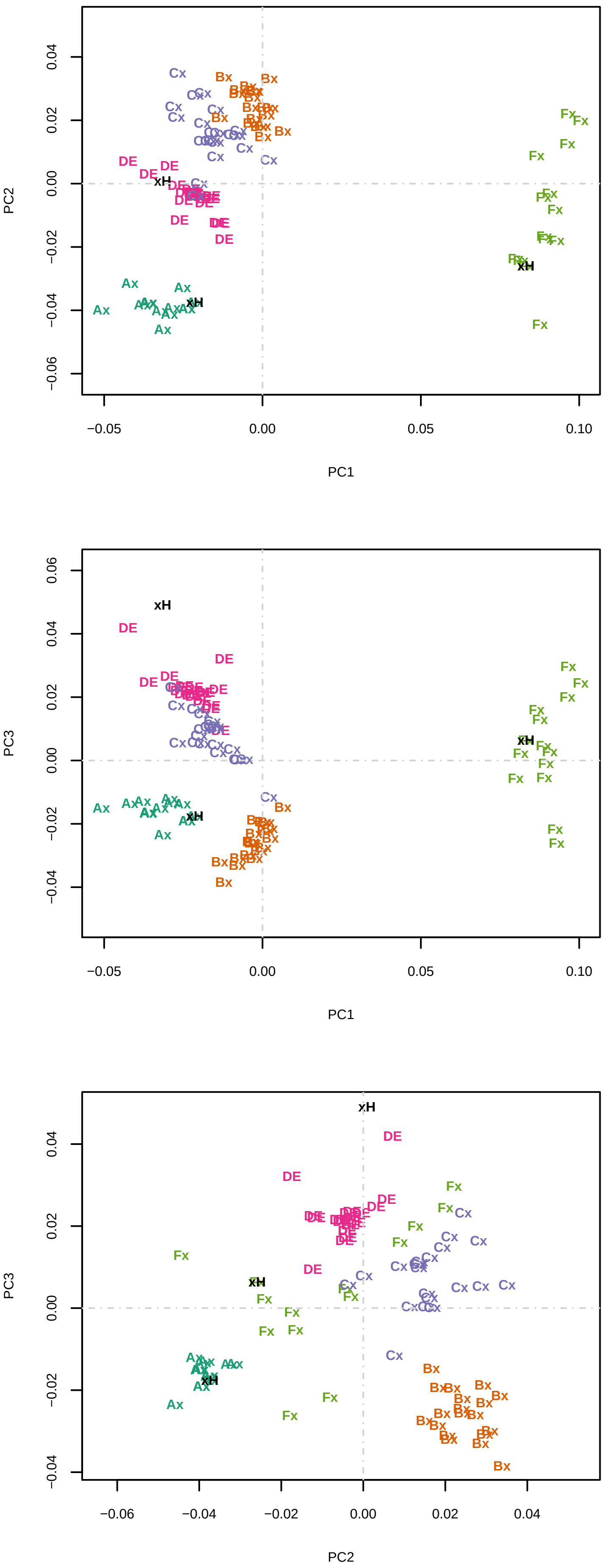

GMA tree (K80): Pt07 Second sample X/preCore region

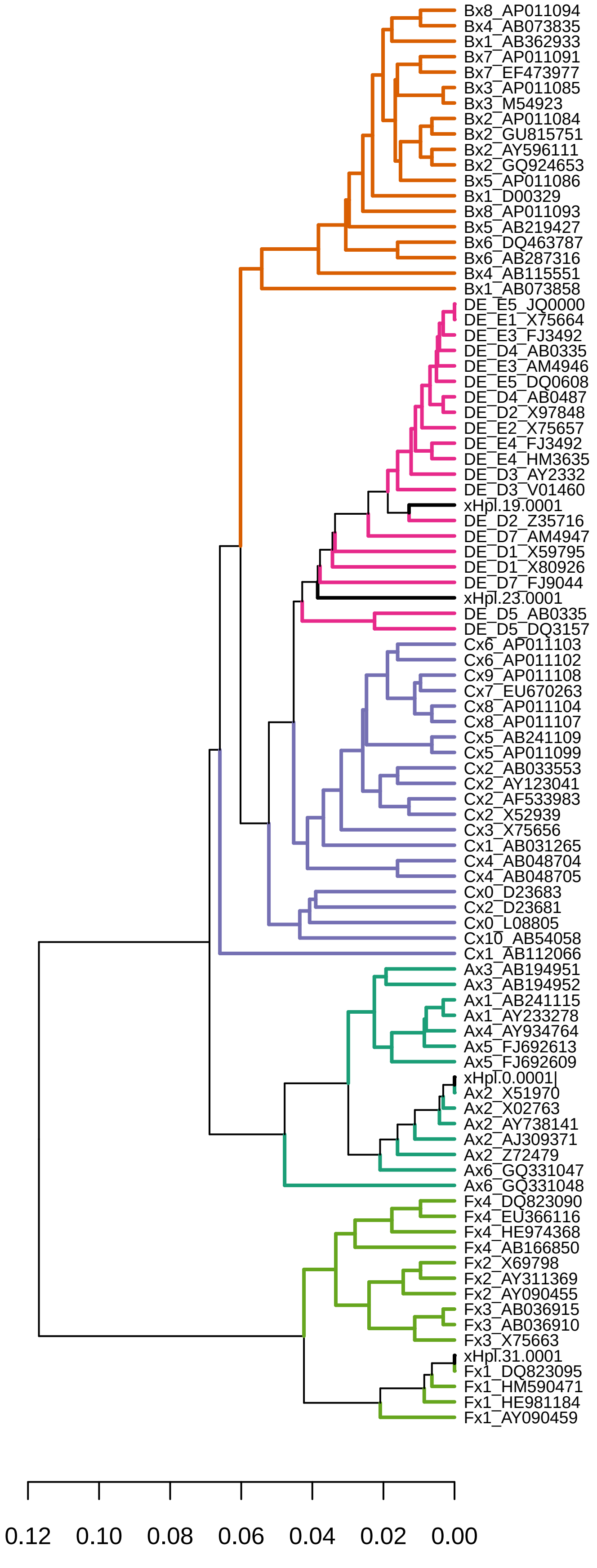

MDS map (K80): Pt07 Second sample X/preCore region

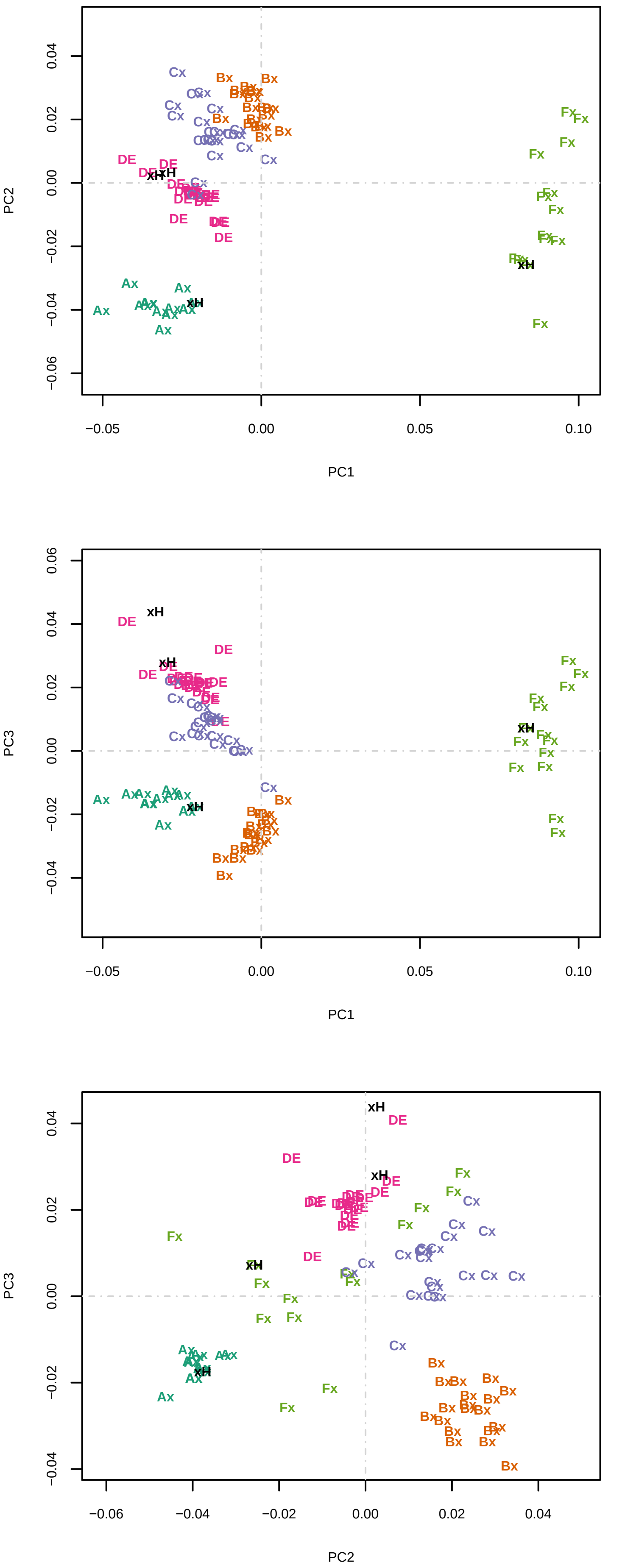

PGMA tree (K80): Pt07 Third sample X/preCore region

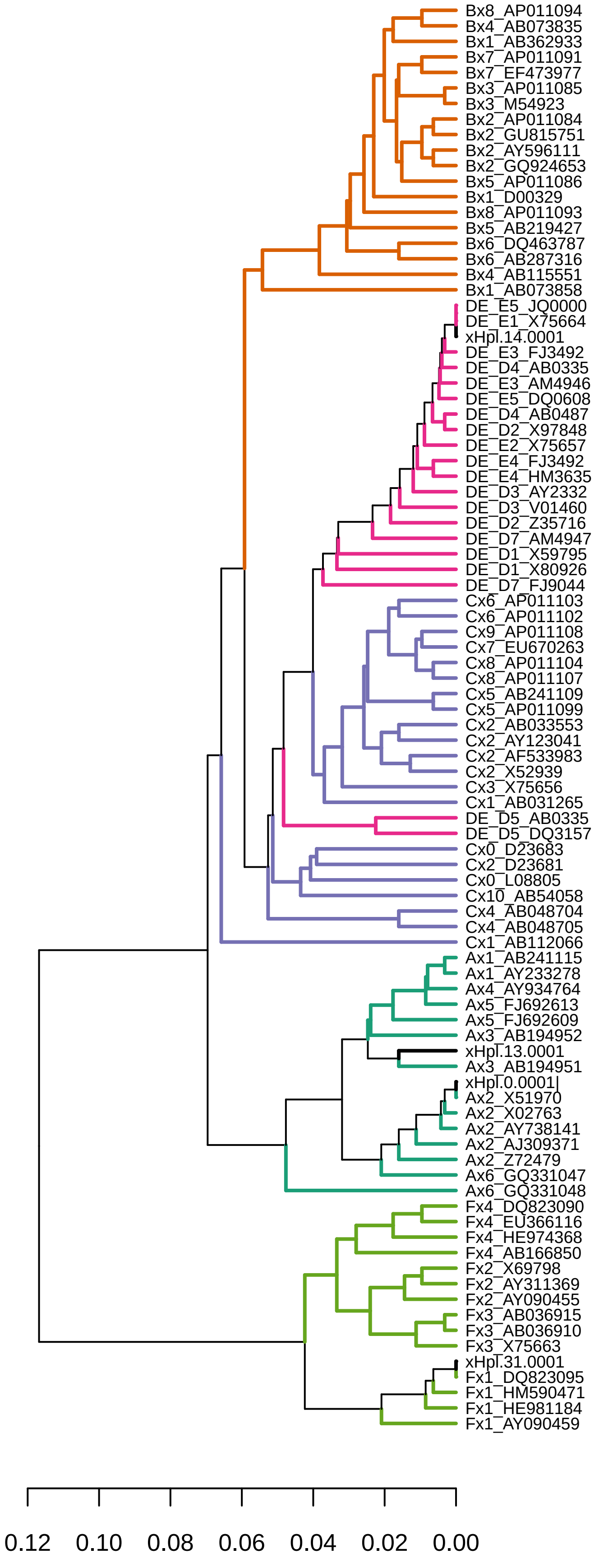

MDS map (K80): Pt07 Third sample X/preCore region

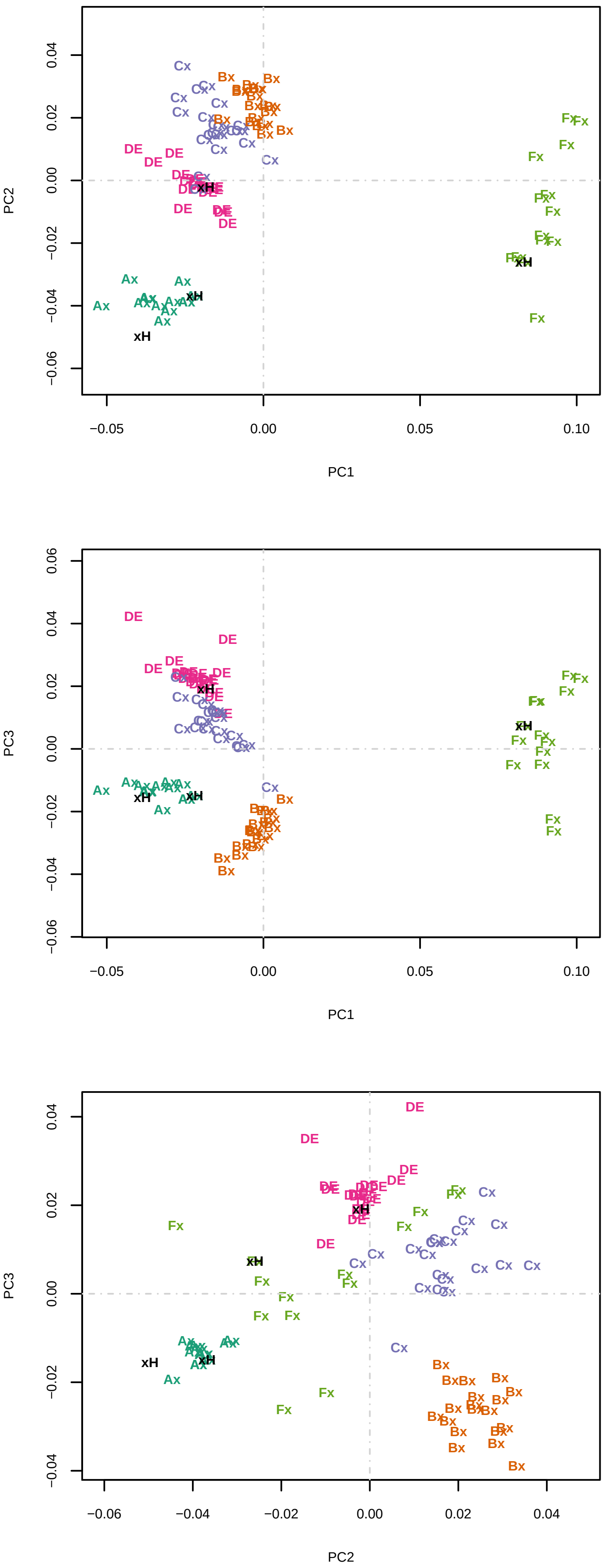

PGMA tree (K80): Pt08 First sample X/preCore region

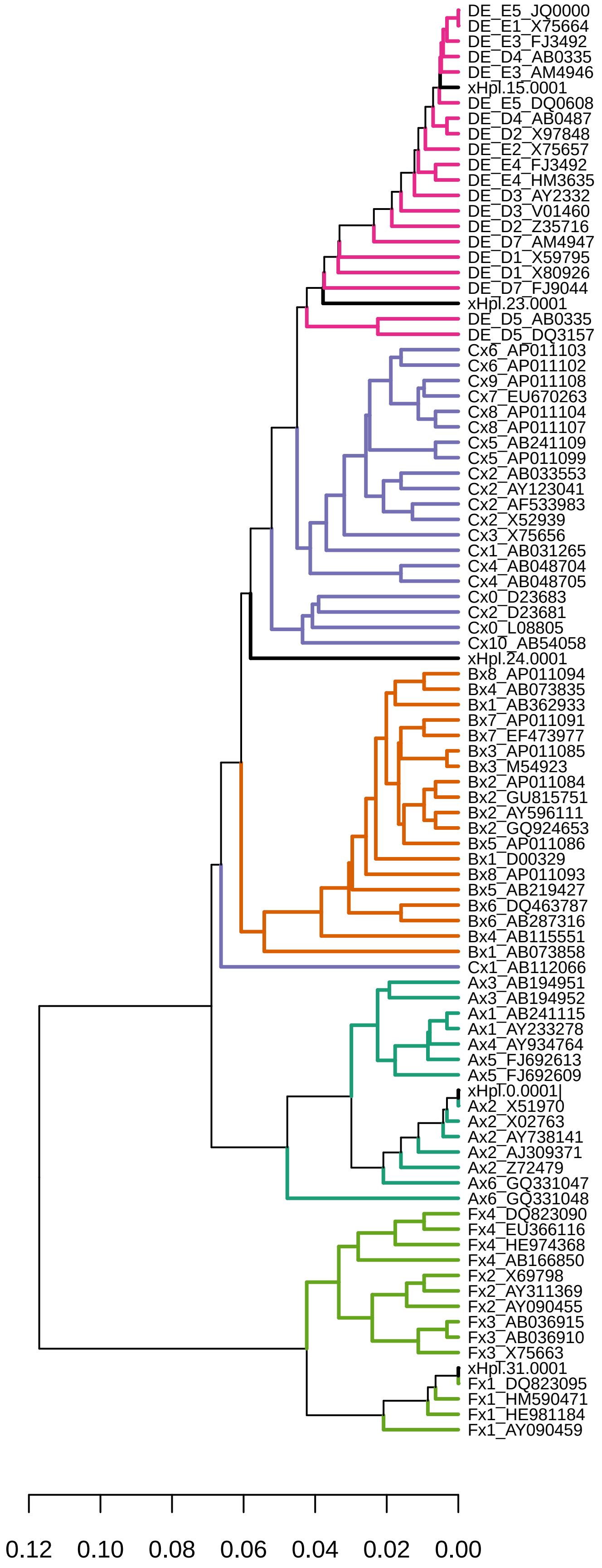

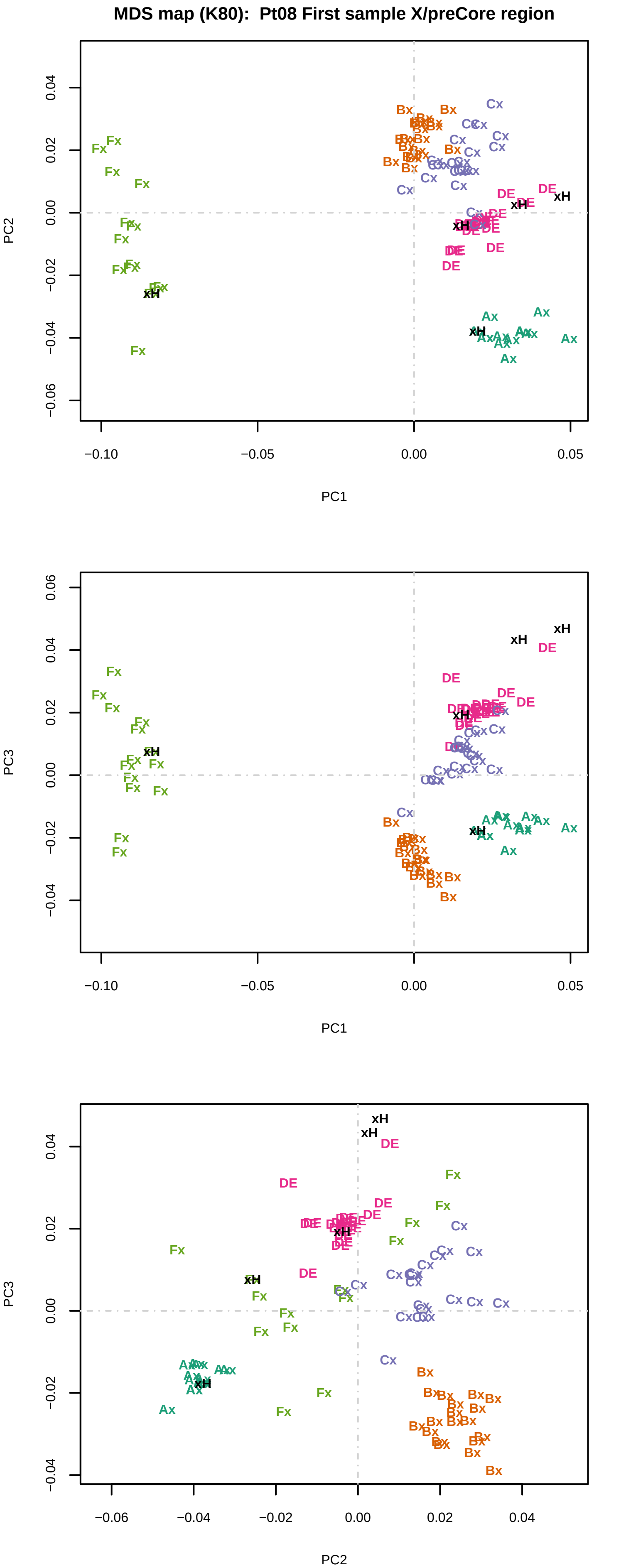

MDS map (K80): Pt08 First sample X/preCore region
PC1
PC2
PC3
−0.10
−0.05
0.00
0.05
0.04
0.02
0.00
−0.02
−0.04
−0.06
0.06
Fx
Bx
Cx
DE
Ax
xH

GMA tree (K80): Pt08 Second sample X/preCore region

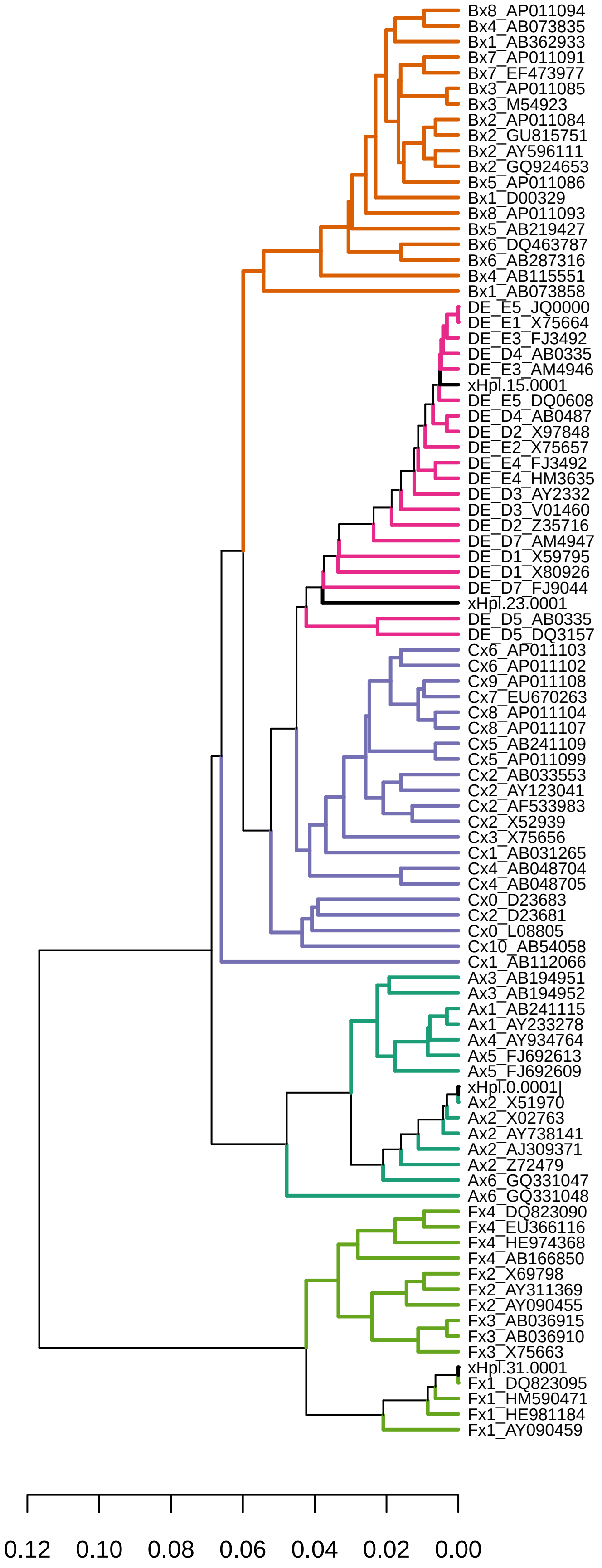

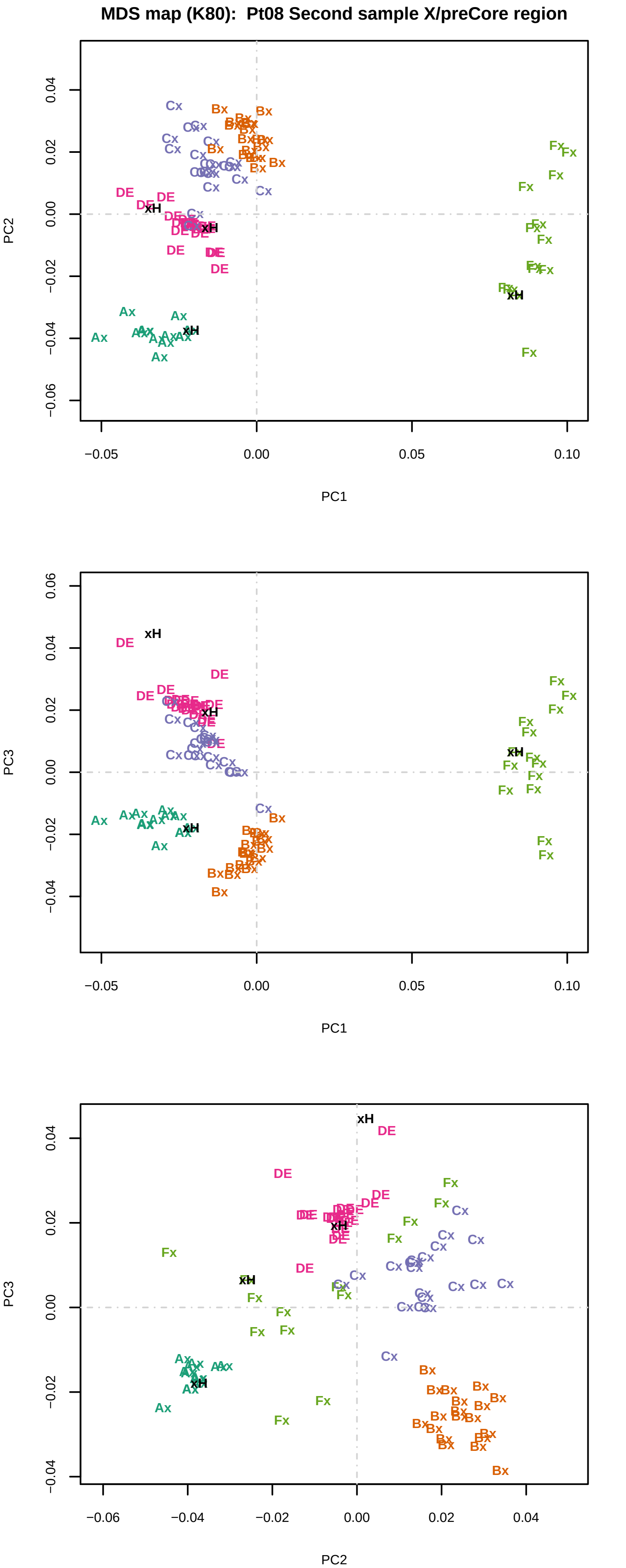
MDS map (K80): Pt08 Second sample X/preCore region
PC1
PC2
PC3
Ax
Bx
Cx
DE
Fx
xH

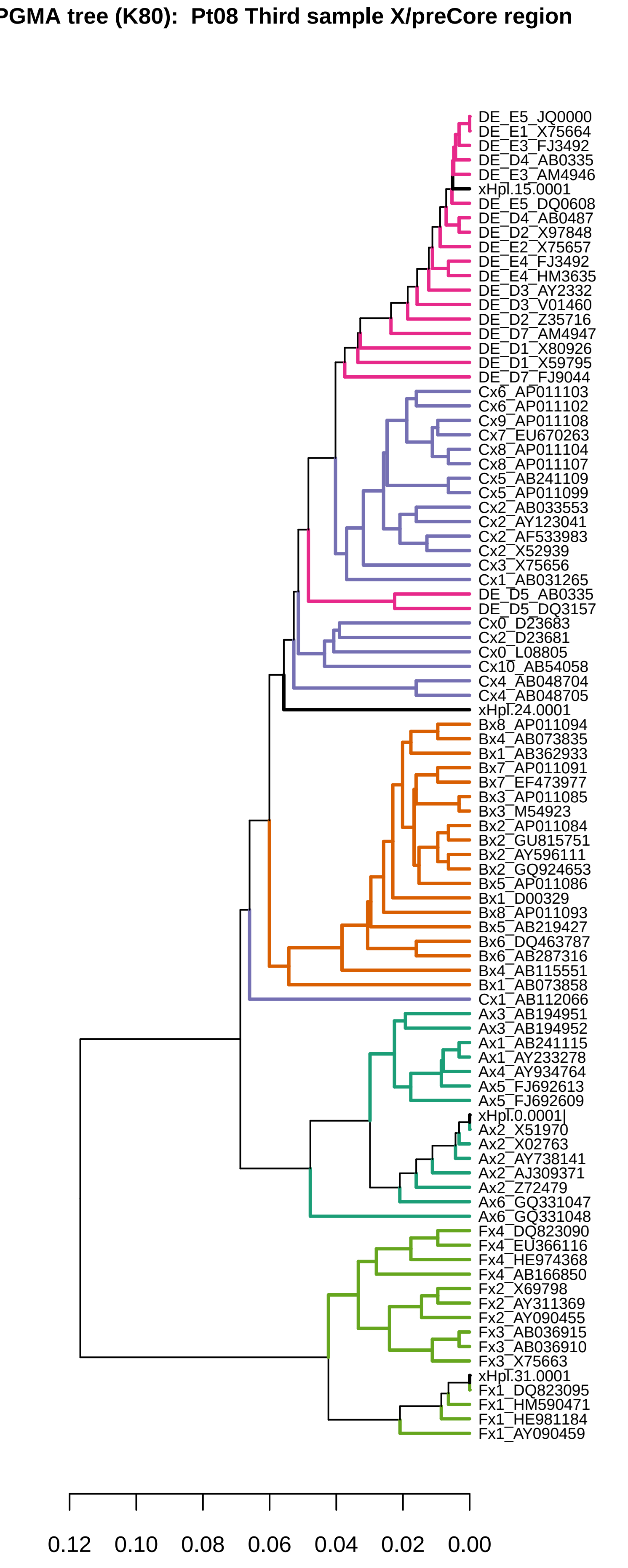
PGMA tree (K80): Pt08 Third sample X/preCore region
DE_E5_JQ0000
DE_E1_X75664
DE_E3_FJ3492
DE_D4_AB0335
DE_E3_AM4946
xHpl.15.0001
DE_E5_DQ0608
DE_D4_AB0487
DE_D2_X97848
DE_E2_X75657
DE_E4_FJ3492
DE_E4_HM3635
DE_D3_AY2332
DE_D3_V01460
DE_D2_Z35716
DE_D7_AM4947
DE_D1_X80926
DE_D1_X59795
DE_D7_FJ9044
Cx6_AP011103
Cx6_AP011102
Cx9_AP011108
Cx7_EU670263
Cx8_AP011104
Cx8_AP011107
Cx5_AB241109
Cx5_AP011099
Cx2_AB033553
Cx2_AY123041
Cx2_AF533983
Cx2_X52939
Cx3_X75656
Cx1_AB031265
DE_D5_AB0335
DE_D5_DQ3157
Cx0_D23683
Cx2_D23681
Cx0_L08805
Cx10_AB54058
Cx4_AB048704
Cx4_AB048705
xHpl.24.0001
Bx8_AP011094
Bx4_AB073835
Bx1_AB362933
Bx7_AP011091
Bx7_EF473977
Bx3_AP011085
Bx3_M54923
Bx2_AP011084
Bx2_GU815751
Bx2_AY596111
Bx2_GQ924653
Bx5_AP011086
Bx1_D00329
Bx8_AP011093
Bx5_AB219427
Bx6_DQ463787
Bx6_AB287316
Bx4_AB115551
Bx1_AB073858
Cx1_AB112066
Ax3_AB194951
Ax3_AB194952
Ax1_AB241115
Ax1_AY233278
Ax4_AY934764
Ax5_FJ692613
Ax5_FJ692609
xHpl.0.0001
Ax2_X51970
Ax2_X02763
Ax2_AY738141
Ax2_AJ309371
Ax2_Z72479
Ax6_GQ331047
Ax6_GQ331048
Fx4_DQ823090
Fx4_EU366116
Fx4_HE974368
Fx4_AB166850
Fx2_X69798
Fx2_AY311369
Fx2_AY090455
Fx3_AB036915
Fx3_AB036910
Fx3_X75663
xHpl.31.0001
Fx1_DQ823095
Fx1_HM590471
Fx1_HE981184
Fx1_AY090459
0.12
0.10
0.08
0.06
0.04
0.02
0.00

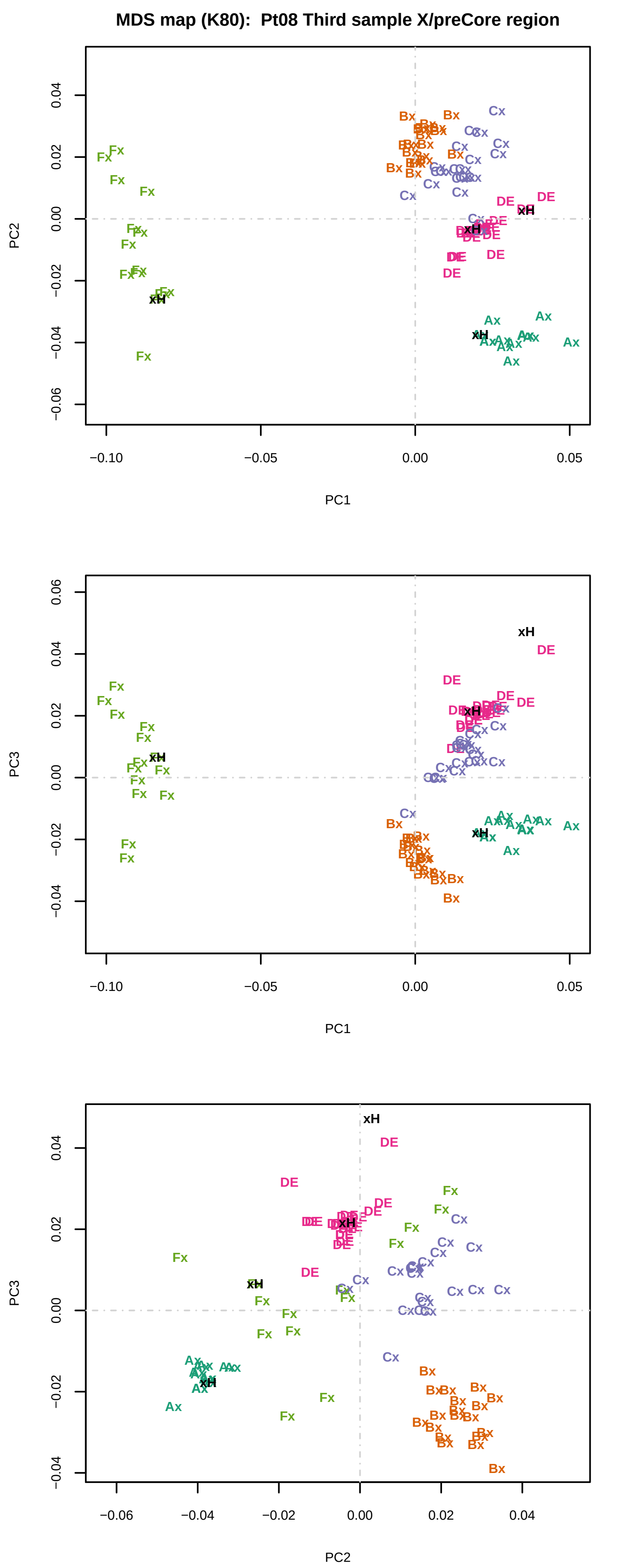
MDS map (K80): Pt08 Third sample X/preCore region
PC1
PC2
PC3
−0.10
−0.05
0.00
0.05
−0.06
−0.04
−0.02
0.02
0.04
0.06
Ax
Bx
Cx
DE
Fx
xH

PGMA tree (K80): Pt09 First sample X/preCore region

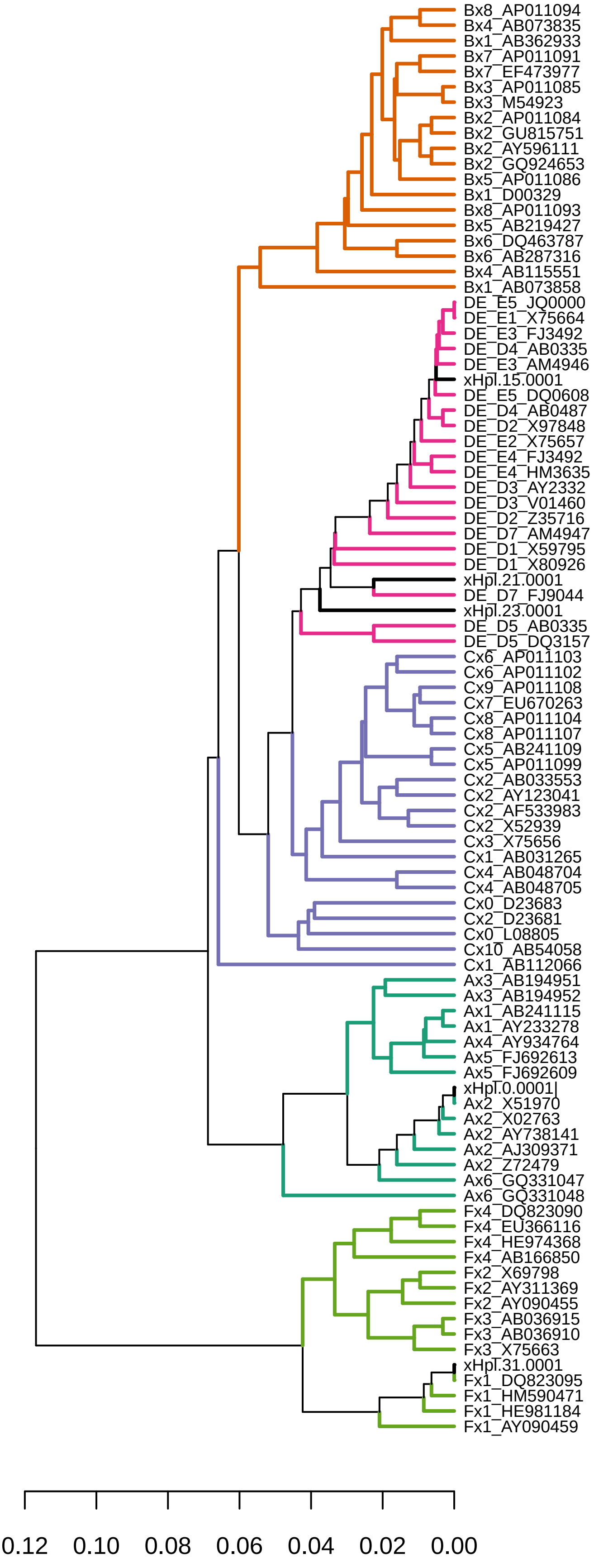

MDS map (K80): Pt09 First sample X/preCore region

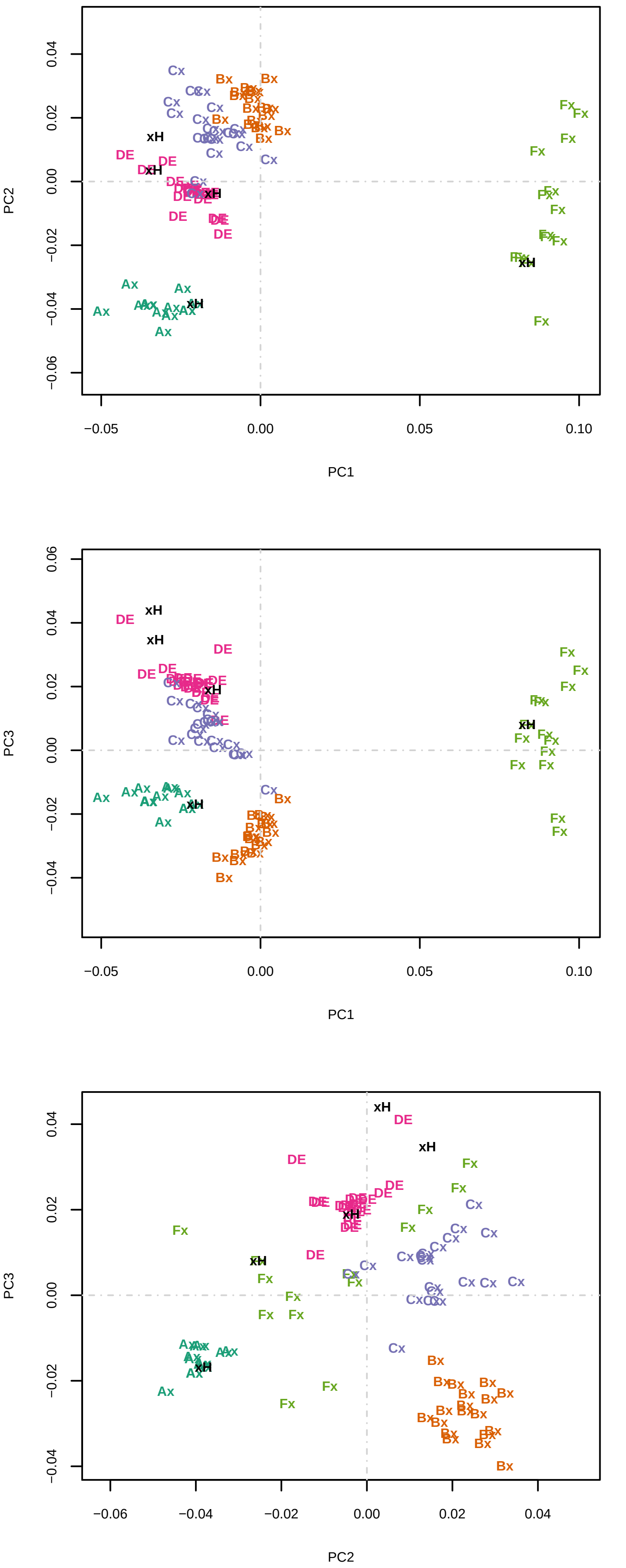

GMA tree (K80): Pt09 Second sample X/preCore region

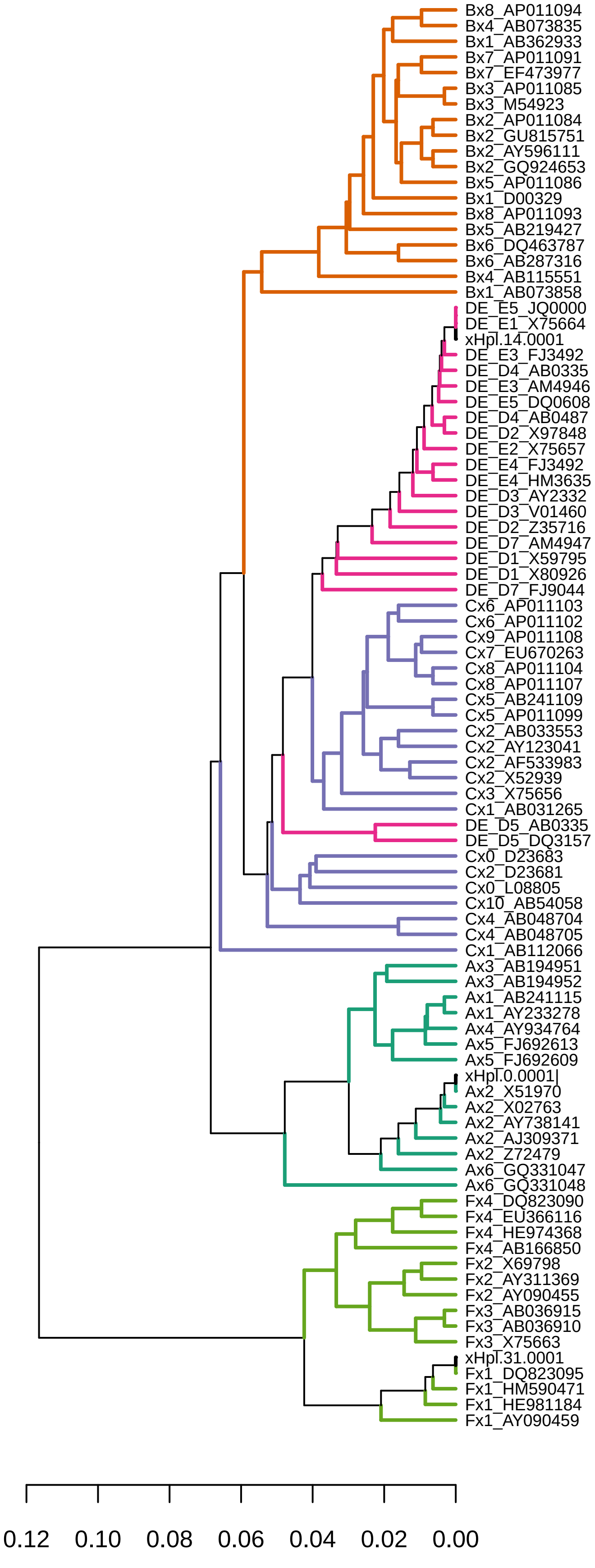

MDS map (K80): Pt09 Second sample X/preCore region

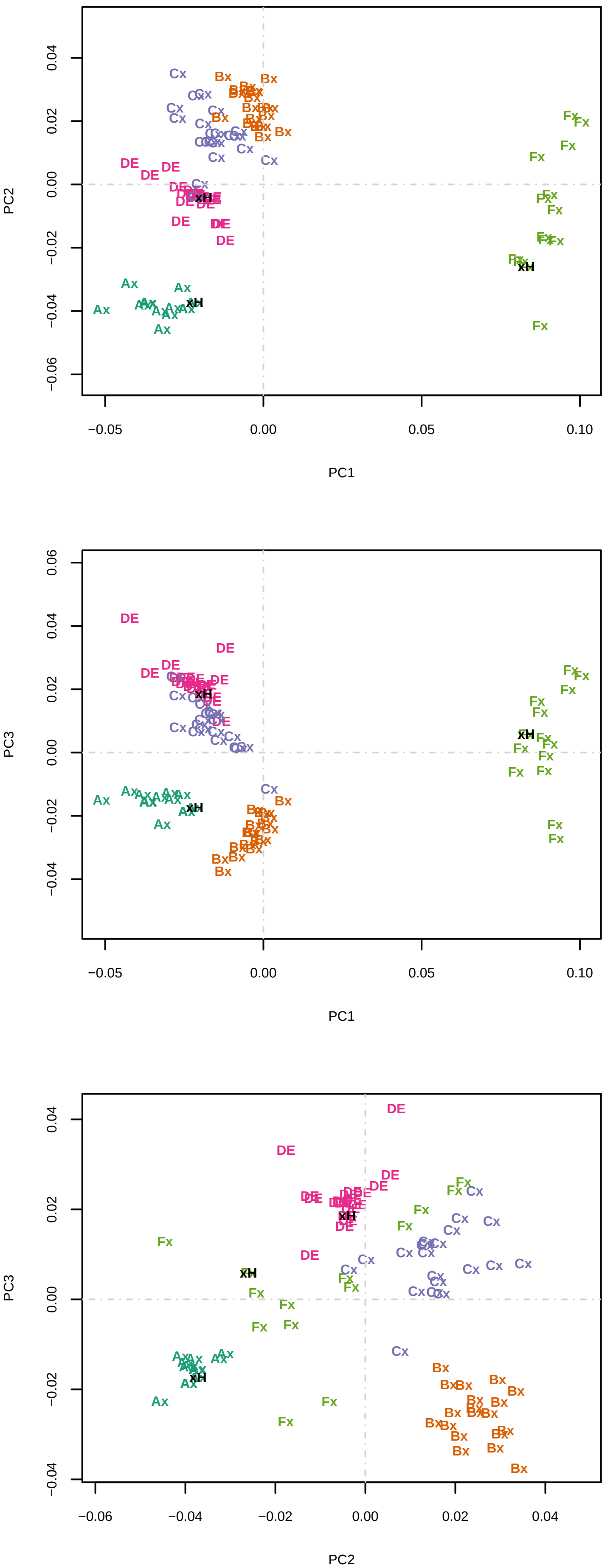

PGMA tree (K80): Pt09 Third sample X/preCore region

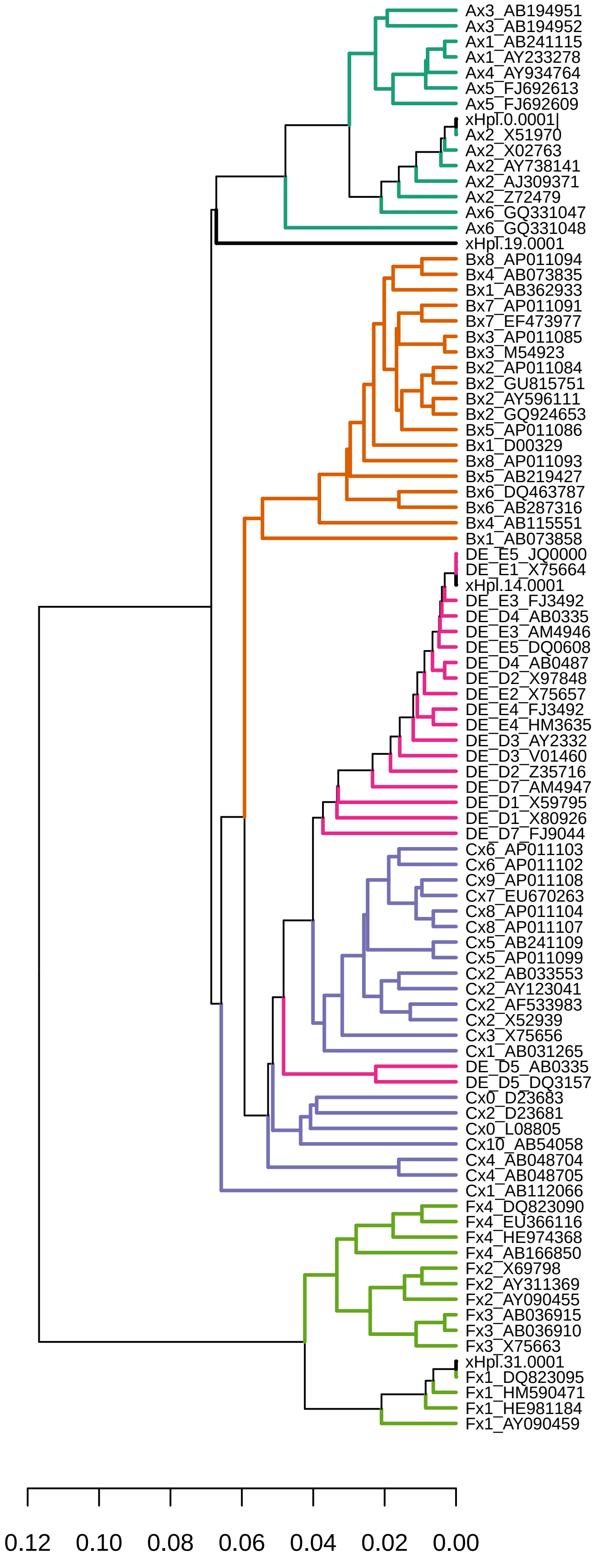

MDS map (K80): Pt09 Third sample X/preCore region

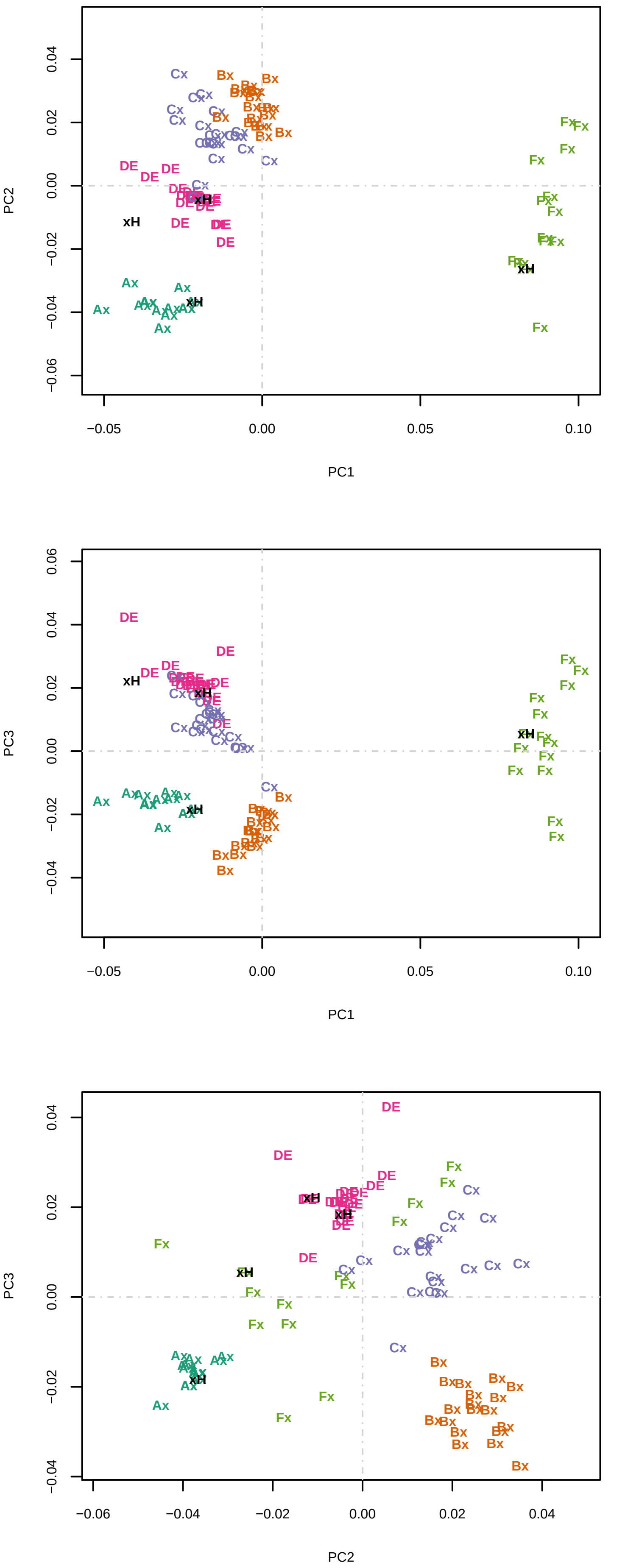

PGMA tree (K80): Pt10 First sample X/preCore region

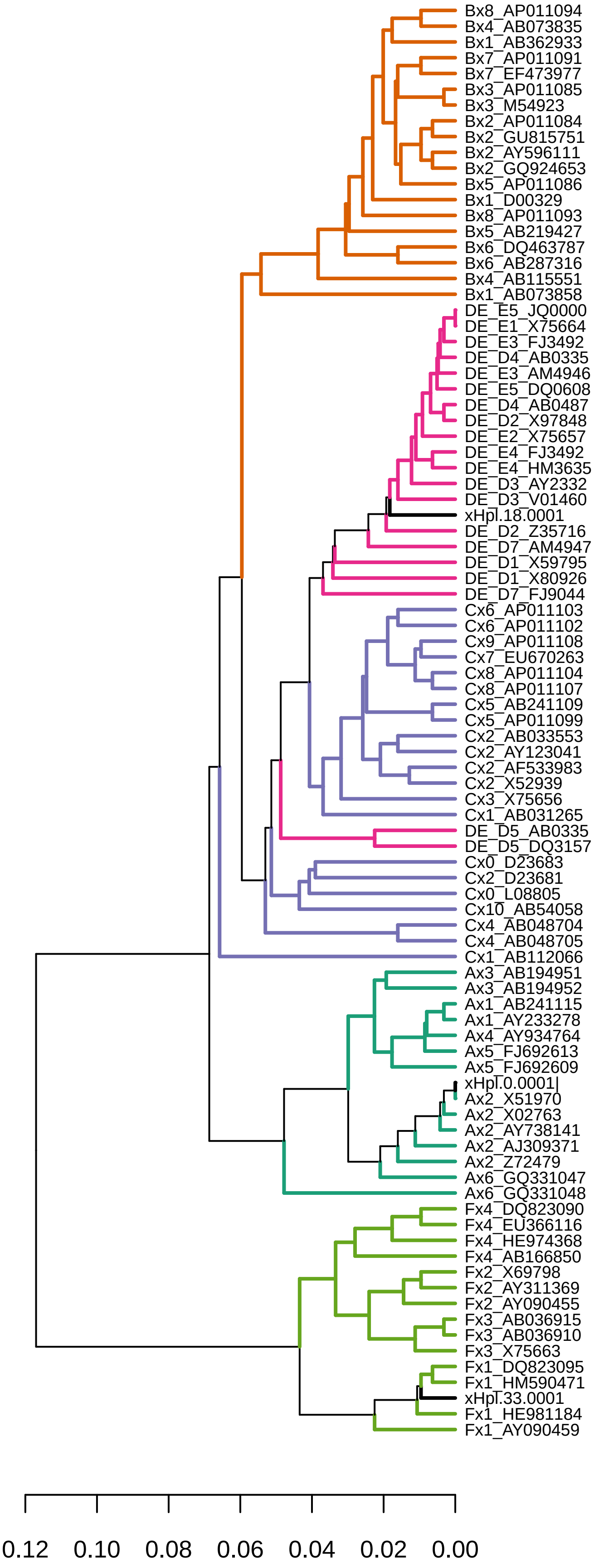

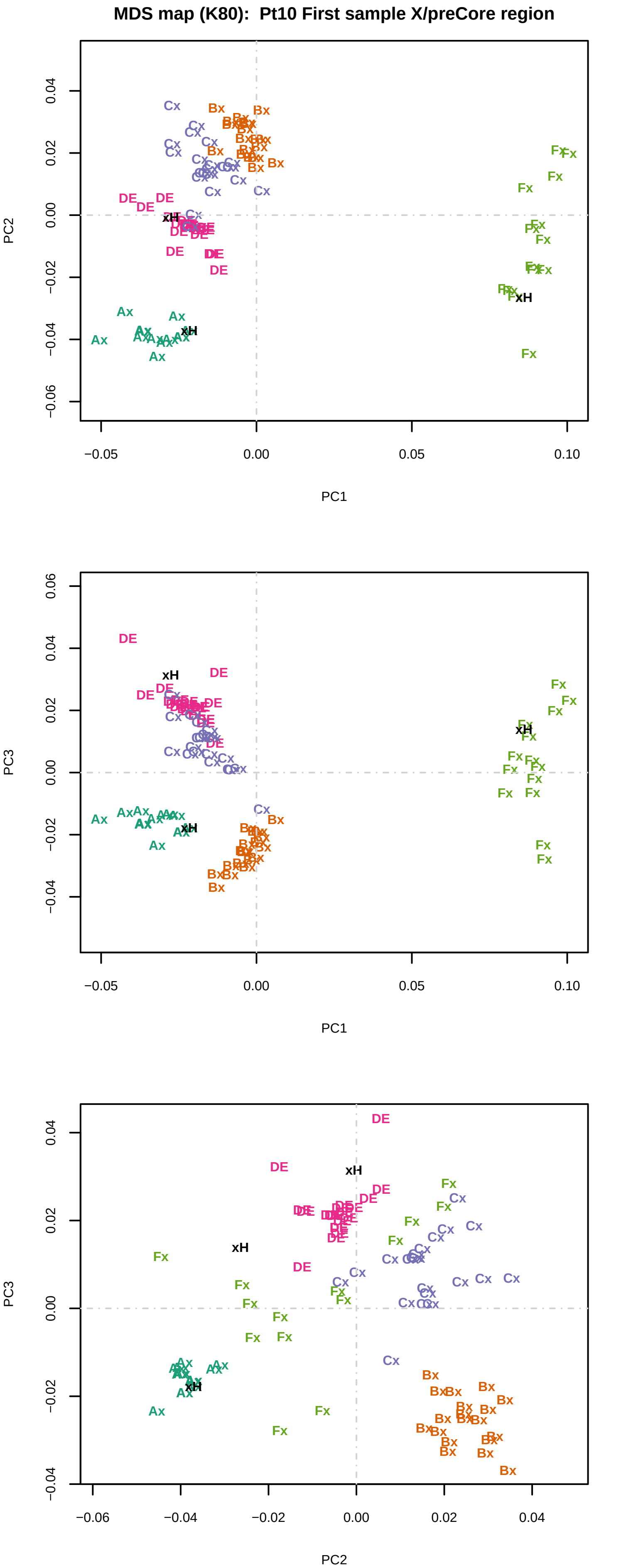

MDS map (K80): Pt10 First sample X/preCore region
PC1
PC2
PC3
Ax
Bx
Cx
DE
Fx
xH
−0.05
0.00
0.05
0.10
0.04
0.02
0.00
−0.02
−0.04
−0.06
0.06
0.04
0.02
0.00
−0.02
−0.04
−0.06
−0.04
−0.02
0.00
0.02
0.04

GMA tree (K80): Pt10 Second sample X/preCore region

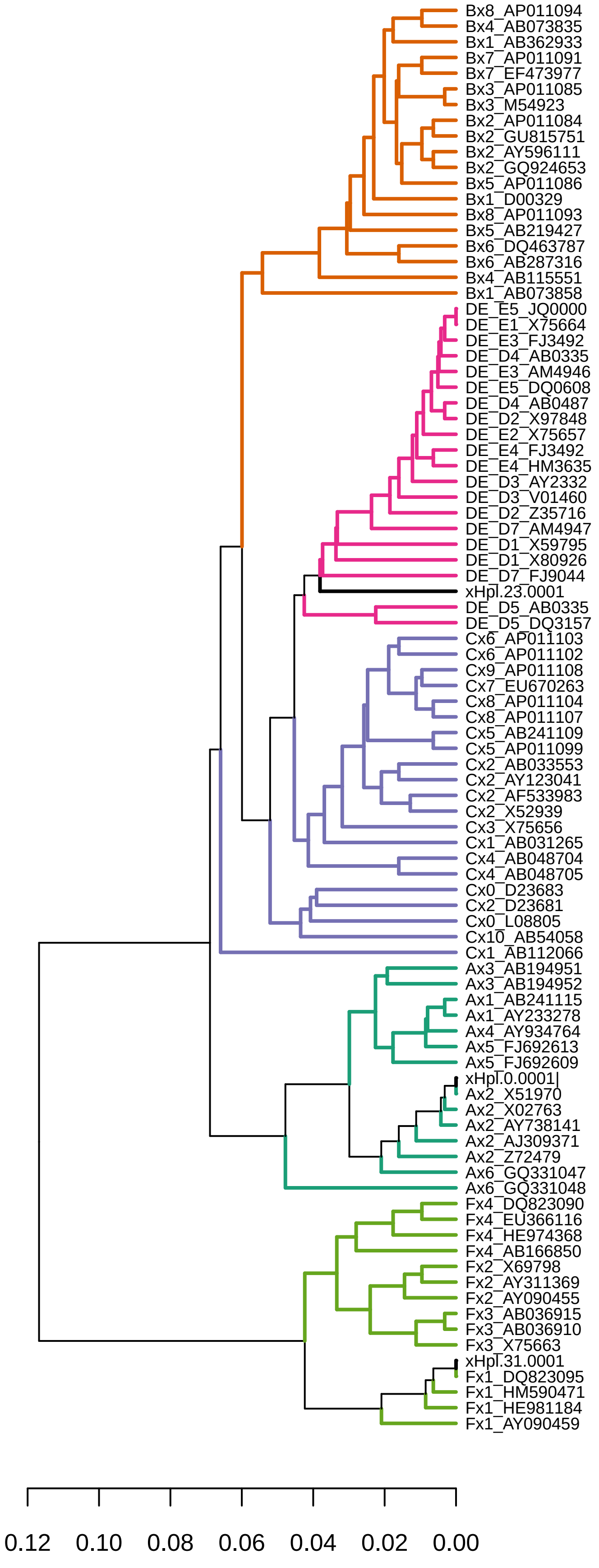

MDS map (K80): Pt10 Second sample X/preCore region

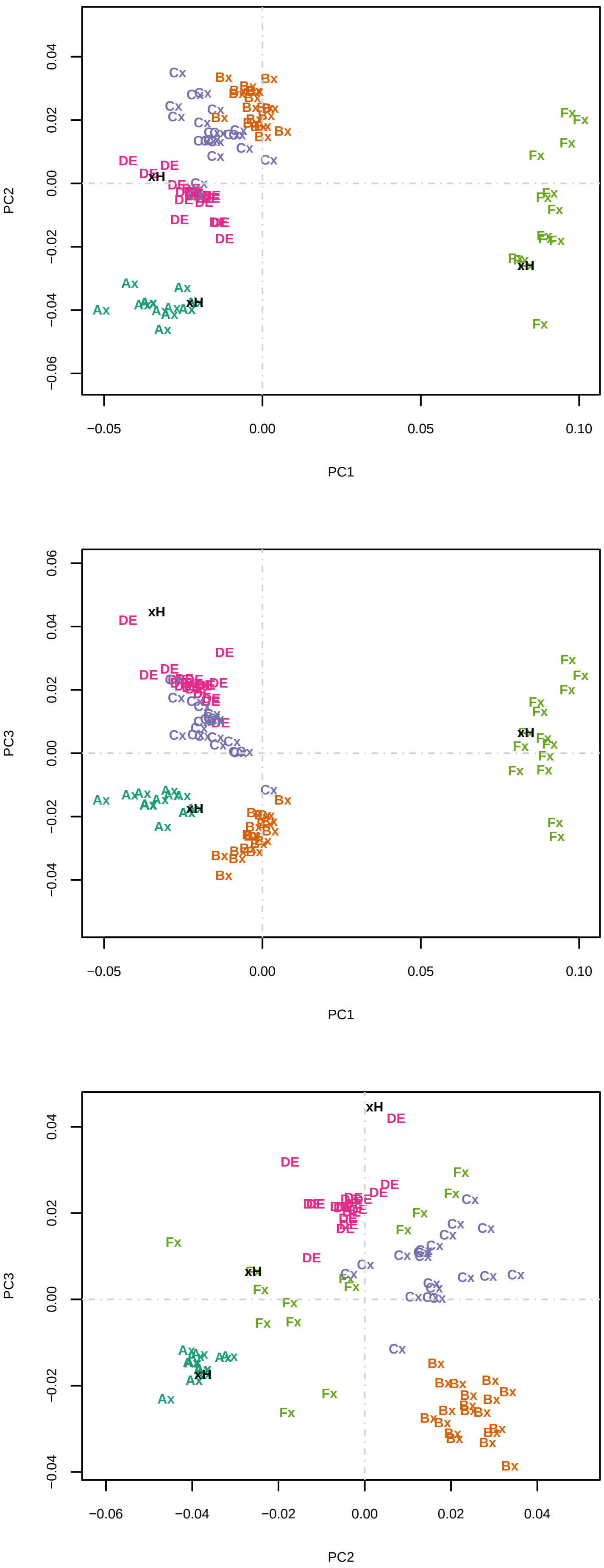

PGMA tree (K80): Pt10 Third sample X/preCore region

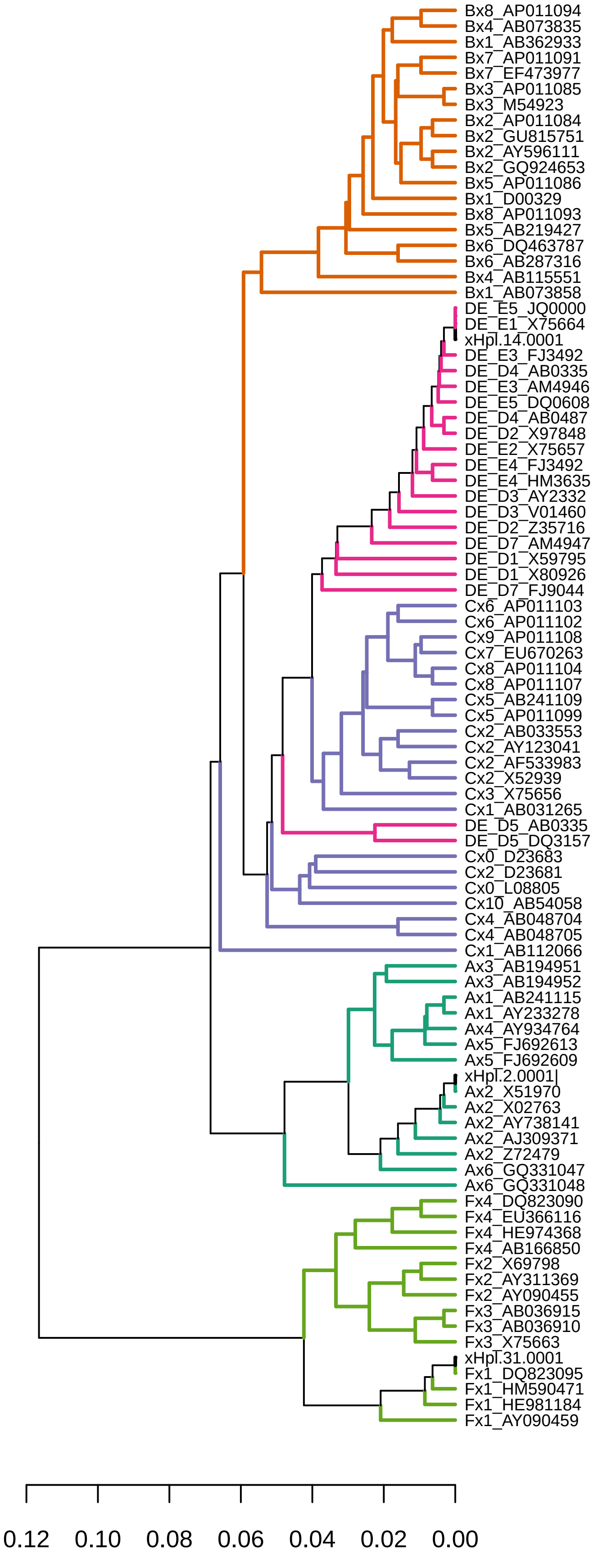

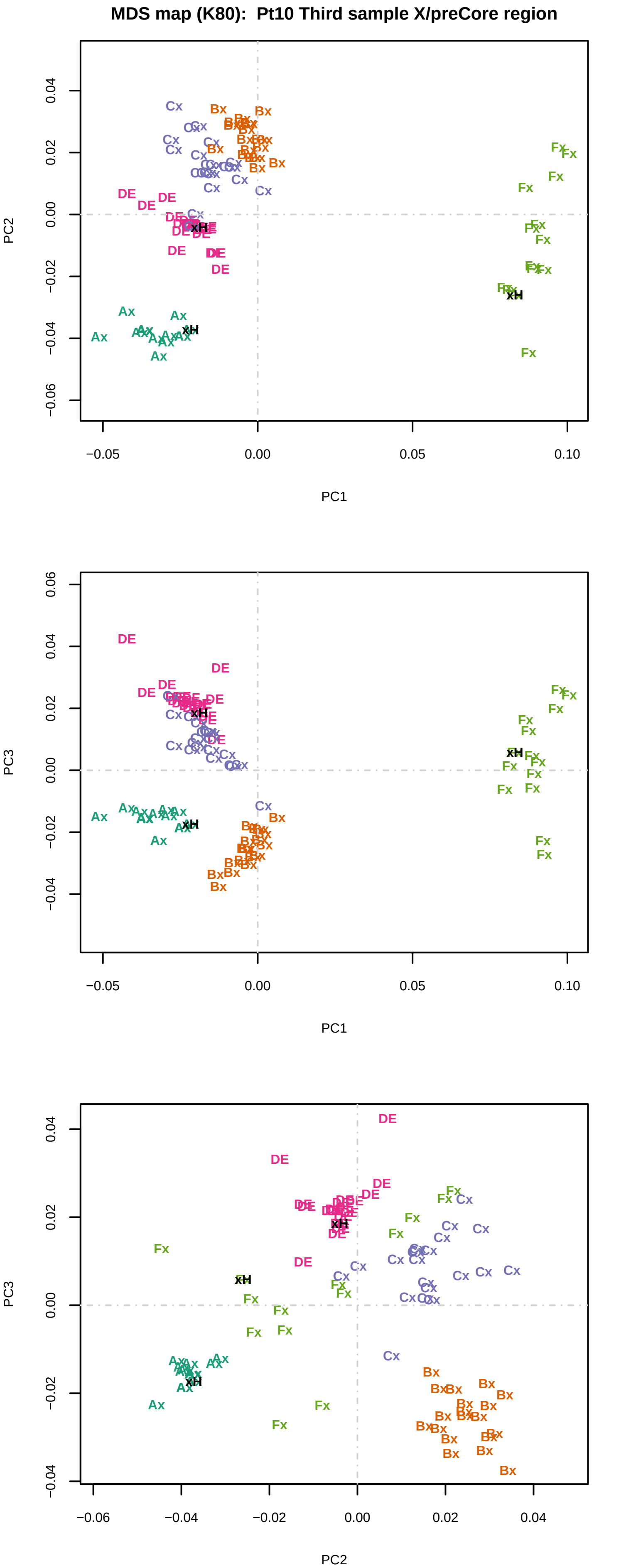
MDS map (K80): Pt10 Third sample X/preCore region
PC1
PC2
PC3
