# Supplementary material for: Complex Genotype Mixtures Analyzed by Deep Sequencing in Two Different Regions of Hepatitis B Virus
Source: PLoS One. 2015 Dec 29;10(12):e0144816. doi: 10.1371/journal.pone.0144816 (PMC4695080; doi:10.1371/journal.pone.0144816)
Supplement: S6 File — (PDF) [file pone.0144816.s006.pdf]

UPGMA tree (N): 1−400

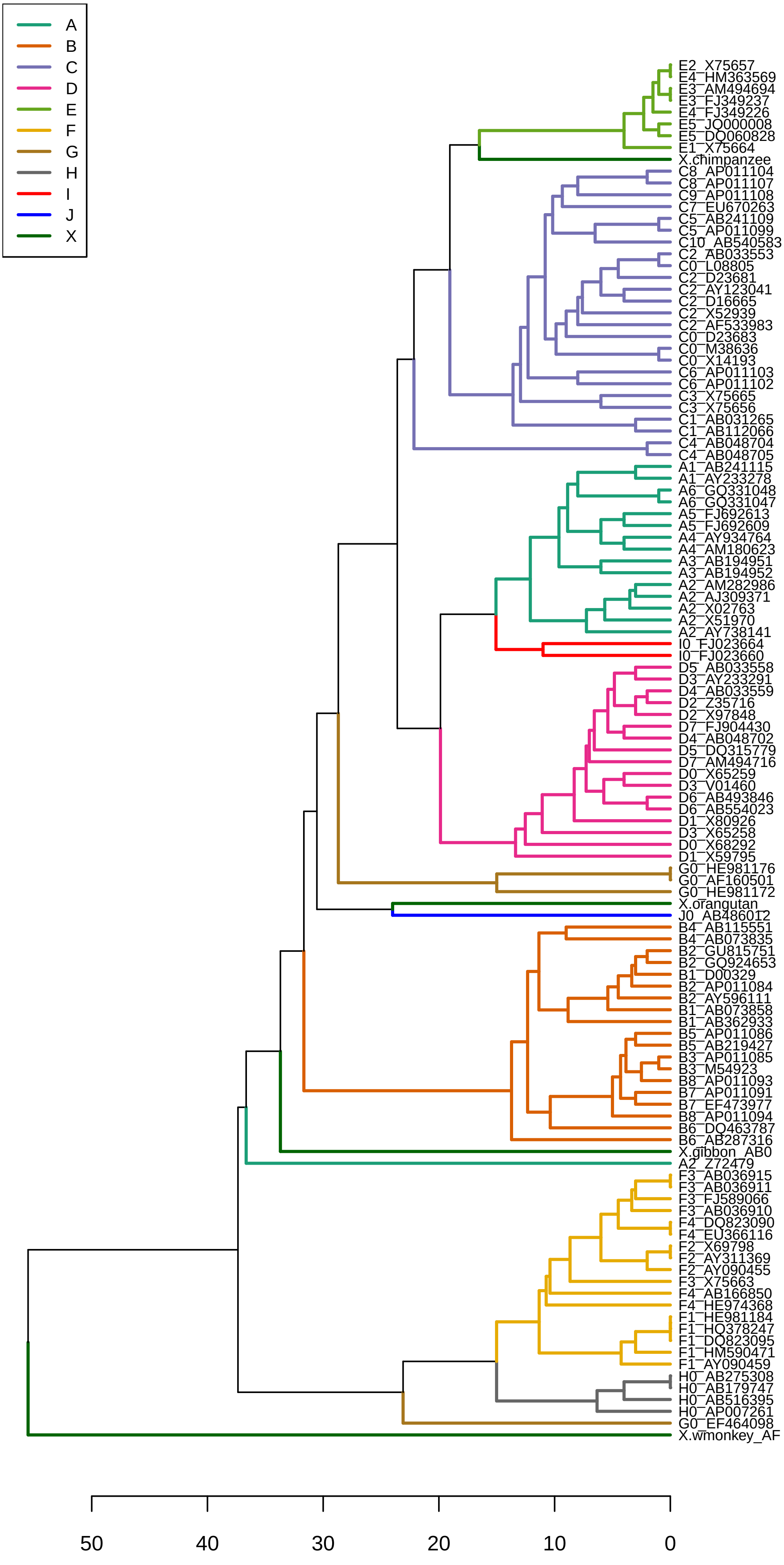

UPGMA tree (N): 41−440

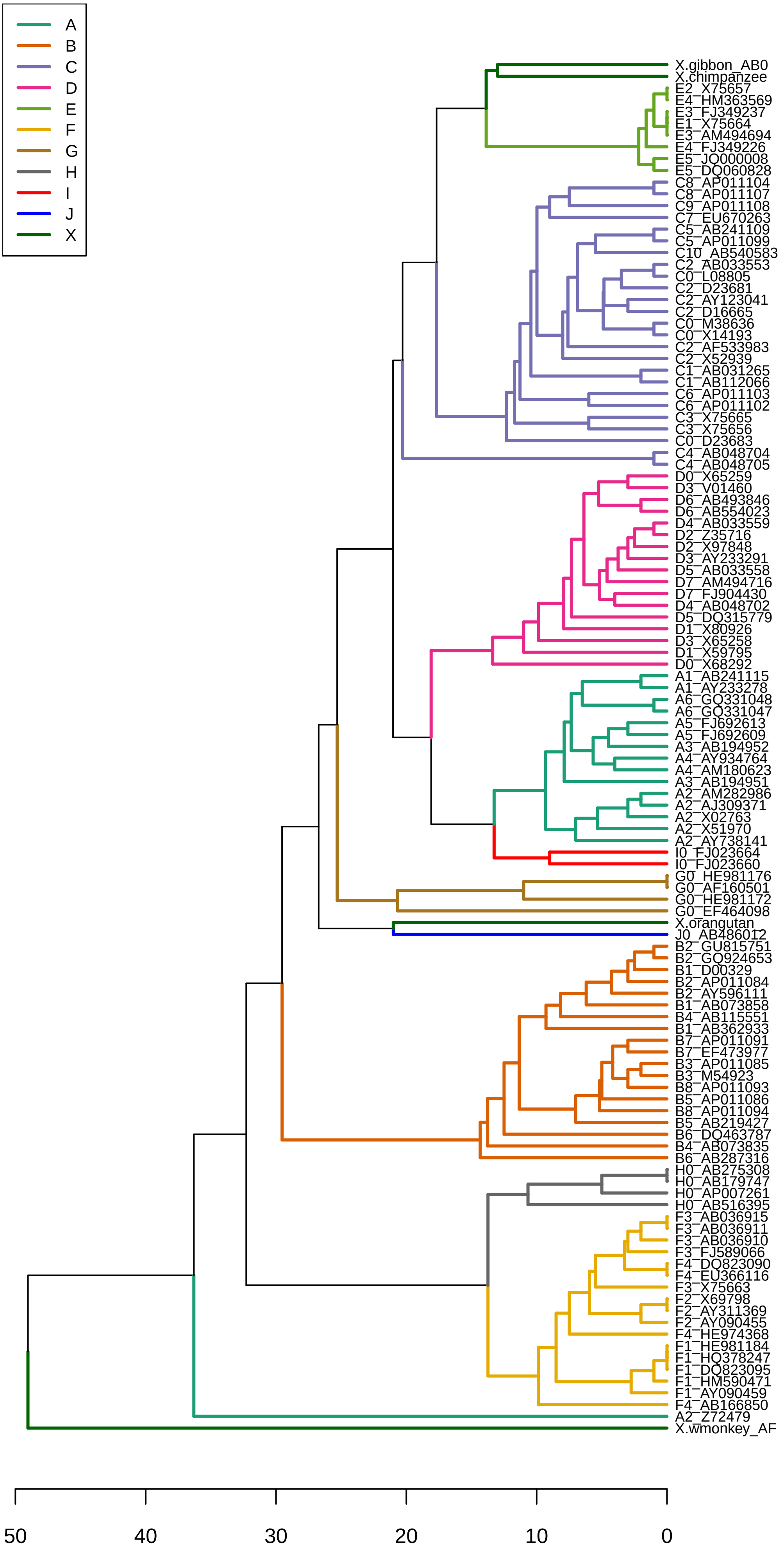

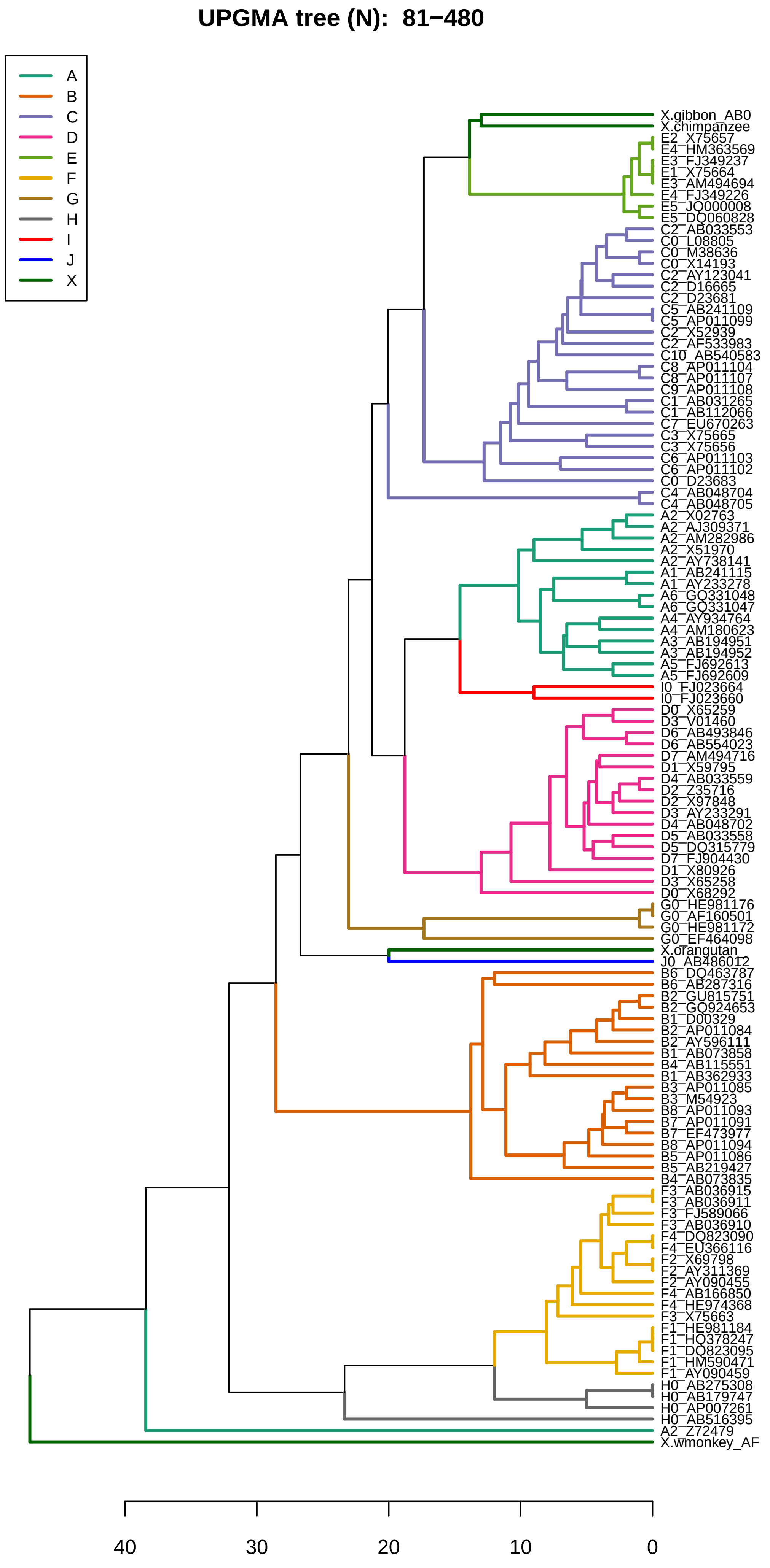
UPGMA tree (N): 81−480
A
B
C
D
E
F
G
H
I
J
X
X.gibbon_AB0
X.chimpanzee
E2_X75657
E4_HM363569
E3_FJ349237
E1_X75664
E3_AM494694
E4_FJ349226
E5_JQ000008
E5_DQ060828
C2_AB033553
C0_L08805
C0_M38636
C0_X14193
C2_AY123041
C2_D16665
C2_D23681
C5_AB241109
C5_AP011099
C2_X52939
C2_AF533983
C10_AB540583
C8_AP011104
C8_AP011107
C9_AP011108
C1_AB031265
C1_AB112066
C7_EU670263
C3_X75665
C3_X75656
C6_AP011103
C6_AP011102
C0_D23683
C4_AB048704
C4_AB048705
A2_X02763
A2_AJ309371
A2_AM282986
A2_X51970
A2_AY738141
A1_AB241115
A1_AY233278
A6_GQ331048
A6_GQ331047
A4_AY934764
A4_AM180623
A3_AB194951
A3_AB194952
A5_FJ692613
A5_FJ692609
I0_FJ023664
I0_FJ023660
D0_X65259
D3_V01460
D6_AB493846
D6_AB554023
D7_AM494716
D1_X59795
D4_AB033559
D2_Z35716
D2_X97848
D3_AY233291
D4_AB048702
D5_AB033558
D5_DQ315779
D7_FJ904430
D1_X80926
D3_X65258
D0_X68292
G0_HE981176
G0_AF160501
G0_HE981172
G0_EF464098
X.orangutan
J0_AB486012
B6_DQ463787
B6_AB287316
B2_GU815751
B2_GQ924653
B1_D00329
B2_AP011084
B2_AY596111
B1_AB073858
B4_AB115551
B1_AB362933
B3_AP011085
B3_M54923
B8_AP011093
B7_AP011091
B7_EF473977
B8_AP011094
B5_AP011086
B5_AB219427
B4_AB073835
F3_AB036915
F3_AB036911
F3_FJ589066
F3_AB036910
F4_DQ823090
F4_EU366116
F2_X69798
F2_AY311369
F2_AY090455
F4_AB166850
F4_HE974368
F3_X75663
F1_HE981184
F1_HQ378247
F1_DQ823095
F1_HM590471
F1_AY090459
H0_AB275308
H0_AB179747
H0_AP007261
H0_AB516395
A2_Z72479
X.wmonkey_AF
40
30
20
10
0

UPGMA tree (N): 121−520

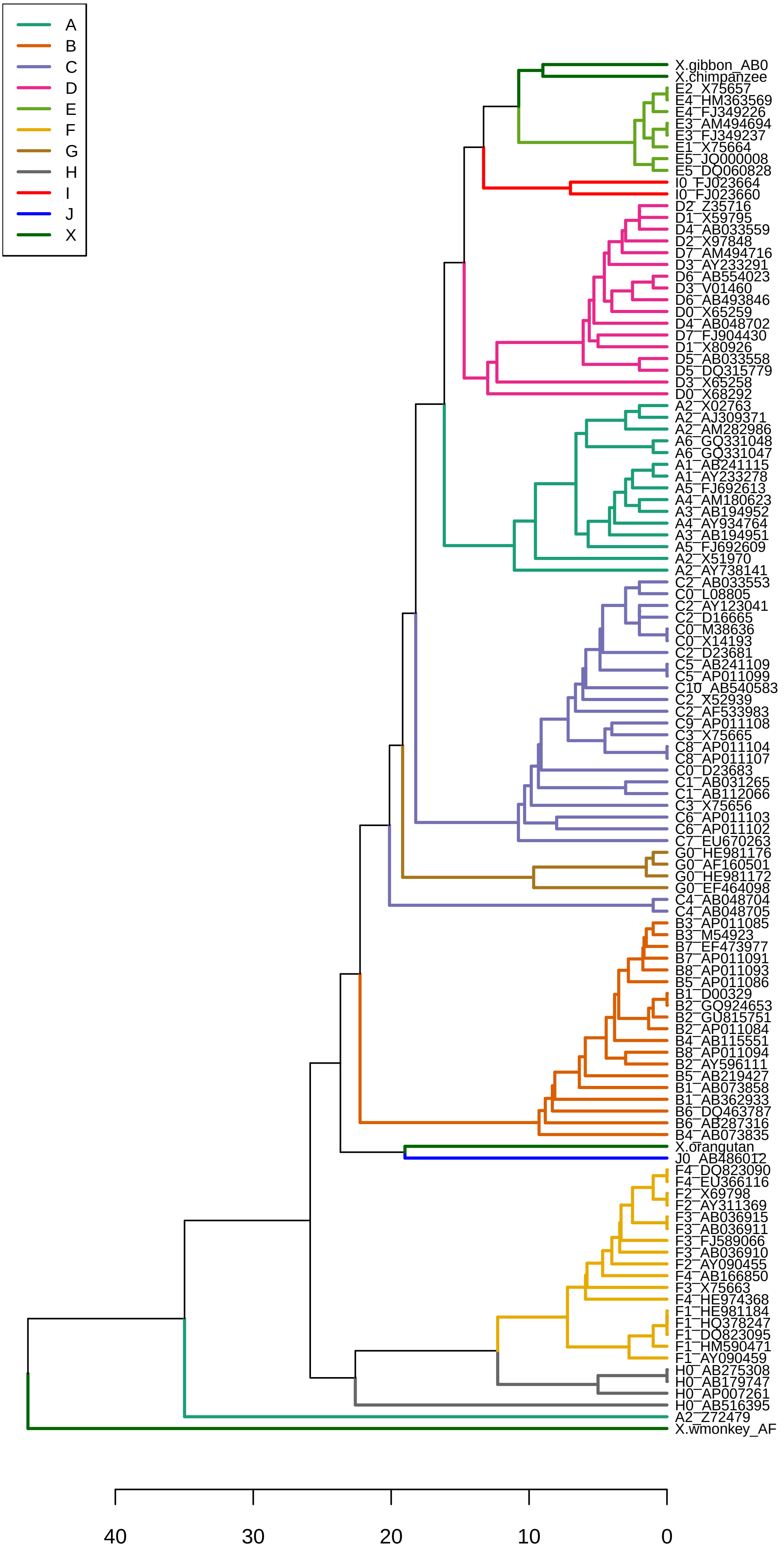

UPGMA tree (N): 161−560

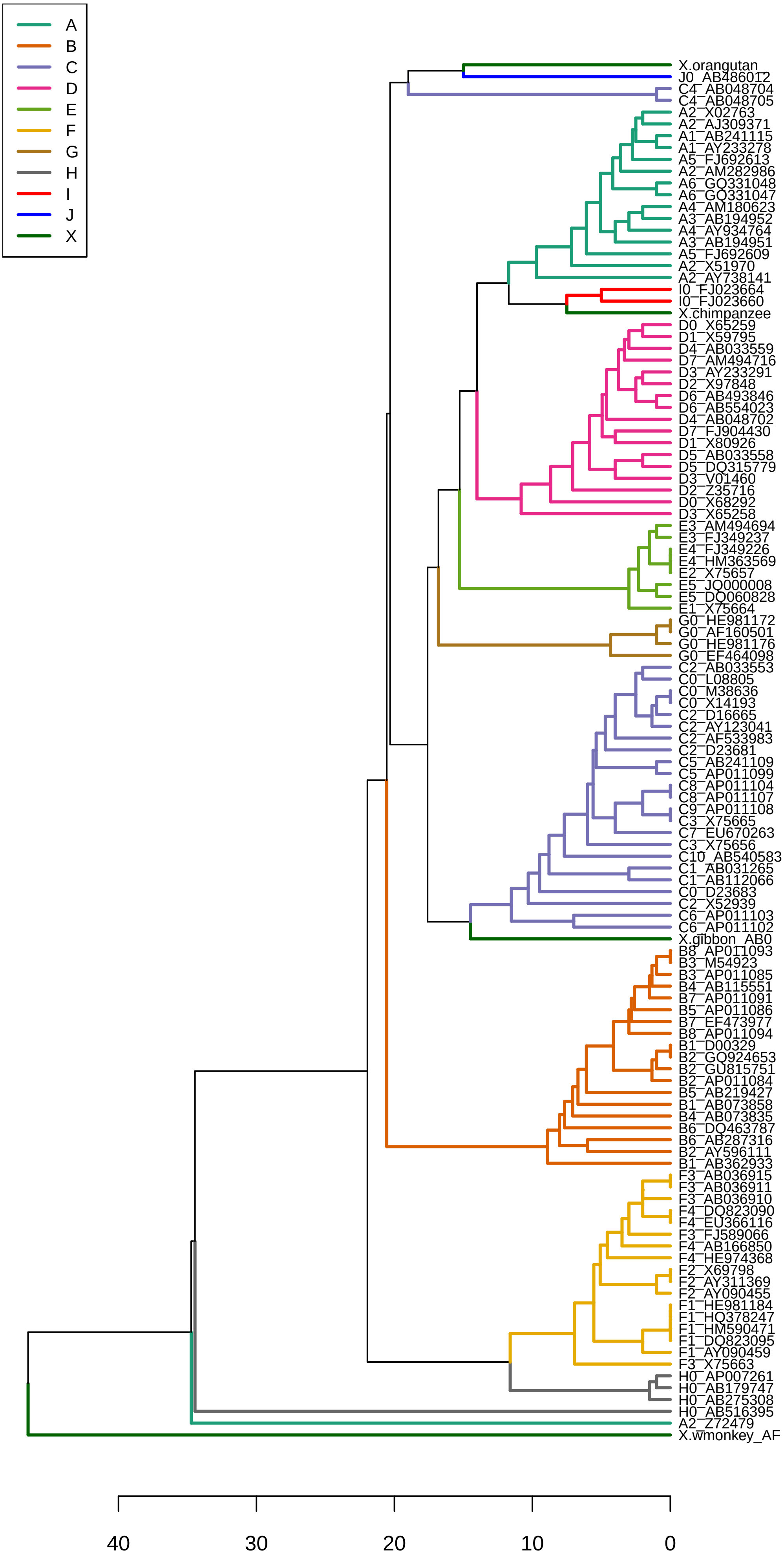

UPGMA tree (N): 201−600

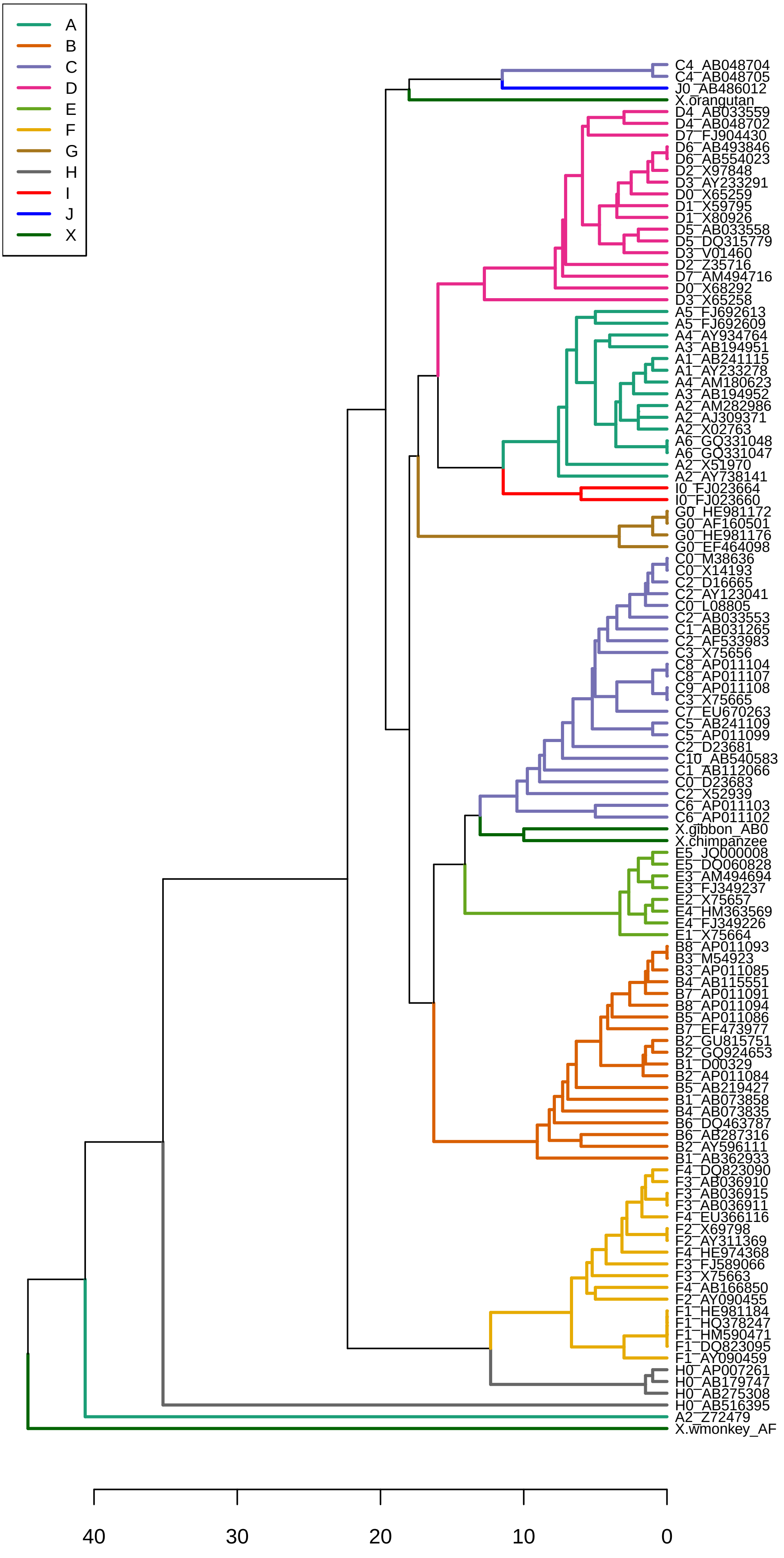

UPGMA tree (N): 241−640

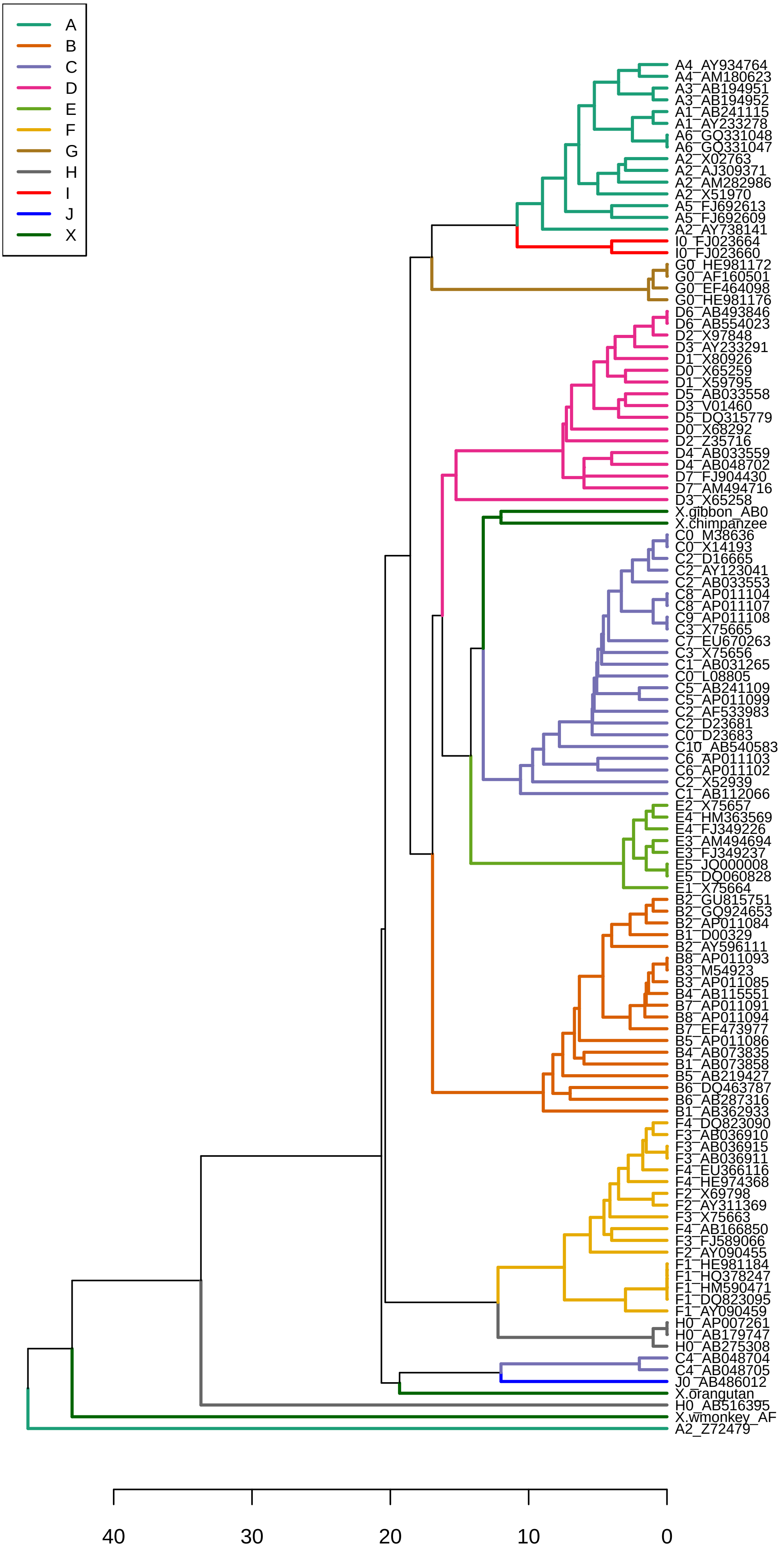

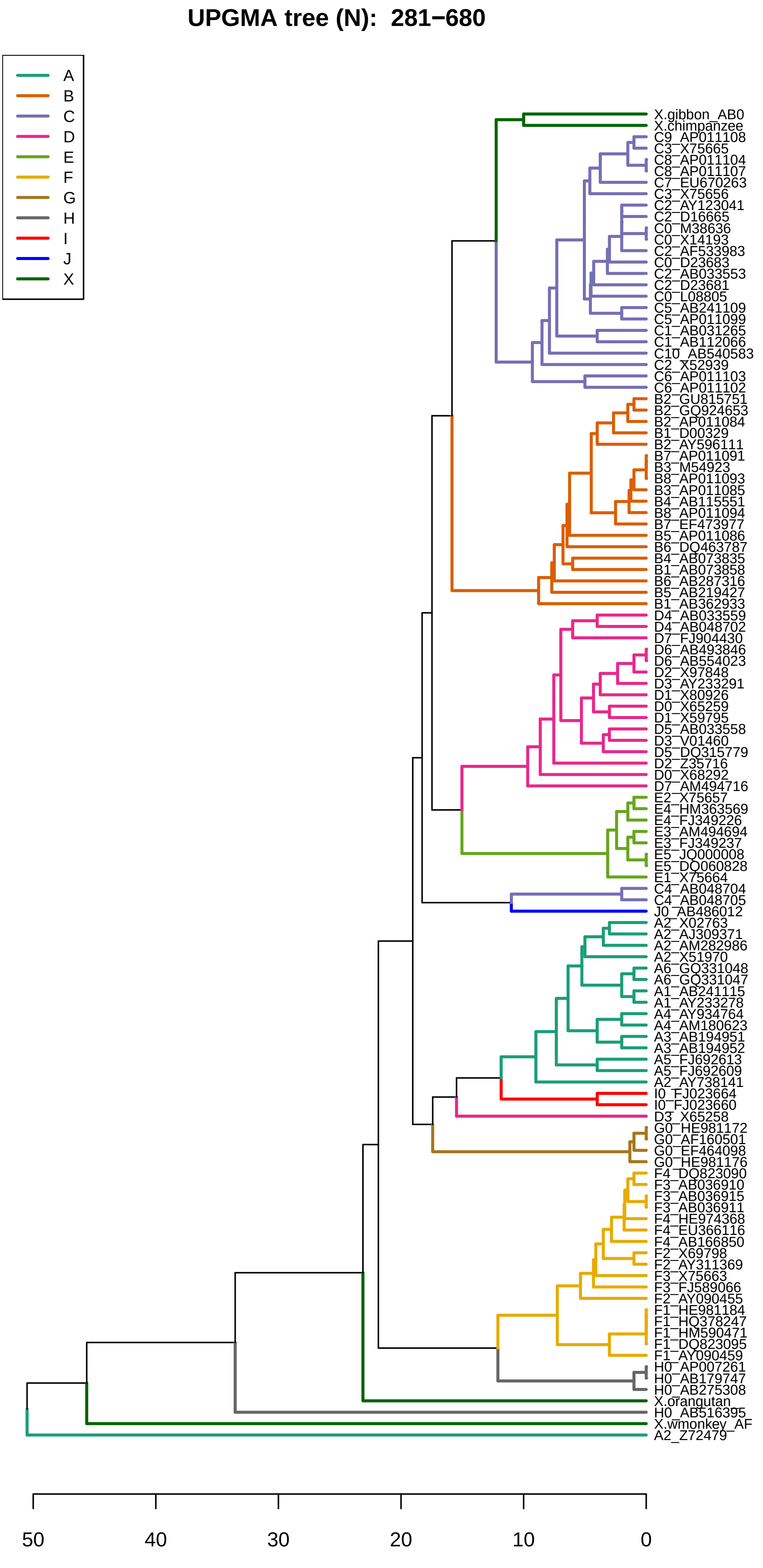

UPGMA tree (N): 281−680
A
B
C
D
E
F
G
H
I
J
X
X.gibbon_AB0
X.chimpanzee
C9_AP011108
C3_X75665
C8_AP011104
C8_AP011107
C7_EU670263
C3_X75656
C2_AY123041
C2_D16665
C0_M38636
C0_X14193
C2_AF533983
C0_D23683
C2_AB033553
C2_D23681
C0_L08805
C5_AB241109
C5_AP011099
C1_AB031265
C1_AB112066
C10_AB540583
C2_X52939
C6_AP011103
C6_AP011102
B2_GU815751
B2_GQ924653
B2_AP011084
B1_D00329
B2_AY596111
B7_AP011091
B3_M54923
B8_AP011093
B3_AP011085
B4_AB115551
B8_AP011094
B7_EF473977
B5_AP011086
B6_DQ463787
B4_AB073835
B1_AB073858
B6_AB287316
B5_AB219427
B1_AB362933
D4_AB033559
D4_AB048702
D7_FJ904430
D6_AB493846
D6_AB554023
D2_X97848
D3_AY233291
D1_X80926
D0_X65259
D1_X59795
D5_AB033558
D3_V01460
D5_DQ315779
D2_Z35716
D0_X68292
D7_AM494716
E2_X75657
E4_HM363569
E4_FJ349226
E3_AM494694
E3_FJ349237
E5_JQ000008
E5_DQ060828
E1_X75664
C4_AB048704
C4_AB048705
J0_AB486012
A2_X02763
A2_AJ309371
A2_AM282986
A2_X51970
A6_GQ331048
A6_GQ331047
A1_AB241115
A1_AY233278
A4_AY934764
A4_AM180623
A3_AB194951
A3_AB194952
A5_FJ692613
A5_FJ692609
A2_AY738141
I0_FJ023664
I0_FJ023660
D3_X65258
G0_HE981172
G0_AF160501
G0_EF464098
G0_HE981176
F4_DQ823090
F3_AB036910
F3_AB036915
F3_AB036911
F4_HE974368
F4_EU366116
F4_AB166850
F2_X69798
F2_AY311369
F3_X75663
F3_FJ589066
F2_AY090455
F1_HE981184
F1_HQ378247
F1_HM590471
F1_DQ823095
F1_AY090459
H0_AP007261
H0_AB179747
H0_AB275308
X.orangutan
H0_AB516395
X.wmonkey_AF
A2_Z72479
50
40
30
20
10
0

UPGMA tree (N): 321−720

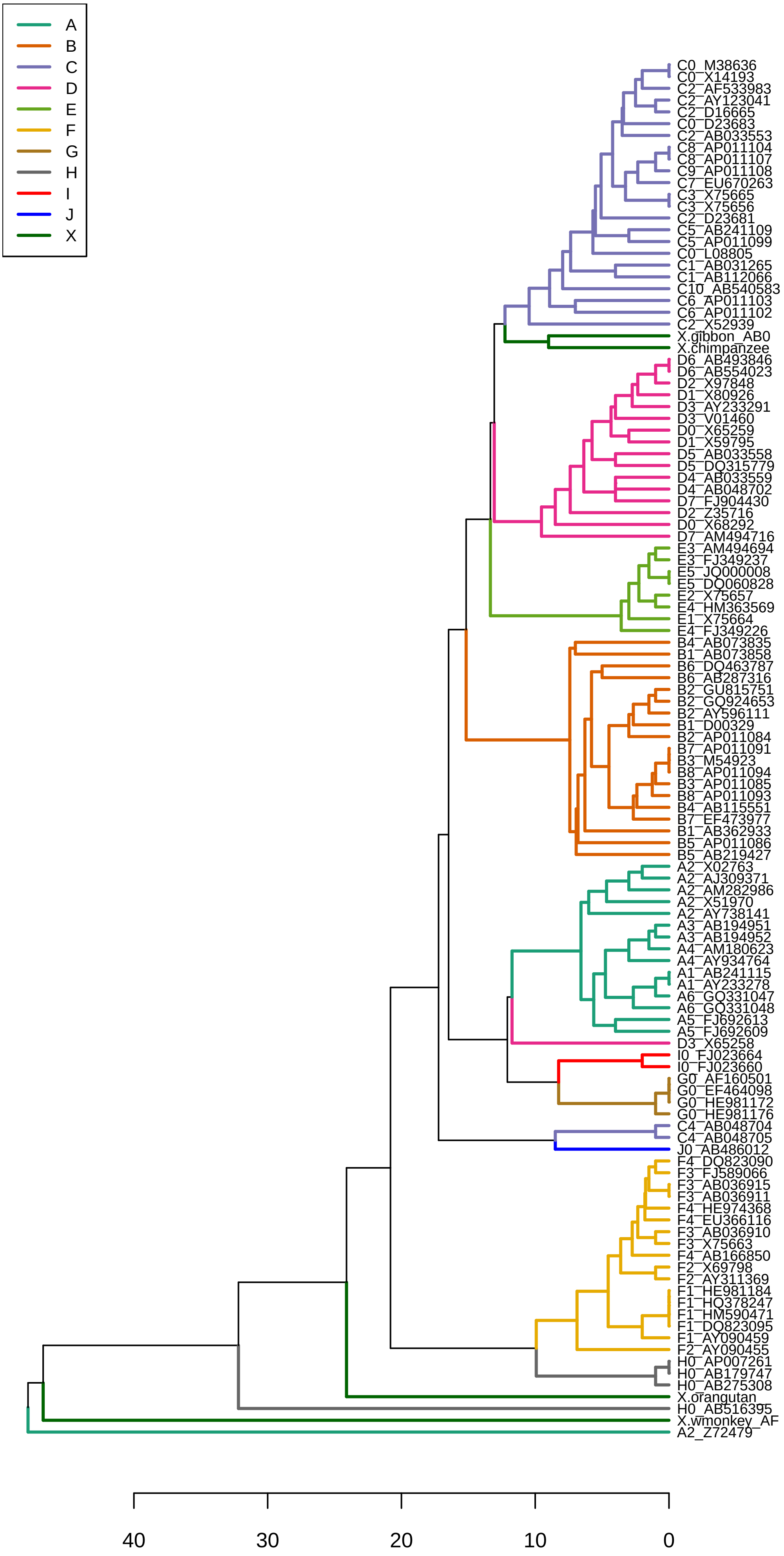

UPGMA tree (N): 361−760

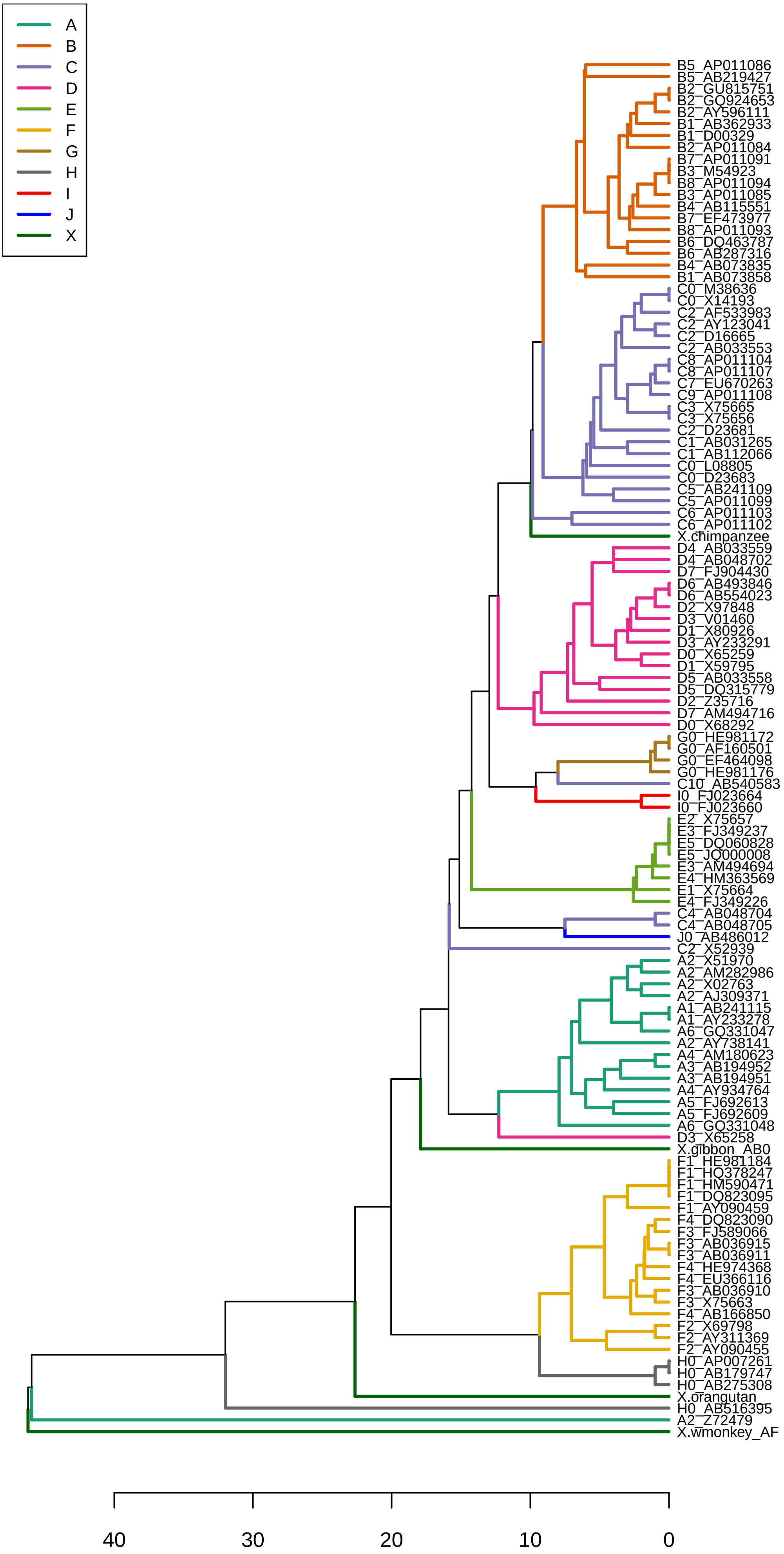

UPGMA tree (N): 401−800

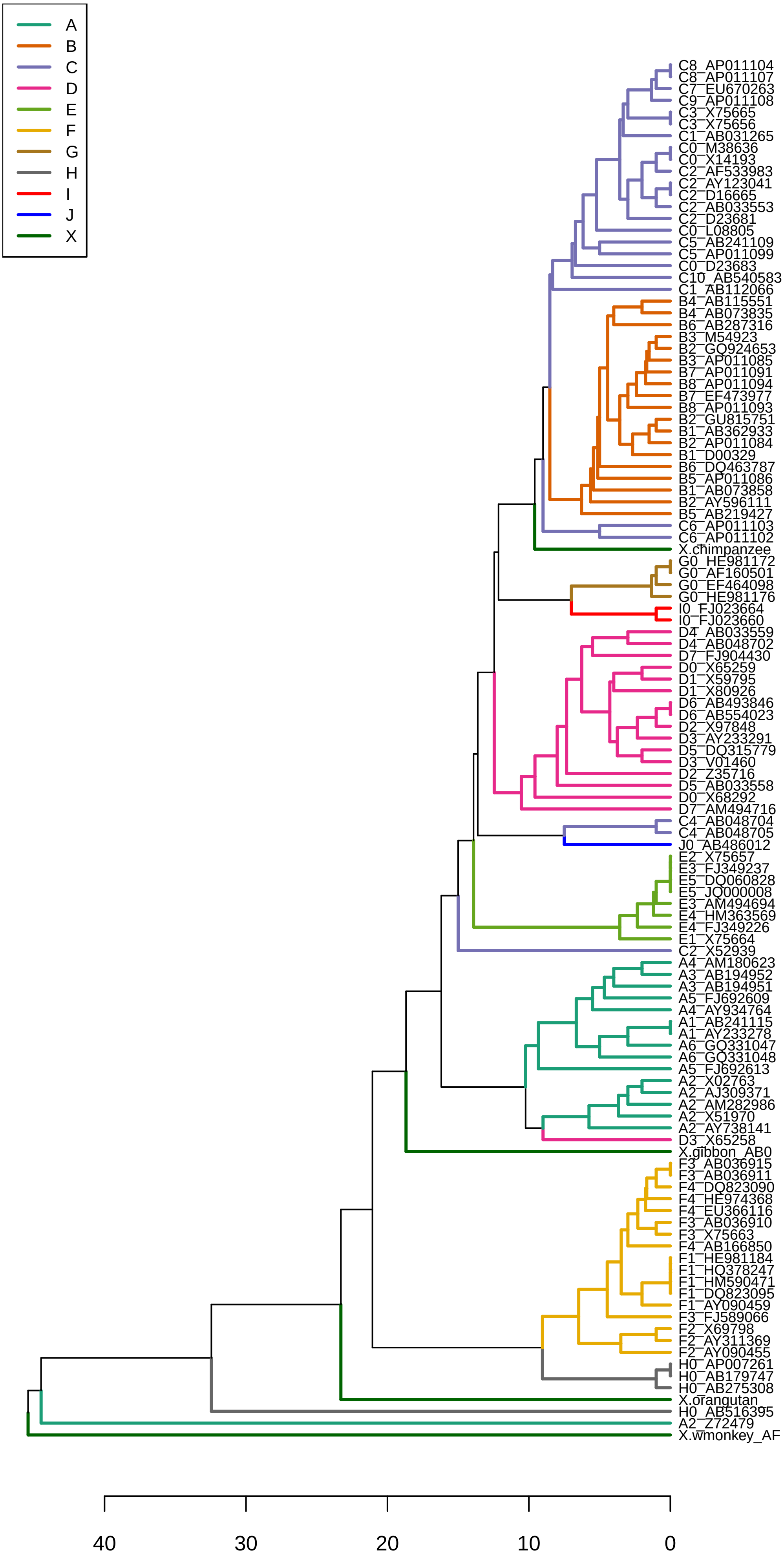

UPGMA tree (N): 441−840

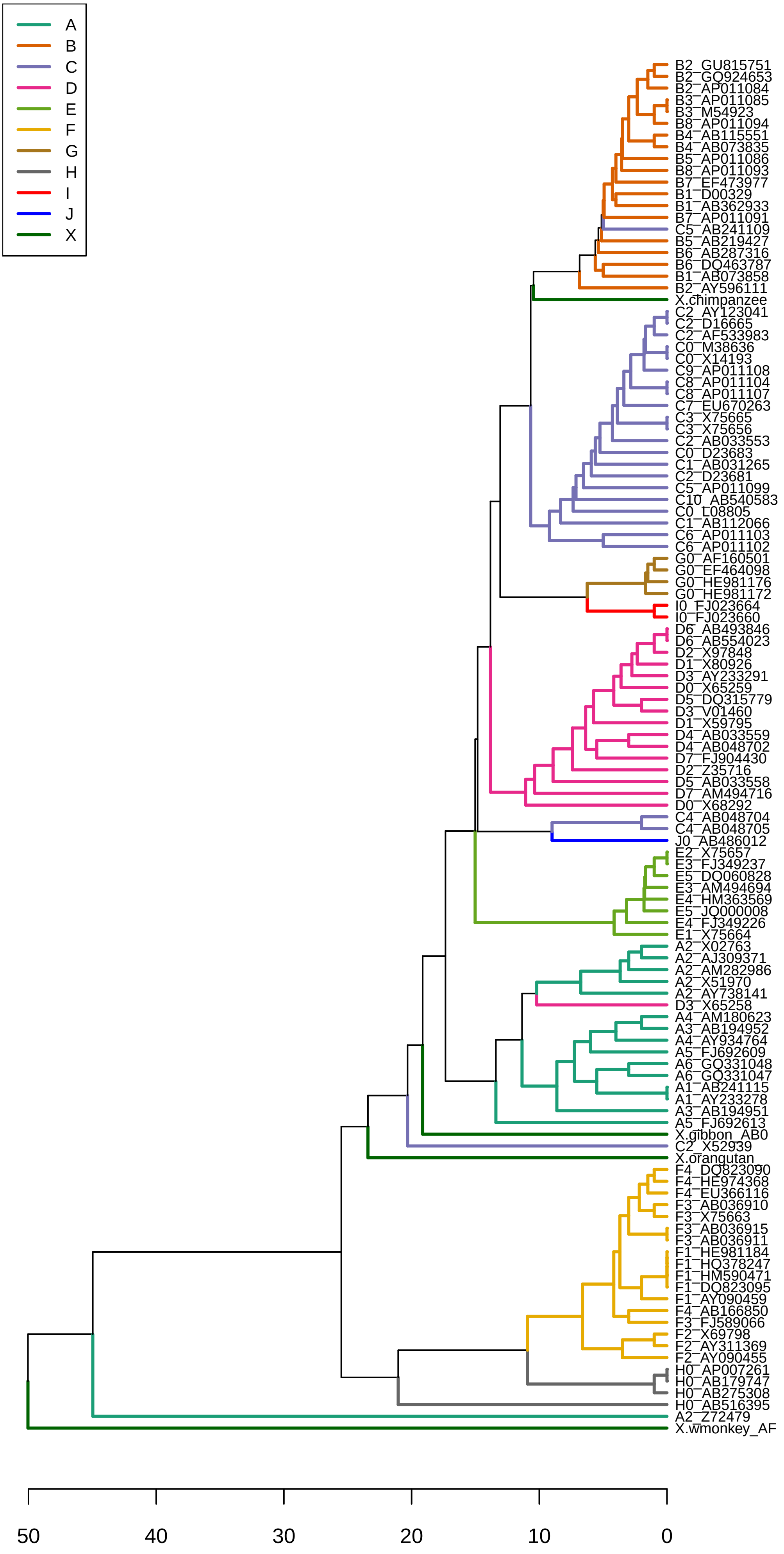

UPGMA tree (N): 481−880

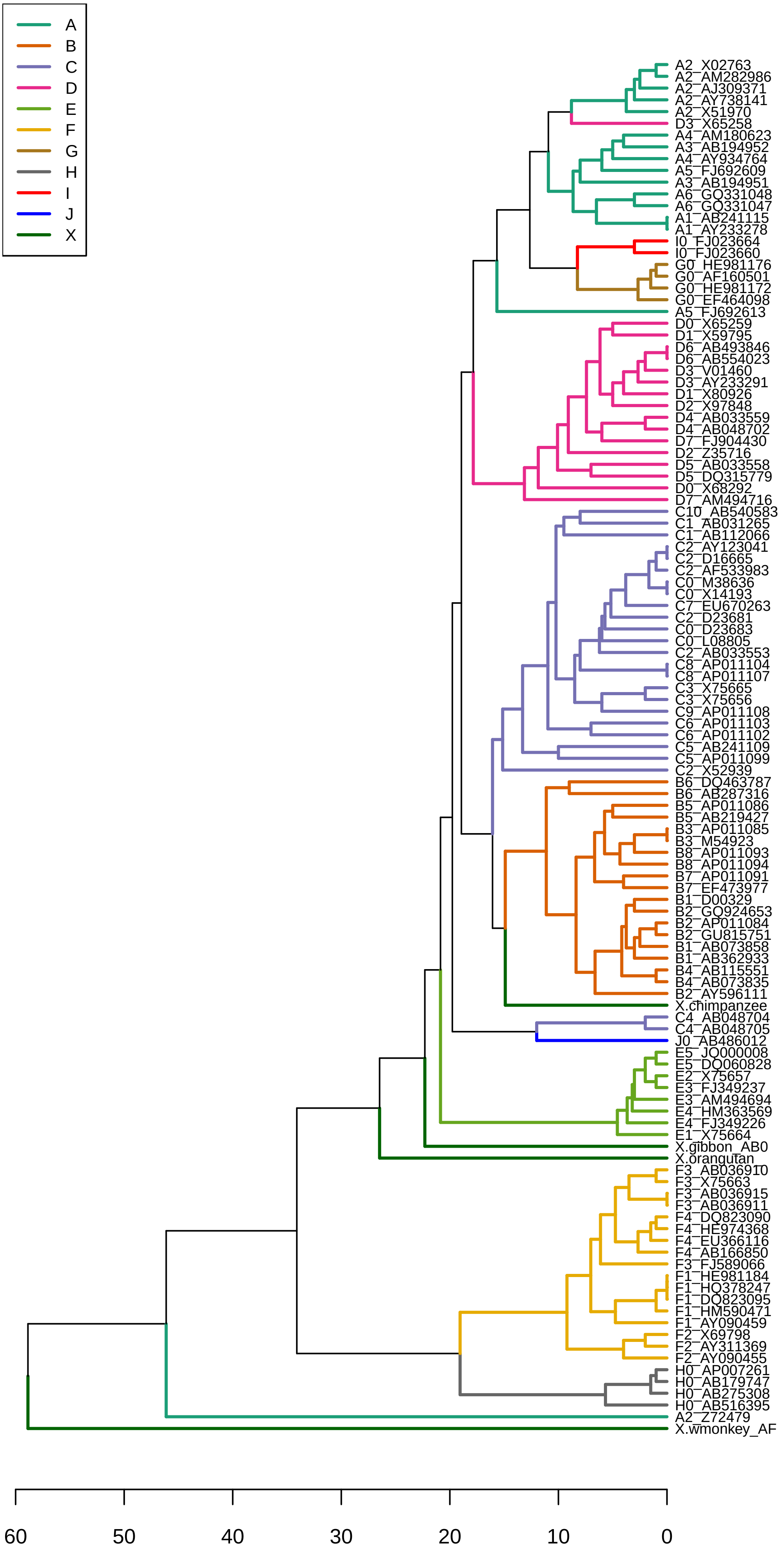

UPGMA tree (N): 521−920

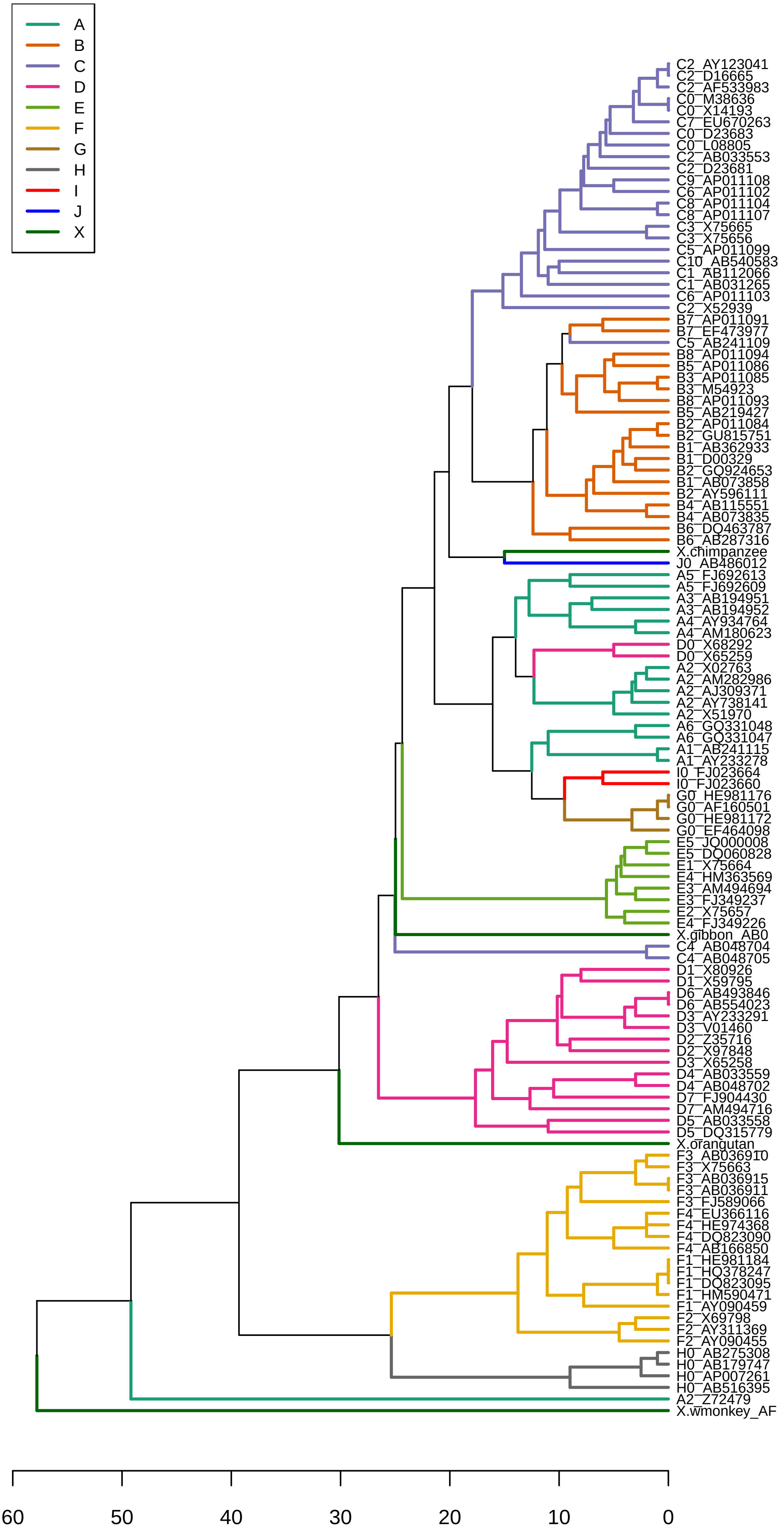

UPGMA tree (N): 561−960

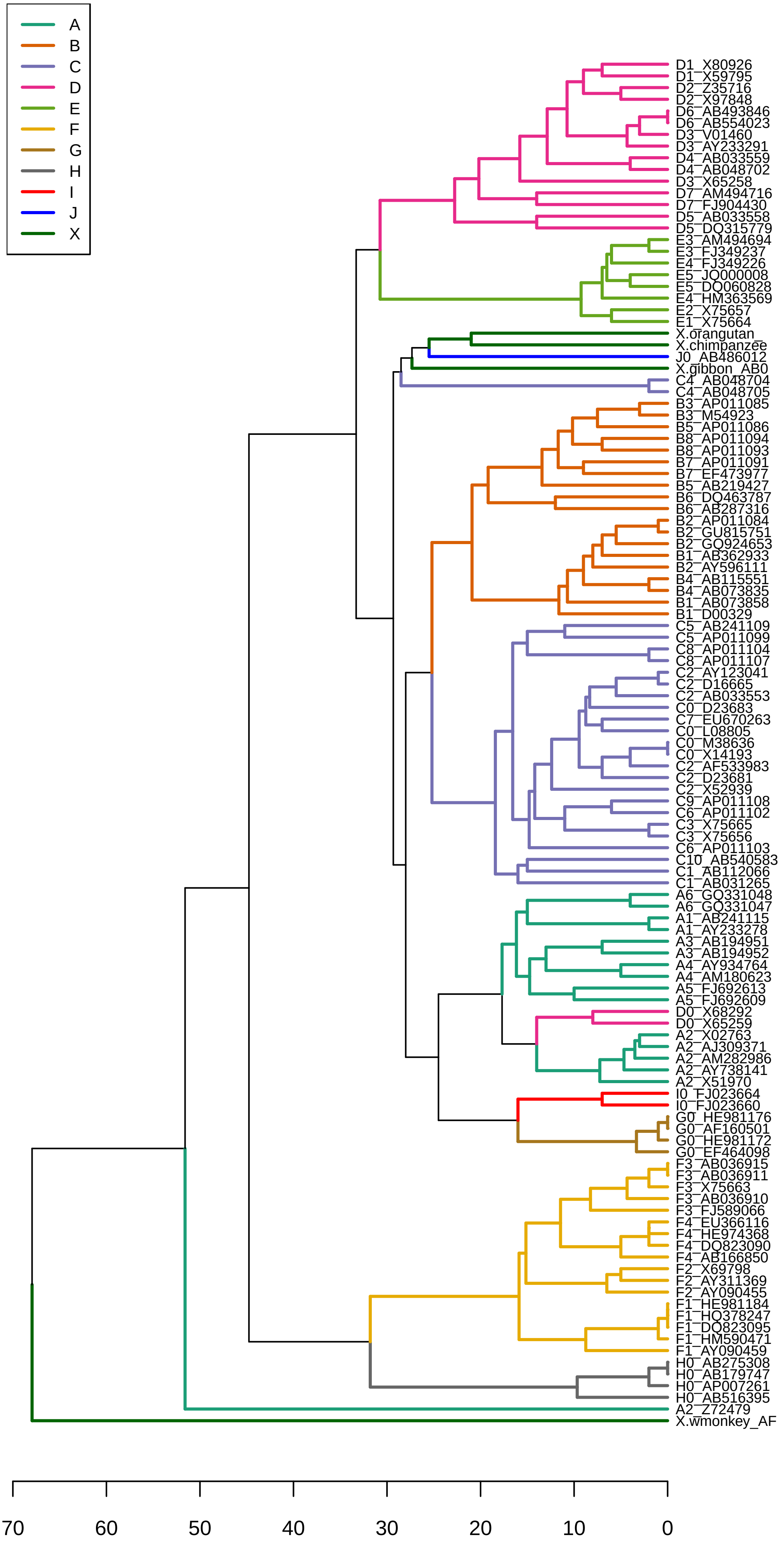

UPGMA tree (N): 601−1000

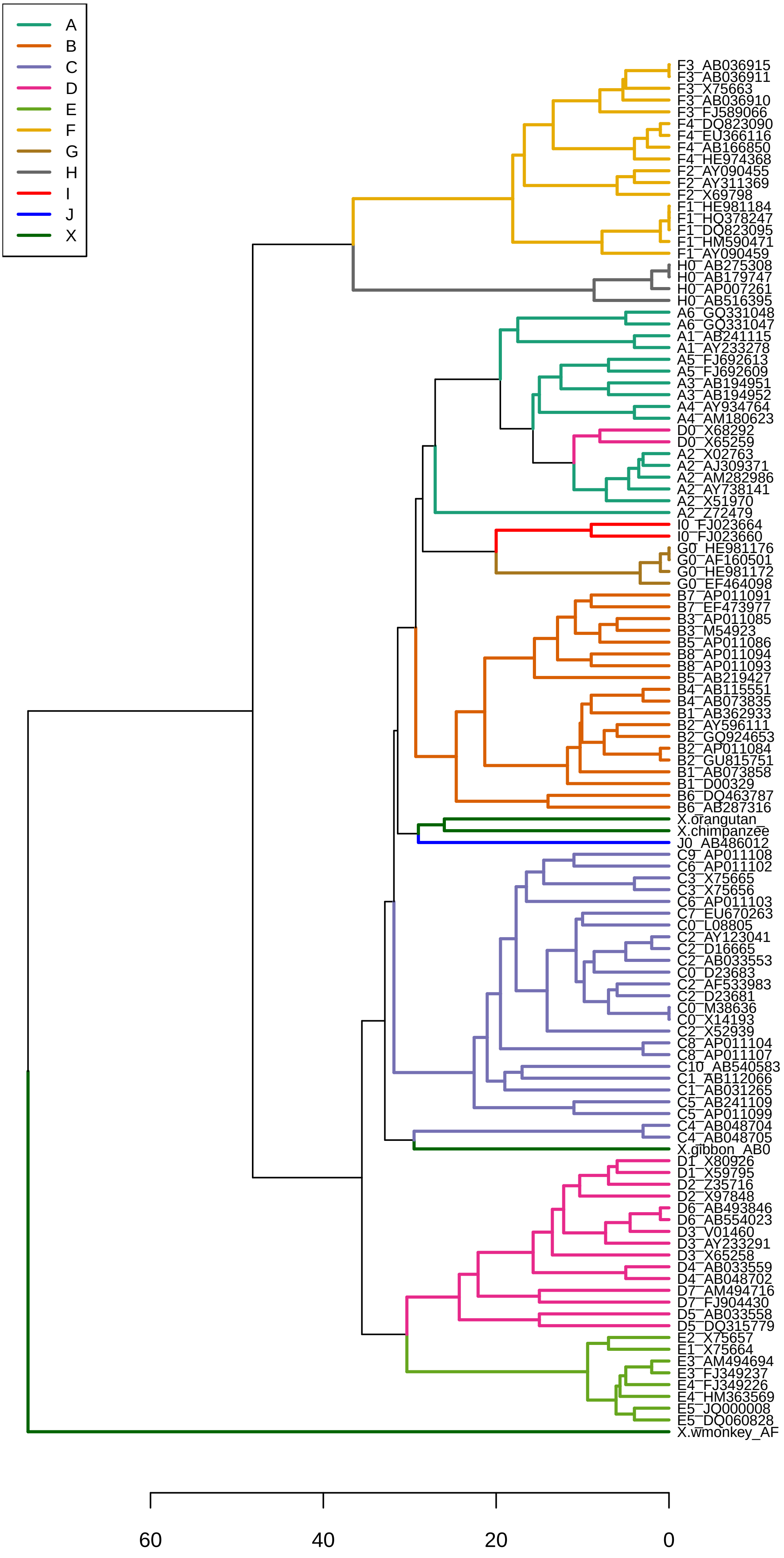

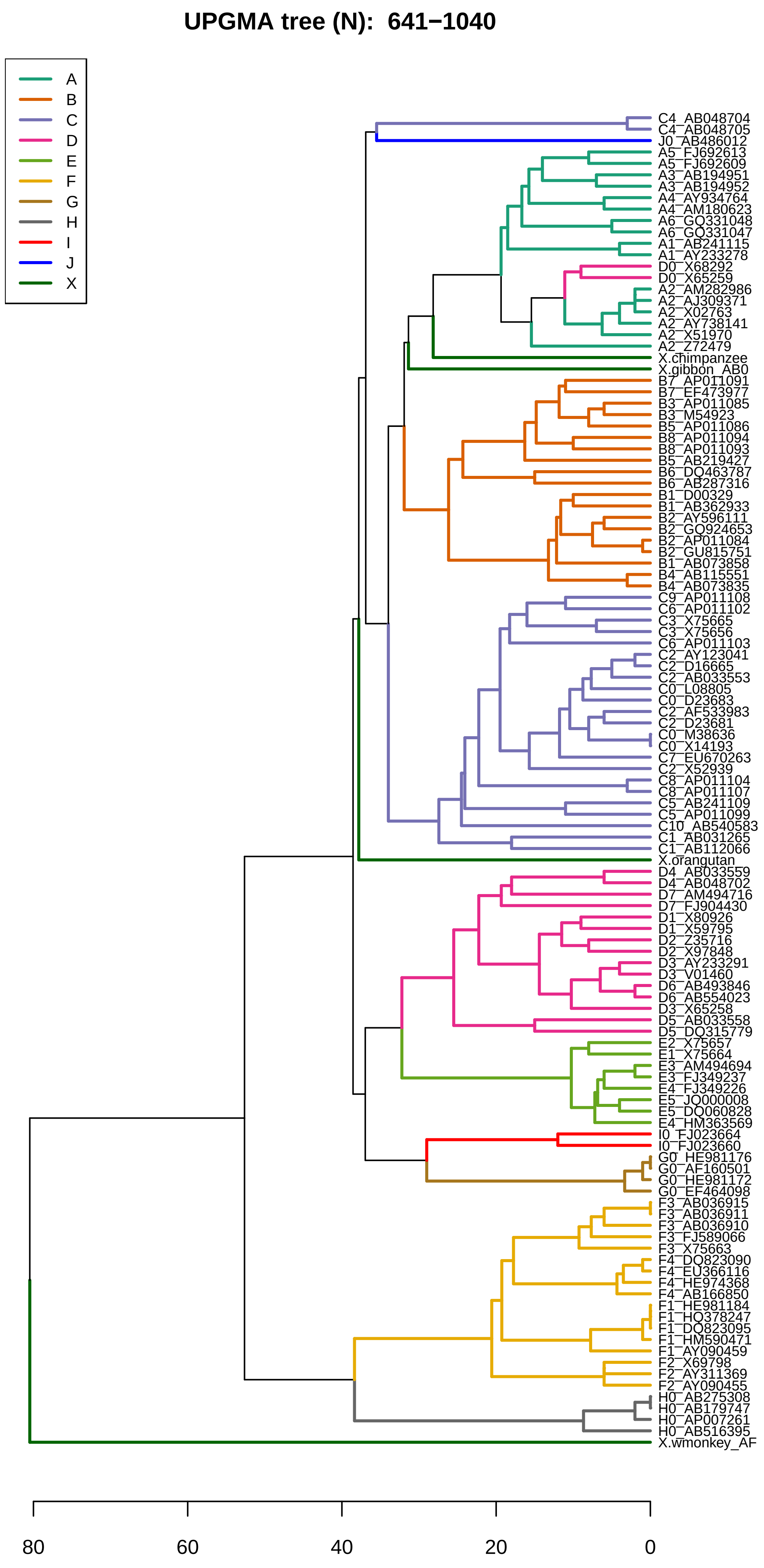

UPGMA tree (N): 641−1040
A
B
C
D
E
F
G
H
I
J
X
C4_AB048704
C4_AB048705
J0_AB486012
A5_FJ692613
A5_FJ692609
A3_AB194951
A3_AB194952
A4_AY934764
A4_AM180623
A6_GQ331048
A6_GQ331047
A1_AB241115
A1_AY233278
D0_X68292
D0_X65259
A2_AM282986
A2_AJ309371
A2_X02763
A2_AY738141
A2_X51970
A2_Z72479
X.chimpanzee
X.gibbon_AB0
B7_AP011091
B7_EF473977
B3_AP011085
B3_M54923
B5_AP011086
B8_AP011094
B8_AP011093
B5_AB219427
B6_DQ463787
B6_AB287316
B1_D00329
B1_AB362933
B2_AY596111
B2_GQ924653
B2_AP011084
B2_GU815751
B1_AB073858
B4_AB115551
B4_AB073835
C9_AP011108
C6_AP011102
C3_X75665
C3_X75656
C6_AP011103
C2_AY123041
C2_D16665
C2_AB033553
C0_L08805
C0_D23683
C2_AF533983
C2_D23681
C0_M38636
C0_X14193
C7_EU670263
C2_X52939
C8_AP011104
C8_AP011107
C5_AB241109
C5_AP011099
C10_AB540583
C1_AB031265
C1_AB112066
X.orangutan
D4_AB033559
D4_AB048702
D7_AM494716
D7_FJ904430
D1_X80926
D1_X59795
D2_Z35716
D2_X97848
D3_AY233291
D3_V01460
D6_AB493846
D6_AB554023
D3_X65258
D5_AB033558
D5_DQ315779
E2_X75657
E1_X75664
E3_AM494694
E3_FJ349237
E4_FJ349226
E5_JQ000008
E5_DQ060828
E4_HM363569
I0_FJ023664
I0_FJ023660
G0_HE981176
G0_AF160501
G0_HE981172
G0_EF464098
F3_AB036915
F3_AB036911
F3_AB036910
F3_FJ589066
F3_X75663
F4_DQ823090
F4_EU366116
F4_HE974368
F4_AB166850
F1_HE981184
F1_HQ378247
F1_DQ823095
F1_HM590471
F1_AY090459
F2_X69798
F2_AY311369
F2_AY090455
H0_AB275308
H0_AB179747
H0_AP007261
H0_AB516395
X.wmonkey_AF
80
60
40
20
0

UPGMA tree (N): 681−1080

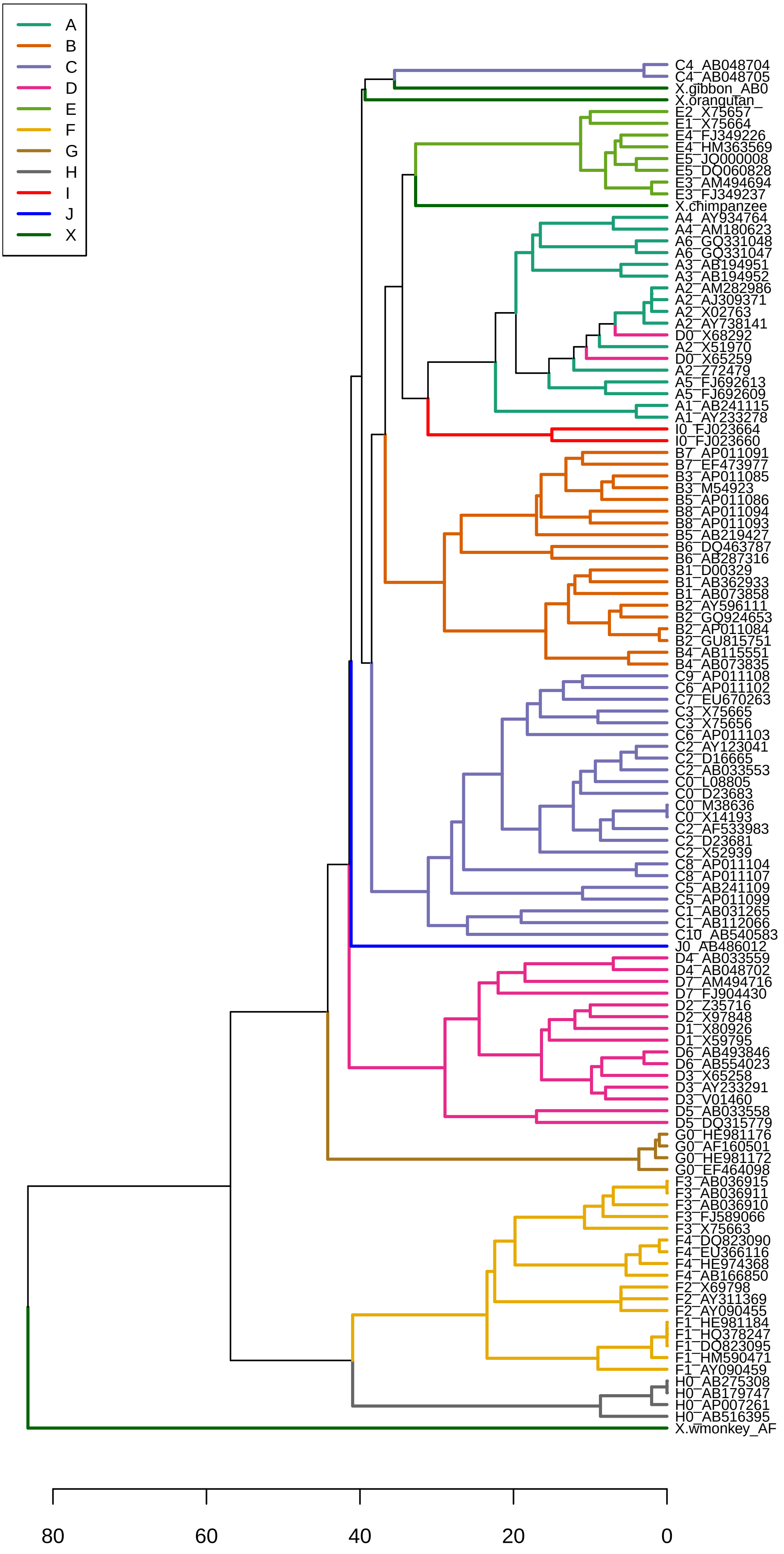

UPGMA tree (N): 721−1120

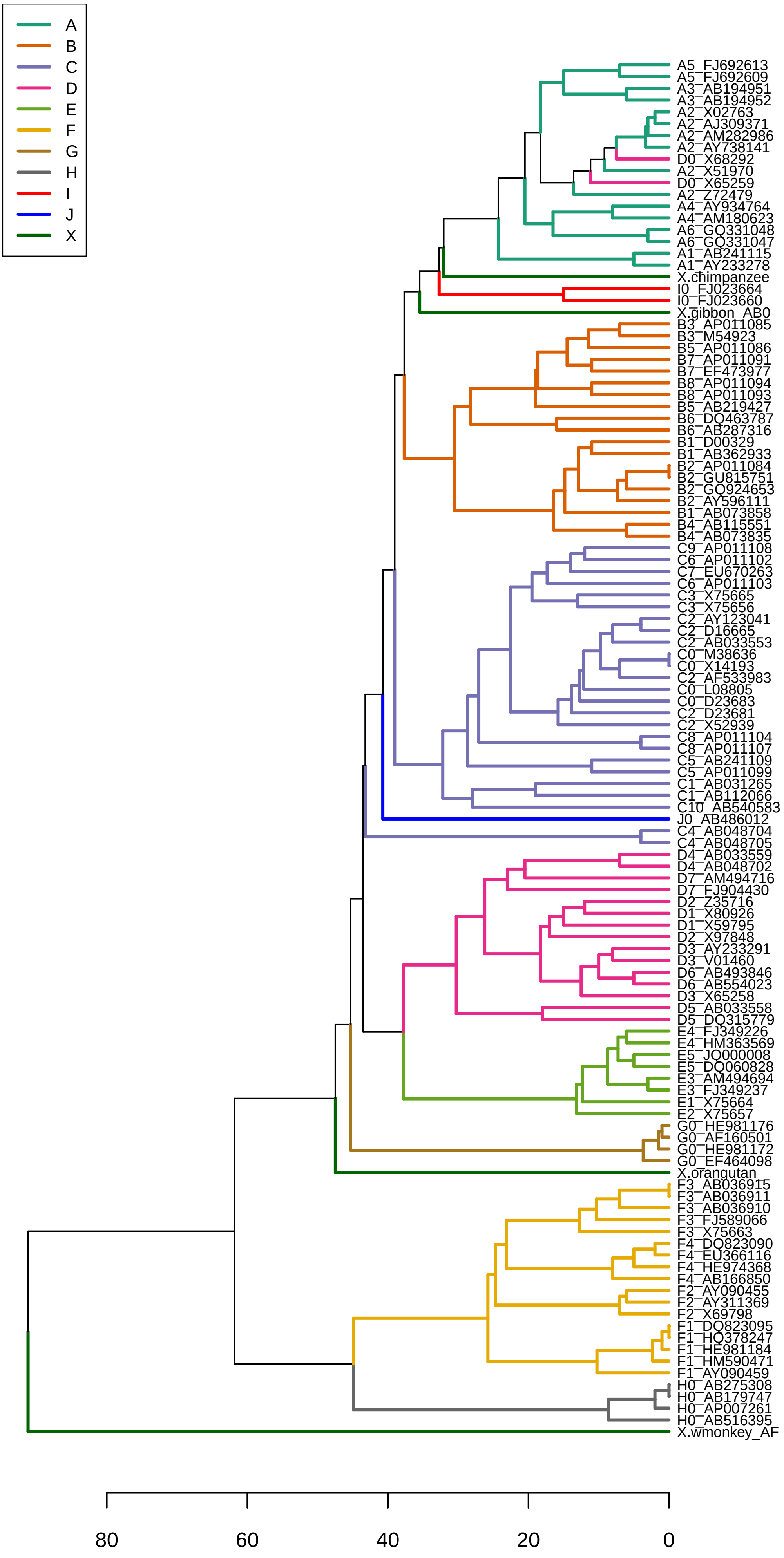

UPGMA tree (N): 761−1160

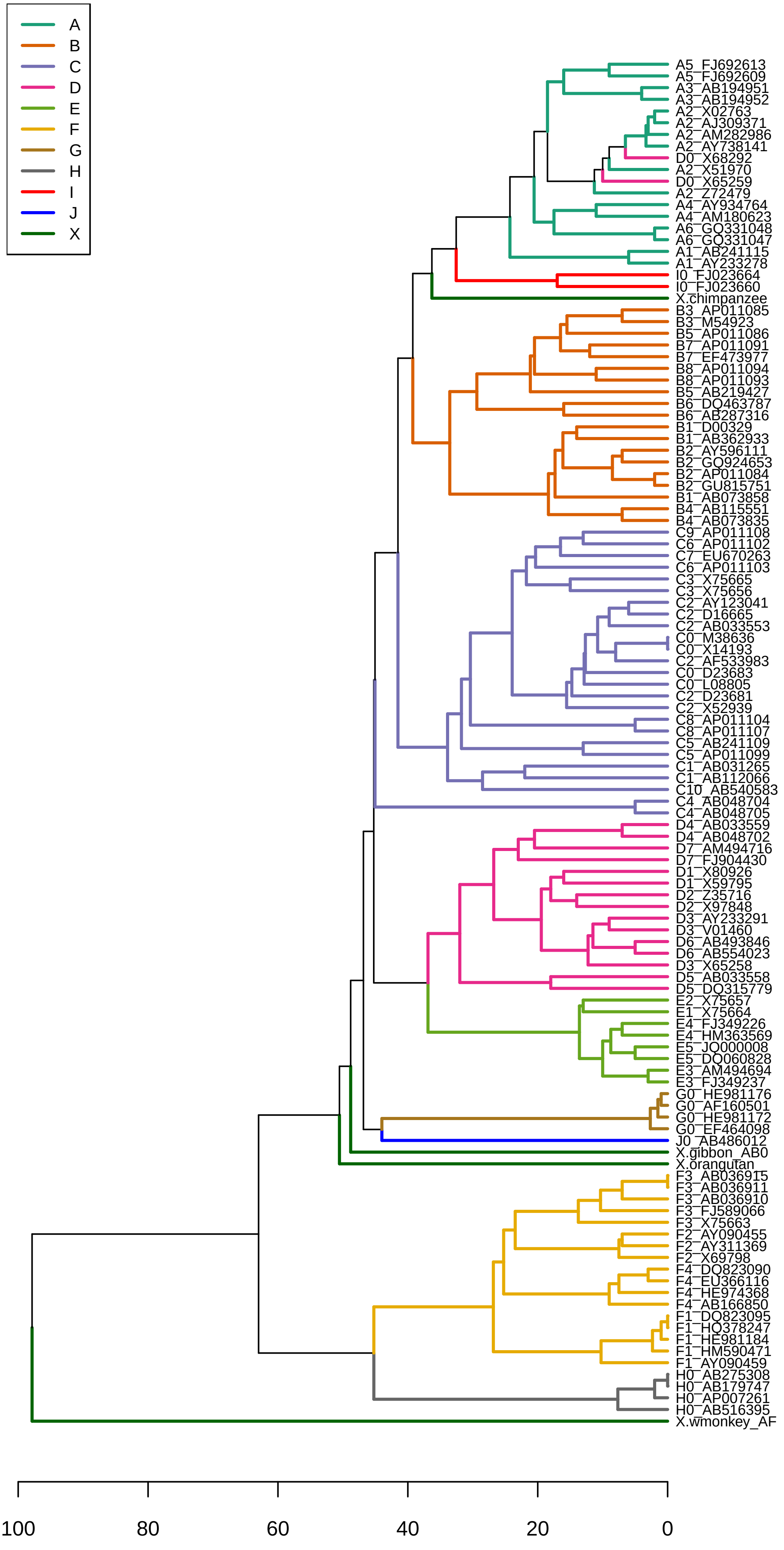

UPGMA tree (N): 801−1200

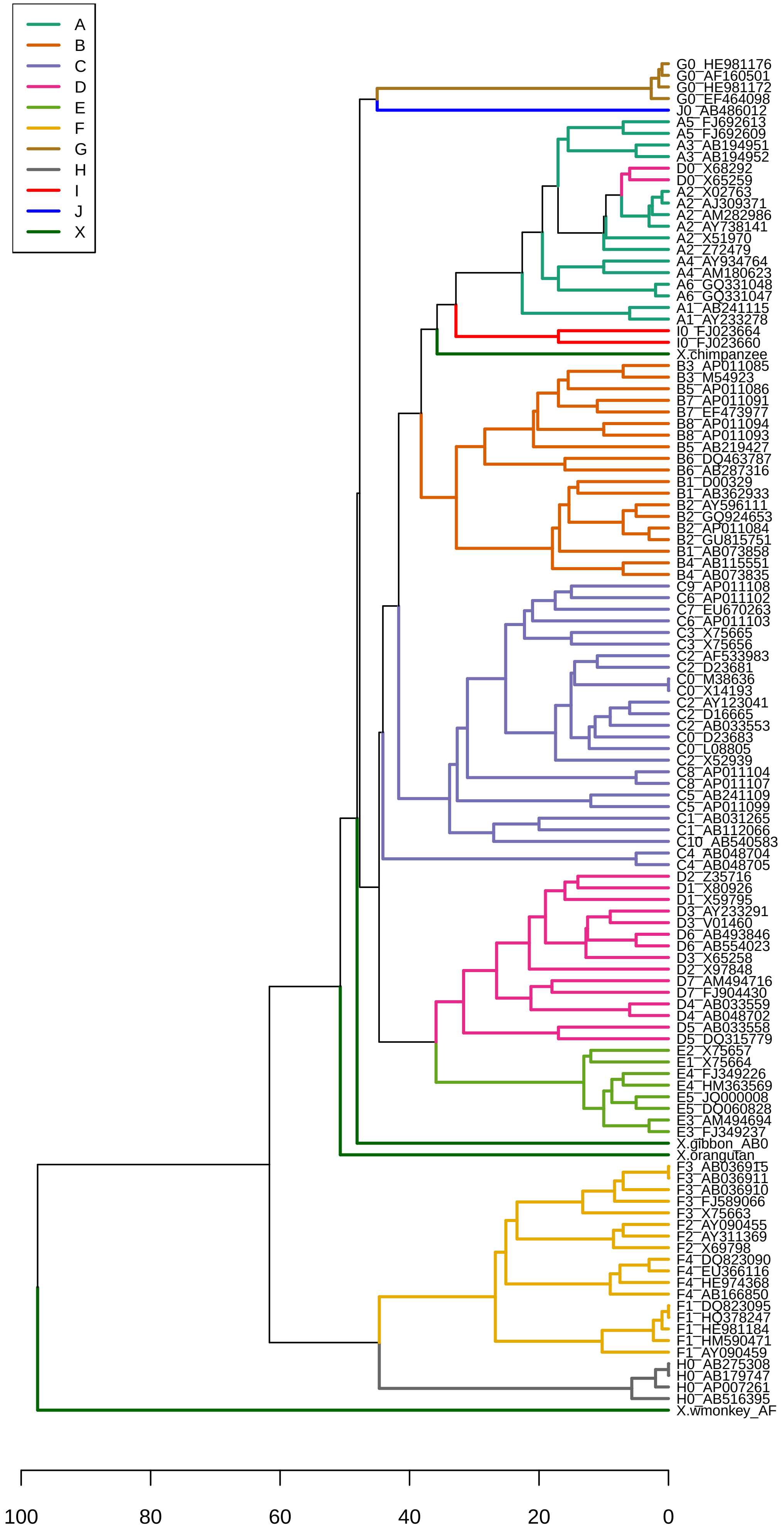

UPGMA tree (N): 841−1240

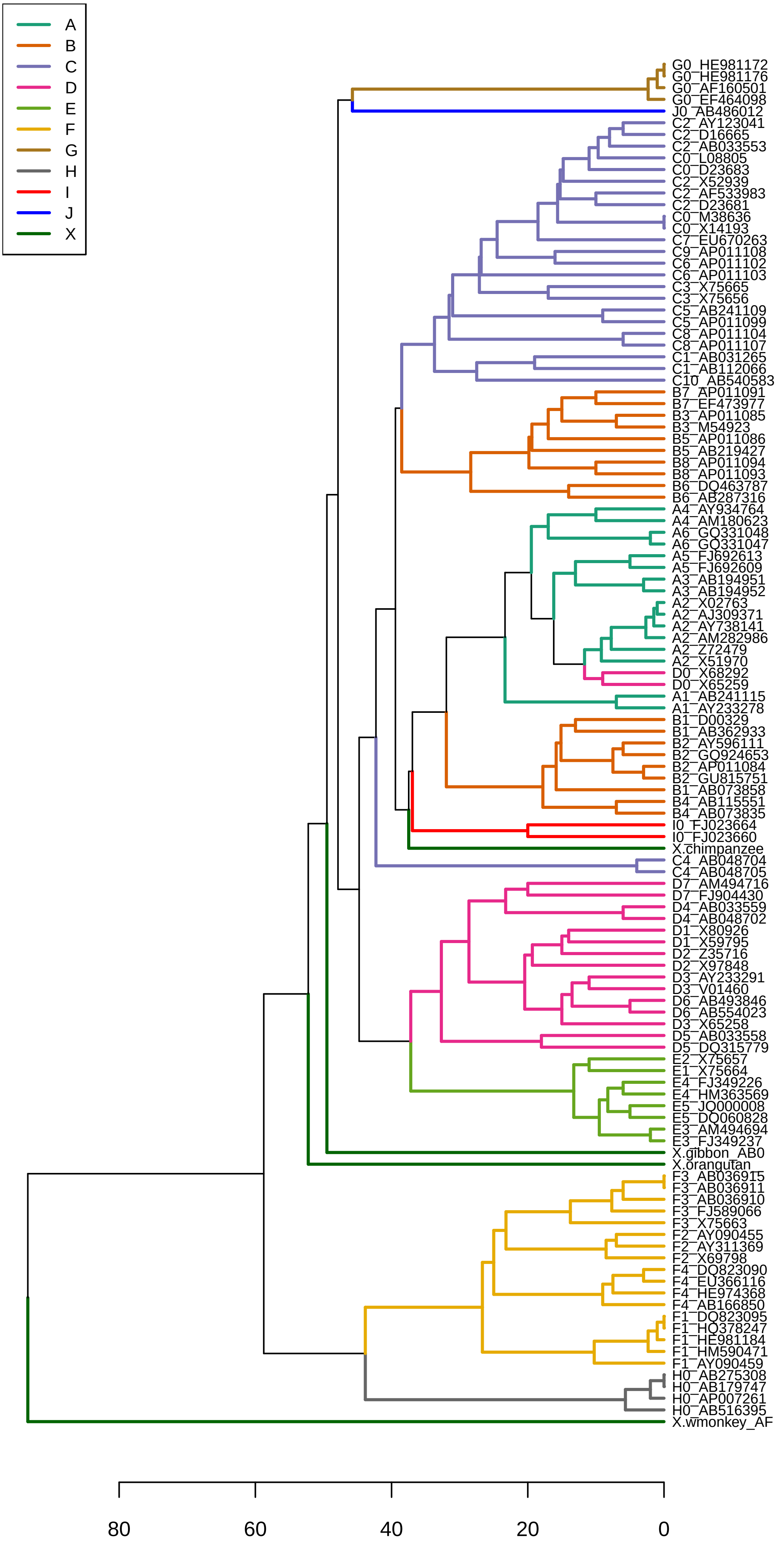

UPGMA tree (N): 881−1280

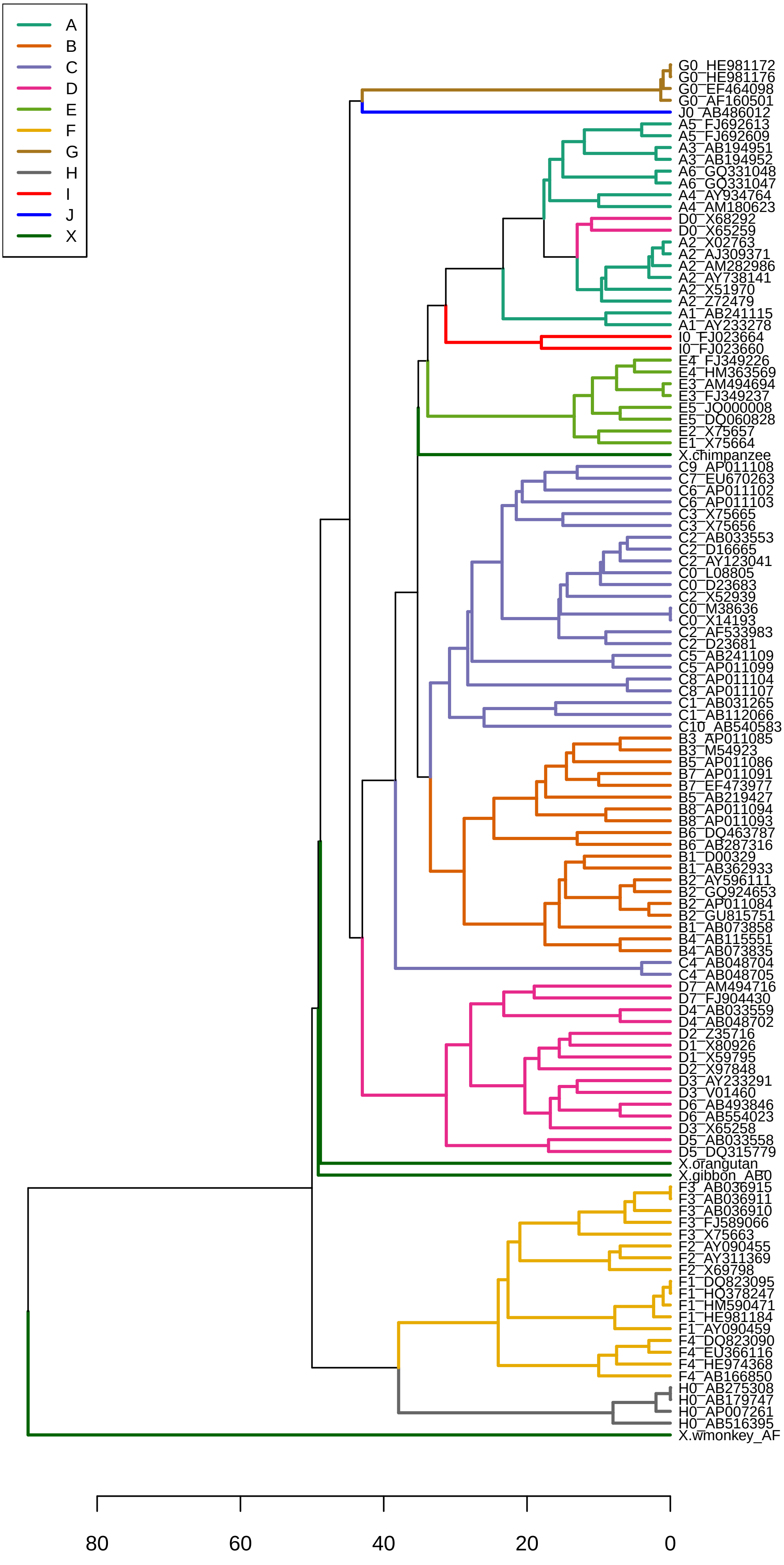

UPGMA tree (N): 921−1320

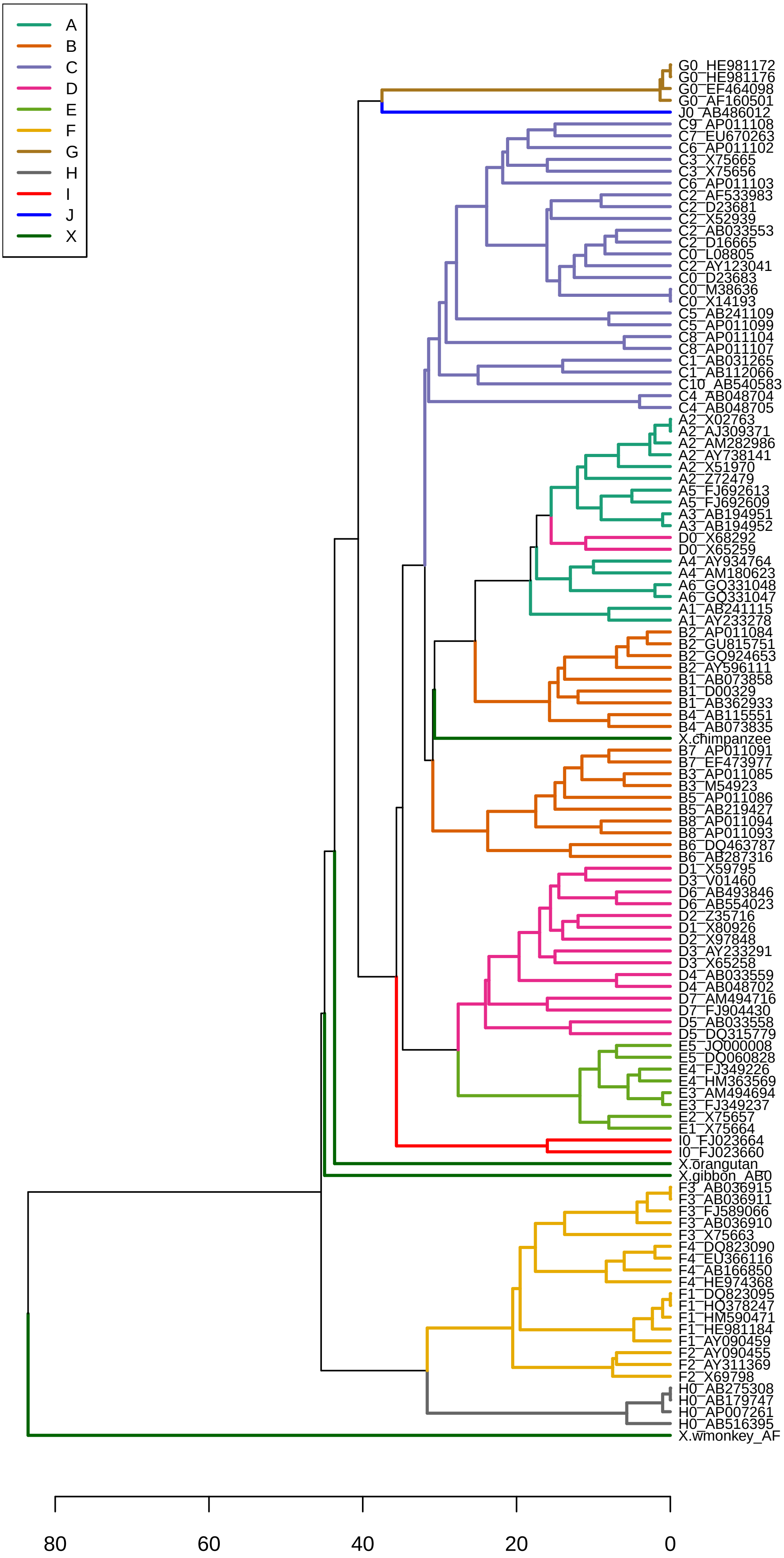

UPGMA tree (N): 961−1360

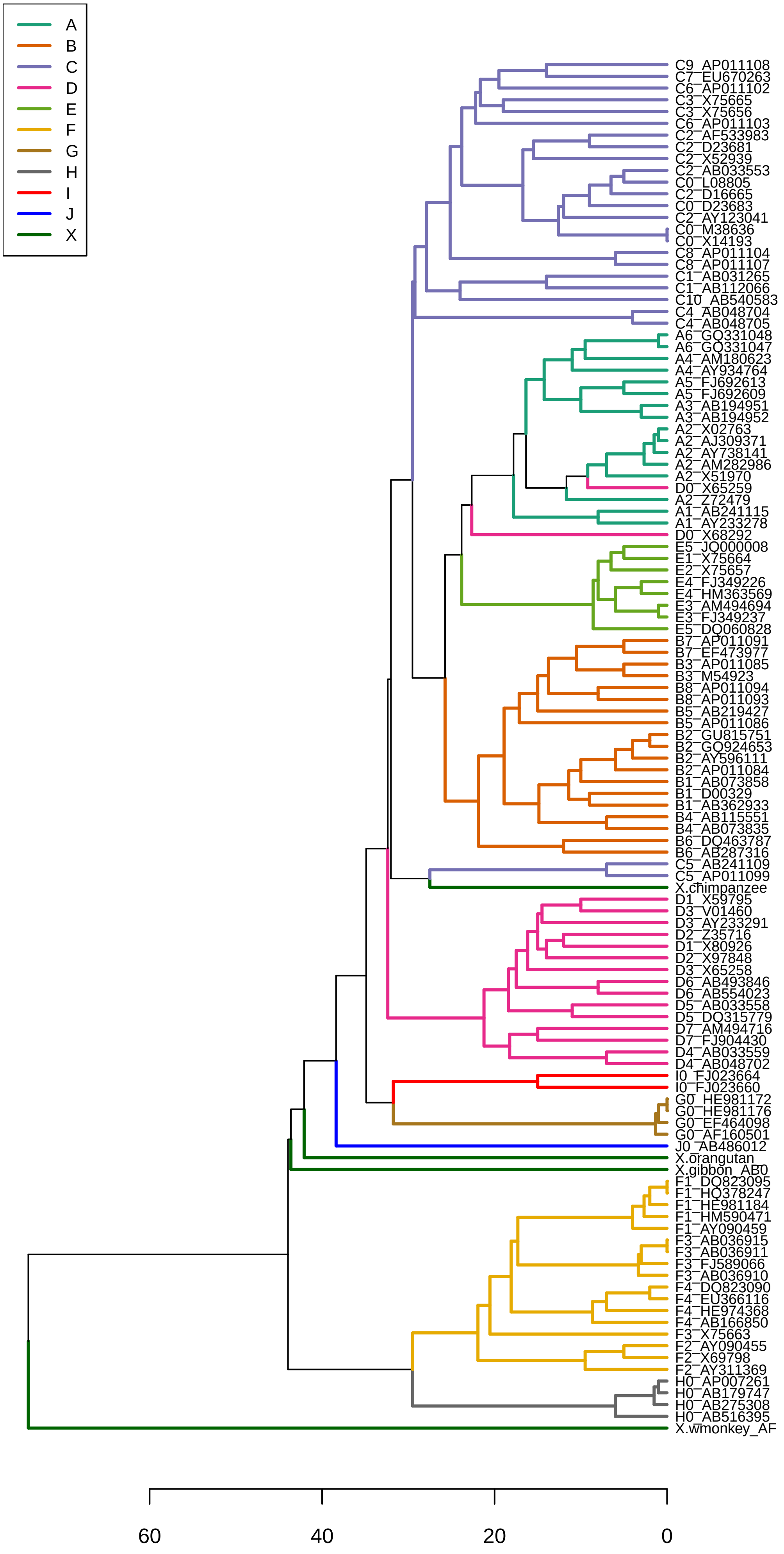

UPGMA tree (N): 1001−1400

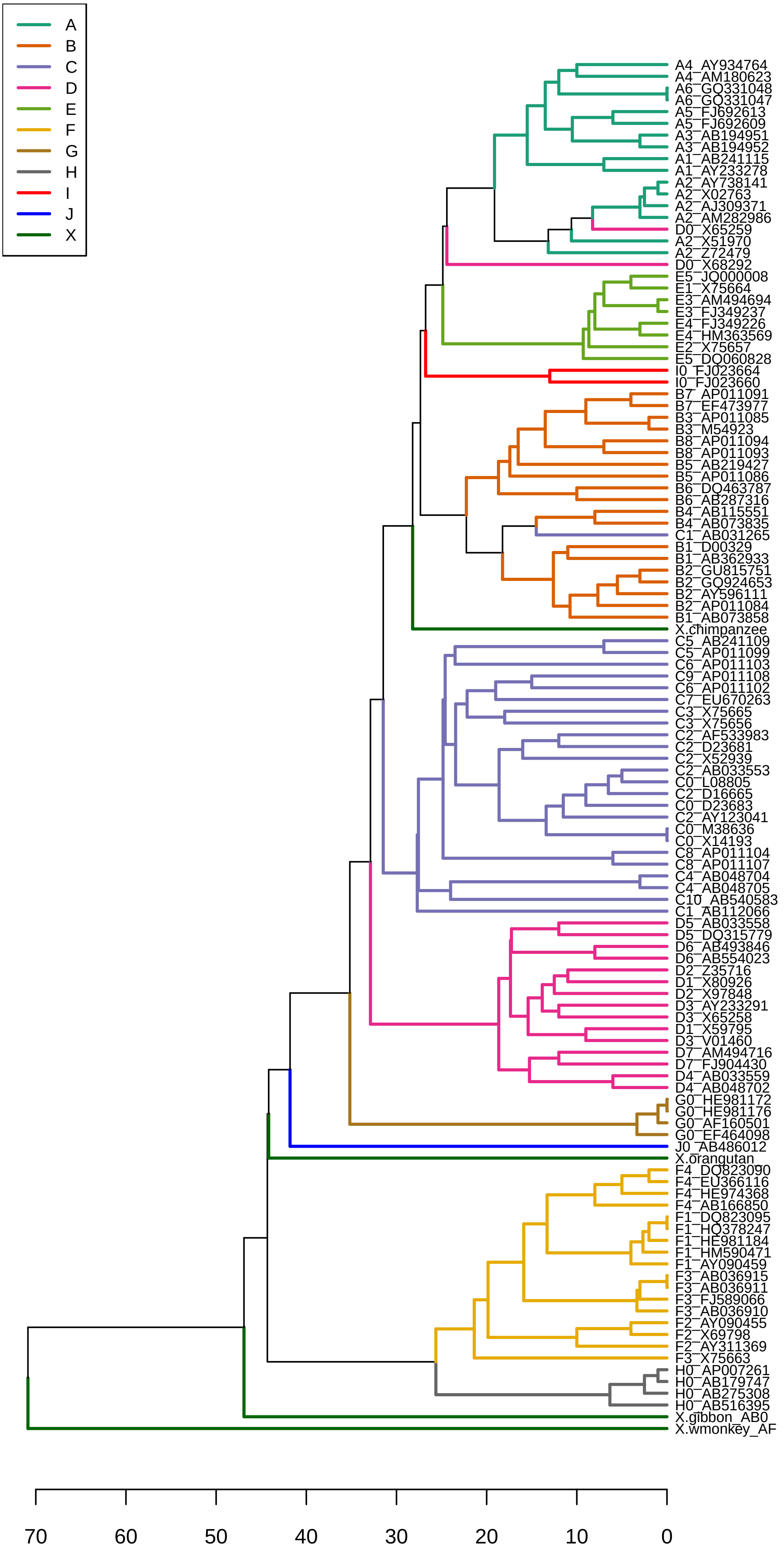

UPGMA tree (N): 1041−1440

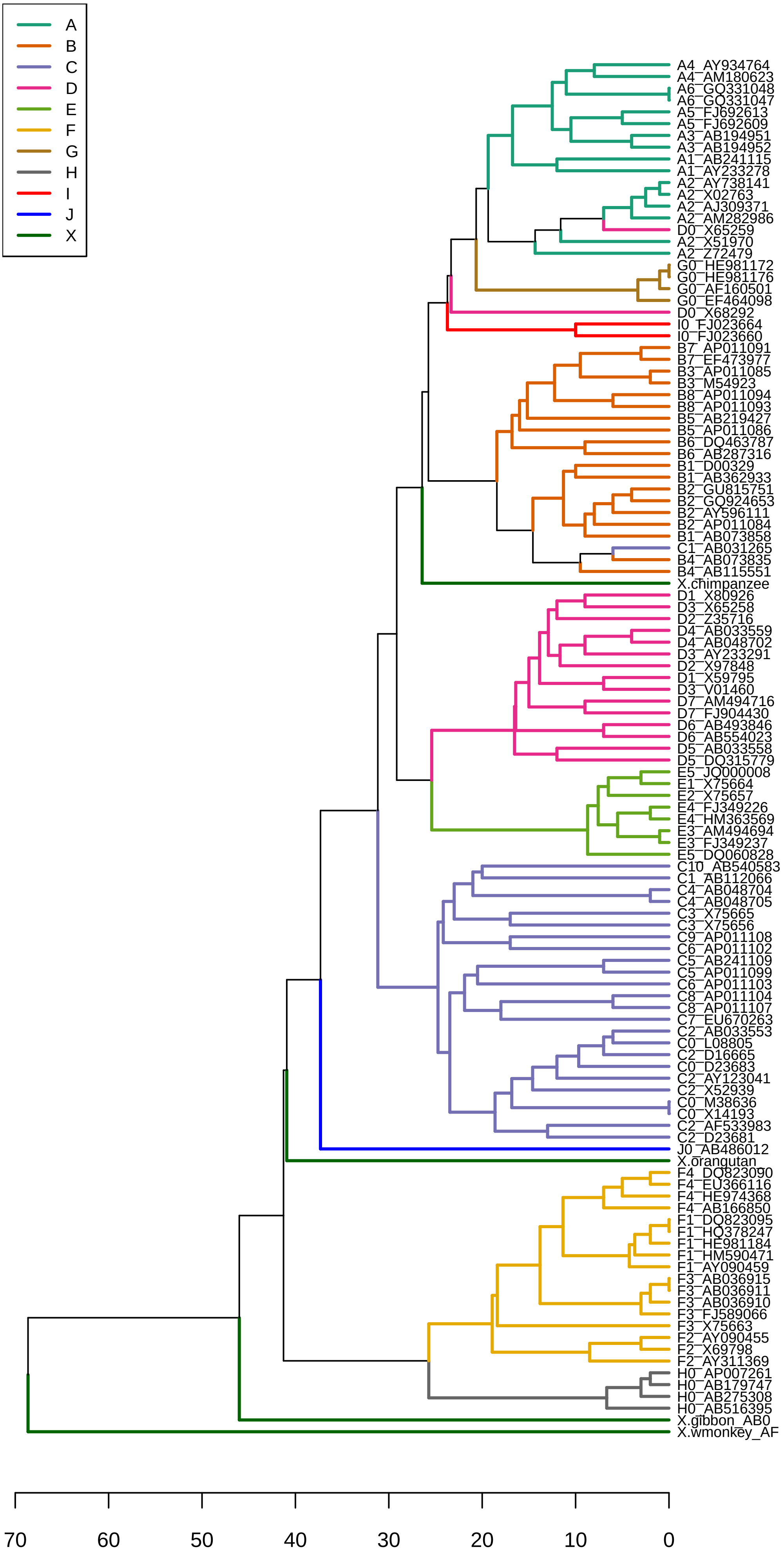

UPGMA tree (N): 1081−1480

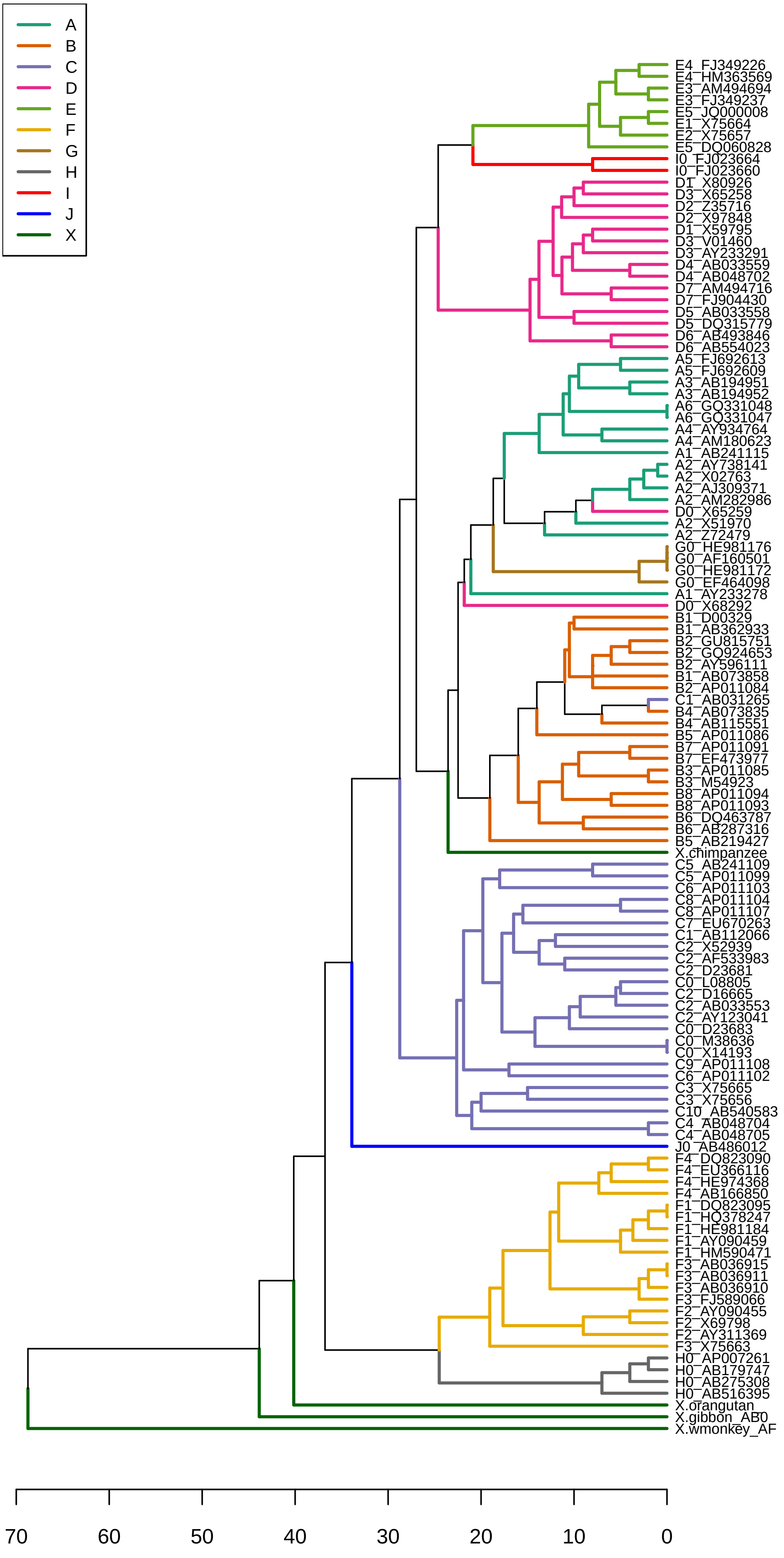

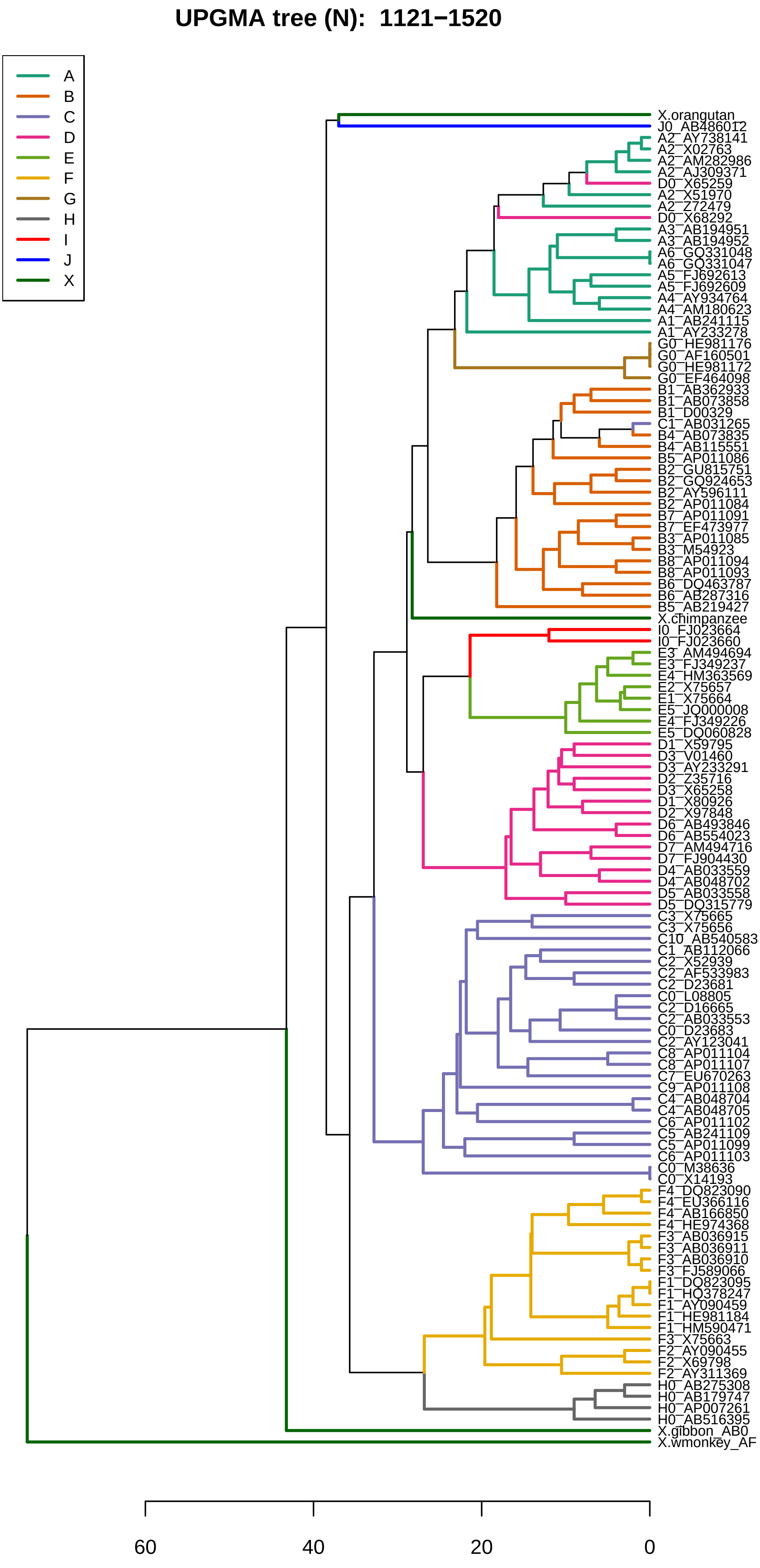
UPGMA tree (N): 1121−1520
A
B
C
D
E
F
G
H
I
J
X
X.orangutan
J0_AB486012
A2_AY738141
A2_X02763
A2_AM282986
A2_AJ309371
D0_X65259
A2_X51970
A2_Z72479
D0_X68292
A3_AB194951
A3_AB194952
A6_GQ331048
A6_GQ331047
A5_FJ692613
A5_FJ692609
A4_AY934764
A4_AM180623
A1_AB241115
A1_AY233278
G0_HE981176
G0_AF160501
G0_HE981172
G0_EF464098
B1_AB362933
B1_AB073858
B1_D00329
C1_AB031265
B4_AB073835
B4_AB115551
B5_AP011086
B2_GU815751
B2_GQ924653
B2_AY596111
B2_AP011084
B7_AP011091
B7_EF473977
B3_AP011085
B3_M54923
B8_AP011094
B8_AP011093
B6_DQ463787
B6_AB287316
B5_AB219427
X.chimpanzee
I0_FJ023664
I0_FJ023660
E3_AM494694
E3_FJ349237
E4_HM363569
E2_X75657
E1_X75664
E5_JQ000008
E4_FJ349226
E5_DQ060828
D1_X59795
D3_V01460
D3_AY233291
D2_Z35716
D3_X65258
D1_X80926
D2_X97848
D6_AB493846
D6_AB554023
D7_AM494716
D7_FJ904430
D4_AB033559
D4_AB048702
D5_AB033558
D5_DQ315779
C3_X75665
C3_X75656
C10_AB540583
C1_AB112066
C2_X52939
C2_AF533983
C2_D23681
C0_L08805
C2_D16665
C2_AB033553
C0_D23683
C2_AY123041
C8_AP011104
C8_AP011107
C7_EU670263
C9_AP011108
C4_AB048704
C4_AB048705
C6_AP011102
C5_AB241109
C5_AP011099
C6_AP011103
C0_M38636
C0_X14193
F4_DQ823090
F4_EU366116
F4_AB166850
F4_HE974368
F3_AB036915
F3_AB036911
F3_AB036910
F3_FJ589066
F1_DQ823095
F1_HQ378247
F1_AY090459
F1_HE981184
F1_HM590471
F3_X75663
F2_AY090455
F2_X69798
F2_AY311369
H0_AB275308
H0_AB179747
H0_AP007261
H0_AB516395
X.gibbon_AB0
X.wmonkey_AF
60
40
20
0

UPGMA tree (N): 1161−1560

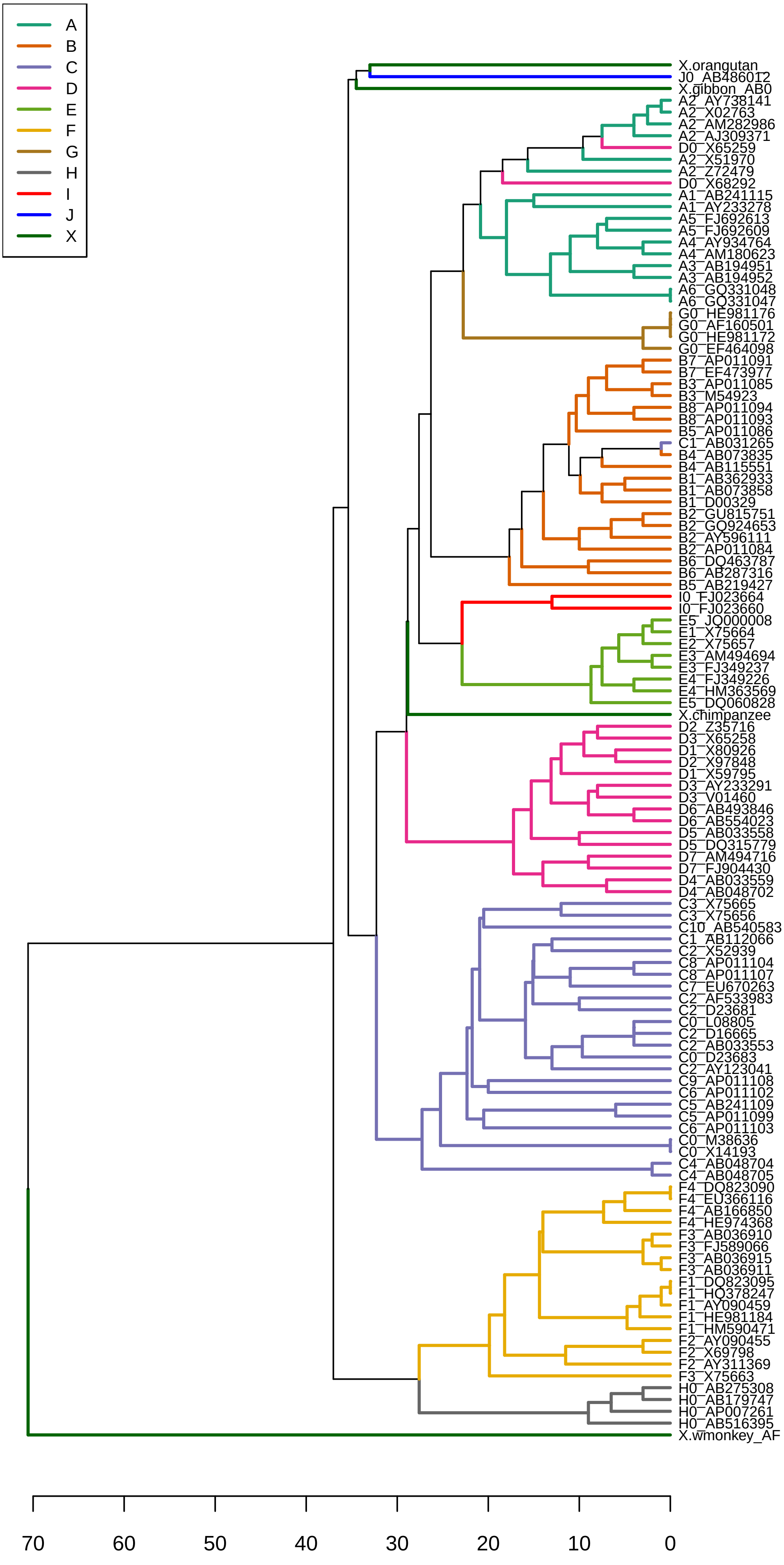

UPGMA tree (N): 1201−1600

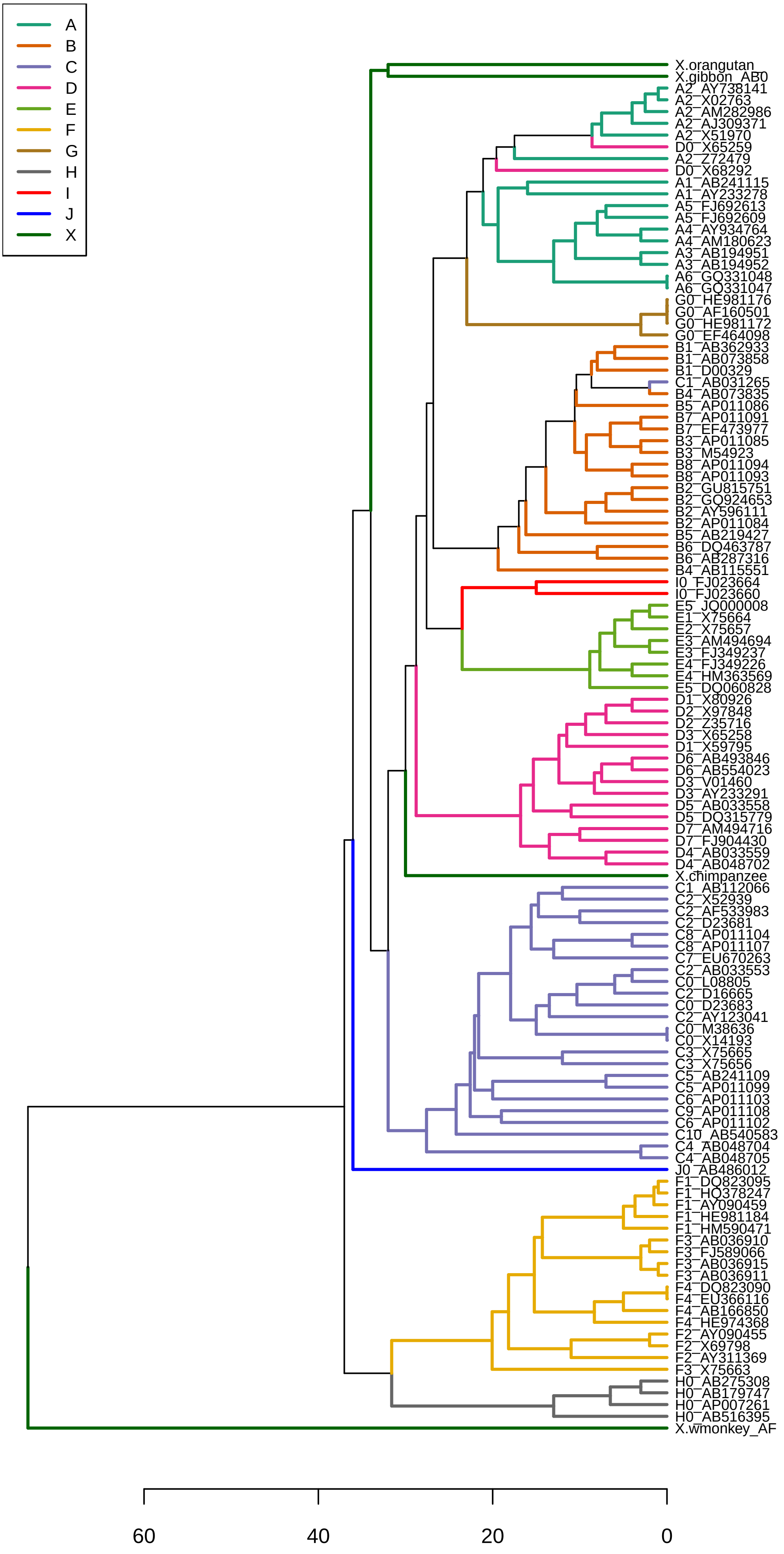

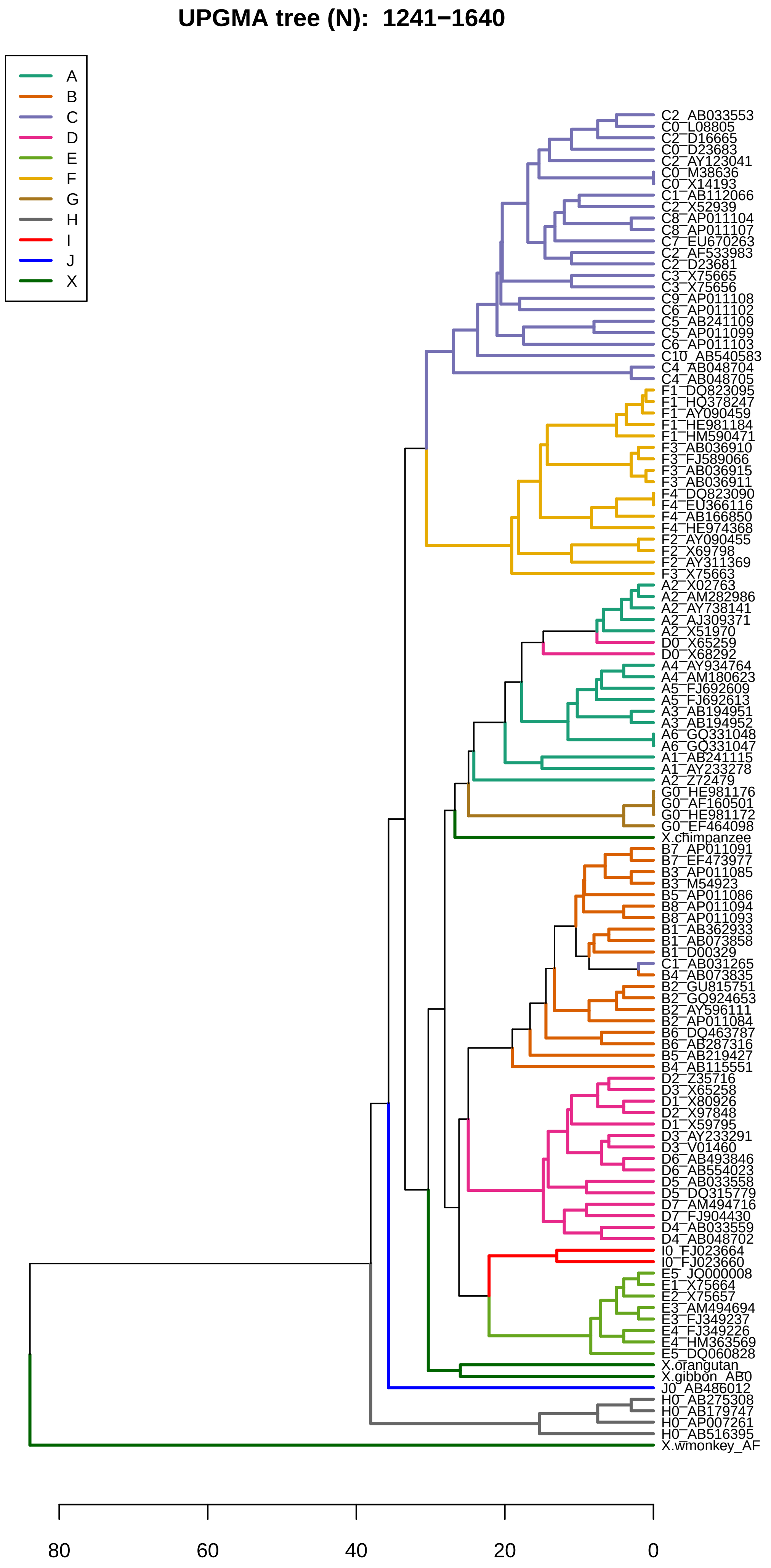

UPGMA tree (N): 1241−1640
A
B
C
D
E
F
G
H
I
J
X
C2_AB033553
C0_L08805
C2_D16665
C0_D23683
C2_AY123041
C0_M38636
C0_X14193
C1_AB112066
C2_X52939
C8_AP011104
C8_AP011107
C7_EU670263
C2_AF533983
C2_D23681
C3_X75665
C3_X75656
C9_AP011108
C6_AP011102
C5_AB241109
C5_AP011099
C6_AP011103
C10_AB540583
C4_AB048704
C4_AB048705
F1_DQ823095
F1_HQ378247
F1_AY090459
F1_HE981184
F1_HM590471
F3_AB036910
F3_FJ589066
F3_AB036915
F3_AB036911
F4_DQ823090
F4_EU366116
F4_AB166850
F4_HE974368
F2_AY090455
F2_X69798
F2_AY311369
F3_X75663
A2_X02763
A2_AM282986
A2_AY738141
A2_AJ309371
A2_X51970
D0_X65259
D0_X68292
A4_AY934764
A4_AM180623
A5_FJ692609
A5_FJ692613
A3_AB194951
A3_AB194952
A6_GQ331048
A6_GQ331047
A1_AB241115
A1_AY233278
A2_Z72479
G0_HE981176
G0_AF160501
G0_HE981172
G0_EF464098
X.chimpanzee
B7_AP011091
B7_EF473977
B3_AP011085
B3_M54923
B5_AP011086
B8_AP011094
B8_AP011093
B1_AB362933
B1_AB073858
B1_D00329
C1_AB031265
B4_AB073835
B2_GU815751
B2_GQ924653
B2_AY596111
B2_AP011084
B6_DQ463787
B6_AB287316
B5_AB219427
B4_AB115551
D2_Z35716
D3_X65258
D1_X80926
D2_X97848
D1_X59795
D3_AY233291
D3_V01460
D6_AB493846
D6_AB554023
D5_AB033558
D5_DQ315779
D7_AM494716
D7_FJ904430
D4_AB033559
D4_AB048702
I0_FJ023664
I0_FJ023660
E5_JQ000008
E1_X75664
E2_X75657
E3_AM494694
E3_FJ349237
E4_FJ349226
E4_HM363569
E5_DQ060828
X.orangutan
X.gibbon_AB0
J0_AB486012
H0_AB275308
H0_AB179747
H0_AP007261
H0_AB516395
X.wmonkey_AF
80
60
40
20
0

UPGMA tree (N): 1281−1680

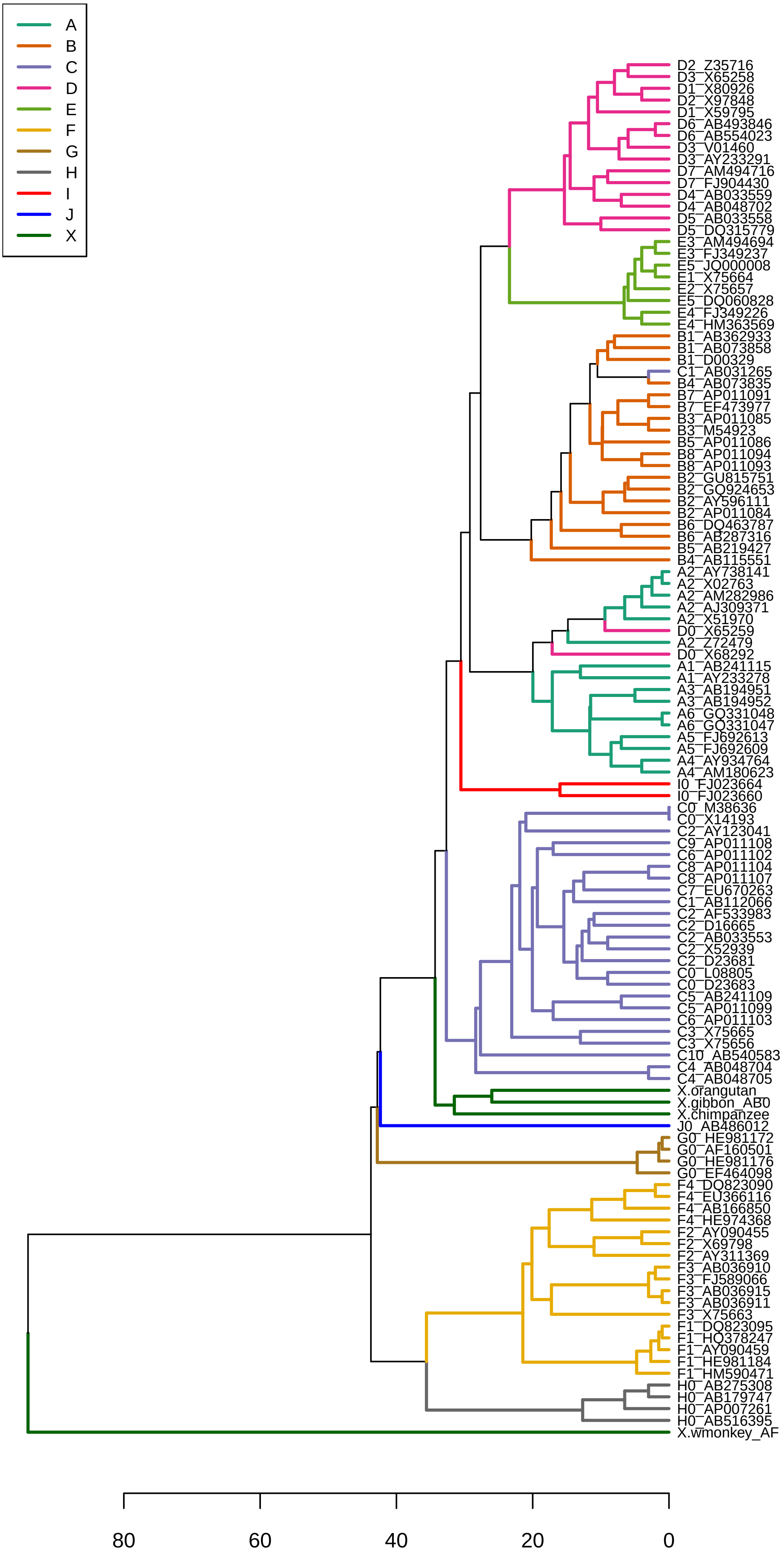

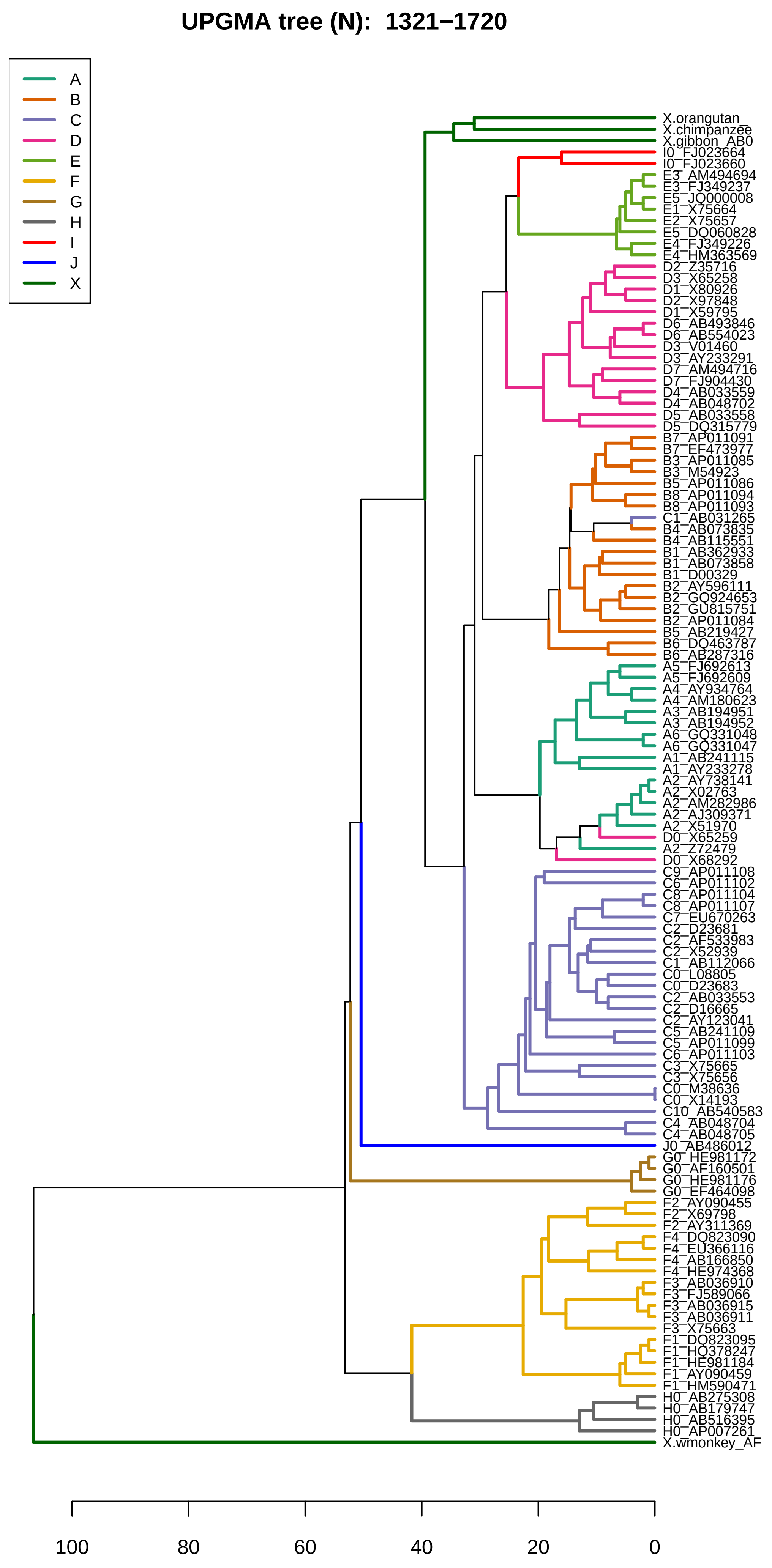
UPGMA tree (N): 1321−1720
A
B
C
D
E
F
G
H
I
J
X
X.orangutan_
X.chimpanzee
X.gibbon_AB0
I0_FJ023664
I0_FJ023660
E3_AM494694
E3_FJ349237
E5_JQ000008
E1_X75664
E2_X75657
E5_DQ060828
E4_FJ349226
E4_HM363569
D2_Z35716
D3_X65258
D1_X80926
D2_X97848
D1_X59795
D6_AB493846
D6_AB554023
D3_V01460
D3_AY233291
D7_AM494716
D7_FJ904430
D4_AB033559
D4_AB048702
D5_AB033558
D5_DQ315779
B7_AP011091
B7_EF473977
B3_AP011085
B3_M54923
B5_AP011086
B8_AP011094
B8_AP011093
C1_AB031265
B4_AB073835
B4_AB115551
B1_AB362933
B1_AB073858
B1_D00329
B2_AY596111
B2_GQ924653
B2_GU815751
B2_AP011084
B5_AB219427
B6_DQ463787
B6_AB287316
A5_FJ692613
A5_FJ692609
A4_AY934764
A4_AM180623
A3_AB194951
A3_AB194952
A6_GQ331048
A6_GQ331047
A1_AB241115
A1_AY233278
A2_AY738141
A2_X02763
A2_AM282986
A2_AJ309371
A2_X51970
D0_X65259
A2_Z72479
D0_X68292
C9_AP011108
C6_AP011102
C8_AP011104
C8_AP011107
C7_EU670263
C2_D23681
C2_AF533983
C2_X52939
C1_AB112066
C0_L08805
C0_D23683
C2_AB033553
C2_D16665
C2_AY123041
C5_AB241109
C5_AP011099
C6_AP011103
C3_X75665
C3_X75656
C0_M38636
C0_X14193
C10_AB540583
C4_AB048704
C4_AB048705
J0_AB486012
G0_HE981172
G0_AF160501
G0_HE981176
G0_EF464098
F2_AY090455
F2_X69798
F2_AY311369
F4_DQ823090
F4_EU366116
F4_AB166850
F4_HE974368
F3_AB036910
F3_FJ589066
F3_AB036915
F3_AB036911
F3_X75663
F1_DQ823095
F1_HQ378247
F1_HE981184
F1_AY090459
F1_HM590471
H0_AB275308
H0_AB179747
H0_AB516395
H0_AP007261
X.wmonkey_AF
100
80
60
40
20
0

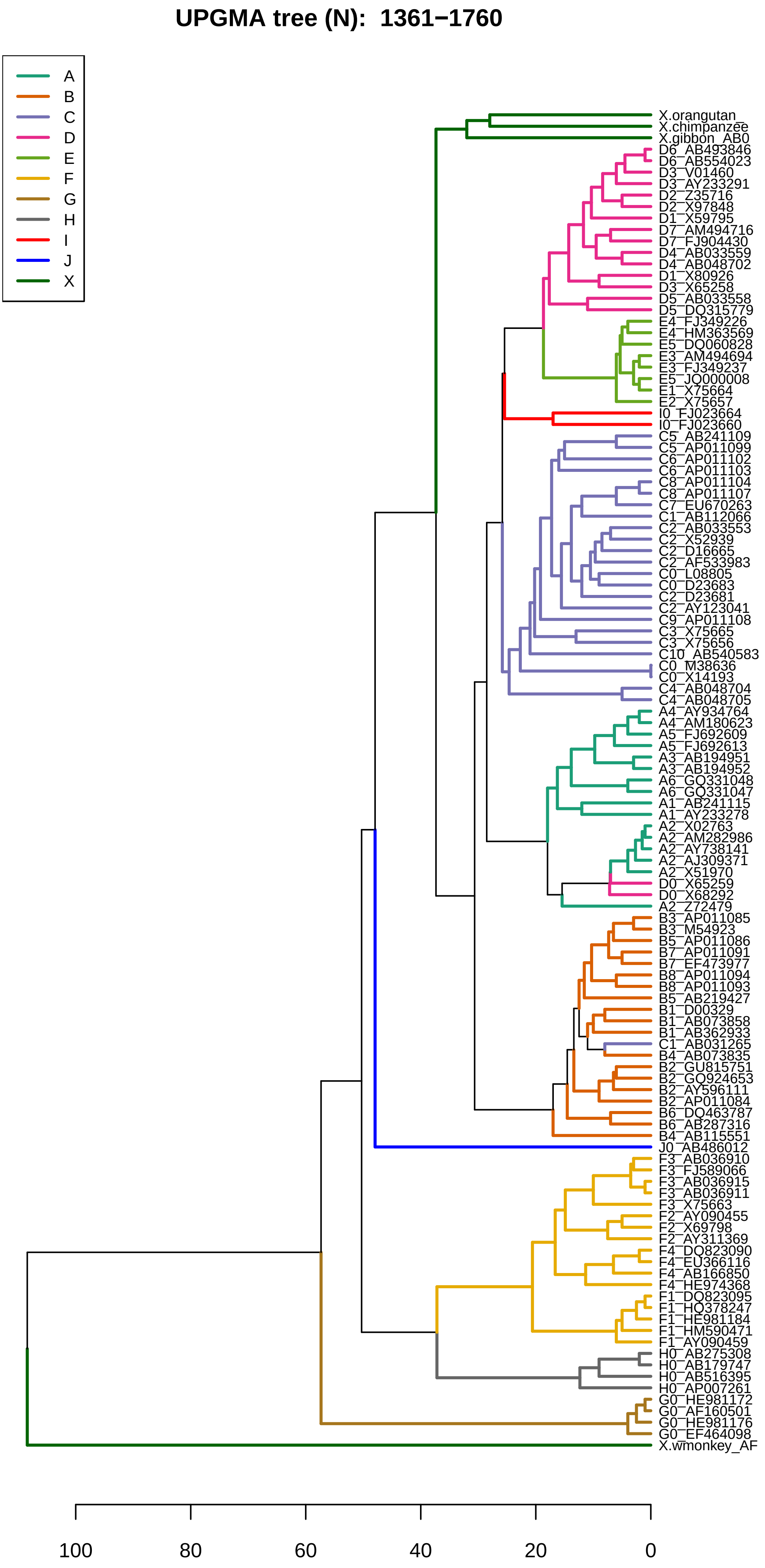

UPGMA tree (N): 1361−1760
A
B
C
D
E
F
G
H
I
J
X
X.orangutan_
X.chimpanzee
X.gibbon_AB0
D6_AB493846
D6_AB554023
D3_V01460
D3_AY233291
D2_Z35716
D2_X97848
D1_X59795
D7_AM494716
D7_FJ904430
D4_AB033559
D4_AB048702
D1_X80926
D3_X65258
D5_AB033558
D5_DQ315779
E4_FJ349226
E4_HM363569
E5_DQ060828
E3_AM494694
E3_FJ349237
E5_JQ000008
E1_X75664
E2_X75657
I0_FJ023664
I0_FJ023660
C5_AB241109
C5_AP011099
C6_AP011102
C6_AP011103
C8_AP011104
C8_AP011107
C7_EU670263
C1_AB112066
C2_AB033553
C2_X52939
C2_D16665
C2_AF533983
C0_L08805
C0_D23683
C2_D23681
C2_AY123041
C9_AP011108
C3_X75665
C3_X75656
C10_AB540583
C0_M38636
C0_X14193
C4_AB048704
C4_AB048705
A4_AY934764
A4_AM180623
A5_FJ692609
A5_FJ692613
A3_AB194951
A3_AB194952
A6_GQ331048
A6_GQ331047
A1_AB241115
A1_AY233278
A2_X02763
A2_AM282986
A2_AY738141
A2_AJ309371
A2_X51970
D0_X65259
D0_X68292
A2_Z72479
B3_AP011085
B3_M54923
B5_AP011086
B7_AP011091
B7_EF473977
B8_AP011094
B8_AP011093
B5_AB219427
B1_D00329
B1_AB073858
B1_AB362933
C1_AB031265
B4_AB073835
B2_GU815751
B2_GQ924653
B2_AY596111
B2_AP011084
B6_DQ463787
B6_AB287316
B4_AB115551
J0_AB486012
F3_AB036910
F3_FJ589066
F3_AB036915
F3_AB036911
F3_X75663
F2_AY090455
F2_X69798
F2_AY311369
F4_DQ823090
F4_EU366116
F4_AB166850
F4_HE974368
F1_DQ823095
F1_HQ378247
F1_HE981184
F1_HM590471
F1_AY090459
H0_AB275308
H0_AB179747
H0_AB516395
H0_AP007261
G0_HE981172
G0_AF160501
G0_HE981176
G0_EF464098
X.wmonkey_AF
100
80
60
40
20
0

UPGMA tree (N): 1401−1800

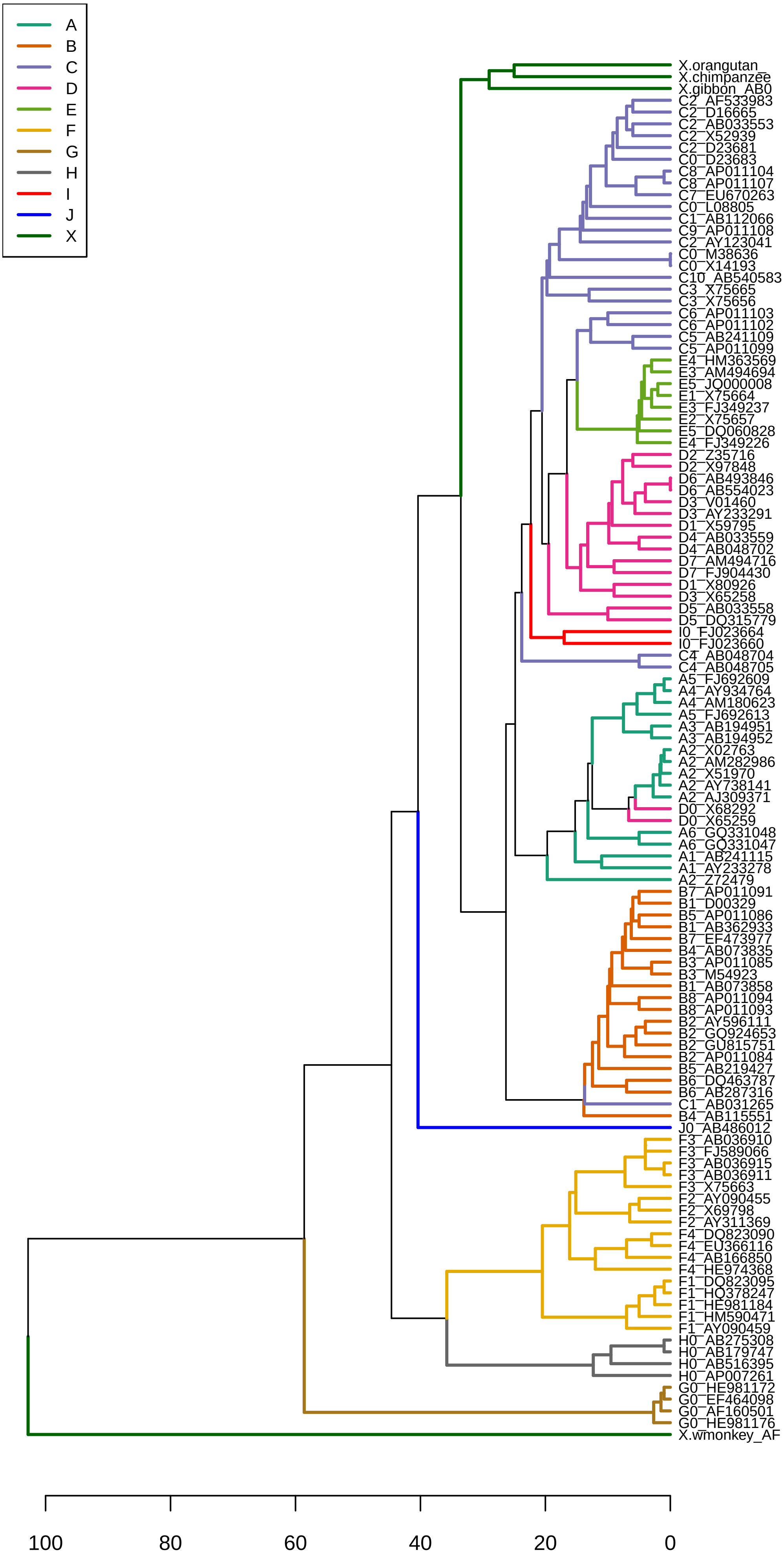

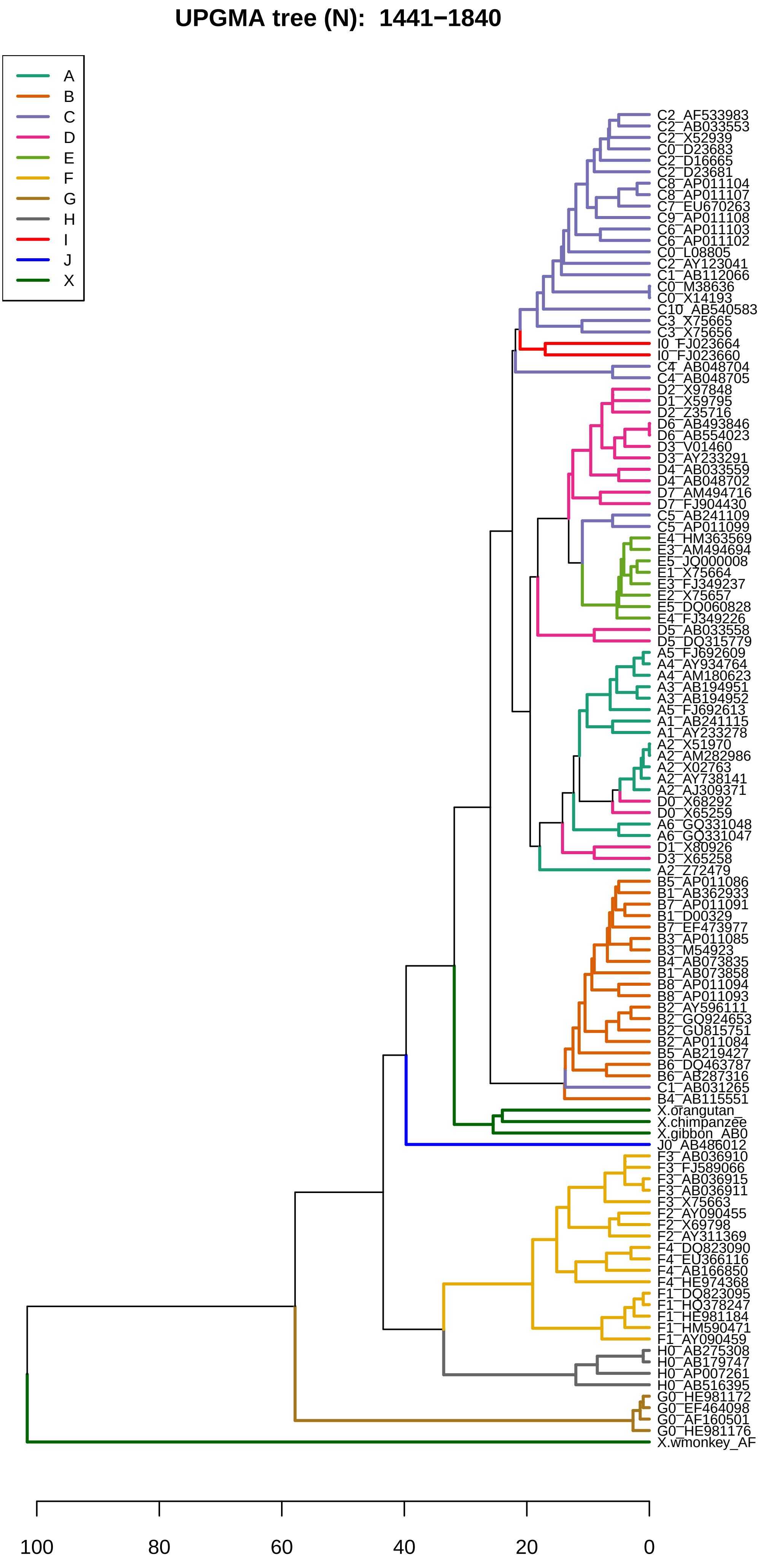
UPGMA tree (N): 1441−1840
A
B
C
D
E
F
G
H
I
J
X
C2_AF533983
C2_AB033553
C2_X52939
C0_D23683
C2_D16665
C2_D23681
C8_AP011104
C8_AP011107
C7_EU670263
C9_AP011108
C6_AP011103
C6_AP011102
C0_L08805
C2_AY123041
C1_AB112066
C0_M38636
C0_X14193
C10_AB540583
C3_X75665
C3_X75656
I0_FJ023664
I0_FJ023660
C4_AB048704
C4_AB048705
D2_X97848
D1_X59795
D2_Z35716
D6_AB493846
D6_AB554023
D3_V01460
D3_AY233291
D4_AB033559
D4_AB048702
D7_AM494716
D7_FJ904430
C5_AB241109
C5_AP011099
E4_HM363569
E3_AM494694
E5_JQ000008
E1_X75664
E3_FJ349237
E2_X75657
E5_DQ060828
E4_FJ349226
D5_AB033558
D5_DQ315779
A5_FJ692609
A4_AY934764
A4_AM180623
A3_AB194951
A3_AB194952
A5_FJ692613
A1_AB241115
A1_AY233278
A2_X51970
A2_AM282986
A2_X02763
A2_AY738141
A2_AJ309371
D0_X68292
D0_X65259
A6_GQ331048
A6_GQ331047
D1_X80926
D3_X65258
A2_Z72479
B5_AP011086
B1_AB362933
B7_AP011091
B1_D00329
B7_EF473977
B3_AP011085
B3_M54923
B4_AB073835
B1_AB073858
B8_AP011094
B8_AP011093
B2_AY596111
B2_GQ924653
B2_GU815751
B2_AP011084
B5_AB219427
B6_DQ463787
B6_AB287316
C1_AB031265
B4_AB115551
X.orangutan_
X.chimpanzee
X.gibbon_AB0
J0_AB486012
F3_AB036910
F3_FJ589066
F3_AB036915
F3_AB036911
F3_X75663
F2_AY090455
F2_X69798
F2_AY311369
F4_DQ823090
F4_EU366116
F4_AB166850
F4_HE974368
F1_DQ823095
F1_HQ378247
F1_HE981184
F1_HM590471
F1_AY090459
H0_AB275308
H0_AB179747
H0_AP007261
H0_AB516395
G0_HE981172
G0_EF464098
G0_AF160501
G0_HE981176
X.wmonkey_AF
100
80
60
40
20
0

UPGMA tree (N): 1481−1880

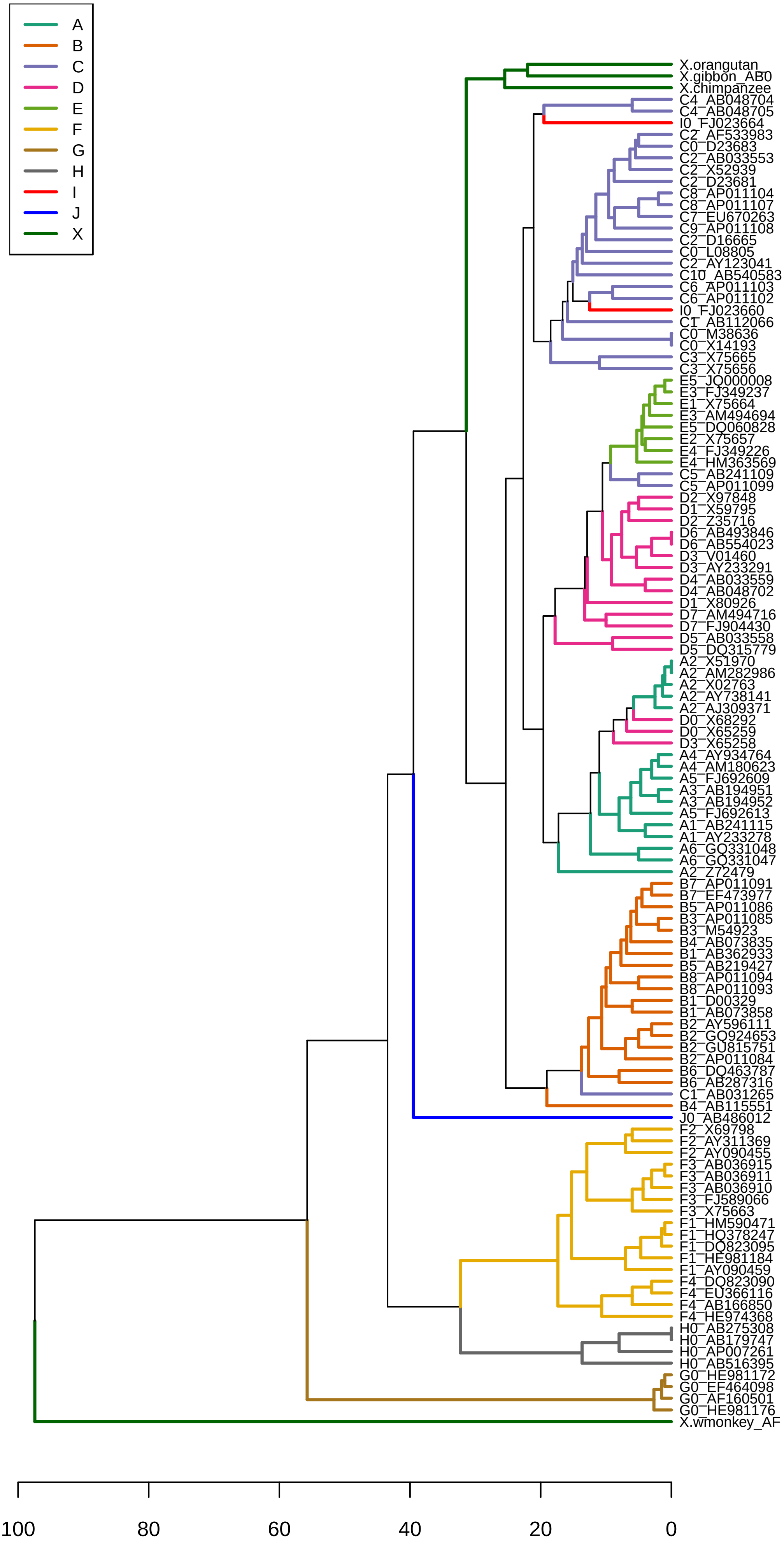

UPGMA tree (N): 1521−1920

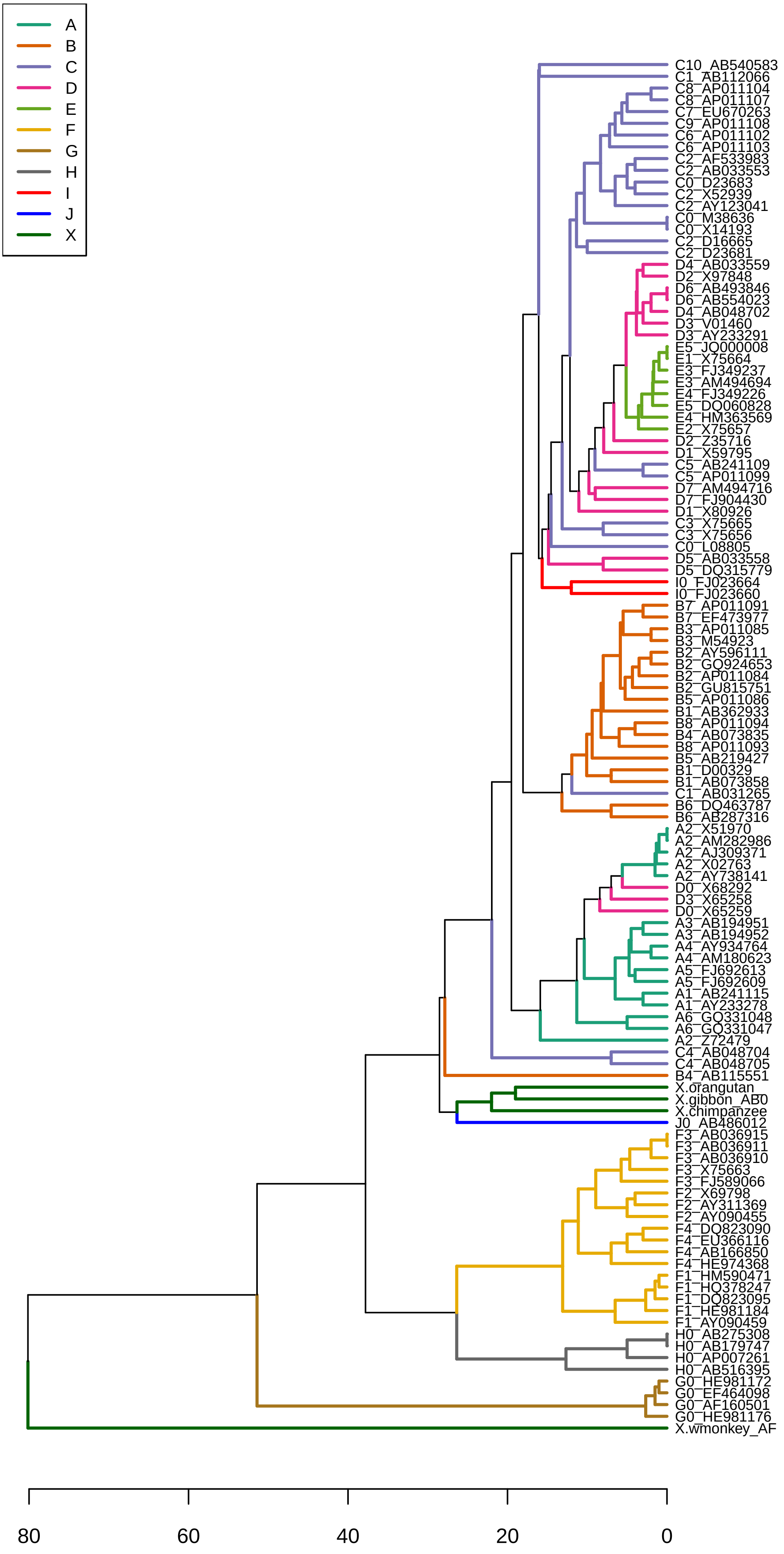

UPGMA tree (N): 1561−1960

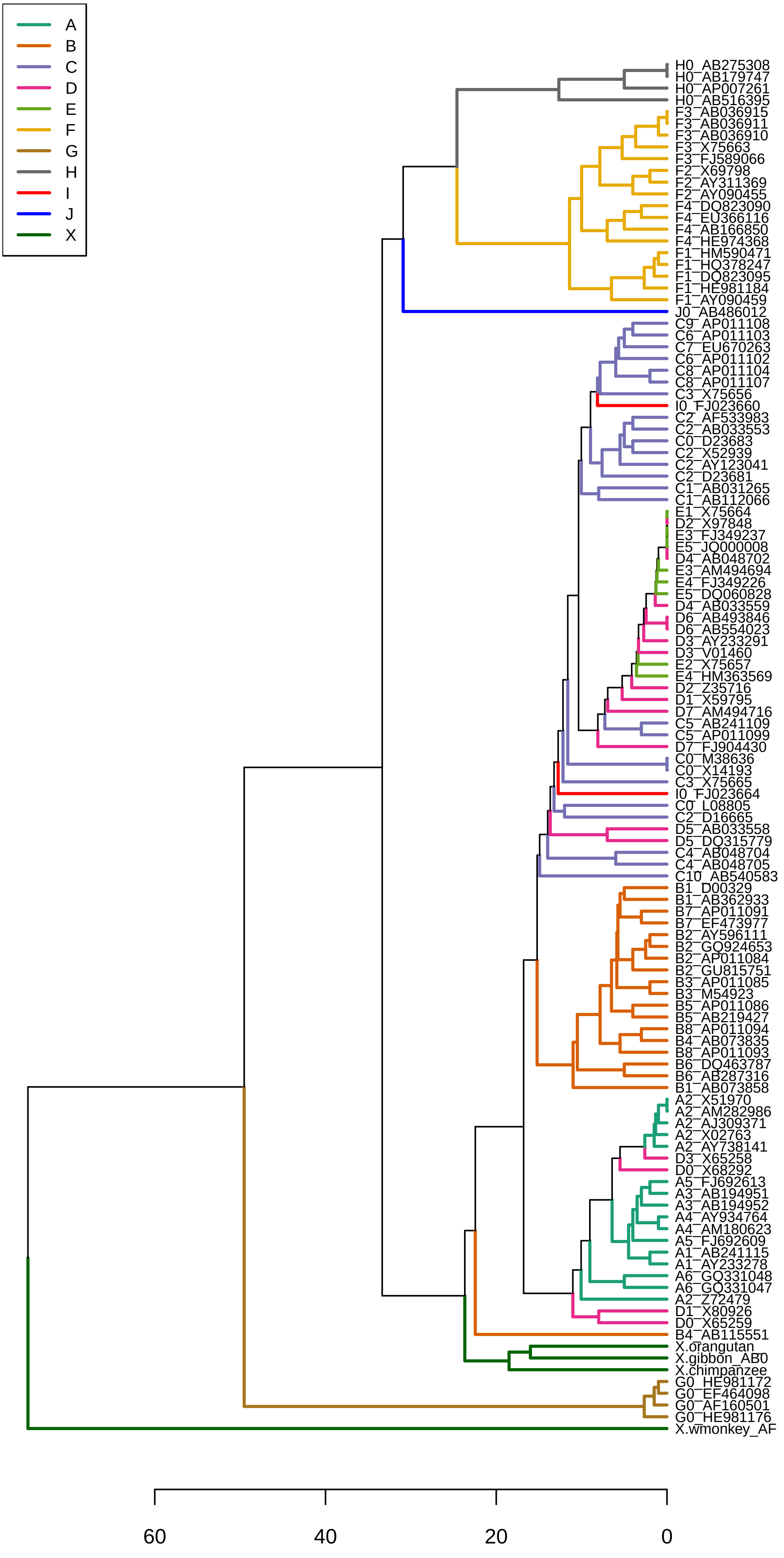

UPGMA tree (N): 1601−2000

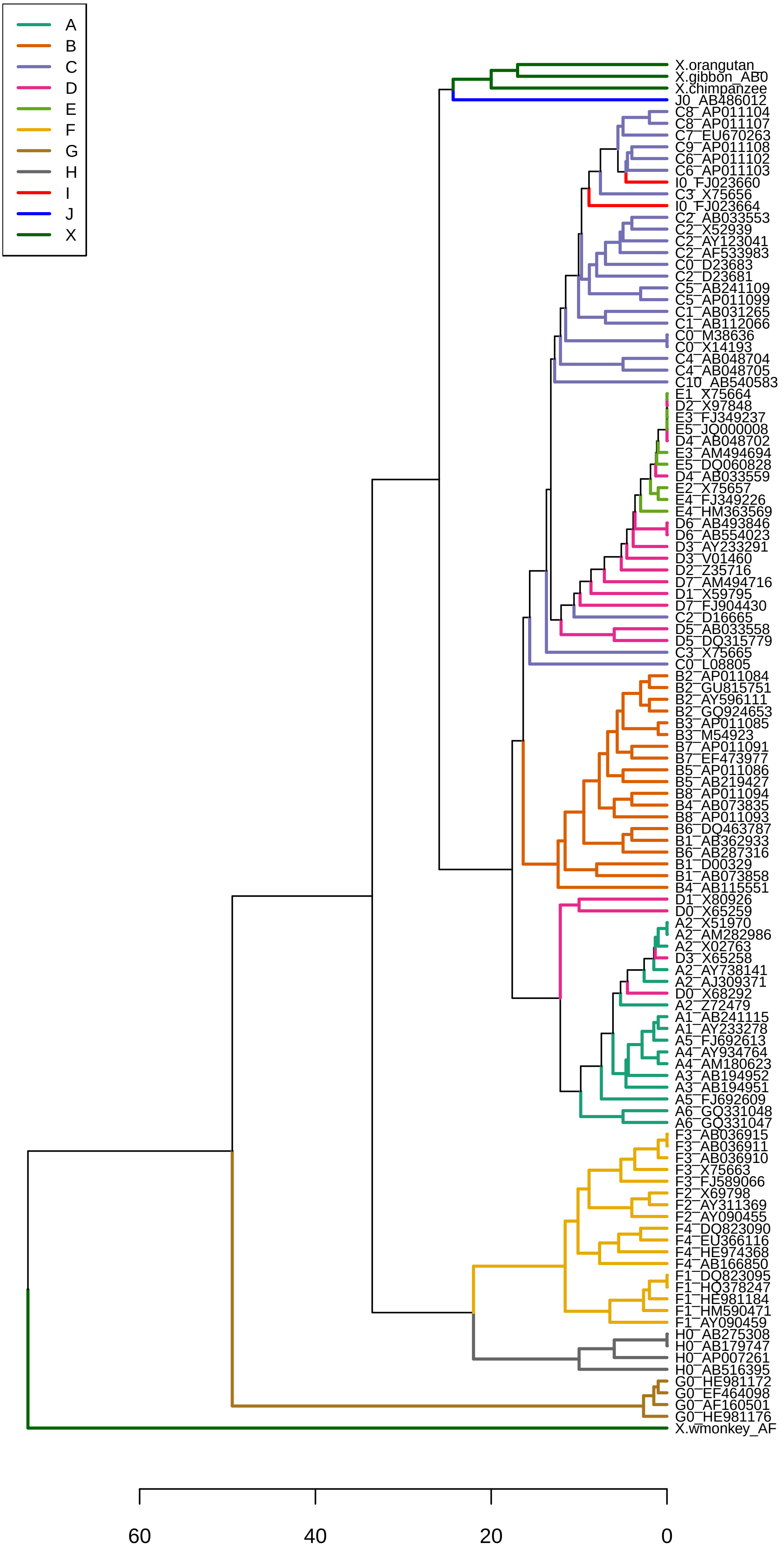

UPGMA tree (N): 1641−2040

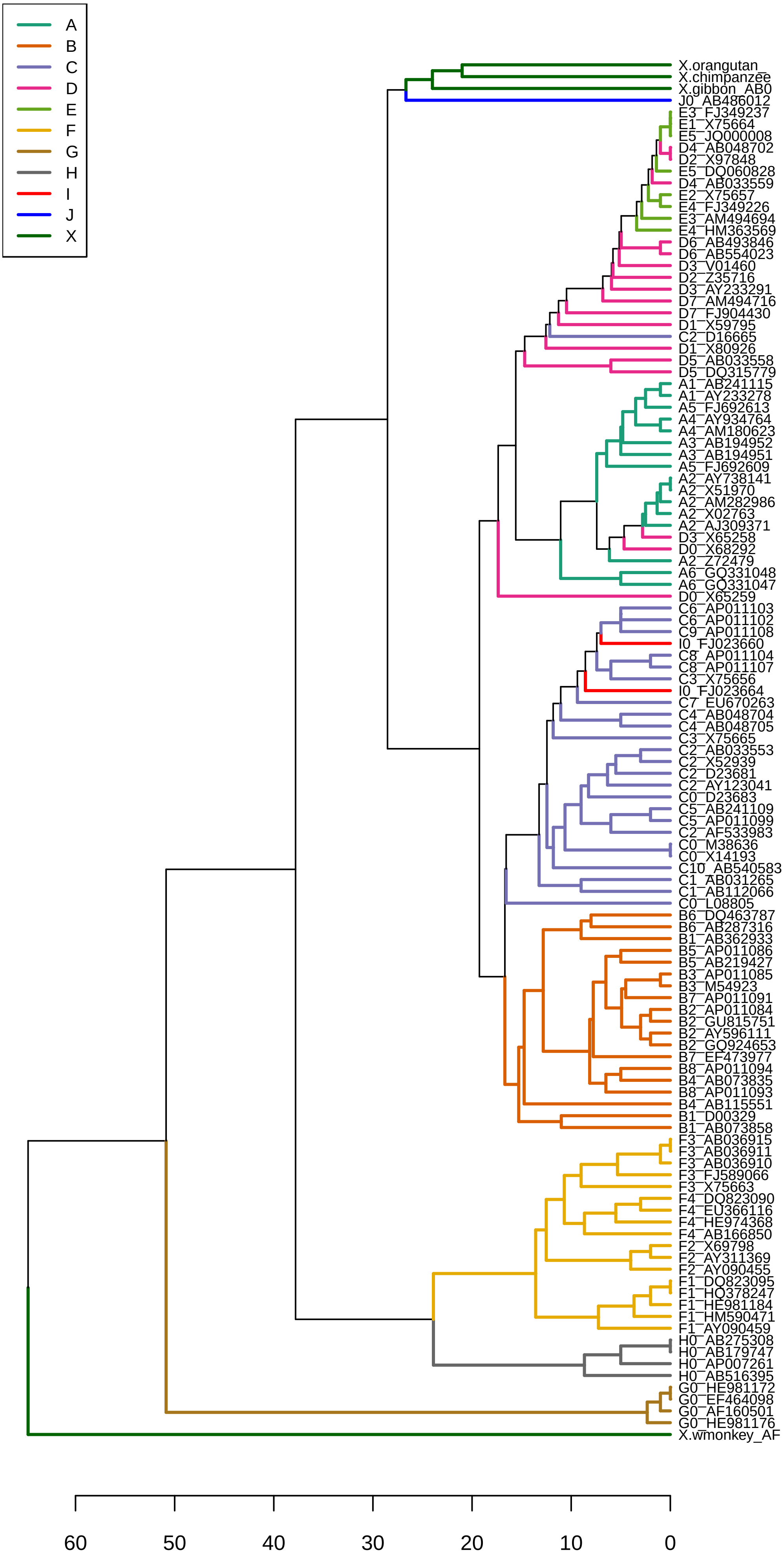

UPGMA tree (N): 1681−2080

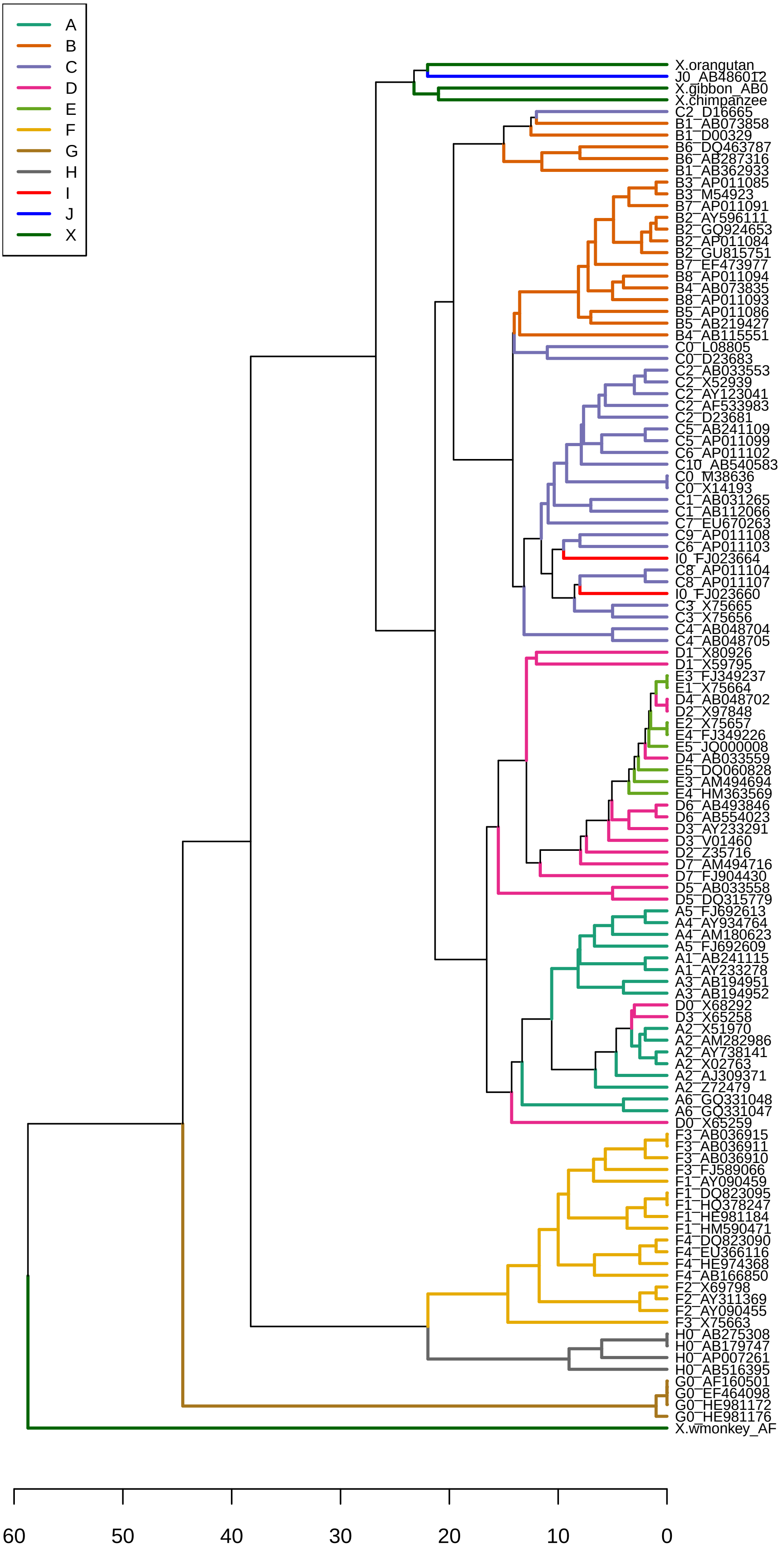

UPGMA tree (N): 1721−2120

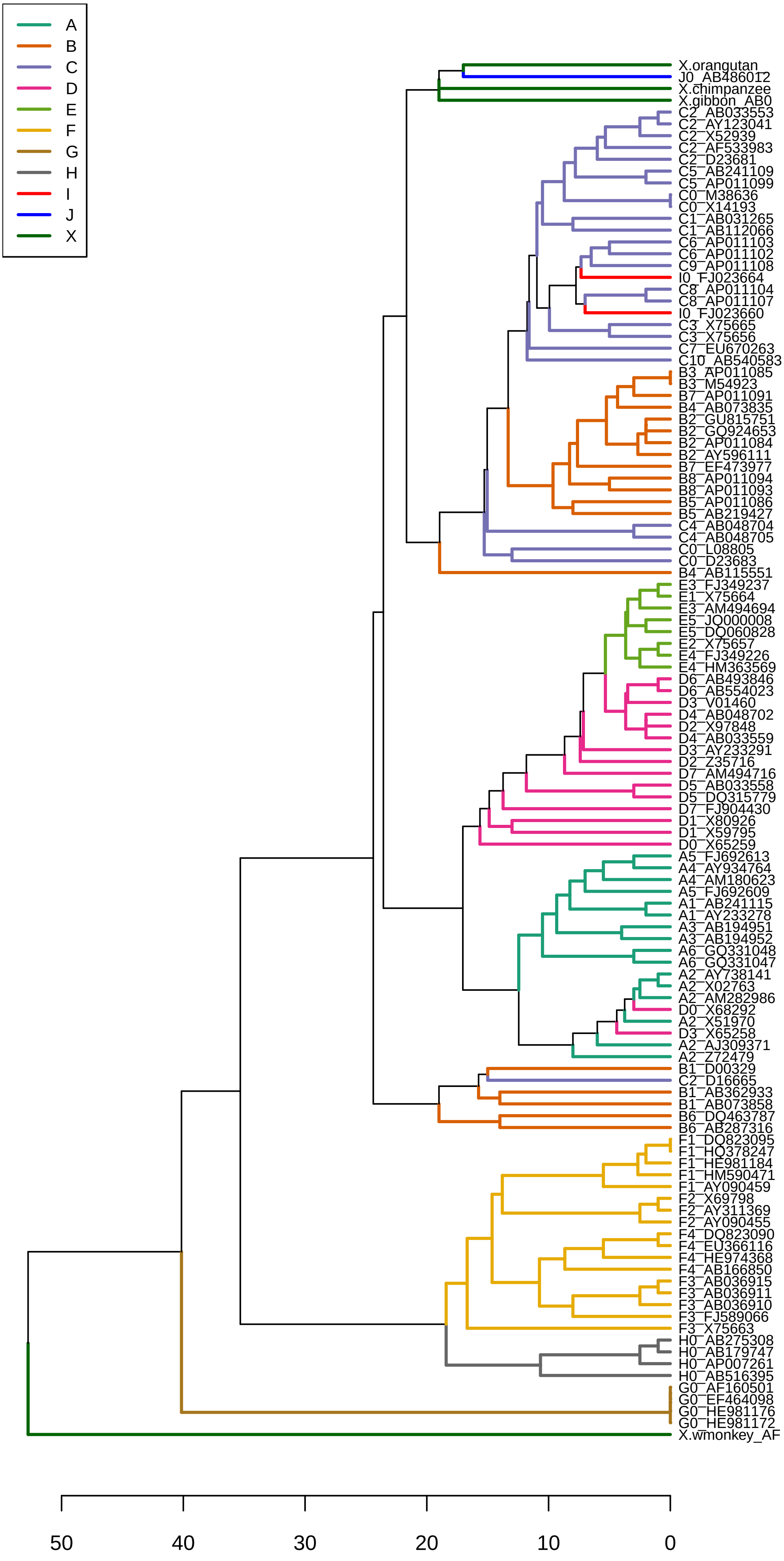

UPGMA tree (N): 1761−2160

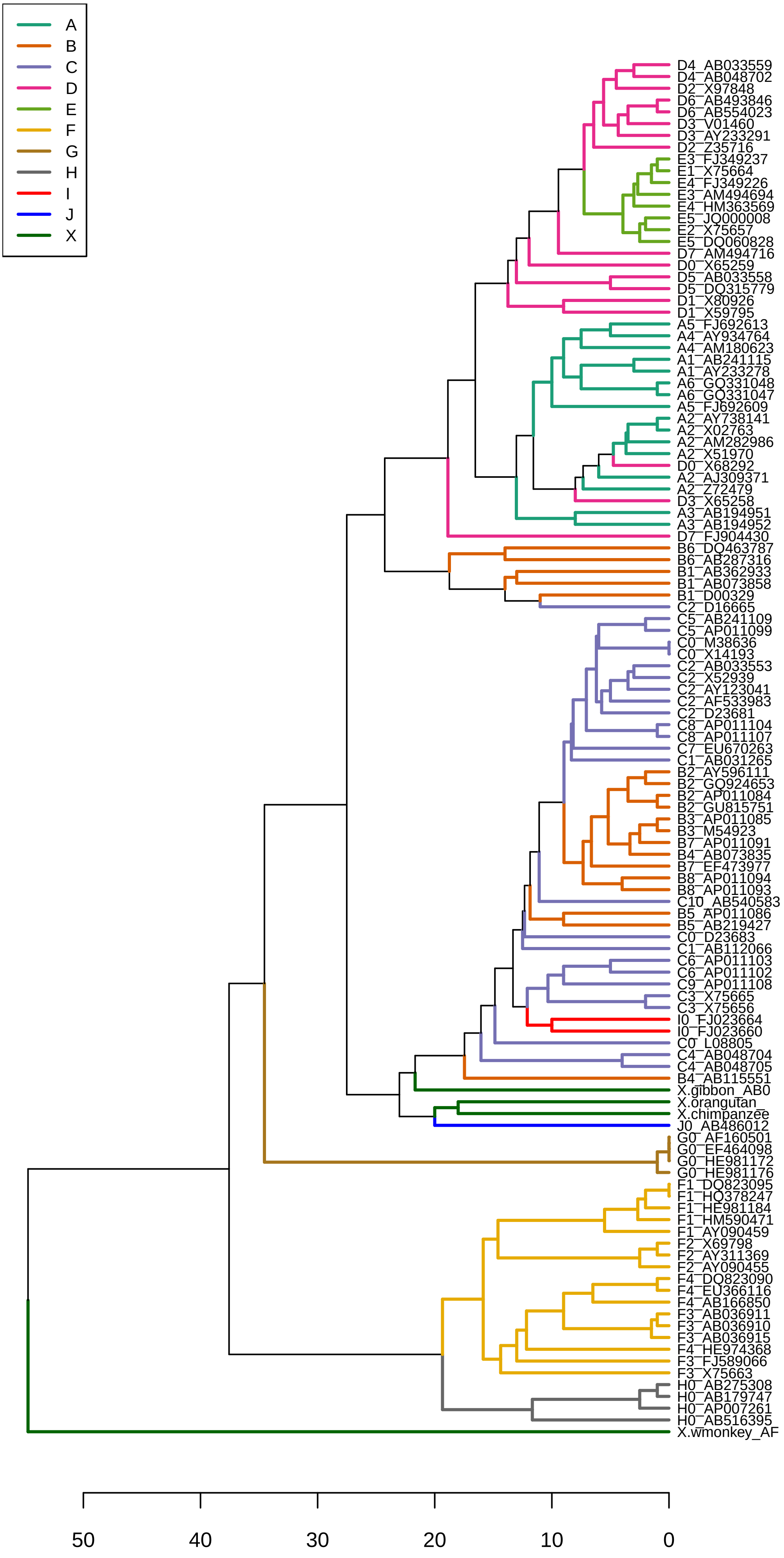

UPGMA tree (N): 1801−2200

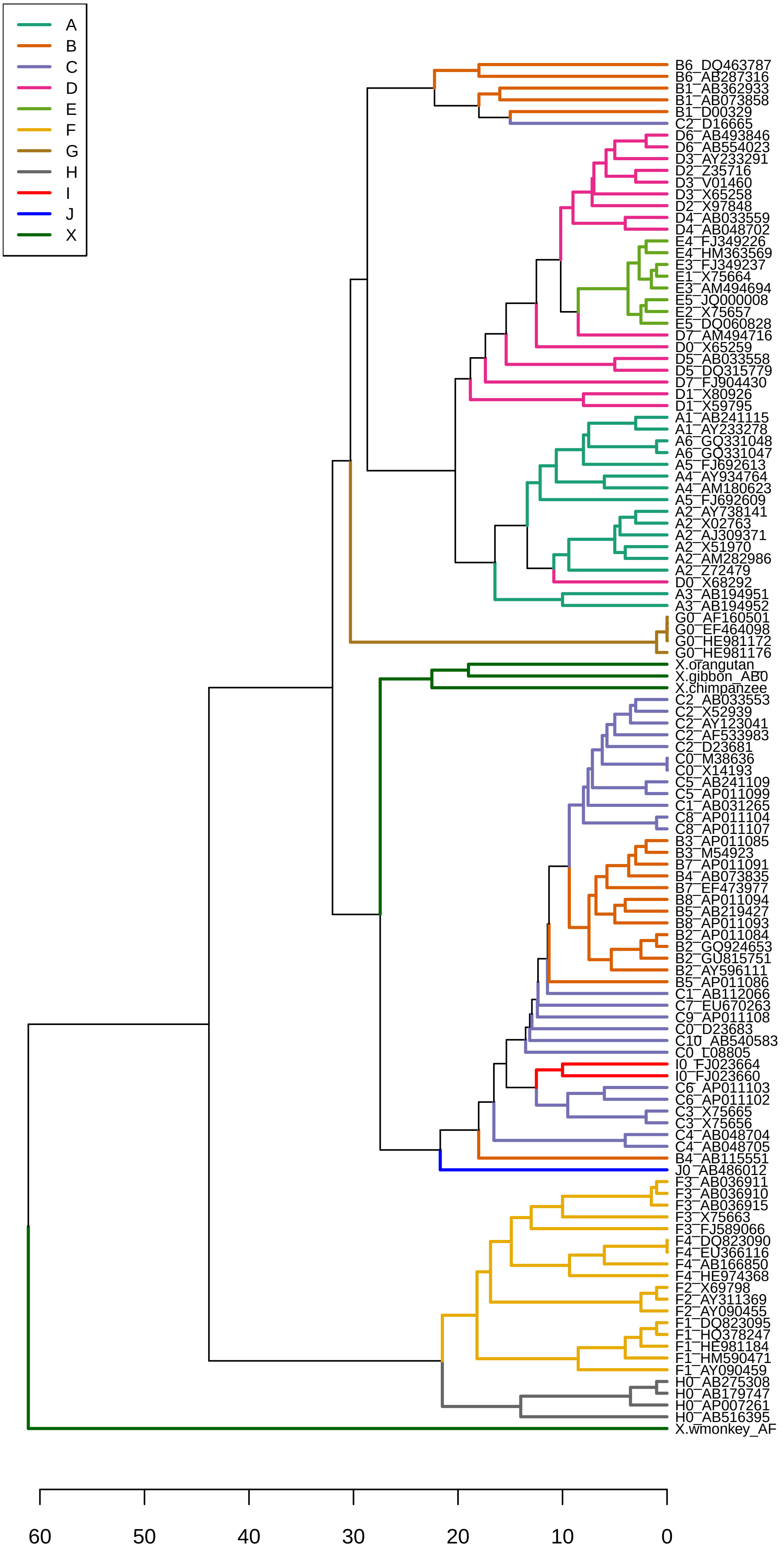

UPGMA tree (N): 1841−2240

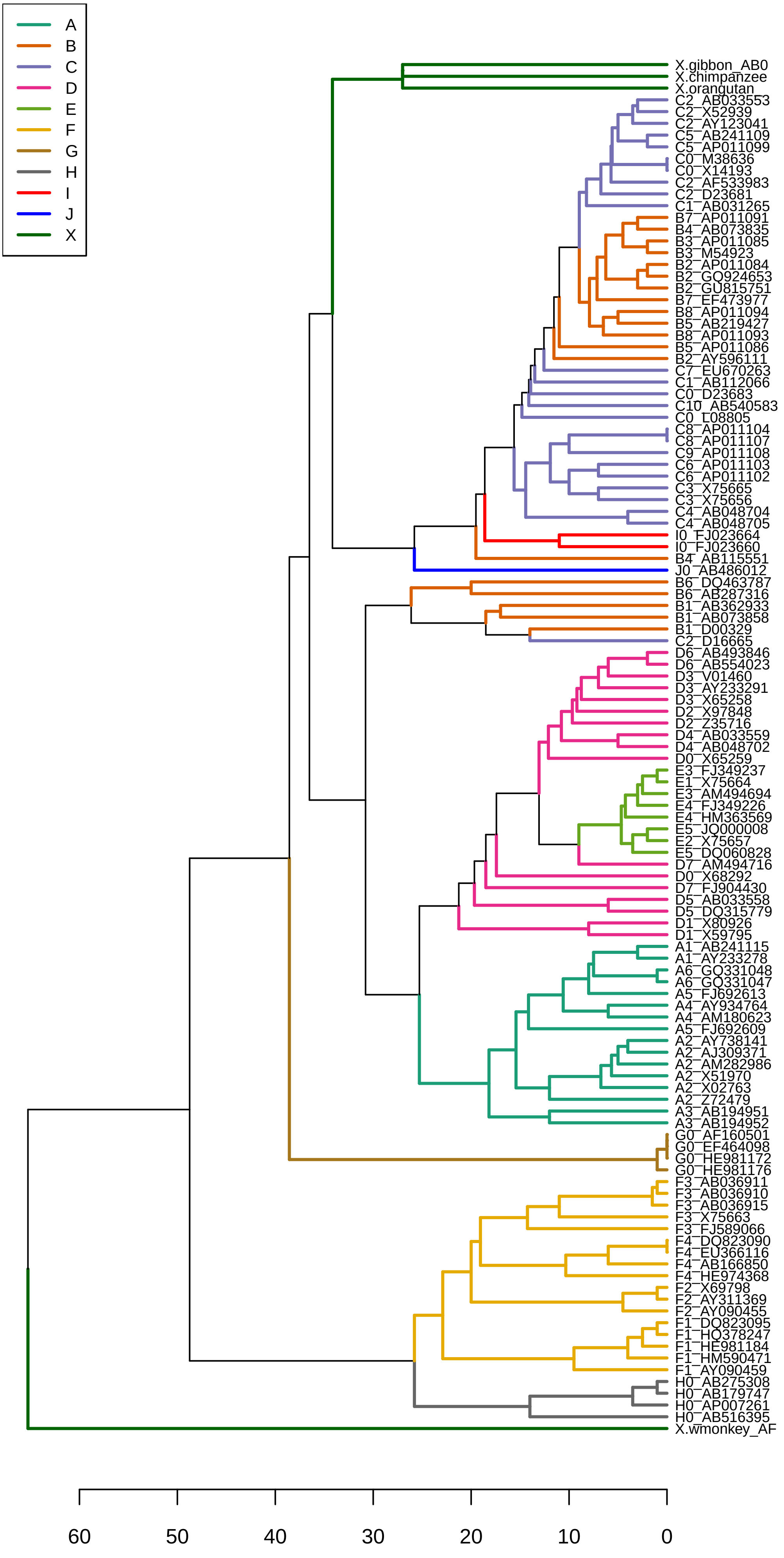

UPGMA tree (N): 1881−2280

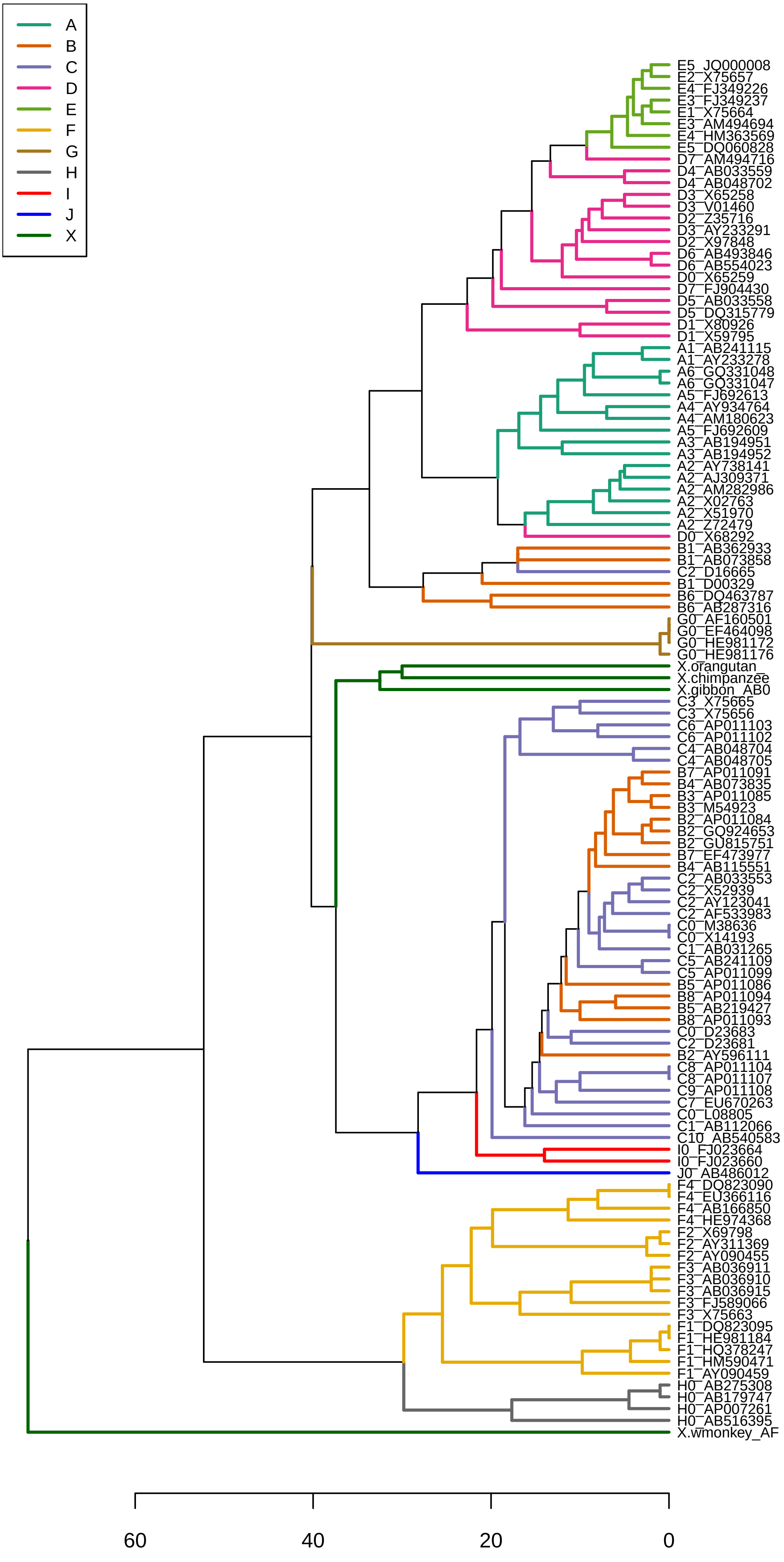

UPGMA tree (N): 1921−2320

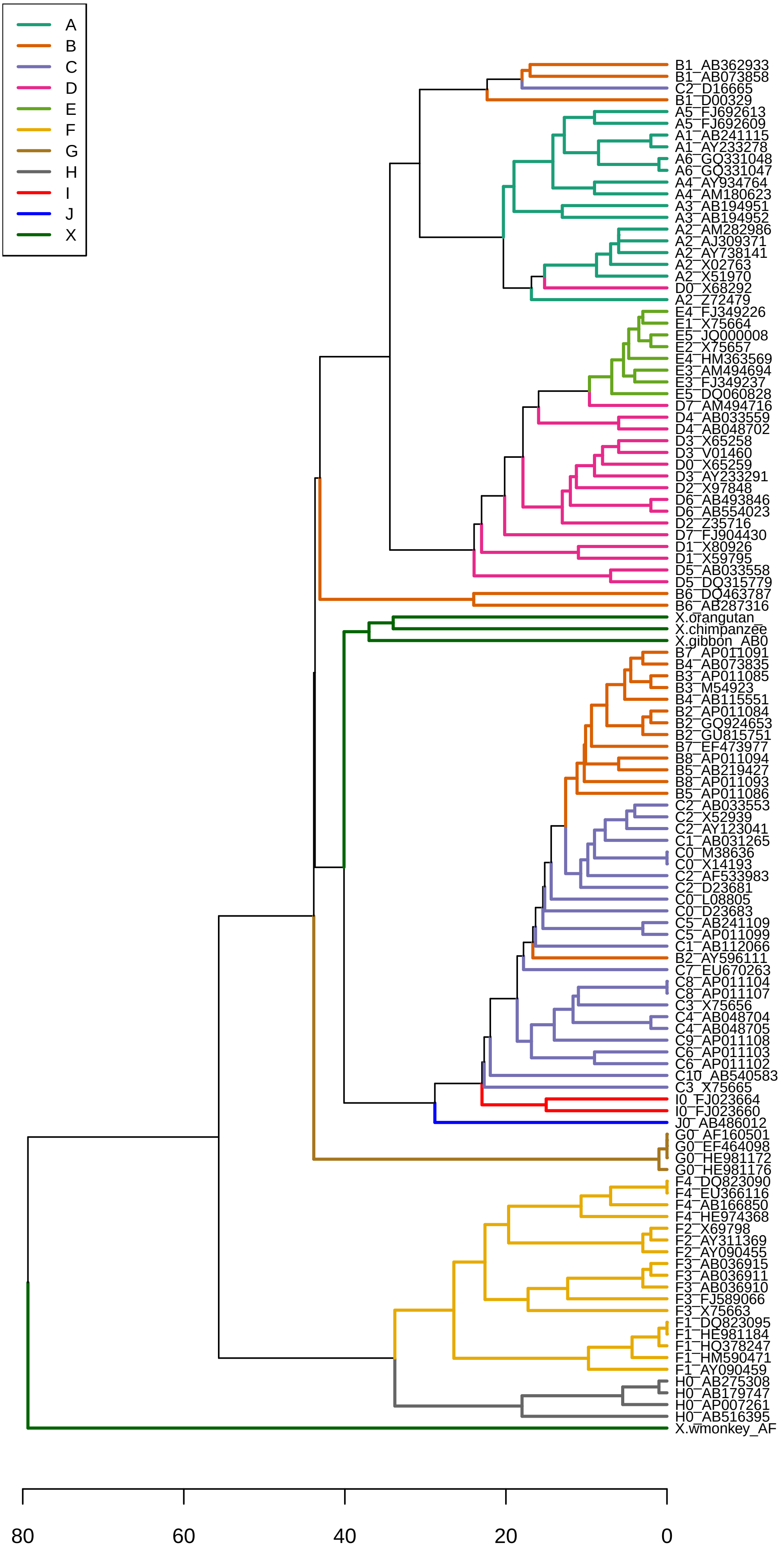

UPGMA tree (N): 1961−2360

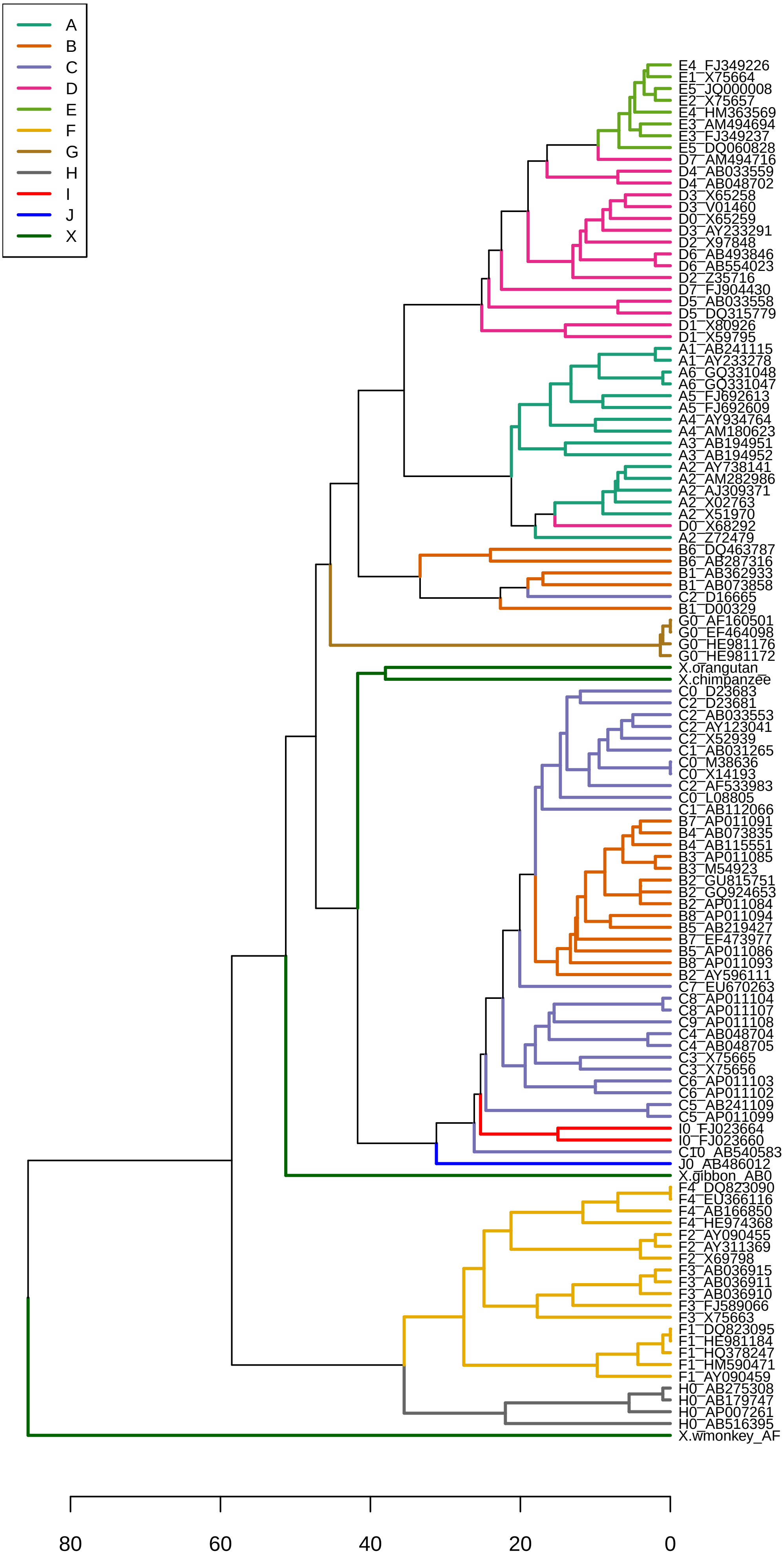

UPGMA tree (N): 2001−2400

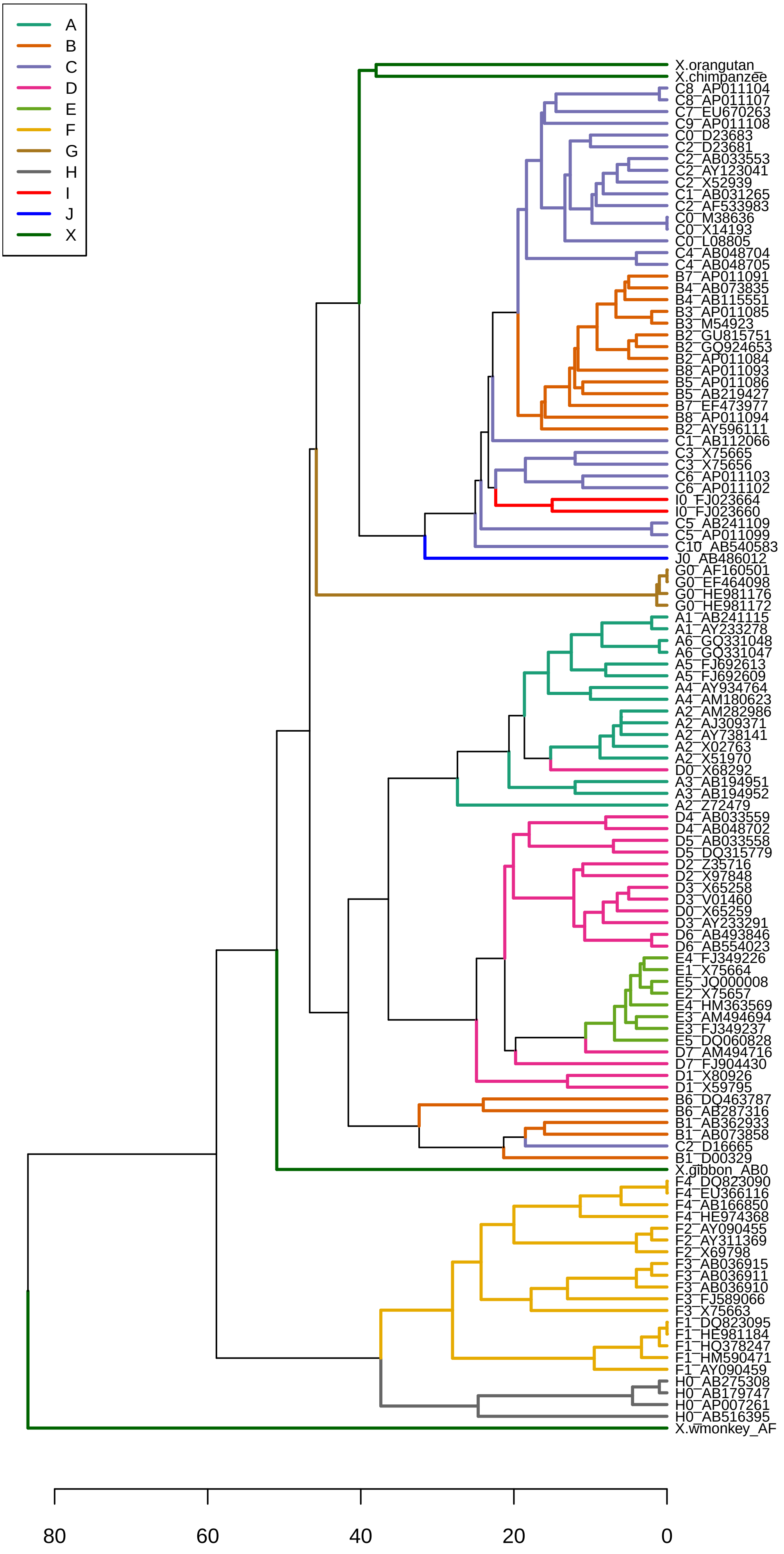

UPGMA tree (N): 2041−2440

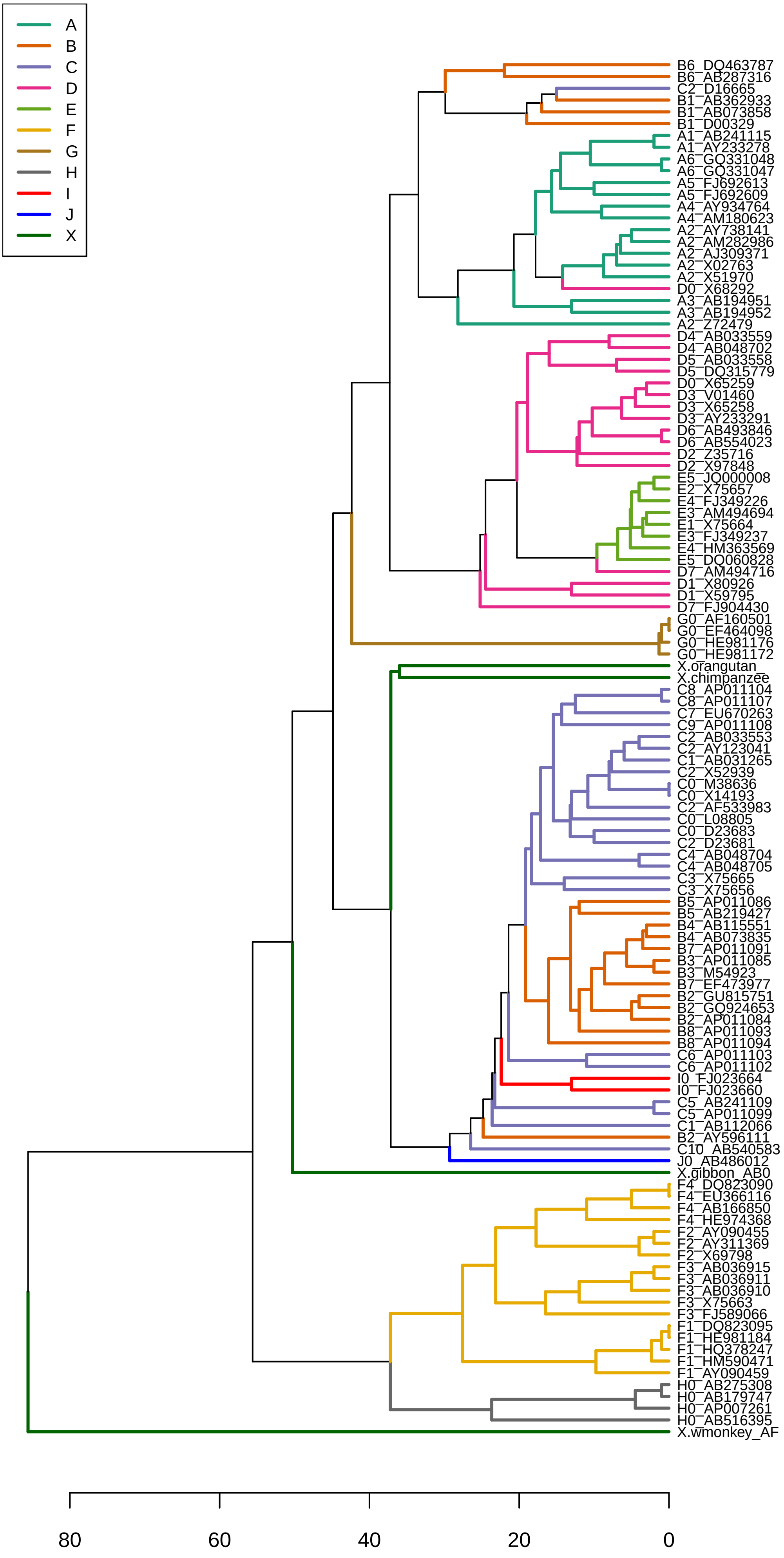

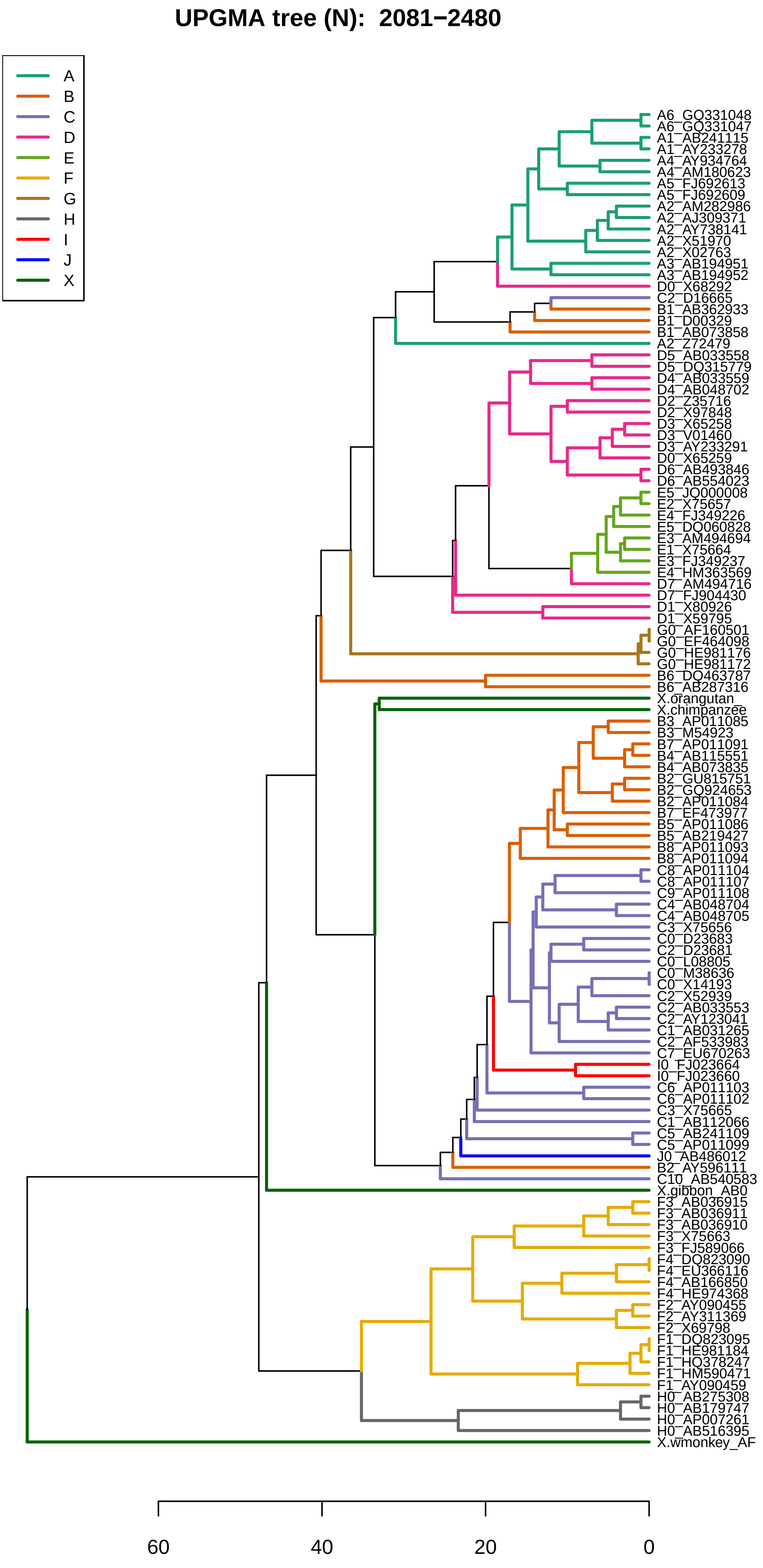
UPGMA tree (N): 2081−2480
A
B
C
D
E
F
G
H
I
J
X
A6_GQ331048
A6_GQ331047
A1_AB241115
A1_AY233278
A4_AY934764
A4_AM180623
A5_FJ692613
A5_FJ692609
A2_AM282986
A2_AJ309371
A2_AY738141
A2_X51970
A2_X02763
A3_AB194951
A3_AB194952
D0_X68292
C2_D16665
B1_AB362933
B1_D00329
B1_AB073858
A2_Z72479
D5_AB033558
D5_DQ315779
D4_AB033559
D4_AB048702
D2_Z35716
D2_X97848
D3_X65258
D3_V01460
D3_AY233291
D0_X65259
D6_AB493846
D6_AB554023
E5_JQ000008
E2_X75657
E4_FJ349226
E5_DQ060828
E3_AM494694
E1_X75664
E3_FJ349237
E4_HM363569
D7_AM494716
D7_FJ904430
D1_X80926
D1_X59795
G0_AF160501
G0_EF464098
G0_HE981176
G0_HE981172
B6_DQ463787
B6_AB287316
X.orangutan_
X.chimpanzee
B3_AP011085
B3_M54923
B7_AP011091
B4_AB115551
B4_AB073835
B2_GU815751
B2_GQ924653
B2_AP011084
B7_EF473977
B5_AP011086
B5_AB219427
B8_AP011093
B8_AP011094
C8_AP011104
C8_AP011107
C9_AP011108
C4_AB048704
C4_AB048705
C3_X75656
C0_D23683
C2_D23681
C0_L08805
C0_M38636
C0_X14193
C2_X52939
C2_AB033553
C2_AY123041
C1_AB031265
C2_AF533983
C7_EU670263
I0_FJ023664
I0_FJ023660
C6_AP011103
C6_AP011102
C3_X75665
C1_AB112066
C5_AB241109
C5_AP011099
J0_AB486012
B2_AY596111
C10_AB540583
X.gibbon_AB0
F3_AB036915
F3_AB036911
F3_AB036910
F3_X75663
F3_FJ589066
F4_DQ823090
F4_EU366116
F4_AB166850
F4_HE974368
F2_AY090455
F2_AY311369
F2_X69798
F1_DQ823095
F1_HE981184
F1_HQ378247
F1_HM590471
F1_AY090459
H0_AB275308
H0_AB179747
H0_AP007261
H0_AB516395
X.wmonkey_AF
60
40
20
0

UPGMA tree (N): 2121−2520

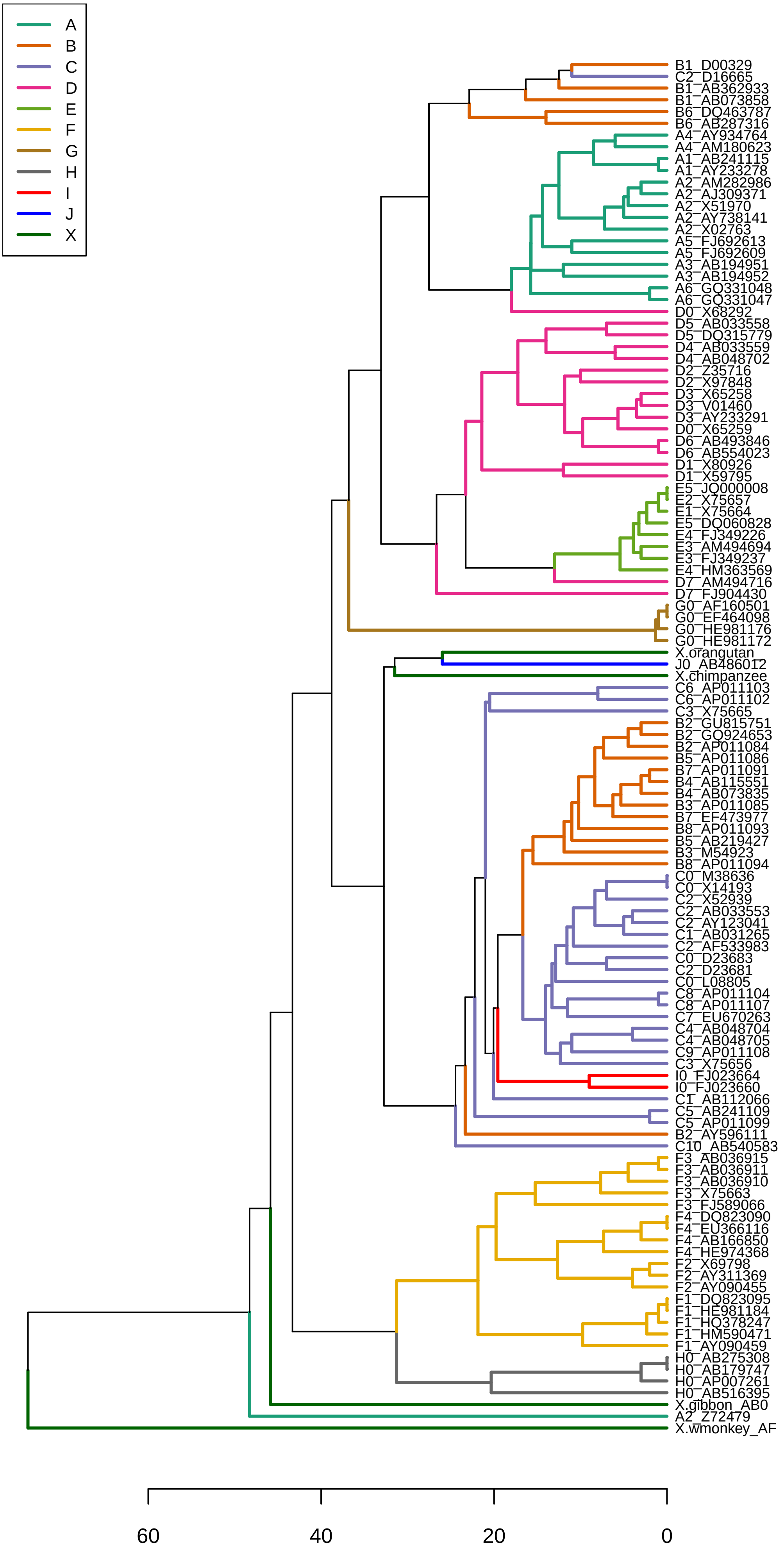

UPGMA tree (N): 2161−2560

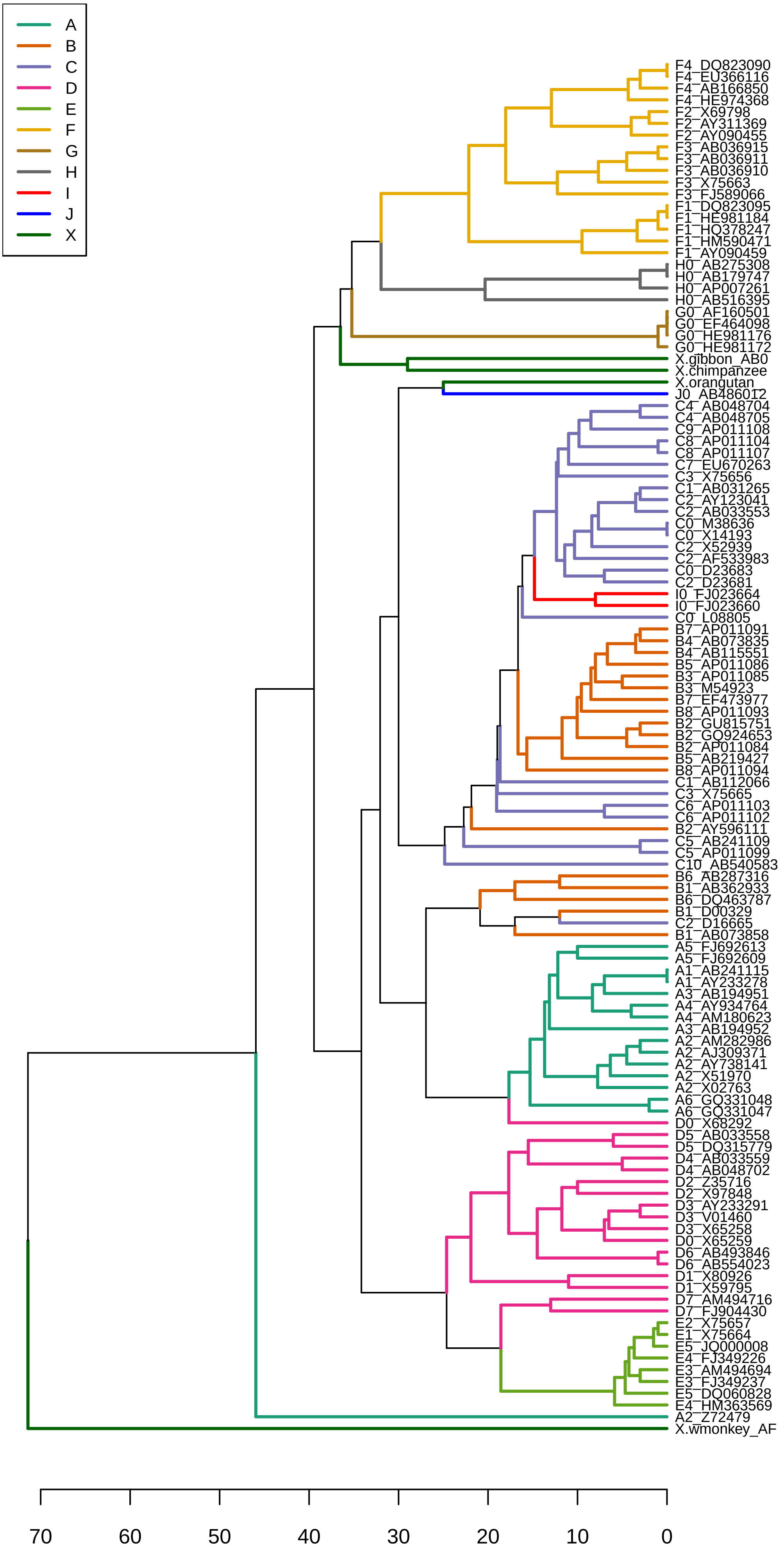

UPGMA tree (N): 2201−2600

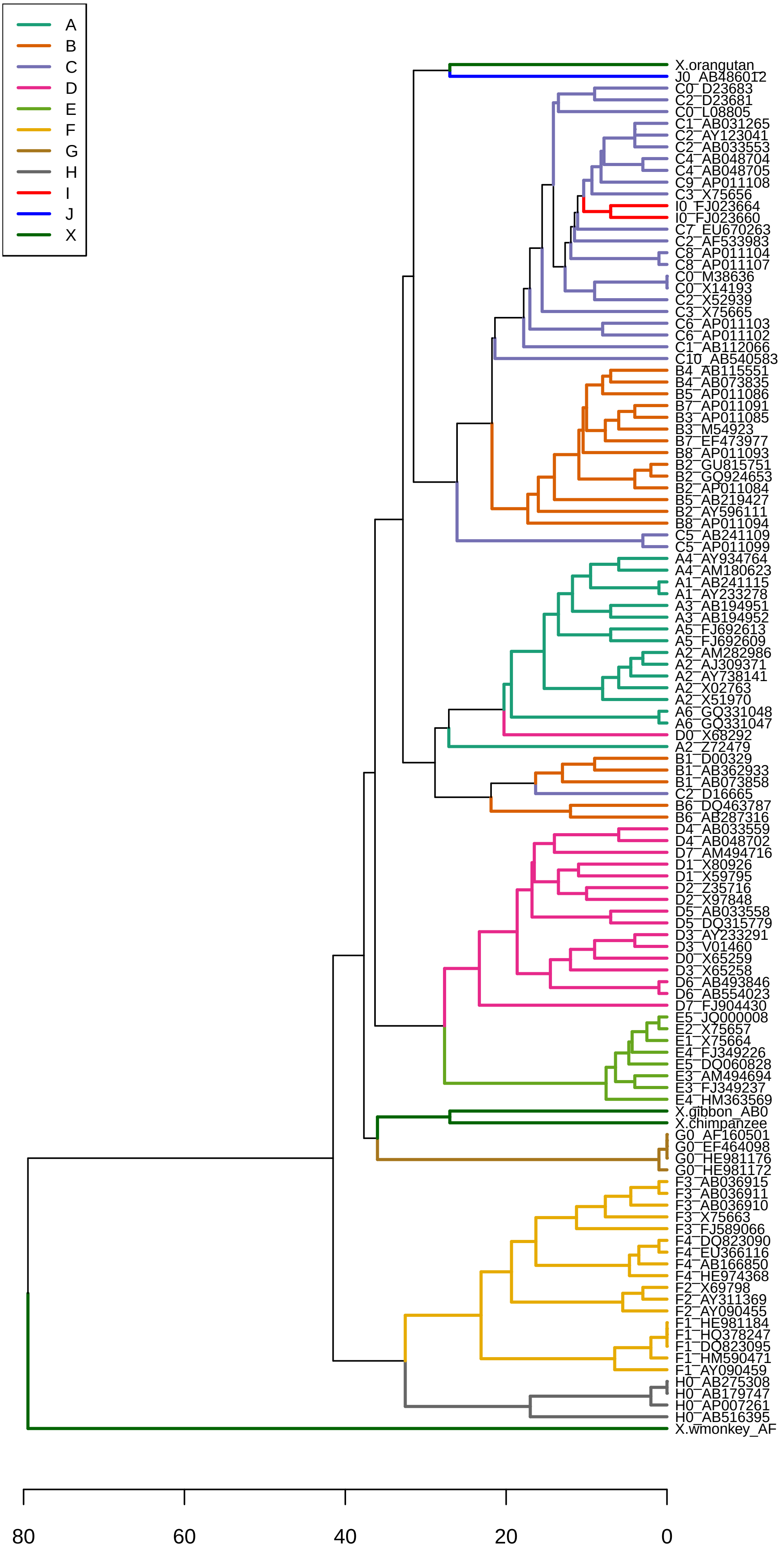

UPGMA tree (N): 2241−2640

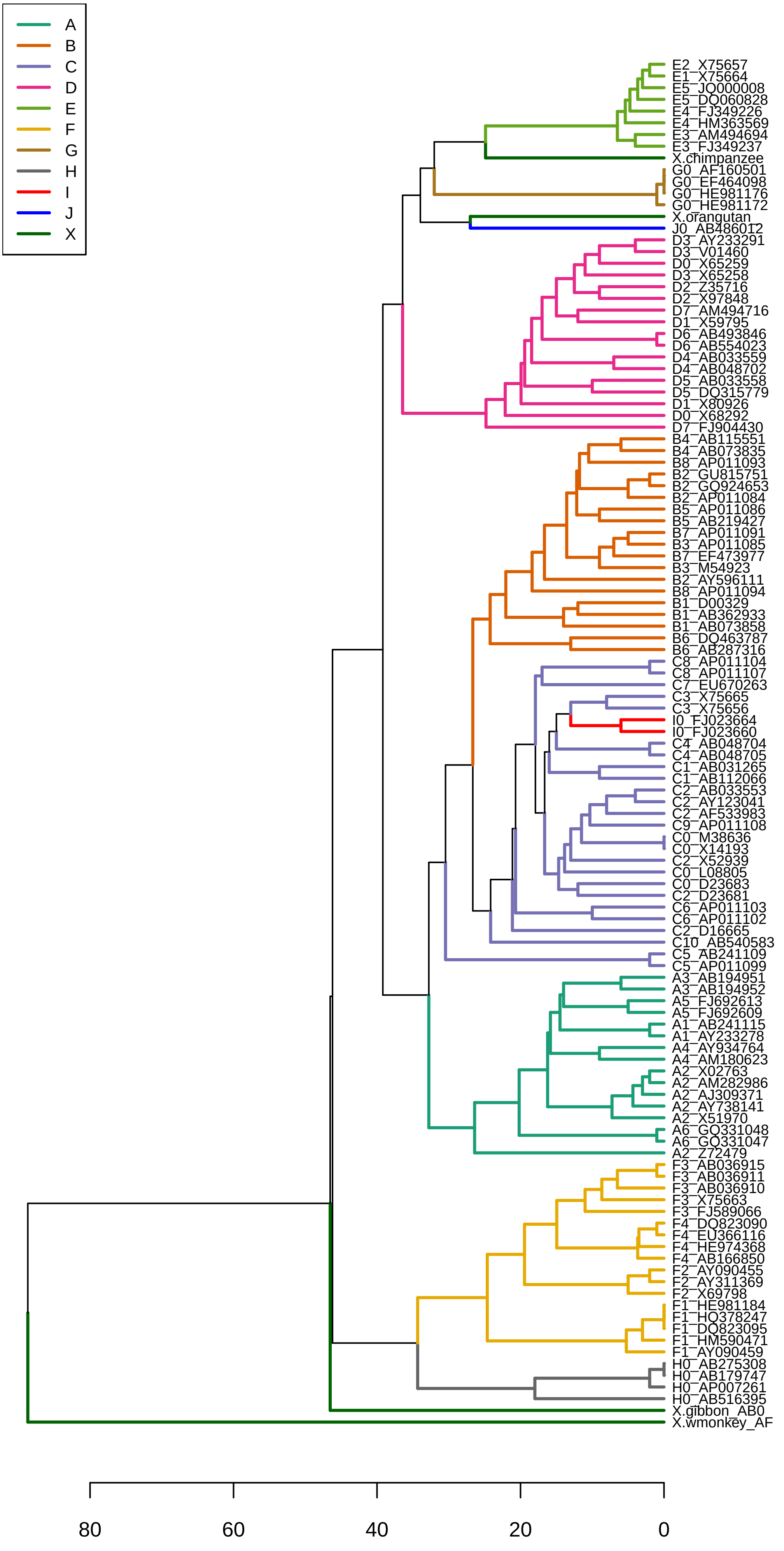

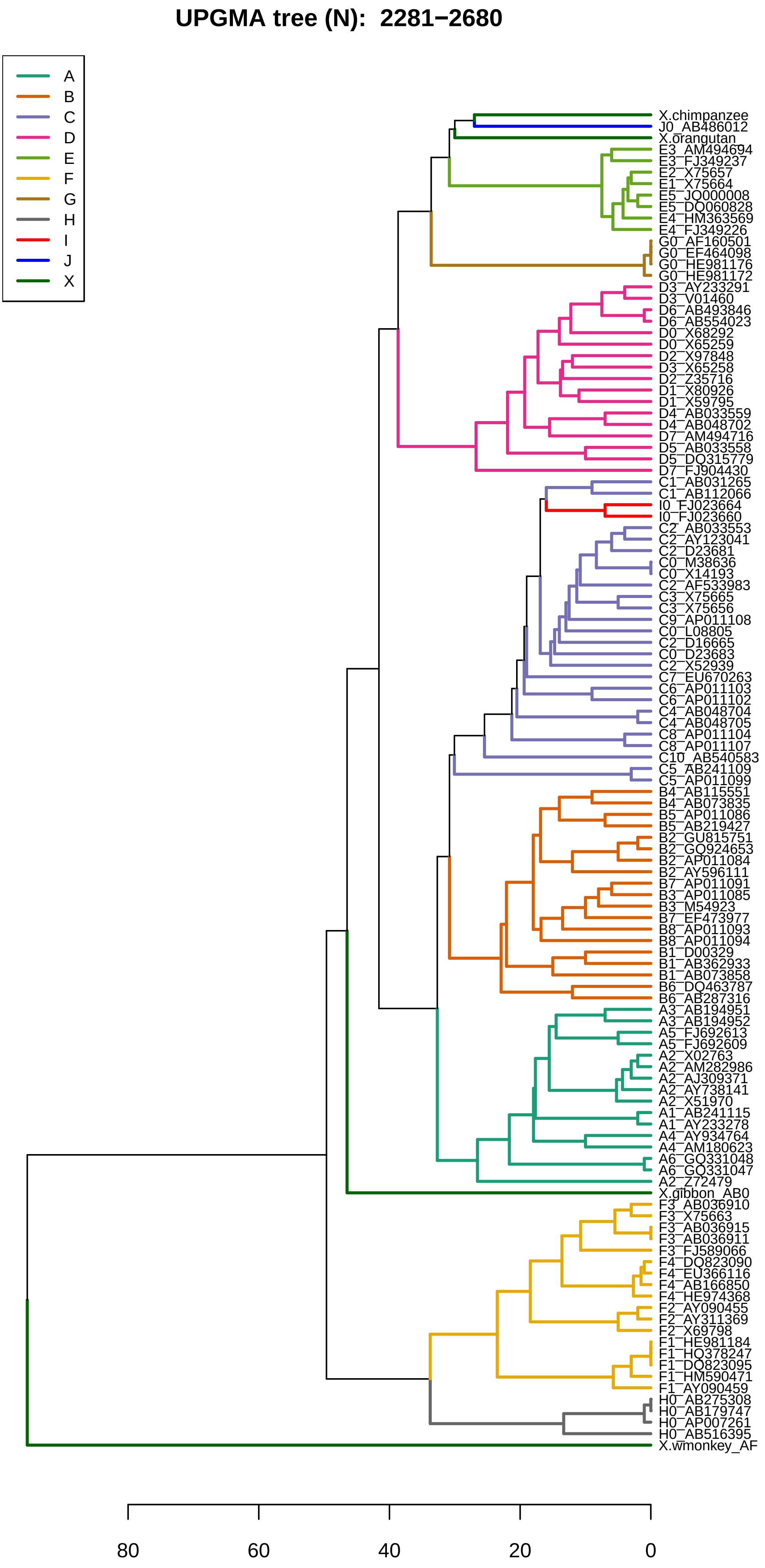
UPGMA tree (N): 2281−2680
A
B
C
D
E
F
G
H
I
J
X
X.chimpanzee
J0_AB486012
X.orangutan
E3_AM494694
E3_FJ349237
E2_X75657
E1_X75664
E5_JQ000008
E5_DQ060828
E4_HM363569
E4_FJ349226
G0_AF160501
G0_EF464098
G0_HE981176
G0_HE981172
D3_AY233291
D3_V01460
D6_AB493846
D6_AB554023
D0_X68292
D0_X65259
D2_X97848
D3_X65258
D2_Z35716
D1_X80926
D1_X59795
D4_AB033559
D4_AB048702
D7_AM494716
D5_AB033558
D5_DQ315779
D7_FJ904430
C1_AB031265
C1_AB112066
I0_FJ023664
I0_FJ023660
C2_AB033553
C2_AY123041
C2_D23681
C0_M38636
C0_X14193
C2_AF533983
C3_X75665
C3_X75656
C9_AP011108
C0_L08805
C2_D16665
C0_D23683
C2_X52939
C7_EU670263
C6_AP011103
C6_AP011102
C4_AB048704
C4_AB048705
C8_AP011104
C8_AP011107
C10_AB540583
C5_AB241109
C5_AP011099
B4_AB115551
B4_AB073835
B5_AP011086
B5_AB219427
B2_GU815751
B2_GQ924653
B2_AP011084
B2_AY596111
B7_AP011091
B3_AP011085
B3_M54923
B7_EF473977
B8_AP011093
B8_AP011094
B1_D00329
B1_AB362933
B1_AB073858
B6_DQ463787
B6_AB287316
A3_AB194951
A3_AB194952
A5_FJ692613
A5_FJ692609
A2_X02763
A2_AM282986
A2_AJ309371
A2_AY738141
A2_X51970
A1_AB241115
A1_AY233278
A4_AY934764
A4_AM180623
A6_GQ331048
A6_GQ331047
A2_Z72479
X.gibbon_AB0
F3_AB036910
F3_X75663
F3_AB036915
F3_AB036911
F3_FJ589066
F4_DQ823090
F4_EU366116
F4_AB166850
F4_HE974368
F2_AY090455
F2_AY311369
F2_X69798
F1_HE981184
F1_HQ378247
F1_DQ823095
F1_HM590471
F1_AY090459
H0_AB275308
H0_AB179747
H0_AP007261
H0_AB516395
X.wmonkey_AF
80
60
40
20
0

UPGMA tree (N): 2321−2720

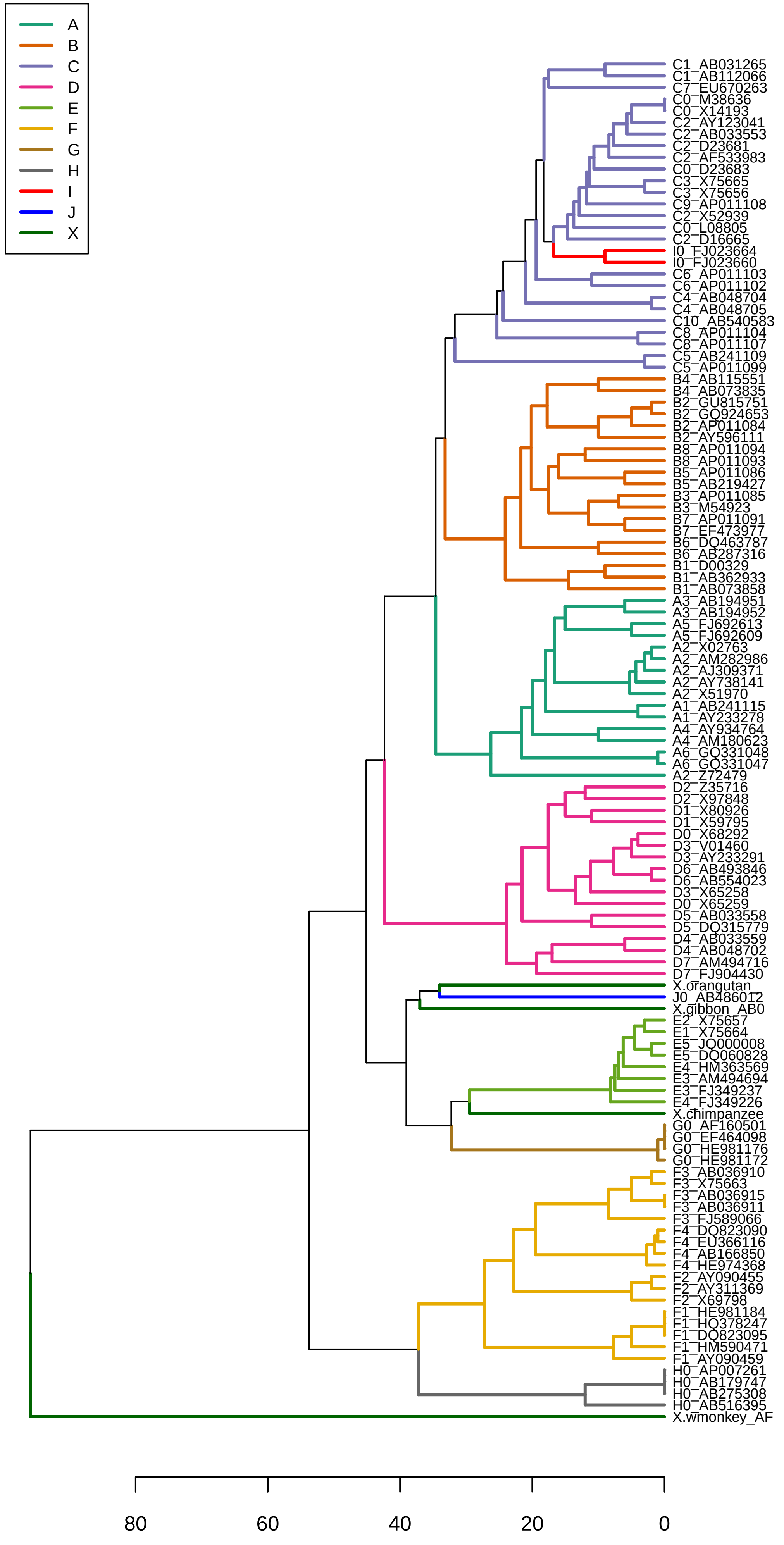

UPGMA tree (N): 2361−2760

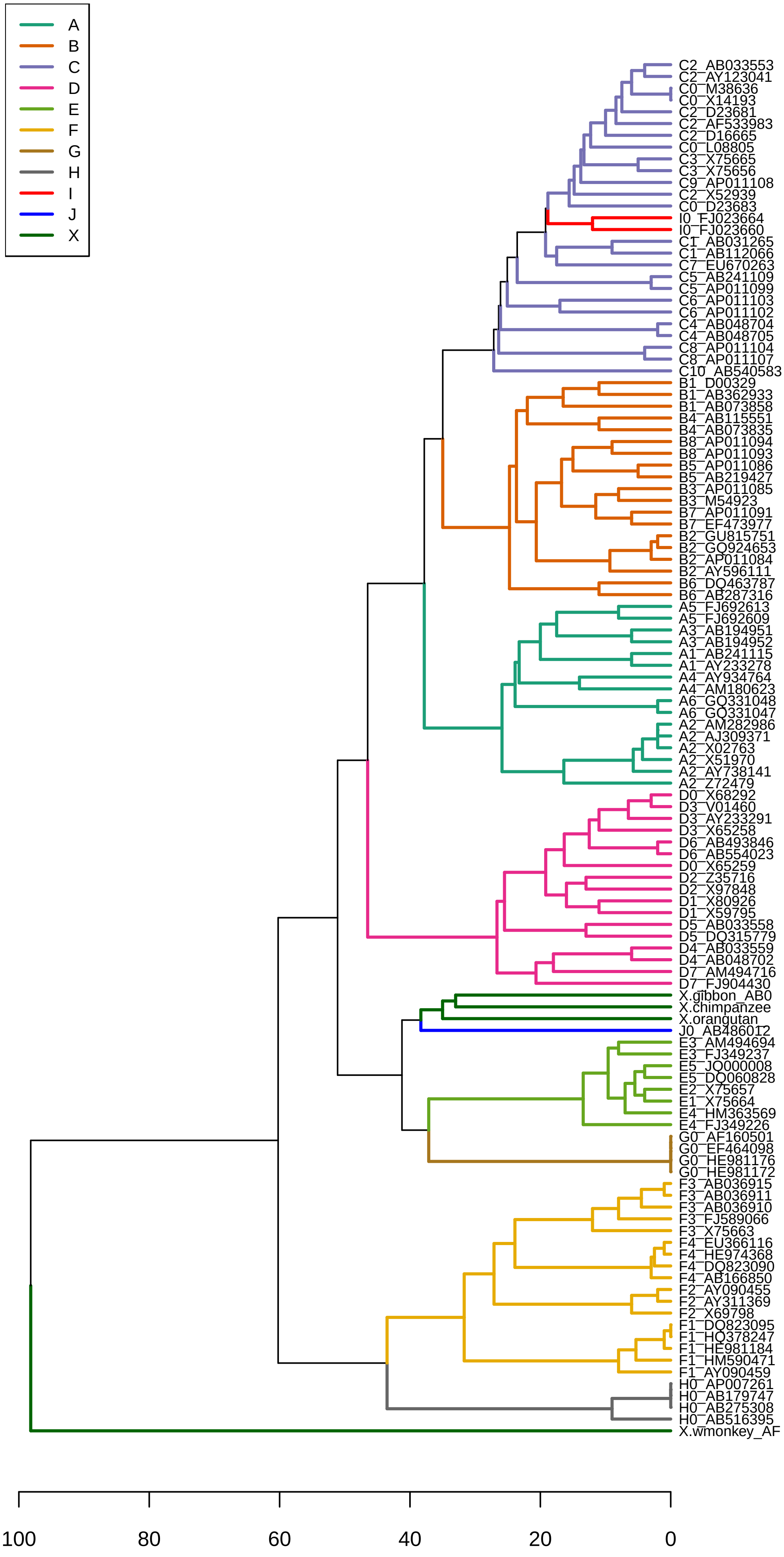

UPGMA tree (N): 2401−2800

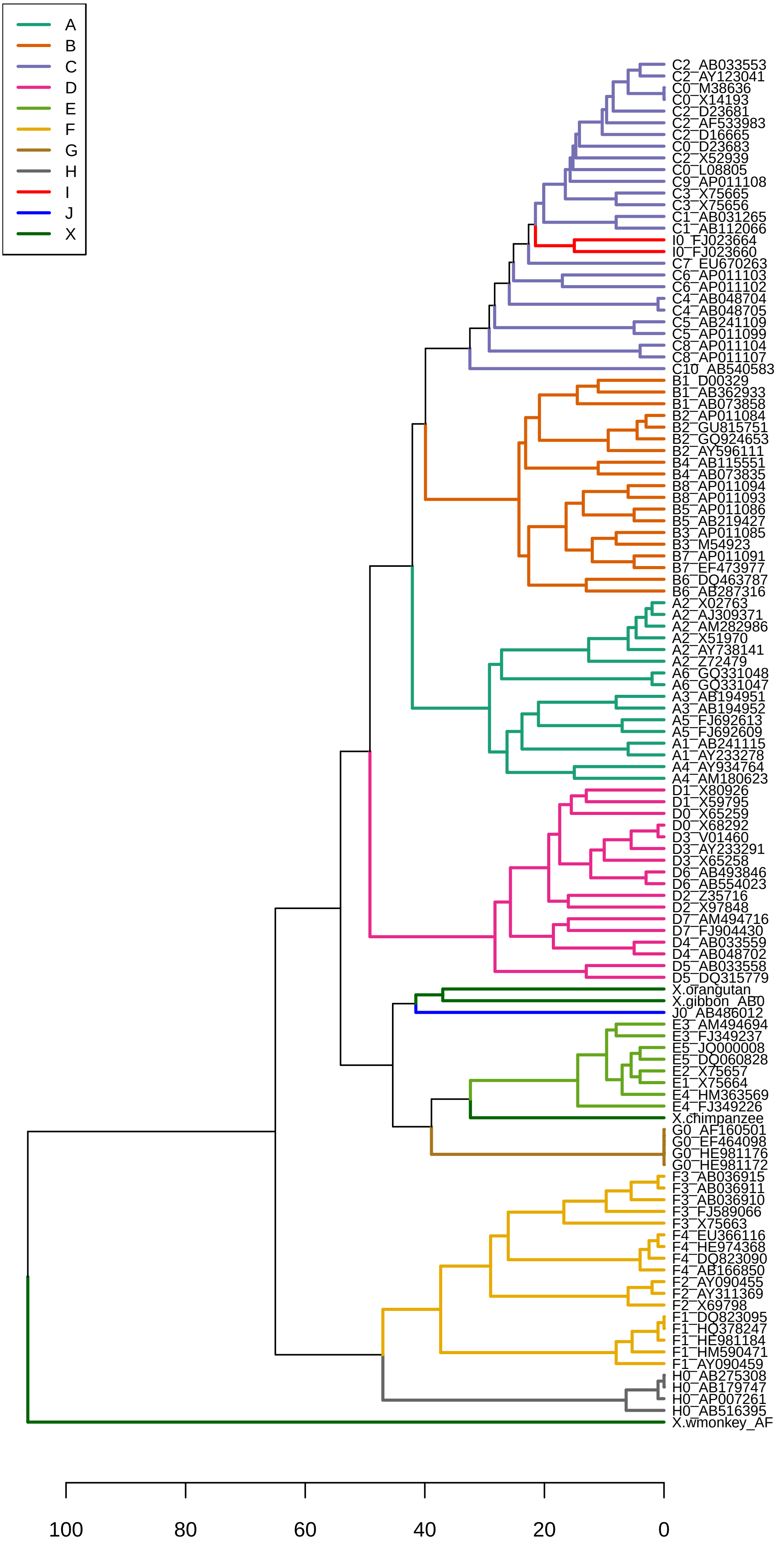

UPGMA tree (N): 2441−2840

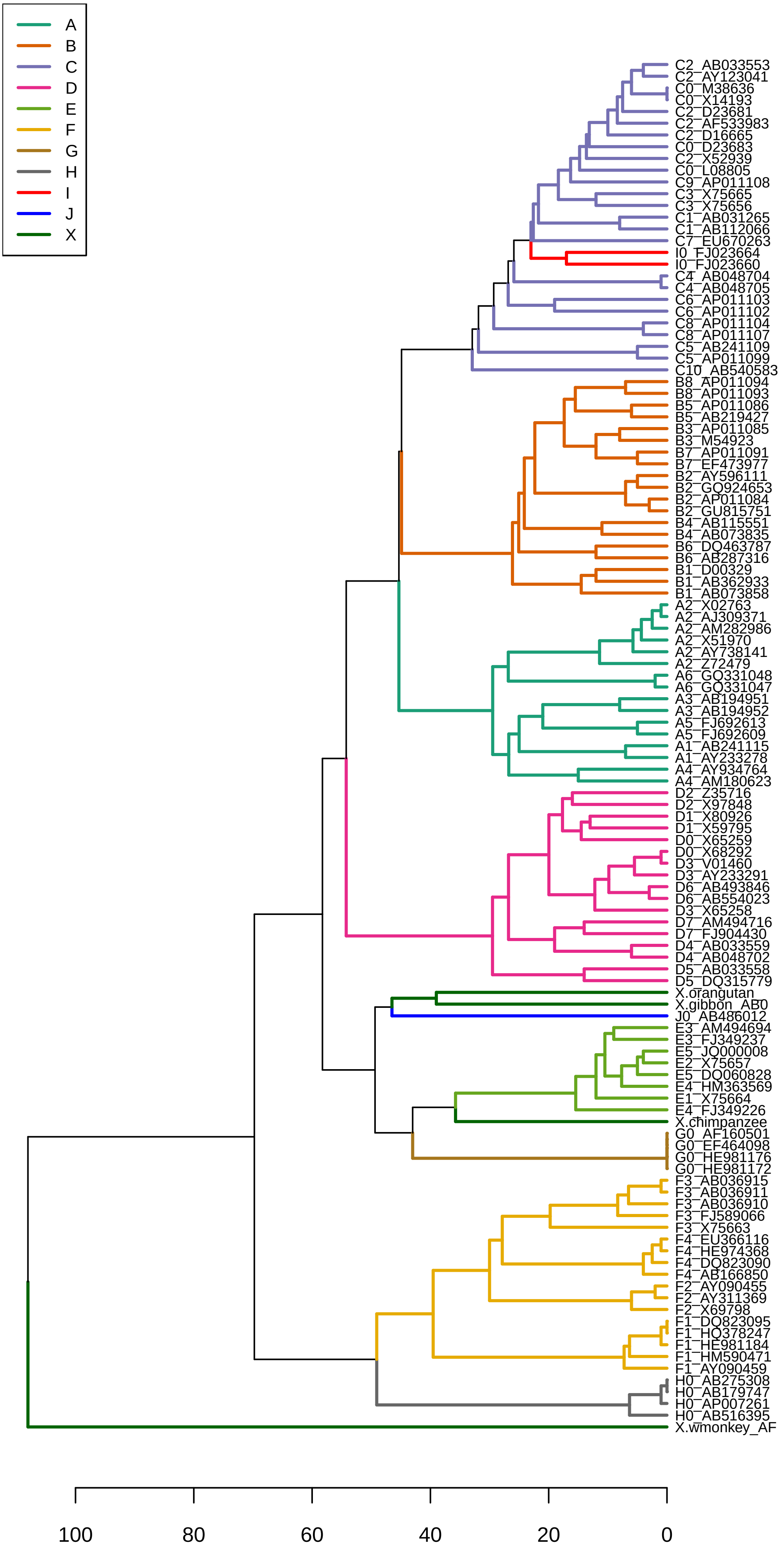

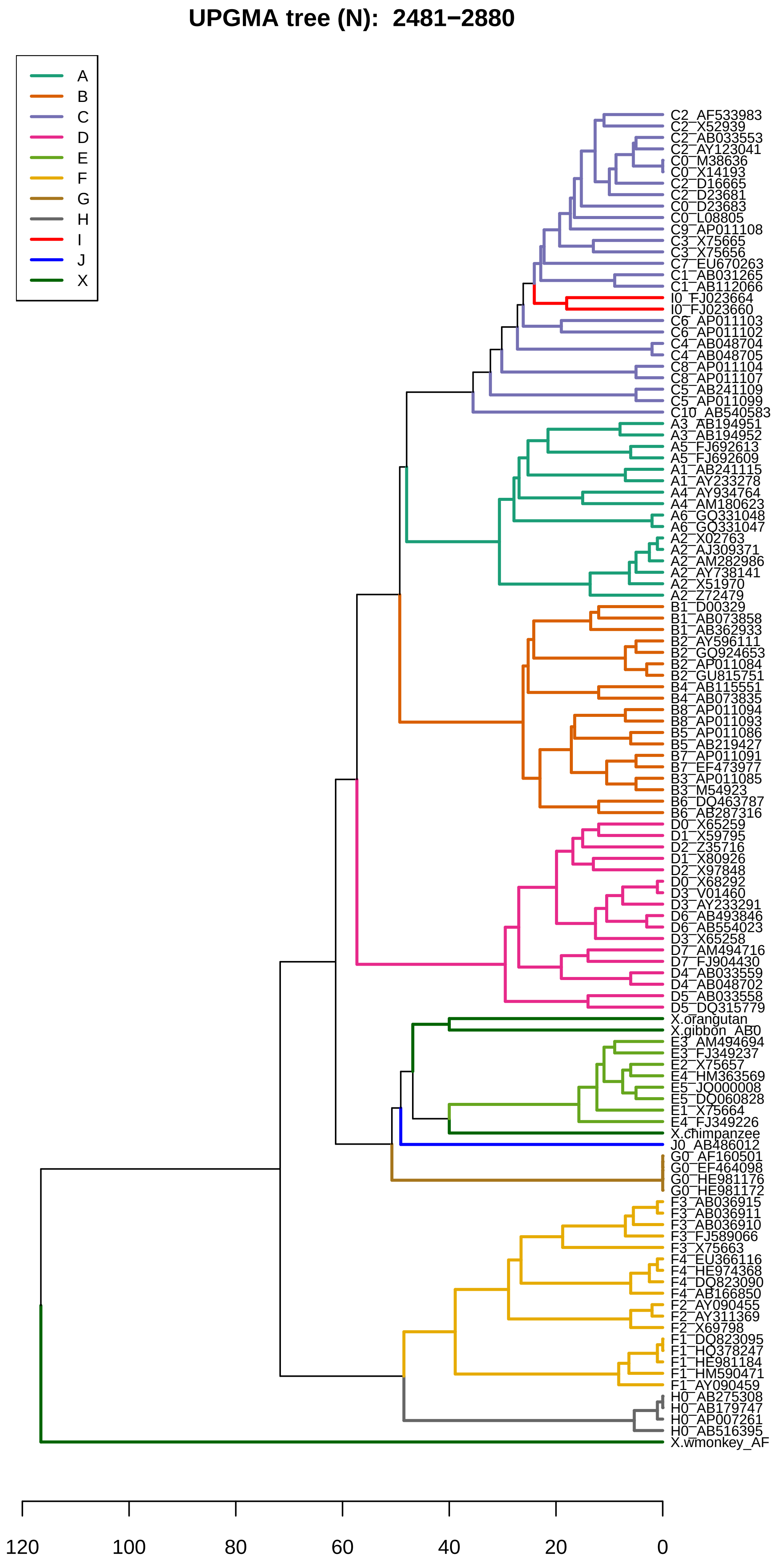
UPGMA tree (N): 2481−2880
A
B
C
D
E
F
G
H
I
J
X
C2_AF533983
C2_X52939
C2_AB033553
C2_AY123041
C0_M38636
C0_X14193
C2_D16665
C2_D23681
C0_D23683
C0_L08805
C9_AP011108
C3_X75665
C3_X75656
C7_EU670263
C1_AB031265
C1_AB112066
I0_FJ023664
I0_FJ023660
C6_AP011103
C6_AP011102
C4_AB048704
C4_AB048705
C8_AP011104
C8_AP011107
C5_AB241109
C5_AP011099
C10_AB540583
A3_AB194951
A3_AB194952
A5_FJ692613
A5_FJ692609
A1_AB241115
A1_AY233278
A4_AY934764
A4_AM180623
A6_GQ331048
A6_GQ331047
A2_X02763
A2_AJ309371
A2_AM282986
A2_AY738141
A2_X51970
A2_Z72479
B1_D00329
B1_AB073858
B1_AB362933
B2_AY596111
B2_GQ924653
B2_AP011084
B2_GU815751
B4_AB115551
B4_AB073835
B8_AP011094
B8_AP011093
B5_AP011086
B5_AB219427
B7_AP011091
B7_EF473977
B3_AP011085
B3_M54923
B6_DQ463787
B6_AB287316
D0_X65259
D1_X59795
D2_Z35716
D1_X80926
D2_X97848
D0_X68292
D3_V01460
D3_AY233291
D6_AB493846
D6_AB554023
D3_X65258
D7_AM494716
D7_FJ904430
D4_AB033559
D4_AB048702
D5_AB033558
D5_DQ315779
X.orangutan
X.gibbon_AB0
E3_AM494694
E3_FJ349237
E2_X75657
E4_HM363569
E5_JQ000008
E5_DQ060828
E1_X75664
E4_FJ349226
X.chimpanzee
J0_AB486012
G0_AF160501
G0_EF464098
G0_HE981176
G0_HE981172
F3_AB036915
F3_AB036911
F3_AB036910
F3_FJ589066
F3_X75663
F4_EU366116
F4_HE974368
F4_DQ823090
F4_AB166850
F2_AY090455
F2_AY311369
F2_X69798
F1_DQ823095
F1_HQ378247
F1_HE981184
F1_HM590471
F1_AY090459
H0_AB275308
H0_AB179747
H0_AP007261
H0_AB516395
X.wmonkey_AF
120
100
80
60
40
20
0

UPGMA tree (N): 2521−2920

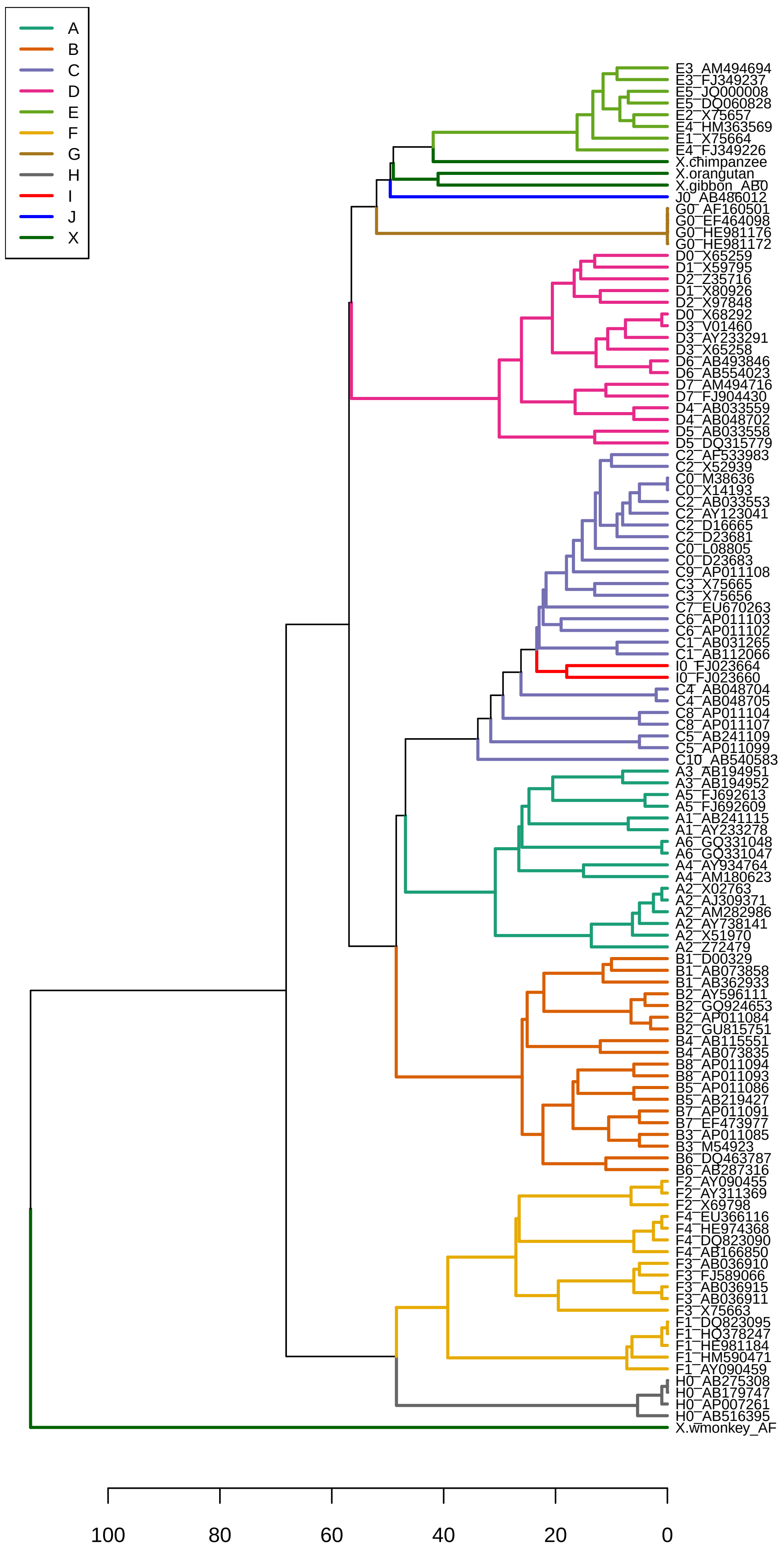

UPGMA tree (N): 2561−2960

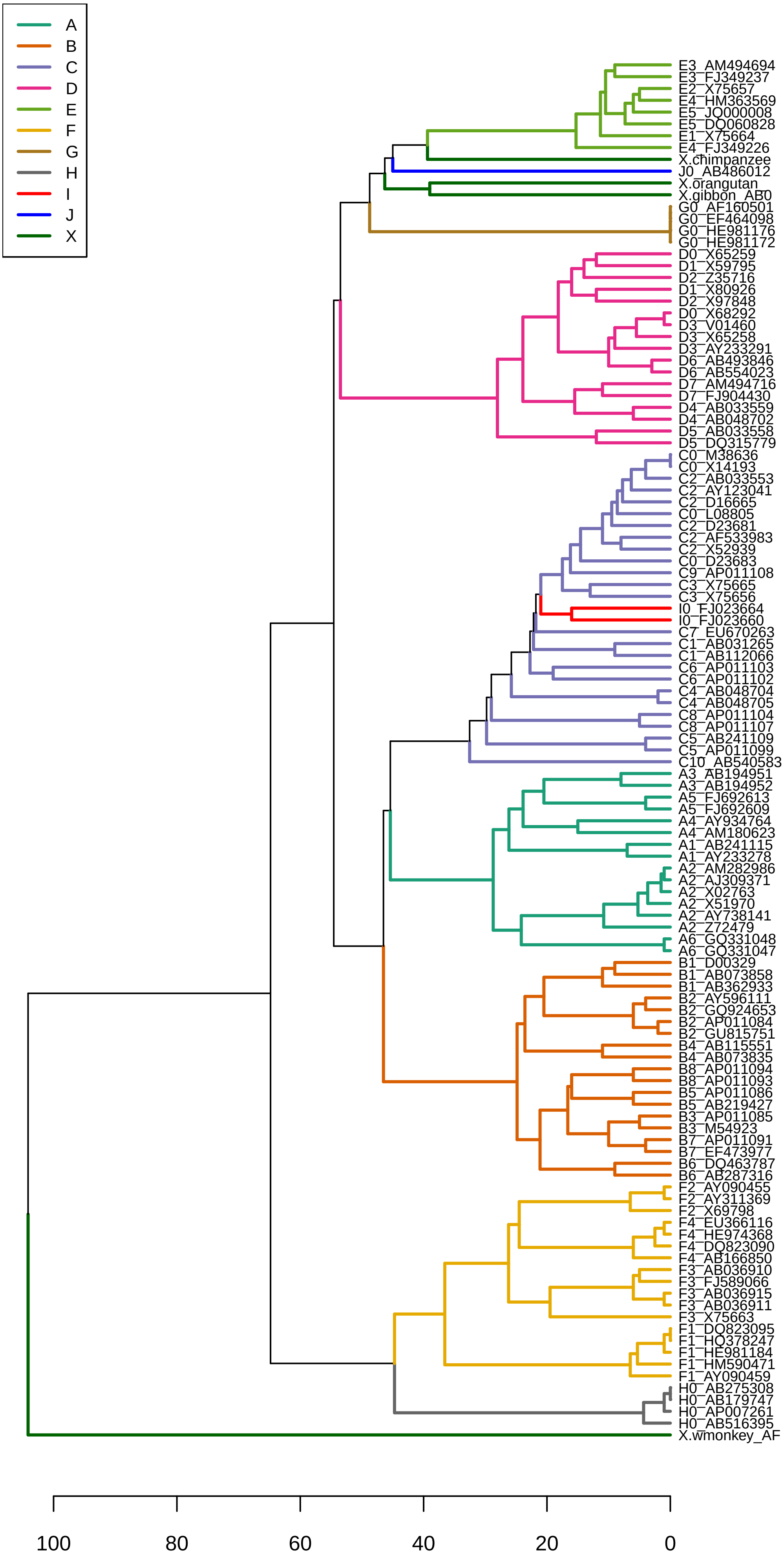

UPGMA tree (N): 2601−3000

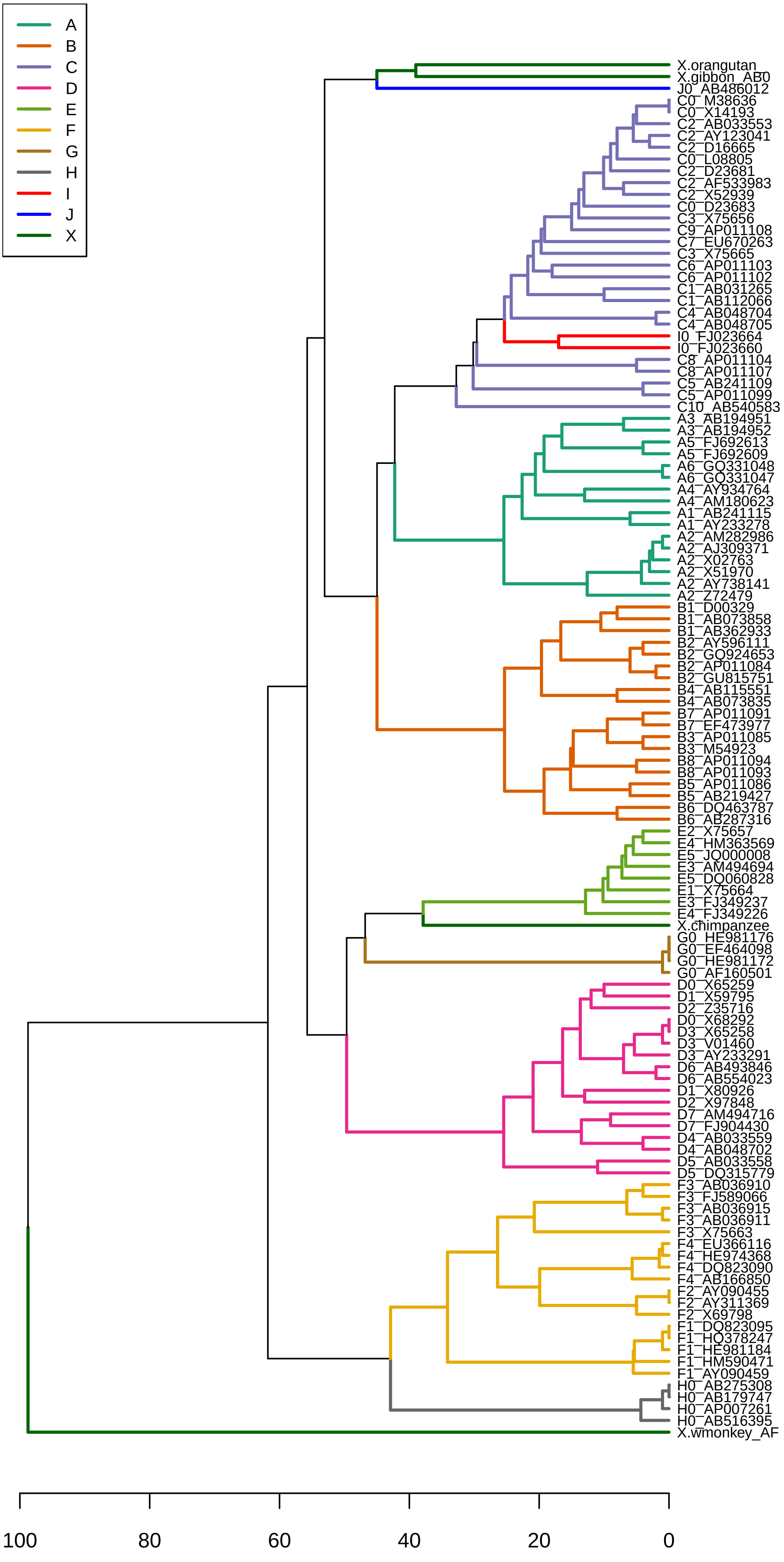

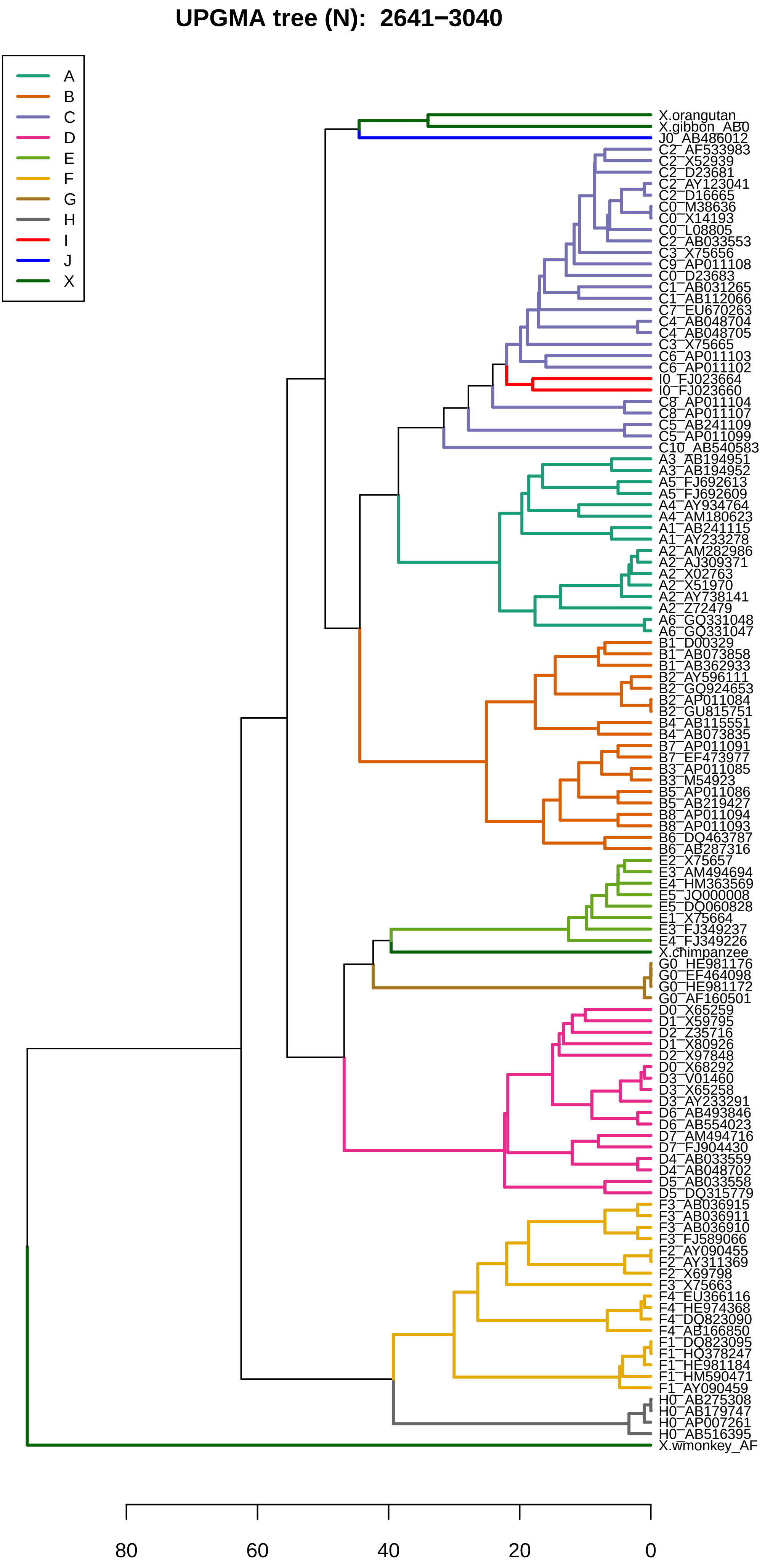
UPGMA tree (N): 2641−3040
A
B
C
D
E
F
G
H
I
J
X
X.orangutan
X.gibbon_AB0
J0_AB486012
C2_AF533983
C2_X52939
C2_D23681
C2_AY123041
C2_D16665
C0_M38636
C0_X14193
C0_L08805
C2_AB033553
C3_X75656
C9_AP011108
C0_D23683
C1_AB031265
C1_AB112066
C7_EU670263
C4_AB048704
C4_AB048705
C3_X75665
C6_AP011103
C6_AP011102
I0_FJ023664
I0_FJ023660
C8_AP011104
C8_AP011107
C5_AB241109
C5_AP011099
C10_AB540583
A3_AB194951
A3_AB194952
A5_FJ692613
A5_FJ692609
A4_AY934764
A4_AM180623
A1_AB241115
A1_AY233278
A2_AM282986
A2_AJ309371
A2_X02763
A2_X51970
A2_AY738141
A2_Z72479
A6_GQ331048
A6_GQ331047
B1_D00329
B1_AB073858
B1_AB362933
B2_AY596111
B2_GQ924653
B2_AP011084
B2_GU815751
B4_AB115551
B4_AB073835
B7_AP011091
B7_EF473977
B3_AP011085
B3_M54923
B5_AP011086
B5_AB219427
B8_AP011094
B8_AP011093
B6_DQ463787
B6_AB287316
E2_X75657
E3_AM494694
E4_HM363569
E5_JQ000008
E5_DQ060828
E1_X75664
E3_FJ349237
E4_FJ349226
X.chimpanzee
G0_HE981176
G0_EF464098
G0_HE981172
G0_AF160501
D0_X65259
D1_X59795
D2_Z35716
D1_X80926
D2_X97848
D0_X68292
D3_V01460
D3_X65258
D3_AY233291
D6_AB493846
D6_AB554023
D7_AM494716
D7_FJ904430
D4_AB033559
D4_AB048702
D5_AB033558
D5_DQ315779
F3_AB036915
F3_AB036911
F3_AB036910
F3_FJ589066
F2_AY090455
F2_AY311369
F2_X69798
F3_X75663
F4_EU366116
F4_HE974368
F4_DQ823090
F4_AB166850
F1_DQ823095
F1_HQ378247
F1_HE981184
F1_HM590471
F1_AY090459
H0_AB275308
H0_AB179747
H0_AP007261
H0_AB516395
X.wmonkey_AF
80
60
40
20
0

UPGMA tree (N): 2681−3080

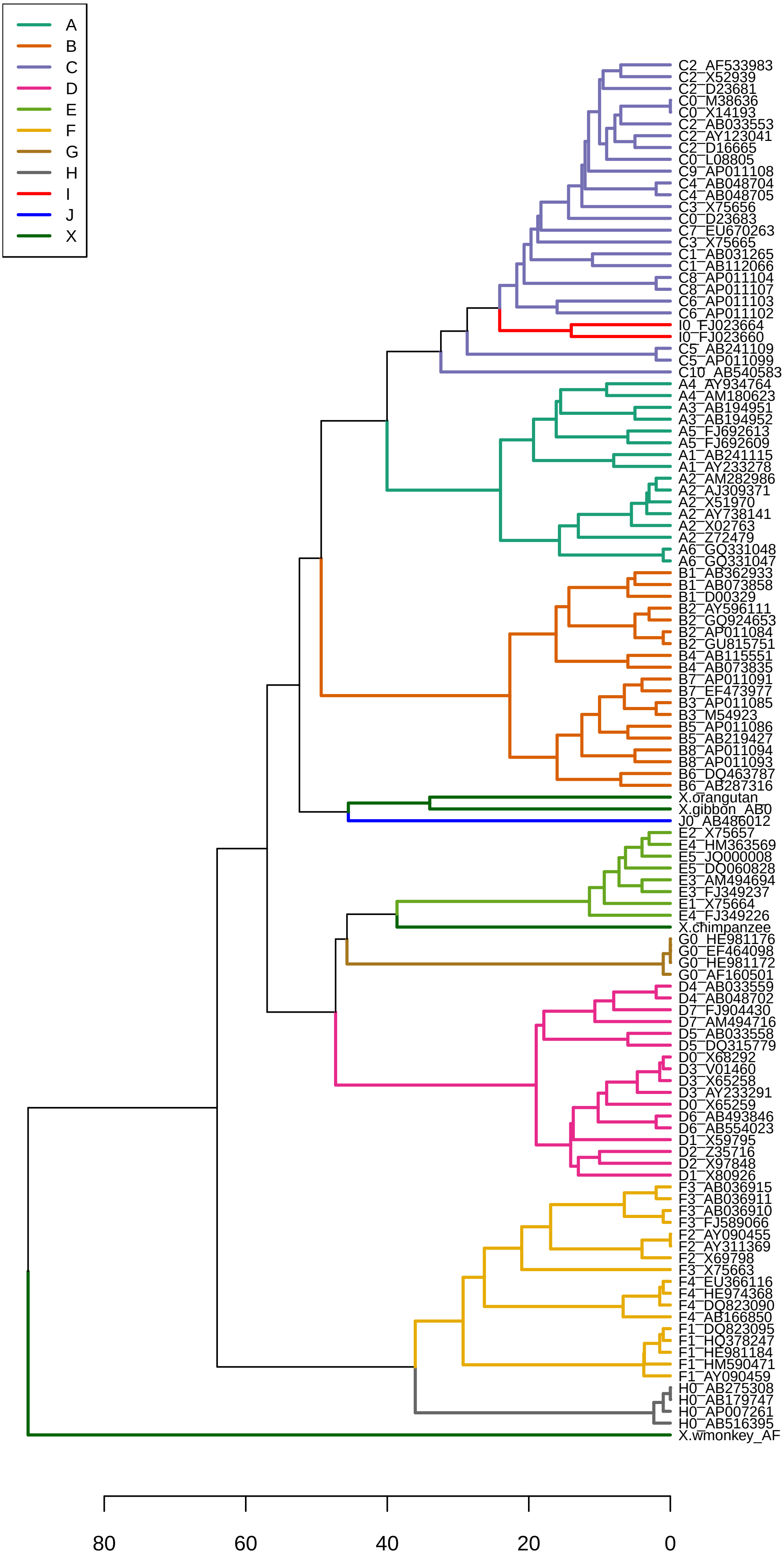

UPGMA tree (N): 2721−3120

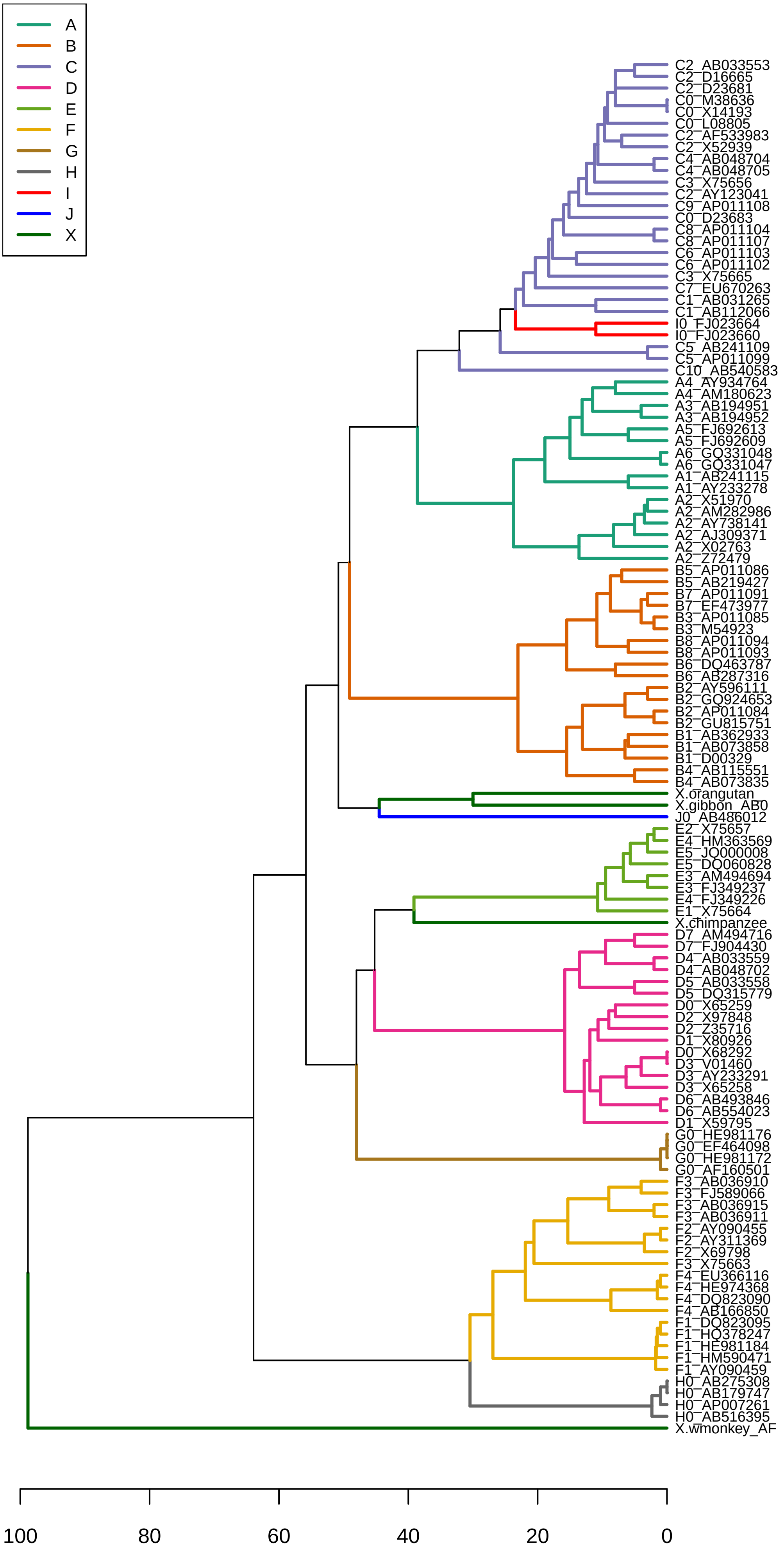

UPGMA tree (N): 2761−3160

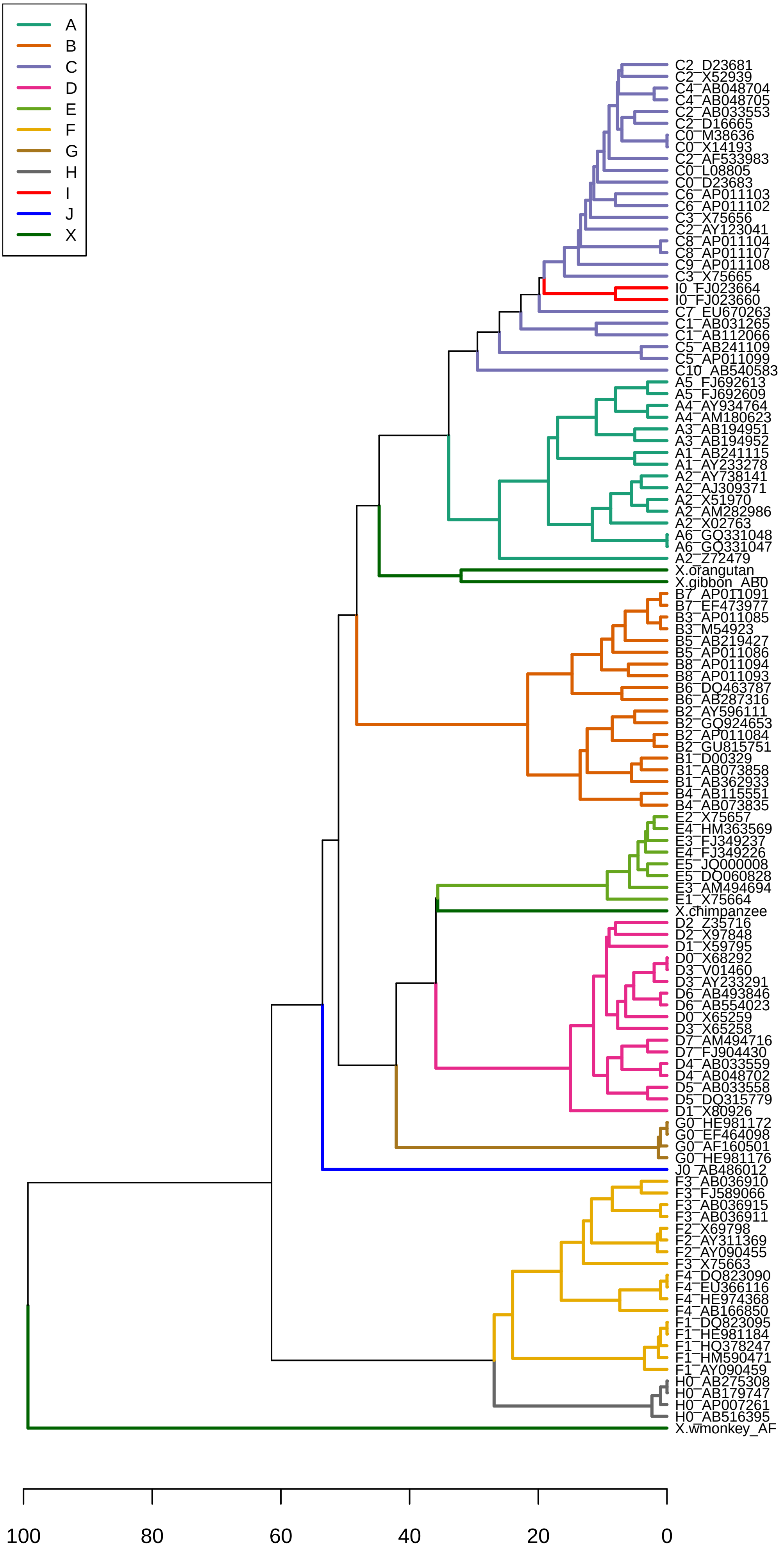

UPGMA tree (N): 2801−3200

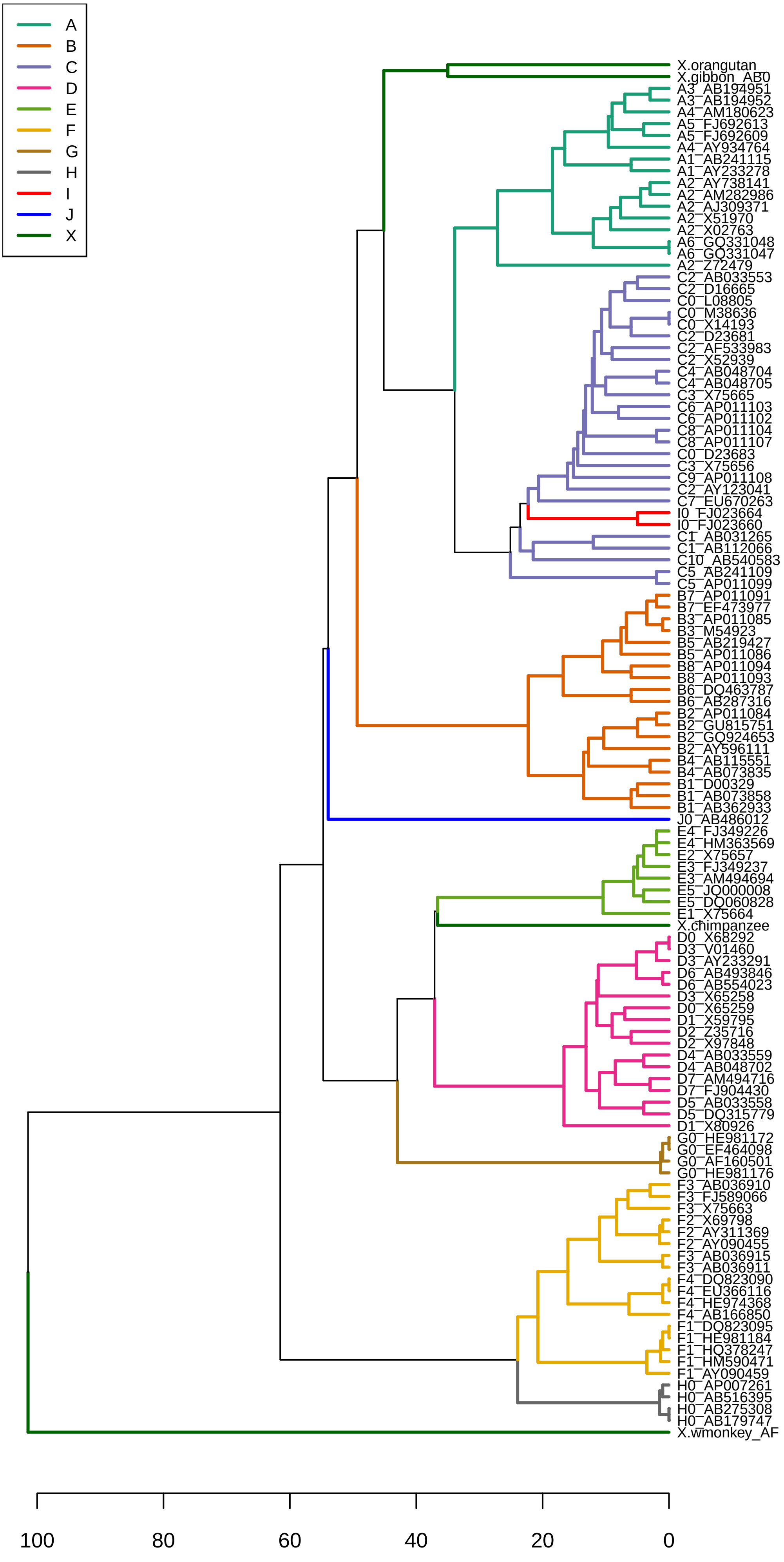

UPGMA tree (N): 2841−3240

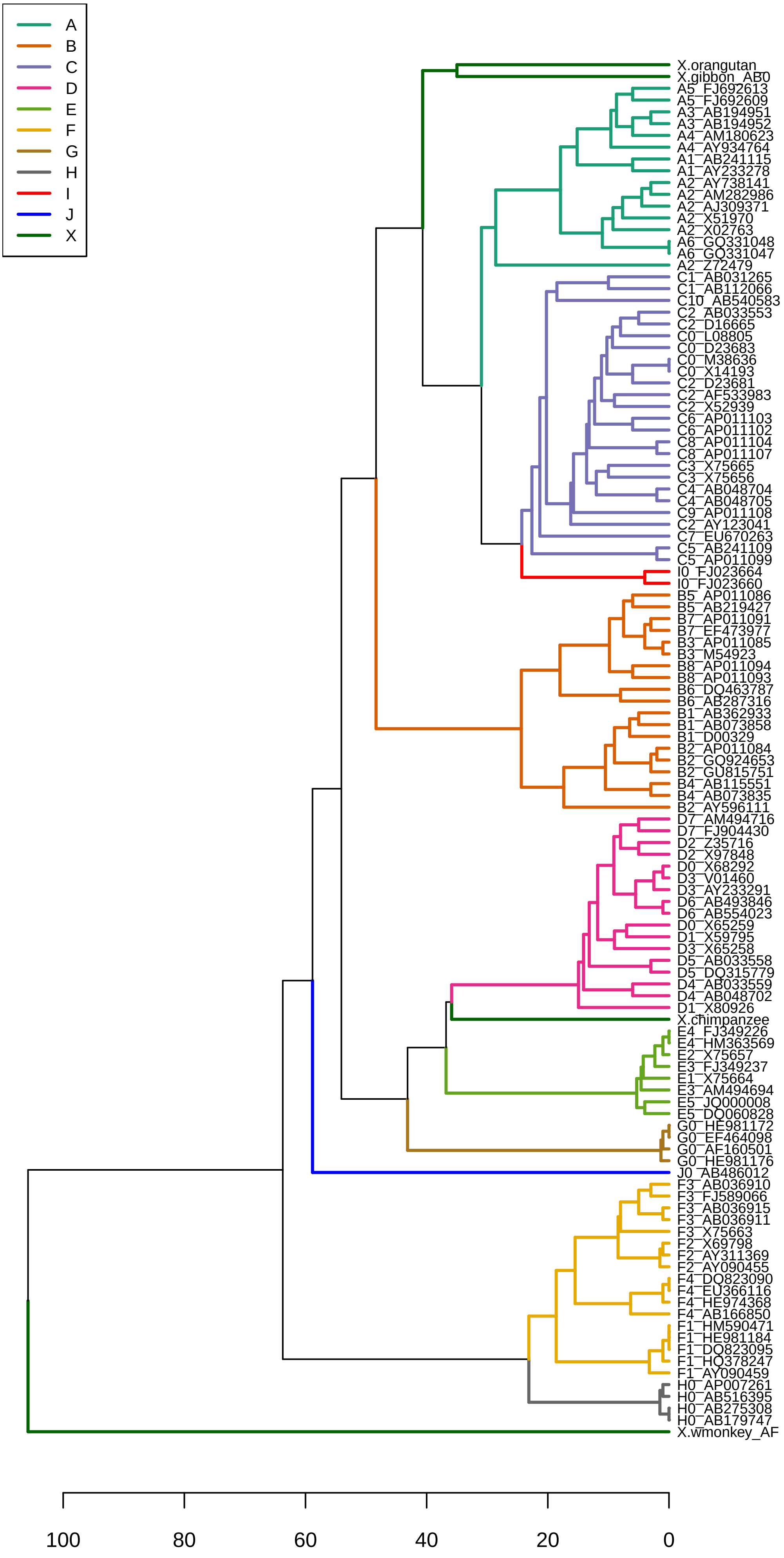

UPGMA tree (N): 2881−3278

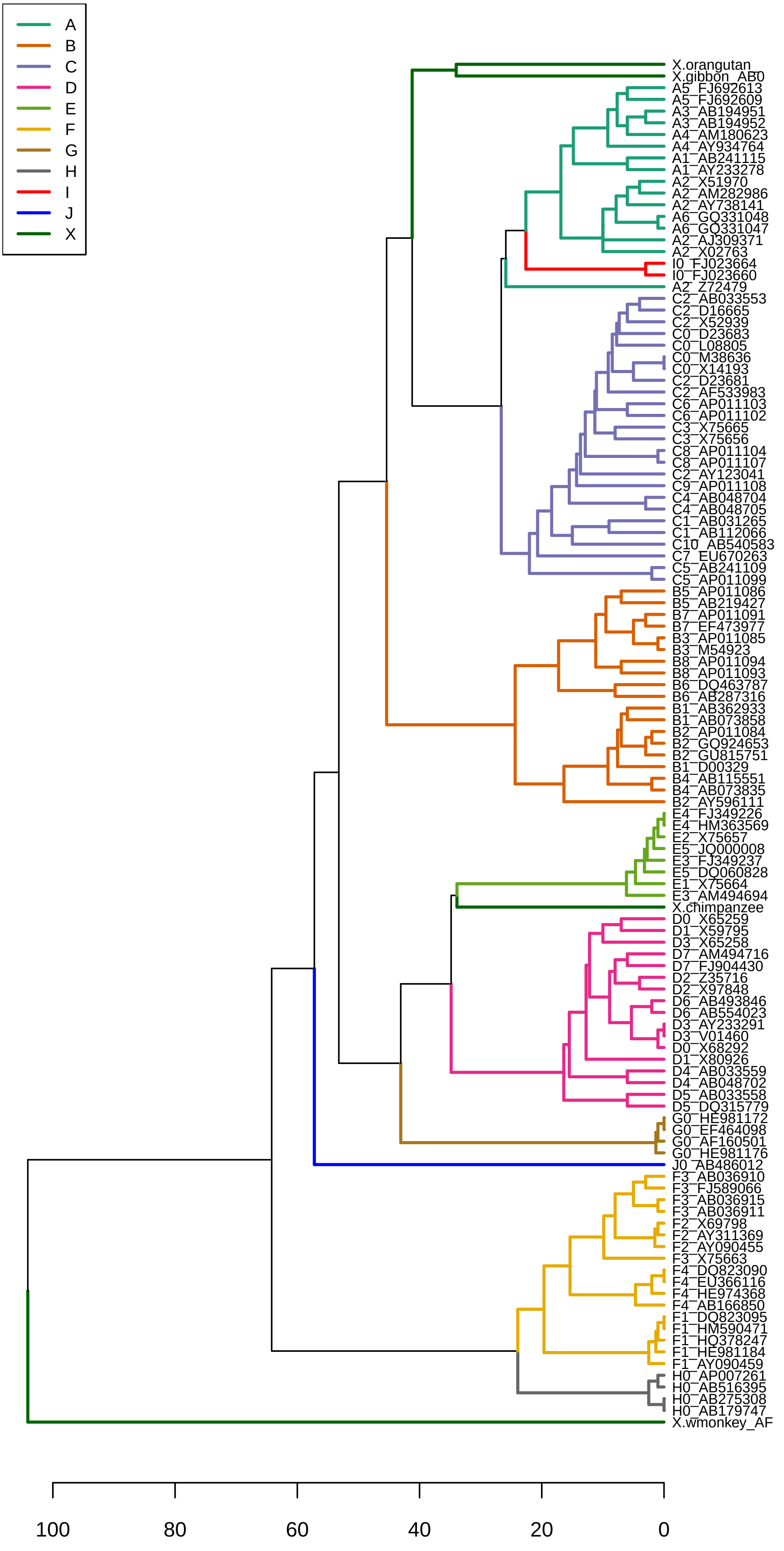

UPGMA tree (N): 2921−3278

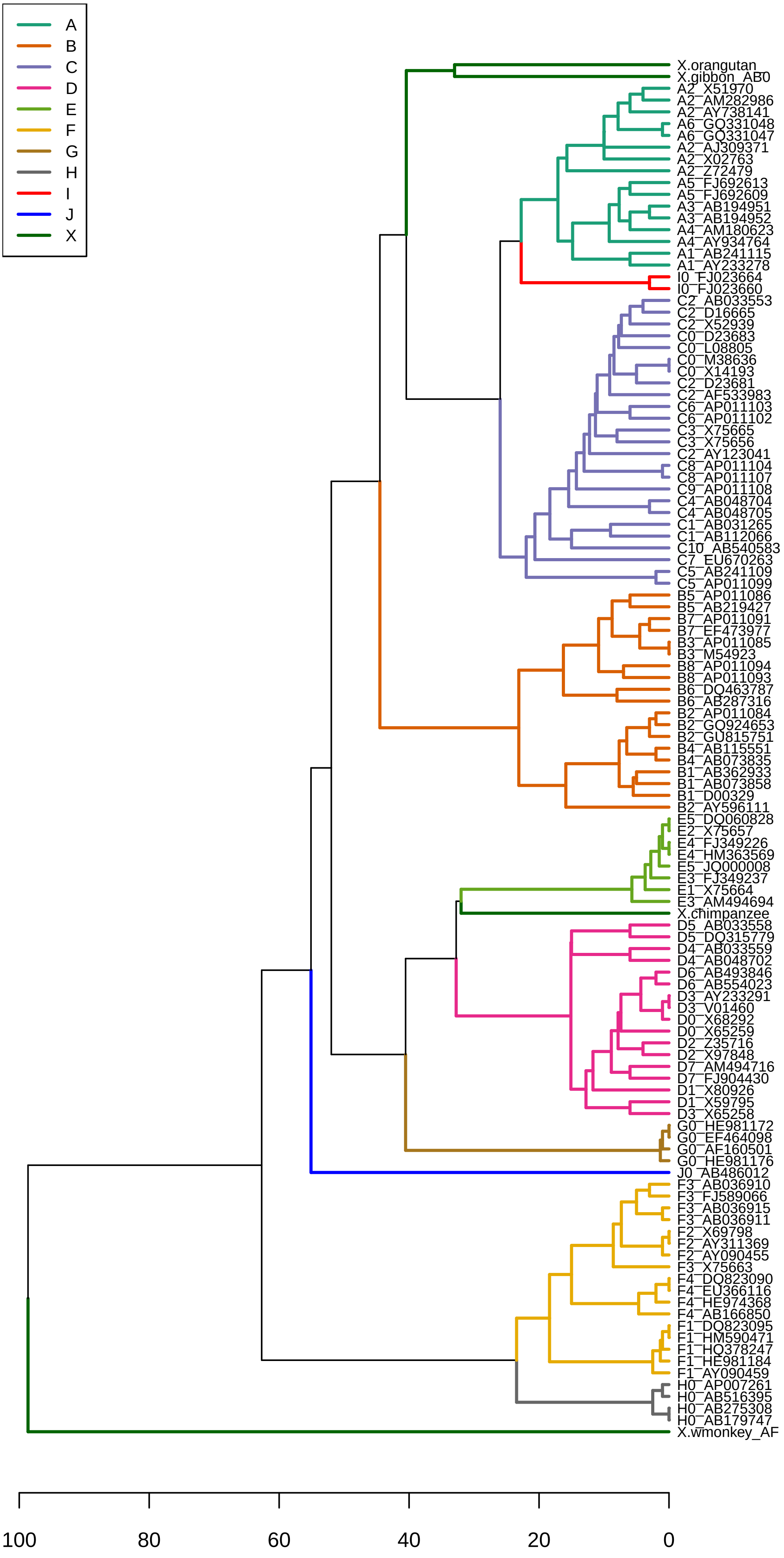

UPGMA tree (N): 2961−3278

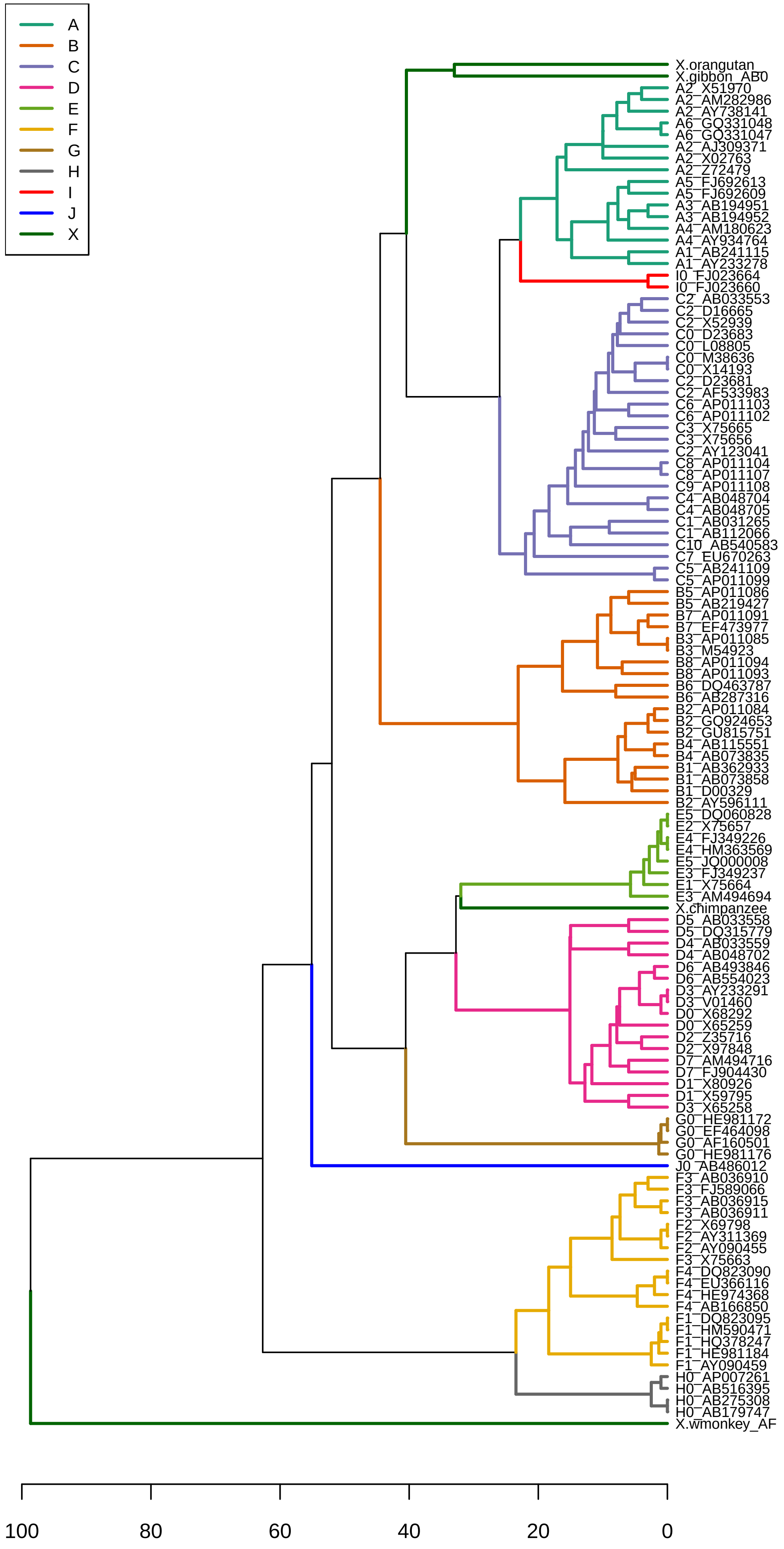

UPGMA tree (N): 3001−3278

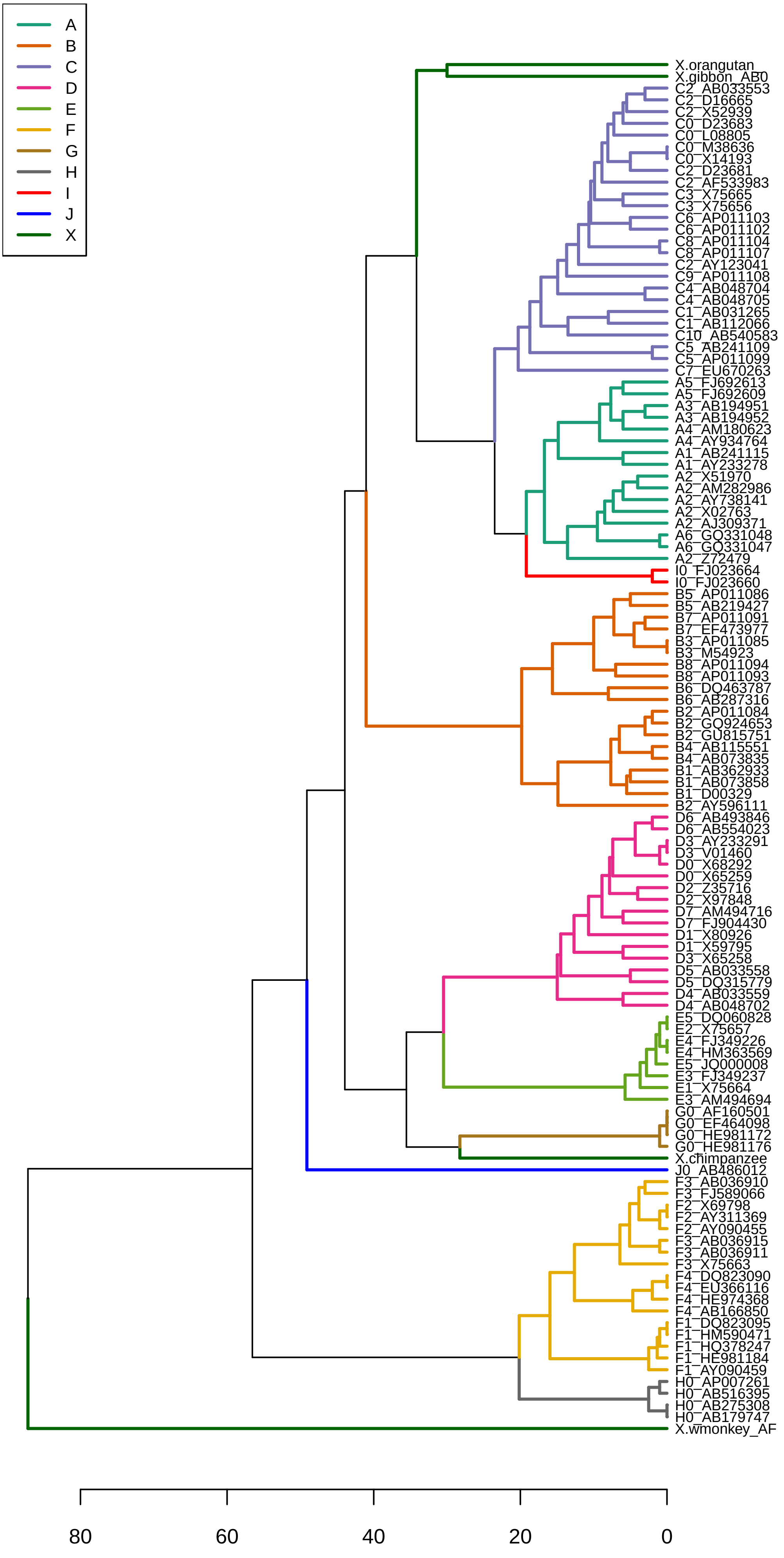

UPGMA tree (N): 3041−3278

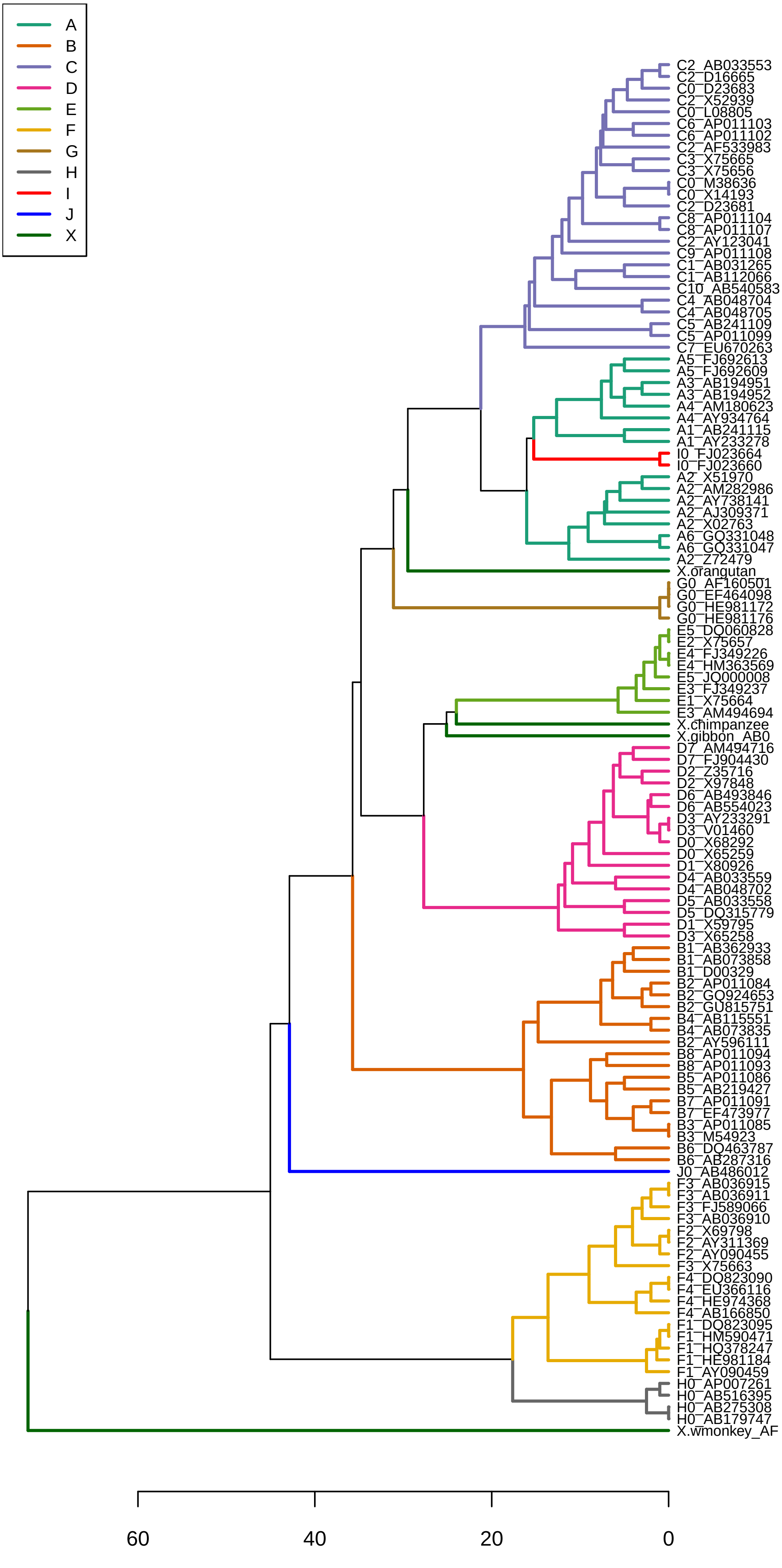

UPGMA tree (N): 3081−3278

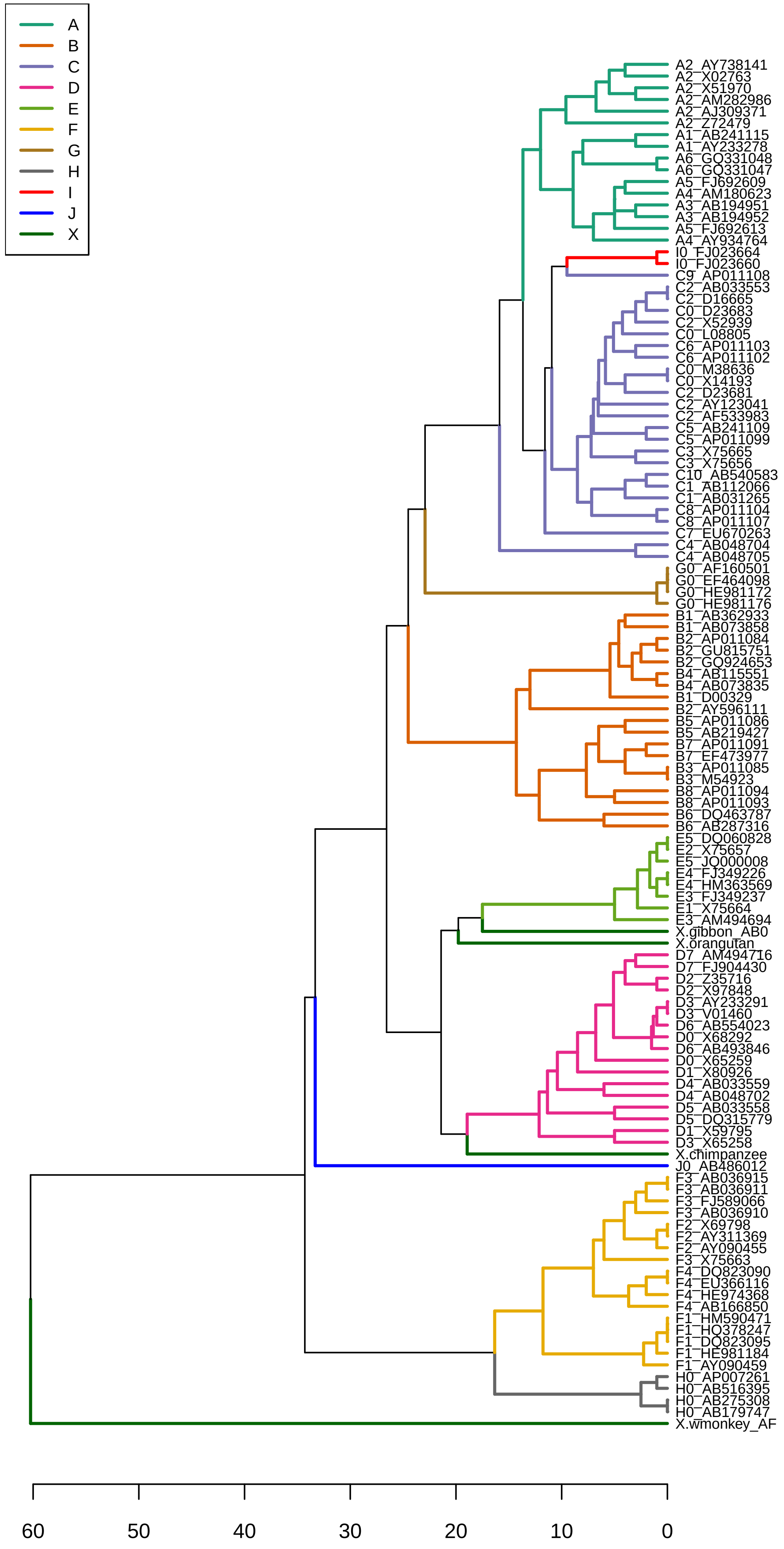

UPGMA tree (N): 3121−3278

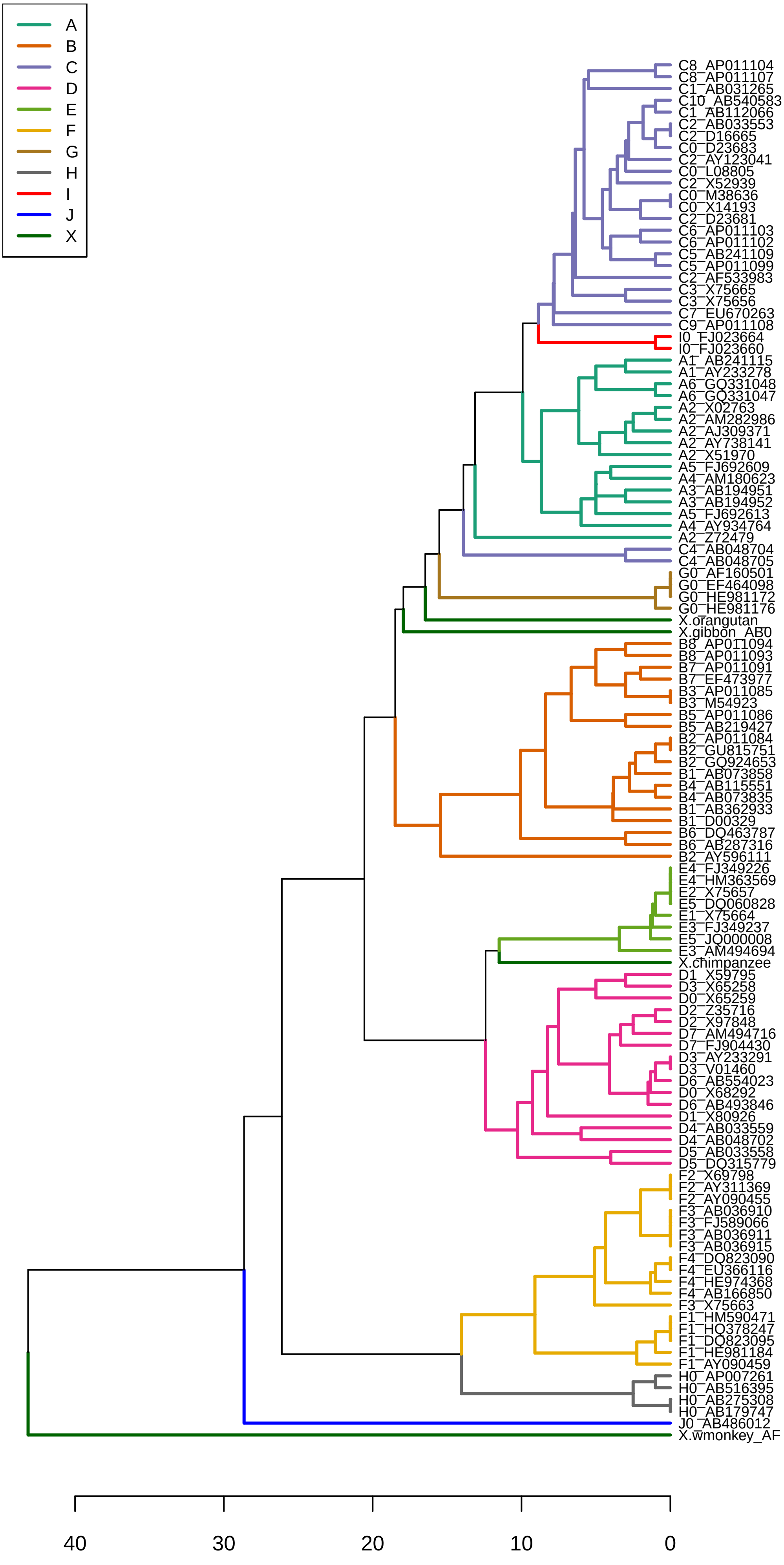

UPGMA tree (N): 3161−3278

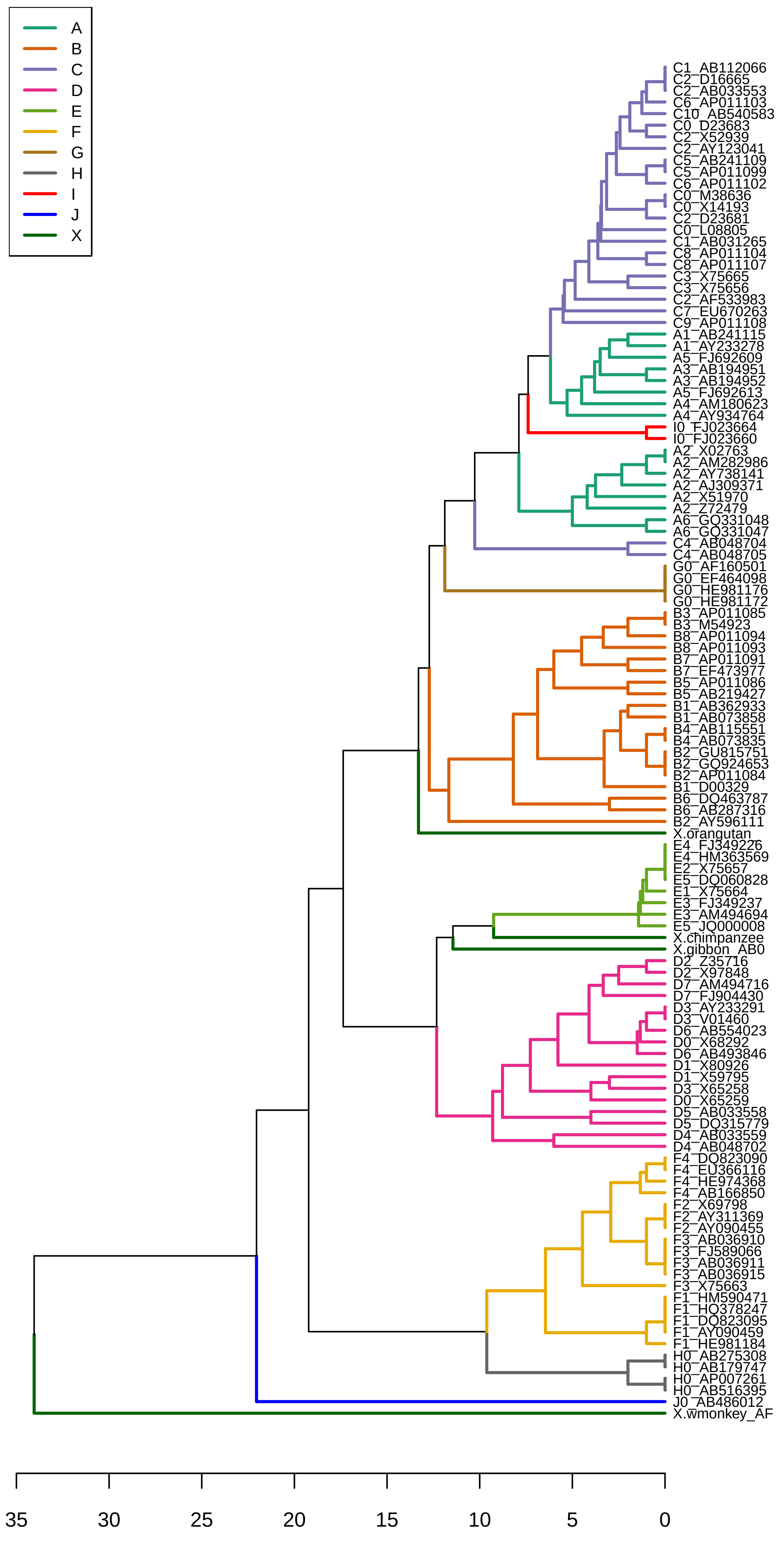

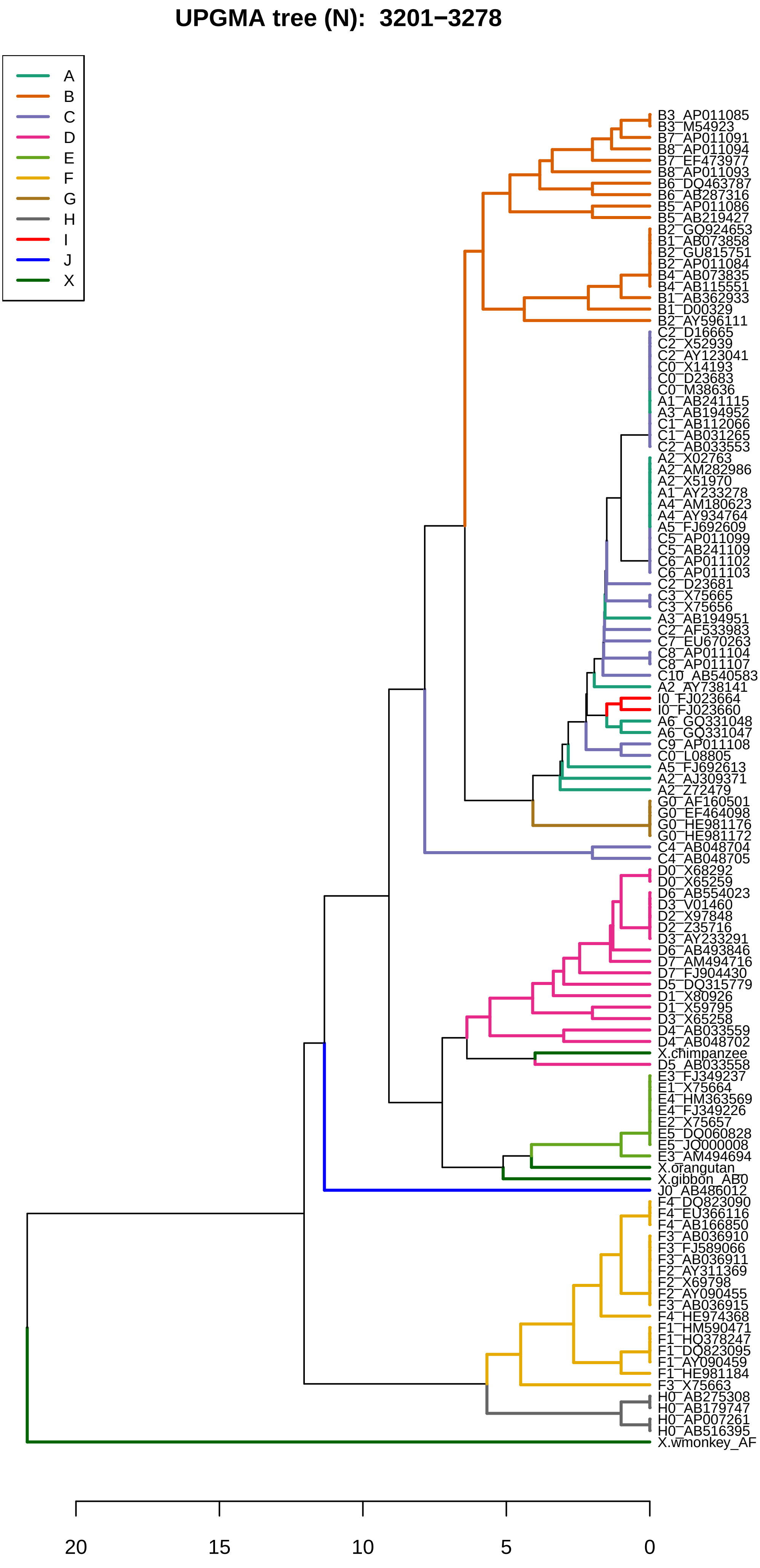
UPGMA tree (N): 3201−3278
A
B
C
D
E
F
G
H
I
J
X
B3_AP011085
B3_M54923
B7_AP011091
B8_AP011094
B7_EF473977
B8_AP011093
B6_DQ463787
B6_AB287316
B5_AP011086
B5_AB219427
B2_GQ924653
B1_AB073858
B2_GU815751
B2_AP011084
B4_AB073835
B4_AB115551
B1_AB362933
B1_D00329
B2_AY596111
C2_D16665
C2_X52939
C2_AY123041
C0_X14193
C0_D23683
C0_M38636
A1_AB241115
A3_AB194952
C1_AB112066
C1_AB031265
C2_AB033553
A2_X02763
A2_AM282986
A2_X51970
A1_AY233278
A4_AM180623
A4_AY934764
A5_FJ692609
C5_AP011099
C5_AB241109
C6_AP011102
C6_AP011103
C2_D23681
C3_X75665
C3_X75656
A3_AB194951
C2_AF533983
C7_EU670263
C8_AP011104
C8_AP011107
C10_AB540583
A2_AY738141
I0_FJ023664
I0_FJ023660
A6_GQ331048
A6_GQ331047
C9_AP011108
C0_L08805
A5_FJ692613
A2_AJ309371
A2_Z72479
G0_AF160501
G0_EF464098
G0_HE981176
G0_HE981172
C4_AB048704
C4_AB048705
D0_X68292
D0_X65259
D6_AB554023
D3_V01460
D2_X97848
D2_Z35716
D3_AY233291
D6_AB493846
D7_AM494716
D7_FJ904430
D5_DQ315779
D1_X80926
D1_X59795
D3_X65258
D4_AB033559
D4_AB048702
X.chimpanzee
D5_AB033558
E3_FJ349237
E1_X75664
E4_HM363569
E4_FJ349226
E2_X75657
E5_DQ060828
E5_JQ000008
E3_AM494694
X.orangutan
X.gibbon_AB0
J0_AB486012
F4_DQ823090
F4_EU366116
F4_AB166850
F3_AB036910
F3_FJ589066
F3_AB036911
F2_AY311369
F2_X69798
F2_AY090455
F3_AB036915
F4_HE974368
F1_HM590471
F1_HQ378247
F1_DQ823095
F1_AY090459
F1_HE981184
F3_X75663
H0_AB275308
H0_AB179747
H0_AP007261
H0_AB516395
X.wmonkey_AF
20
15
10
5
0

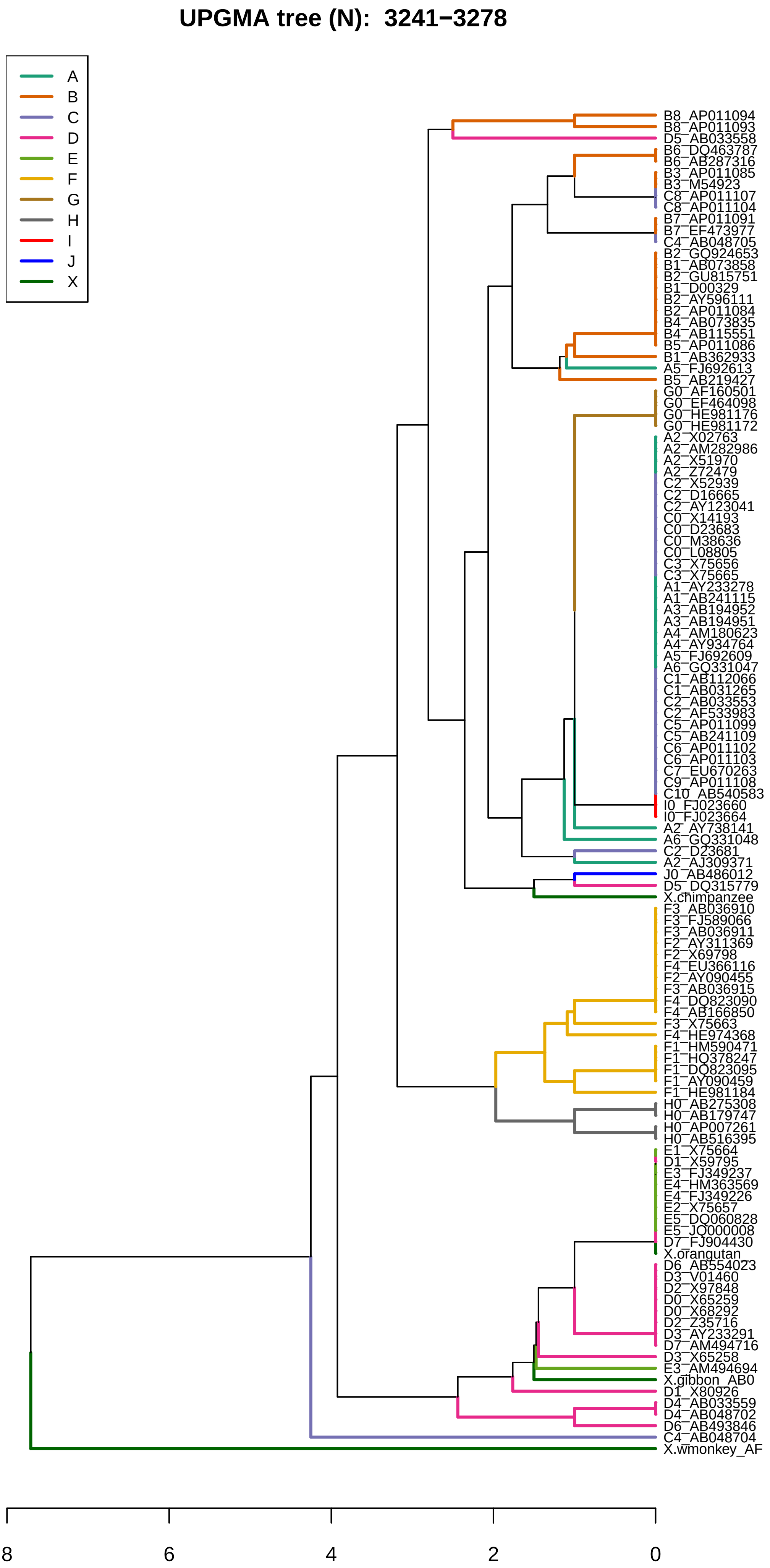
UPGMA tree (N): 3241−3278
A
B
C
D
E
F
G
H
I
J
X
B8_AP011094
B8_AP011093
D5_AB033558
B6_DQ463787
B6_AB287316
B3_AP011085
B3_M54923
C8_AP011107
C8_AP011104
B7_AP011091
B7_EF473977
C4_AB048705
B2_GQ924653
B1_AB073858
B2_GU815751
B1_D00329
B2_AY596111
B2_AP011084
B4_AB073835
B4_AB115551
B5_AP011086
B1_AB362933
A5_FJ692613
B5_AB219427
G0_AF160501
G0_EF464098
G0_HE981176
G0_HE981172
A2_X02763
A2_AM282986
A2_X51970
A2_Z72479
C2_X52939
C2_D16665
C2_AY123041
C0_X14193
C0_D23683
C0_M38636
C0_L08805
C3_X75656
C3_X75665
A1_AY233278
A1_AB241115
A3_AB194952
A3_AB194951
A4_AM180623
A4_AY934764
A5_FJ692609
A6_GQ331047
C1_AB112066
C1_AB031265
C2_AB033553
C2_AF533983
C5_AP011099
C5_AB241109
C6_AP011102
C6_AP011103
C7_EU670263
C9_AP011108
C10_AB540583
I0_FJ023660
I0_FJ023664
A2_AY738141
A6_GQ331048
C2_D23681
A2_AJ309371
J0_AB486012
D5_DQ315779
X.chimpanzee
F3_AB036910
F3_FJ589066
F3_AB036911
F2_AY311369
F2_X69798
F4_EU366116
F2_AY090455
F3_AB036915
F4_DQ823090
F4_AB166850
F3_X75663
F4_HE974368
F1_HM590471
F1_HQ378247
F1_DQ823095
F1_AY090459
F1_HE981184
H0_AB275308
H0_AB179747
H0_AP007261
H0_AB516395
E1_X75664
D1_X59795
E3_FJ349237
E4_HM363569
E4_FJ349226
E2_X75657
E5_DQ060828
E5_JQ000008
D7_FJ904430
X.orangutan
D6_AB554023
D3_V01460
D2_X97848
D0_X65259
D0_X68292
D2_Z35716
D3_AY233291
D7_AM494716
D3_X65258
E3_AM494694
X.gibbon_AB0
D1_X80926
D4_AB033559
D4_AB048702
D6_AB493846
C4_AB048704
X.wmonkey_AF
8
6
4
2
0
