# Supplementary material for: Complex Genotype Mixtures Analyzed by Deep Sequencing in Two Different Regions of Hepatitis B Virus
Source: PLoS One. 2015 Dec 29;10(12):e0144816. doi: 10.1371/journal.pone.0144816 (PMC4695080; doi:10.1371/journal.pone.0144816)
Supplement: S7 File — (PDF) [file pone.0144816.s007.pdf]

Distances to genotype A

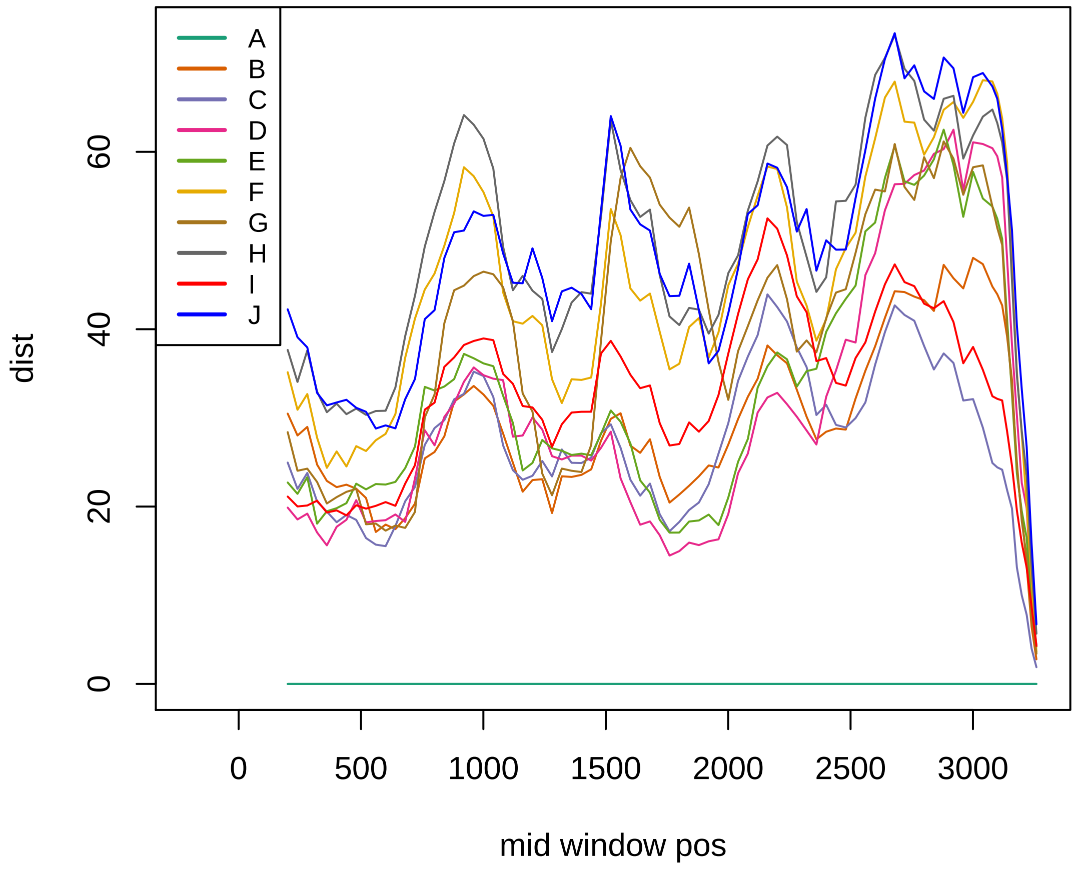

Distances to genotype B

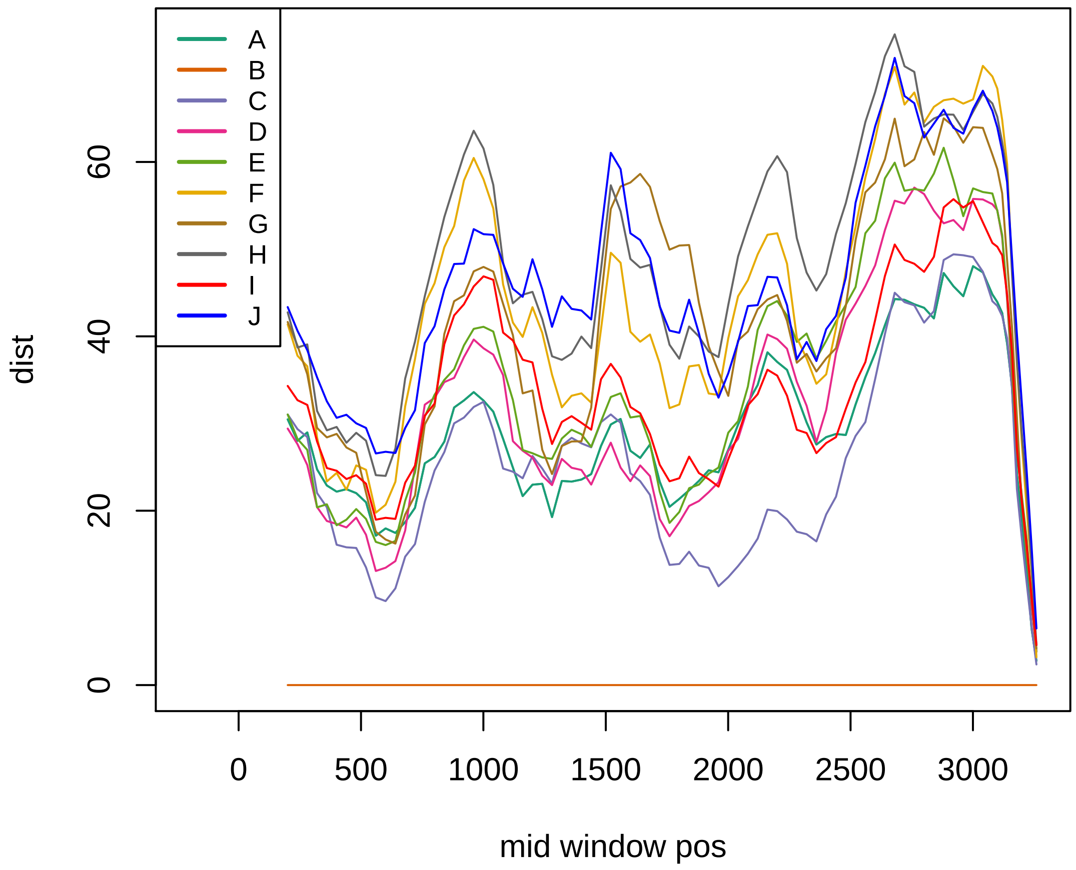

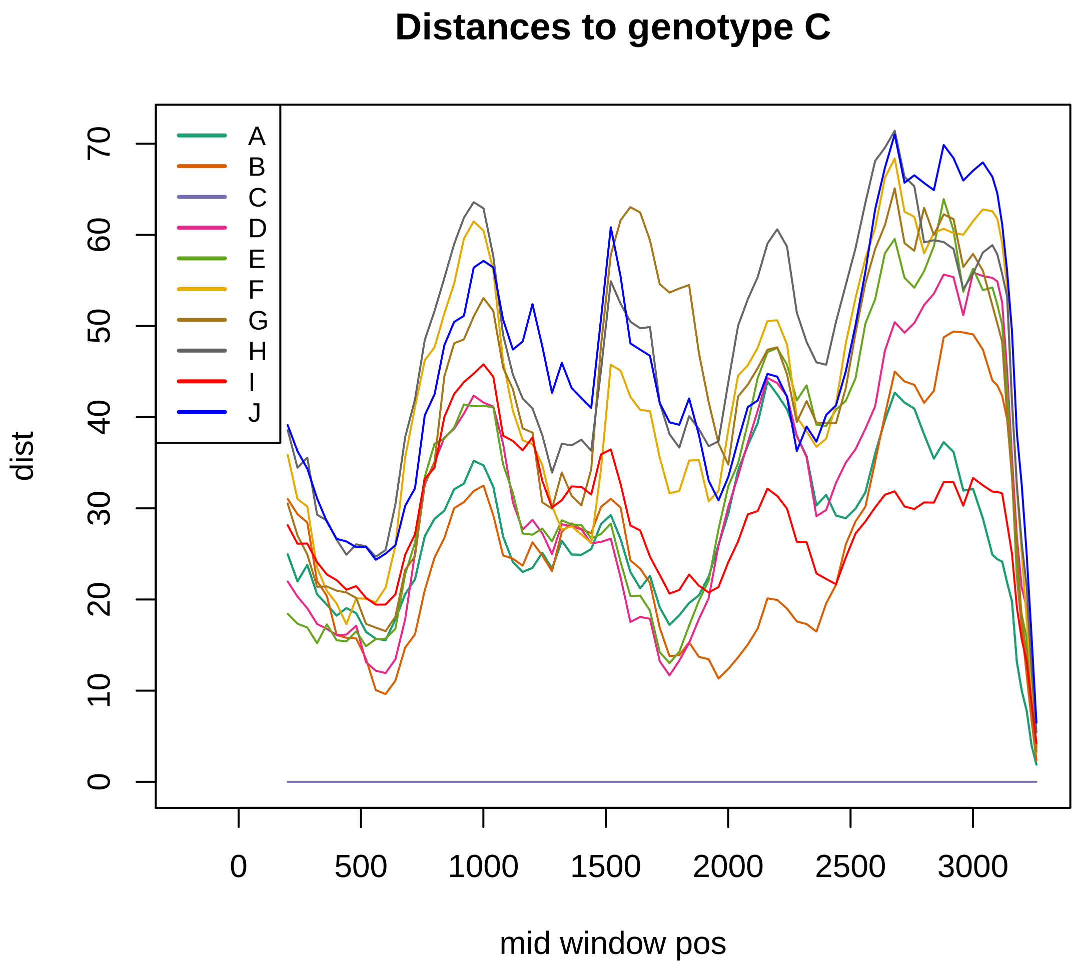

Distances to genotype C
A
B
C
D
E
F
G
H
I
J
dist
0
10
20
30
40
50
60
70
0
500
1000
1500
2000
2500
3000
mid window pos

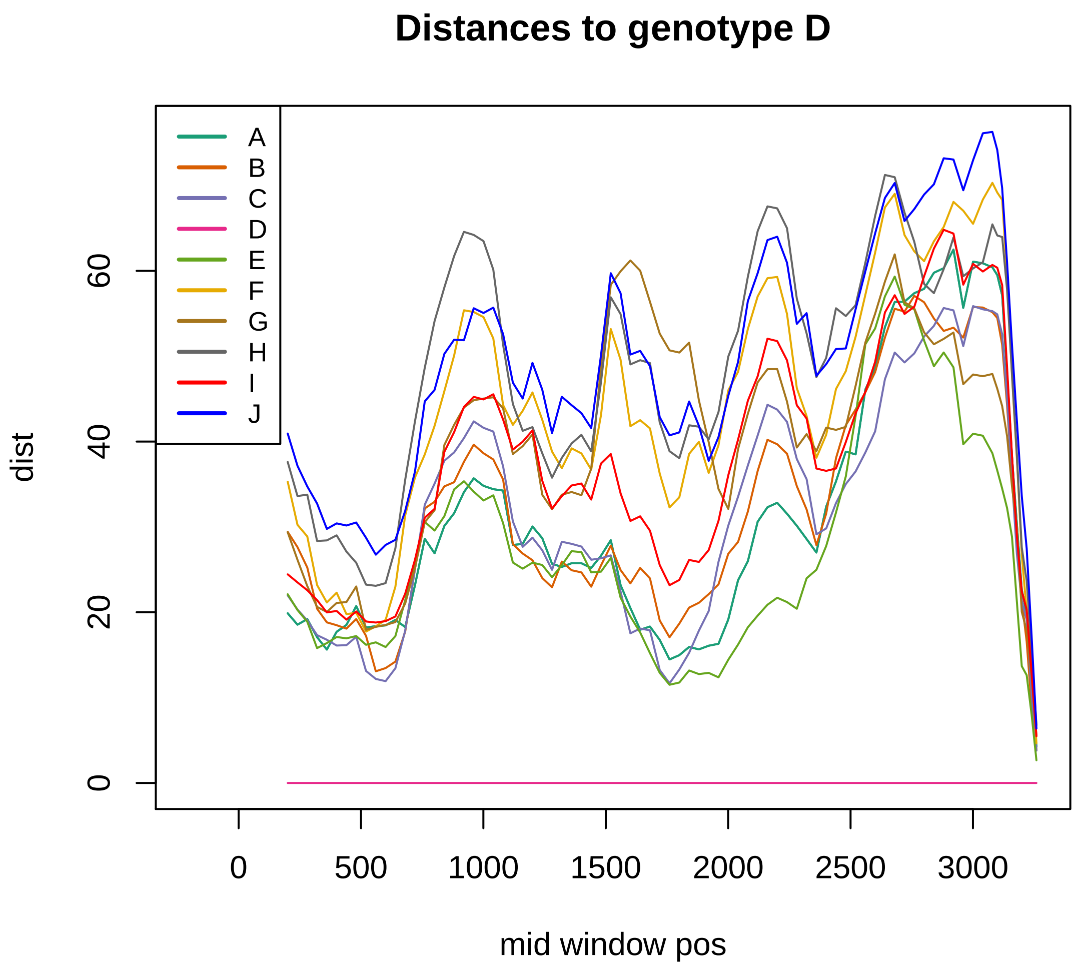

Distances to genotype D
A
B
C
D
E
F
G
H
I
J
dist
0
20
40
60
0
500
1000
1500
2000
2500
3000
mid window pos

Distances to genotype E

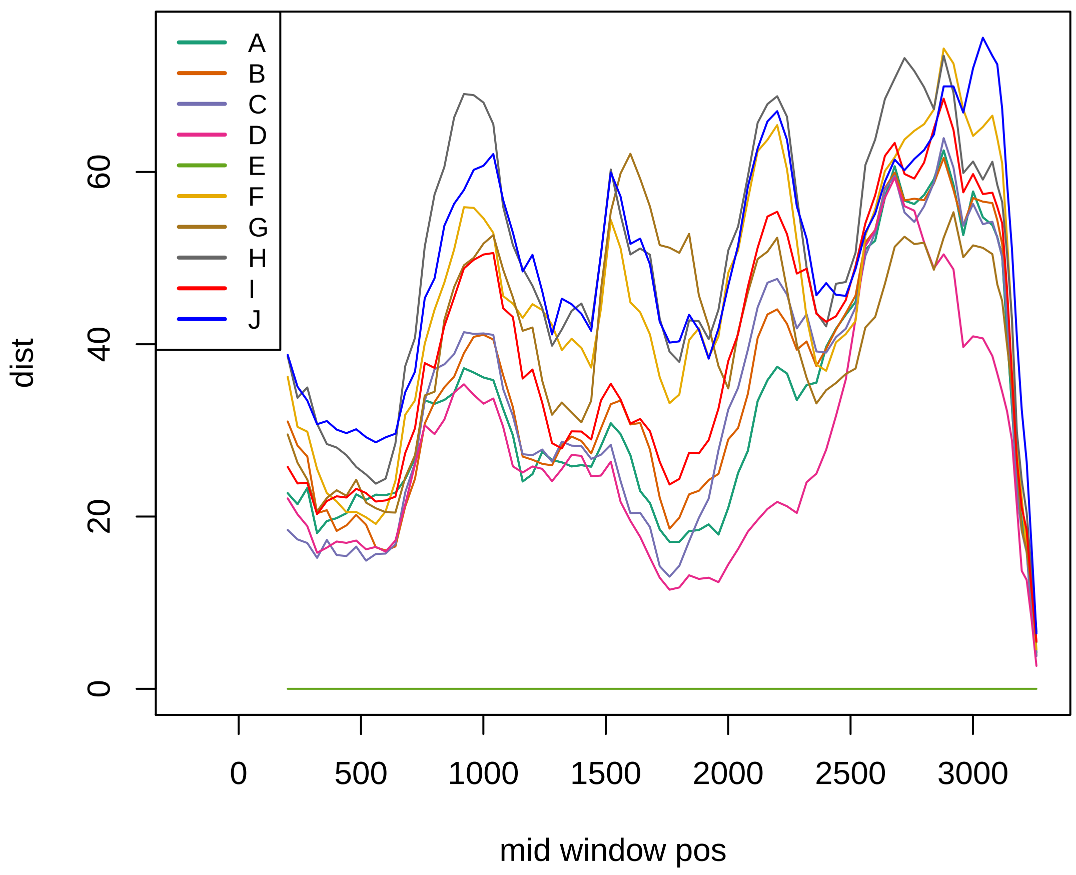

Distances to genotype F

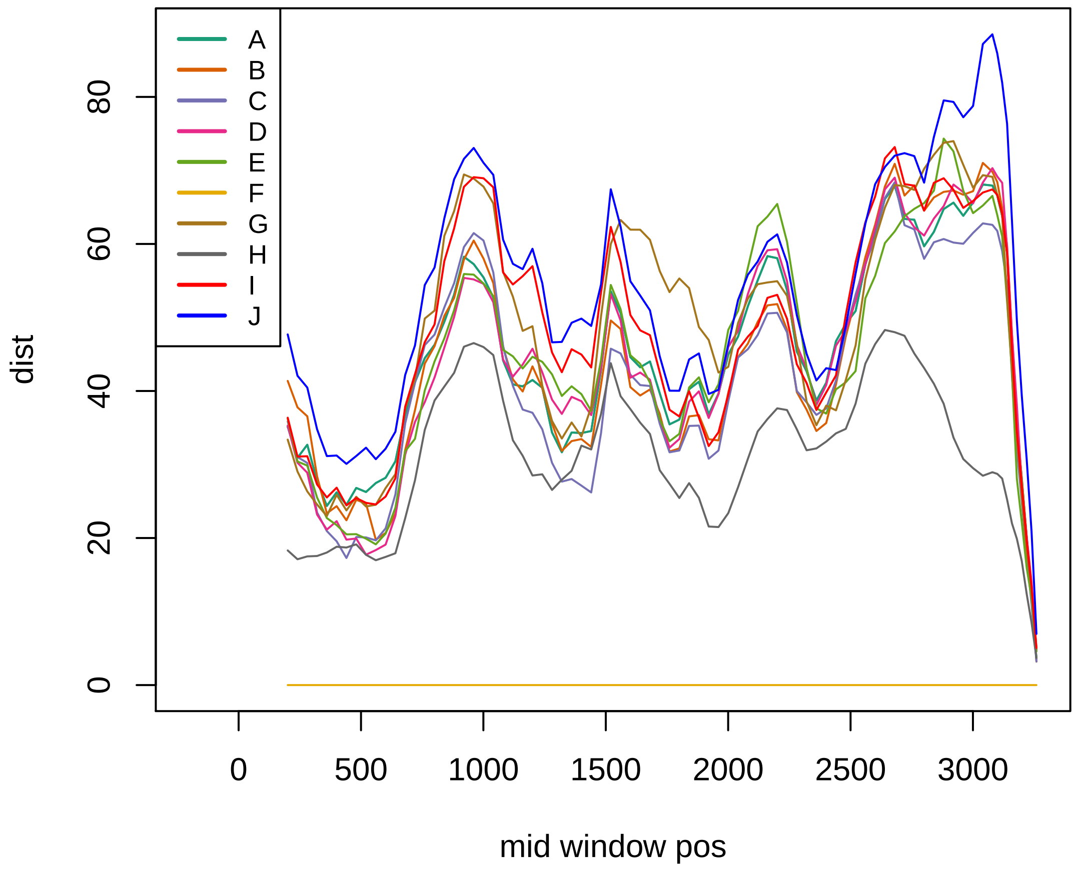

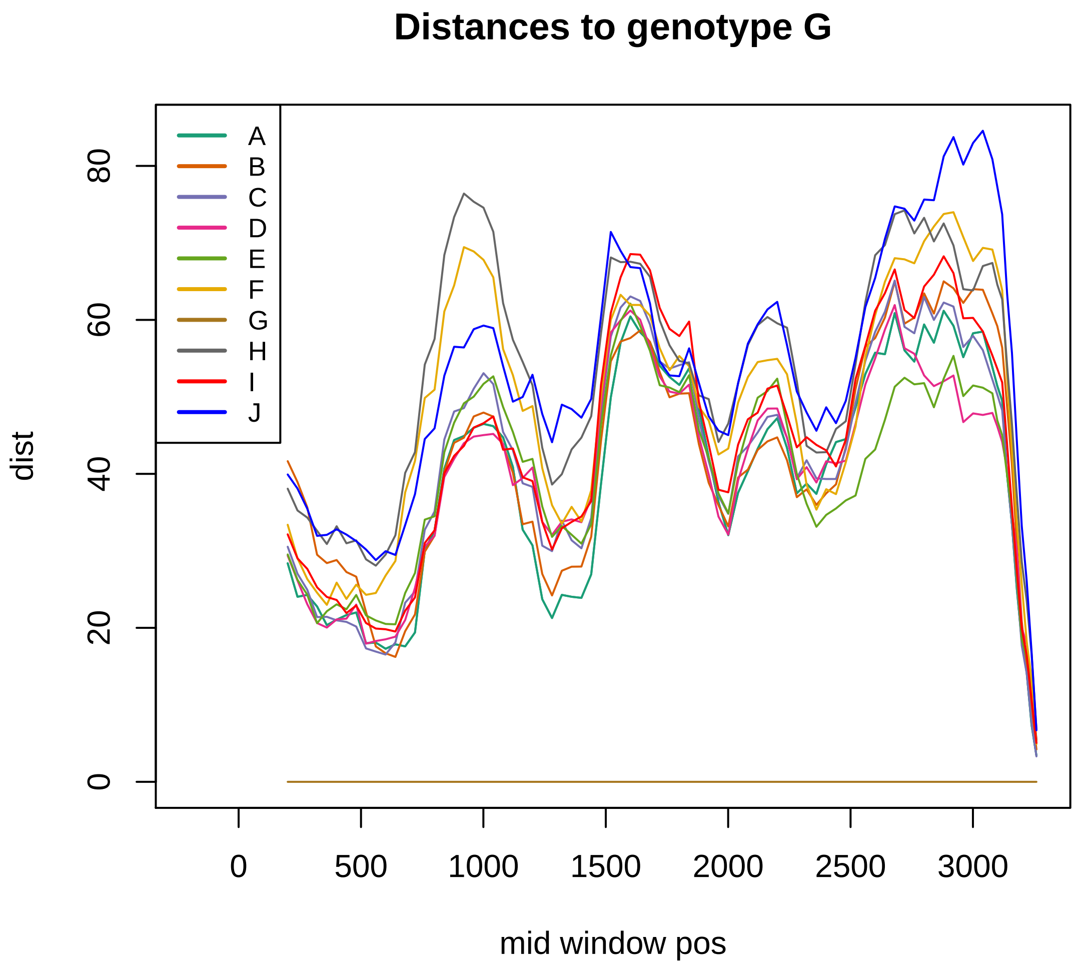
Distances to genotype G
A
B
C
D
E
F
G
H
I
J
dist
0
20
40
60
80
0
500
1000
1500
2000
2500
3000
mid window pos

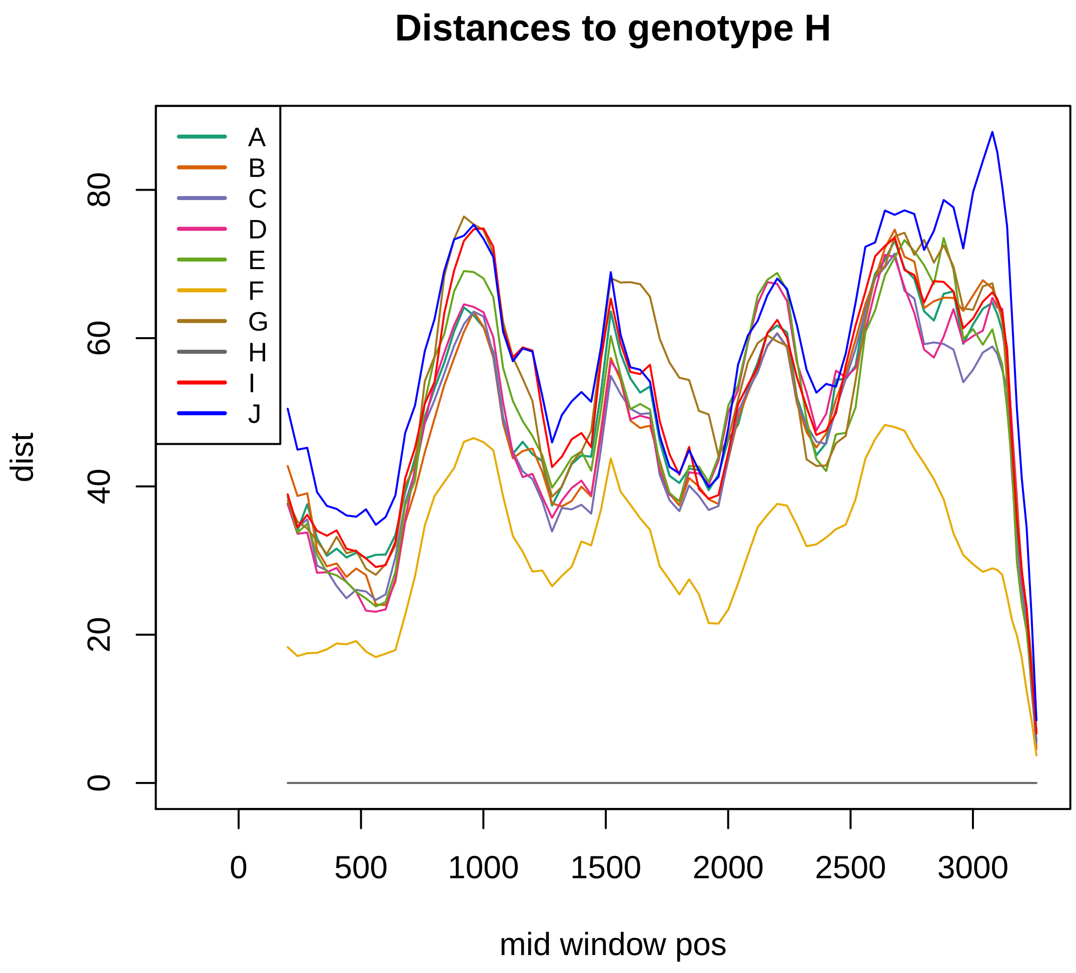
Distances to genotype H
A
B
C
D
E
F
G
H
I
J
dist
0
20
40
60
80
0
500
1000
1500
2000
2500
3000
mid window pos

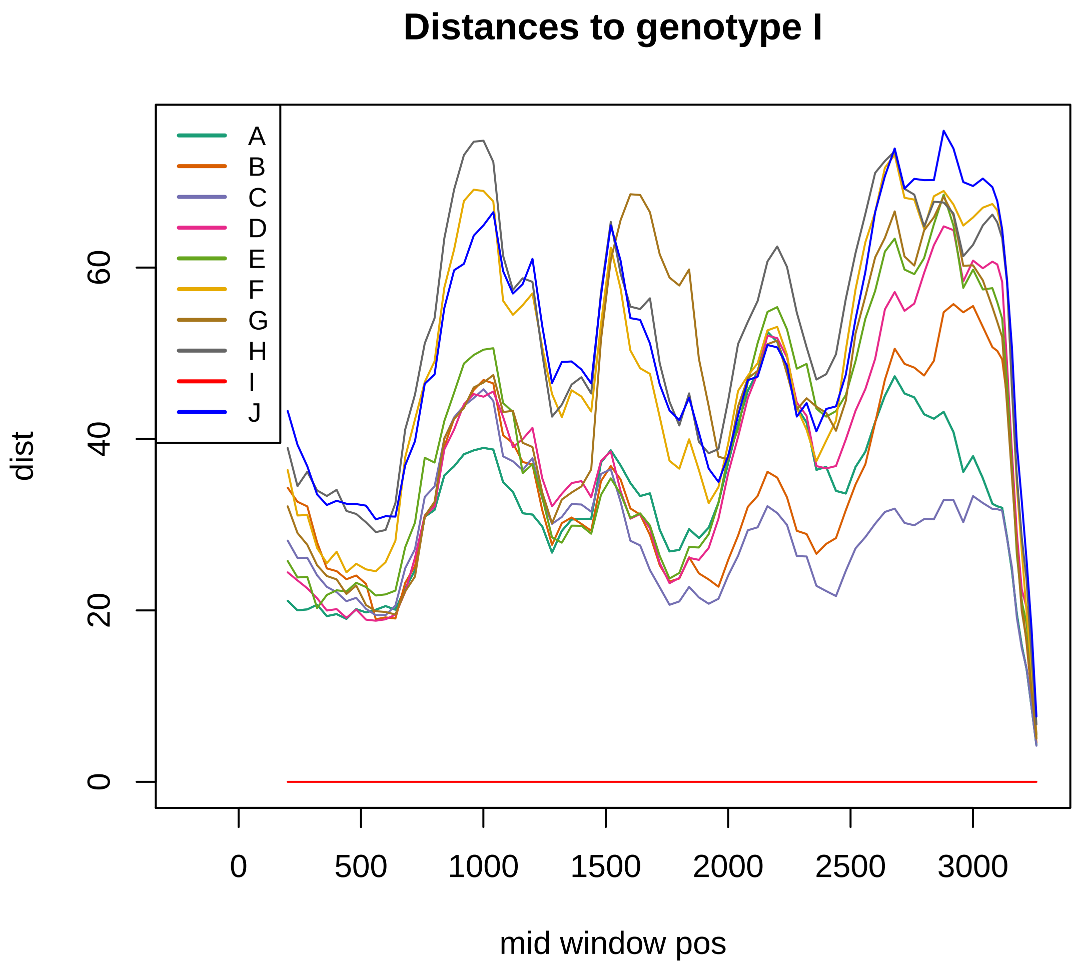
Distances to genotype I
A
B
C
D
E
F
G
H
I
J
dist
0
20
40
60
0
500
1000
1500
2000
2500
3000
mid window pos

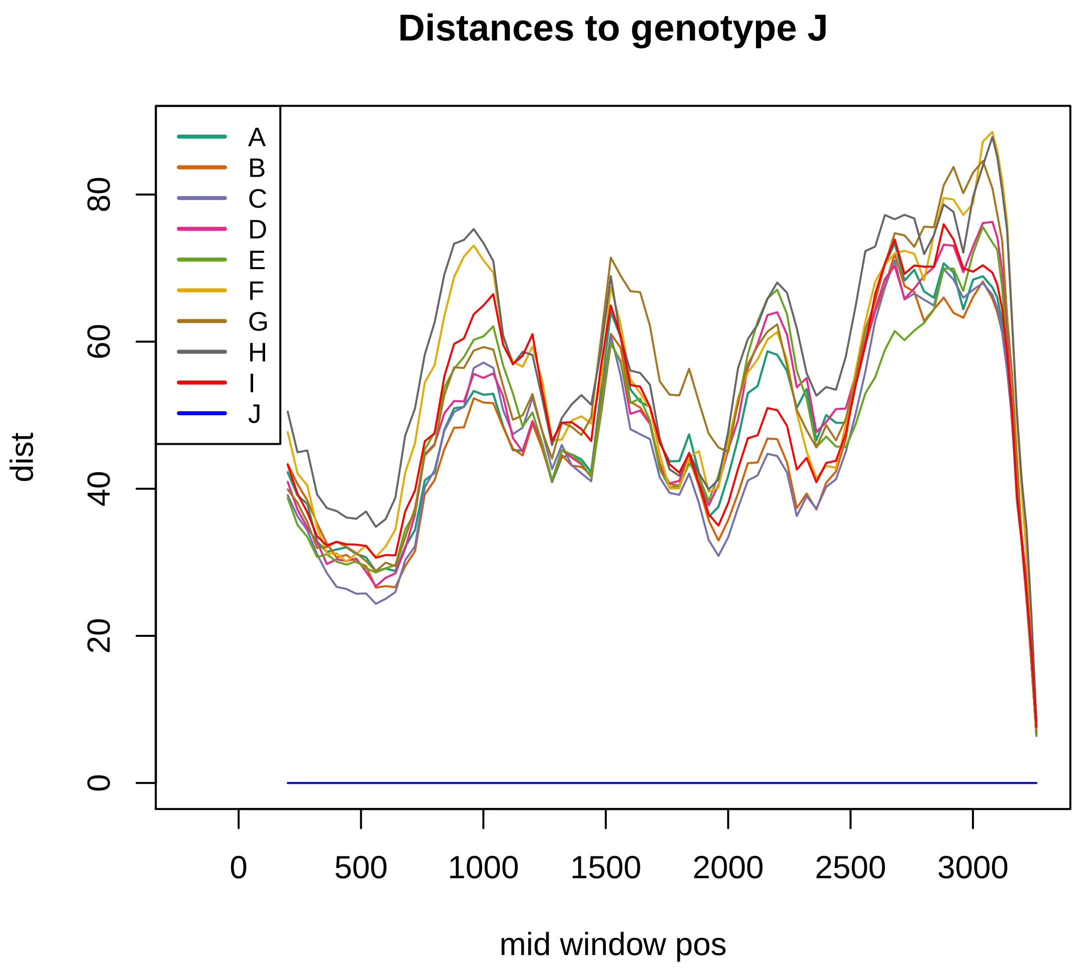
Distances to genotype J
A
B
C
D
E
F
G
H
I
J
dist
0
20
40
60
80
0
500
1000
1500
2000
2500
3000
mid window pos
