# Supplementary material for: Complex Genotype Mixtures Analyzed by Deep Sequencing in Two Different Regions of Hepatitis B Virus
Source: PLoS One. 2015 Dec 29;10(12):e0144816. doi: 10.1371/journal.pone.0144816 (PMC4695080; doi:10.1371/journal.pone.0144816)
Supplement: S1 Protocol — Detailed description of amplification of the P/S and X/preCore regions, UDPS data treatment, and genotyping, description of genotyping and examination of HBV genomtypes reference sequence. (DOCX) [file pone.0144816.s008.docx]

**S1 Protocol**

**SUPPLEMENTARY MATERIALS AND METHODS**

***P/S fragment amplicon preparation***

The first PCR was carried out using the following primers: sense 5’-***GTAAAACGACGGCCAGT***ACCTGTATTCCCATCCCAT-3’ (nt positions 596-614) and anti-sense 5’-***AACAGCTATGACCATG***ACATACTTTCCAATCAATAGG-3’ (nt positions 970-990), both of which include an M13 universal adaptor sequence at the 5’ end (in bold and italics). Briefly, PCR was performed using 1x Pfu Ultra II polymerase buffer, 0.25 mM of each dNTP, 0.2 µM of forward and reverse primers, and 2.5 U of Pfu Ultra II DNA polymerase (Stratagene, Agilent Technologies) in a final volume of 10 µL. After a single denaturation step of 2 min at 95ºC, samples underwent 35 cycles of 20 s at 95ºC, 20 s at 55ºC, and 15 s at 72ºC, and a single final 3-min step at 72ºC. The nested PCR was performed using a specific pair of primers for each sample: sense 5’-CGTATCGCCTCCCTCGCGCCATCAG-**MID**-***GTAAAACGACGGCCAGT***-3’ and anti-sense 5’-CTATGCGCCTTGCCAGCCCGCTCAG-**MID**-***AACAGCTATGACCATG****-*3’. These primers contain the 5’ sequences A and B, which are adaptors for the elements of the UDPS system, followed by a unique identifier that enables grouping of the sequences derived from each sample (MID sequence, in bold), and the same M13 universal adaptor sequences as the primers used in the first PCR (in bold and italics). To avoid errors and facilitate the complex process of using a different pair of primers for each sample, universal nested primers were lyophilized and preloaded in 96-well PCR plates, as described in our previous report (1). Nested PCR was performed using 5 µL Pfu Ultra II polymerase buffer, 10mM of each dNTP, 5 µmol of forward and reverse primers, 10% DMSO, and 2.5 U of Pfu Ultra II DNA polymerase (Stratagene, Agilent Technologies) in a final volume of 50 µL. After a single denaturation step of 2 min at 95ºC, samples underwent 20 cycles of 30 s at 95ºC, 30 s at 60ºC, and 30 s at 72ºC and a single final 5-min step at 72ºC. Finally, 505-bp amplicons were obtained.

***X/preCore fragment amplicon preparation***

This UDPS-analyzed fragment was also amplified by nested PCR. The first PCR was carried out using the following primers: sense 5’-***GTAAAACGACGGCCAGT*** TGTGCACTTCGCC/TTCACC-3’ (nt positions 1580-1597) and anti-sense 5’-***AACAGCTATGACCATG***AGA/TAGCTCCAAATTCTTTATAAGG-3’ (nt positions 1913-1936). Briefly, PCR was performed using 1 µL Pfu Ultra II polymerase buffer, 10mM of each dNTP, 0.4 µmol of forward and reverse primers, 5% DMSO, and 2.5 U of Pfu Ultra II DNA polymerase in a final volume of 10 µL. After a single denaturation step of 2 min at 95ºC, samples underwent 35 cycles of 20 s at 95ºC, 20 s at 67ºC and 15s at 72ºC, and a single final 3-min step at 72ºC. A second nested PCR was performed using the same primers and experimental protocol described for the P/S fragment. Finally, 468 bp amplicons were obtained.

***PCR products isolation***

The PCR products were isolated from 1.5% agarose gel. The quality and length of the HBV DNA amplicons was verified with the Agilent Bioanalyzer and DNA1000 kit (LifeScience, Santa Clara, CA, USA), and quantified using Quant-iT Picogreen dsDNA reagent (Invitrogen).

***UDPS data treatment***

Briefly, haplotypes carrying sequencing errors were eliminated as follows: 1) By discarding all sequences with more than one indetermination, those that did not cover the full amplicon, and those with an identity relative to a given reference sequence or to the dominant haplotype below 70%; 2) By collapsing the resulting sequences to unique haplotypes with their corresponding frequencies and taking haplotypes in amounts above 0.1% that were common to the forward and reverse strands; and 3) By removing all common forward and reverse haplotypes with an abundance below a noise level, estimated as being 0.25% for HBV with coverage of around 10,000 reads. The resulting haplotypes were denominated consensus haplotypes (CH).

**GENOTYPING**

The consensus haplotypes were clustered with an identity above 97% for the X/preCore amplicon and 96% for the P/S amplicon. The centroid of each cluster was taken as the most abundant haplotype in the cluster. By analysis of the distances from pairs of reference sequences, identity thresholds were established as being higher than the maximum distance within a single genotype, but lower than the minimum distance between pairs of sequences of different genotypes.

The consensus haplotype centroids were multiple aligned with a set of reference sequences taken from GenBank (**S1 Supporting information**) by Clustal Omega and genotyping was done by distance-based discriminant analysis (2,3) which takes into account the intraclass variability of each genotype. The genetic distances were computed according to the Kimura-80 model (4) Briefly, to correct for intraclass variability in a given sequence to be classified, the DB rule obtains a discriminant value for each genotype as the mean squared genetic distance from the problem sequence to all sequences in the genotype, minus the mean squared genetic distance between the reference sequences within the genotype. The sequence is then classified according to the minimum discriminant value obtained. Visualization of genetic distances between sequences is provided by UPGMA trees and Multidimensional Scaling (MDS) plots (5).

**EXAMINATION OF HBV GENOTYPE REFERENCE SEQUENCES**

The genotype discriminant capacity of a set of selected whole genome sequences from GenBank was studied using the DB rule with the leave-one-out method. Each whole genome sequence in the set was classified using the remaining sequences. The clustering capacity of these reference sequences was further examined by UPGMA trees and by plots on the first three components of a multimensional scaling analysis of the matrix of genetic distances between pairs of reference sequences. The accessions of these references sequences and the results of the analysis including tables and plots are presented in **S1 Supporting information**. All sequences could be perfectly classified except for Gen/J, for which there is only one sequence.

The set of reference sequences was multiple aligned and trimmed to positions 615 to 969, covering the P/S amplicon. Sequences that were equivalent after trimming were removed, leaving only one. The same analysis of discriminant capacity described above was conducted. This region still offers good genotyping capacity despite its reduced dimension, with all sequences correctly classified except for two Gen/C sequences misclassified as Gen/A. The two Gen/C sequences clustered outside the Gen/C group in both the UPGMA tree and MDS plots. The accessions of these reference sequences and the results of the analysis including tables and plots are shown in **S2 Supporting information**.

The same set was also trimmed to positions 1596 to 1912 in the X/preCore region. This amplicon showed a clear intermixing of Gen/D and Gen/E sequences and of Gen/C and Gen/I on the other. The two conflicting Gen/C sequences in the P/S region, however, clustered nicely with the other C sequences. The accession numbers of these reference sequences and the results of the analysis including tables and plots are summarized in **S3 Supporting information.**

Sliding windows exploration of 400 nucleotides in steps of 40 in sequences from the whole genome showed that the region spanning positions 2600 to 3000 in the pre-S region was the 400-nucleotide amplicon that best classified genotypes and most subtypes, except for Gen/I which classifies inside the Gen/C cluster (**S6 and S7 Supporting information)**.

**Supplementary references**

1. Quer J, Gregori J, Rodríguez-Frias F, Buti M, Madejon A, Perez-del-Pulgar S, et al. High-resolution hepatitis C virus subtyping using NS5B deep sequencing and phylogeny, an alternative to current methods. J Clin Microbiol. 2015 Jan;53(1):219–26.

2. Cuadras C. Distance analysis in discrimination and classification using both continuous and categorical variables. In: Dodge Y, editor. Statistical AData analysis and Interference. Amsterdam: Elsevier; 1989. p. 459–73.

3. Cuadras C. A distance approach to discriminant analysis and its properties. Mathematics preprint series. Barcelona; 1991.

4. Kumar S, Gadagkar SR. Efficiency of the neighbor-joining method in reconstructing deep and shallow evolutionary relationships in large phylogenies. J Mol Evol. 2000 Dec;51(6):544–53.

5. Borg I, PJF G. Modern multidimensional scaling: theory and applications. 2nd ed. New York: Springer; 2005.
