# Supplementary material for: Complex Genotype Mixtures Analyzed by Deep Sequencing in Two Different Regions of Hepatitis B Virus
Source: PLoS One. 2015 Dec 29;10(12):e0144816. doi: 10.1371/journal.pone.0144816 (PMC4695080; doi:10.1371/journal.pone.0144816)
Supplement: S1 Table — (DOCX) [file pone.0144816.s009.docx]

**S1 Table**  Biosample accession numbers for each sample and regions analyzed

| **Patient** | **Sample** | **Region** | **Biosample accession number** |
| --- | --- | --- | --- |
| 1 | First | P/S | SAMN03944753 |
| 1 | First | x/preCore | SAMN03944754 |
| 1 | Second | P/S | SAMN03944757 |
| 1 | Second | x/preCore | SAMN03944758 |
| 1 | Third | P/S | SAMN03944755 |
| 1 | Third | x/preCore | SAMN03944756 |
| 2 | First | P/S | SAMN03944759 |
| 2 | First | x/preCore | SAMN03944760 |
| 2 | Second | P/S | SAMN03944763 |
| 2 | Second | x/preCore | SAMN03944764 |
| 2 | Third | P/S | SAMN03944761 |
| 2 | Third | x/preCore | SAMN03944762 |
| 3 | First | P/S | SAMN03944765 |
| 3 | First | x/preCore | SAMN03944766 |
| 3 | Second | P/S | SAMN03944769 |
| 3 | Second | x/preCore | SAMN03944770 |
| 3 | Third | P/S | SAMN03944767 |
| 3 | Third | x/preCore | SAMN03944768 |
| 4 | First | P/S | SAMN03944771 |
| 4 | First | x/preCore | SAMN03944772 |
| 4 | Second | P/S | SAMN03944775 |
| 4 | Second | x/preCore | SAMN03944776 |
| 4 | Third | P/S | SAMN03944773 |
| 4 | Third | x/preCore | SAMN03944774 |
| 5 | First | P/S | SAMN03944777 |
| 5 | First | x/preCore | SAMN03944778 |
| 5 | Second | P/S | SAMN03944781 |
| 5 | Second | x/preCore | SAMN03944782 |
| 5 | Third | P/S | SAMN03944779 |
| 5 | Third | x/preCore | SAMN03944780 |
| 6 | First | P/S | SAMN03944783 |
| 6 | First | x/preCore | SAMN03944784 |
| 6 | Second | P/S | SAMN03944787 |
| 6 | Second | x/preCore | SAMN03944788 |
| 6 | Third | P/S | SAMN03944785 |
| 6 | Third | x/preCore | SAMN03944786 |
| 7 | First | P/S | SAMN03944789 |
| 7 | First | x/preCore | SAMN03944790 |
| 7 | Second | P/S | SAMN03944793 |
| 7 | Second | x/preCore | SAMN03944794 |
| 7 | Third | P/S | SAMN03944791 |
| 7 | Third | x/preCore | SAMN03944792 |
| 8 | First | P/S | SAMN03944795 |
| 8 | First | x/preCore | SAMN03944796 |
| 8 | Second | P/S | SAMN03944799 |
| 8 | Second | x/preCore | SAMN03944800 |
| 8 | Third | P/S | SAMN03944797 |
| 8 | Third | x/preCore | SAMN03944798 |
| 9 | First | P/S | SAMN03944801 |
| 9 | First | x/preCore | SAMN03944802 |
| 9 | Second | P/S | SAMN03944805 |
| 9 | Second | x/preCore | SAMN03944806 |
| 9 | Third | P/S | SAMN03944803 |
| 9 | Third | x/preCore | SAMN03944804 |
| 10 | First | P/S | SAMN03944807 |
| 10 | First | x/preCore | SAMN03944808 |
| 10 | Second | P/S | SAMN03944811 |
| 10 | Second | x/preCore | SAMN03944812 |
| 10 | Third | P/S | SAMN03944809 |
| 10 | Third | x/preCore | SAMN03944810 |
