# Supplementary material for: Complex Genotype Mixtures Analyzed by Deep Sequencing in Two Different Regions of Hepatitis B Virus
Source: PLoS One. 2015 Dec 29;10(12):e0144816. doi: 10.1371/journal.pone.0144816 (PMC4695080; doi:10.1371/journal.pone.0144816)
Supplement: S2 Table — These parameters were obtained for the three samples per patient analyzed: the first available at diagnosis (1st), the second before starting treatment (2nd), and the third while under treatment (3rd). (DOCX) [file pone.0144816.s010.docx]

**S2 Table**

Parameters of complexity obtained for the P/S and X/preCore region from the three samples per patient that were analyzed: the first available at diagnosis (1^st^), the second before starting treatment (2^nd^), and the third at treatment failure (3^rd^).

The table include the number of reads (reads), the number of haplotypes (Hapl), the number of mutations (NM), the number of polymorphic sites (PS), and the mutation frequency and the nucleotide diversity for the 30 samples of the study,

| **Pt** | **Samples** | **P/S region (615 to 969)** | | | | | | **X/preCore region (1596 to 1912)** | | | | | |
| --- | --- | --- | --- | --- | --- | --- | --- | --- | --- | --- | --- | --- | --- |
|  |  | **Reads** | **Hapl** | **NM** | **PS** | **Mf·10^3^** | **Pi·10^3^** | **Reads** | **Hapl** | **NM** | **PS** | **Mf·10^3^** | **Pi·10^3^** |
| **P1** | **1st** | 21,078 | 18 | 79 | 71 | 8.51 | 16.13 | 4,498 | 17 | 60 | 57 | 8,15 | 14,29 |
|  | **2nd** | 21,082 | 11 | 10 | 10 | 0.48 | 0.86 | 12,044 | 10 | 27 | 27 | 0,84 | 1,67 |
|  | **3rd** | 9,922 | 16 | 39 | 39 | 8.11 | 14.21 | 2,219 | 27 | 64 | 60 | 10,14 | 17,54 |
| **P2** | **1st** | 2,815 | 16 | 82 | 74 | 28.23 | 46.24 | 22,097 | 19 | 61 | 58 | 12.75 | 22.78 |
|  | **2nd** | 1.739 | 21 | 53 | 52 | 2.97 | 5.24 | 187,619 | 29 | 69 | 65 | 17.95 | 30.01 |
|  | **3rd*** | 474 | - | - | - | - | - | 13,127 | 24 | 72 | 68 | 6.76 | 12.48 |
| **P3** | **1st** | 26.528 | 31 | 69 | 65 | 2.31 | 4.50 | 7,293 | 43 | 73 | 67 | 17.78 | 25.44 |
|  | **2nd** | 31.945 | 19 | 13 | 13 | 1.93 | 2.97 | 77,563 | 40 | 76 | 73 | 9.64 | 16.78 |
|  | **3rd** | 6.568 | 68 | 19 | 19 | 20.37 | 25.21 | 6,375 | 26 | 70 | 65 | 13.31 | 22.34 |
| **P4** | **1st** | 4.750 | 29 | 7 | 7 | 3.23 | 4.52 | 87,532 | 28 | 68 | 65 | 11.49 | 19.40 |
|  | **2nd** | 18.377 | 5 | 4 | 3 | 0.05 | 0.10 | 15,853 | 34 | 73 | 67 | 12.46 | 21.59 |
|  | **3rd** | 14.956 | 24 | 82 | 75 | 28.69 | 39.10 | 202,070 | 19 | 55 | 53 | 3.60 | 6.41 |
| **P5** | **1st*** | 661 | 4 | 3 | 3 | 0.09 | 0.18 | 27,661 | 1 | 0 | 0 | 0.00 | 0.00 |
|  | **2nd** | 16.947 | 10 | 9 | 8 | 0.36 | 0.69 | 21,172 | 36 | 77 | 72 | 9.82 | 16.89 |
|  | **3rd** | 6.054 | 9 | 8 | 6 | 0.38 | 0.73 | 93,731 | 27 | 66 | 64 | 10.03 | 16.85 |
| **P6** | **1st** | 37.235 | 11 | 8 | 8 | 0.32 | 0.63 | 5,651 | 11 | 34 | 33 | 5.53 | 9.96 |
|  | **2nd** | 30.387 | 13 | 12 | 12 | 0.21 | 0.42 | 7,518 | 21 | 63 | 61 | 10.11 | 17.17 |
|  | **3rd** | 2.429 | 22 | 33 | 33 | 1.33 | 2.61 | 10,666 | 45 | 81 | 74 | 15.46 | 26.54 |
| **P7** | **1st** | 9.727 | 3 | 2 | 2 | 0.05 | 0.11 | 7,010 | 27 | 63 | 59 | 7.30 | 13.83 |
|  | **2nd** | 11.998 | 6 | 5 | 5 | 0.10 | 0.19 | 7,808 | 25 | 69 | 68 | 4.81 | 9.19 |
|  | **3rd** | 24.312 | 10 | 10 | 9 | 0.30 | 0.57 | 8,366 | 50 | 84 | 76 | 24.95 | 39.85 |
| **P8** | **1st** | 18.792 | 43 | 107 | 89 | 33.57 | 52.54 | 22,285 | 37 | 80 | 74 | 8.96 | 15.57 |
|  | **2nd** | 17.832 | 53 | 91 | 79 | 10.51 | 19.30 | 5,120 | 33 | 72 | 68 | 12.92 | 22.25 |
|  | **3rd** | 13.956 | 19 | 36 | 34 | 15.95 | 23.62 | 8,797 | 13 | 52 | 52 | 9.78 | 16.41 |
| **P9** | **1st*** | 453 | - | - | - | - | - | 8,867 | 17 | 59 | 57 | 13.97 | 23.43 |
|  | **2nd** | 5.649 | 56 | 26 | 24 | 3.68 | 6.03 | 15,335 | 51 | 89 | 86 | 7.44 | 13.58 |
|  | **3rd*** | 53 | - | - | - | - | - | 11,146 | 40 | 79 | 74 | 21.03 | 33.11 |
| **P10** | **1st** | 12.871 | 19 | 17 | 17 | 1.13 | 1.98 | 15,016 | 39 | 77 | 71 | 12.96 | 22.00 |
|  | **2nd** | 9.833 | 23 | 20 | 19 | 0.75 | 1.45 | 19,657 | 40 | 77 | 72 | 5.88 | 11.24 |
|  | **3rd*** | 15 | - | - | - | - | - | 10,088 | 16 | 54 | 52 | 11.64 | 20.70 |

*Samples discarded from the study of quasispecies complexity due to low coverage of the samples.
